# An Investigation into the Protein Composition of the Teneral *Glossina morsitans morsitans* Peritrophic Matrix

**DOI:** 10.1371/journal.pntd.0002691

**Published:** 2014-04-24

**Authors:** Clair Rose, Rodrigo Belmonte, Stuart D. Armstrong, Gemma Molyneux, Lee R. Haines, Michael J. Lehane, Jonathan Wastling, Alvaro Acosta-Serrano

**Affiliations:** 1 Department of Parasitology, Liverpool School of Tropical Medicine, Liverpool, United Kingdom; 2 Department of Vector Biology, Liverpool School of Tropical Medicine, Liverpool, United Kingdom; 3 Institute for Infection Biology, University of Liverpool, Liverpool, United Kingdom; National Institute of Allergy and Infectious Diseases, United States of America

## Abstract

**Background:**

Tsetse flies serve as biological vectors for several species of African trypanosomes. In order to survive, proliferate and establish a midgut infection, trypanosomes must cross the tsetse fly peritrophic matrix (PM), which is an acellular gut lining surrounding the blood meal. Crossing of this multi-layered structure occurs at least twice during parasite migration and development, but the mechanism of how trypanosomes do so is not understood. In order to better comprehend the molecular events surrounding trypanosome penetration of the tsetse PM, a mass spectrometry-based approach was applied to investigate the PM protein composition using *Glossina morsitans morsitans* as a model organism.

**Methods:**

PMs from male teneral (young, unfed) flies were dissected, solubilised in urea/SDS buffer and the proteins precipitated with cold acetone/TCA. The PM proteins were either subjected to an in-solution tryptic digestion or fractionated on 1D SDS-PAGE, and the resulting bands digested using trypsin. The tryptic fragments from both preparations were purified and analysed by LC-MS/MS.

**Results:**

Overall, nearly 300 proteins were identified from both analyses, several of those containing signature Chitin Binding Domains (CBD), including novel peritrophins and peritrophin-like glycoproteins, which are essential in maintaining PM architecture and may act as trypanosome adhesins. Furthermore, 27 proteins from the tsetse secondary endosymbiont, *Sodalis glossinidius*, were also identified, suggesting this bacterium is probably in close association with the tsetse PM.

**Conclusion:**

To our knowledge this is the first report on the protein composition of teneral *G. m. morsitans*, an important vector of African trypanosomes. Further functional analyses of these proteins will lead to a better understanding of the tsetse physiology and may help identify potential molecular targets to block trypanosome development within the tsetse.

## Author Summary

African trypanosomes are transmitted by the haematophagous tsetse vector. For transmission to occur, bloodmeal ingested trypanosomes must overcome numerous barriers imposed by the fly. The first obstacle is the crossing of peritrophic matrix (PM), a cell-free structure that protects the midgut epithelial cells from coming under attack by the hosts' digestive enzymes, aids in water retention and helps prevent harmful pathogens from establishing a systemic infection. Trypanosomes cross the tsetse PM at least twice in their development but how they do so remains to be elucidated. Despite being a recognised barrier to trypanosome infections, there is limited knowledge of the molecular components of the tsetse PM. In this study we identified nearly 300 PM proteins using two mass spectrometry approaches. Several of the identified components were peritrophins, which are a key group of glycoproteins essential for PM integrity. In addition, we detected proteins from *Sodalis glossinidius*, a commensal bacterium linked to increased susceptibility to trypanosome infection in tsetse. Our study provides the first comprehensive identification of proteins from the tsetse PM, which provides a starting point for research into potential targets for vector control.

## Introduction

The concept of blocking trypanosome development within its tsetse host has been underexplored, primarily due to a lack of understanding the molecular events involved in the vector-parasite interactions and also difficulties in accessing an established colony of tsetse flies needed to implement such studies. Tsetse (Diptera: *Glossina* spp) are the sole cyclical vectors of trypanosomes in sub-Sahara Africa. *Glossina morsitans morsitans* belong to the morsitans (savannah) group that infest huge areas of sub-Saharan Africa and hinder the progression of cattle farming over ten million square kilometres [1]. They are also the vectors of the human pathogens *Trypanosoma brucei gambiense* and *T. b. rhodesiense*, which cause debilitating and ultimately fatal diseases if left untreated. Due to evidence of emerging parasite resistance to the current frontline therapeutics [2], mammalian toxicity to treatment and no working vaccine, new disease transmission control ideas have shifted to investigating the vector-parasite interface rather than targeting parasite interactions within the mammalian host.

For successful transmission to occur, salivarian trypanosomes must overcome many immunological and physical barriers to undergo a complex migration and development in the fly. Once ingested with a bloodmeal, the bloodstream form transforms in the midgut lumen into the procyclic stage within 1–2 days post-ingestion. After a successful differentiation into procyclics, the parasites then must avoid the proteolytic attack of tsetse digestive enzymes, reactive oxygen species [3], immune peptides [4] and serum complement [5]. They do this by escaping to the ectoperitrophic space (ES) thereby crossing the peritrophic matrix (PM), an acellular secretion that lines the midgut of many insects and could be present in more than one life stage [6], [7]. After establishing an infection in the ES, the trypanosomes then colonise the proventriculus (PV) or cardia, where they continue to develop into long epimastigotes, which then cross the PM again *en route* to the salivary glands.

In general, insect PMs are believed to be multi-functional and several roles have been proposed for this structure. Most functions depend on the selective permeability of the PM, but it is generally accepted that this tissue is analogous to the mucous secretions of mammalian digestive tracts [6], [7], [8], in that it acts as a physical barrier to abrasive food particles and digestive enzymes. It has also been demonstrated that the PM acts as a biochemical barrier retaining ingested toxins [9], [10], [11], thereby preventing cell damage and lethality to the insect. Perhaps more importantly, insect PMs impose physical barriers that prevent pathogens from reaching the midgut epithelium as demonstrated in mosquito studies [12], [13], [14], and more recently, shown in two publications in *Drosophila* and *Glossina* [15], [16].

There are two types of insect PMs described: type I and type II. Many heamatophagous adult diptera and important parasite vectors such as sand flies and mosquitoes possess a type I, which is secreted once from the midgut epithelial cells. Tsetse produce a type II PM, which is present prior to taking a bloodmeal and is continually secreted by a specialised group of cells in the PV. Electron microscopy, in combination with cytochemistry and lectin binding approaches, revealed that adult tsetse possess a highly organized, 3-layered PM (∼340 nm thick) composed of glycosaminoglycans (GAGs), glycoproteins of unidentified nature and chitin (poly β-(1,4)-*N*-acetyl-D-glucosamine [GlcNAc]) fibers [8], [17]. In addition, very little is known on its overall protein composition and there is limited knowledge of the number of peritrophins that compose the tsetse PM. Until now, only Proventriculin 1 (GmmPro1) and Proventriculin 2 (GmmPro2) have been identified as putative components of the tsetse PM since these proteins are produced exclusively in the PM-secreting PV [18]. These putative peritrophins have barely been characterised, however, it is known that GmmPro2 is upregulated in susceptible tsetse lines (salmon flies) [19].

Peritrophins are structural PM proteins that are characterized by containing at least one chitin binding domain (CBD) that in turn have several conserved aromatic residues [20]. These CBDs interact with and bind chitin fibres present in the PM and other chitin containing proteins, which effectively influence PM tensile strength, elasticity and porosity, whilst the aromatic residues may bind carbohydrates. Peritrophins can also possess one or more mucin domains, reflecting the fact that they are believed to have evolved from mucins with the acquisition of CBDs [20], [21]. These mucin domains possibly act as secretory compounds that aid water retention and resist enzymatic proteolysis.

The teneral tsetse PM is the only partial physical barrier to trypanosome infection in the tsetse midgut and modifications to the PM as the fly ages may lead to a complete barrier to infection [22]. There is good evidence using electron microscopy that trypanosomes penetrate the tsetse PM [23], [24]. However, this process must be dependent on the activity of PM-degrading enzymes since the pores in the tsetse PM are approximately 9 nm in size, which are too small for procyclic trypanosomes (several microns long) to pass through [25], [26]. It is possible that proteins integral to the tsetse PM are important in infection establishment considering that parasites of other invertebrates secrete hydrolytic enzymes to degrade PM proteins in their respective hosts. To understand such strategies, a thorough revision of the composition and structure of the tsetse PM is required. This study provides the first insight into the overall protein content of the tsetse PM in an effort to understand, at the molecular level, the events involving trypanosome migration within the tsetse vector.

## Materials and Methods

### Tsetse fly maintenance and dissection of peritrophic matrices

*Glossina morsitans morsitans* (Westwood) were taken from an established colony at the Liverpool School of Tropical Medicine, which was maintained on sterile, defribinated horse blood (TCS Biosciences) at a relative humidity of 65–75% and an ambient temperature of 27°C±2°C. Experimental flies where collected at <24 hours post eclosion where they were briefly chilled at 4°C for initial sorting and kept in a 12 hour light and dark cycle in the same conditions as the colony until they were 72 hours old. All flies used in this study were teneral (unfed) male adults. PMs were dissected in sterile, chilled phosphate buffered saline solution (PBS, 140 mM NaCl, 1 mM KCl, 6 mM phosphate buffer, pH 7.4), transferred to 1.5 ml microcentrifuge tubes containing 200 µL of sterile PBS and centrifuged at 18,400×*g* for 5 minutes at 4°C. The supernatant was removed and the remaining PM pellet was washed three times in ice-cold distilled water for 10 minutes each at 18,400×*g* (to remove excess salts, non-adhered bacteria and midgut contaminants) then snap frozen and kept at −80°C until needed.

### Solubilisation of PM proteins

PMs from ∼150 tsetse were thawed and re-suspended in 150 µL of 50 mM Tris-HCl (pH 6.8), containing 8 M urea, 3% SDS and 50 mM Dithiothreitol (DTT). The sample was then sonicated in a sonicating ice-cold water bath 3 times for 5 minutes each and PM proteins precipitated with trichloroacetic acid (TCA)-acetone. Briefly, the PM suspension was mixed with 100% ice-cold acetone and 100% TCA (1∶8∶1, V/V/V respectively) and kept at −20°C for 1 hour [27]. After precipitation, the sample was centrifuged at 12,400× *g* for 15 minutes at 4°C, the supernatant discarded, and the protein pellet was washed twice with 1 ml ice-cold acetone. After the last wash, the remaining acetone was allowed to evaporate at room temperature, and the protein pellet was then re-dissolved in distilled water, mixed with Laemmli buffer [28], and heated for 10 minutes at 95°C. In a separate experiment, 150 PMs were extracted and solubilized in urea buffer as described above, and then processed for in-solution tryptic digestion as described below.

### 1D Polyacrylamide Gel Electrophoresis and staining with Coomassie Brilliant Blue G-250 for proteomic analysis

The PM protein preparation was fractionated on a NuPAGE (Invitrogen) precast 4–12% gel Tris-Bis gradient gel according to the manufacturer's recommendations. The gel was fixed overnight and the proteins were stained with colloidal Coomassie Blue G-250 (Sigma) as described by Neuhoff [29], to allow sensitive visualization and destaining of proteins prior to mass spectrometry analysis.

### Western blotting

Approximately 10 µg/lane of a preparative PM protein urea extract were fractionated on a 12% SDS-PAGE and then transferred onto BioTrace polyvinylidene diflouride (PVDF) membrane at 90 V for 30 minutes. The membrane was then incubated overnight at 4°C in blocking buffer (PBS/0.1% (v/v) Tween 20/5% (w/v) skimmed milk powder), containing 0.05% (w/v) sodium azide to prevent bacterial growth. After several washes in washing buffer (PBS/0.1% (w/v) Tween 20), separate membrane strips (containing equal amounts of protein) were probed for 1 hour at room temperature with either anti-tsetse or anti-bacterial primary antibodies: 1) mAb 4A2 (mouse anti-Proventriculin 2 (Pro2)) 1∶25 dilution, 2) mAb TBRP/247 (mouse anti-EP procyclin) 1∶10 dilution, 3) mAb 3B2 (mouse anti-lectin) 1∶2 dilution 4) mAb 1H1 (mouse anti-symbiont GroEL) 1∶20 dilution, and 5) polyclonal rabbit anti-*Sodalis glossinidus* 1∶10,000 dilution. All antibodies were a generous gift from Prof. Terry Pearson (University of Victoria, Canada). After several washes, the strips were incubated with a 1∶50,000 dilution of secondary antibody (goat anti-mouse IgG (antibodies 1–4), or mouse anti-rabbit, Thermoscientific (antibody 5) (all conjugated to horse radish peroxidase (HRPO)) at room temperature for 1 hour. After several washes, the strips were incubated with SuperSignal West Dura (Pierce, UK) peroxidase buffer and luminol/enhancer solution at a 1∶1 ratio, and developed by chemiluminescence, which continued for up to 3 hours.

### Tryptic digestion

Excised gel plugs were destained in 50% acetonitrile/25 mM ammonium bicarbonate (pH∼8), reduced for 30 minutes at 37°C with 10 mM dithiothreitol (Sigma) in 50 mM ammonium bicarbonate and alkylated with 55 mM iodoacetamide (Sigma) in 50 mM ammonium bicarbonate for 30 minutes in the dark at room temperature. Gel plugs were washed for 15 minutes in 50 mM ammonium bicarbonate and dehydrated with 100% acetonitrile. Acetonitrile was removed and the gel plugs rehydrated with 0.01 µg/µL proteomic grade trypsin (Sigma) in 50 mM ammonium bicarbonate. Digestion was performed overnight at 37°C. Peptides were extracted from the gel plugs using successive 15 minute incubations of 2% (v/v) acetonitrile, 1% (v/v) formic acid. Peptide extracts were pooled and reduced to dryness using a centrifugal evaporator (Jouan RC10-22), and re-suspended in 3% (v/v) acetonitrile, 0.1% (v/v) TFA for analysis by mass spectrometry.

For in-solution digestion, acetone precipitated PM material was solubilised with 0.1% (v/v) Rapigest (Waters Corp.) in 25 mM ammonium bicarbonate. The sample was heated at 80°C for 10 min, reduced with 3 mM DTT (Sigma) at 60°C for 10 min, and then alkylated with 9 mM iodoacetamide (Sigma) at room temperature for 30 min in the dark. Proteomic grade trypsin (Sigma) was added at a protein∶trypsin ratio of 50∶1, and samples were incubated at 37°C overnight. Rapigest was removed by adding TFA to a final concentration of 1% (v/v) with incubation at 37°C for 2 hours. The peptide samples were then centrifuged at 12,000×*g* for 60 min at 4°C to remove precipitated Rapigest. Peptides were desalted using C18 Stage tips (Thermo scientific), then reduced to dryness centrifugal evaporator (Jouan RC10-22), and re-suspended in 3% (v/v) acetonitrile, 0.1% (v/v) TFA for analysis by mass spectrometry.

### Liquid chromatography-mass spectrometry (LC-MS) analysis of tryptic peptides

Peptide mixtures, generated by in-gel proteolysis of excised protein bands from polyacrylamide gels, were analysed by reverse-phase liquid chromatography (RPLC) using an UltiMate™ 3000 LC system (DIONEX) coupled to an LTQ (Thermo Fisher Scientific) mass spectrometer. Peptides (10 µl) were injected onto a C18 column (2 µm particle size (100), 75 µm diameter×150 mm long) at nanoflow rate (300 nl/min) and separated over a 50 minutes linear chromatographic gradient. The gradient consisted of the following phases: 0–30 min, 0–50% buffer B (linear); 30–30.1 min, 50–100% buffer B (linear); 30.1–35 min, 100% buffer B; 35.1–50 min, 0% buffer B. Full scan MS spectra (*m/z* range, 400–2000) were acquired by the LTQ operating in triple-play acquisition mode. The top three most intense ions were selected for zoom scan and tandem MS by collision-induced dissociation (CID).

Peptide mixtures, generated by in-solution proteolysis, were analysed by on-line LC using the nanoACQUITY-nLC system (Waters Corp.) coupled to an LTQ-Orbitrap Velos (Thermo Fisher Scientific) mass spectrometer. Peptides (∼500 ng) were injected onto the analytical column (nanoACQUITY UPLC™ BEH130 C18. 15 cm×75 µm, 1.7 µm capillary column) at nanoflow rate (300 nl/min). The linear gradient consisted of 3–40% acetonitrile in 0.1% formic acid (v/v) over 120 min, followed by a ramp of 40–85% acetonitrile in 0.1% formic acid for 3 min. Full scan MS spectra (*m/z* range. 300–2000) were acquired by the Orbitrap at a resolution of 30,000. A data-dependent CID data acquisition method was used. The top 20 most intense ions from the MS1 scan (full MS) were selected for CID in the LTQ ion trap.

### Protein identification

Tandem MS data were searched against the *Glossina morsitans morsitans* database Glossina-morsitans-Yale_PEPTIDES_GmorY1.1.fa.gz downloaded from VectorBase (https://www.vectorbase.org/proteomes) using the Mascot (version 2.3.02, Matrix Science, Liverpool) search engine. Search parameters were a precursor mass tolerance of 10 ppm for the in-solution digest using the LTQ-Orbitrap Velos and 0.6 ppm for the lower resolution LTQ instrument. Fragment mass tolerance was 0.6 Da for both instruments. One missed cleavage was permitted, carbamidomethylation was set as a fixed modification and oxidation (M) was included as a variable modification. For in-solution data, the false discovery rate was filtered at 1%, and individual ion scores ≥30 were considered to indicate identity or extensive homology (*p*<0.05). Individual MS/MS spectra for single peptide hits with an ion score of 30 or above have been inspected manually and only included if a series of at least four continuous fragment ions were observed (Supplemental Figure S1 and S2).

Tandem MS data were also searched against the *Sodalis glossinidius* peptide database generated from the latest re-annotated coding sequences [30] using the same search engine and parameters as described above.

## Results and Discussion

**Figure 1:**
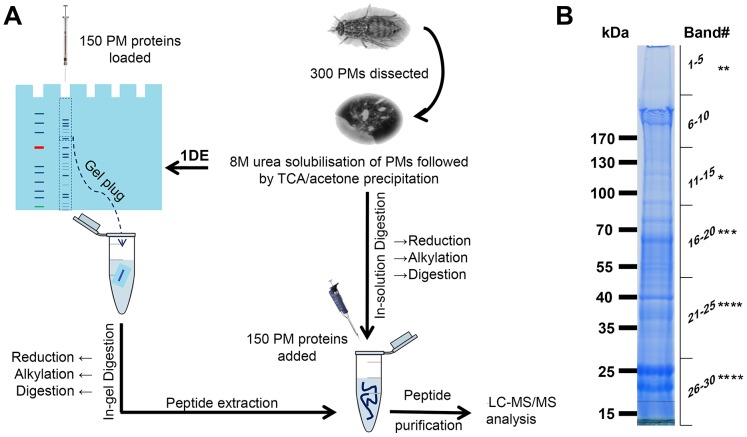
Experimental flow diagram. A total of 300 PMs were dissected and split equally into 2 tubes for urea/SDS solublisation and acetone precipitation. (A) One batch of PMs were sent directly for in-solution trypsin digestion whilst the other were fractionated on a 4–20% gradient gel. (B) 30 bands were cored from the gel from top to bottom and each gel plug was processed for in-gel trypsination mass spectrometry analysis. The position and number of asterisks indicate the regions of the gel where GmmPer66 was identified.

Approximately two batches of 150 male teneral (young, unfed) flies were used to determine the proteome of *G. m. morsitans* PM (one batch of 150 were used for in-gel analyses, the other 150 were used for in-solution analyses). The PM samples were extensively washed before homogenisation in Tris-HCl buffer containing 8M urea and 3% SDS, and cold-precipitation with acetone/TCA (Figure 1). The urea/SDS homogenisation produced a higher yield of proteins (as judged by analysis of Coomassie blue-stained SDS-PAGE gels) compared to the extraction with 3% SDS-DTT alone followed by either mild TFA hydrolysis (40 mM TFA for 30 minutes at 100°C, to break mild acid sensitive Asp-Pro or Gly-Pro bonds [31] or anhydrous trifluoromethanesulfonic acid (TFMS) (for chemical deglycosylation of proteins) [32] (not shown). In addition, we did not find significant differences in the pattern of bands on SDS-PAGE between PM samples extracted from either teneral or 15 day old, bloodfed flies, or in samples from either sex. Nevertheless, to avoid contamination from horse blood proteins in our mass spectrometry analyses and considering their high susceptibility to a trypanosome infection, we decided to analyse only PMs extracted from teneral male flies.

In order to increase the number of proteins identified, we decided to use two approaches. One sample (containing ∼150 PMs) recovered after acetone/TCA precipitation was fractionated on a 4–20% NuPAGE precast gel (Figure 1B) and the excised bands were digested with trypsin and processed for LC-MS/MS analysis (below), whilst another batch of ∼150 PMs were directly trypsinised in-solution after urea solubilisation and precipitation as above, before mass spectrometry analysis. Furthermore, peptide sequences from both analyses were BLAST searched against the genomes of *G. m. morsitans* and the midgut symbiont *S. glossinidus*.

### In-gel analysis

After colloidal Coomassie blue staining, many proteins with apparent molecular masses from ∼21 kDa up to >200 kDa were visualised, although a slight smeariness in a number of bands indicated the presence of highly modified proteins. Since many proteins do not stain with Coomassie blue (e.g. mucins and peritrophins due to their high negative charge and acidity [33]), we decided to increase coverage by slicing the stained gel lane in 30 pieces from top to bottom (Figure 1B). The individual bands were then excised, the proteins in-gel trypsinised and analysed by LC-MS/MS (Figure 1 and Table 1). This approach provided useful information regarding the relative abundance and masses of the different proteins (Table 1 and S1), whilst the in-solution analysis (below) increased the number of proteins identified.

**Table 1:** List of the most abundant proteins detected by mass spectrometry from in-gel analyses of the peritrophic matrix of teneral *G. m. morsitans*.

VectorBase ID #^1^	Protein name	Occurrence	Main feature(s)
GMOY002708	GmmPer66/Peritrophin-like	14^2^	3 CBDs, 2 *N*-glycs.
GMOY009892	Dynein AAA+ ATPase	13	AAA+ ATPase domain
GMOY011773	Perlecan	11^3^	Ig set I-domain, ConA-like lectin
GMOY001776	Actin	9	Actin-related domain
GMOY005703	Myosin heavy chain	9	Myosin motor domain
GMOY007063	Midgut trypsin	8^4^	Trypsin-like serine peptidase domain, SP
GMOY009248	Lamin	5	Lamin tail domain
GMOY004611	Vesicular transport factor dp115	4	Armadillo repeats
GMOY006294	Glutamate semialdehyde dehydrogenase	4	Uridylate kinase, aldehyde dehydrogenase
GMOY009756	Trypsin/Proventriculin3 (Pro3)	4^5^	Trypsin-like serine peptidase domain, SP
GMOY000153	Chitinase Chit1 precursor	3	1 CBD (PAD), glycosidase
GMOY003579	Na/K transport. ATPase	3	P-type ATPase, 4 TMD
GMOY005442	Lipophorin	3	Vitellogenin lipid transport domain, SP
GMOY009587	Proventriculin2 (Pro2)	2^6^	Partial CBD (PCD)
GMOY003306	TsetseEP	2^7^	Tsetse-specific, immunity, SP
GMOY000672	Serine protease 6	2	Trypsin-like Ser peptidase domain, SP
GMOY009757	Serine type endopeptidase	2	Trypsin-like serine peptidase domain
GMOY011520	Alanyl aminopeptidase	2	Peptidase M1, DUF domain, *N*-glyc.
GMOY002421	Chaperonin-60 kDa	2	Cpn60/TCP-1, GroEL-like apical domain
GMOY011805	Choline O-acyltransf.	2	1 TMD, *O*-glyc.
GMOY007524	Hypothetical	2	*O*-glyc.
GMOY007847	Hypothetical	2	unknown
GMOY008627	Hypothetical	2	Unknown, SP
GMOY008757	Hypothetical	2	Unknown
GMOY001198	Hypothetical	2	Ig I-set domain

1 VectorBase *G. m morsitans* database version GmoY1.1, 2013. Glossina-morsitans-Yale_PEPTIDES_GmorY1.1.fa.gz.

2 GmmPer66 was found in bands 1, 3, 12, 16, 18, 20 to 24, 26 and 28 to30. CBD (PAD type).

3 Identified from bands 4, 7 to 15 and 24.

4 Found in bands 19 and 24 to 30.

5 Identifed from bands 24 to 26 and 28.

6 Identified from bands 29 and 30.

7 Found in bands 23 and 24, which is consistent with the protein's predicted *Mr* of 37.5 kDa.

SP: Signal Peptide.

TMD: Transmembrane Domain.

The most visually abundant proteins on the gel were a doublet migrating with relative molecular masses around 26 and 21 kDa (bands 27 and 28, respectively), which were identified as midgut trypsins. However, the most abundant and frequent hit in many of the bands analysed was a new type of peritrophin herein referred to as GmmPer66 (discussed below in the peritrophins section). In addition, GmmPro2, another known peritrophin-like protein that is produced in the PV [18] and the immunomodulatory TsetseEP protein [34], [35], were also detected in several bands (Table 1 and S1). The possible significance of the high occurrence of these proteins is discussed below. Furthermore, other peptidases, including GmmPro3 [18], one serine peptidase and one putative metalloprotease, one chitinase, and several uncharacterised/conserved/hypothetical proteins were also found. Not surprisingly, abundant hits were also found for metabolic proteins, transporters and extracellular matrix proteins. The significance of the presence of these proteins is discussed below.

### In-solution analysis

**Figure 2:**
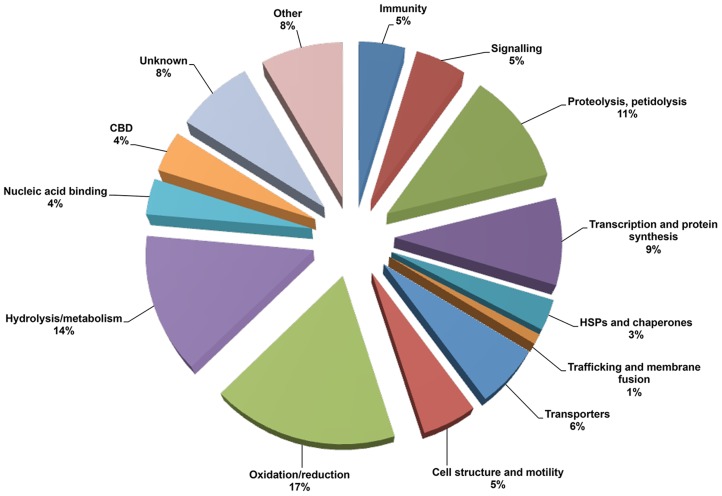
Categorization of the *G. m. morsitans* peritrophic matrix proteins as identified through LC-MS/MS according to their putative functions.

In order to increase detection of PM proteins, a urea/SDS extract was also trypsinised in-solution and directly analysed by LC-MS/MS. A minimum of 195 *G. m. morsitans* proteins were identified. Only those with an ion score cut off of 30 or above were considered, with the majority of them having 2 or more identifying peptides and annotated on the VectorBase database (version GmorY1.1, 2013) and *S. glossinidius* genome. Proteins were classified and grouped by functional classifications (Figure 2), according to their GO terms and domain features as predicted by ExPASy Prosite, VectorBase and EMBL-EBI InterProScan. Hypothetical proteins were classified based on the presence of family domains.

The majority of tsetse proteins (92%) fit into 13 of the categories, whereas 15 proteins (8%) could not be assigned to any category. However, all of these unknown proteins had orthologues in several insects and insect vectors, most of which had either no description or were described as conserved hypothetical proteins, suggestive of being ubiquitous among insects. Of the 195 proteins, 28 contained a predicted signal peptide (SP), 26 were found to contain a transmembrane domain (TM) only, 16 had both a predicted signal peptide and at least one TM and the remainder (125) were predicted to be soluble (i.e. neither SP nor TM domain). Interestingly, one of the most abundant hits corresponded to GmmPer66. Two other novel peritrophins were also discovered: GmmPer12 (GMOY011810) and GmmPer108 (GMOY007191).

### Validation of some of the proteins identified by western blotting

Western blot analysis was performed in order to validate some of the protein hits identified in both the in-gel and in-solution digested samples. Tsetse PMs were dissected, washed and solubilised with urea/SDS, processed for Western blotting and ∼10 PMs per lane probed separately with several anti-tsetse and two anti-*Sodalis* antibodies.

**Figure 3:**
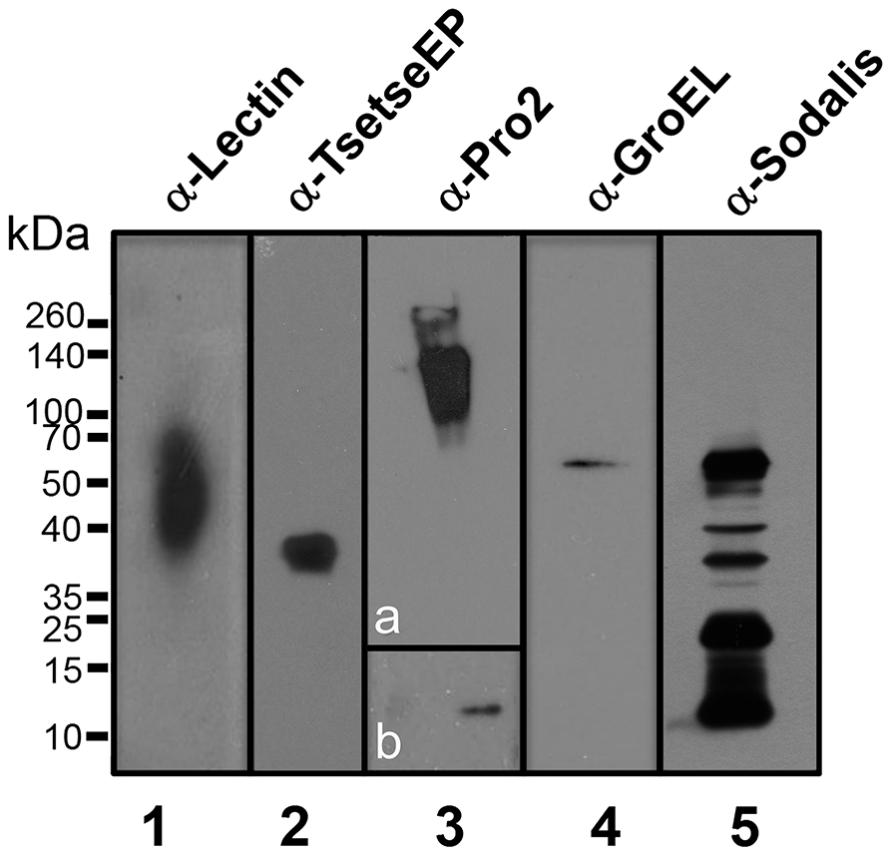
Western blotting analysis of tsetse PM proteins. Homogenates from ∼10 PM equivalents were loaded per lane and after transferred to PVDF separately probed against an anti-tsetse lectin antibody (lane 1), anti-procyclin mAb 247 (lane 2), anti-Pro2 mAb 4A2 (lane 3), polyclonal anti-GroEL mAb 1H1 (lane 4) and polyclonal anti-*Sodalis* (lane 5) and developed by chemiluminiscence. Developing continued for 30 seconds (lane 1, 2, 3a, 4 and 5) or up to 3 hours (lane 3b).

As shown in Figure 3, we were able to confirm by Western blotting the presence of one C-type lectin (lane 1), TsetseEP protein (lane 2) and Pro2 (lane 3). In addition, the presence of symbiont proteins were confirmed using an anti-GroEL monoclonal antibody (lane 4), which cross-reacts with the GroEL of *Wigglesworthia glossinidia* and *Sodalis glossinidius*. To confirm that *S. glossinidius*, and not *W. glossinidia*, was isolated with the PM, an anti-*Sodalis* polyclonal antiserum was used (lane 5). This antiserum recognizes a suite of *S. glossinidius* proteins that produces a characteristic banding profile, including GroEL (*Mr*∼60 kDa) (Haines, L., unpublished) (Figure 3).

### Analysis of the new tsetse peritrophins

**Figure 4:**
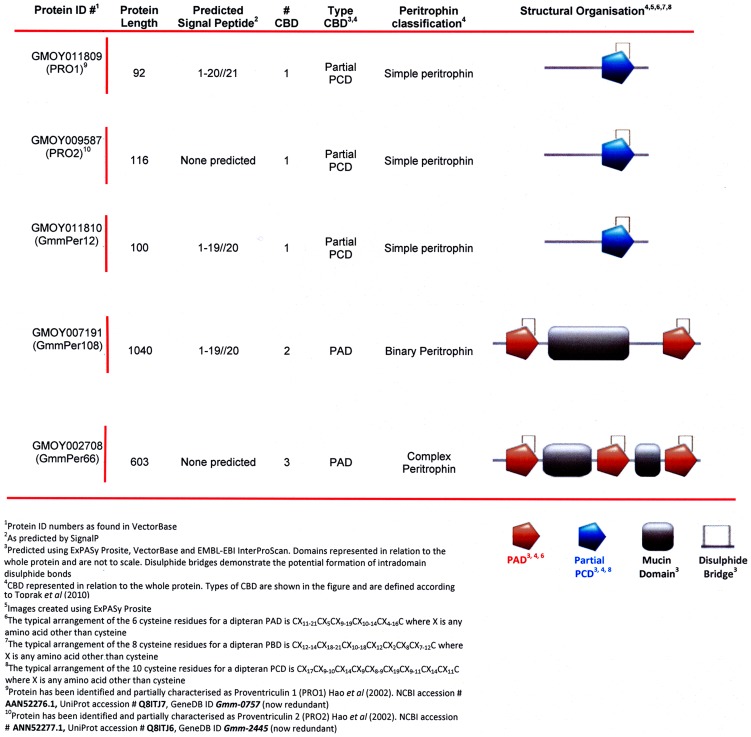
Classification and partial characterization of *G. m. morsitans* peritrophic matrix (PM) peritrophin and peritrophin-like proteins, containing 1 or more chitin binding domains (CBD), as identified by LC-MS/MS.

**Figure 5:**
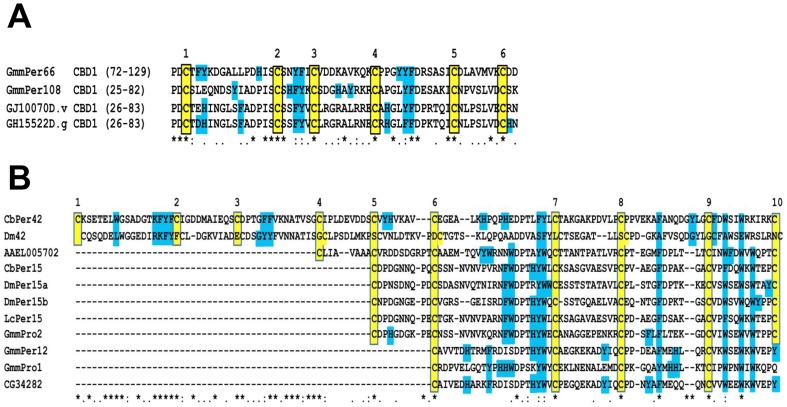
Chitin Binding Domains of tsetse peritrophins as identified by LC-MS/MS aligned against other representative domains from putative Dipteran peritrophins. The positions of the domains within the sequence are shown next to their respective protein IDs for panel A only. The 10 and 6 conserved cysteine residues of a PCD (A) and PAD (B) respectively, which are potentially involved in disulphide bridge formation, are indicated by yellow boxes and asterisks (*). Note the partial PCD of many peritrophins including those in *Glossina*. The numbers above the cysteine residues depict the order of cysteines in the CBD. The conserved aromatic residues, characteristic of chitin binding domains, are denoted by blue boxes and may be involved in carbohydrate binding. Identity of amino acid residues is depicted according to ClustalW.

In total, five peritrophins were identified by mass spectrometry analysis from both in-solution and in-gel digestion and as such, has more than doubled the number of previously reported peritrophins from the *Glossina* PM (Figure 4). Both GmmPro1 (GMOY011809) and GmmPro2 (GMOY009587) are known to be synthesised in the tsetse PV and secreted during the formation of the PM [18]. The remaining three are novel, and this study is the first to positively identify them as being PM constituents. GmmPer12 (GMOY011810) is a small peritrophin of 100 aa with a predicted molecular mass of ∼12 kDa and has a partial Peritrophin C Domain (PCD). Originally, the PCD was thought to consist of 6 conserved cysteine residues [36] with the domain spanning 68–70 residues. Only recently has the PCD been shown to be composed of 120–121 residues and have a motif of 10 conserved cysteines [37] consisting of CX_17_CX_9–10_CX_14_CX_9_CX_8–9_CX_19_CX_9–11_CX_14_CX_11_C and those peritrophins thought to have a full PCD are now categorized as having partial domains. Partial domains may have come about through multiple duplication events or proteolytic degradation of full length proteins whilst retaining the ability to bind chitin. This proteolysis may occur before or after such CBD proteins have been incorporated into the matrix. Some partial CBDs have been shown to have trypsin and chymotrypsin cleavage sites embedded within the CBDs [38] suggesting these proteins are highly resistant to proteolysis owing to the folded nature of their structure through disulphide bond formation. GmmPer12 has a PCD of 4 conserved cysteine residues similar to that of GmmPro1 and GmmPro2 and is analogous to peritrophins found in other insects such as LcPer15, a peritrophin found in the PM of the sheep blowfly *Lucilia cuprina* [39] (Figure 5). A predicted signal peptide between residues 19/20 suggests that GmmPer12 is secreted into the PM after synthesis. GmmPro1, GmmPro2 and GmmPer12 are related to the peritrophin-15 family of proteins, integral proteins from the PMs of many insects [40]. This protein family is suggested to associate with the PM by binding to the ends of chitin fibrils giving structural support and preventing exochitinase action. The lack of *N*- and *O*-glycosylation on these 3 peritrophins supports this assumption. However, their intact forms appear to be absent in the PM, suggesting these three peritrophins are degraded and incorporated into the PM as partial fragments that have retained their ability to function as a chitin-binding domain. The updated *Glossina* VectorBase genome annotation has revealed that GmmPro2 is not 93 amino acids as previously reported [18] (AAN52277.1), but instead has an extension at its N-terminus making the protein 116 amino acids long. This is perhaps evidence that at least GmmPro2 (and probably also GmmPro1 and GmmPer12) have evolved from a larger protein containing many CBDs.

### Non-structural PM proteins

The majority of the proteins identified were hydrolytic enzymes including chitinases, amylases, exopeptidases and digestive enzymes such as trypsin. Although these may be midgut secreted proteins and only transiently associated with the PM, studies have shown these enzymes remain in the PM even after repeated washes and extraction with strong denaturants [14]. A tsetse Chitinase (Cht1) was identified from both in-gel and in-solution analyses. Chitinases have been found associated in the PM of lepidopteran larvae where they are involved in the larvae moulting process [45] and are also found in the PM of adult mosquitoes where their role is less understood [46]. It has been suggested that during insect growth and development, chitin containing structures require the capacity to undergo remodelling and modification in order to allow for growth, maturation and repair [47]. This is especially true under certain conditions such as periods of moult or starvation where PM production can stop. In order for this to happen, tissue specific chitinolytic enzymes and chitin synthases are produced periodically. Chitinases are important in both the shedding of the cuticle during moults and growth and for the degradation and turnover of both the PM and trachea [47]. The fact that chitinases have now been identified in the tsetse PM suggests that PM chitinases in adult tsetse may be involved in degradation of the chitin fibrils thereby modifying the thickness, porosity and tensile strength of the PM during its extension along the length of the midgut.

As expected, a large percentage of proteins (11% from the in-solution digestion) were digestive enzymes such as trypsins, chymotrypsins, peptidases and serine proteases. Their identification may simply reflect transit across the PM to the endoperitrophic space in response to a blood meal, but given the fact that the flies used in this study did not receive a blood meal, it is possible that these enzymes are directly interacting with the PM in anticipation of feeding. These findings add to the reputable evidence that the PM improves digestion by concentrating the food bolus and filtering out indigestible components [48]. One serine protease, Proventriculin 3 or GmmPro3, previously shown to be expressed in the tsetse PV, was also identified in this study suggesting that it might be physically associated to the PM. GmmPro3 is homologous to proteins of the serine protease S3 family and shares similarities with serine proteases from other haematophagus insects such as *Stomoxys calcitrans* and *A. gambiae* [18]. One serine protease inhibitor (Serpin), GmmSpn4, was also identified from the PM suggesting that serine proteases and serpins have a co-relationship involving blood meal digestion and may also modulate the PM structure until it is fully formed. Finally, proteases may also protect the passage of pathogens through the PM. In fact, the surface of procyclic trypanosomes gets “re-shaped” due to extensive proteolysis of the main surface glycoproteins, procyclins [49], which partially may occur during PM crossing.

### Proteins involved in immunity

From the in-solution analysis, 9 proteins (∼5%) were identified as being involved in host-parasite interactions. These proteins were mainly C-type lectins (CTLs), whose presence in the PM was corroborated by Western blotting (Figure 3). CTLs are Ca^2+^-dependent glycan binding proteins and play important roles in insect defence [50]. Carbohydrate binding events mediate a range of processes including cell/cell interactions, cell adhesion and are involved in cell apoptosis. They are also capable of recognizing pathogen-associated molecular patterns in a variety of microbes and in tsetse it has been suggested to be involved in the initial elimination of trypanosome burden by agglutinating parasites [51], although so far no experimental evidence has proved this.

Interestingly, from the in-gel analyses there were many hits for basement membrane-specific heparin sulfate proteoglycan core protein (perlecan). Perlecan is a large proteoglycan with a multitude of diverse domains [52]. These domains bind to and cross-link numerous extracellular components and cell surface molecules. The N-terminal domain consists of ∼195 aa and contains three Ser-Gly-Asp attachment sites for large heparin sulfate chains or, occasionally, chondroitin sulfate. There is microscopy evidence showing that the *G. m. morsitans* PM contains glycosaminoglycan's (GAGs) in the layer facing the ectoperitrophic space (epithelium side) [53], suggesting that this may be the location where perlecan may accumulate after secretion. Other domains include immunoglobulin, laminin and low-density lipoprotein (LDL) receptors that contain multiple cysteine residues able to form disulphide bridges. Perlecan also has an epidermal growth factor (EGF) domain, which is involved in ligand-recognition and protein-protein interactions. It is possible that identification of this protein is due to basement membrane contamination, however, if perlecan is a true PM protein, this may explain why proteins such as collagen, actin, lamin, laminin and fibronectin are found in a number of PM proteome studies [54], [55]. It would be interesting to determine the exact place of perlecan synthesis.

TsetseEP protein was also identified in both the in-gel and in-solution analyses. This is a unique tsetse protein of *Mr*∼36 kDa, which contains a characteristic extended glutamic acid-proline (EP) repeat domain at the C-terminus. Interestingly, its structure resembles that of the *T. brucei* EP-procyclins [31], [34], [56]. Studies have shown that TsetseEP probably acts as an antagonist to trypanosome infection [34]. TsetseEP is also highly upregulated in flies that have been challenged with gram-negative bacteria, which would suggest this protein may have an immunoprotective role [44]. The finding of TsetseEP in our analyses is intriguing. Although secretion of this molecule is enhanced by the presence of pathogenic microorganisms and it contains a lectin domain that may directly interact with pathogen's surface glycans, its elevated production during a midgut infection may also contribute to PM thickening, thus creating a stronger protective barrier. In *Drosophila*, there is genetic evidence showing that the PM structure changes in the presence of pathogenic bacteria [11], [15]. In addition, an interesting recent work has shown that the *Glossina* PM becomes thinner in aposymbiotic flies (i.e. lacking a midgut microbiome), which in turn increases PM permeability and allows an “easier” passage of trypanosomes through the PM [16]. Thus, although it remains to be determined how the structure of the tsetse PM changes in response to either pathogenic or non-pathogenic organisms, it may be possible that TsetseEP has a role in PM remodelling.

Tsetse antigen 5 (Tag5) was identified from the in-solution analysis. This protein of 259 amino acids is related to the large Crisp-Antigen 5 Plant pathogenesis protein families that are found in a huge diversity of organisms [57]. Mostly found in saliva of many insects, these proteins share a core sequence of approximately 200 amino acids that are responsible for their multiple functions. Antigen 5 has been proven as a potent venom allergen in hornets, wasps and fire ants and causes allergic reactions in humans [58], [59]. Although primarily found in the salivary gland tissue of tsetse, it is reported to be expressed in the PV and midgut tissues [60]. A related protein in *Drosophila*, Antigen 5 related (Agr), is also expressed in the PV of both larvae and adult flies [61], [62]. Tag5 has also shown to be upregulated in a susceptible strain of tsetse (salmon flies) [19]. Tag5 may be a true constituent of the tsetse PM and as such may have a bearing on the digestion of the bloodmeal as studies have shown Tag5 prevents homeostasis [63]. As tsetse take up to 3 days to digest a bloodmeal, it is possible that the presence of Tag5 in the PM prevents the ingested bloodmeal from clotting quickly, thus aiding and facilitating digestion.

Another protein identified and involved in immunity was glycoprotein CD36, whose family members are conserved within mammals and have many representative orthologues in insects. They have a variety of functions including lipid transport, immune regulation, homoeostasis and adhesion. One function of CD36 is as a scavenger receptor, which recognizes molecular patterns presented by bacteria, pathogens and viruses and also pathogen infected cells [64], [65]. An ortholog of CD36 in *C. elegans*, CO3F11.3, is responsible for mediating host defences against fungal infection by stimulating the production of cytokines [66]. As a PM constituent, CD36 may have multiple roles from anti-homoeostasis to immune system mediation possibly involved in initial clearance of pathogens. In addition, this protein is highly resistant to proteolysis, which would be favourable given its putative location.

Hemomucin, a 61.7 kDa protein containing extensive *O*-glycosylation at its C-terminus was also identified. It contains a domain showing strictosidine synthase, which is a key enzyme in alkaloid biosynthesis. Alkaloids are important in the immunity of plants and have been shown to be secreted in the venom of the fire ant where they act as potent inhibitors of bacteria [67]. Hemomucin from *Drosophila* proved likely to be involved in induction of antibacterial effector molecules after showing affinity for the snail lectin (*Helix pomatia* hemagglutinin A). This protein was found to be expressed in the PV, suggesting that it may be incorporated into the PM after synthesis [68].

### Other proteins

Proteins involved in stress response (oxidation and reduction) and protein folding (heat shock and chaperones) comprises a total 20% of the detected proteins. Some of these proteins may originate from the layer of epithelial cells that is in close proximity with the PM of teneral (unfed) flies. However, they may have a role in detoxification. Bloodmeal digestion leads to the rapid production of reactive oxygen species (ROS), due to the breakdown of red blood cells, which causes the release of haem and iron. Accumulation of free haem leads to oxidative stress and these oxidation/reduction proteins are needed to detoxify the midgut environment [69]. It has been demonstrated in female *Aedes aegypti* that the PM of these insects are capable of binding haem during bloodmeal digestion as shown by histochemical studies [9] and a subsequent study has shown that at least one PM protein, the peritrophin AeIMUCI, is responsible for this interaction [10]. Haem-regulatory motifs (HRM) have also been found in peritrophins from 2 species of sandfly, *Phlebotomus papatasi* and *Lutzomyia longipalpis* [11].

### *Sodalis* proteins

A total of 27 *S. glossinidius* proteins were identified (Table S2 and Table S3). Given that *Sodalis* proteins have been identified within the PM and their presence verified by Western blotting (Figure 3) suggests that secondary symbionts are intimately associated with the tsetse PM. Alternatively, these proteins may be secreted and incorporated into the PM, and thus they may have a functional role. The majority of these proteins were found to relate to metabolic activities within the bacteria. It has been well documented that *Sodalis* are important for many aspects of tsetse metabolism for example, cofactor and vitamin synthesis to compensate for the restricted diet of blood-meals [70]. Genes encoding biotin, lipoic acid, molybdenum cofactor, riboflavin and folic acid have all been found to be present in the genome of *Sodalis glossinidius* [71].

One interesting protein identified by mass spectrometry analysis was the *Sodalis* putative chitinase (Accession No SG1474). Studies have shown that when flies harbour a high density of *Sodalis*, they are more susceptible to trypanosome infection, thus it is entirely feasible to assume that these endosymbionts confer susceptibility to tsetse [72], [73], [74]. One possible explanation for this is that *Sodalis* may degrade chitin fibrils that comprise the tsetse PM, effectively remodelling it and providing an opportunity for trypanosomes to penetrate [72], [75], [76]. The primary carbon source during the growth of *Sodalis* is *N*-acetyl-β-D-glucosamine (a monomer of chitin), which it produces from the breakdown of chitin using a secreted chitinase. Given that trypanosomes have no chitinase activity, it is reasonable to speculate that *Sodalis* breaks down PM chitin, leaving the PM vulnerable and unknowingly facilitating trypanosome crossing (reviewed in [77]). In addition, the prevalence of trypanosome infection is highest when the fly is young and the PM is not yet fully formed. Proteins containing CBDs such as the peritrophins may have not yet been fully incorporated into the PM leaving the ends of PM chitin fibrils exposed. This may be the critical time point of chitinase activity, thereby degrading the PM and allowing trypanosomes to break through. Other parasites like *Brugia malayi*, *Leishmania spp* and *Plasmodium spp* secrete chitinases and proteases to degrade the proteins within the chitin meshwork and allow penetration of the PM [78], [79], [80]. Although the quantity of chitin has yet to be measured in the *G. m. morsitans* PM, the lack of chitinase expression in trypanosomes suggests that the chitin content of the tsetse PM may be probably low as reported in *Lucilia cuprina* larvae (which also expresses a type II PM). Therefore, the tsetse PM chitin may not a real barrier to trypanosome infection [81].

### What does the *G. m. morsitans* PM proteome tell us about its possible architecture?

Contrary to type I PMs, there is no molecular model representing the architecture of type II PMs. In the case of the *Glossina* PM, it is challenging to predict a model considering that it is composed of three layers [53] (each one of different thickness and probably also in composition) and because of the lack of EM localization of major peritrophins. However, based on its high abundance, number of CBDs and mucin domains, we hypothesise that GmmPer66 may play an essential role in interconnecting chitin fibres with other GmmPer66 monomers and/or other PM peritrophins, like GmmPer108 (with 2 CBDs and 1 mucin domain) or Pro2 (with one CBD and several *O*-glycosylation sites). As suggested for other highly glycosylated molecules, the *O*-glycans from these peritrophins may serve to protect the PM from protease attack and retain water thus allowing the selective trafficking of molecules between the lumen and the ectoperitrophic space. It is conceivable that other peritrophins are also part of the tsetse PM, but their identification by MS was missed due to their resistance to trypsin. In fact, the *G. m. morsitans* genome contains a minimum of 41 peritrophins in the (Attardo, G. *et al.*, in preparation).

### Conclusion

The study presented here has given a comprehensive overview of the main proteins that make up the tsetse PM identified using mass spectrometric techniques. Identification of at least 209 proteins from in-solution analysis and many more from in-gel analysis has provided a foundation of knowledge for which there is potential to expand. The identification of 3 novel peritrophins has expanded the list of known tsetse PM peritrophins from 2 to 5. In addition, the unique banding pattern of one of these peritrophins, GmmPer66, has provided us with useful insights into how their putative degree of crosslinking and how they are potentially incorporated into the PM. Although the quantity of chitin in the PM of *G. m. morsitans* has yet to be confirmed, the lack of chitinase activity in procyclic trypanosomes would suggest that the chitin component of the tsetse PM is extremely low and that chitin is not a real barrier to infection, proposing that the PM is composed mainly of glycoproteins rather than chitin. Therefore, a direct degradation of integral proteins may provide a pathway for trypanosome invasion through the PM. We are currently investigating candidate trypanosome proteases that may be participating in PM degradation. However, for proteases to act, glycosidases must first remove glycans (i.e. chitin and GAGs). Therefore, it is intriguing that procyclic trypanosomes do not express the glycosidases to degrade any of these complex sugars. Alternatively, some of the PM-degrading glycosidases may be supplied by bacterial symbionts present in the tsetse midgut. With the completion of the *Glossina* genome project, a collaborative effort involving the VectorBase community and the Sanger Centre, there is great potential to reveal novel concepts about type II PMs. Insects with a type II PM are often more refractory to infection that those with a type I PM such as mosquitoes and sand flies. Whilst huge efforts have gone into researching larval type II PMs, this is the first study to concentrate on the protein composition of the adult type II PMs from an insect vector.

The mass spectrometry proteomics data have been deposited to the ProteomeXchange Consortium (http://proteomecentral.proteomexchange.org) via the PRIDE partner repository [82] with the dataset identifier PXD000594 and DOI 10.6019/PXD000594

## Supporting Information

Figure S1 In-gel single hit validation.
(PPT)

Figure S2 In-solution single peptide hit validation.
(PPT)

Table S1 List of *Glossina morsitans morsitans* peritrophic matrix proteins from in-gel digestion analysis (ion score cut off of 30).
(DOC)

Table S2 List of *Sodalis glossinidus* proteins associated with the *Glossina morsitans morsitans* peritrophic matrix.
(DOCX)

Table S3 *Glossina morsitans* Mascot data.
(XLS)

Table S4 *Sodalis glossinidius* Mascot data.
(XLS)
