# Supplementary material for: An Investigation into the Protein Composition of the Teneral Glossina morsitans morsitans Peritrophic Matrix
Source: PLoS Negl Trop Dis. 2014 Apr 24;8(4):e2691. doi: 10.1371/journal.pntd.0002691 (PMC3998921; doi:10.1371/journal.pntd.0002691)
Supplement: Figure S1 — In-gel single hit validation. (PPT) [file pntd.0002691.s001.ppt]

## Slide 1
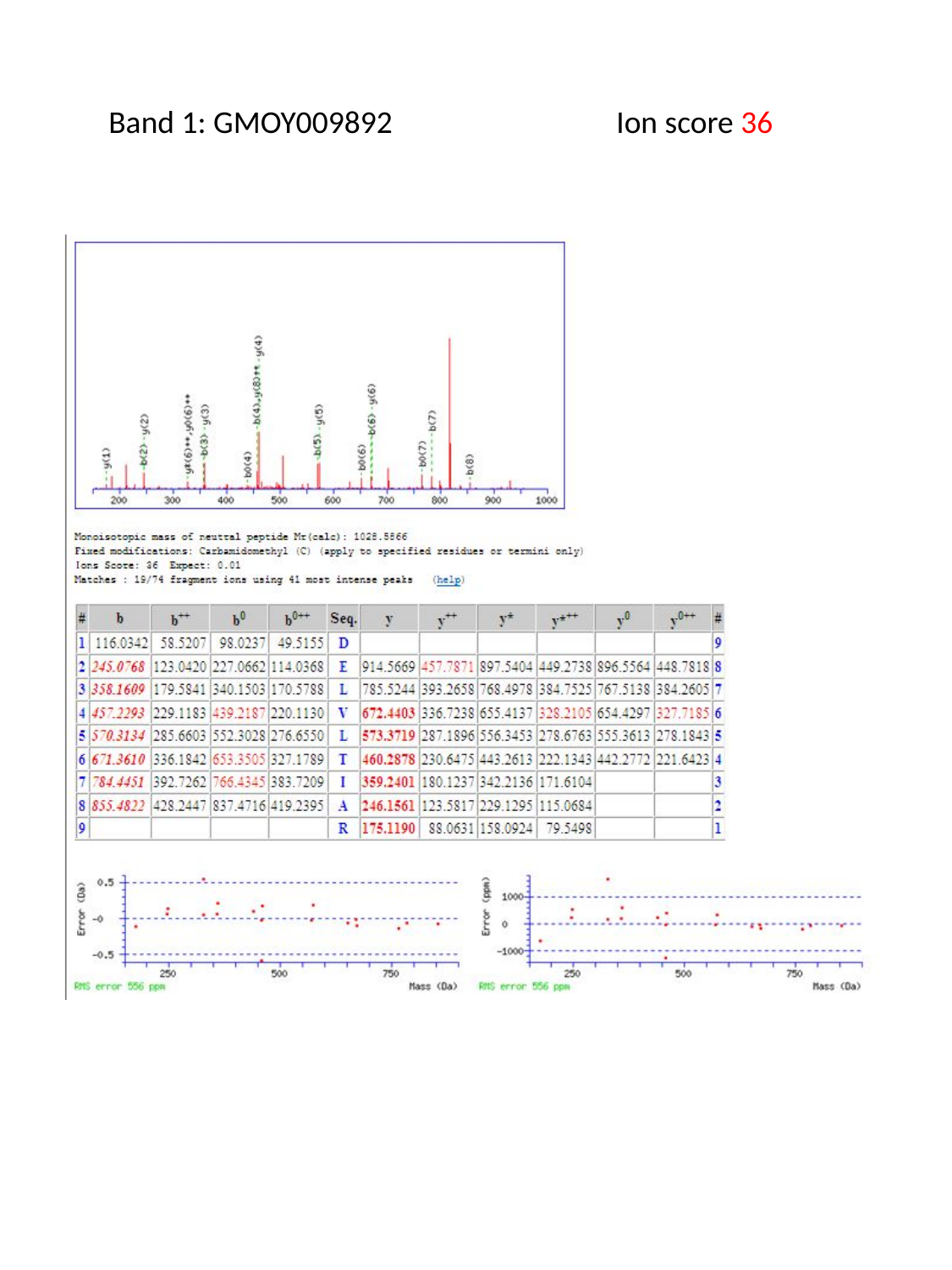

Band 1: GMOY009892 		Ion score 36

## Slide 2
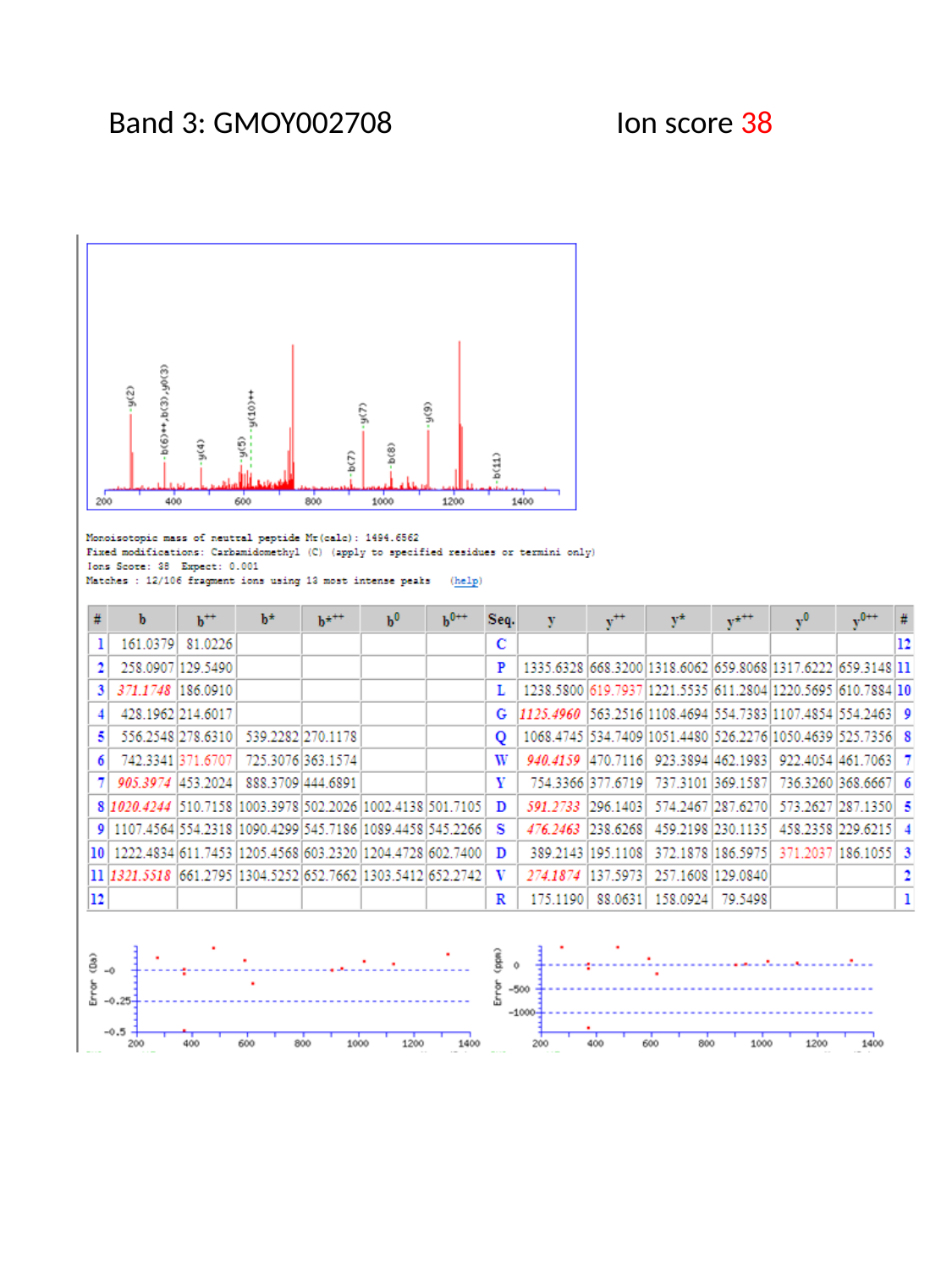

Band 3: GMOY002708		Ion score 38

## Slide 3
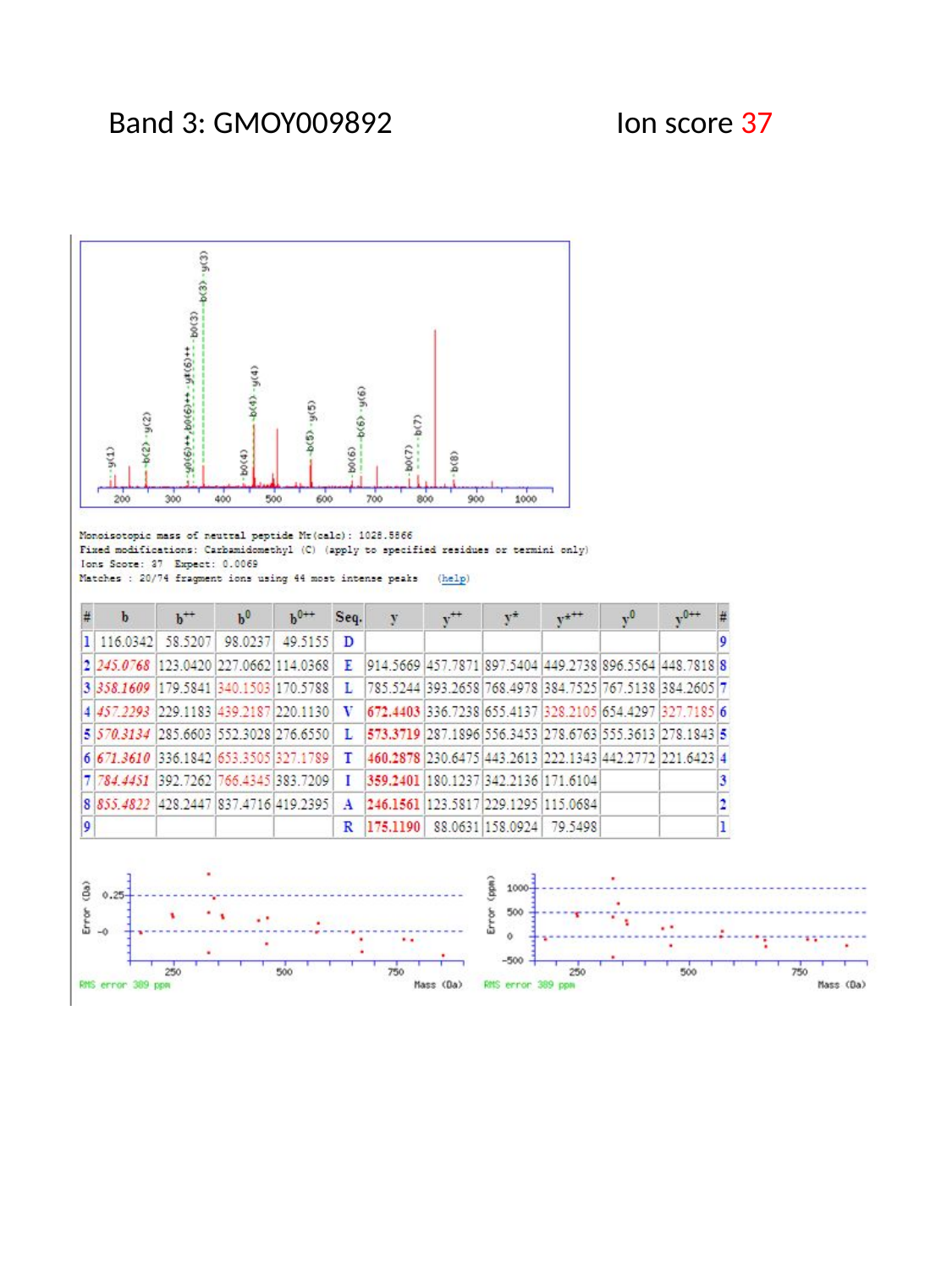

Band 3: GMOY009892		Ion score 37

## Slide 4
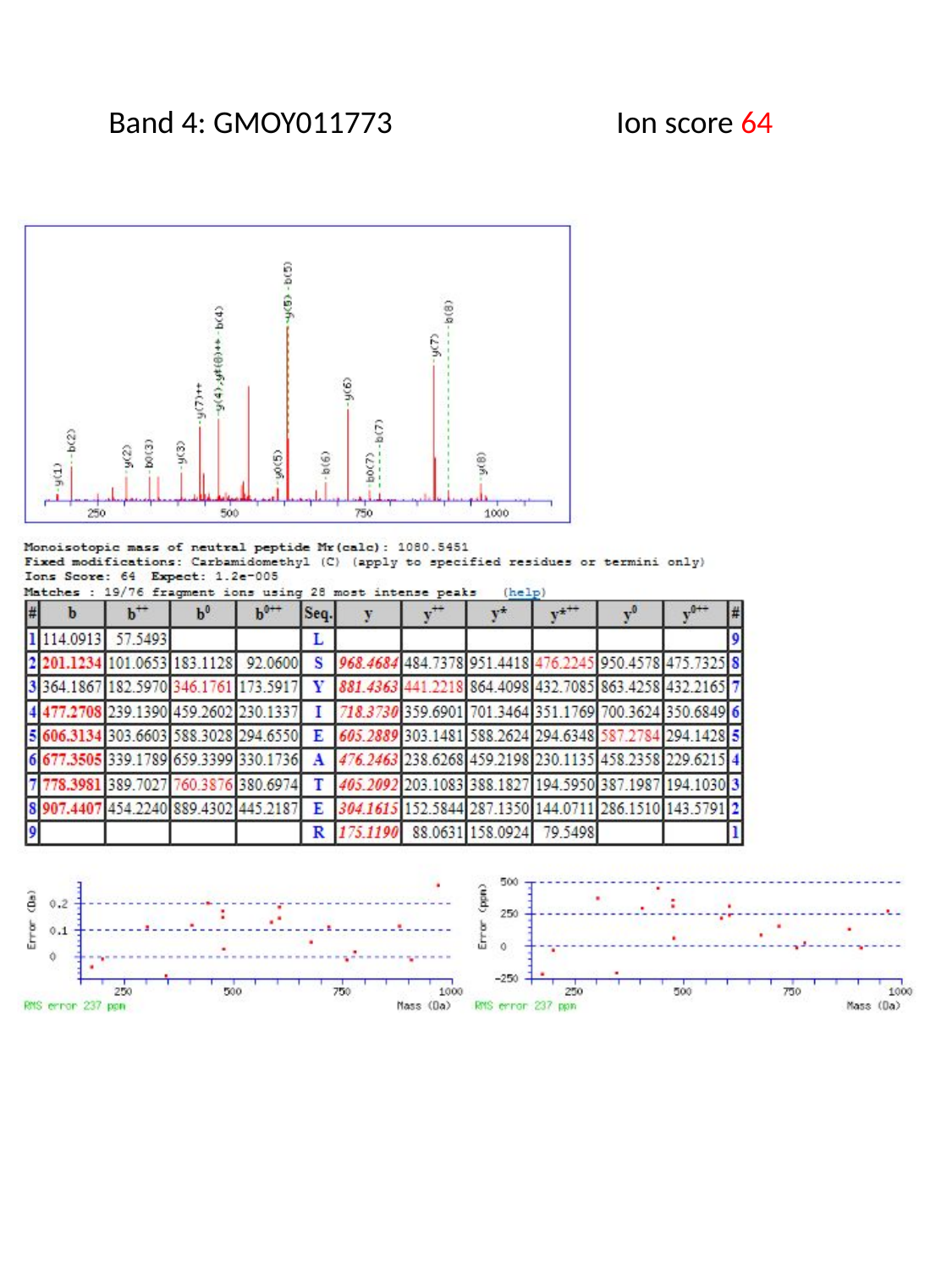

Band 4: GMOY011773 		Ion score 64

## Slide 5
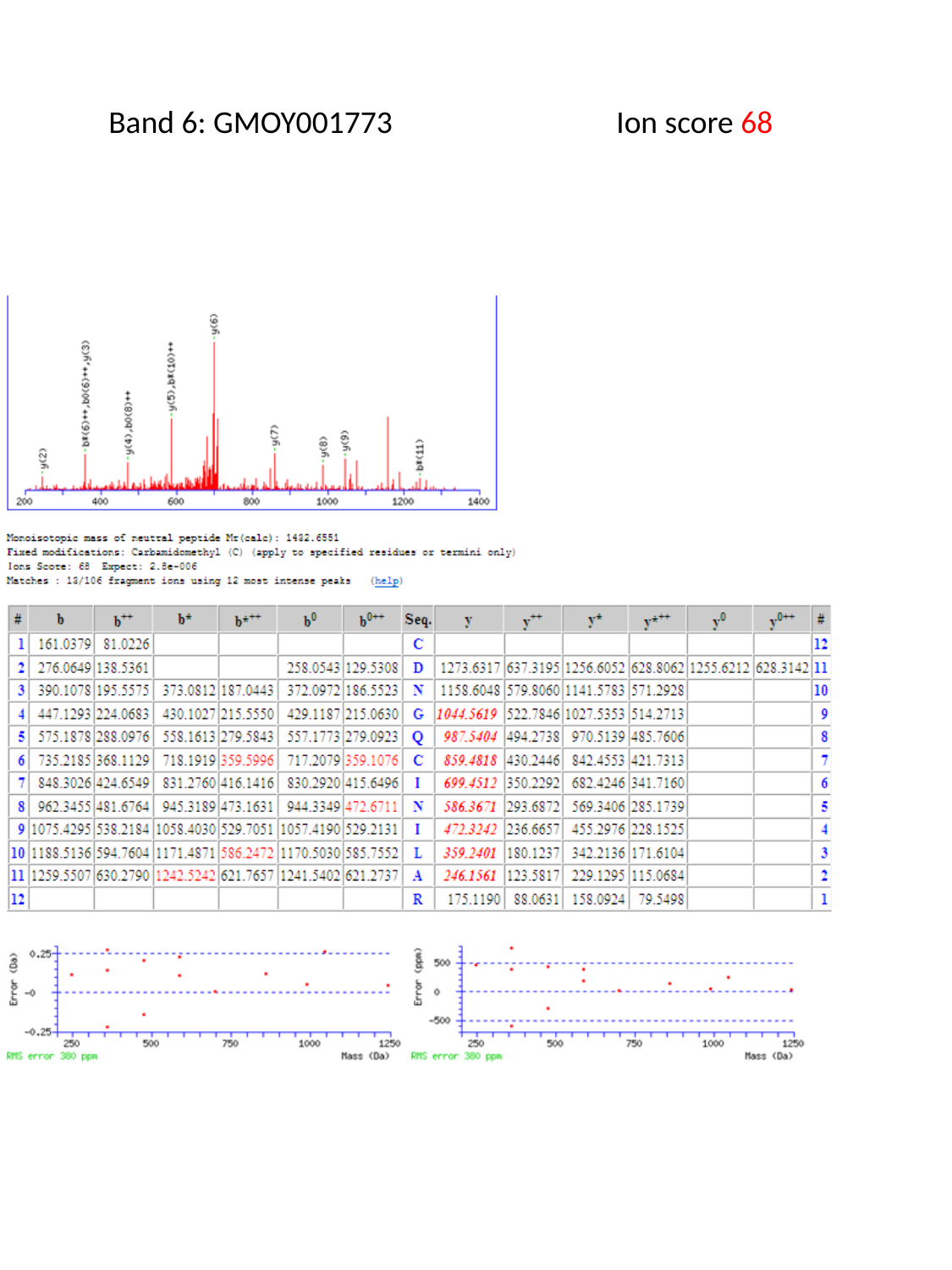

Band 6: GMOY001773 		Ion score 68

## Slide 6
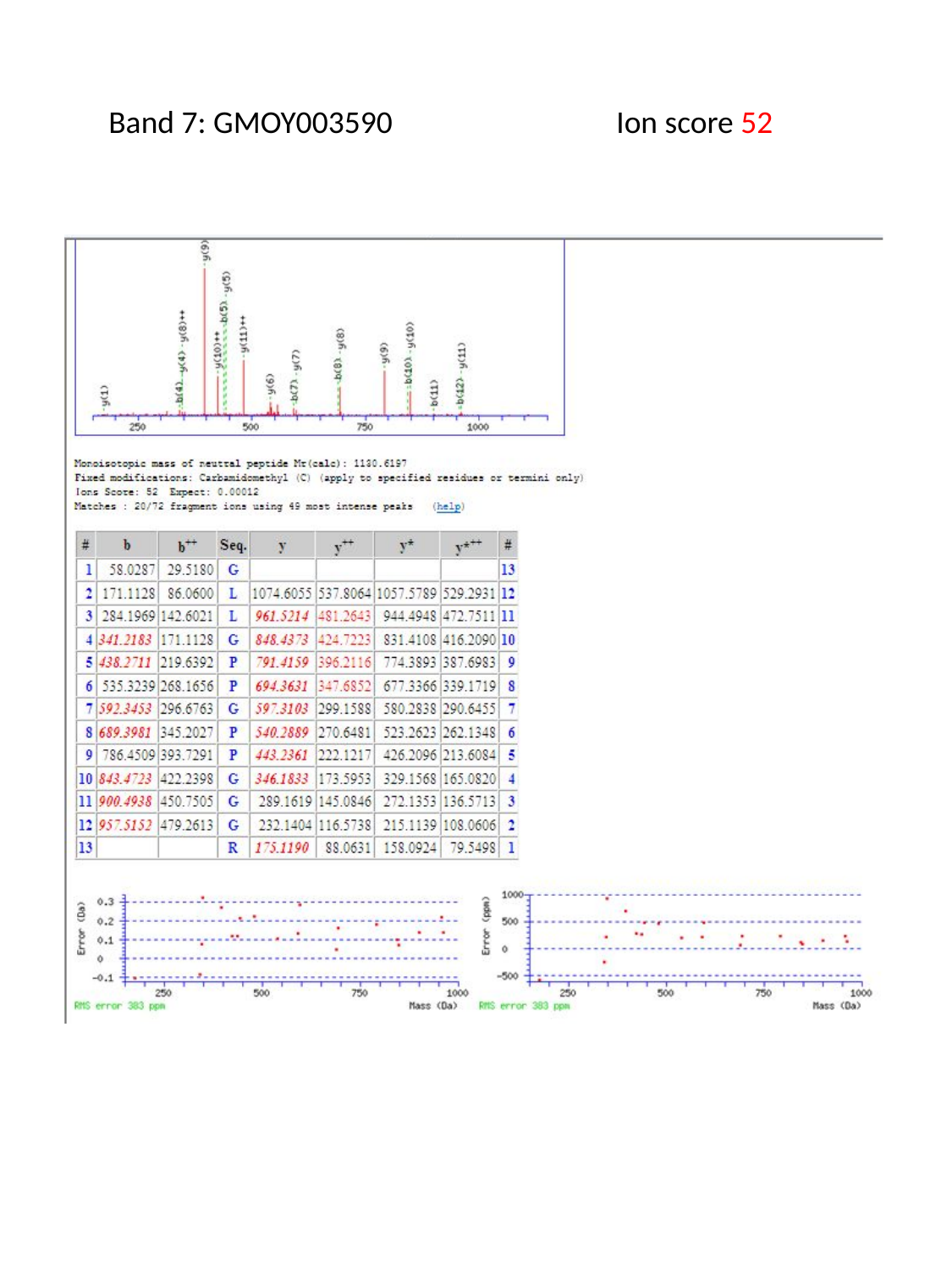

Band 7: GMOY003590 		Ion score 52

## Slide 7
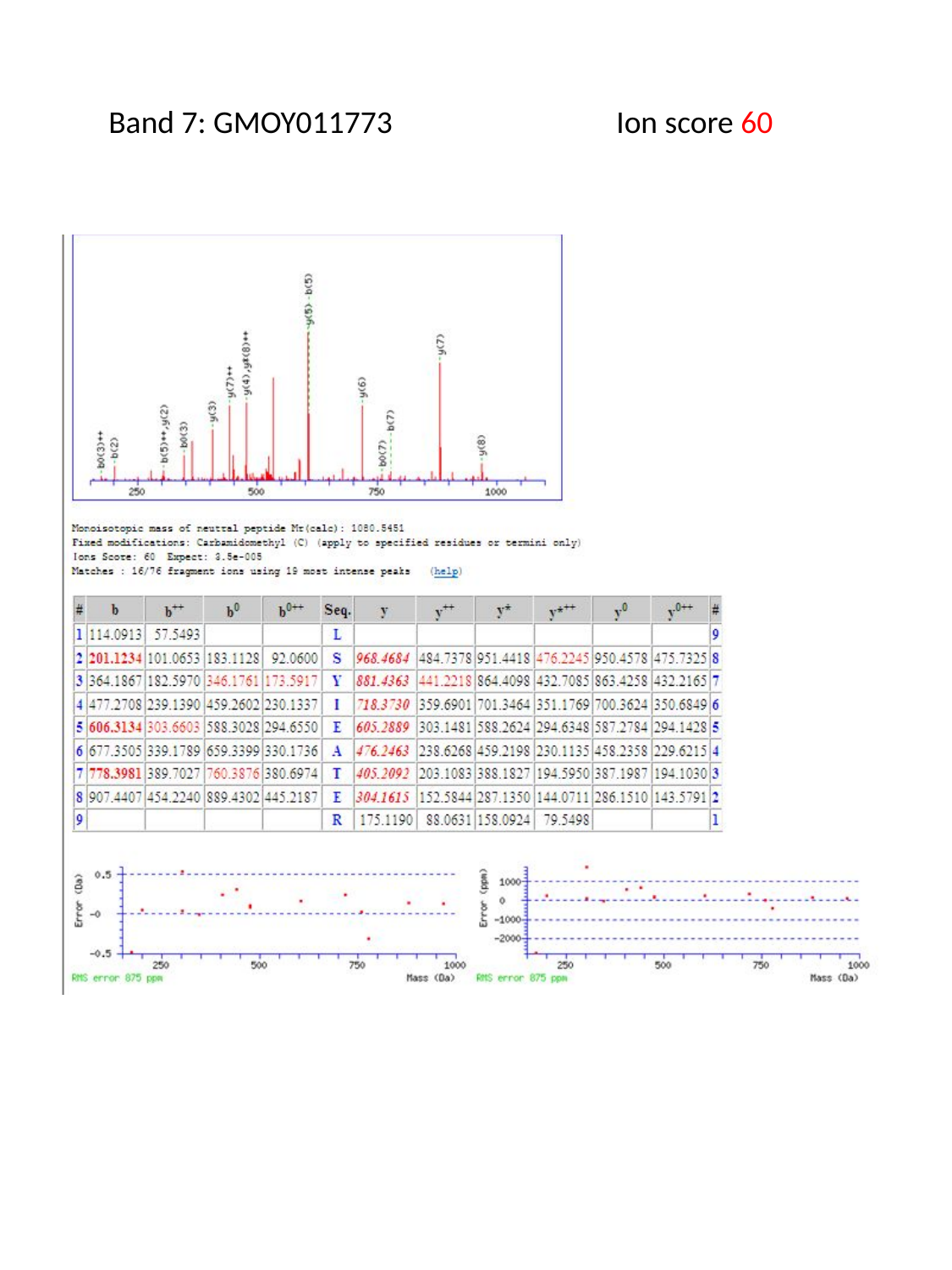

Band 7: GMOY011773 		Ion score 60

## Slide 8
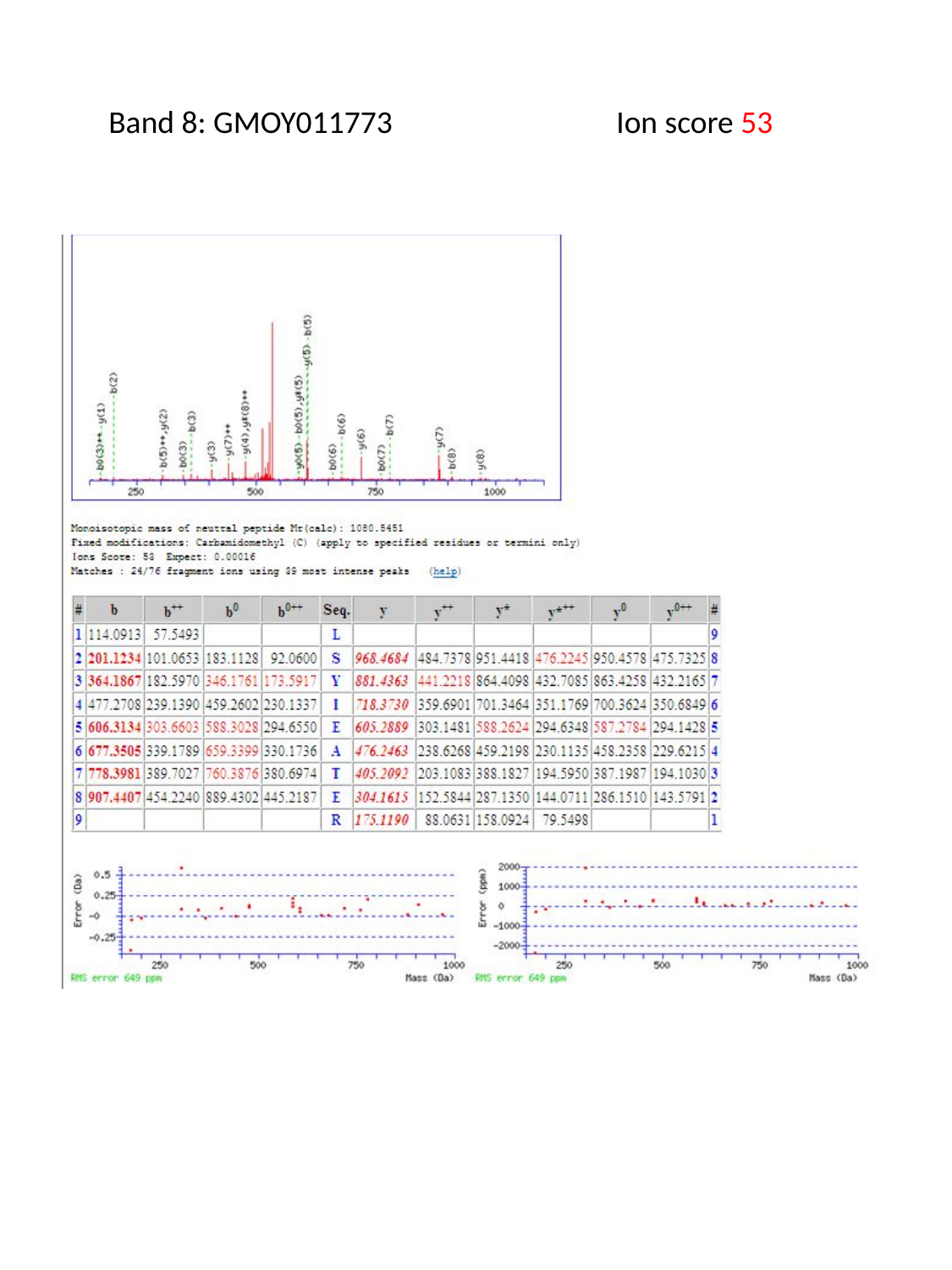

Band 8: GMOY011773 		Ion score 53

## Slide 9
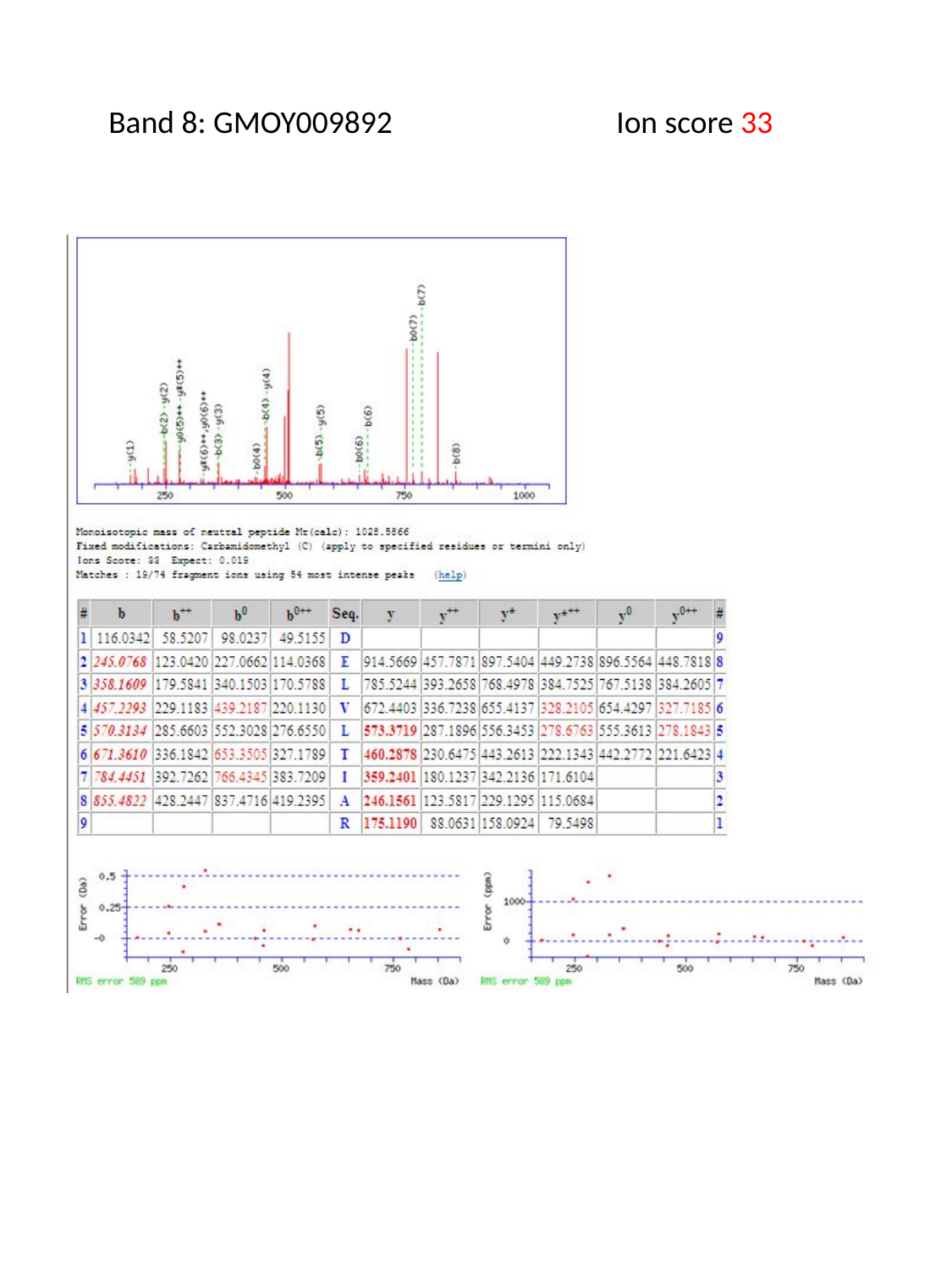

Band 8: GMOY009892 		Ion score 33

## Slide 10
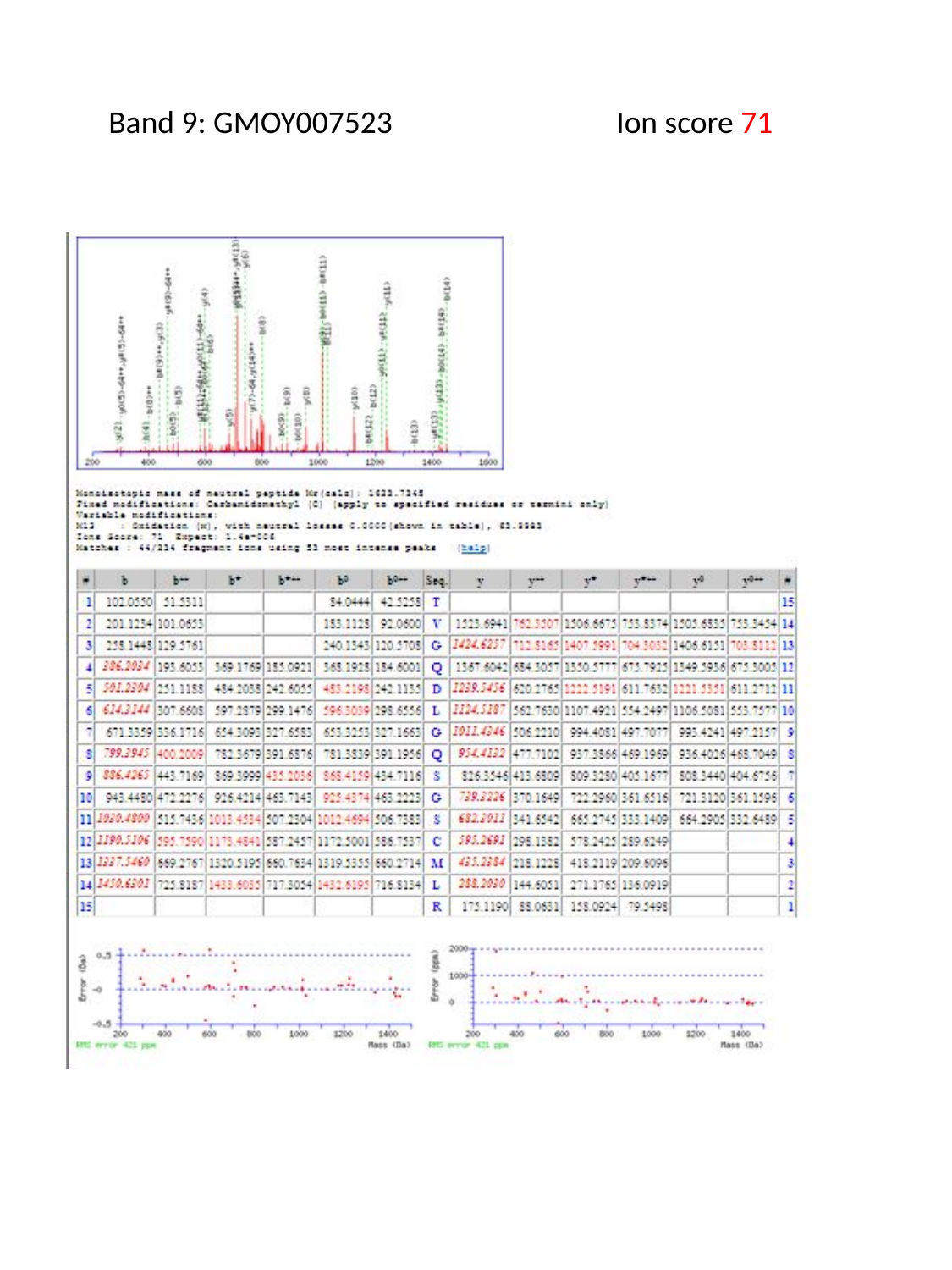

Band 9: GMOY007523 		Ion score 71

## Slide 11
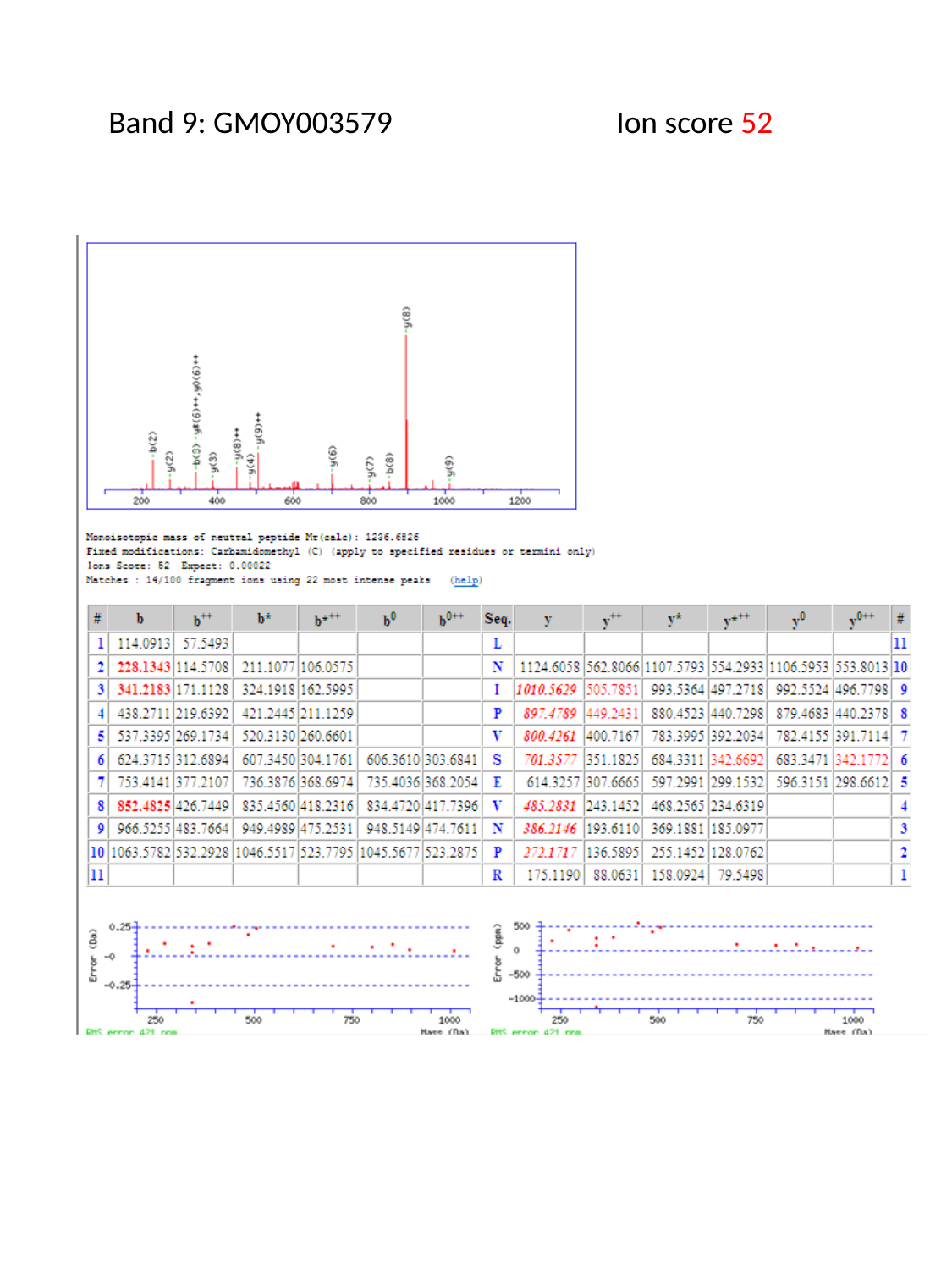

Band 9: GMOY003579 		Ion score 52

## Slide 12
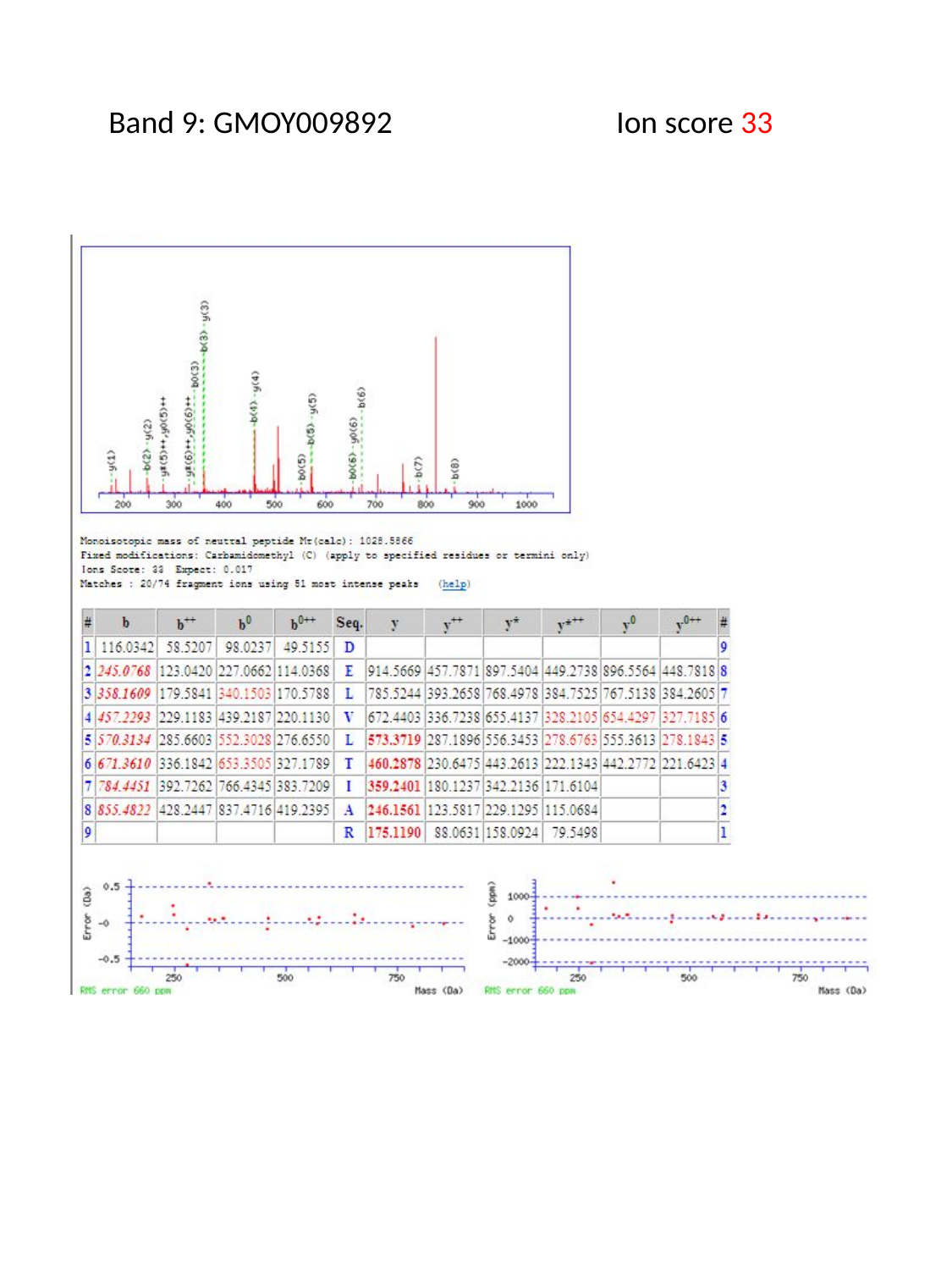

Band 9: GMOY009892 		Ion score 33

## Slide 13
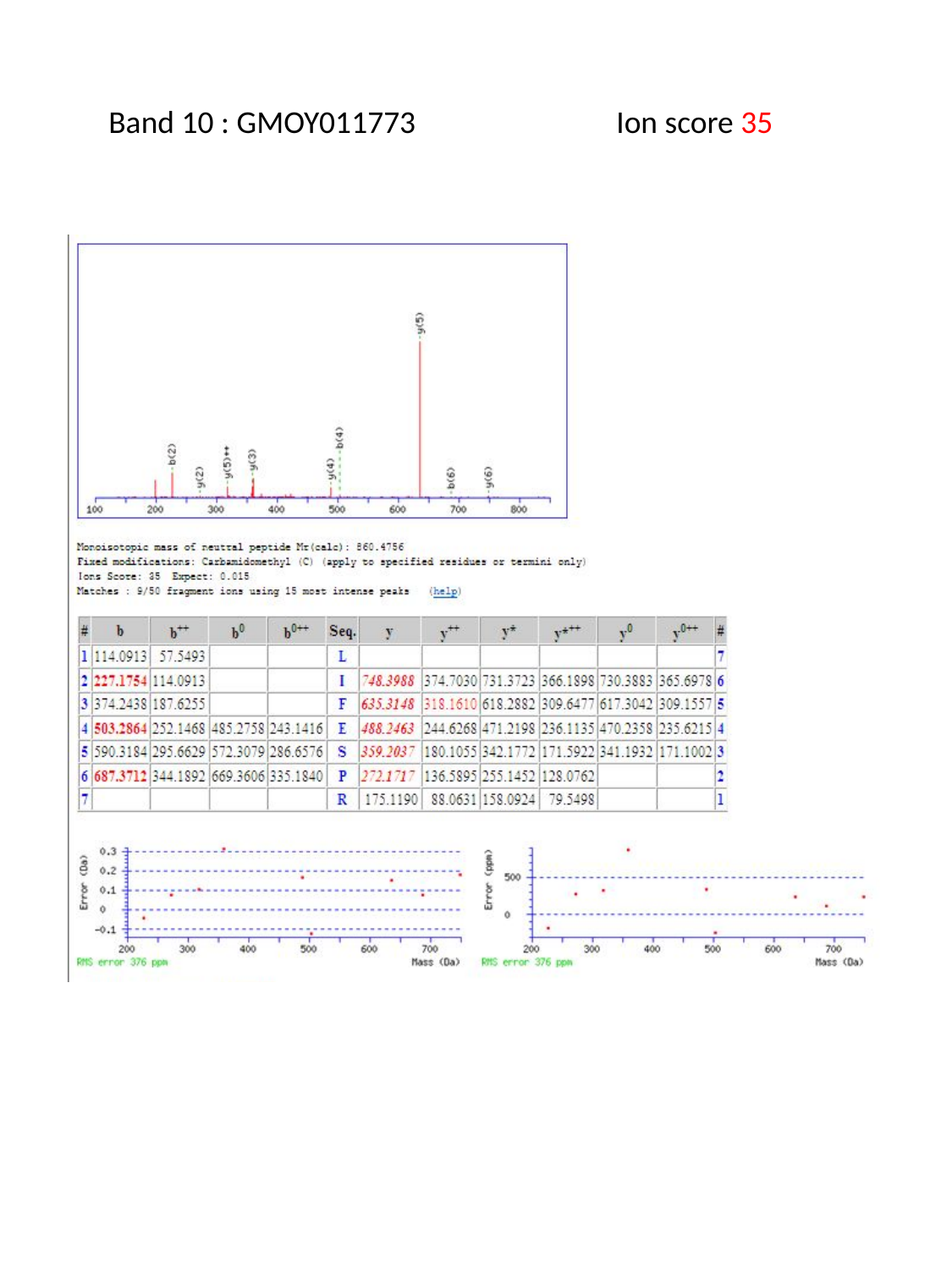

Band 10 : GMOY011773 		Ion score 35

## Slide 14
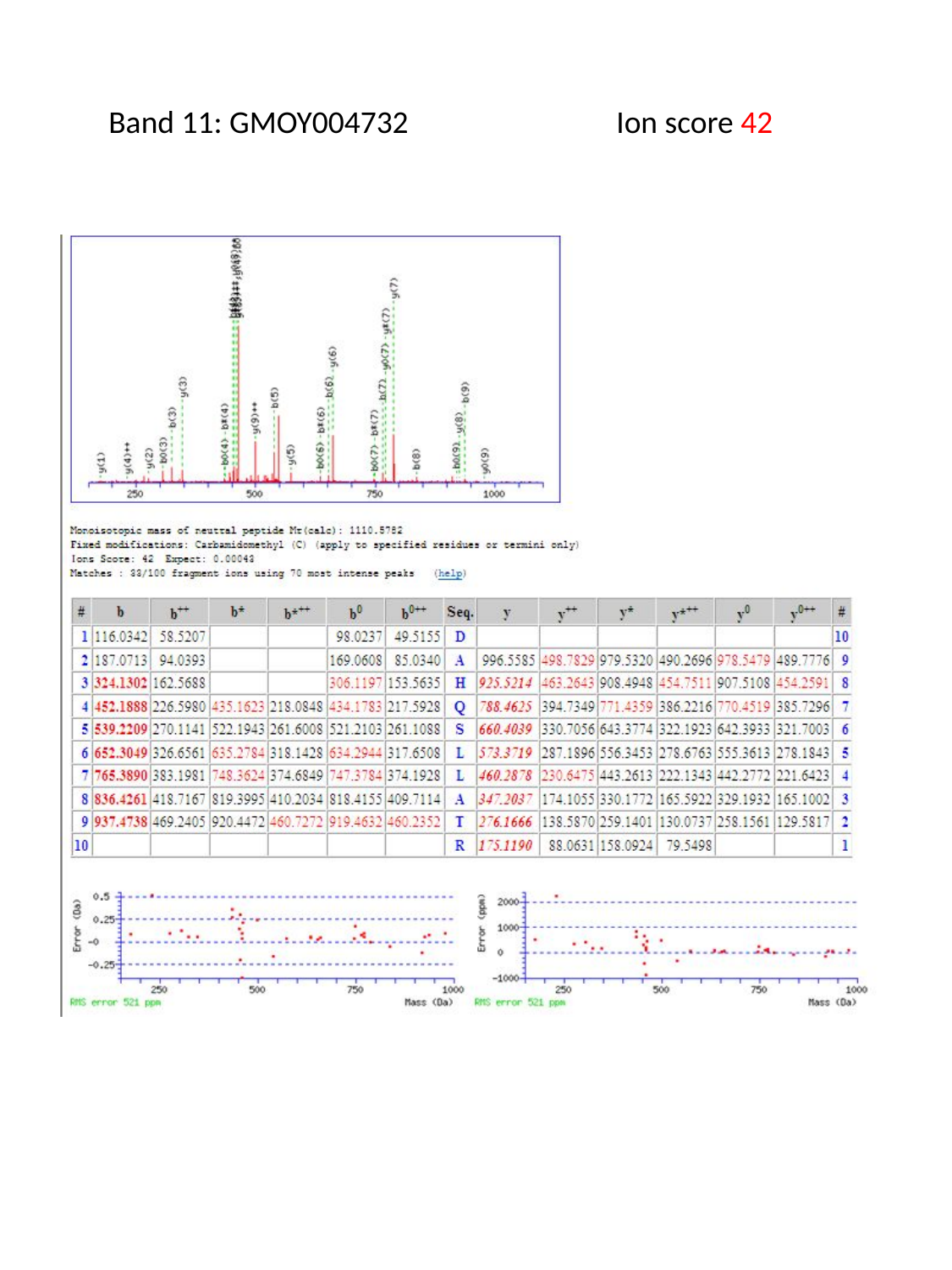

Band 11: GMOY004732 		Ion score 42

## Slide 15
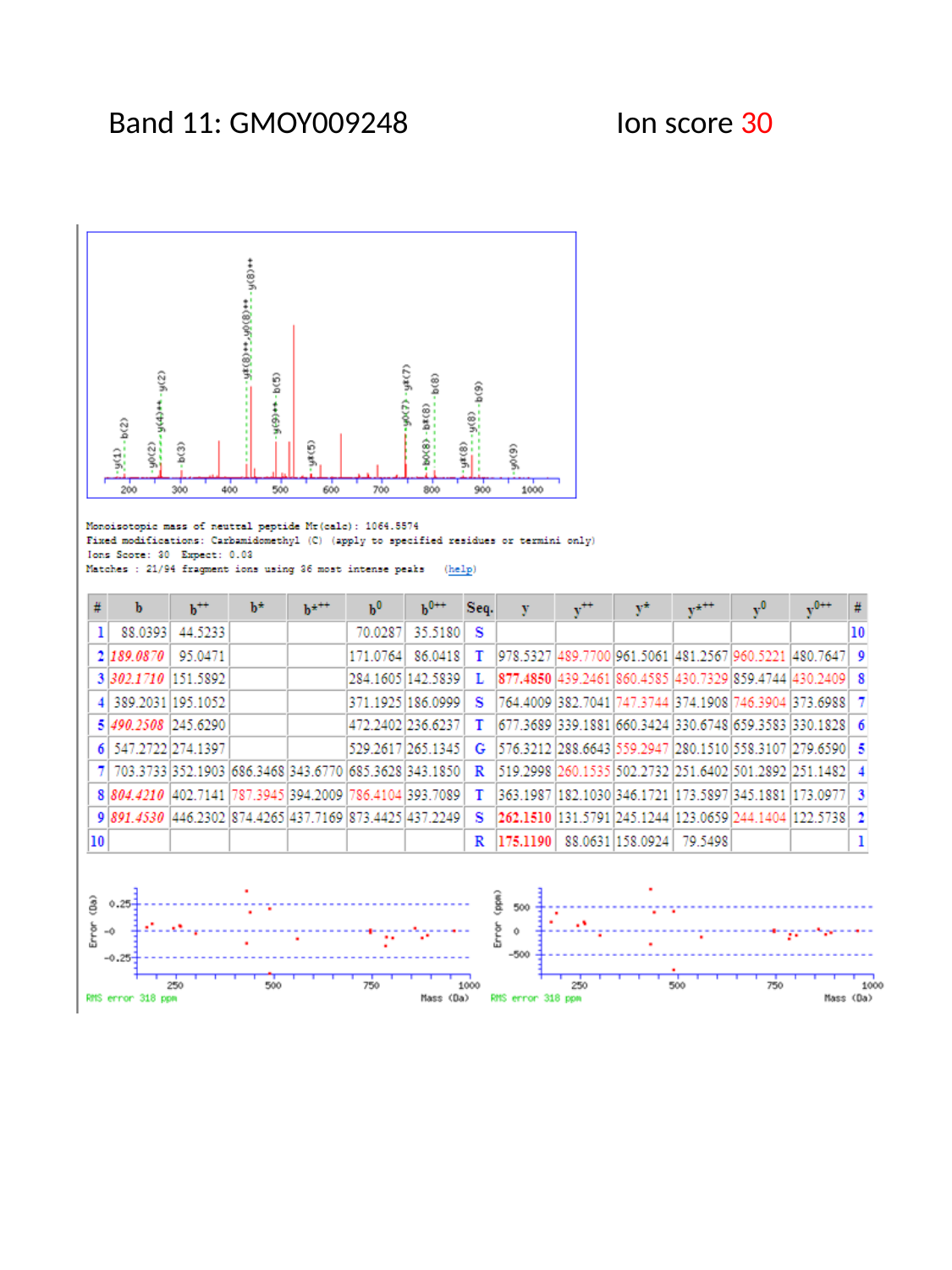

Band 11: GMOY009248 		Ion score 30

## Slide 16
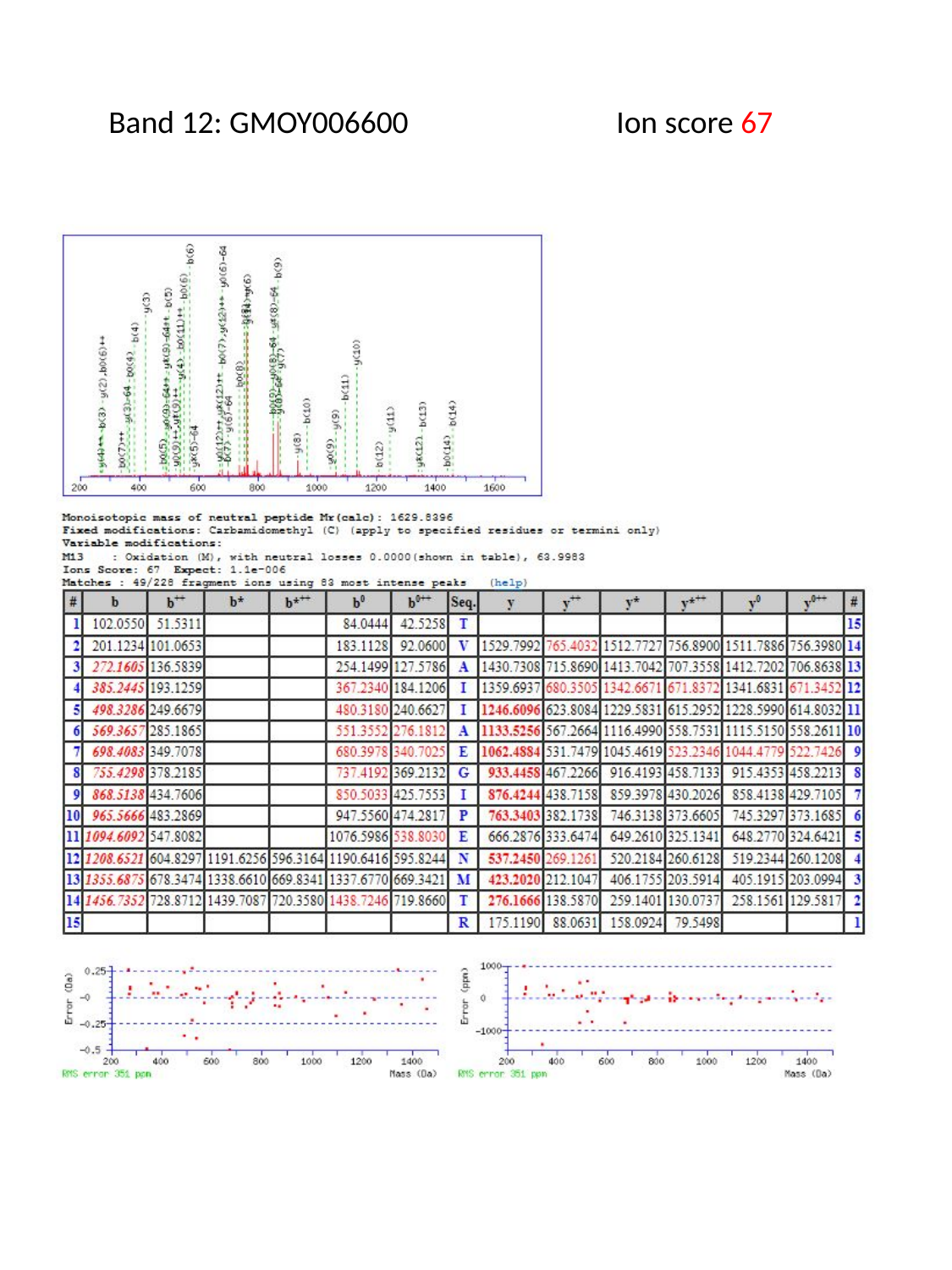

Band 12: GMOY006600 		Ion score 67

## Slide 17
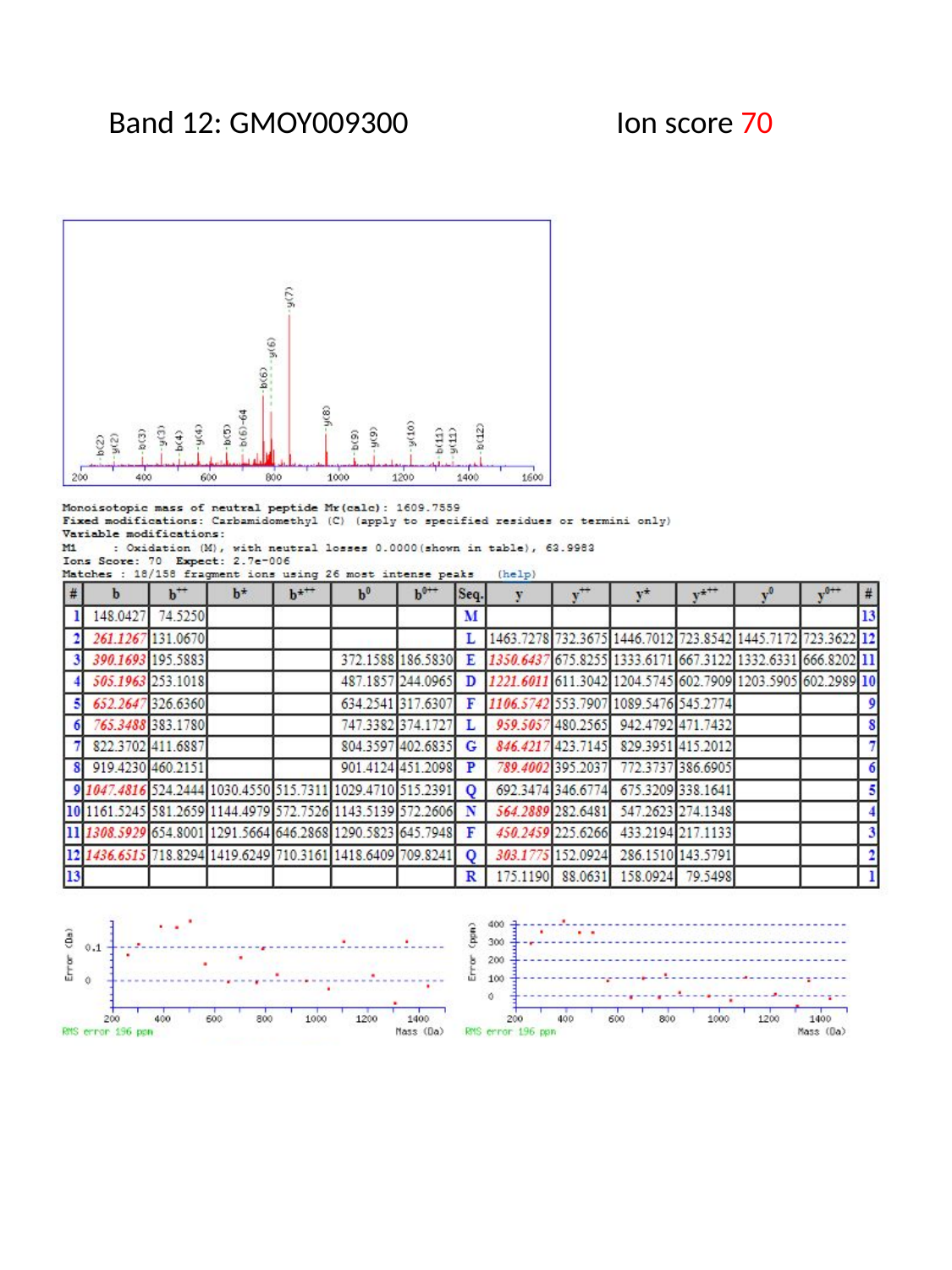

Band 12: GMOY009300 		Ion score 70

## Slide 18
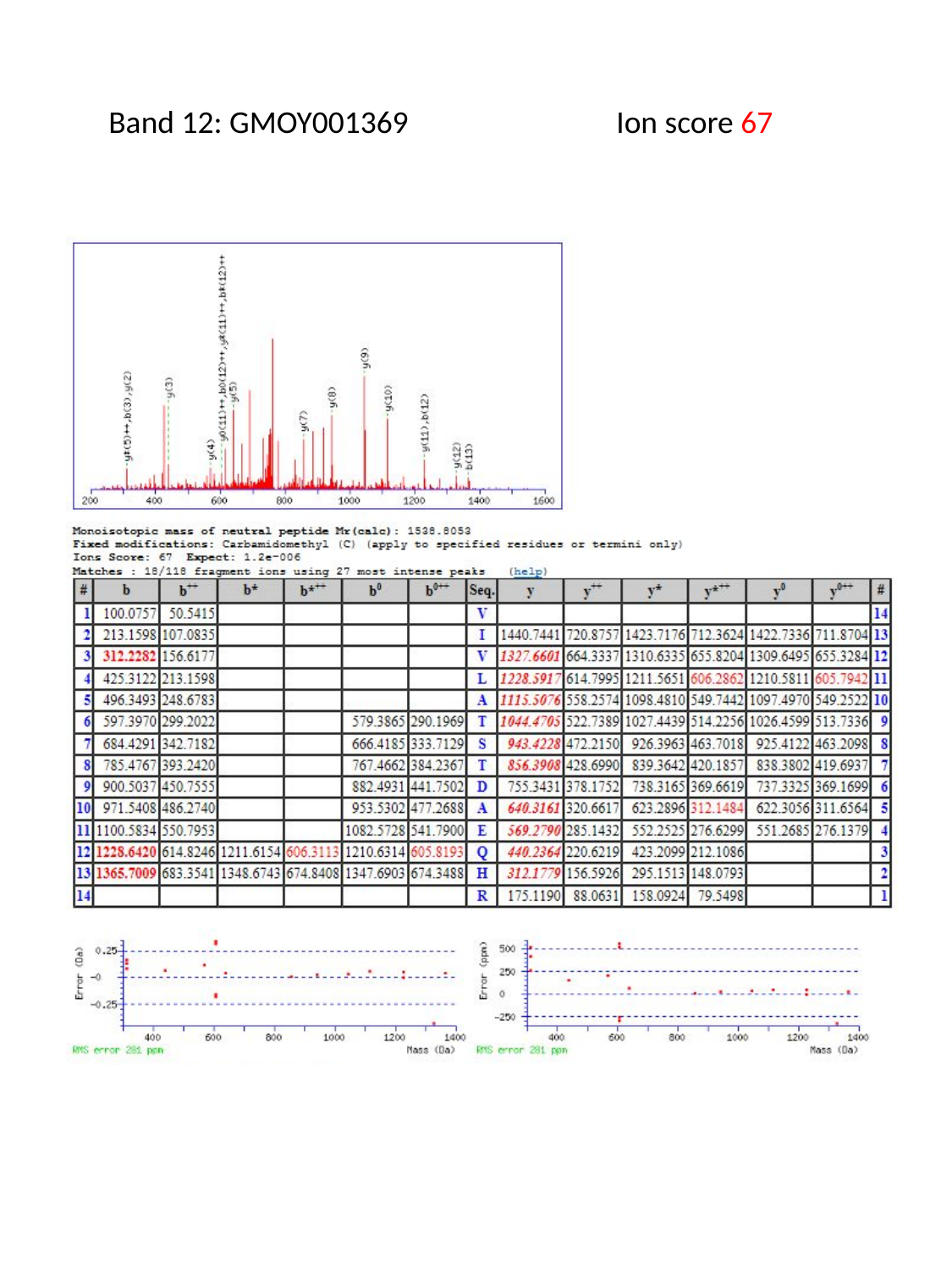

Band 12: GMOY001369 		Ion score 67

## Slide 19
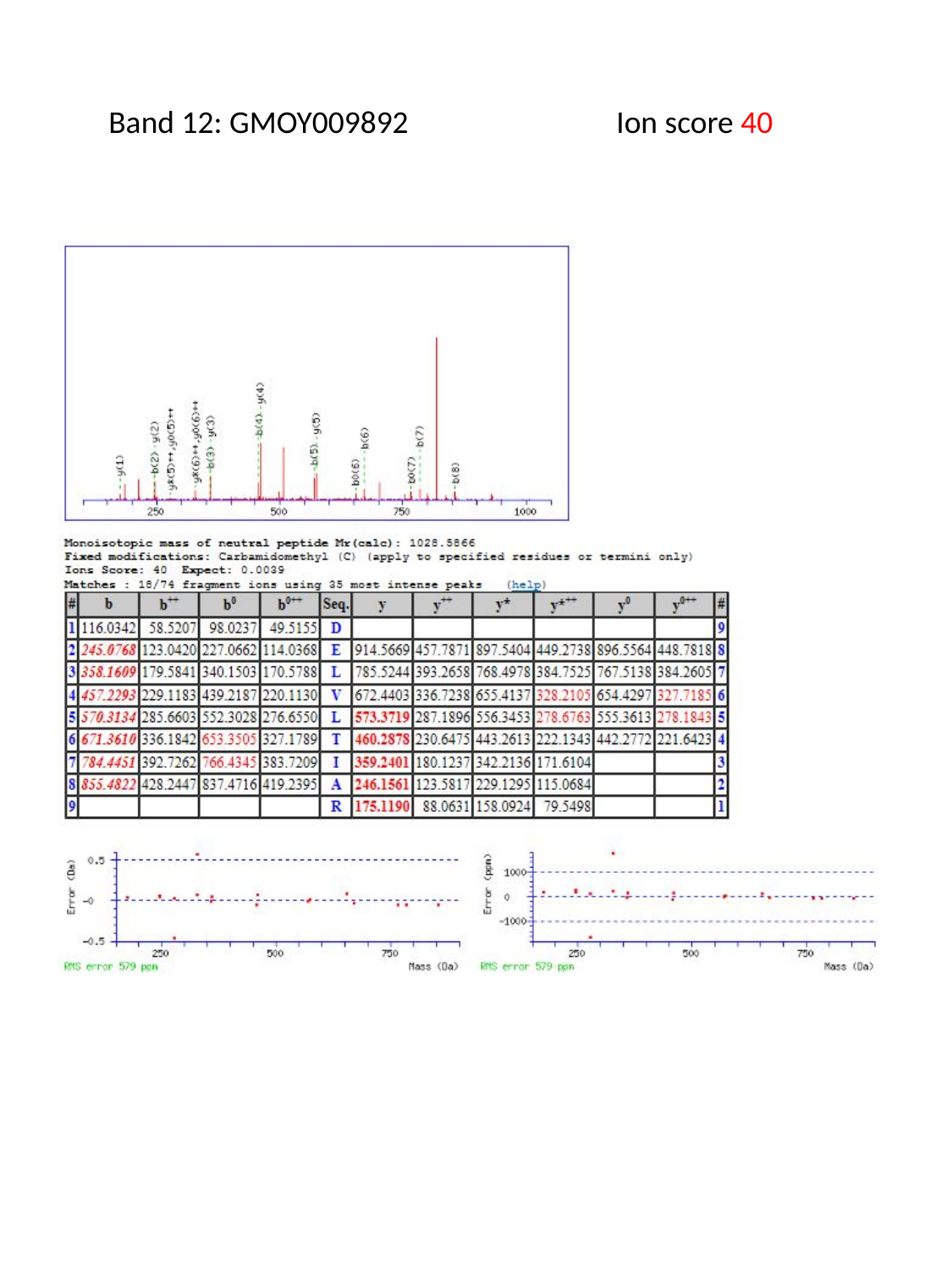

Band 12: GMOY009892 		Ion score 40

## Slide 20
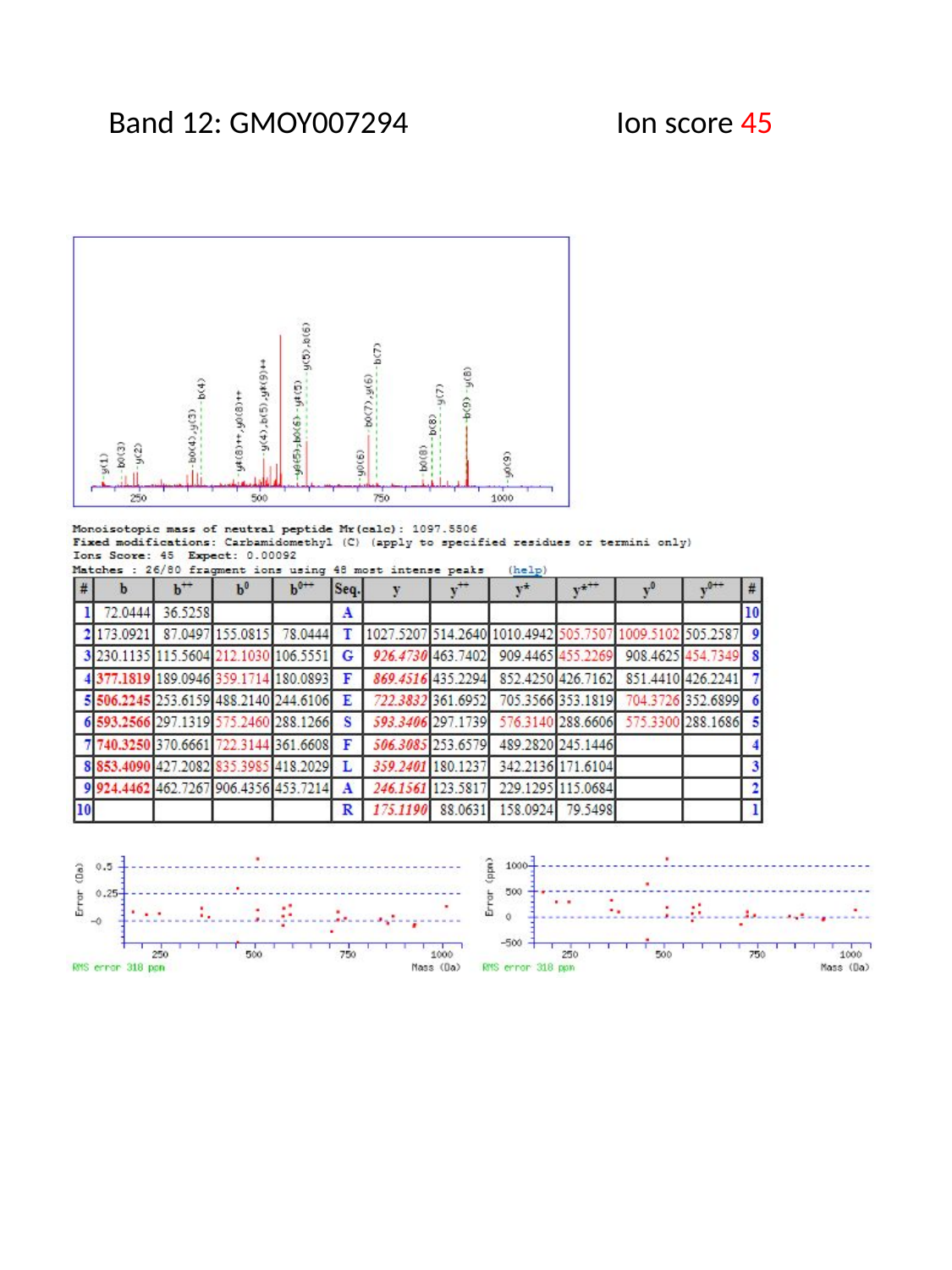

Band 12: GMOY007294 		Ion score 45

## Slide 21
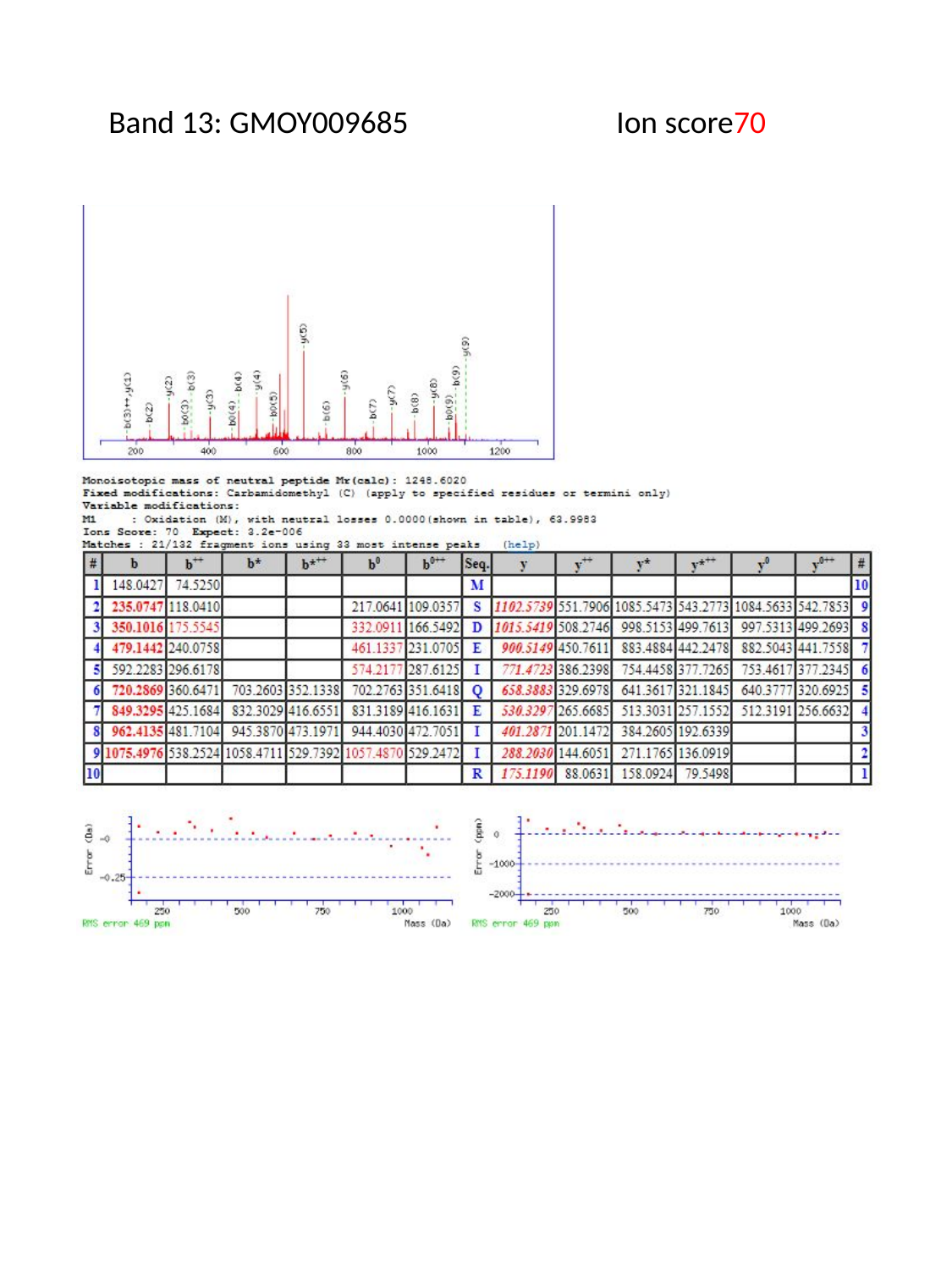

Band 13: GMOY009685 		Ion score70

## Slide 22
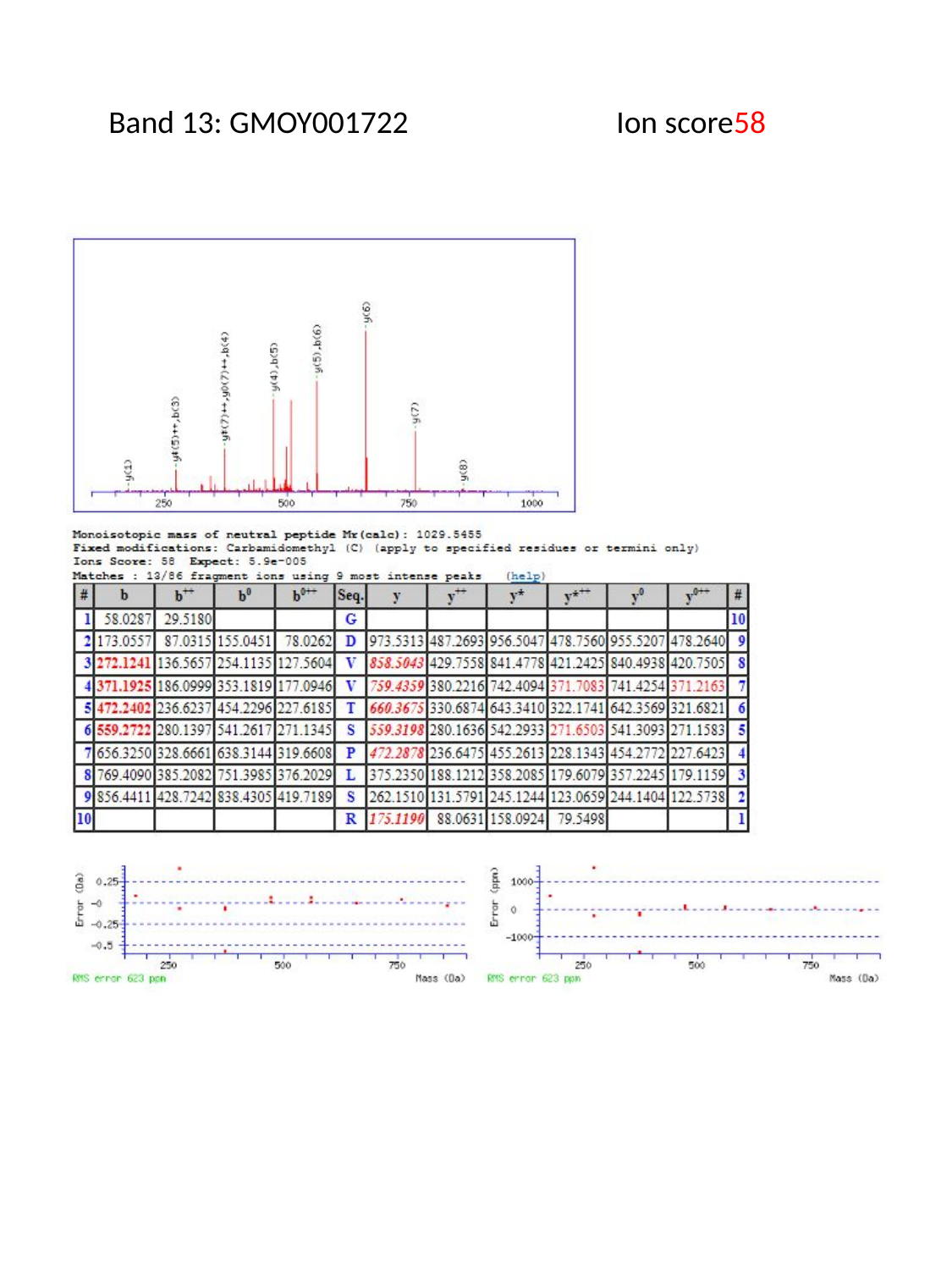

Band 13: GMOY001722 		Ion score58

## Slide 23
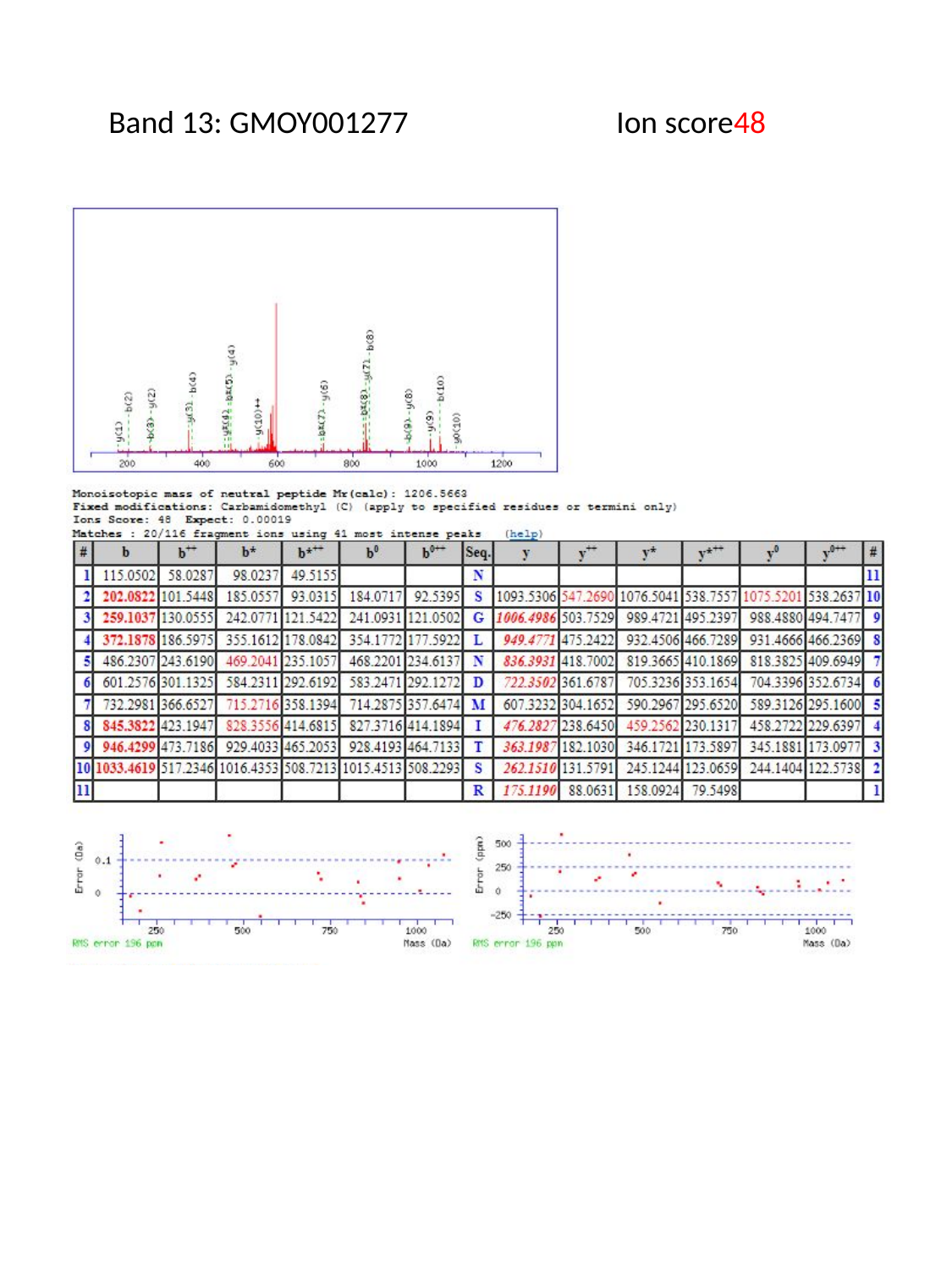

Band 13: GMOY001277 		Ion score48

## Slide 24
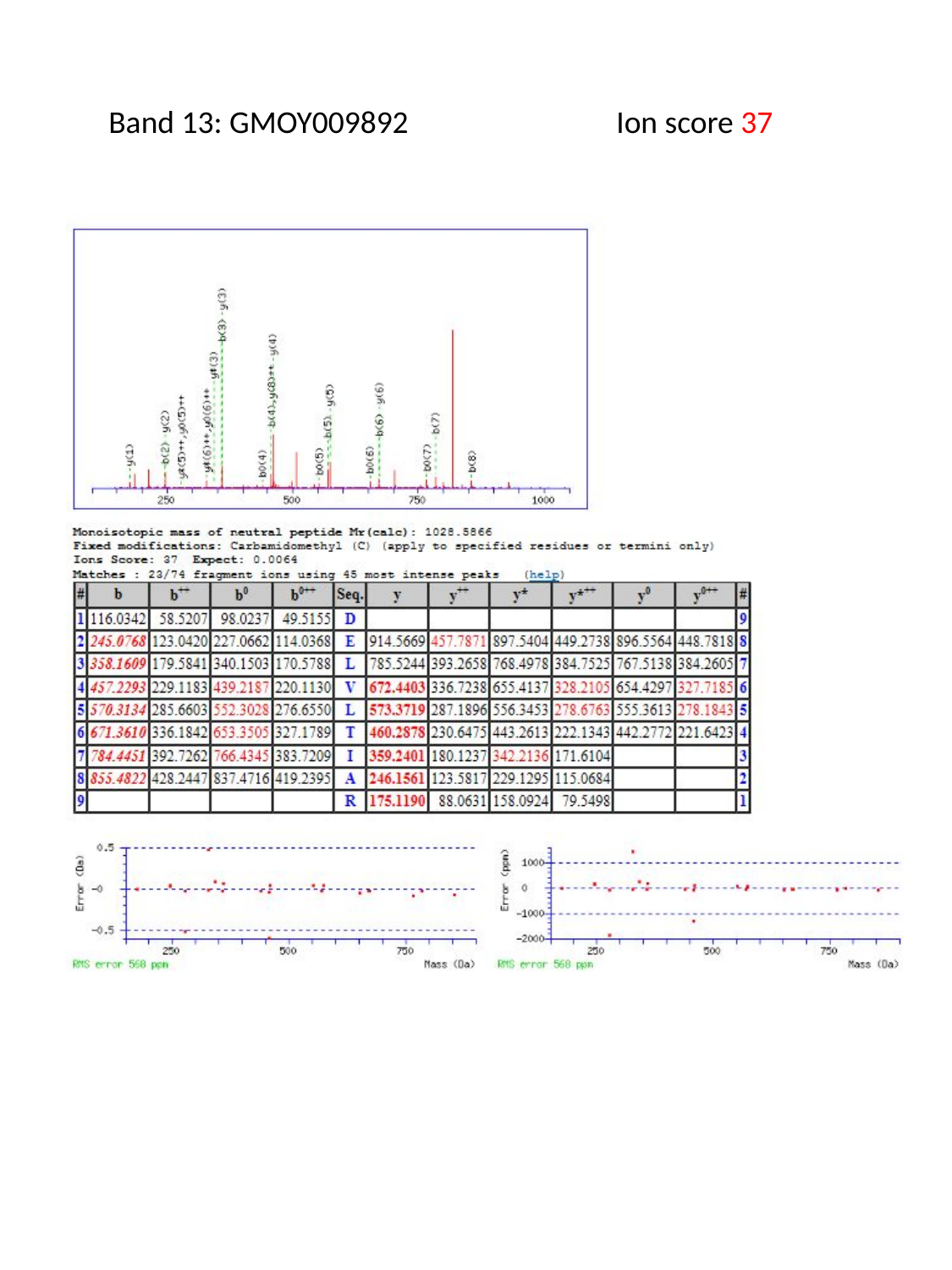

Band 13: GMOY009892 		Ion score 37

## Slide 25
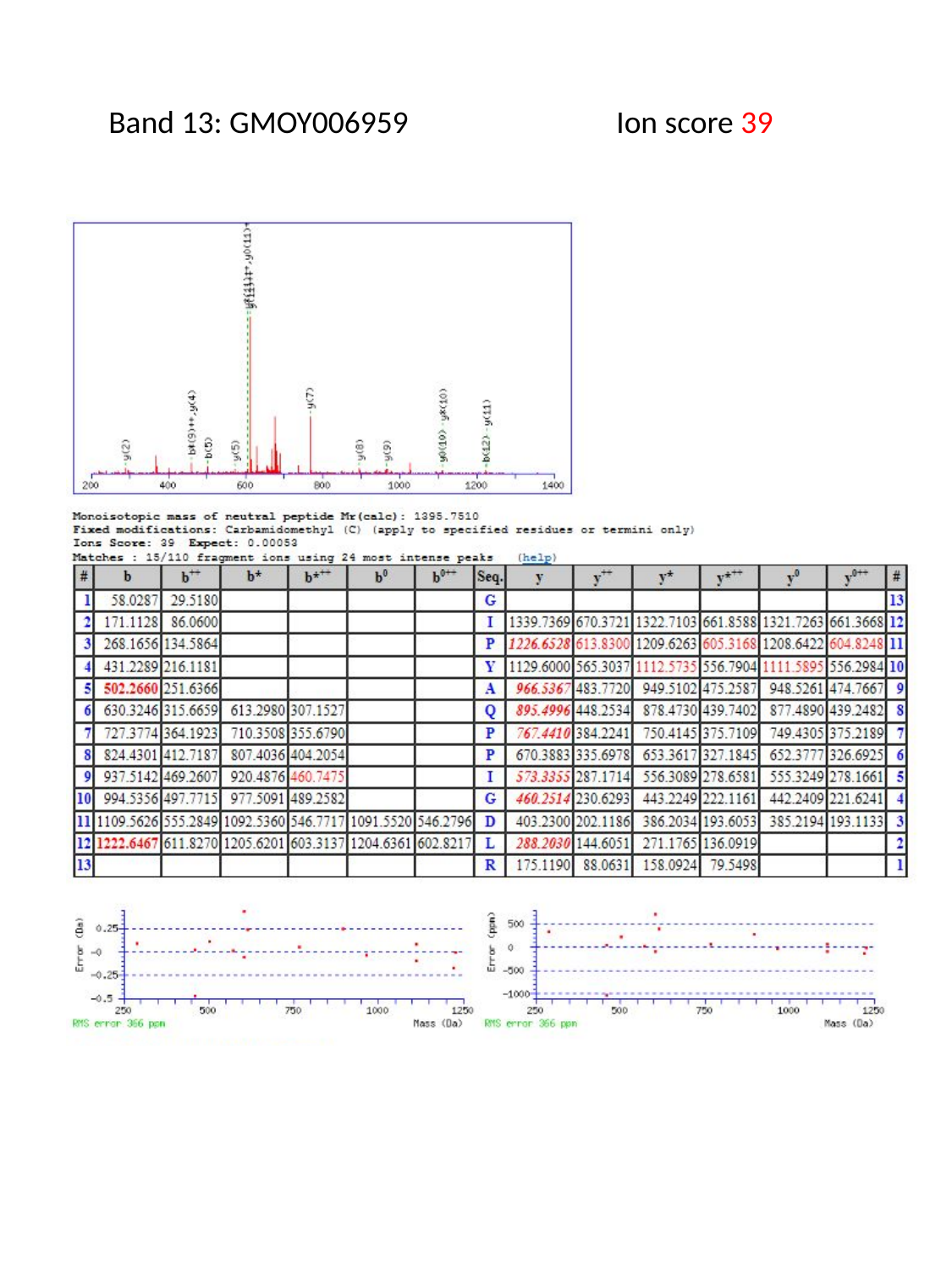

Band 13: GMOY006959 		Ion score 39

## Slide 26
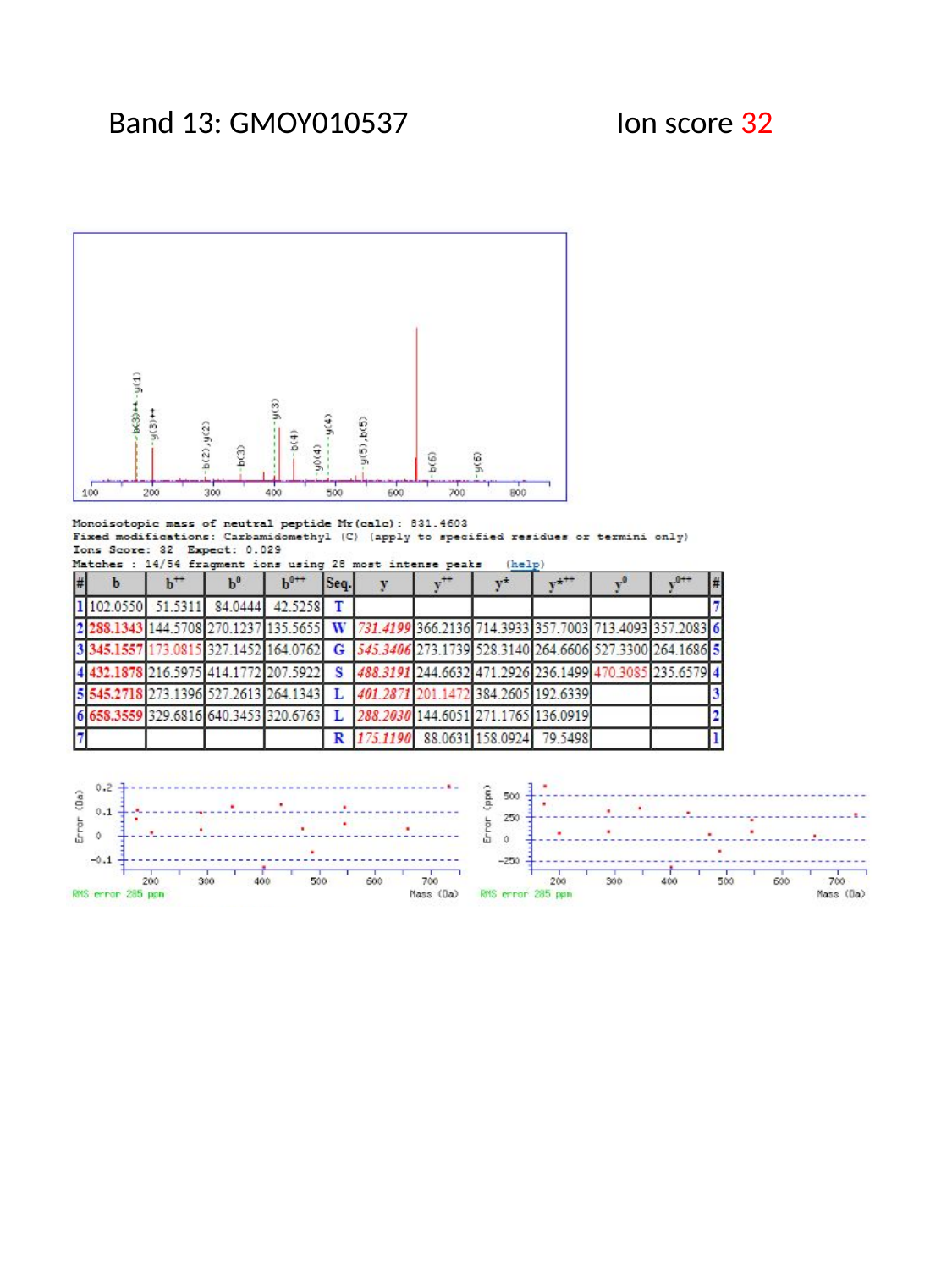

Band 13: GMOY010537 		Ion score 32

## Slide 27
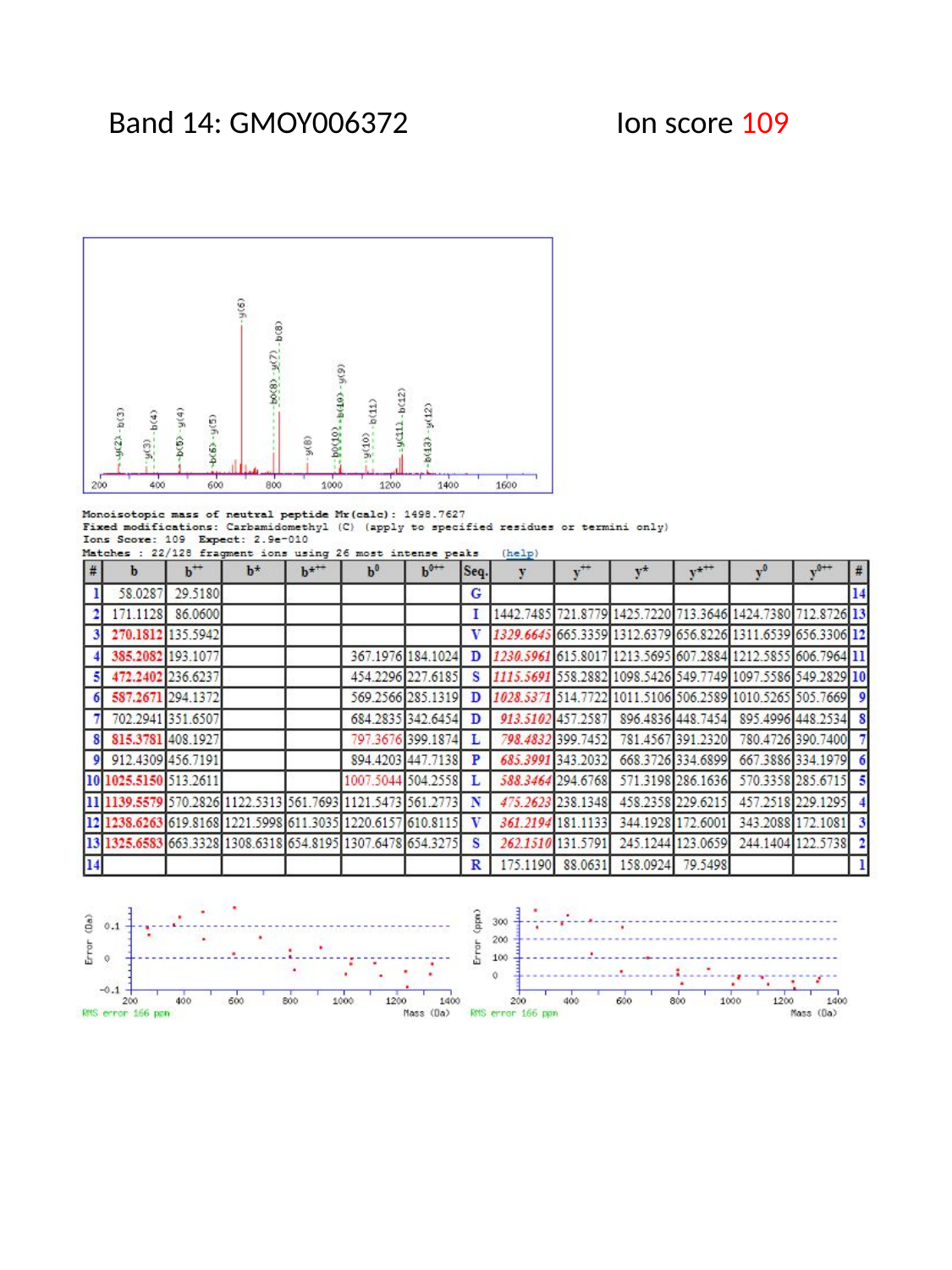

Band 14: GMOY006372 		Ion score 109

## Slide 28
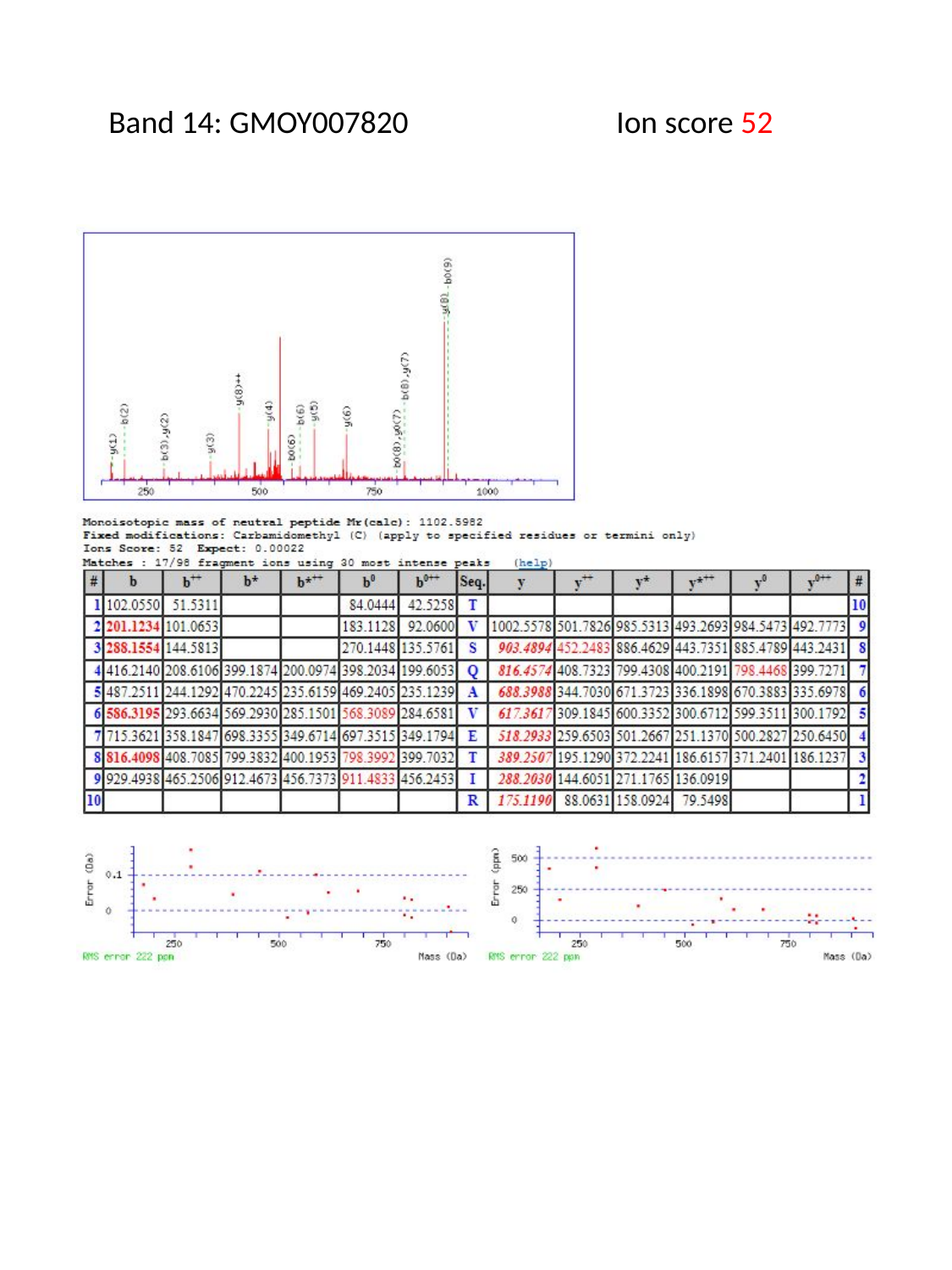

Band 14: GMOY007820 		Ion score 52

## Slide 29
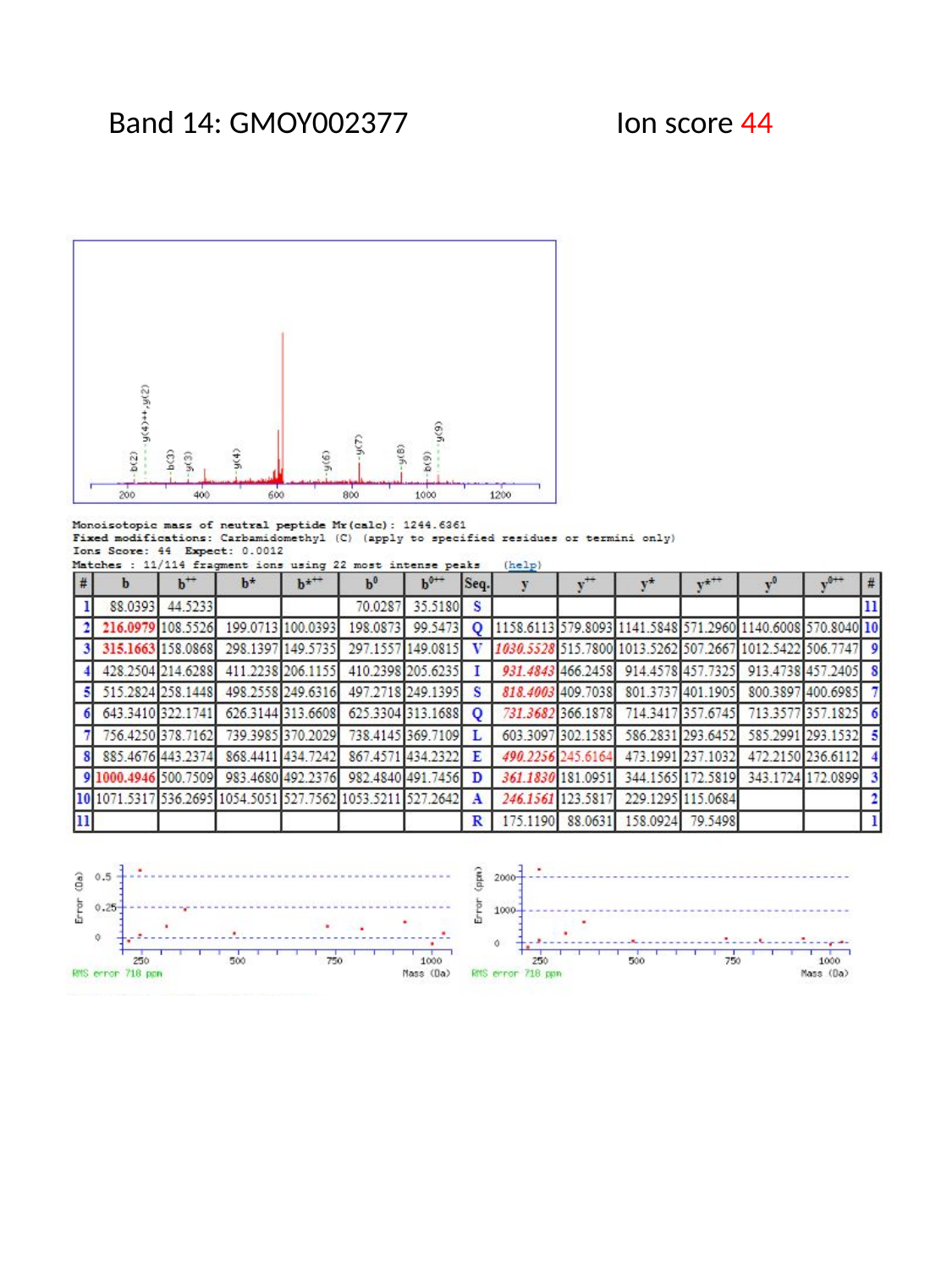

Band 14: GMOY002377 		Ion score 44

## Slide 30
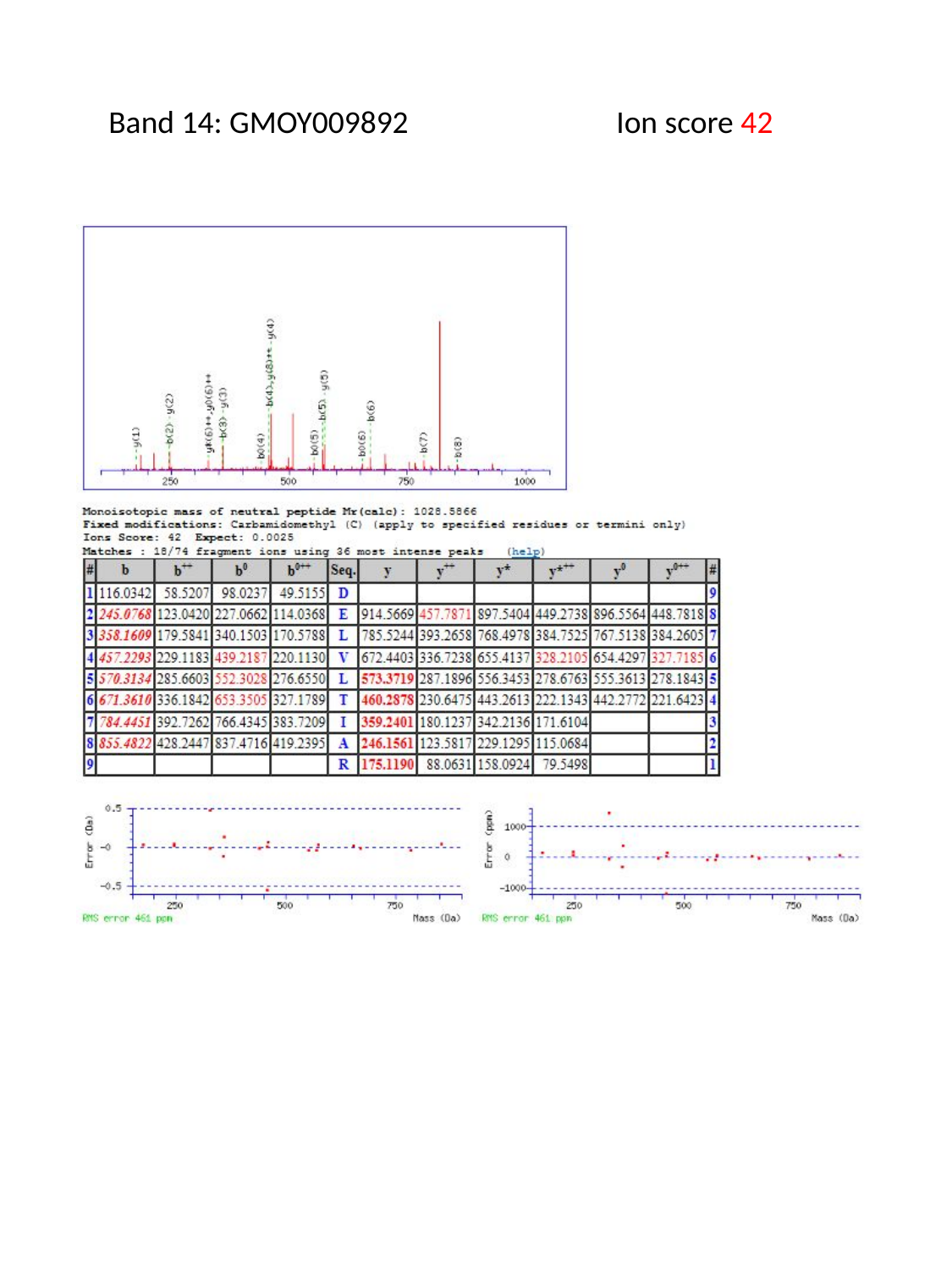

Band 14: GMOY009892 		Ion score 42

## Slide 31
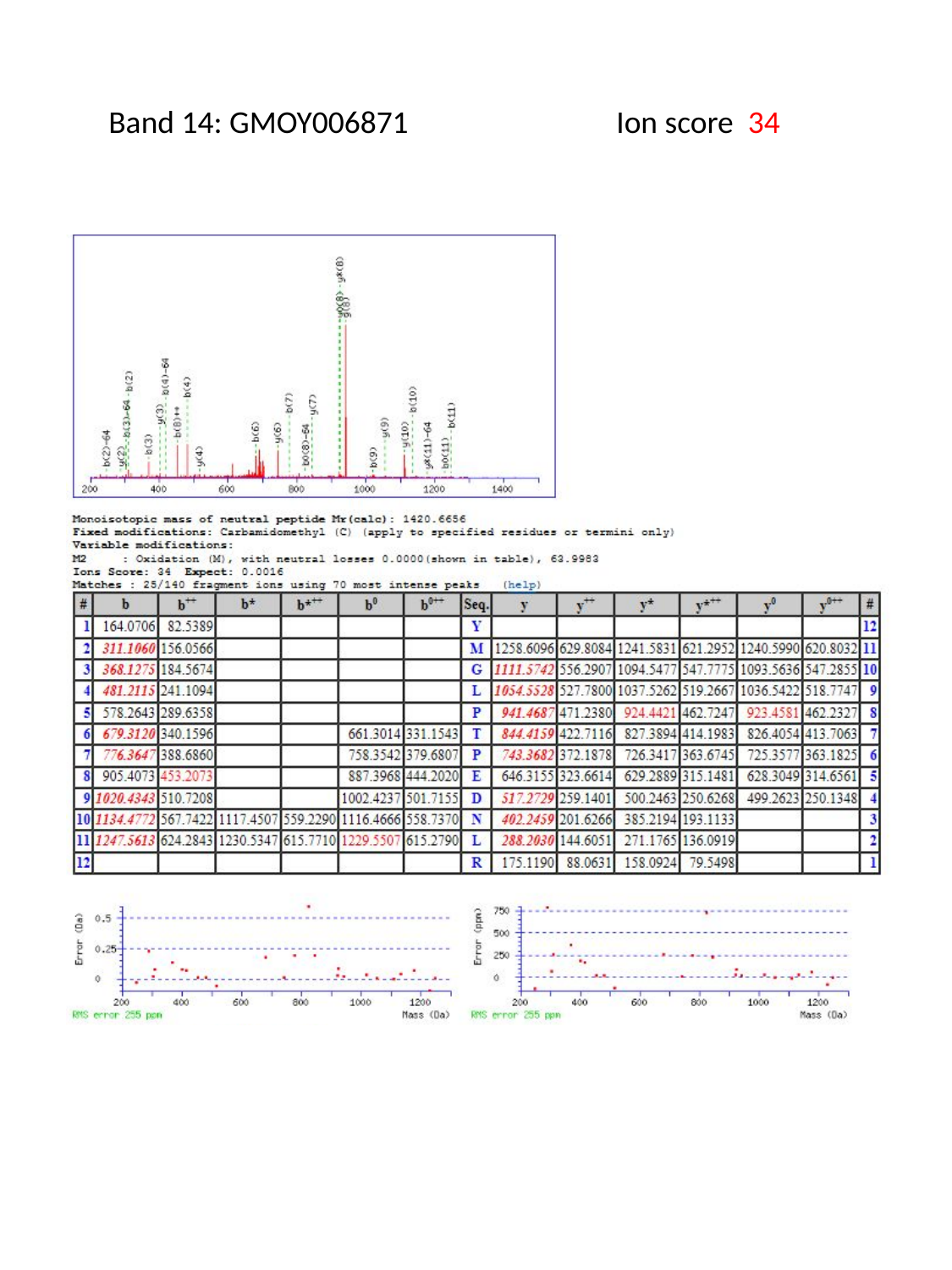

Band 14: GMOY006871 		Ion score 34

## Slide 32
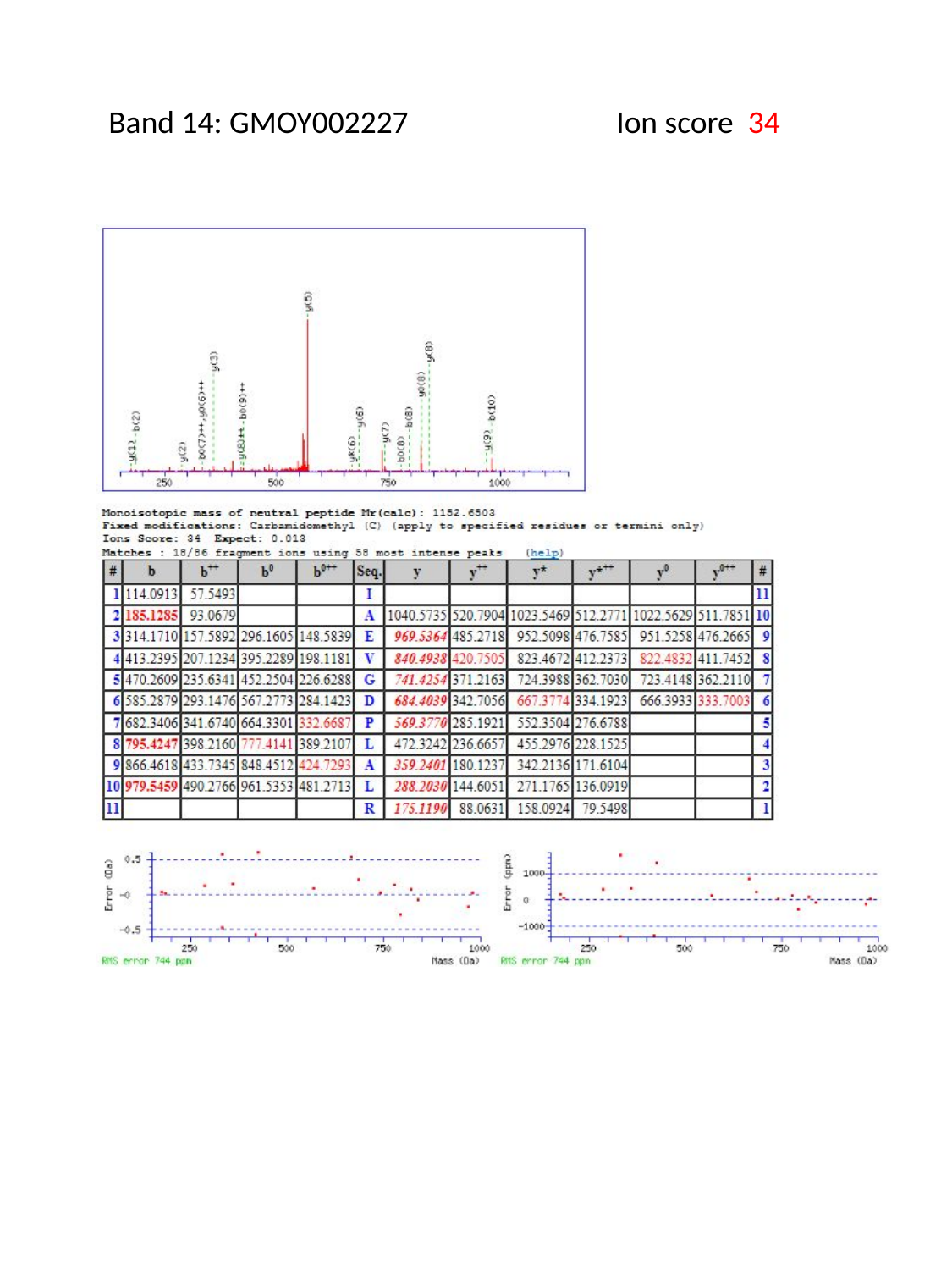

Band 14: GMOY002227 		Ion score 34

## Slide 33
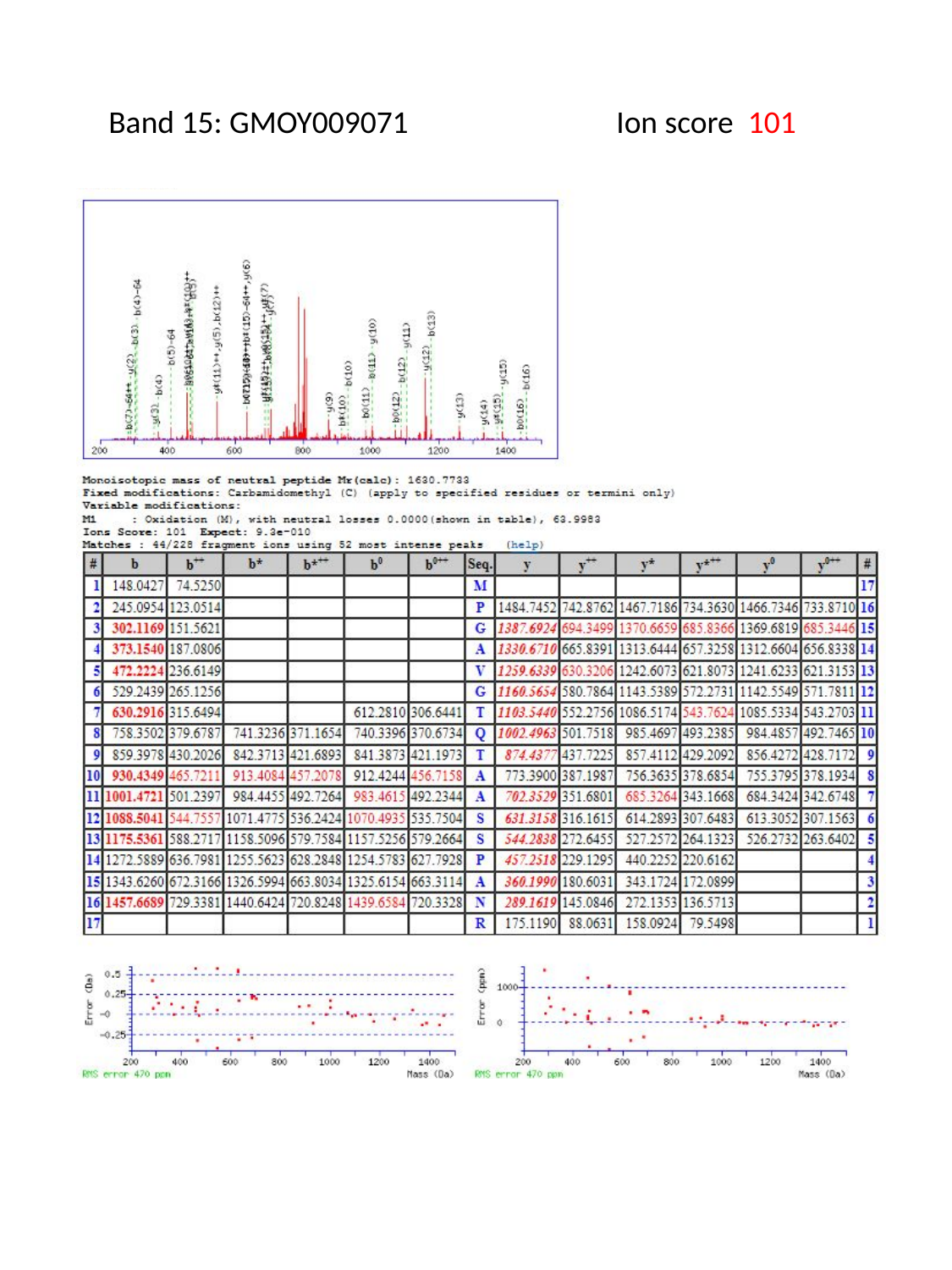

Band 15: GMOY009071 		Ion score 101

## Slide 34
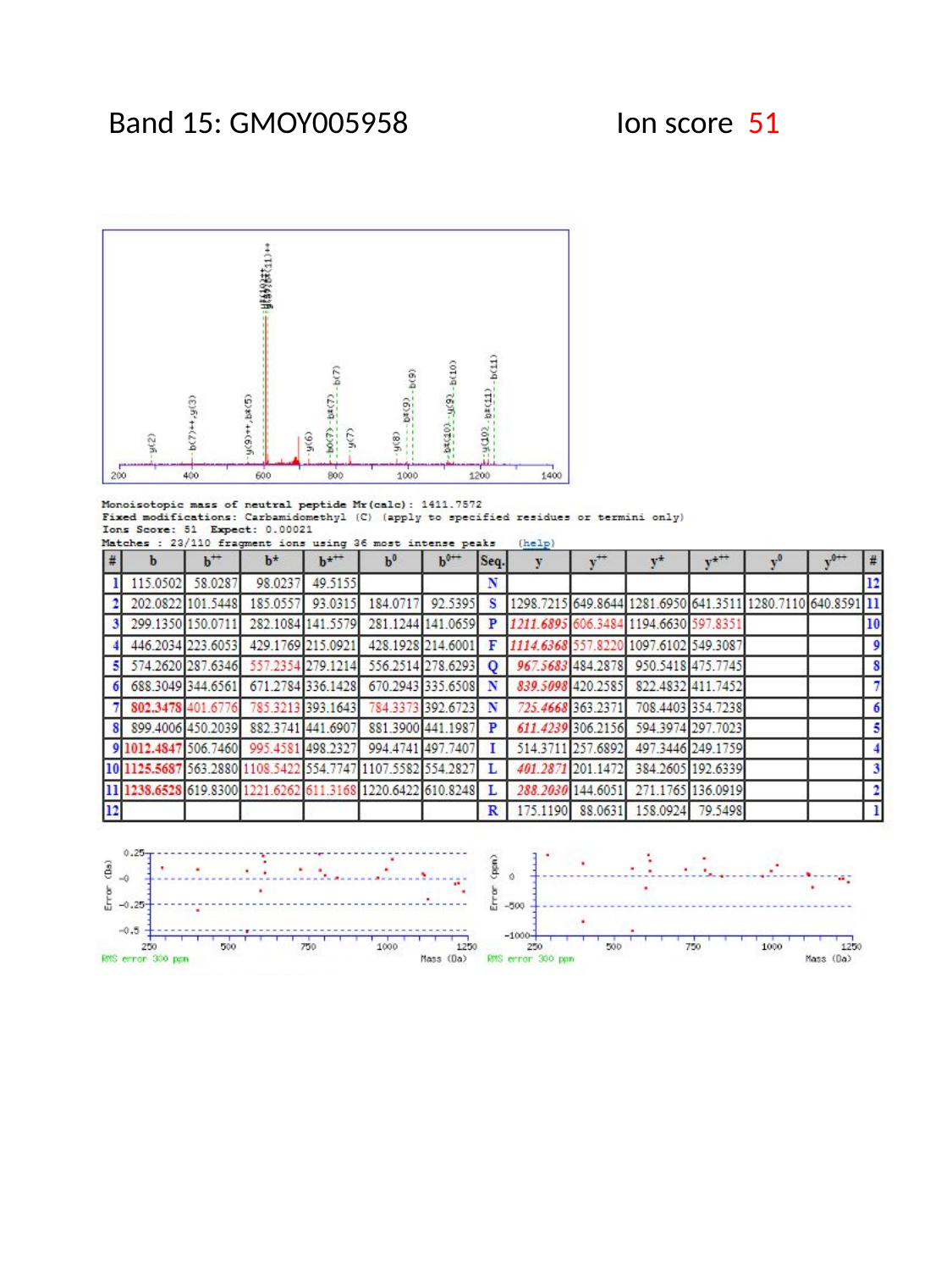

Band 15: GMOY005958 		Ion score 51

## Slide 35
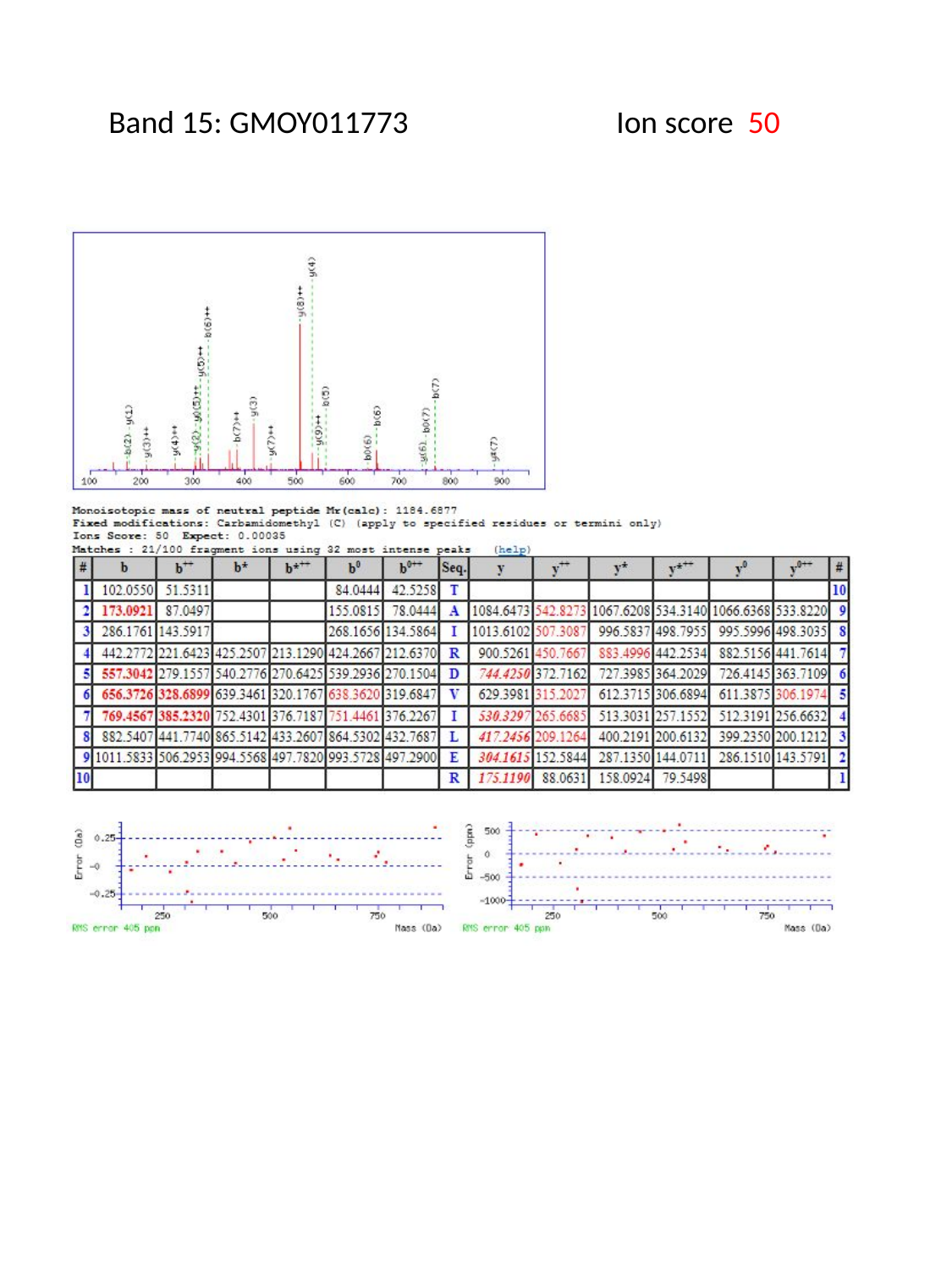

Band 15: GMOY011773 		Ion score 50

## Slide 36
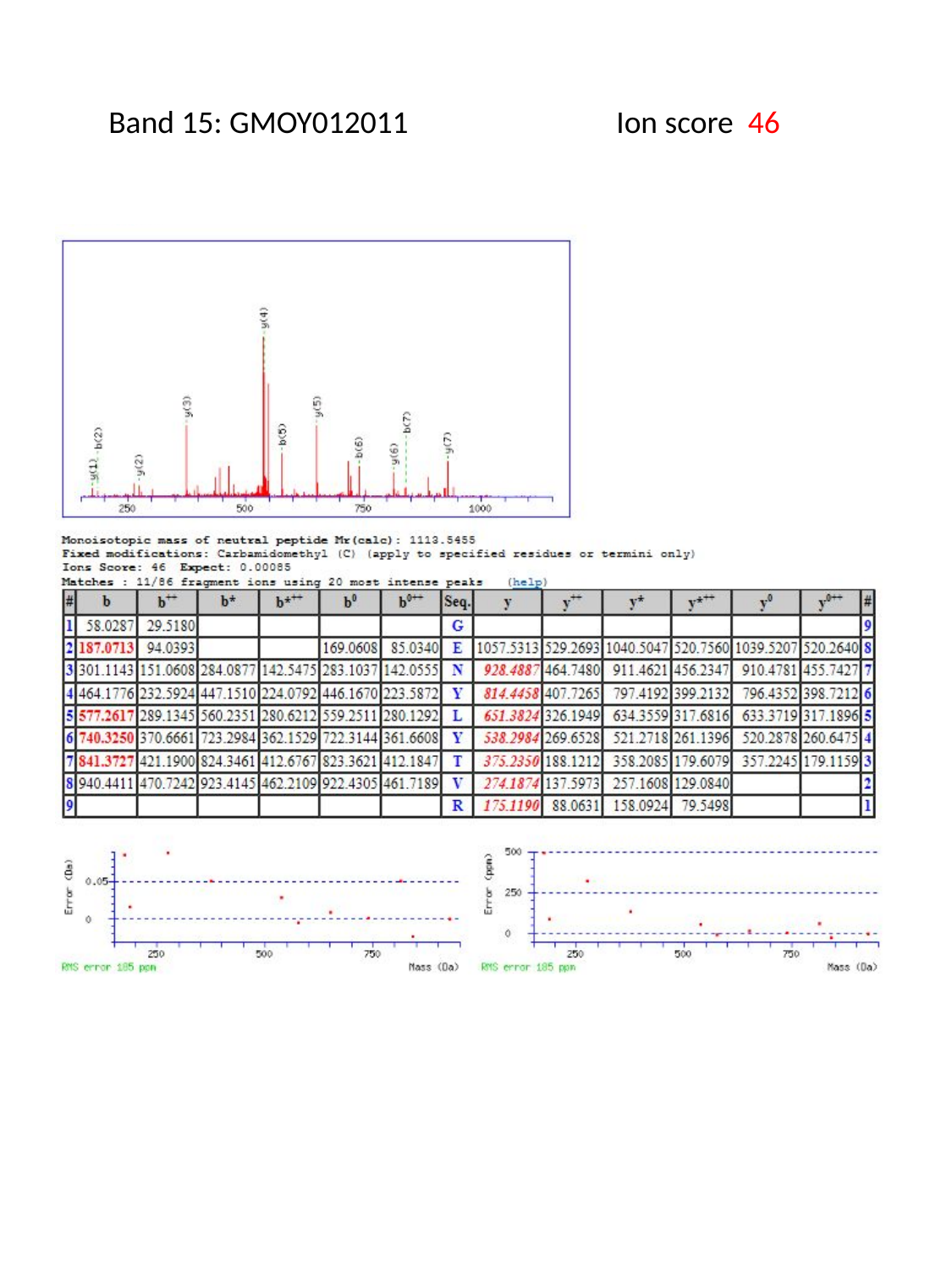

Band 15: GMOY012011 		Ion score 46

## Slide 37
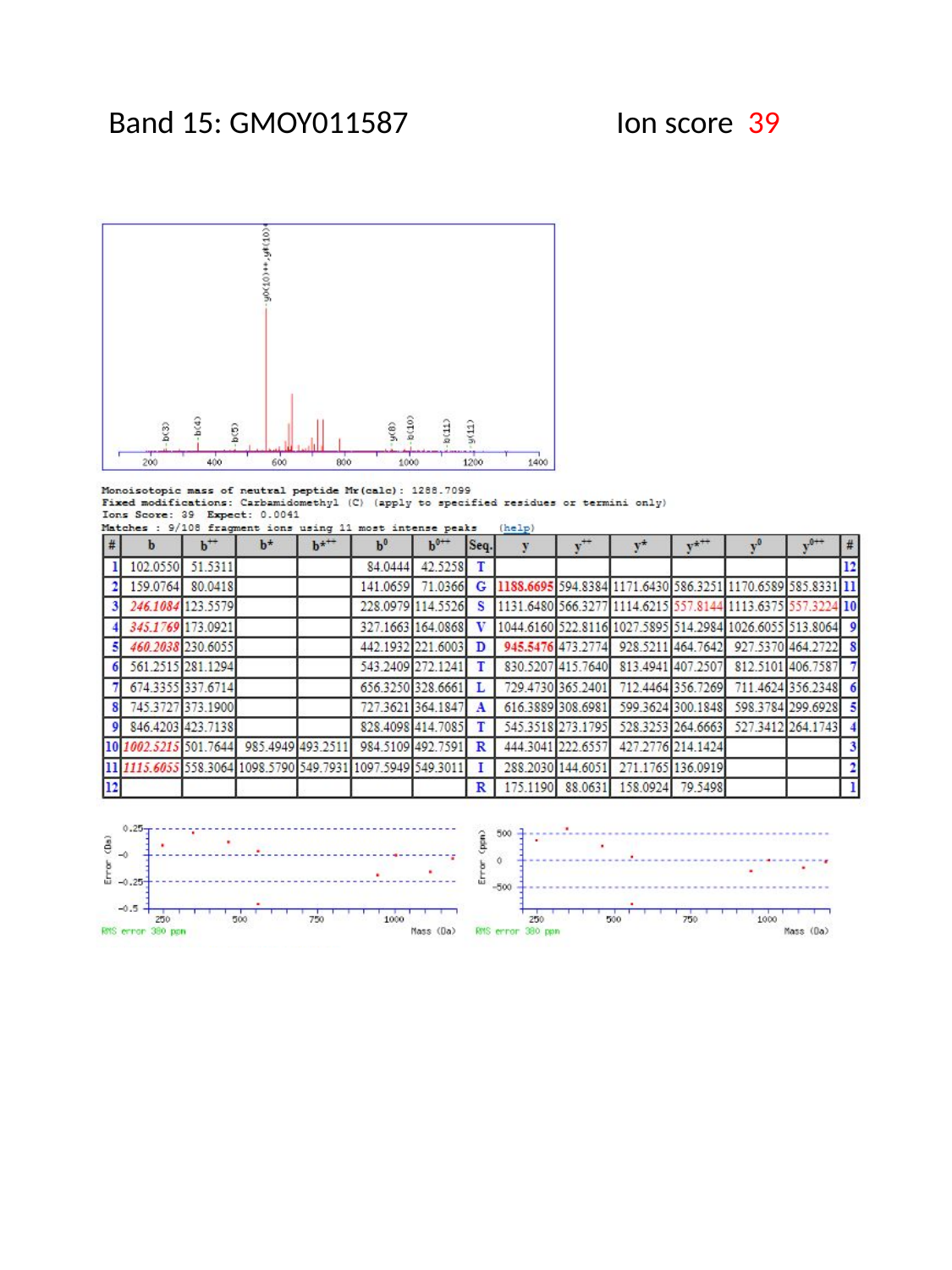

Band 15: GMOY011587 		Ion score 39

## Slide 38
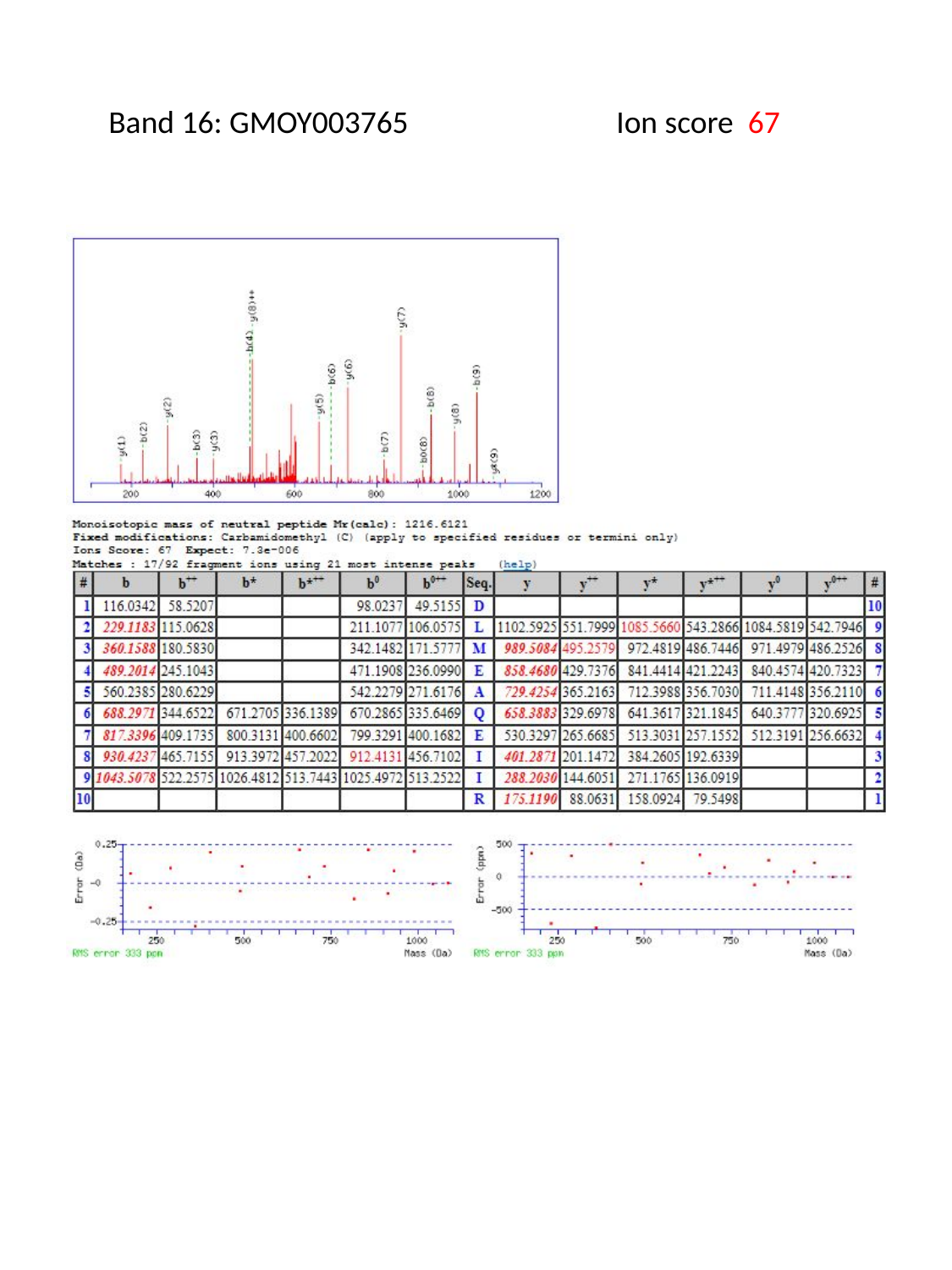

Band 16: GMOY003765 		Ion score 67

## Slide 39
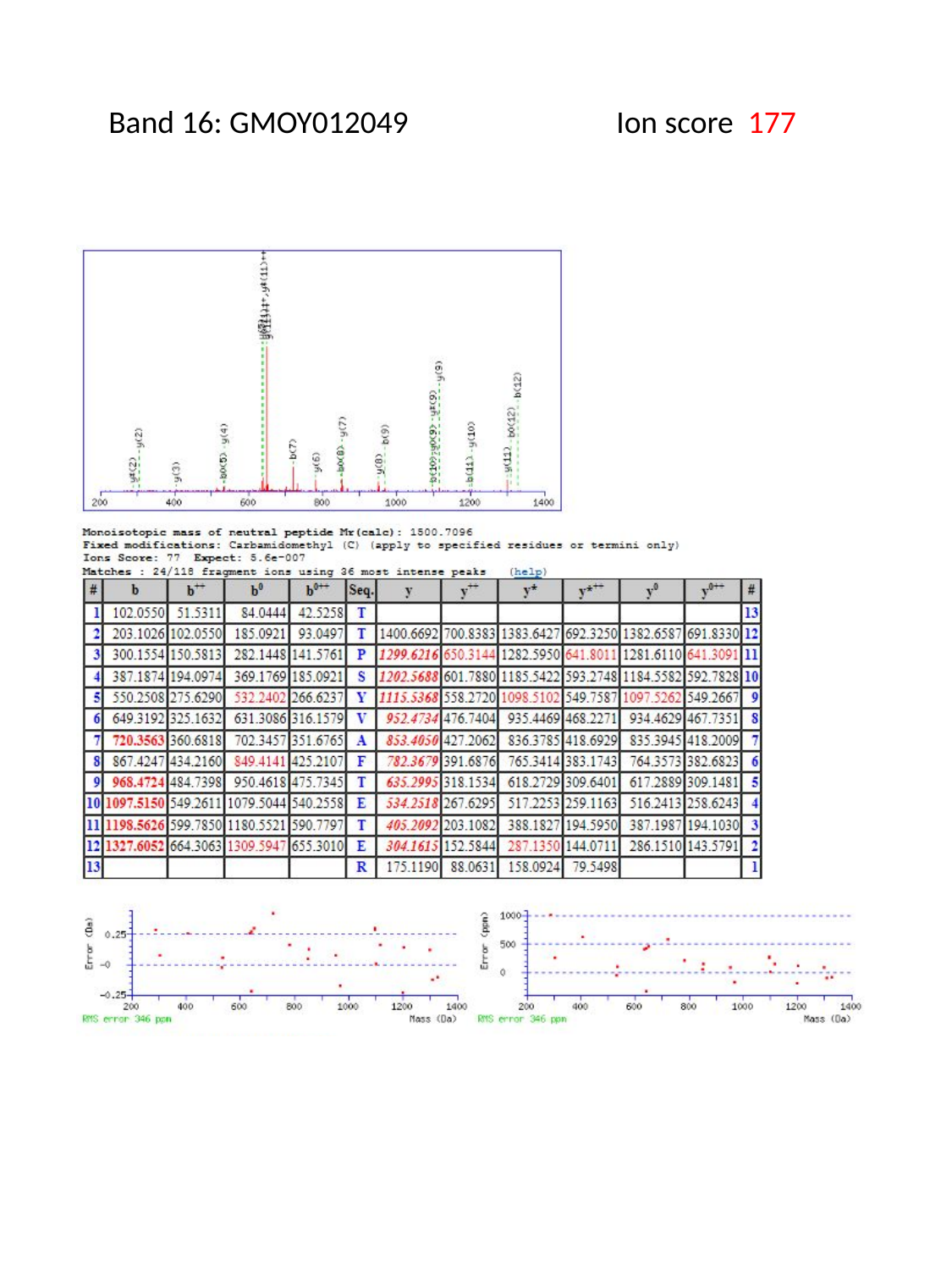

Band 16: GMOY012049 		Ion score 177

## Slide 40
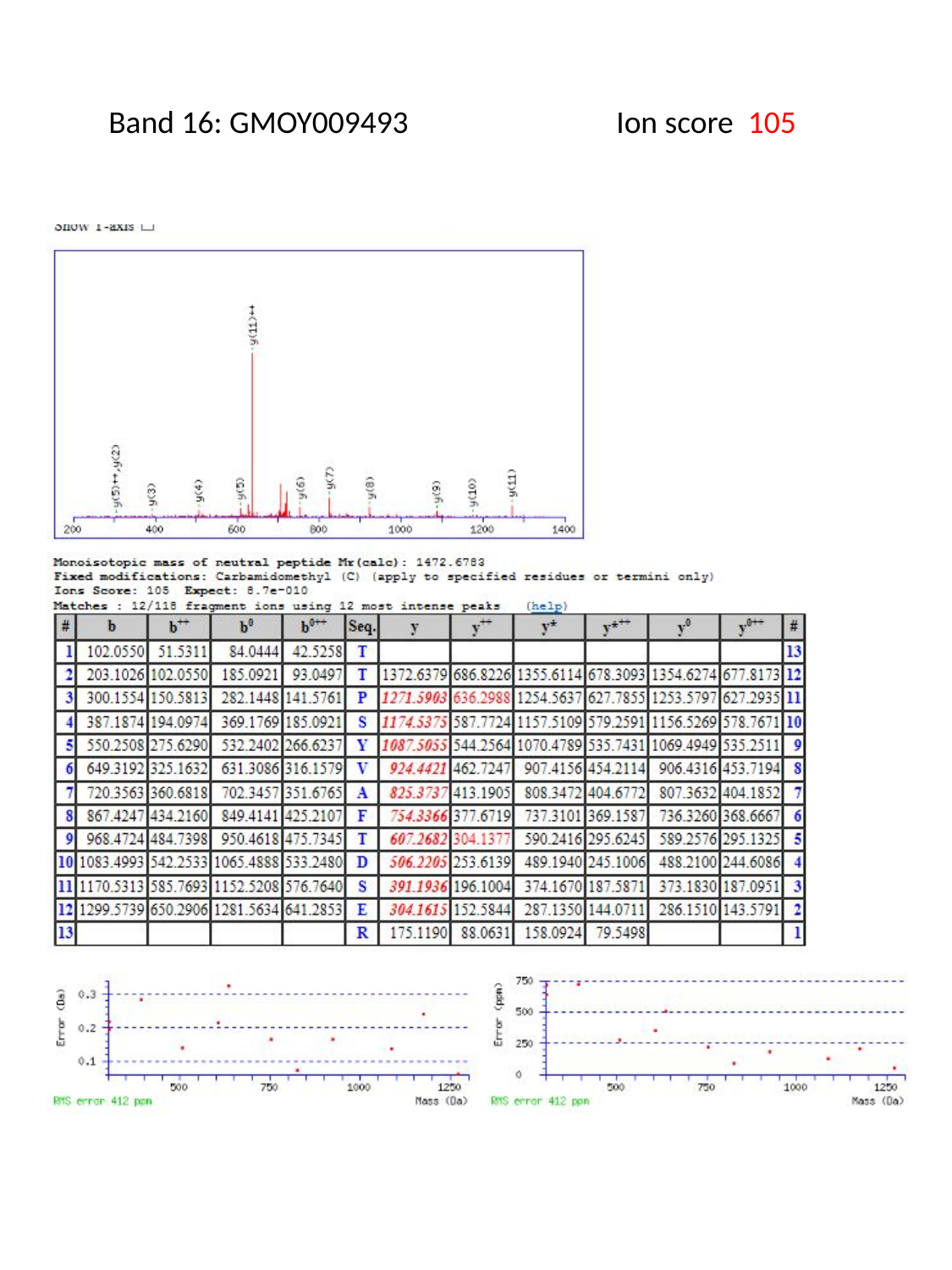

Band 16: GMOY009493 		Ion score 105

## Slide 41
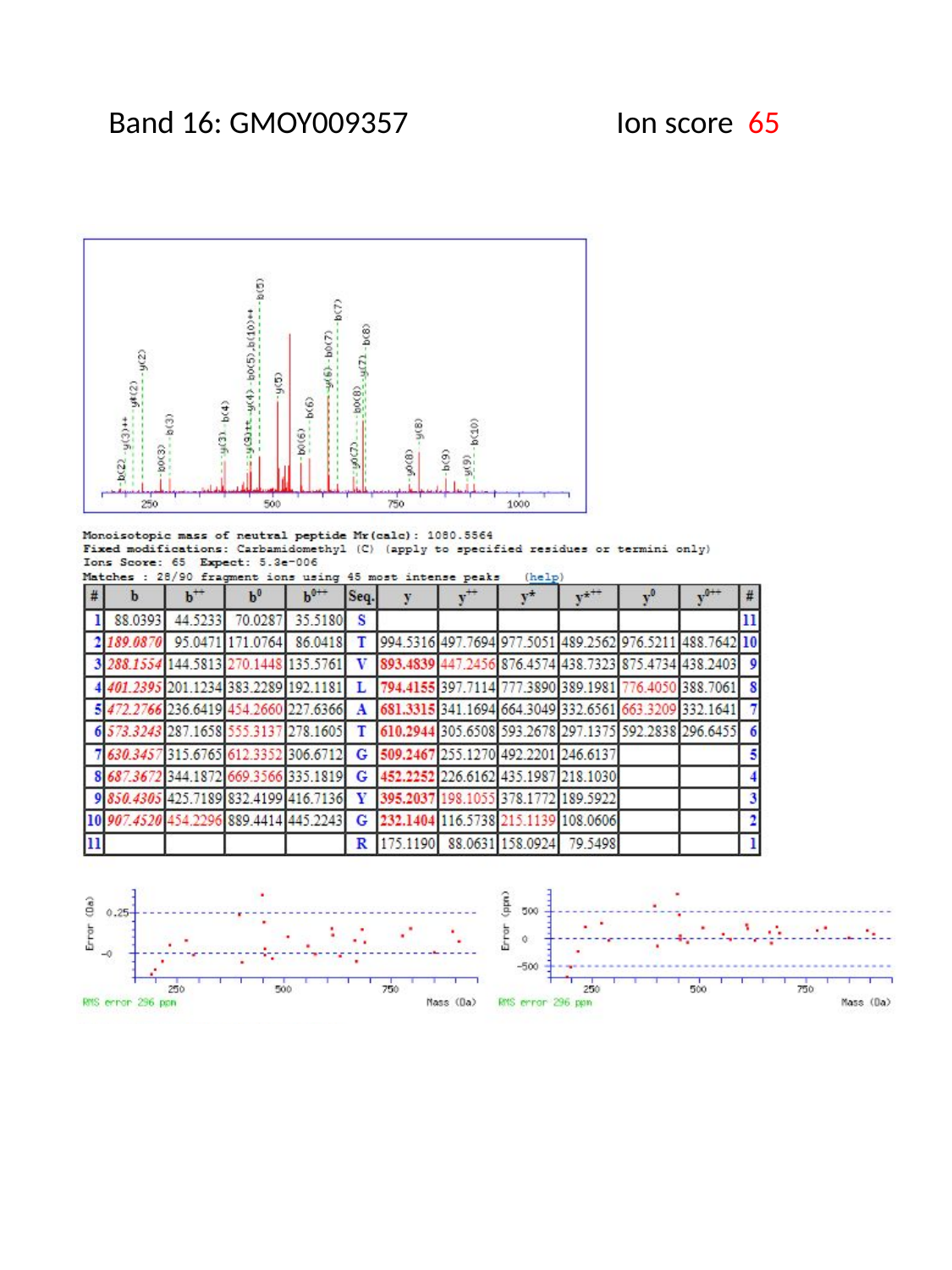

Band 16: GMOY009357 		Ion score 65

## Slide 42
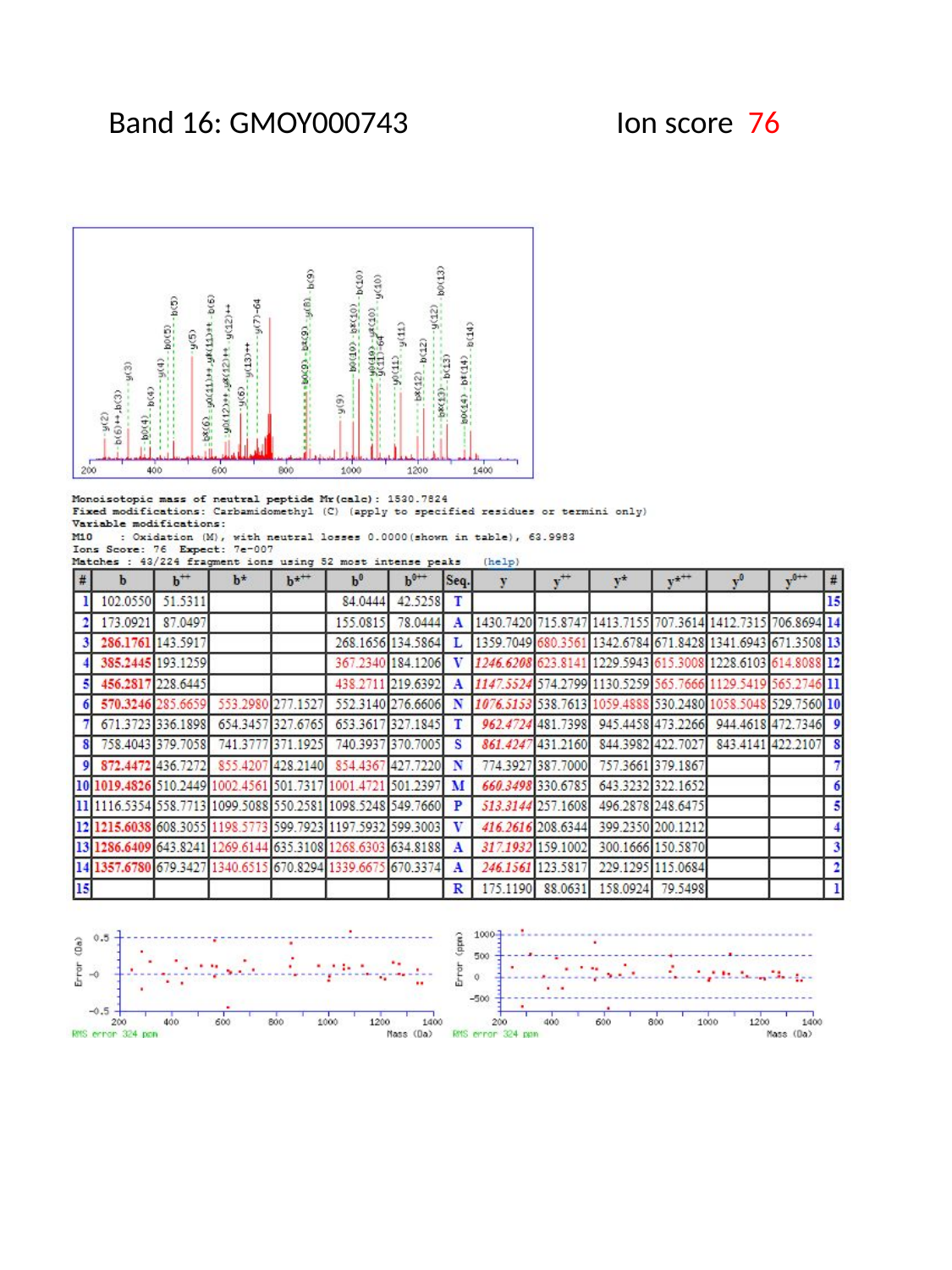

Band 16: GMOY000743 		Ion score 76

## Slide 43
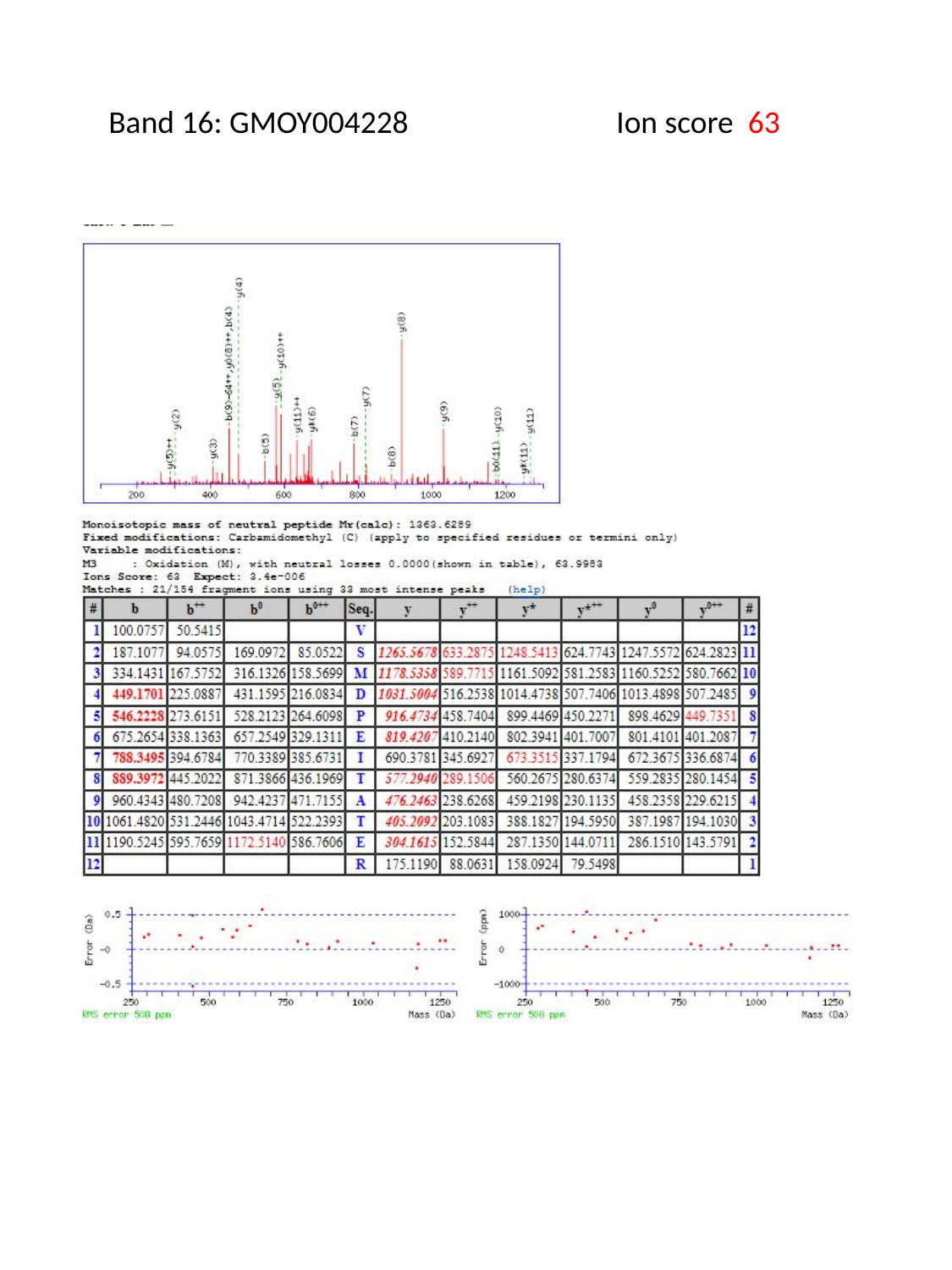

Band 16: GMOY004228 		Ion score 63

## Slide 44
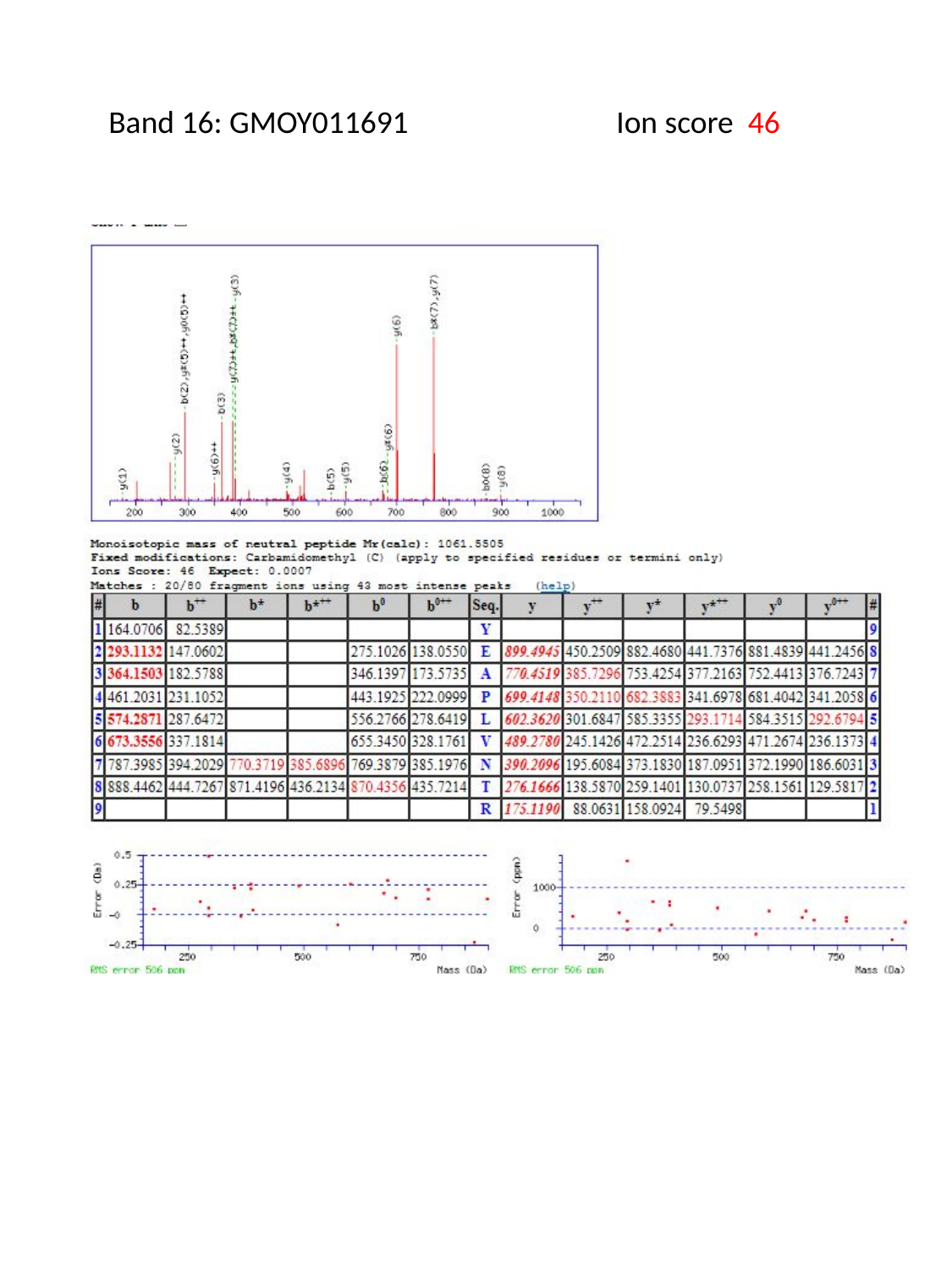

Band 16: GMOY011691 		Ion score 46

## Slide 45
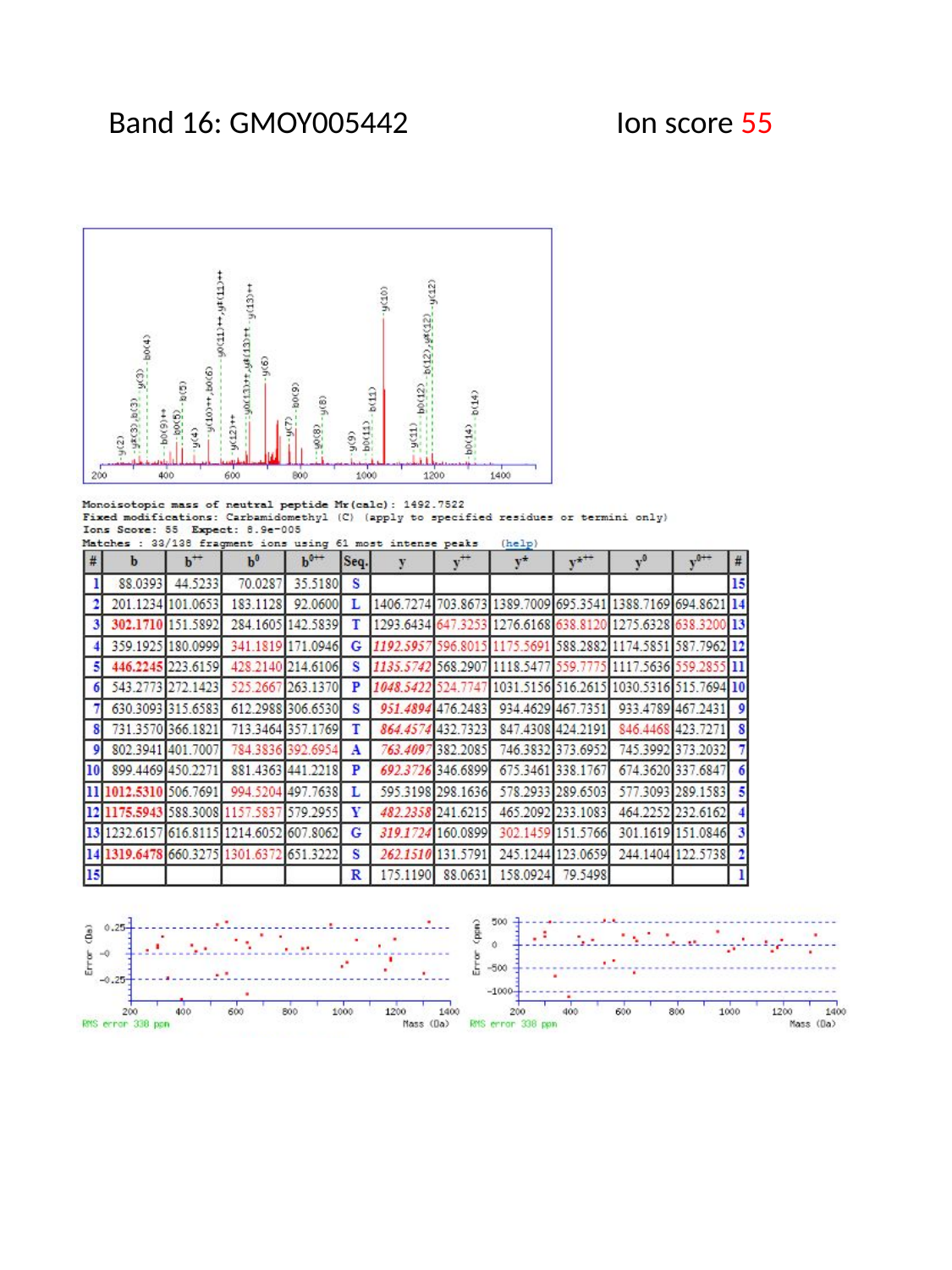

Band 16: GMOY005442 		Ion score 55

## Slide 46
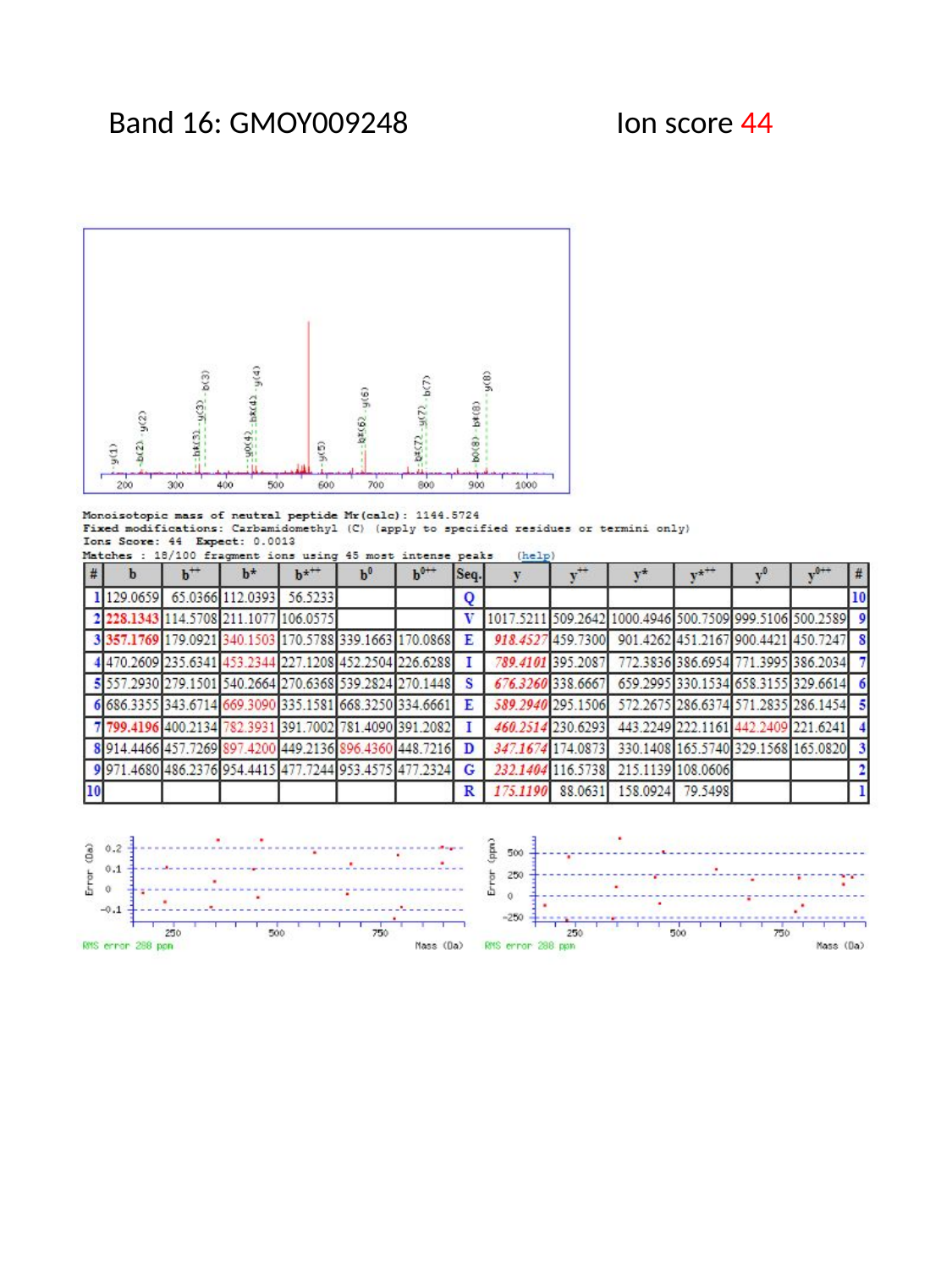

Band 16: GMOY009248 		Ion score 44

## Slide 47
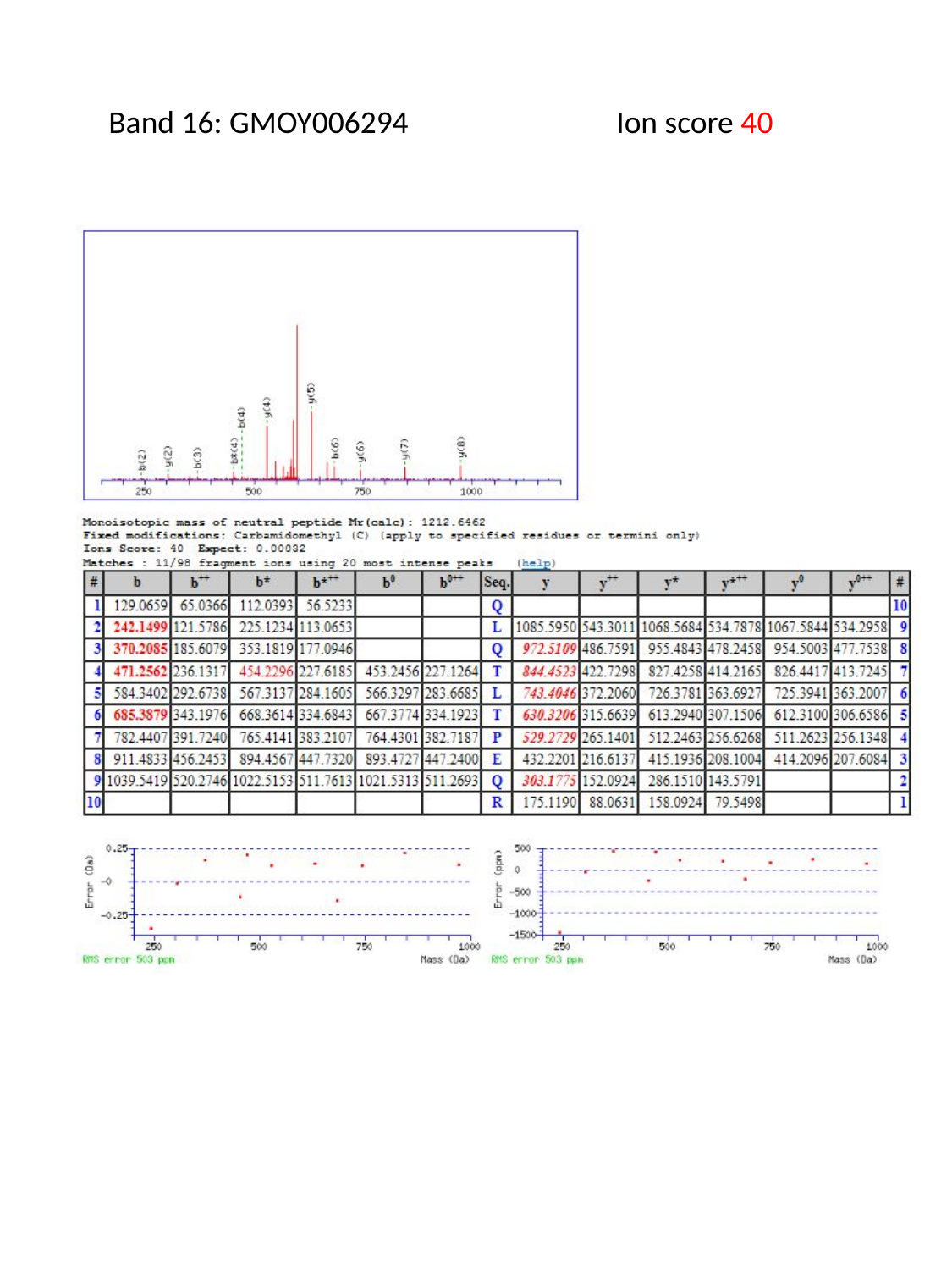

Band 16: GMOY006294 		Ion score 40

## Slide 48
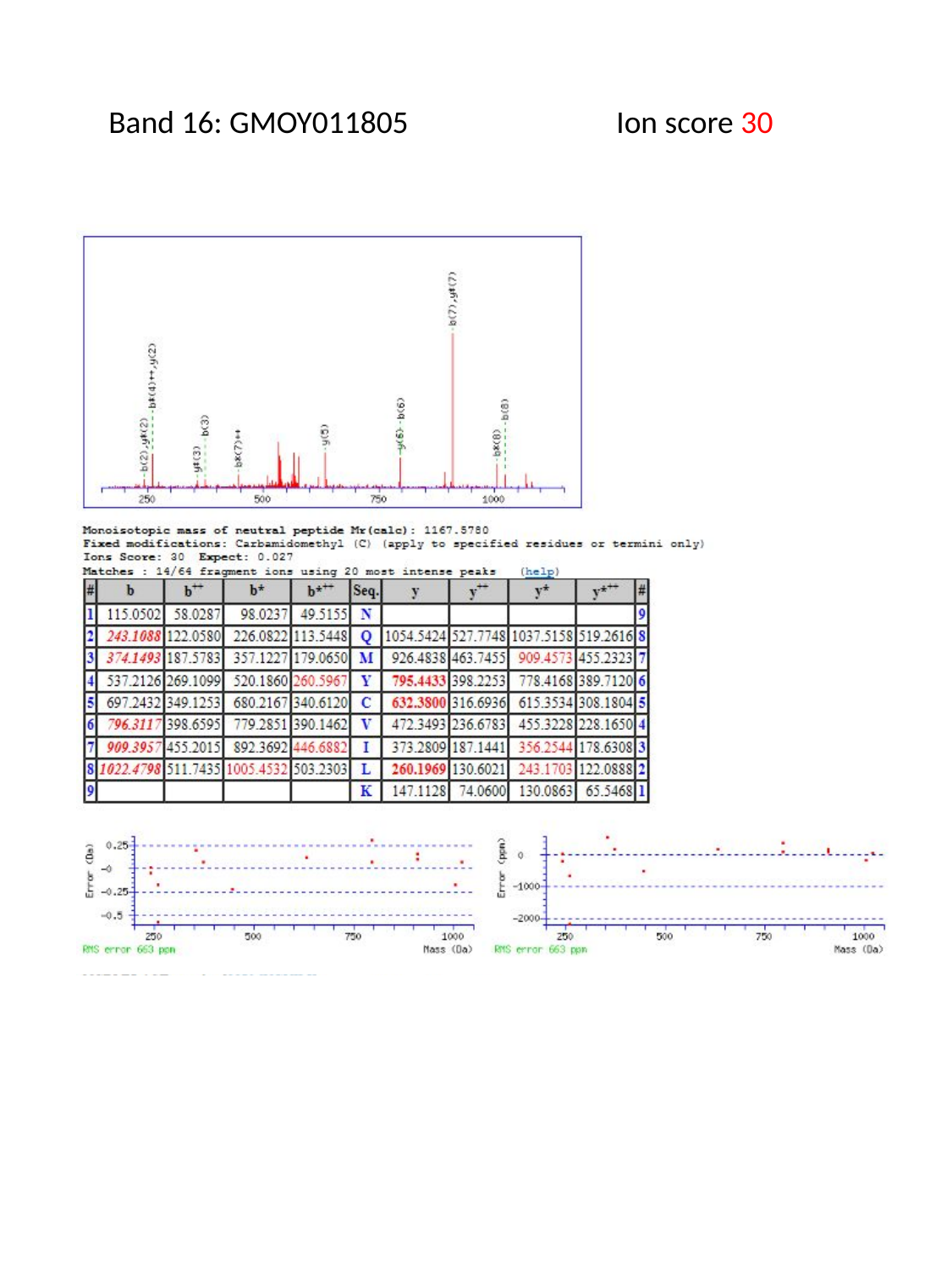

Band 16: GMOY011805 		Ion score 30

## Slide 49
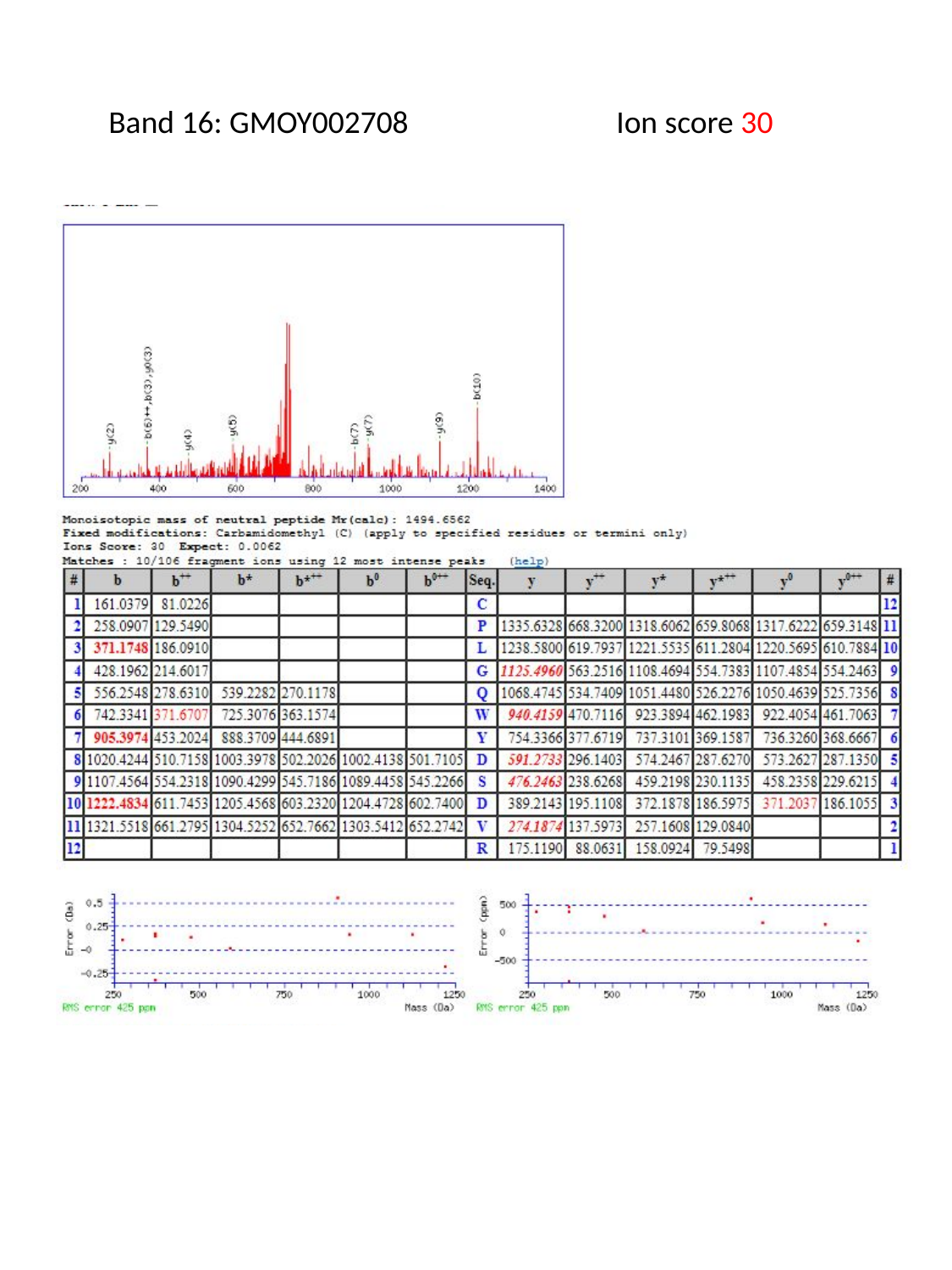

Band 16: GMOY002708 		Ion score 30

## Slide 50
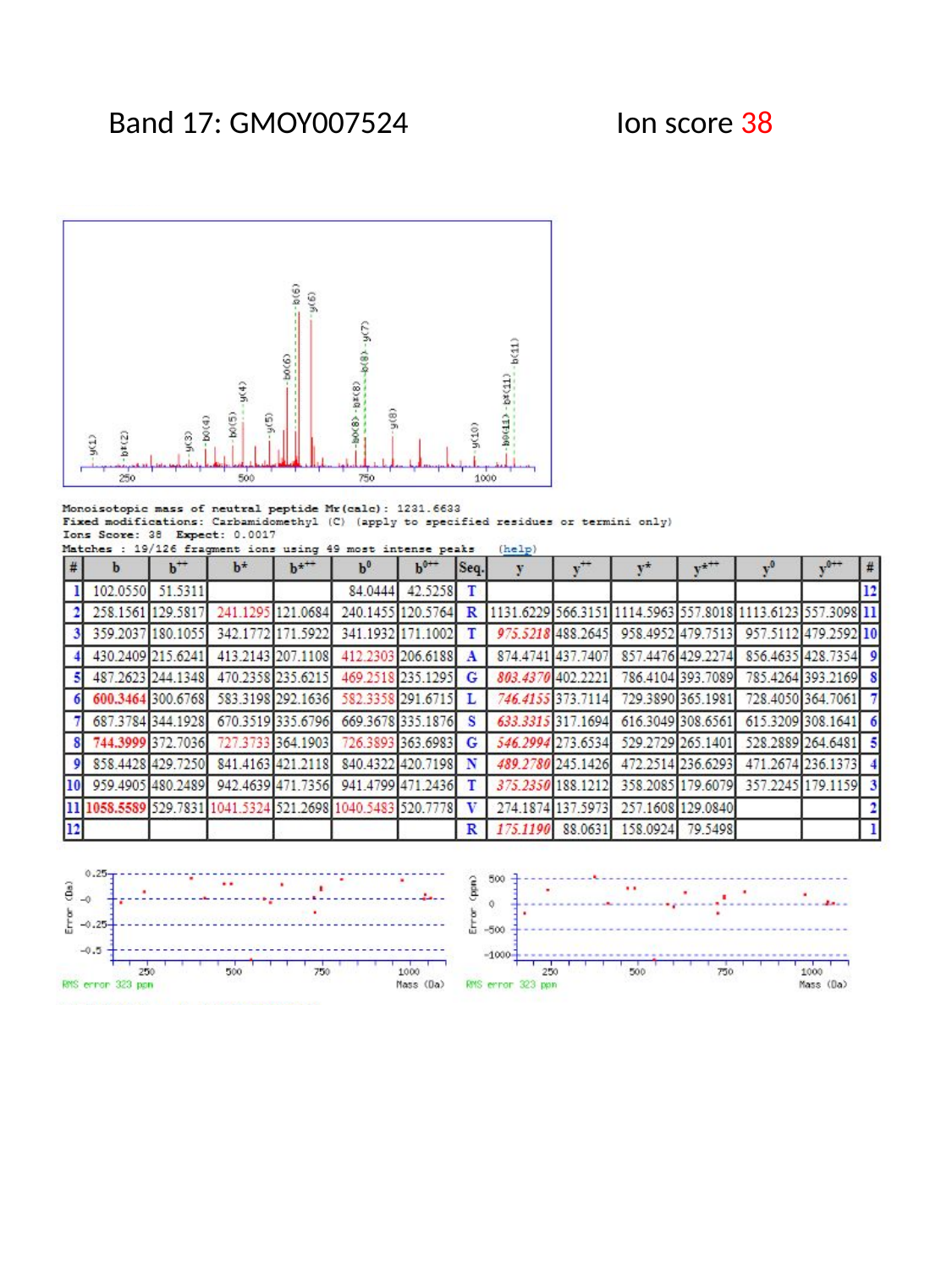

Band 17: GMOY007524 		Ion score 38

## Slide 51
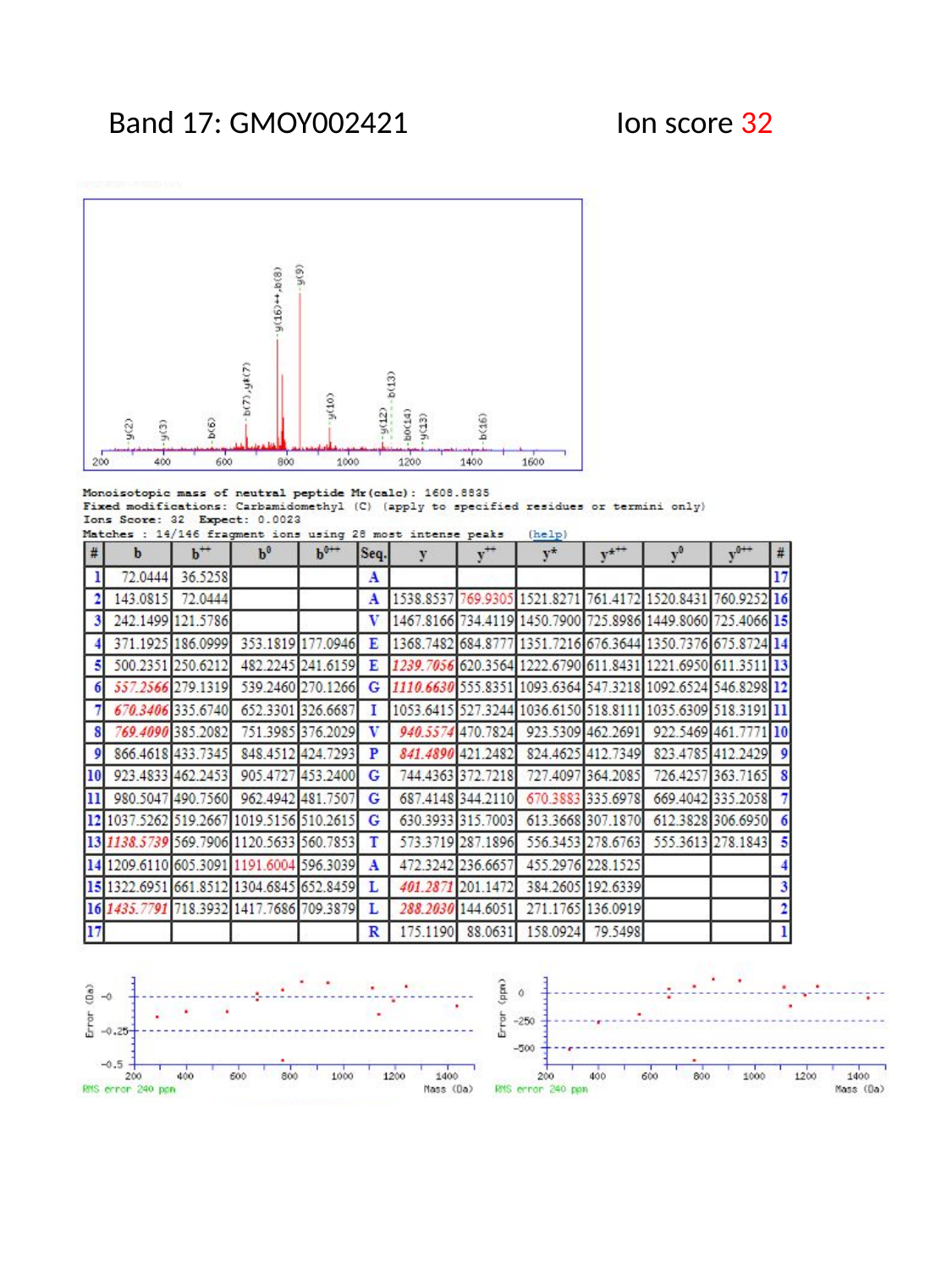

Band 17: GMOY002421 		Ion score 32

## Slide 52
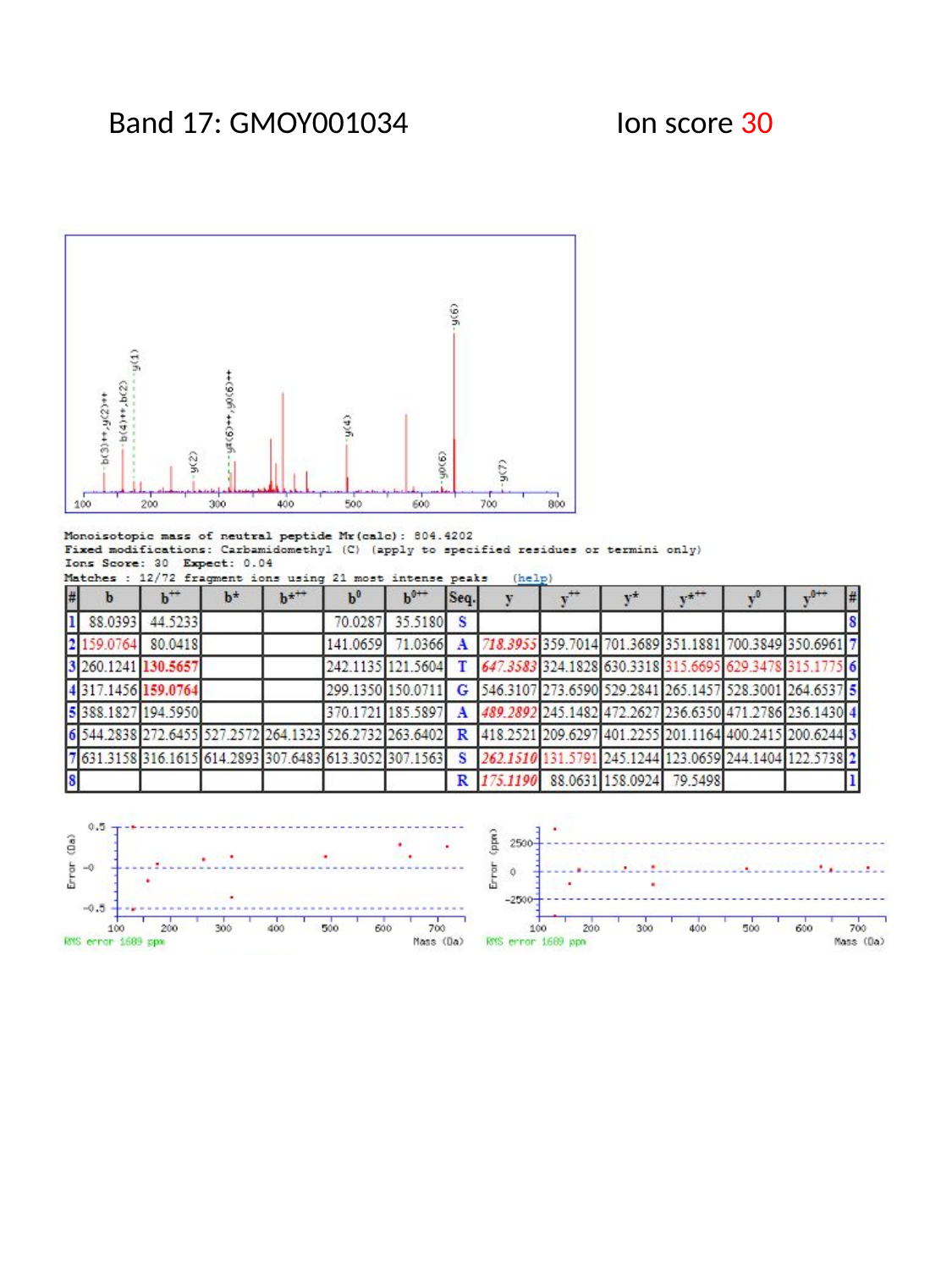

Band 17: GMOY001034 		Ion score 30

## Slide 53
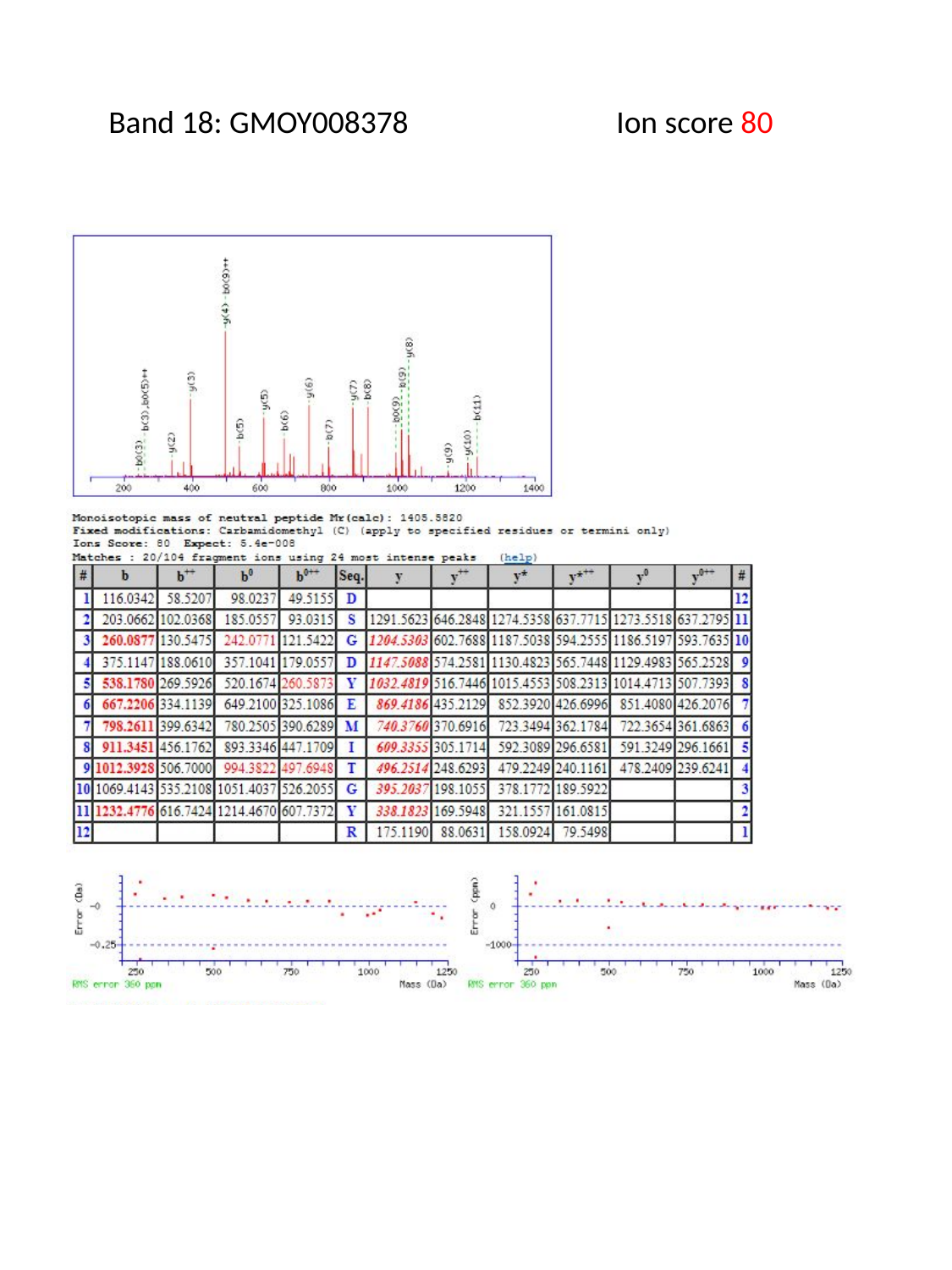

Band 18: GMOY008378 		Ion score 80

## Slide 54
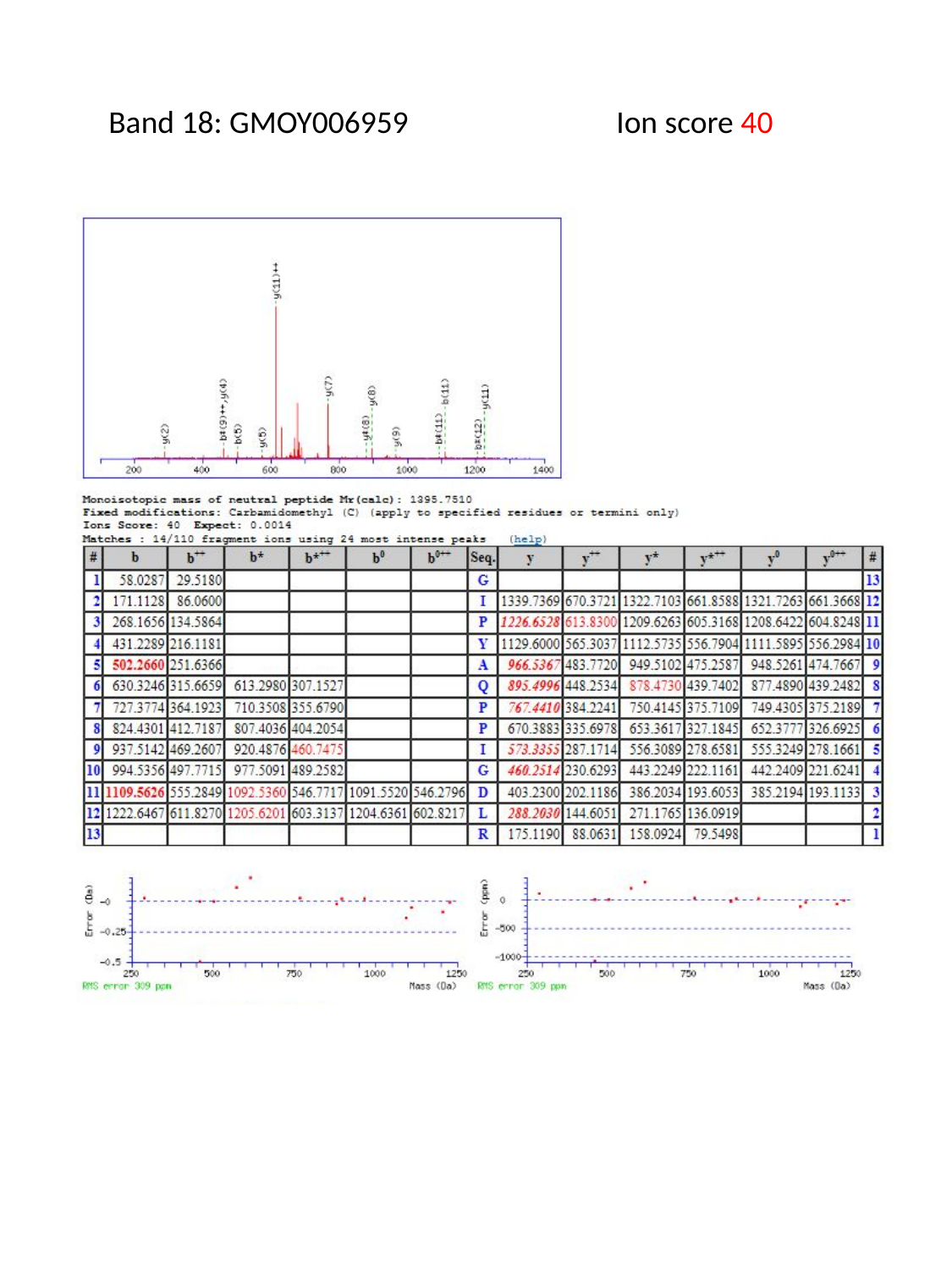

Band 18: GMOY006959 		Ion score 40

## Slide 55
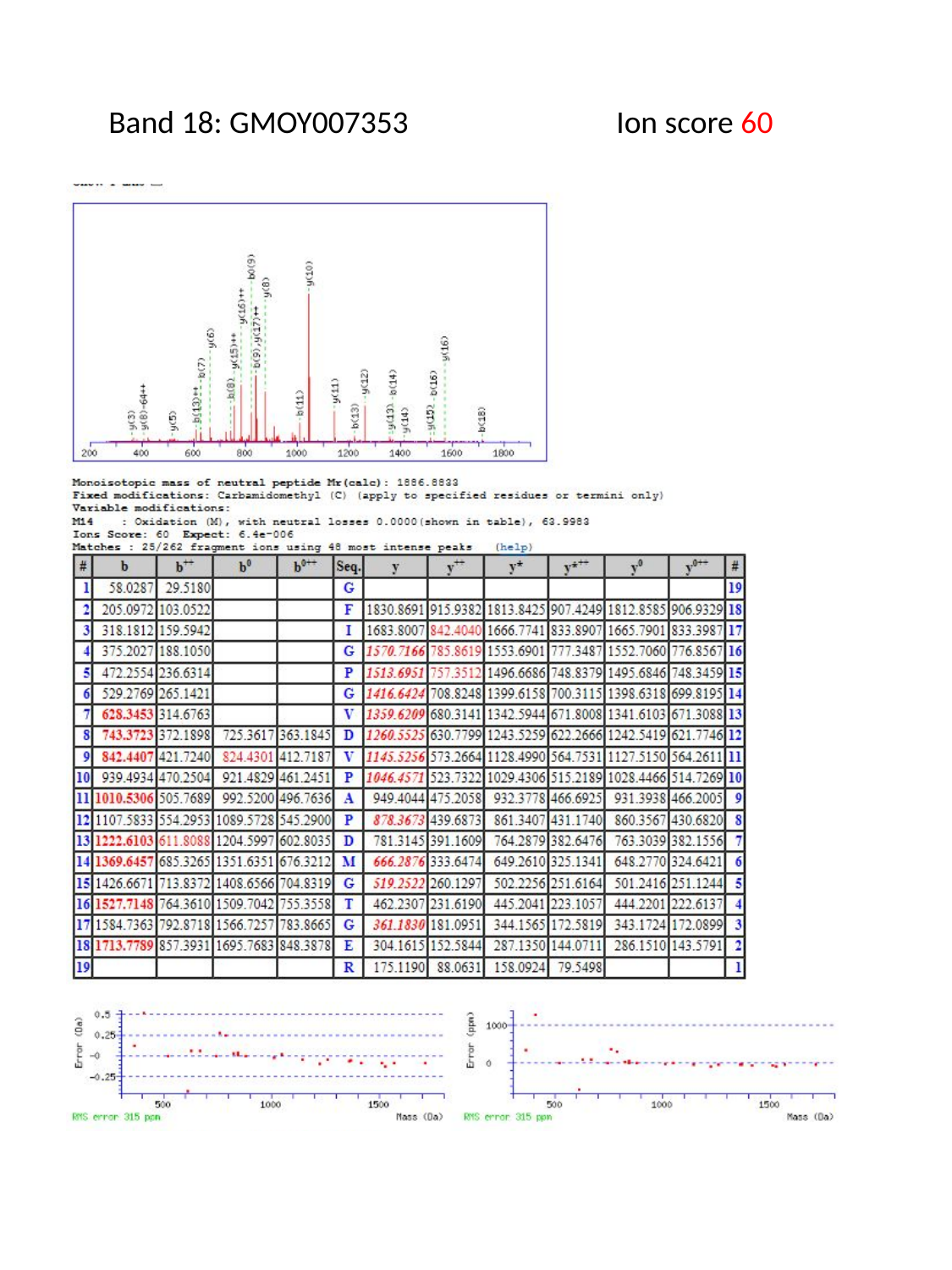

Band 18: GMOY007353 		Ion score 60

## Slide 56
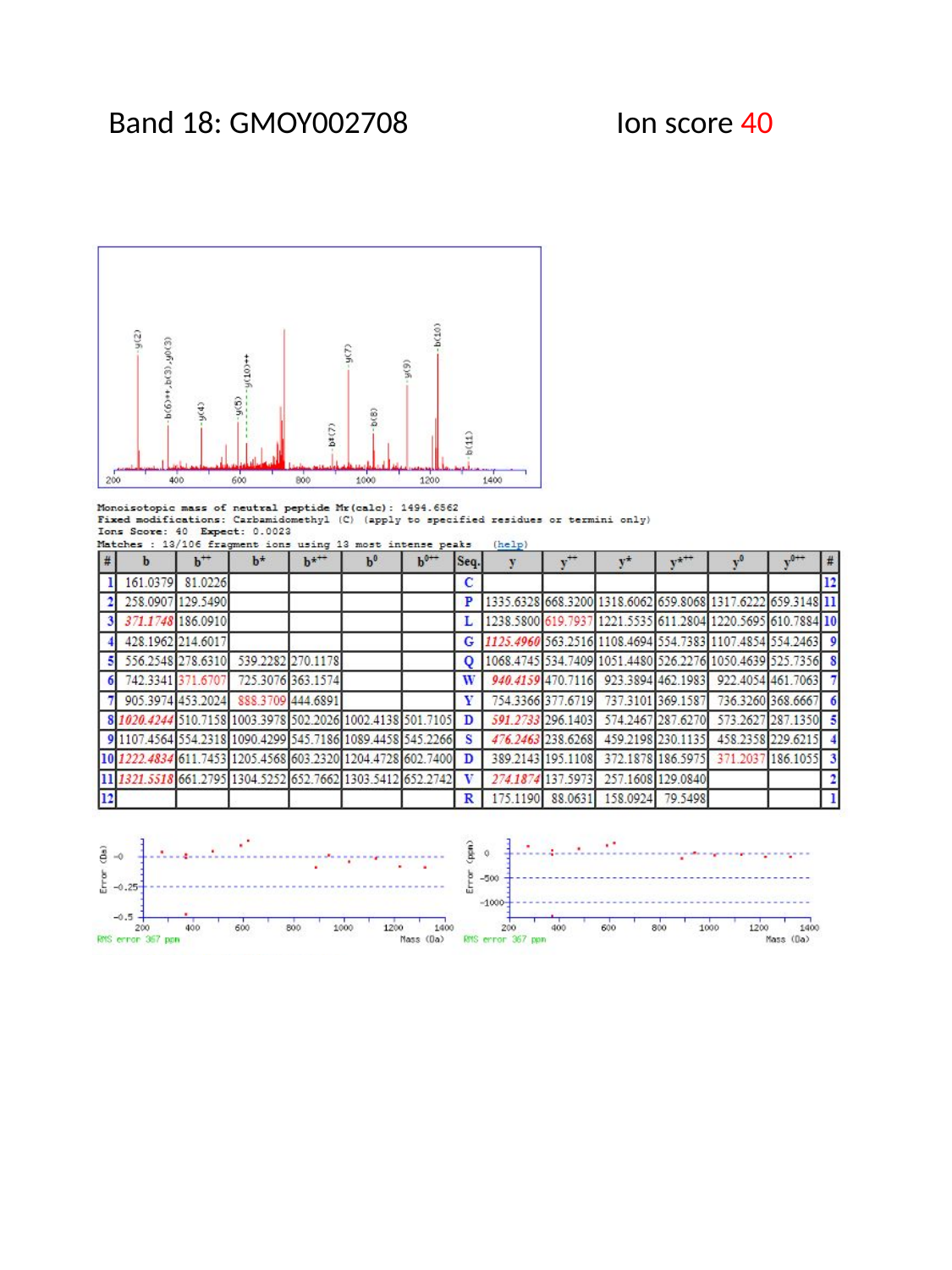

Band 18: GMOY002708 		Ion score 40

## Slide 57
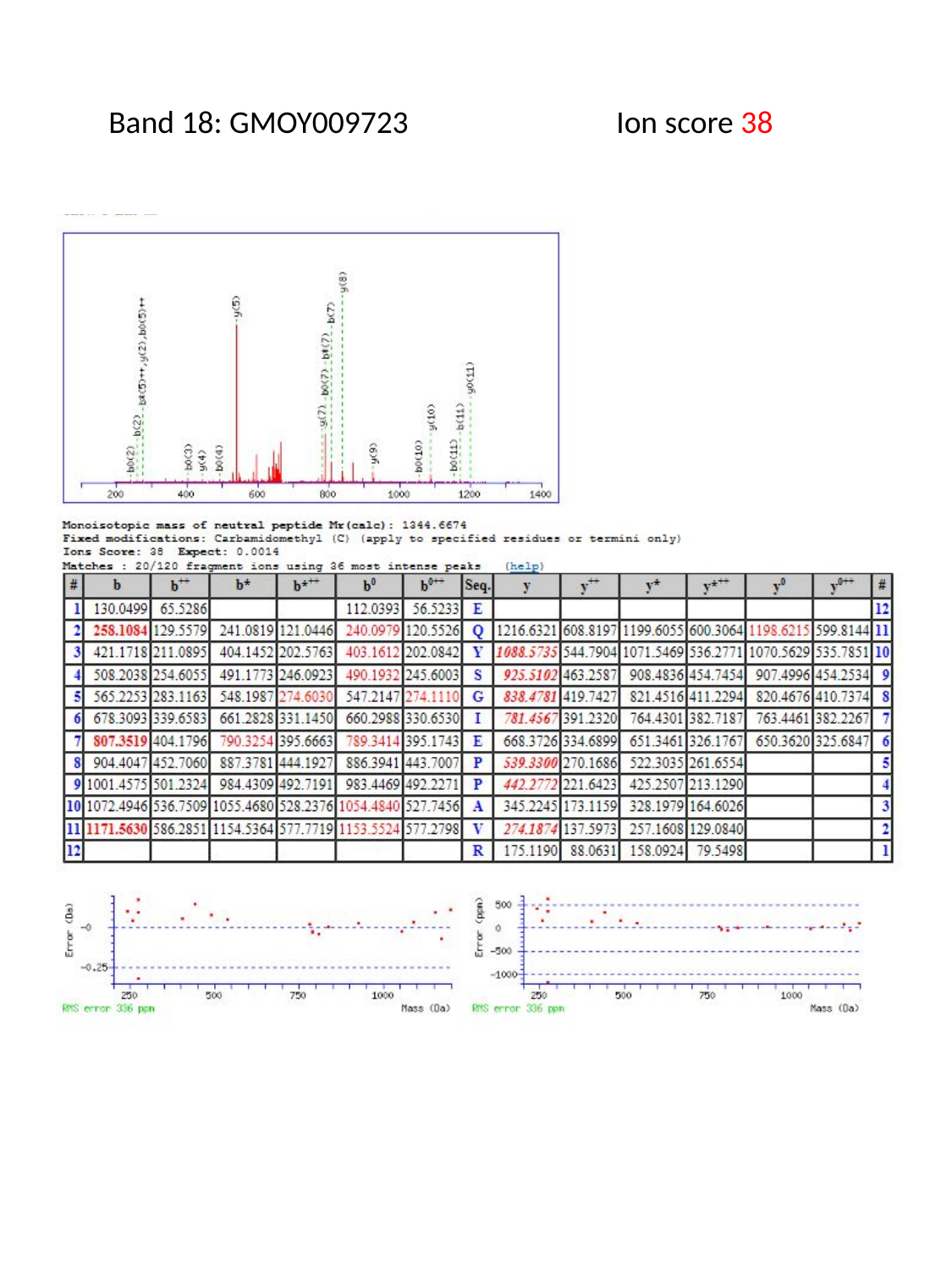

Band 18: GMOY009723 		Ion score 38

## Slide 58
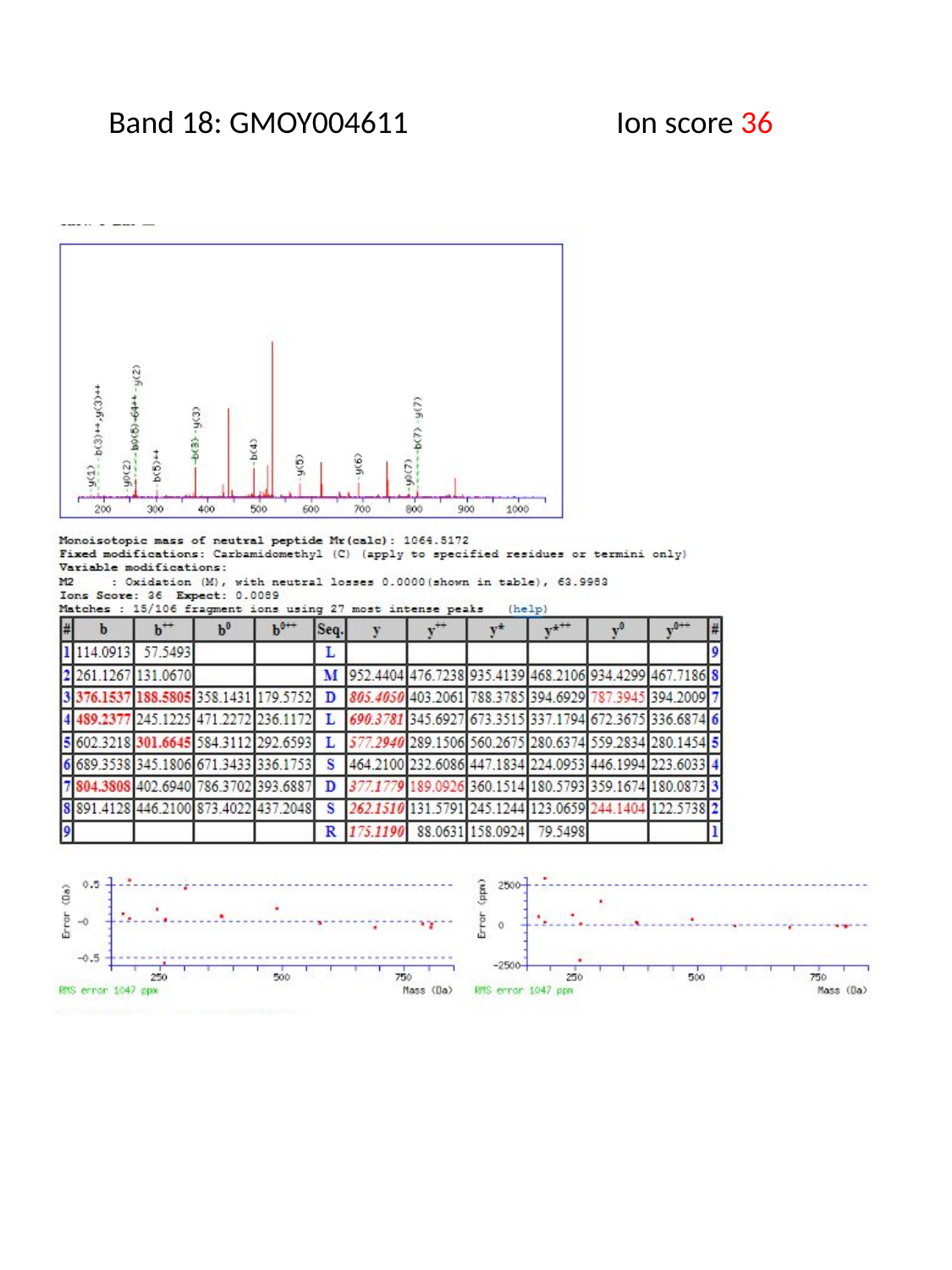

Band 18: GMOY004611 		Ion score 36

## Slide 59
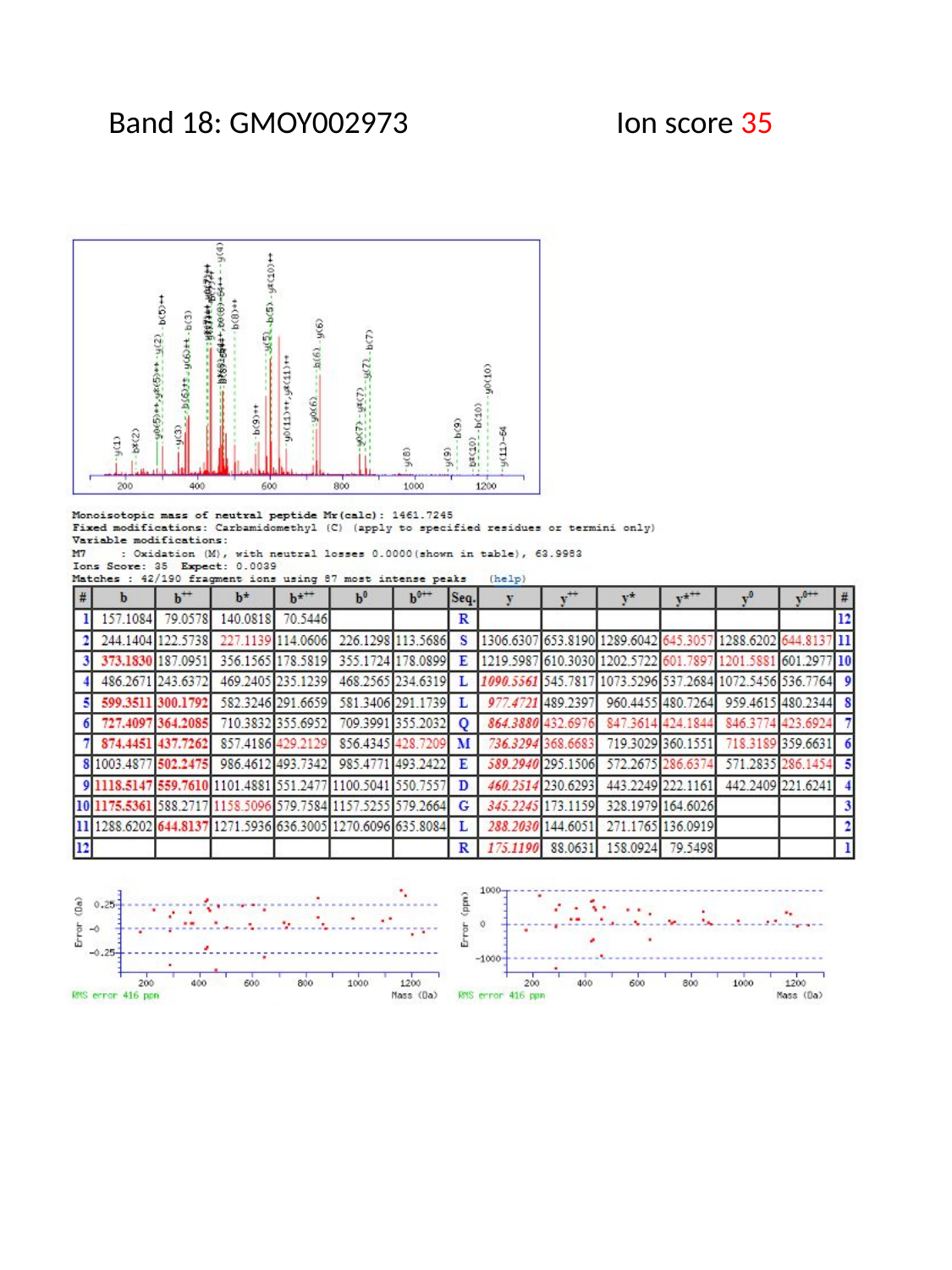

Band 18: GMOY002973 		Ion score 35

## Slide 60
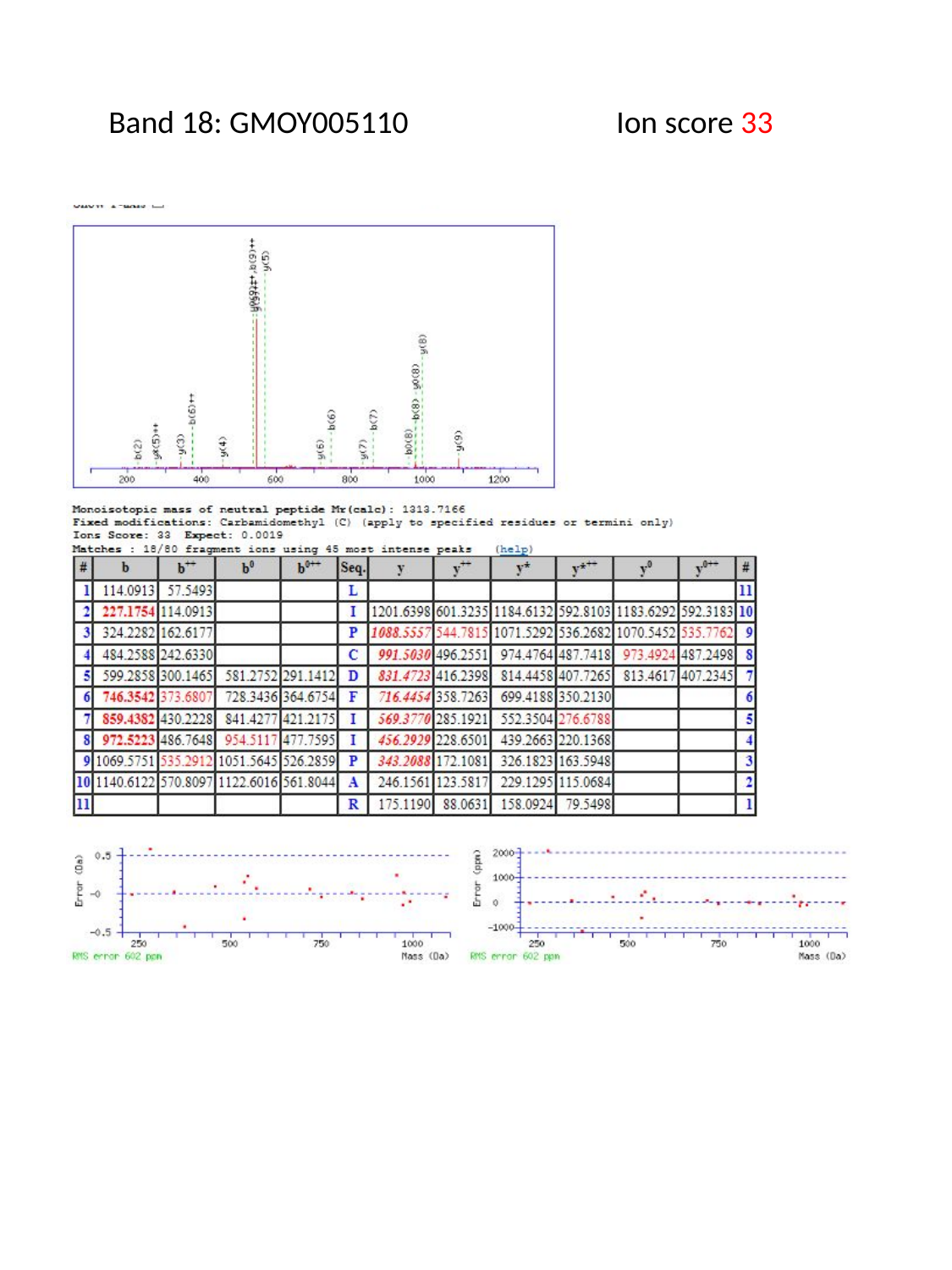

Band 18: GMOY005110 		Ion score 33

## Slide 61
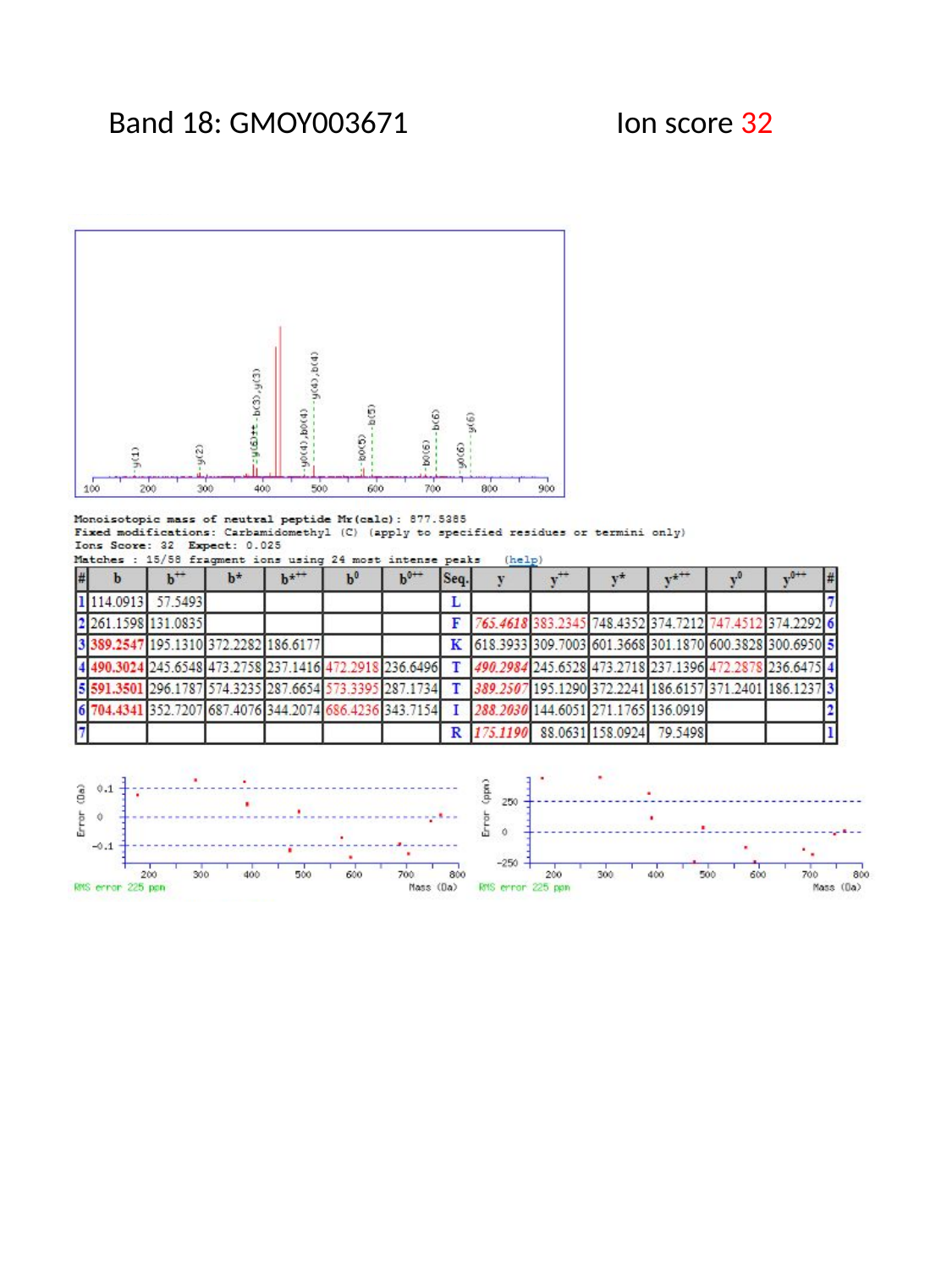

Band 18: GMOY003671 		Ion score 32

## Slide 62
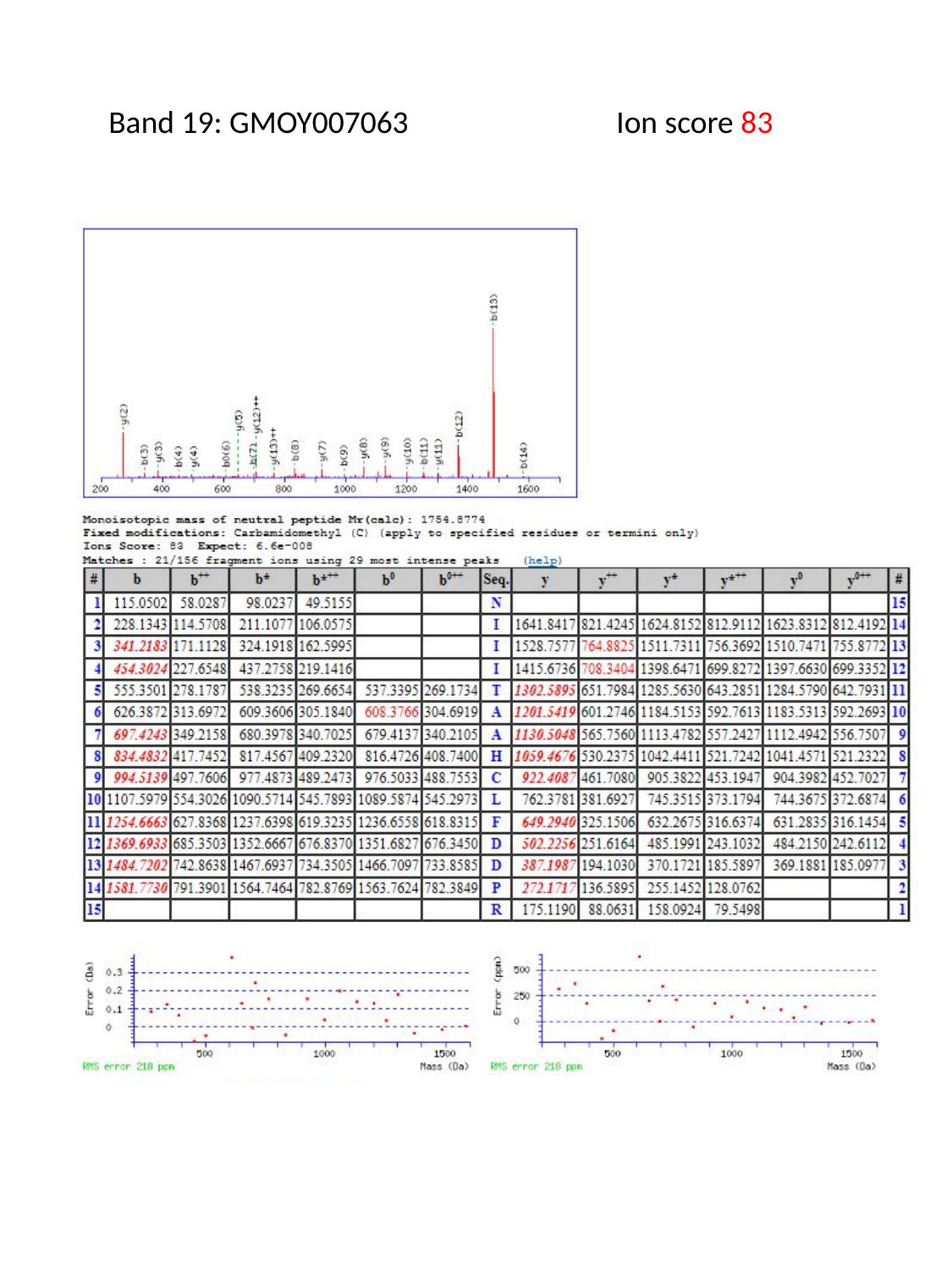

Band 19: GMOY007063 		Ion score 83

## Slide 63
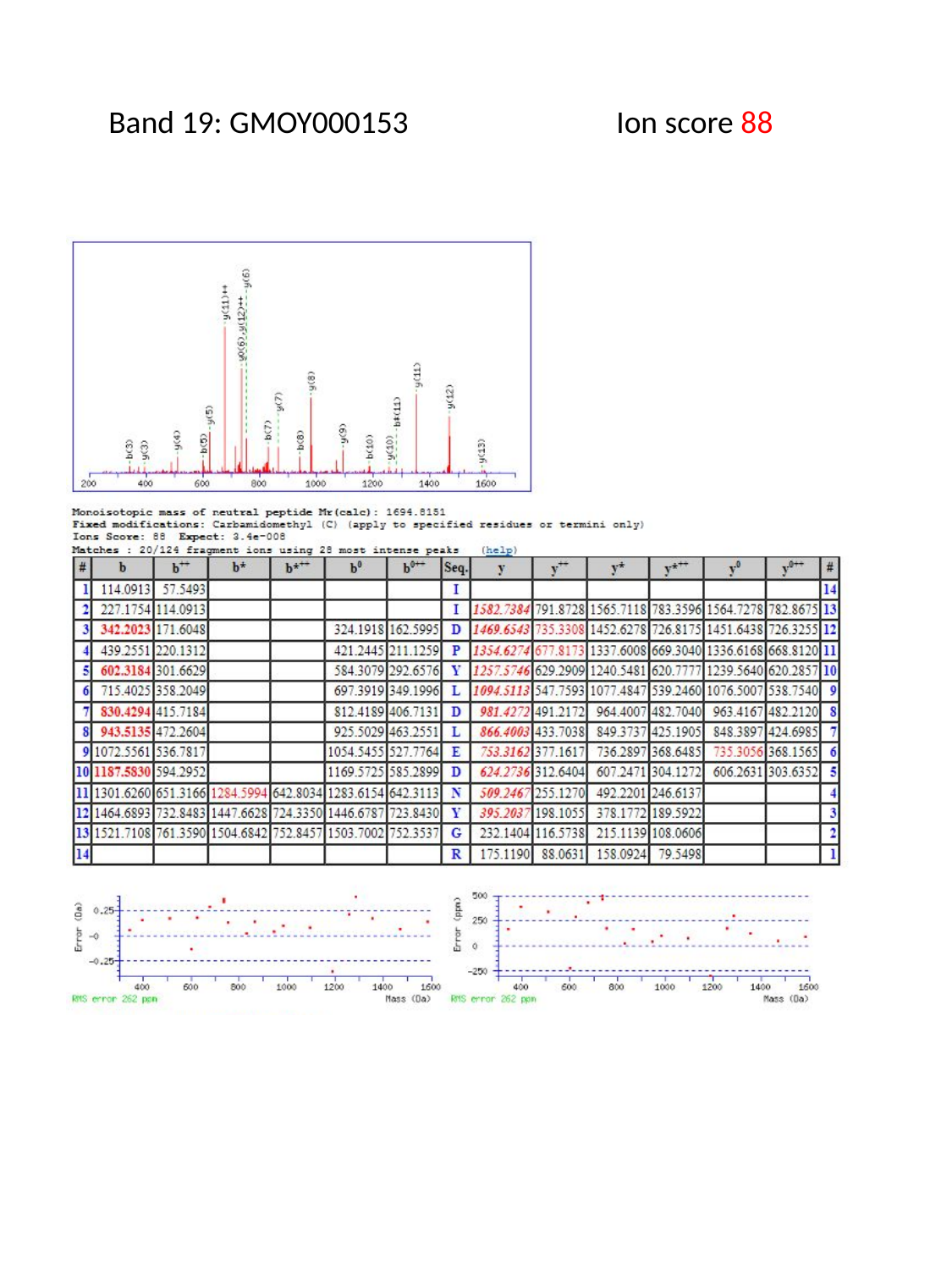

Band 19: GMOY000153 		Ion score 88

## Slide 64
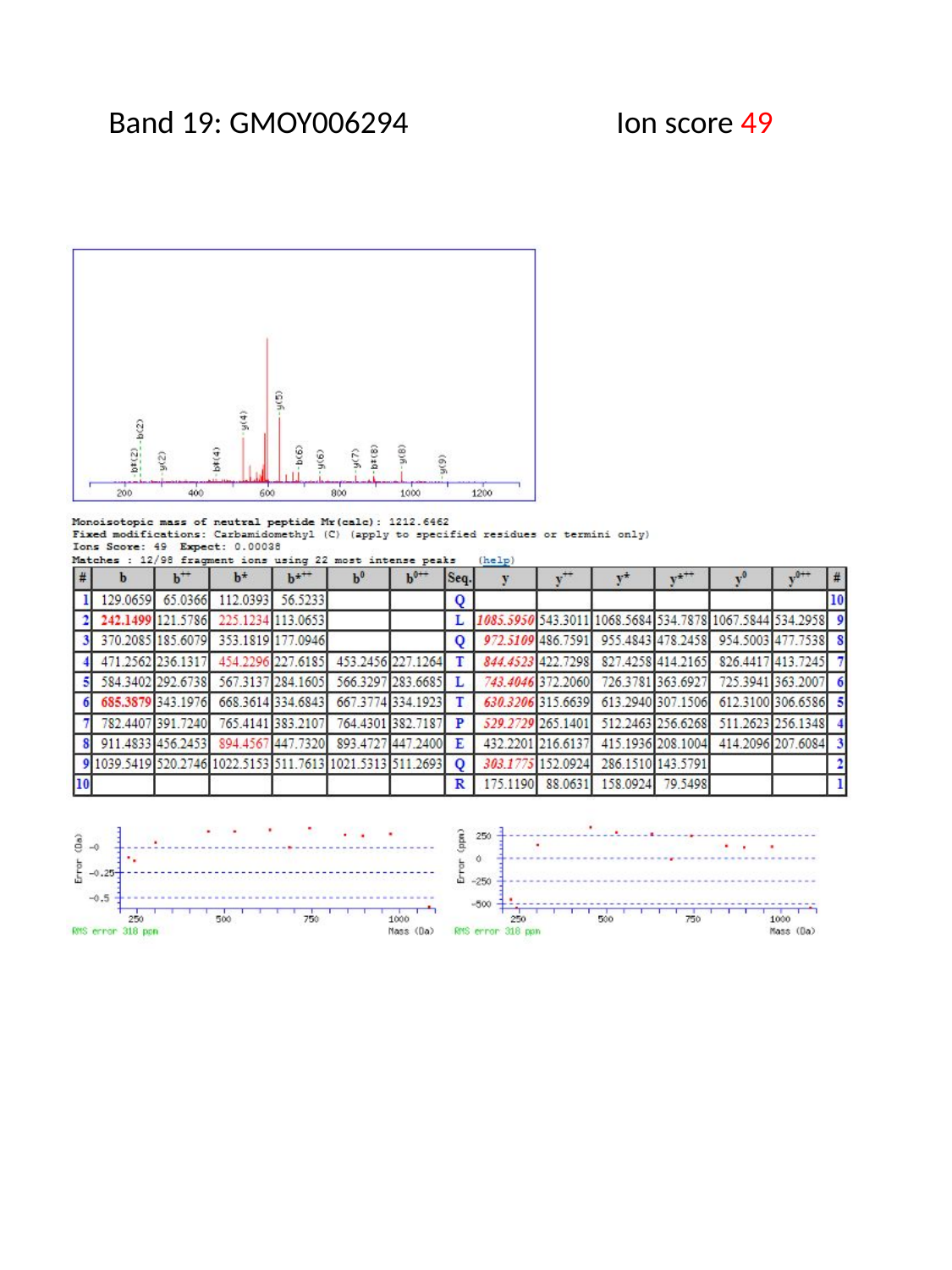

Band 19: GMOY006294 		Ion score 49

## Slide 65
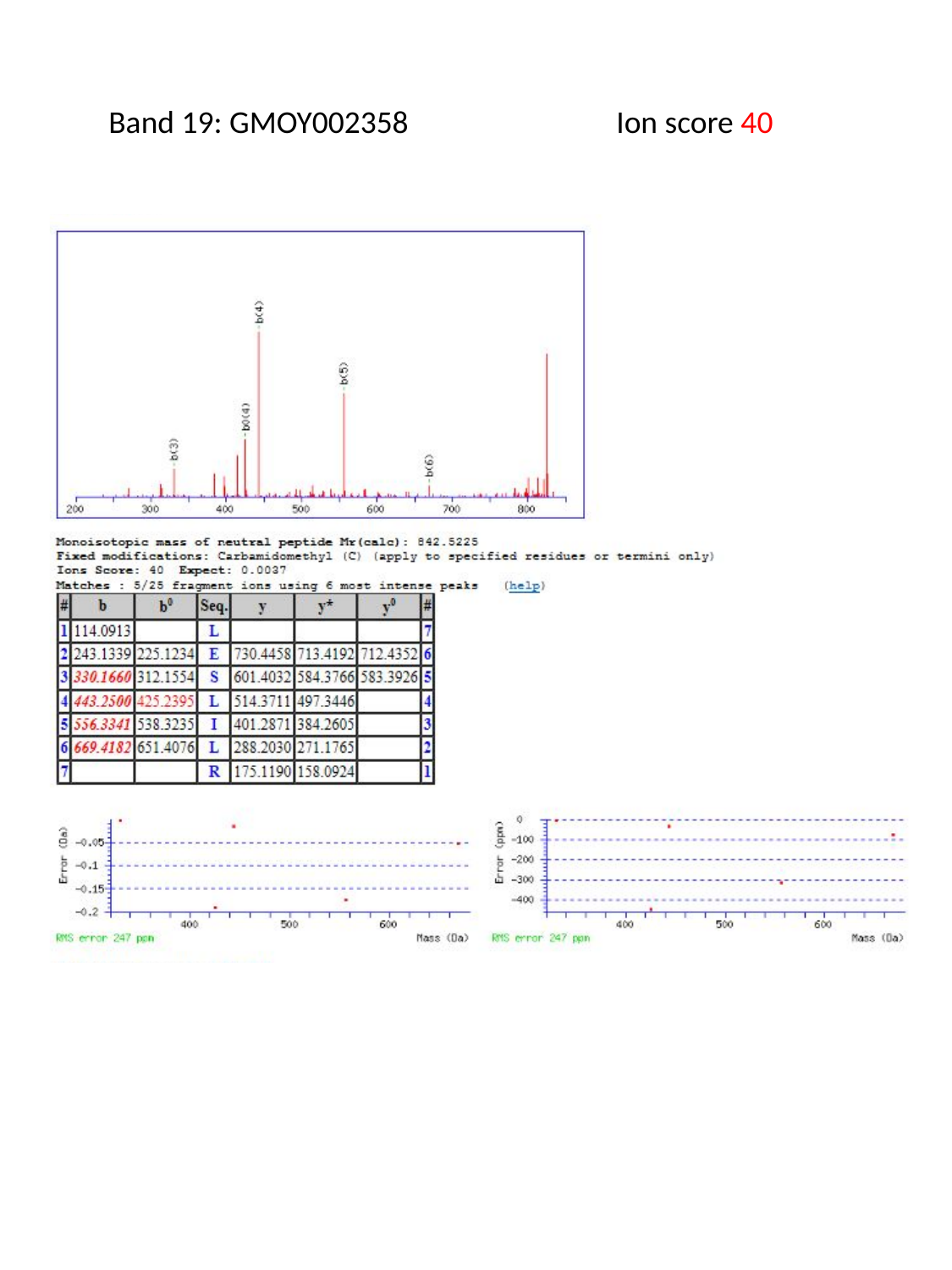

Band 19: GMOY002358 		Ion score 40

## Slide 66
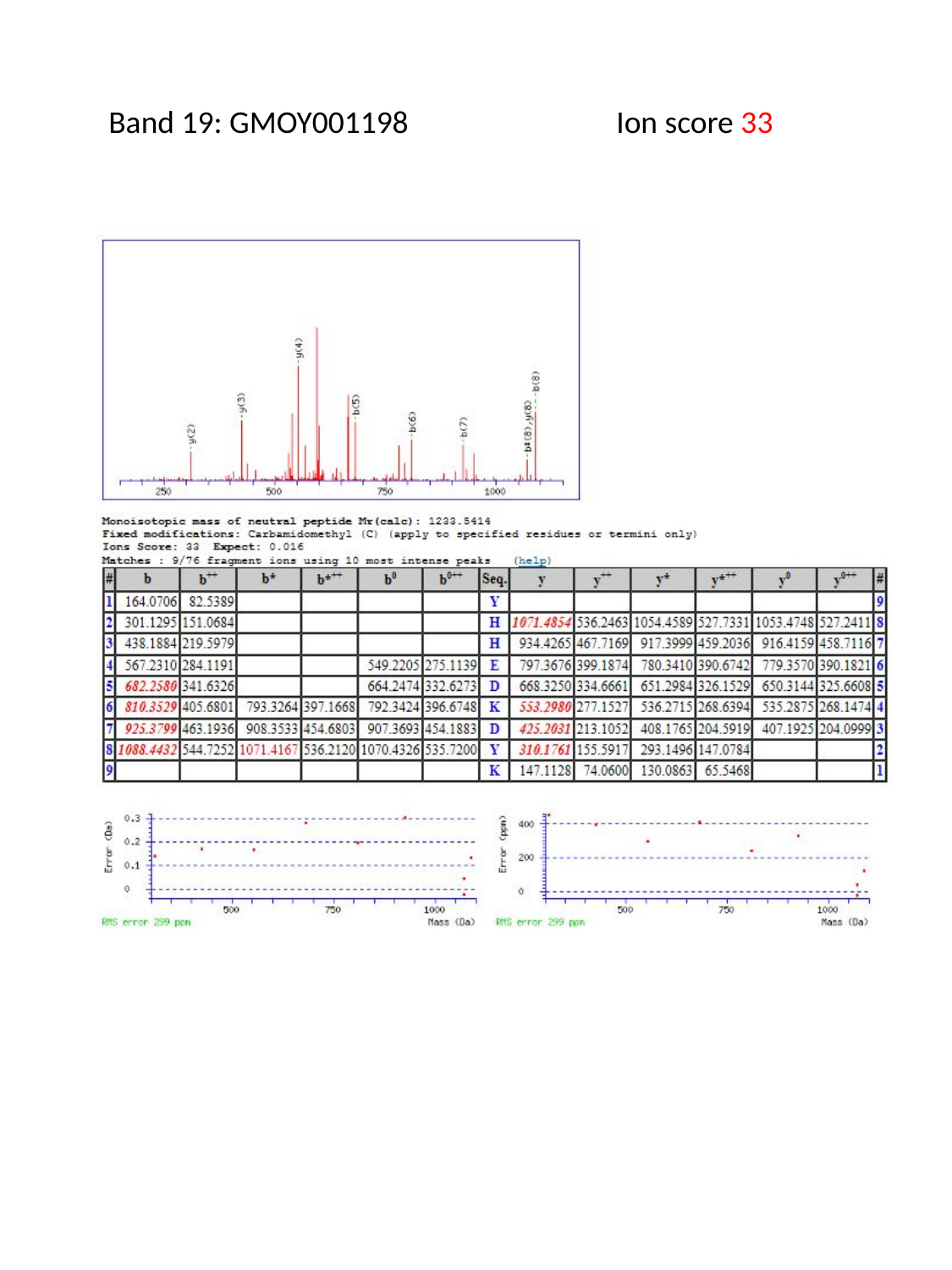

Band 19: GMOY001198 		Ion score 33

## Slide 67
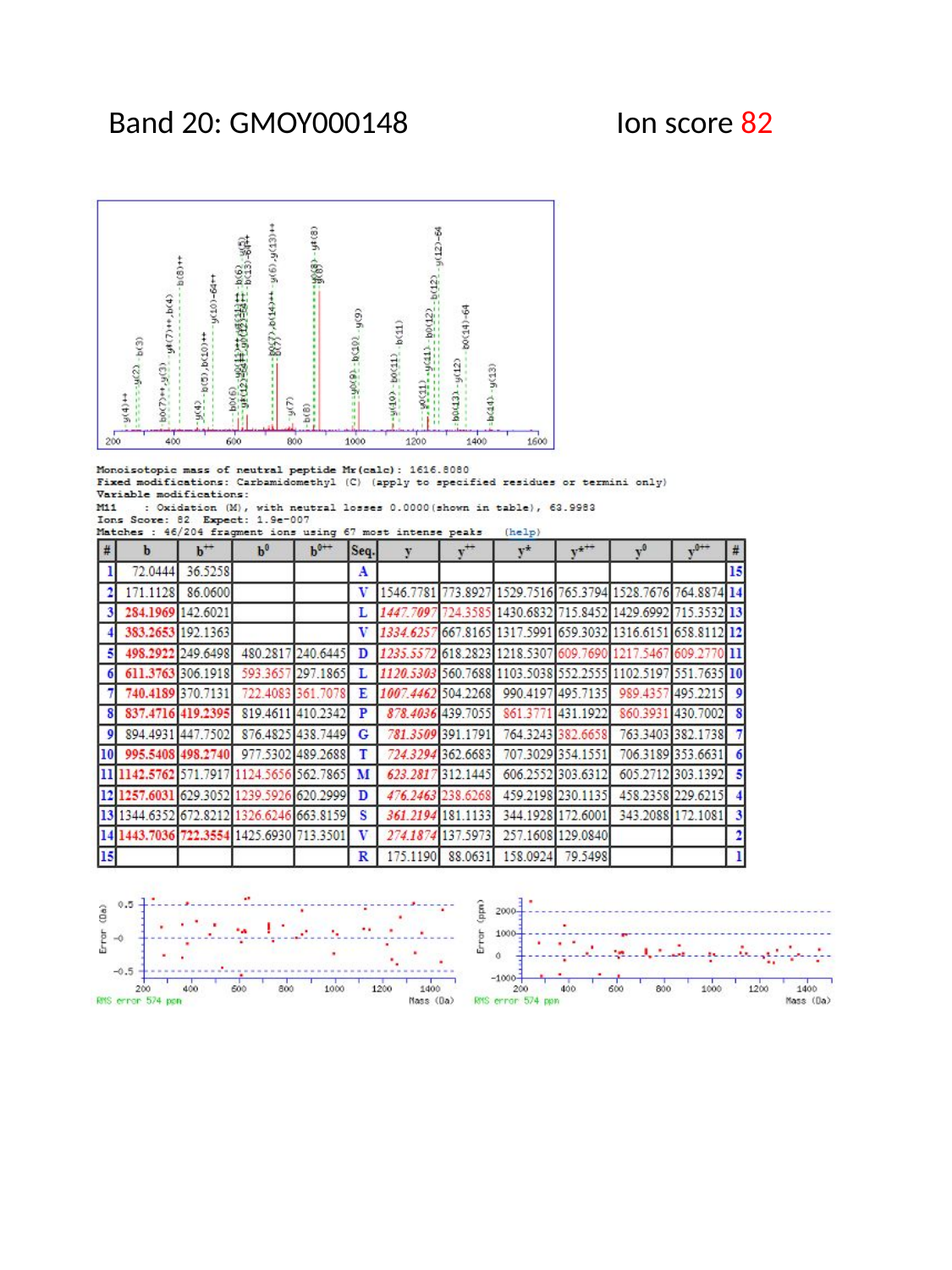

Band 20: GMOY000148 		Ion score 82

## Slide 68
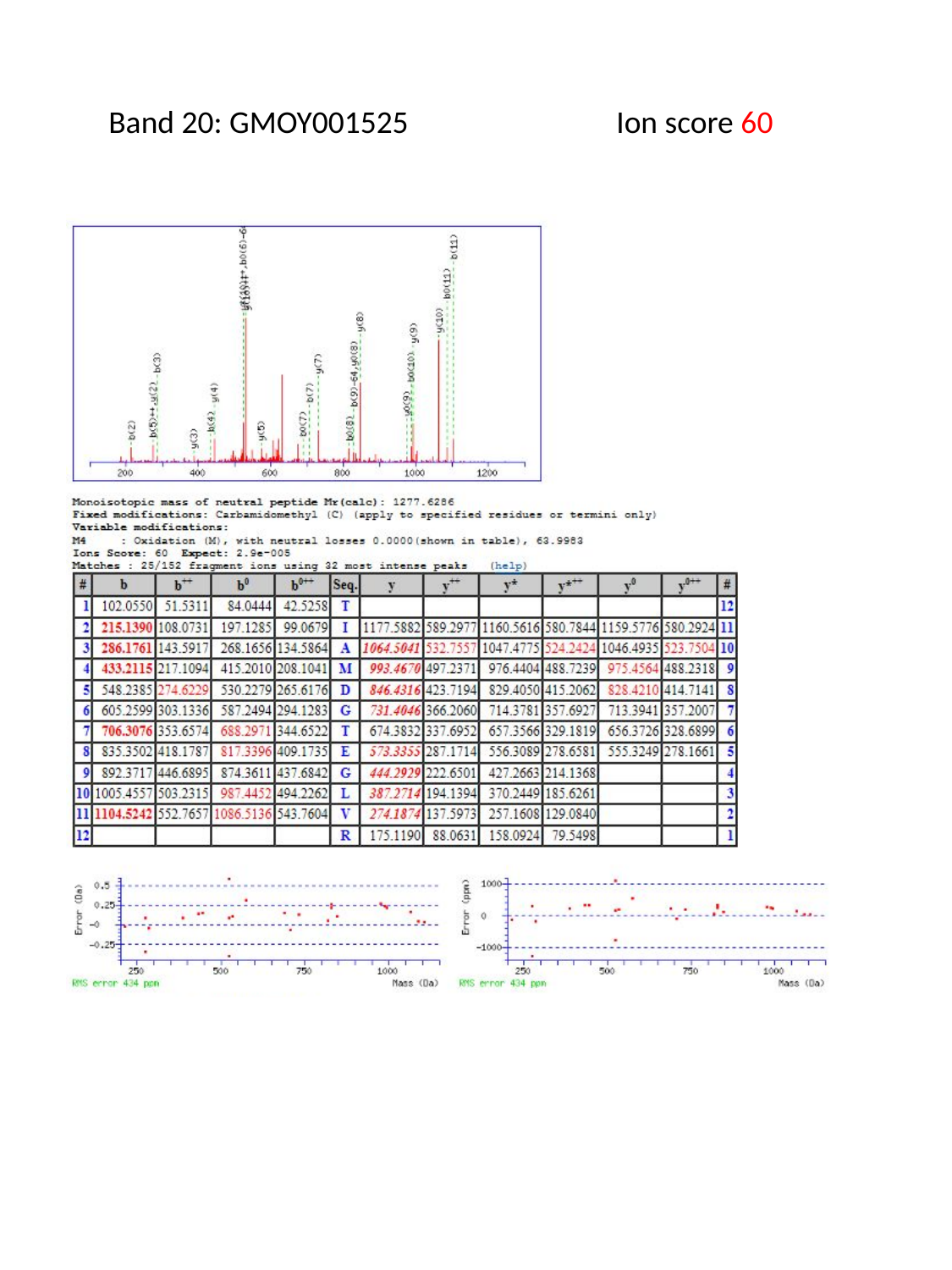

Band 20: GMOY001525 		Ion score 60

## Slide 69
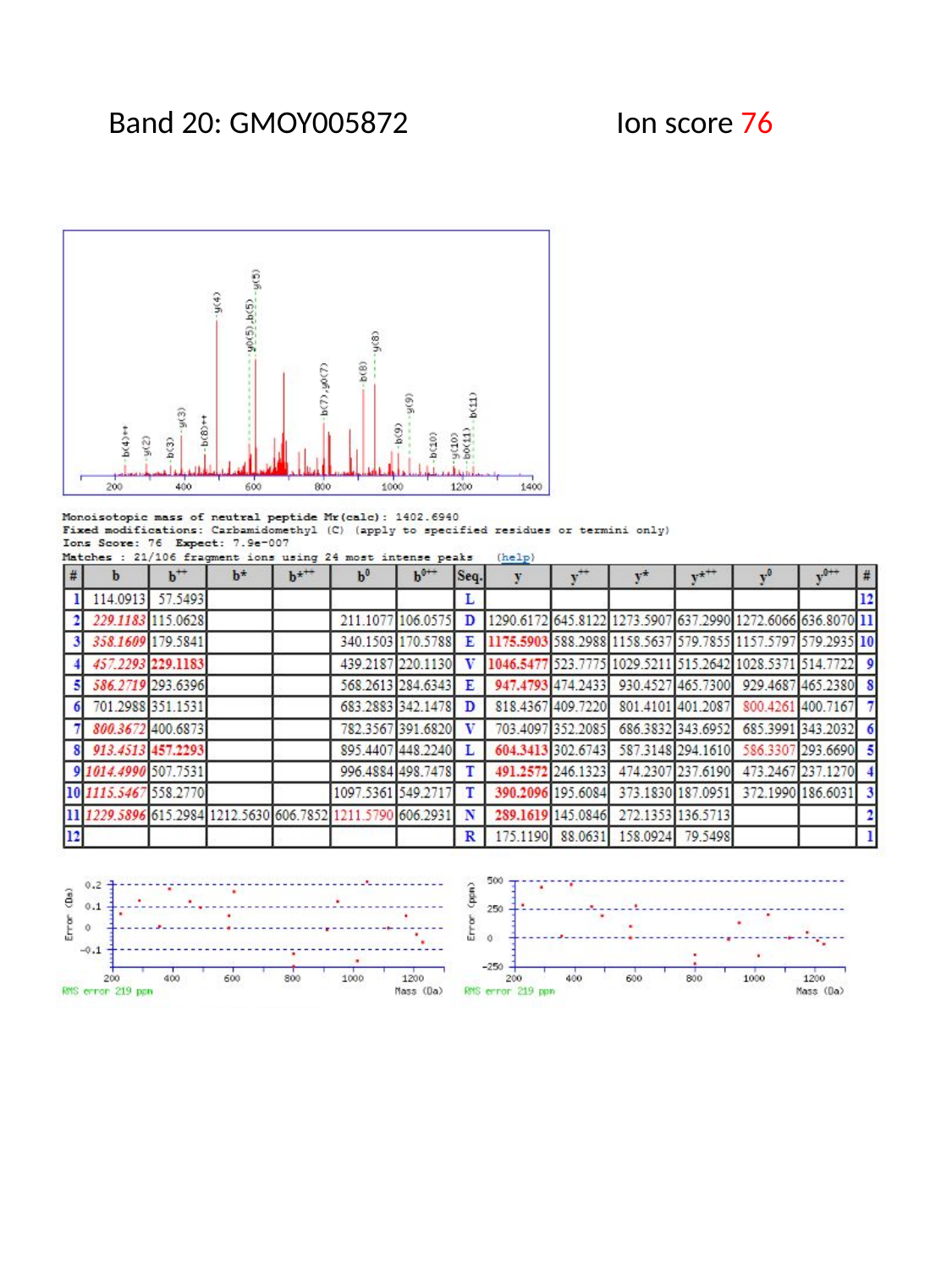

Band 20: GMOY005872 		Ion score 76

## Slide 70
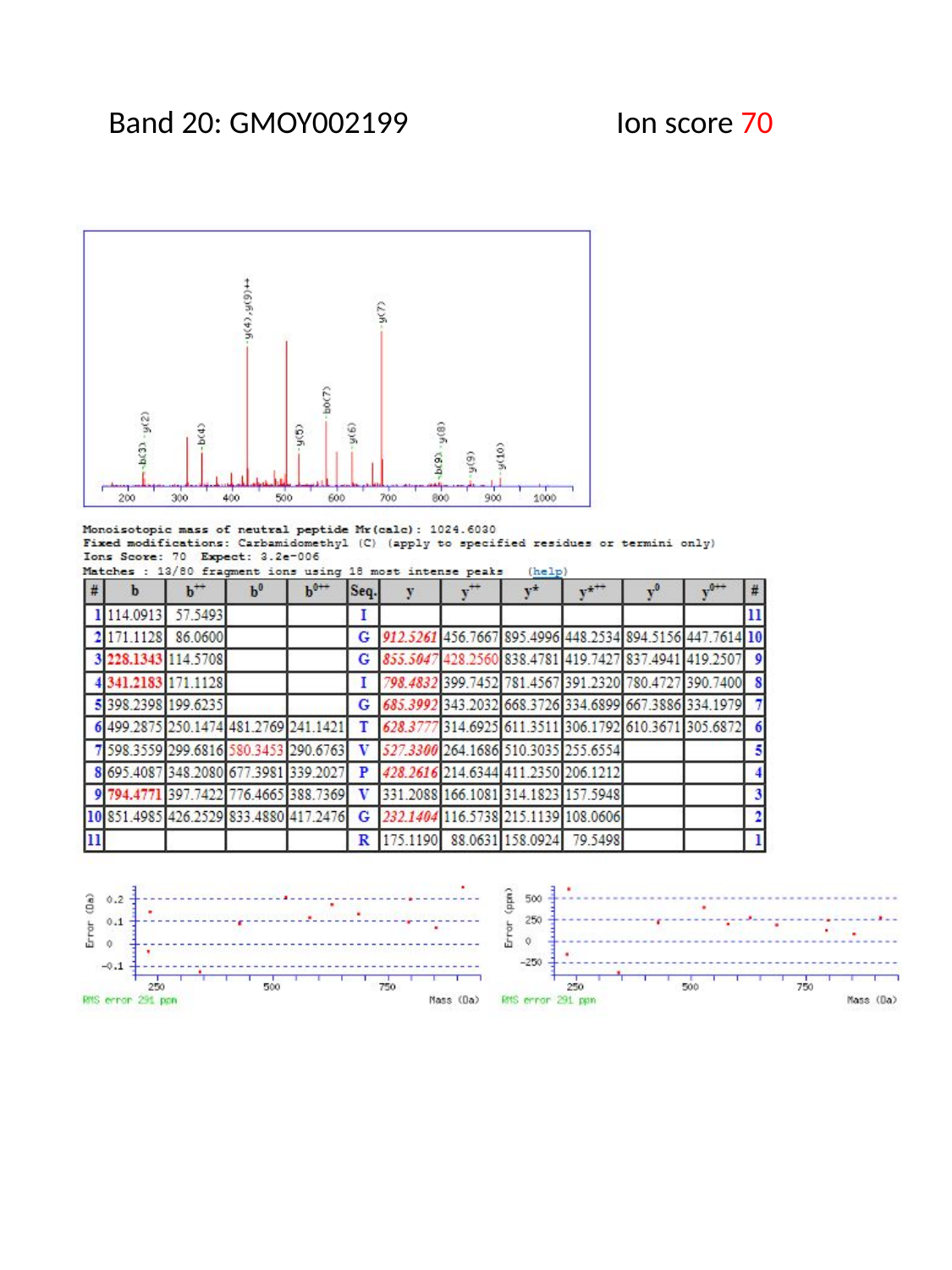

Band 20: GMOY002199 		Ion score 70

## Slide 71
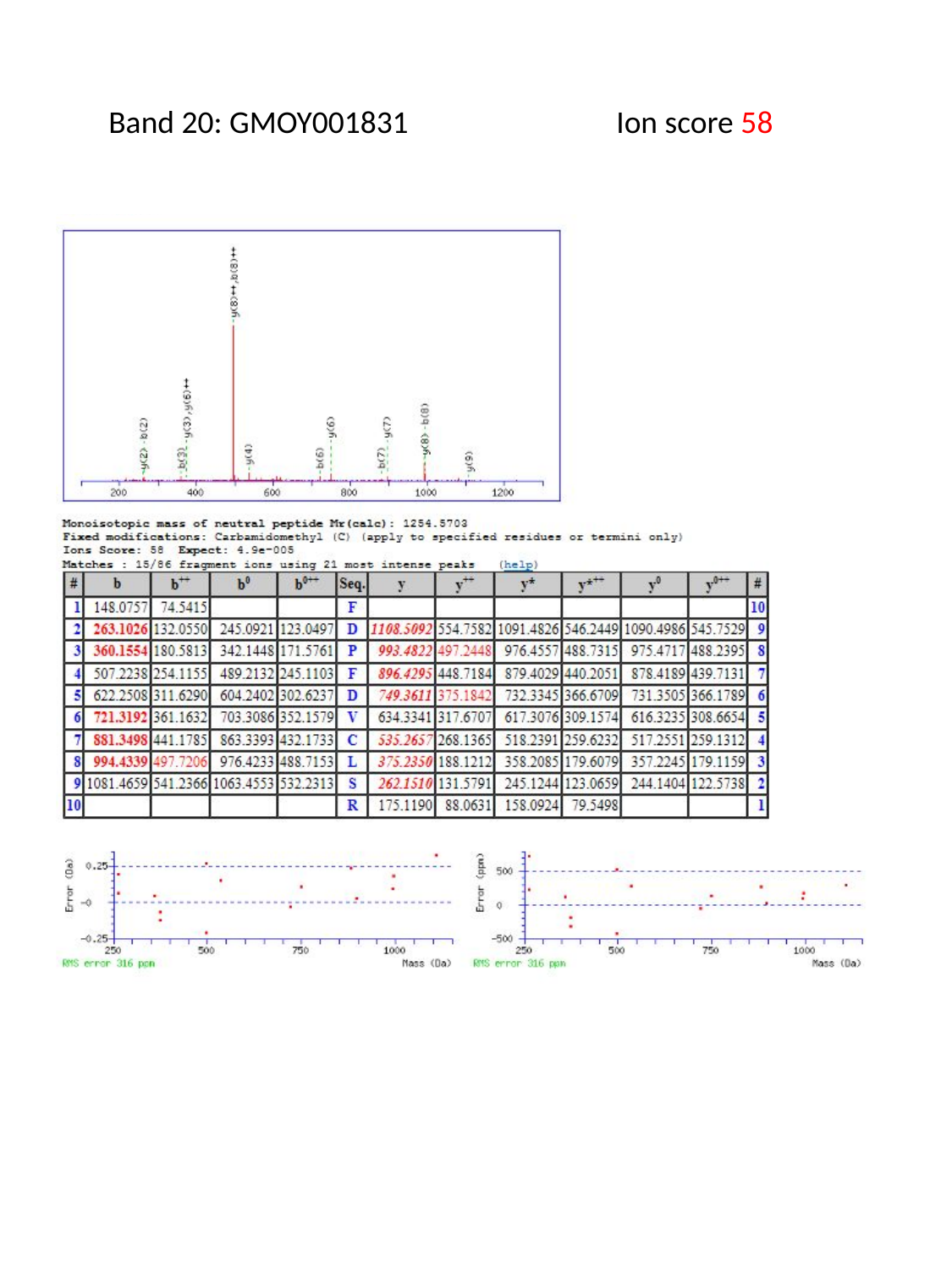

Band 20: GMOY001831 		Ion score 58

## Slide 72
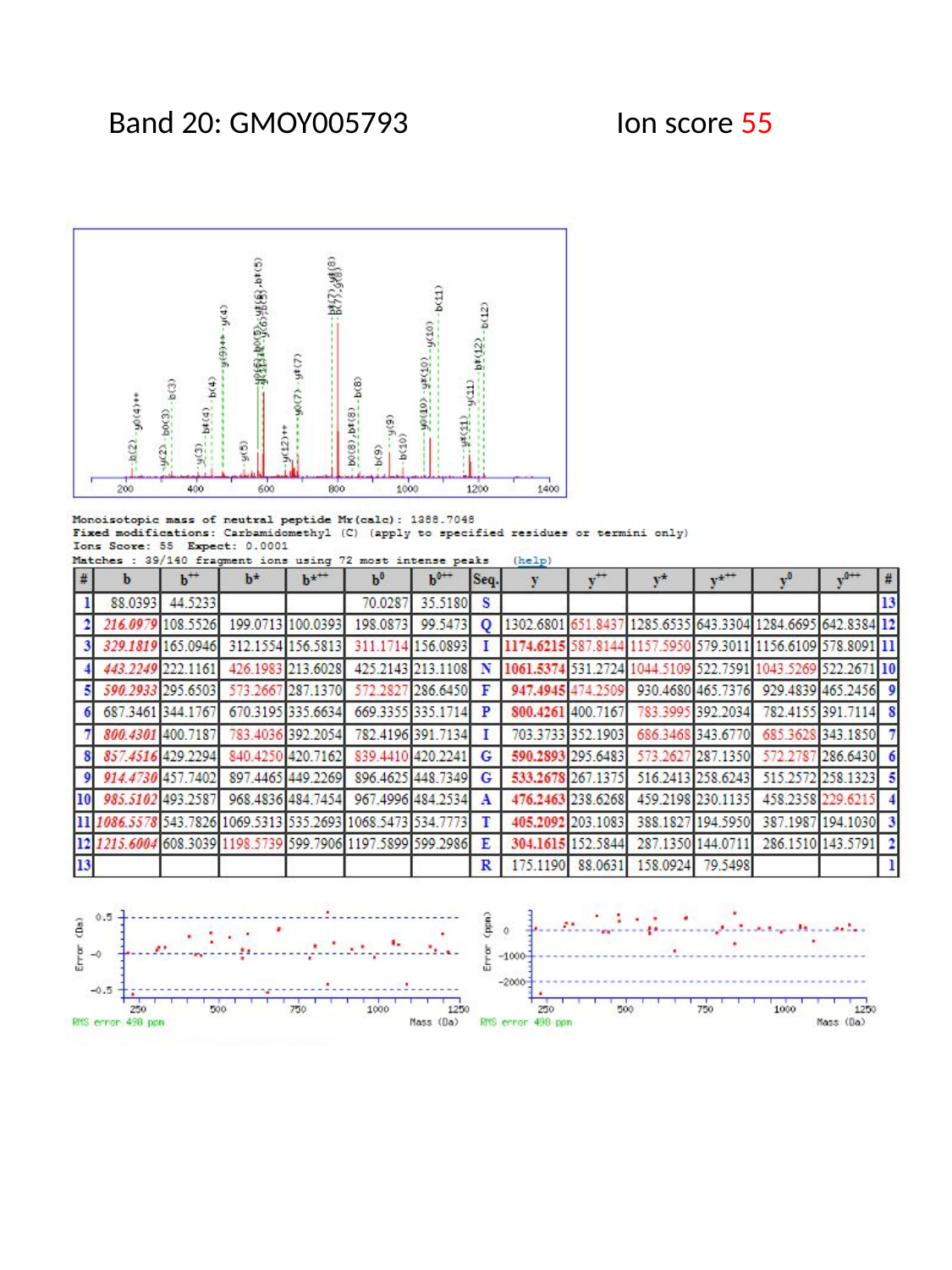

Band 20: GMOY005793 		Ion score 55

## Slide 73
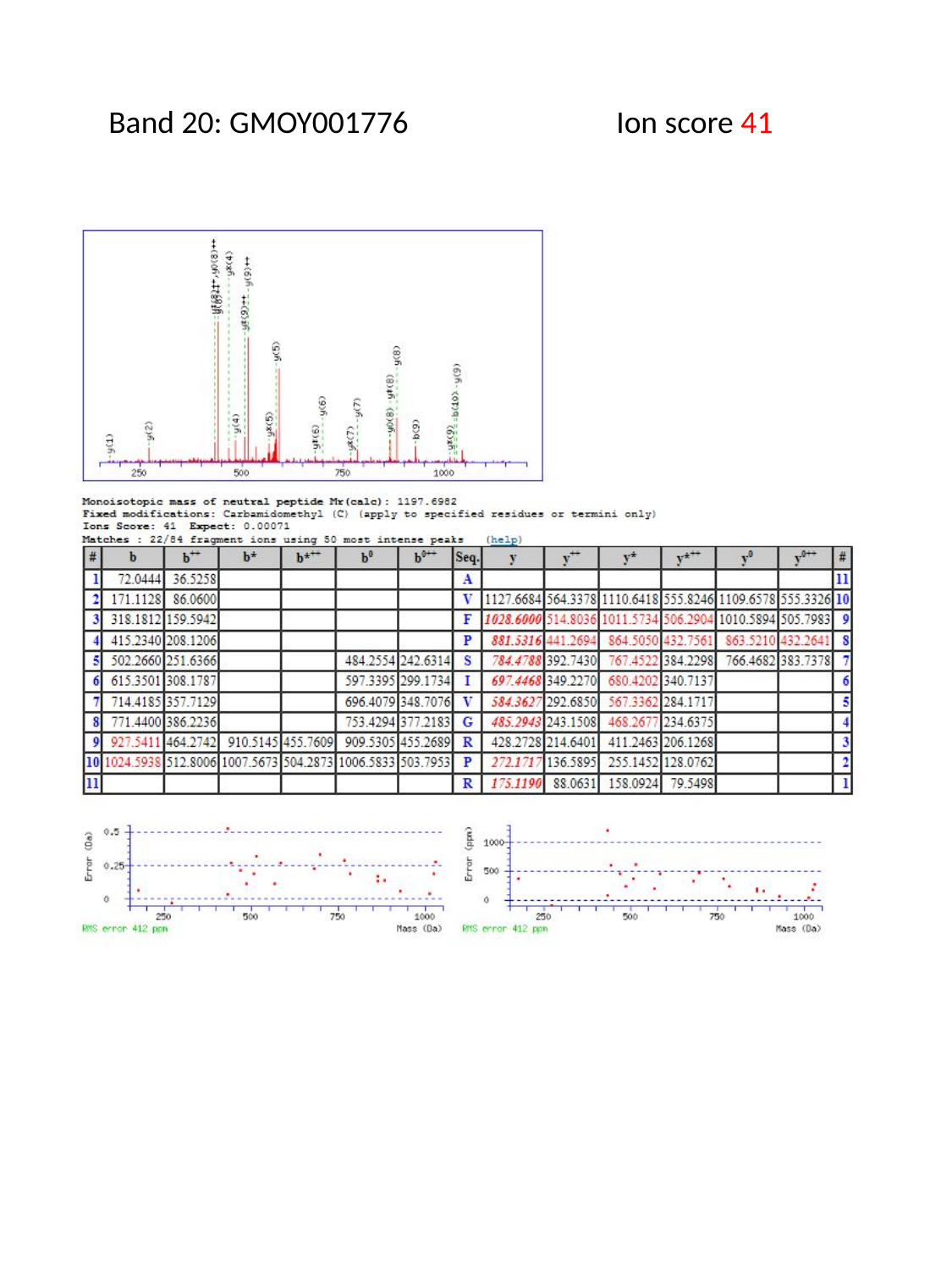

Band 20: GMOY001776 		Ion score 41

## Slide 74
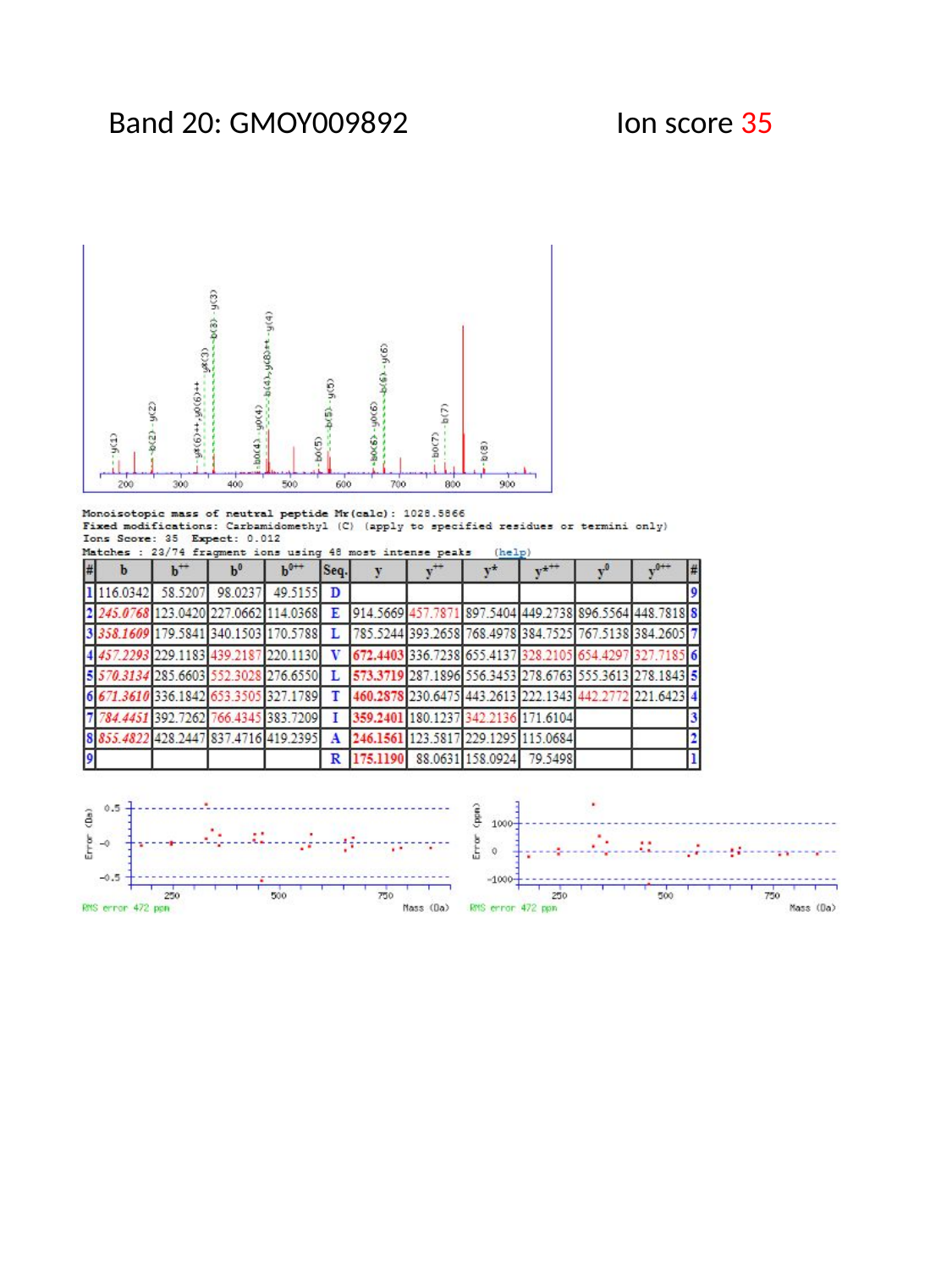

Band 20: GMOY009892 		Ion score 35

## Slide 75
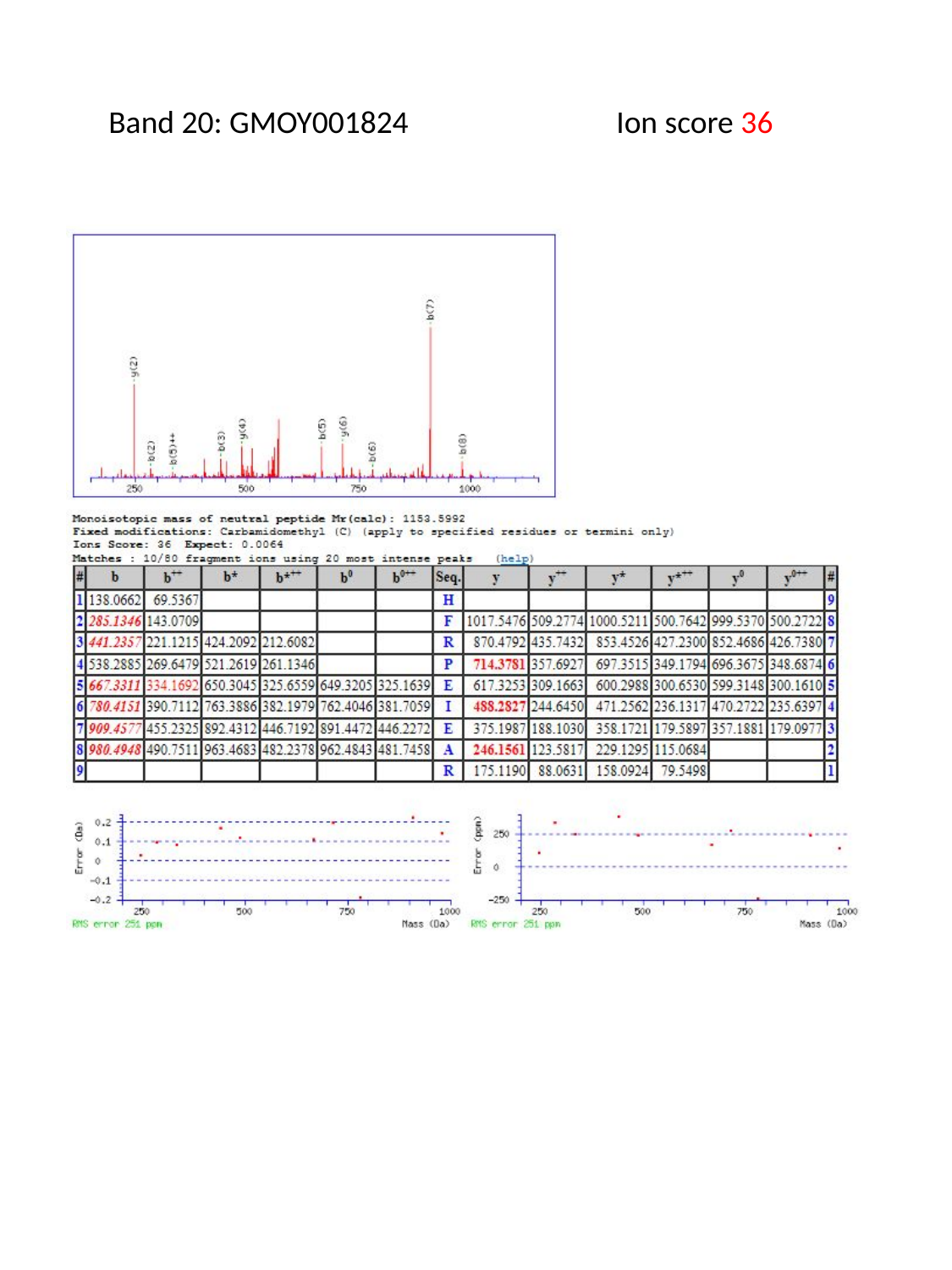

Band 20: GMOY001824 		Ion score 36

## Slide 76
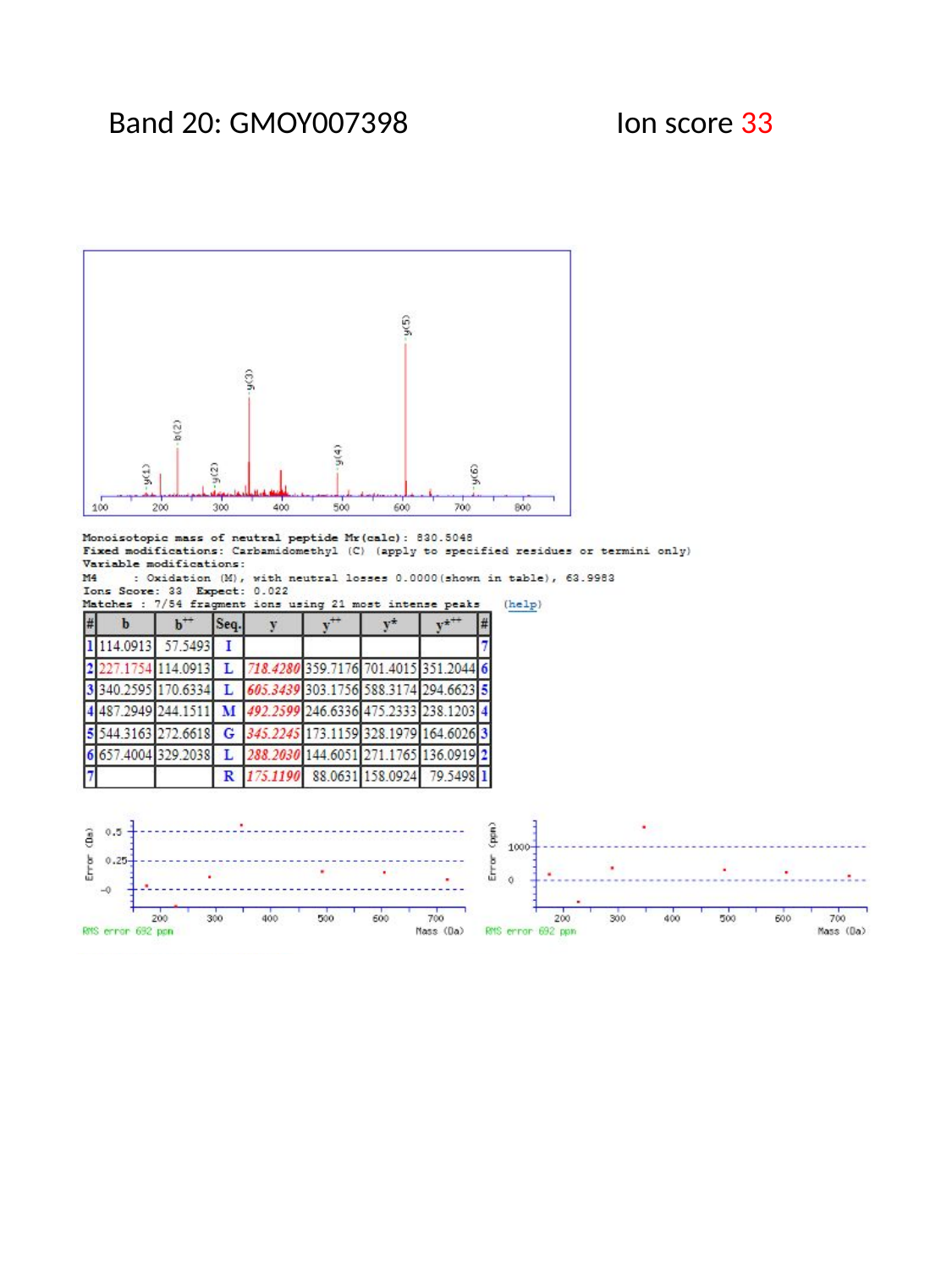

Band 20: GMOY007398 		Ion score 33

## Slide 77
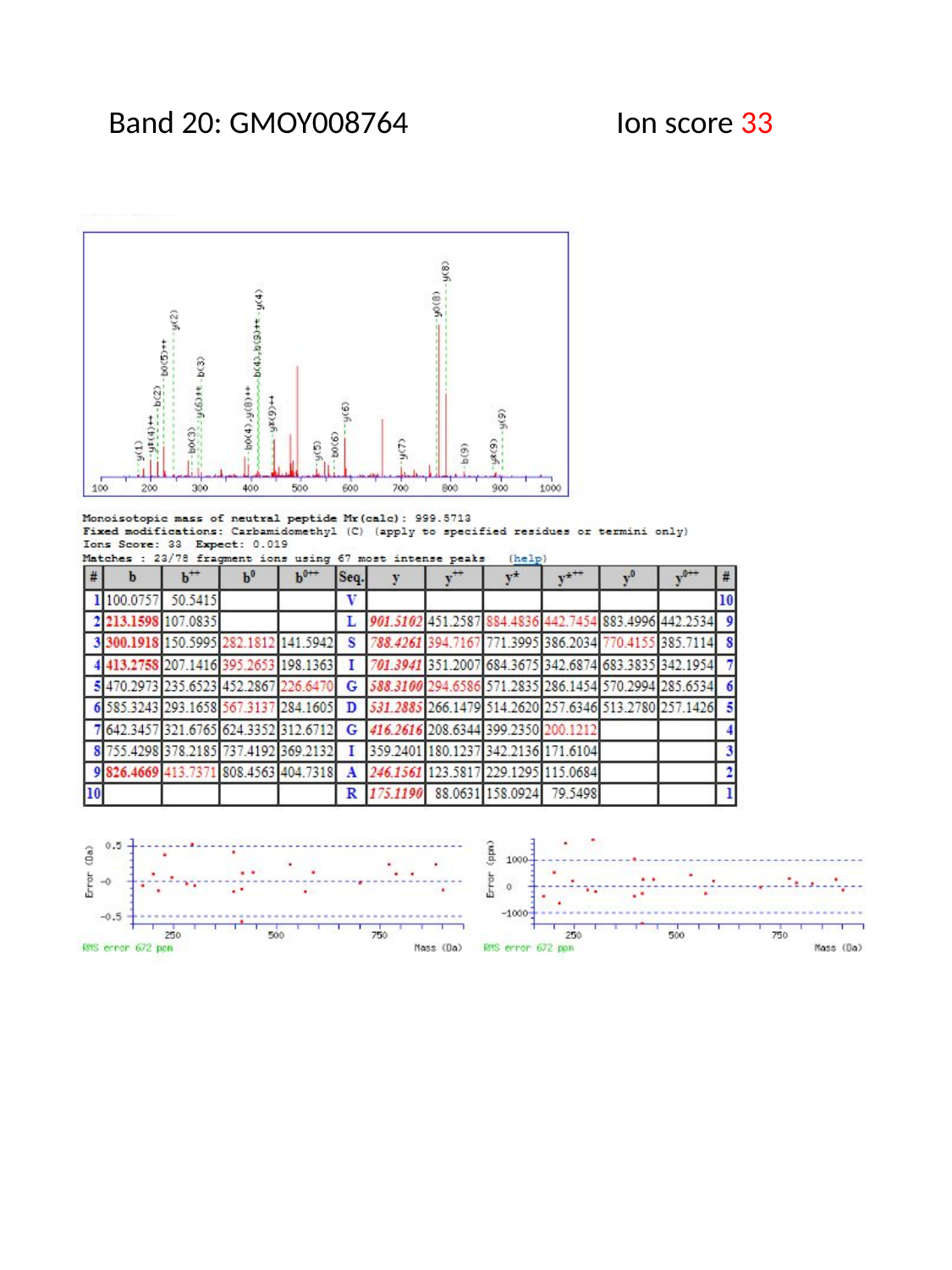

Band 20: GMOY008764 		Ion score 33

## Slide 78
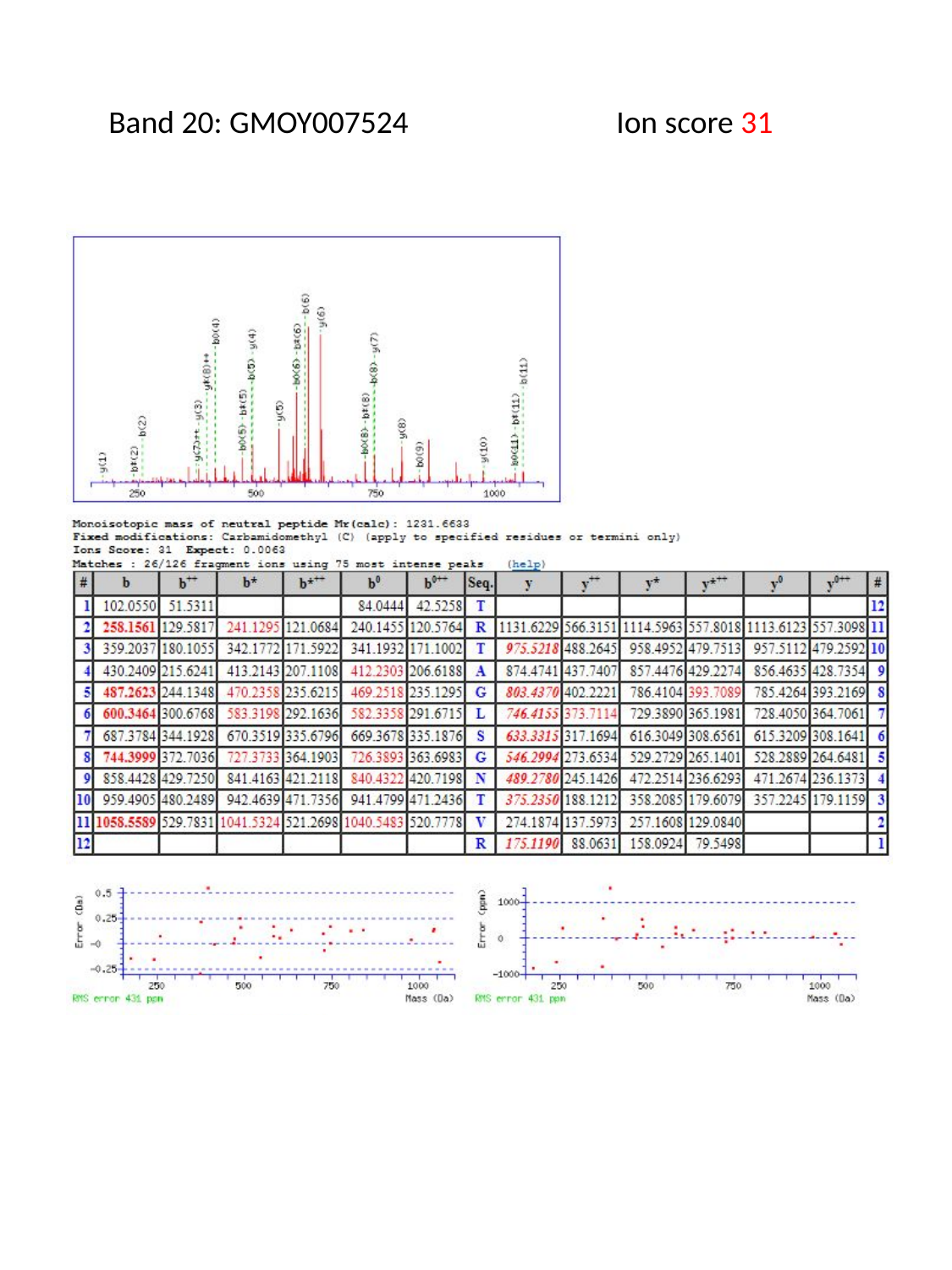

Band 20: GMOY007524 		Ion score 31

## Slide 79
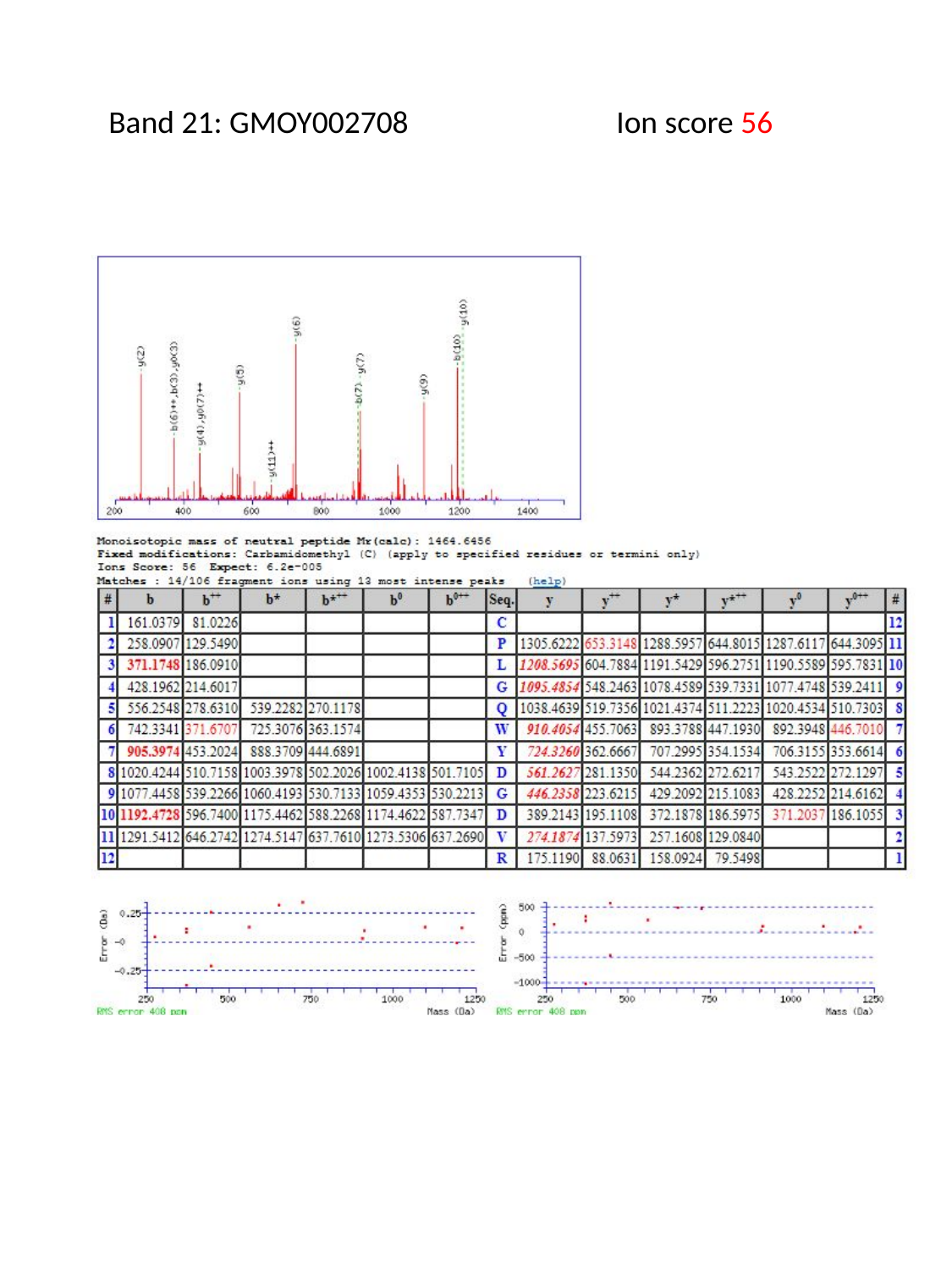

Band 21: GMOY002708 		Ion score 56

## Slide 80
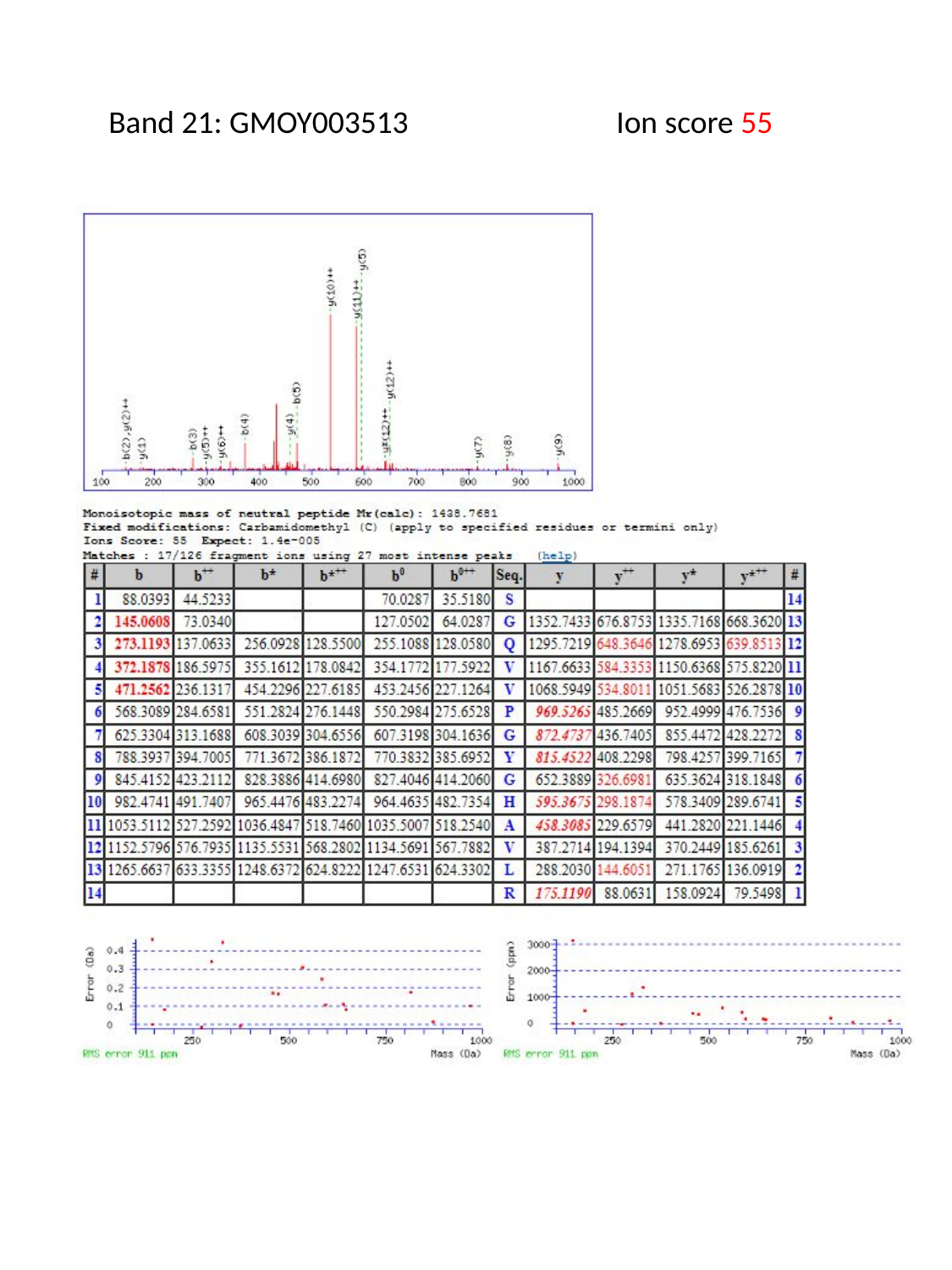

Band 21: GMOY003513 		Ion score 55

## Slide 81
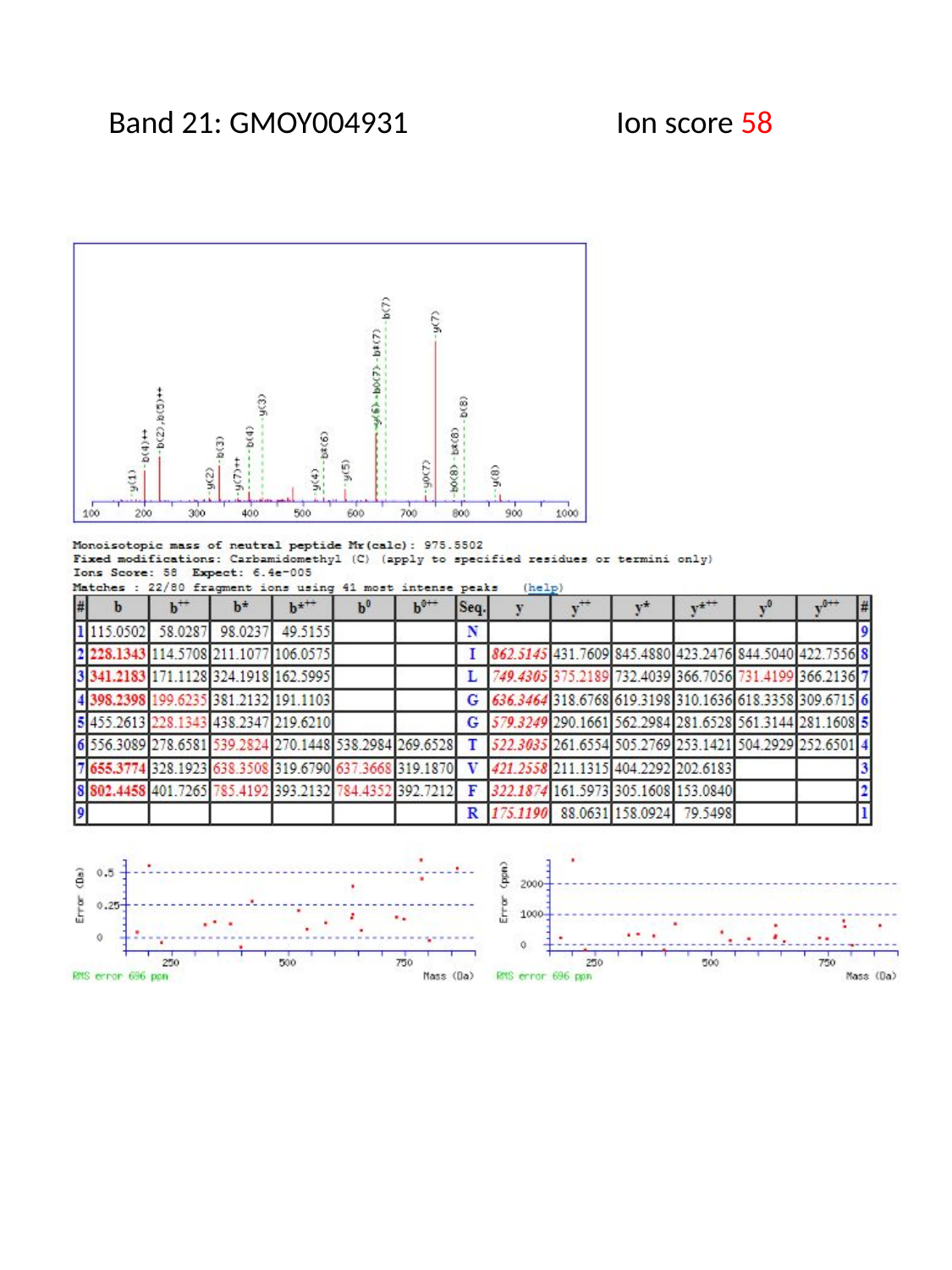

Band 21: GMOY004931 		Ion score 58

## Slide 82
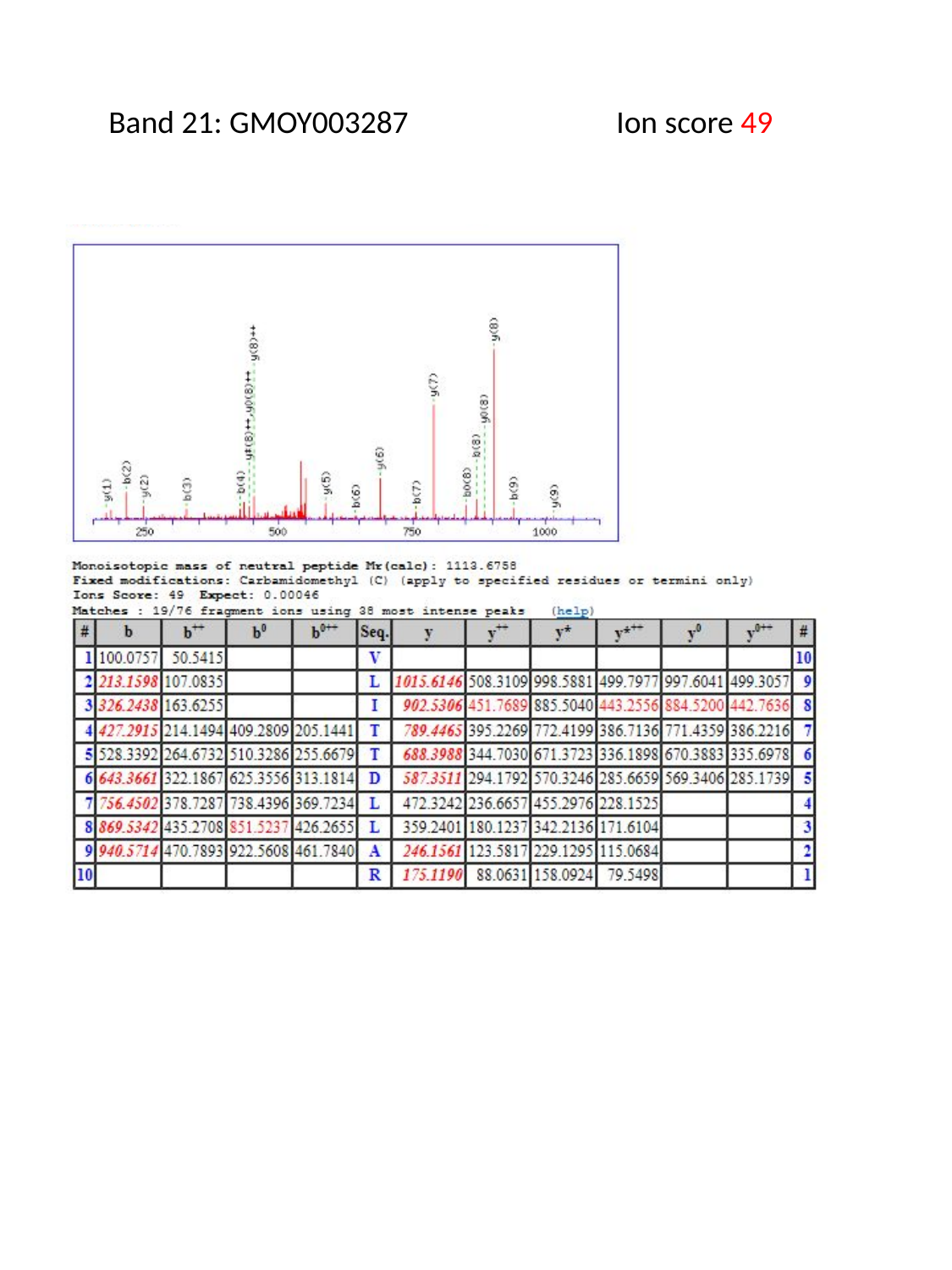

Band 21: GMOY003287 		Ion score 49

## Slide 83
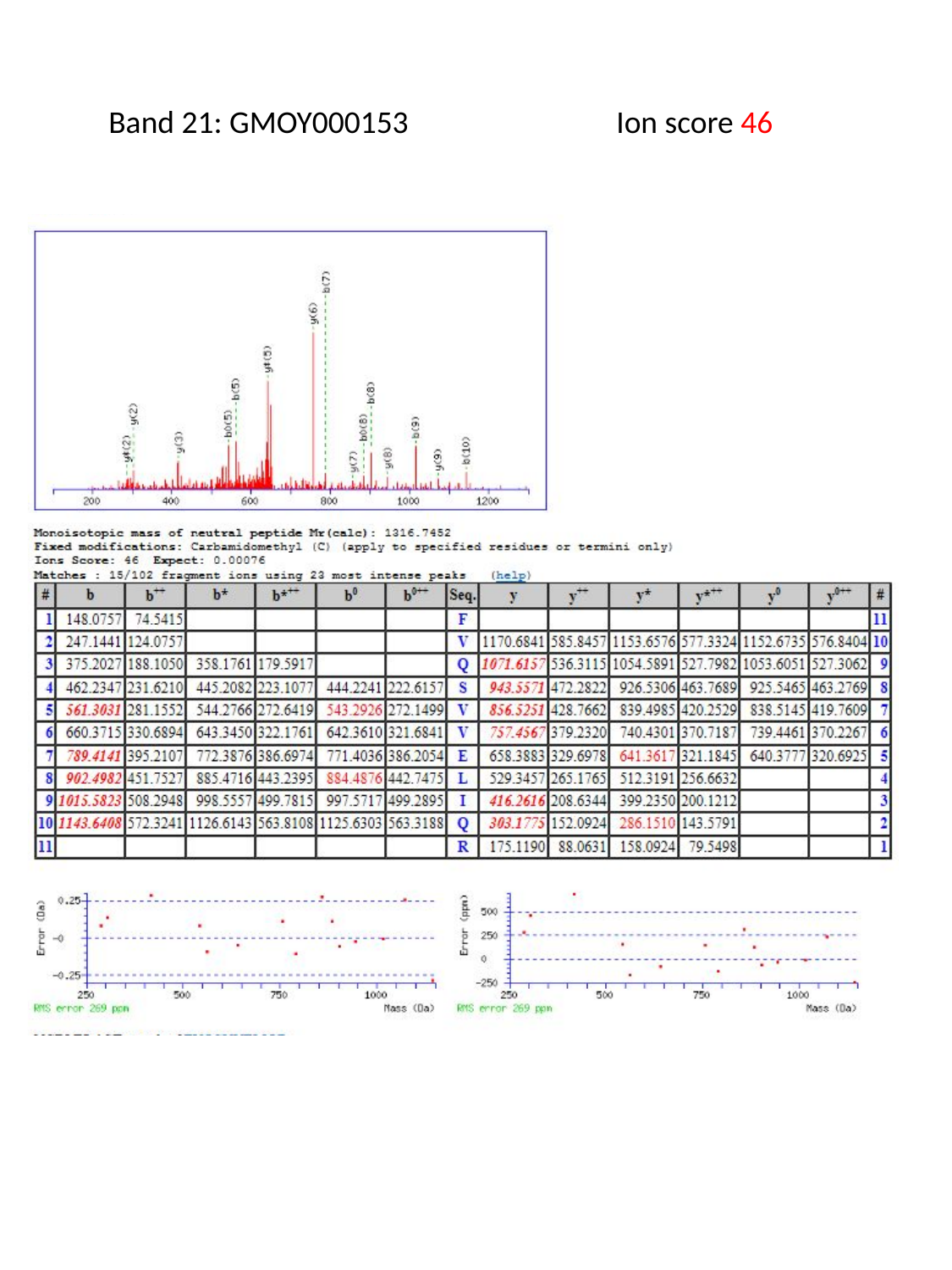

Band 21: GMOY000153 		Ion score 46

## Slide 84
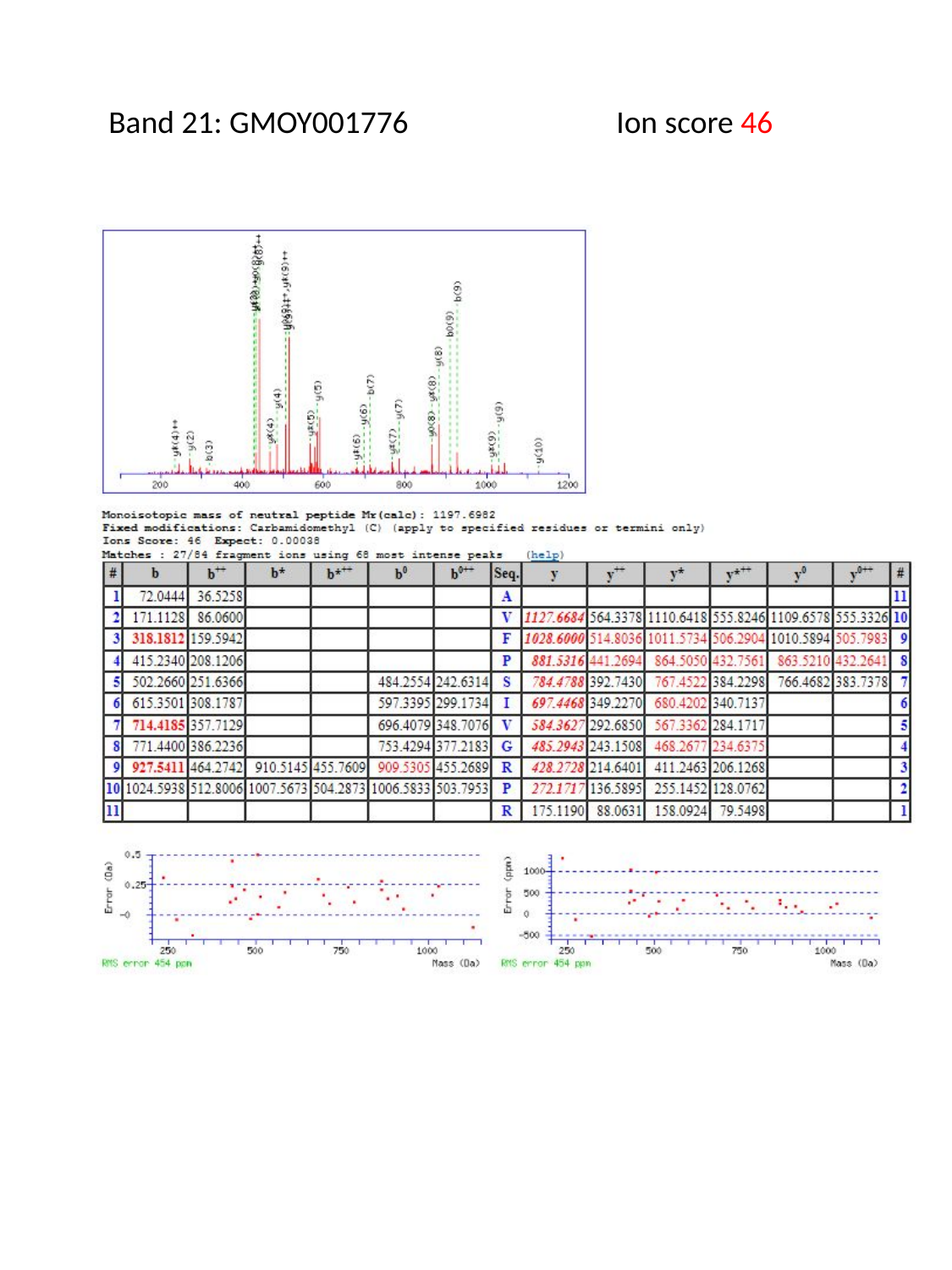

Band 21: GMOY001776 		Ion score 46

## Slide 85
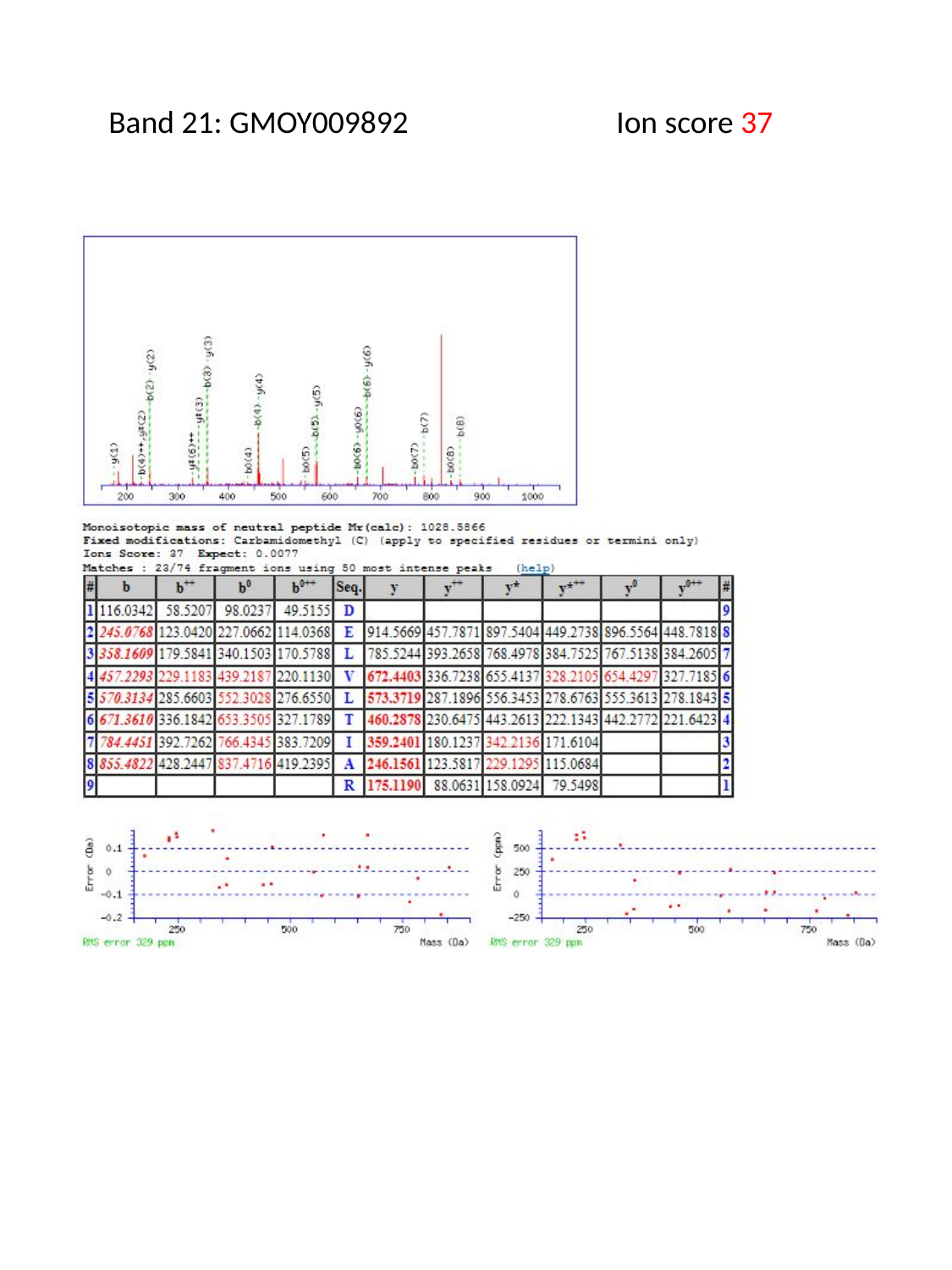

Band 21: GMOY009892 		Ion score 37

## Slide 86
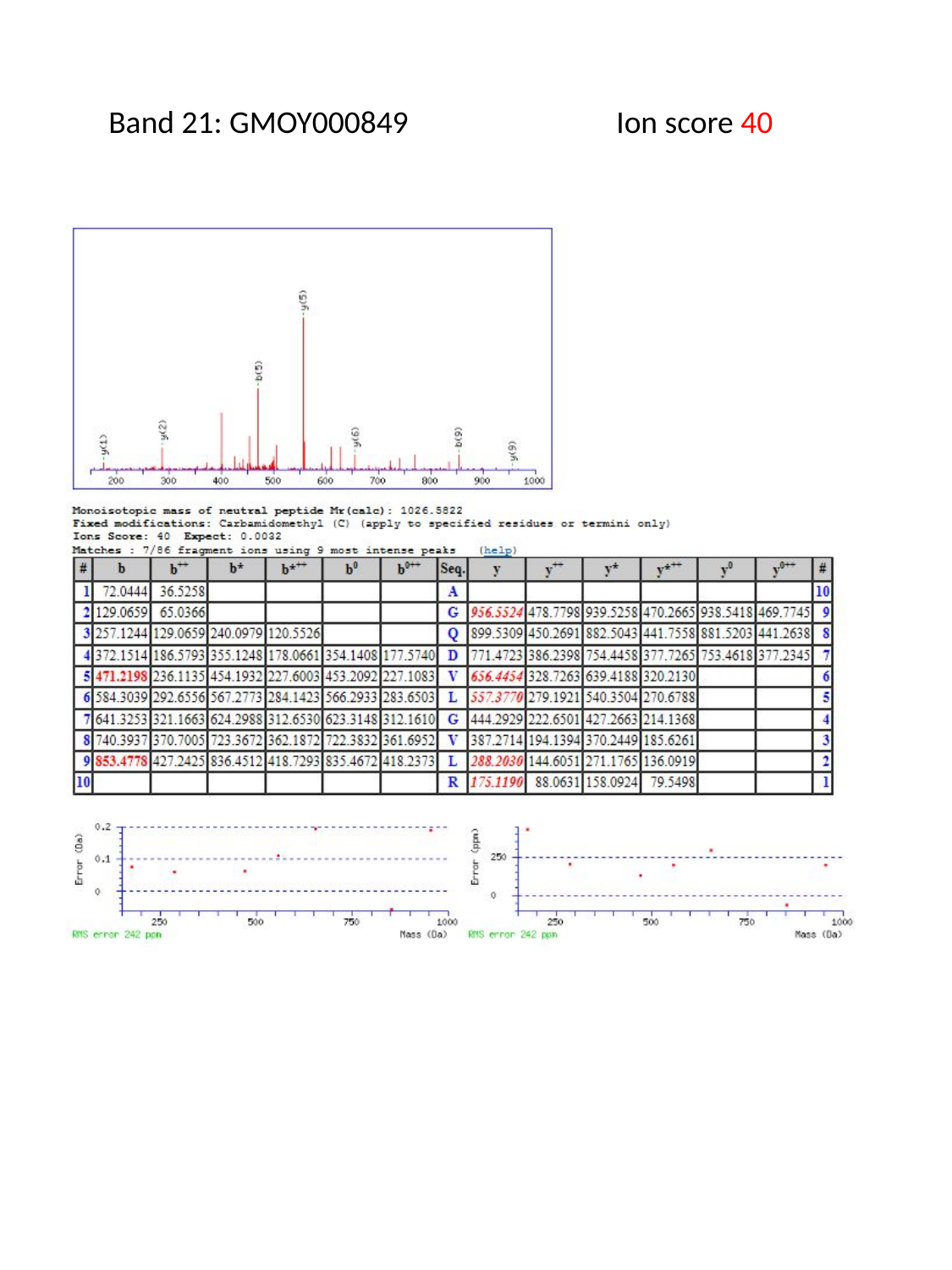

Band 21: GMOY000849 		Ion score 40

## Slide 87
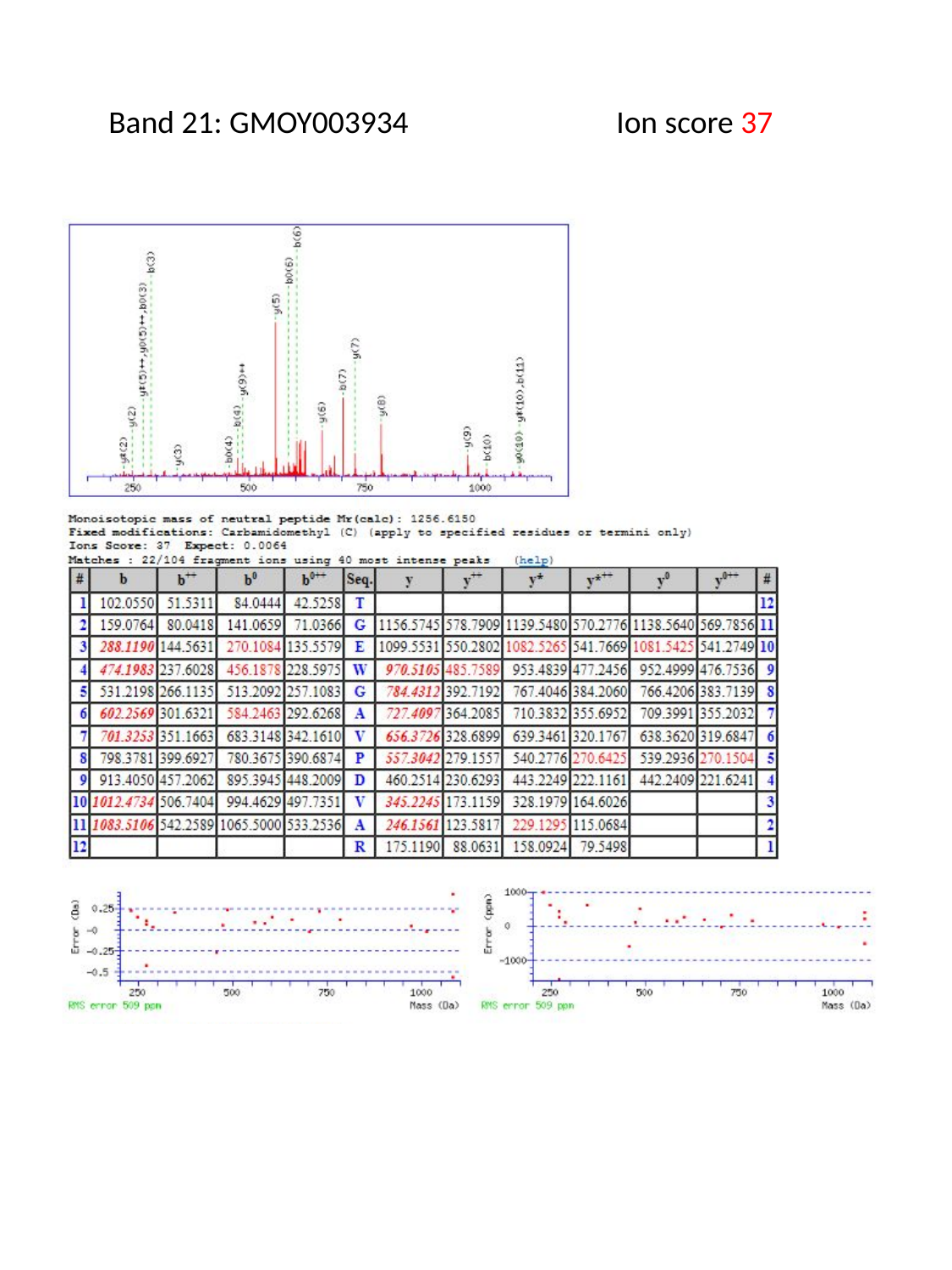

Band 21: GMOY003934 		Ion score 37

## Slide 88
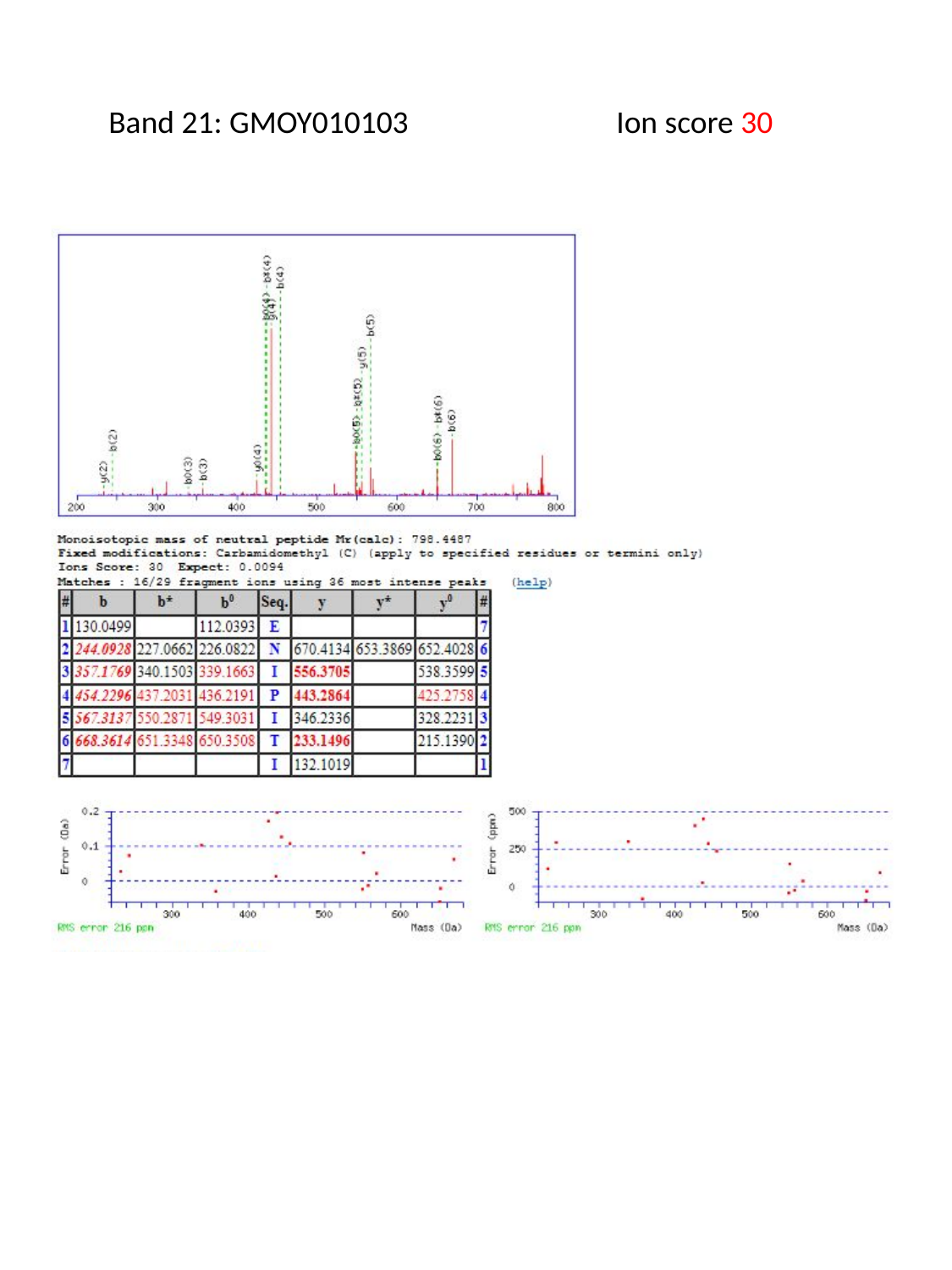

Band 21: GMOY010103 		Ion score 30

## Slide 89
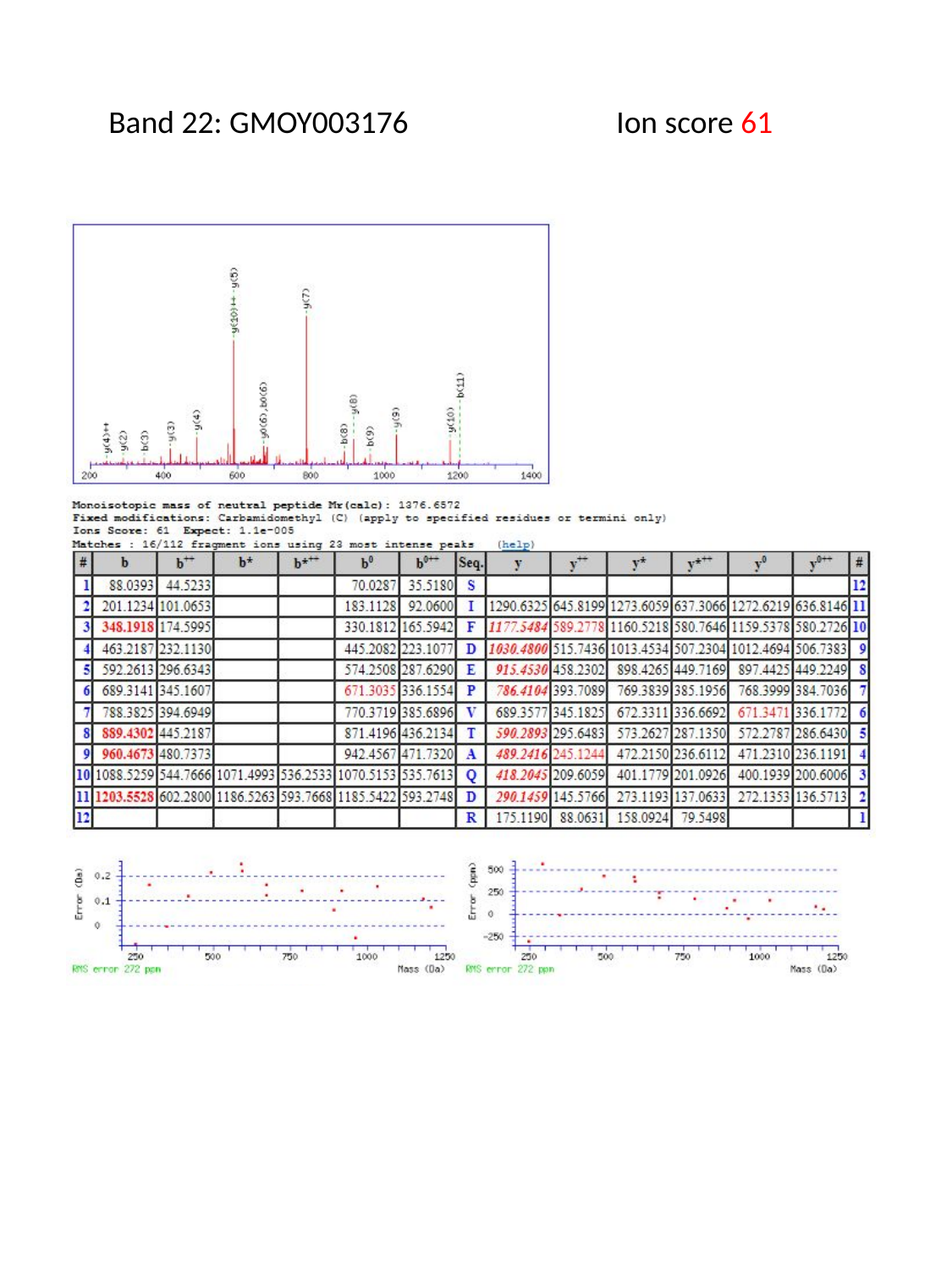

Band 22: GMOY003176 		Ion score 61

## Slide 90
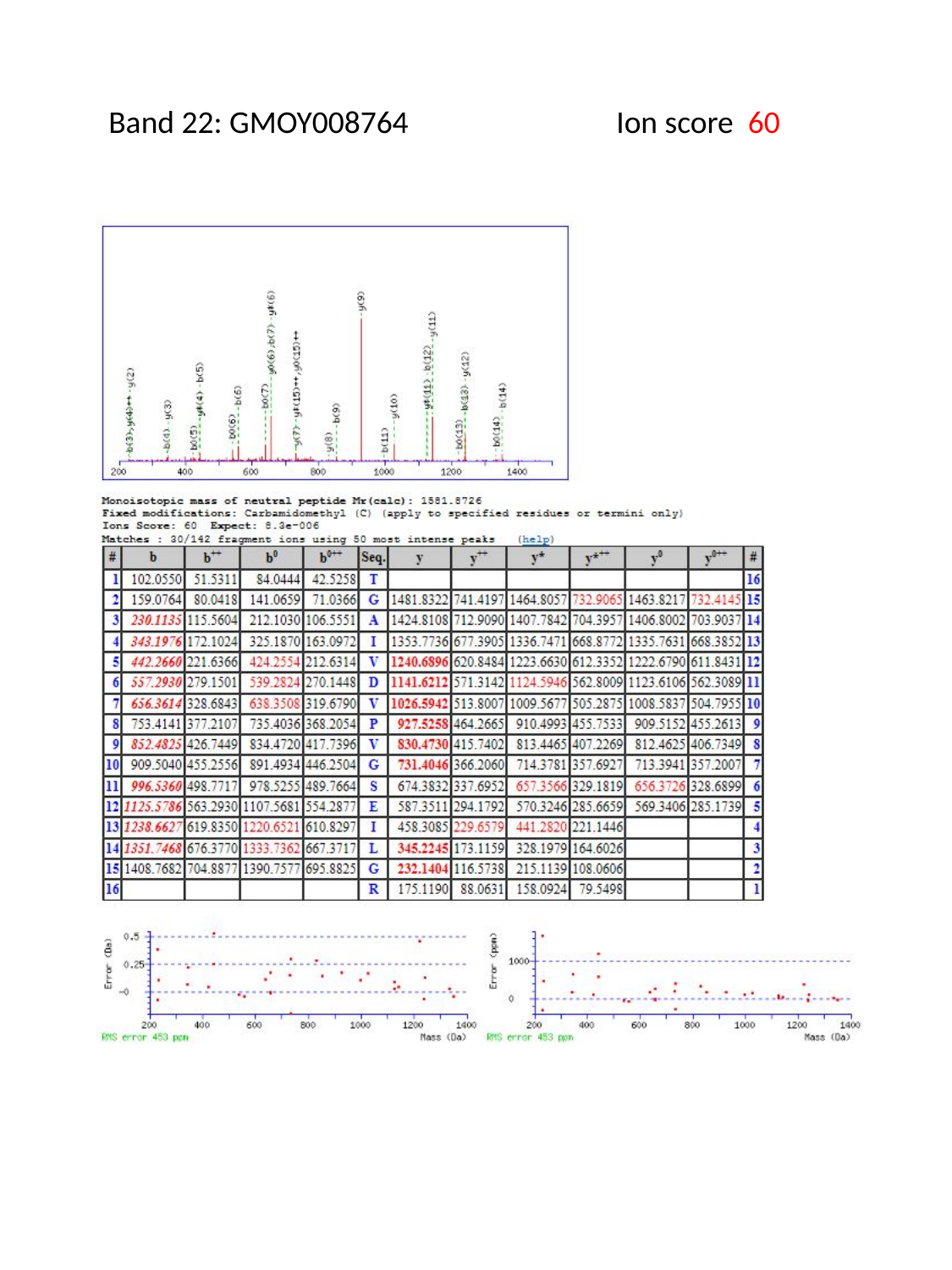

Band 22: GMOY008764 		Ion score 60

## Slide 91
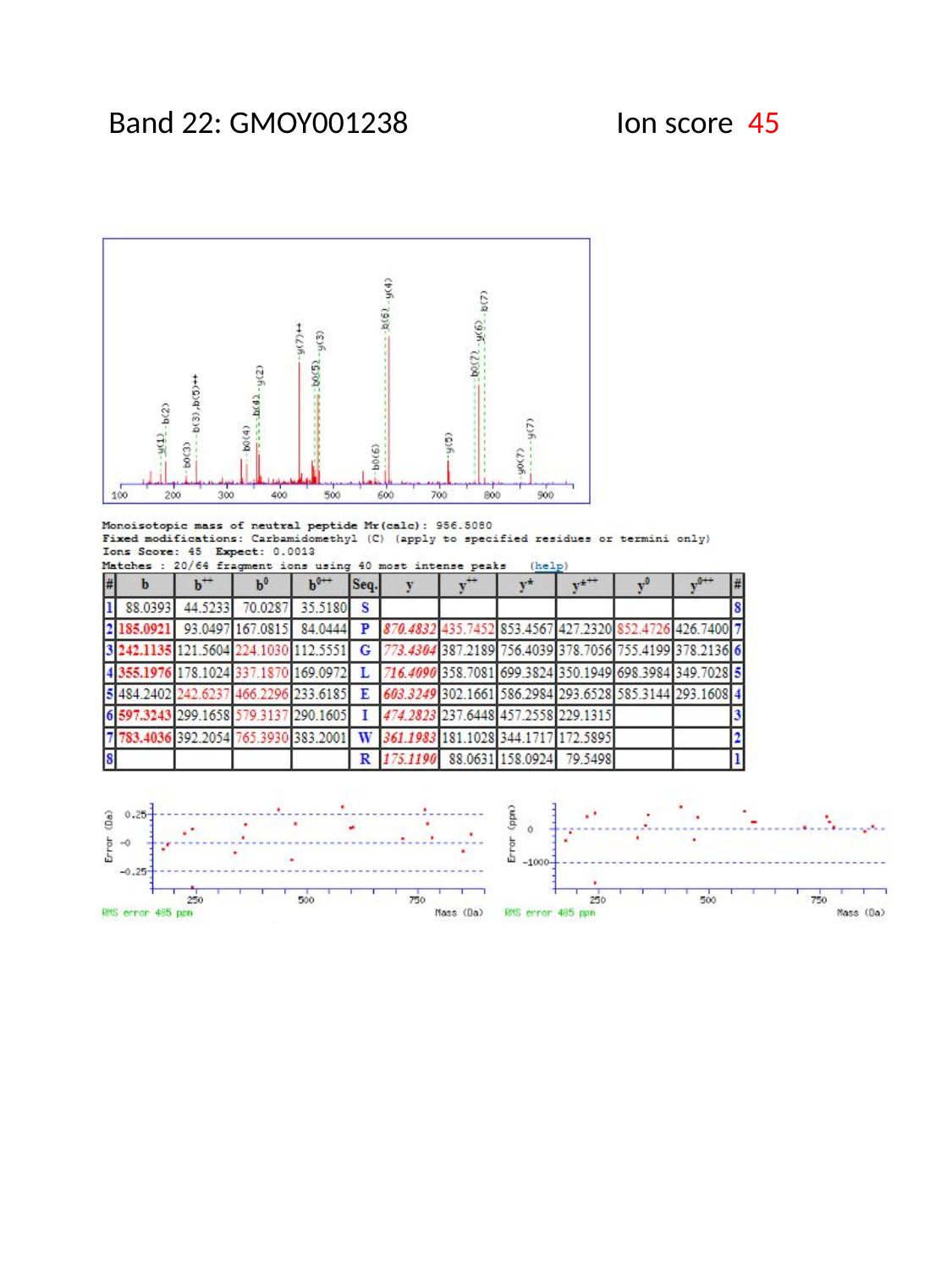

Band 22: GMOY001238 		Ion score 45

## Slide 92
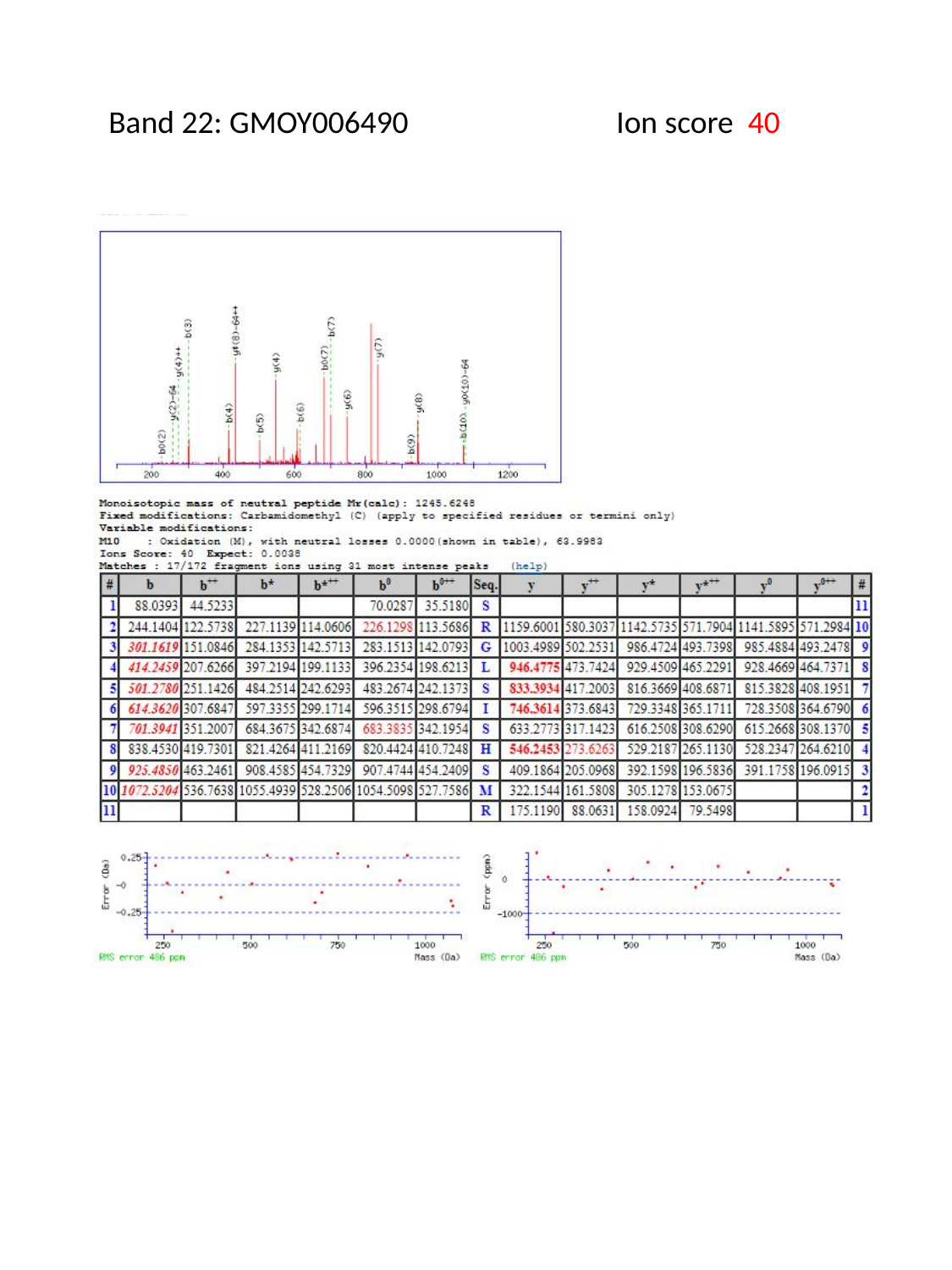

Band 22: GMOY006490 		Ion score 40

## Slide 93
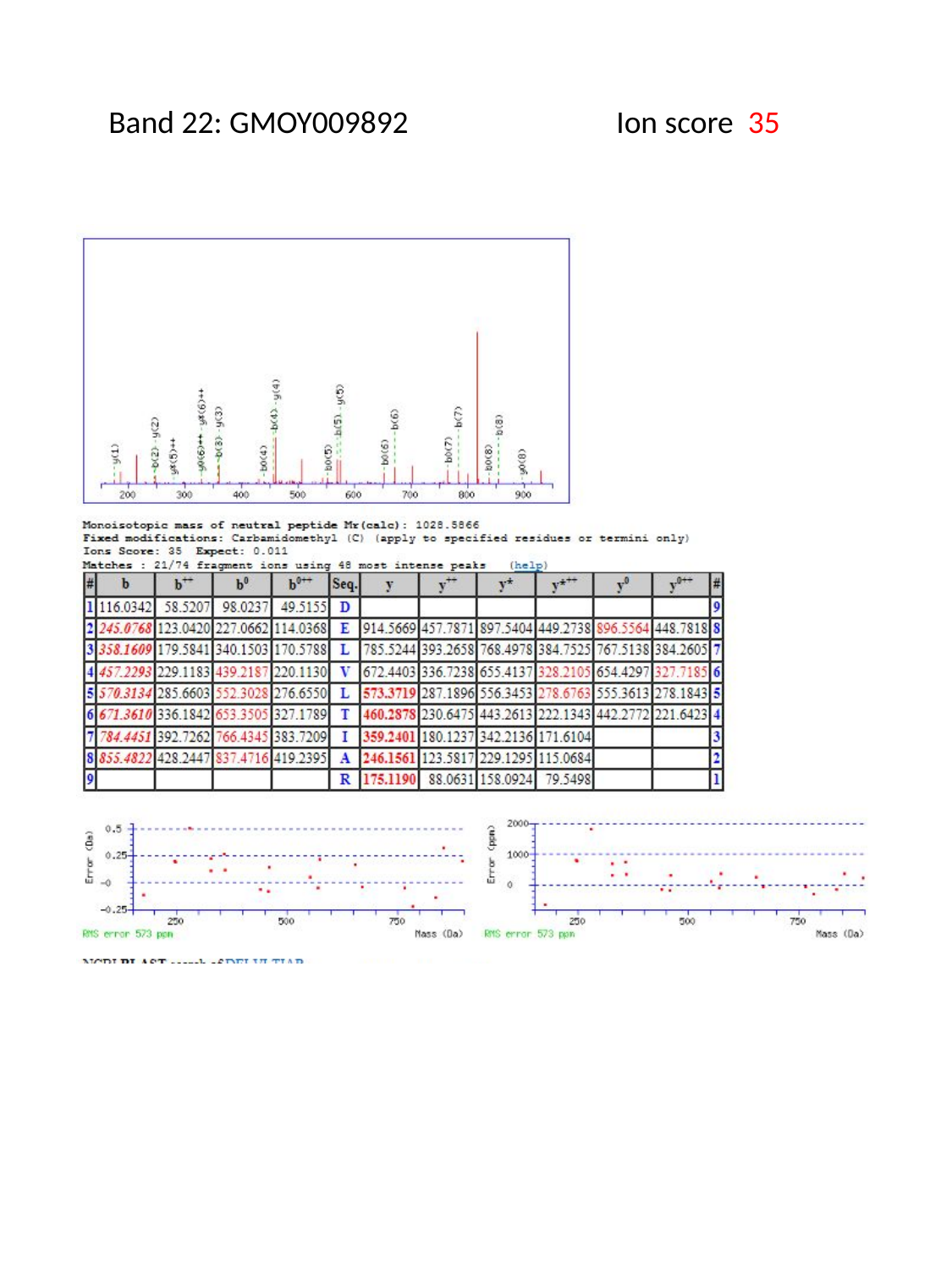

Band 22: GMOY009892 		Ion score 35

## Slide 94
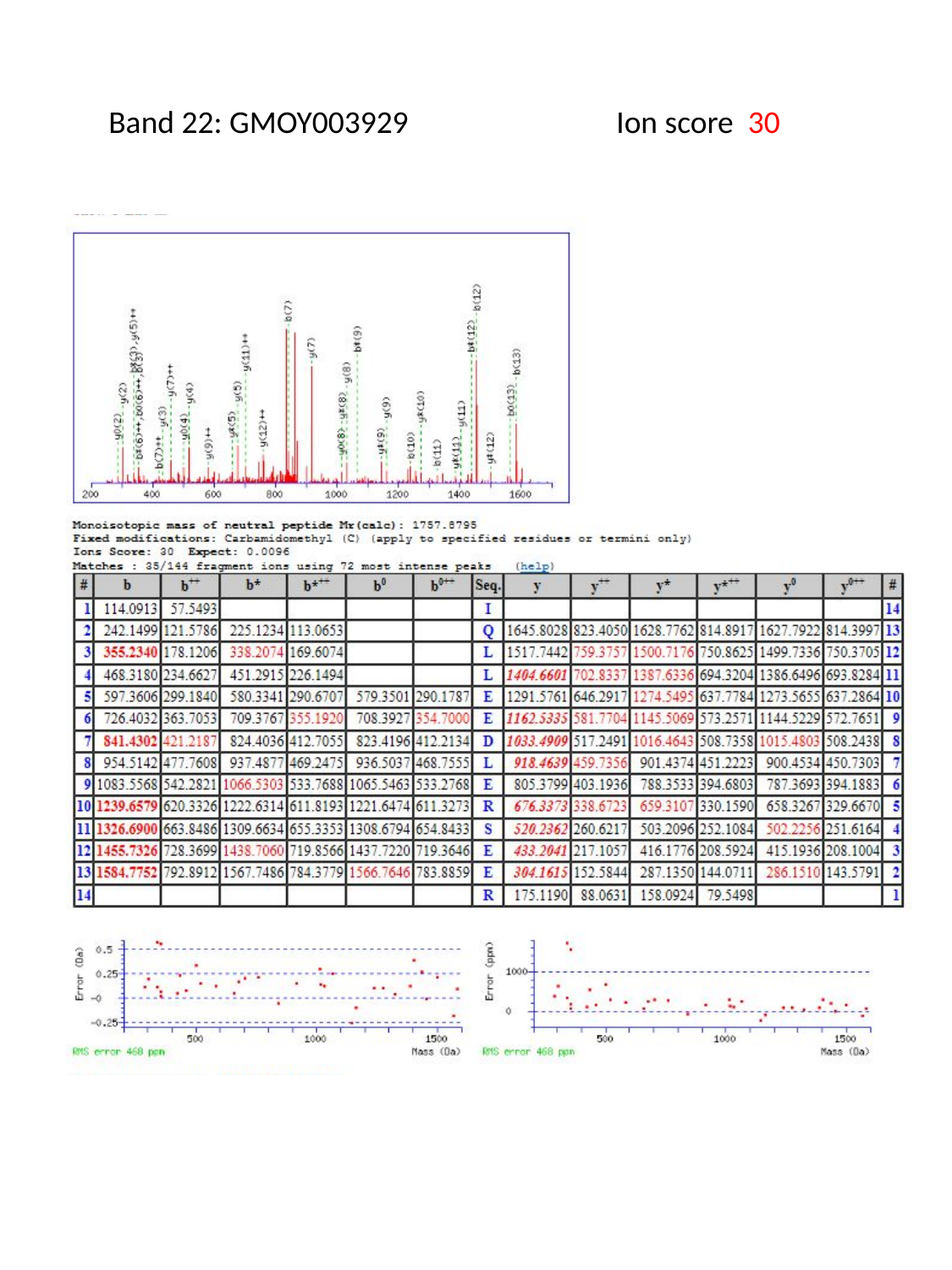

Band 22: GMOY003929 		Ion score 30

## Slide 95
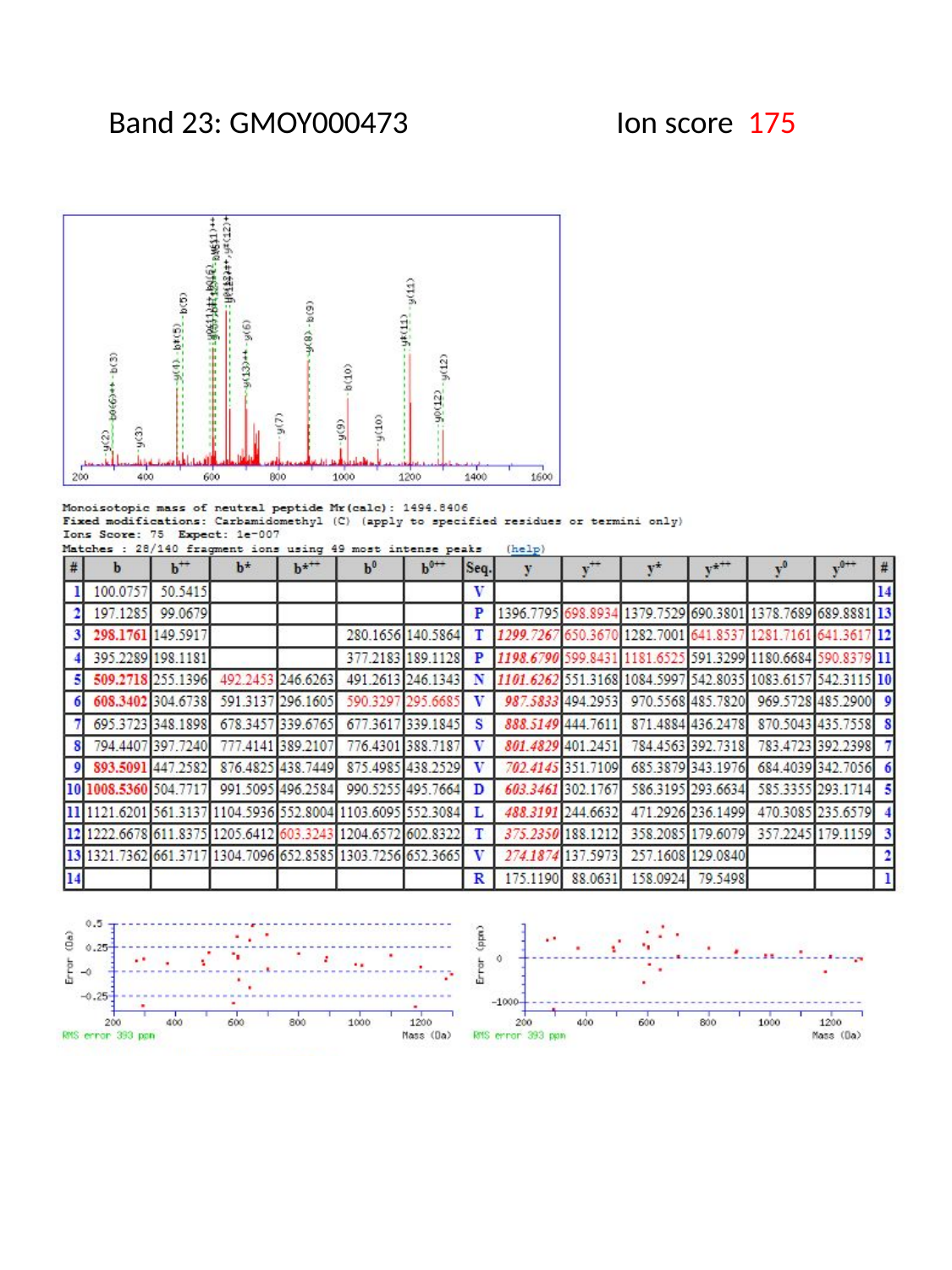

Band 23: GMOY000473 		Ion score 175

## Slide 96
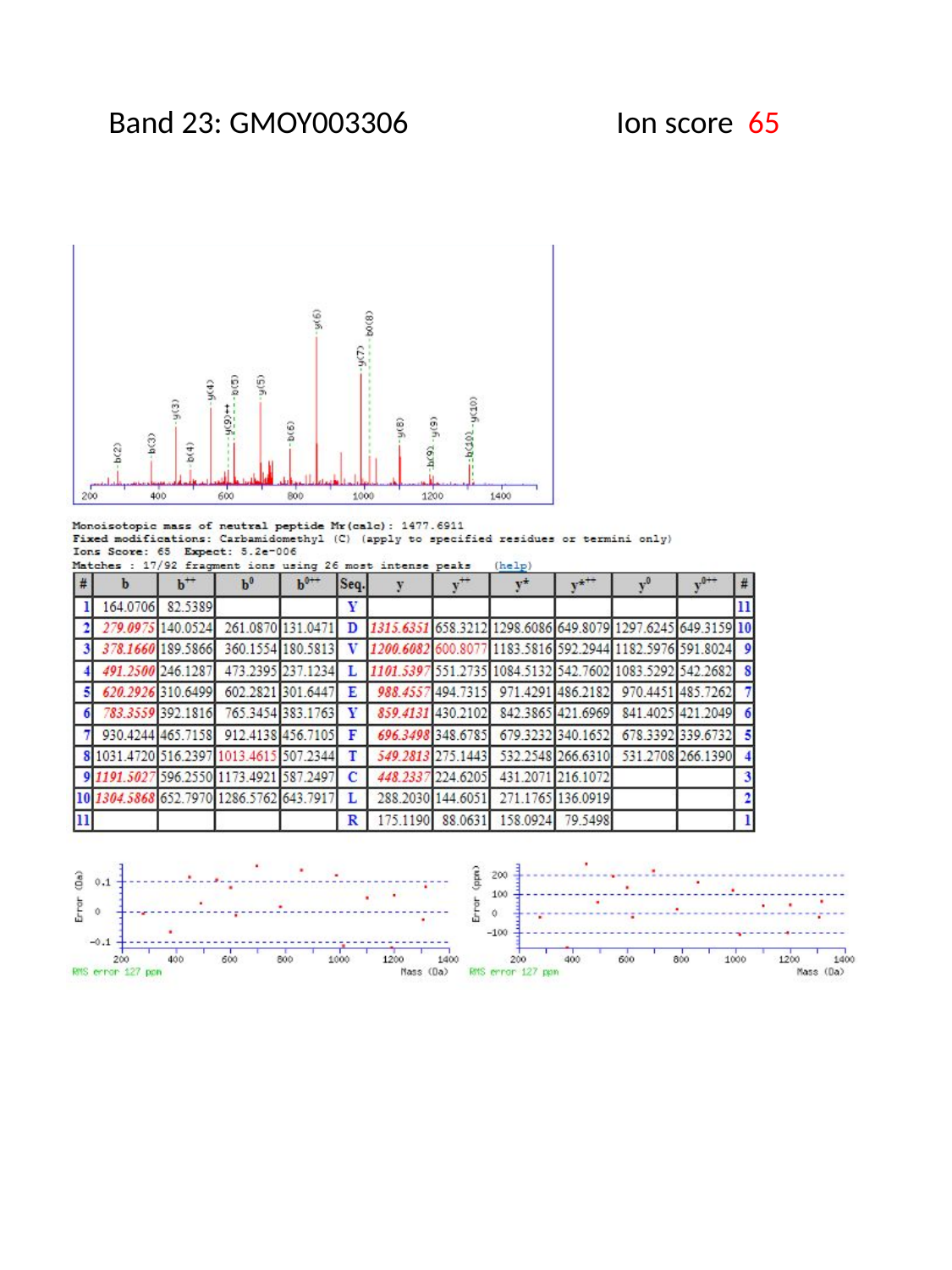

Band 23: GMOY003306 		Ion score 65

## Slide 97
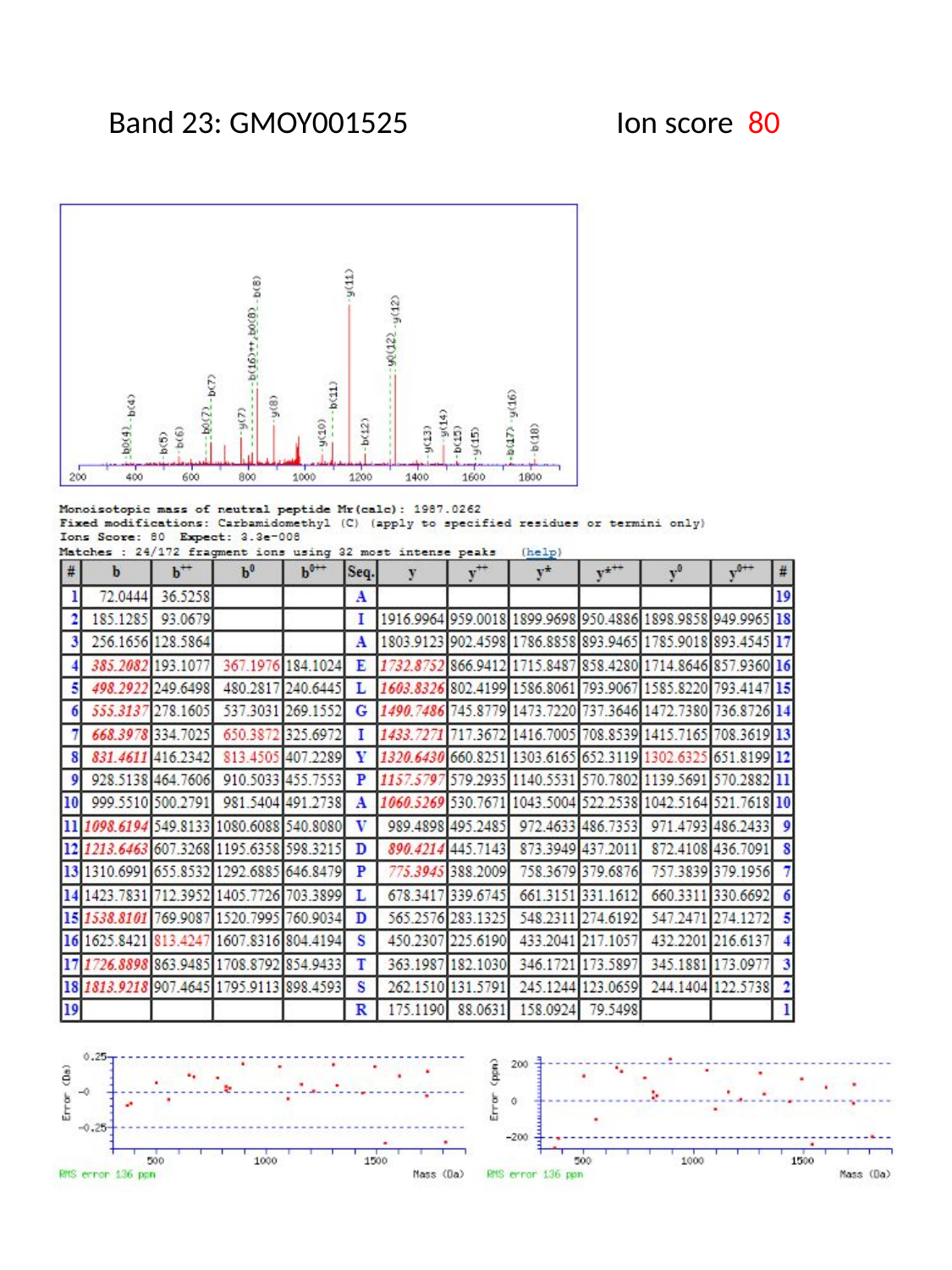

Band 23: GMOY001525 		Ion score 80

## Slide 98
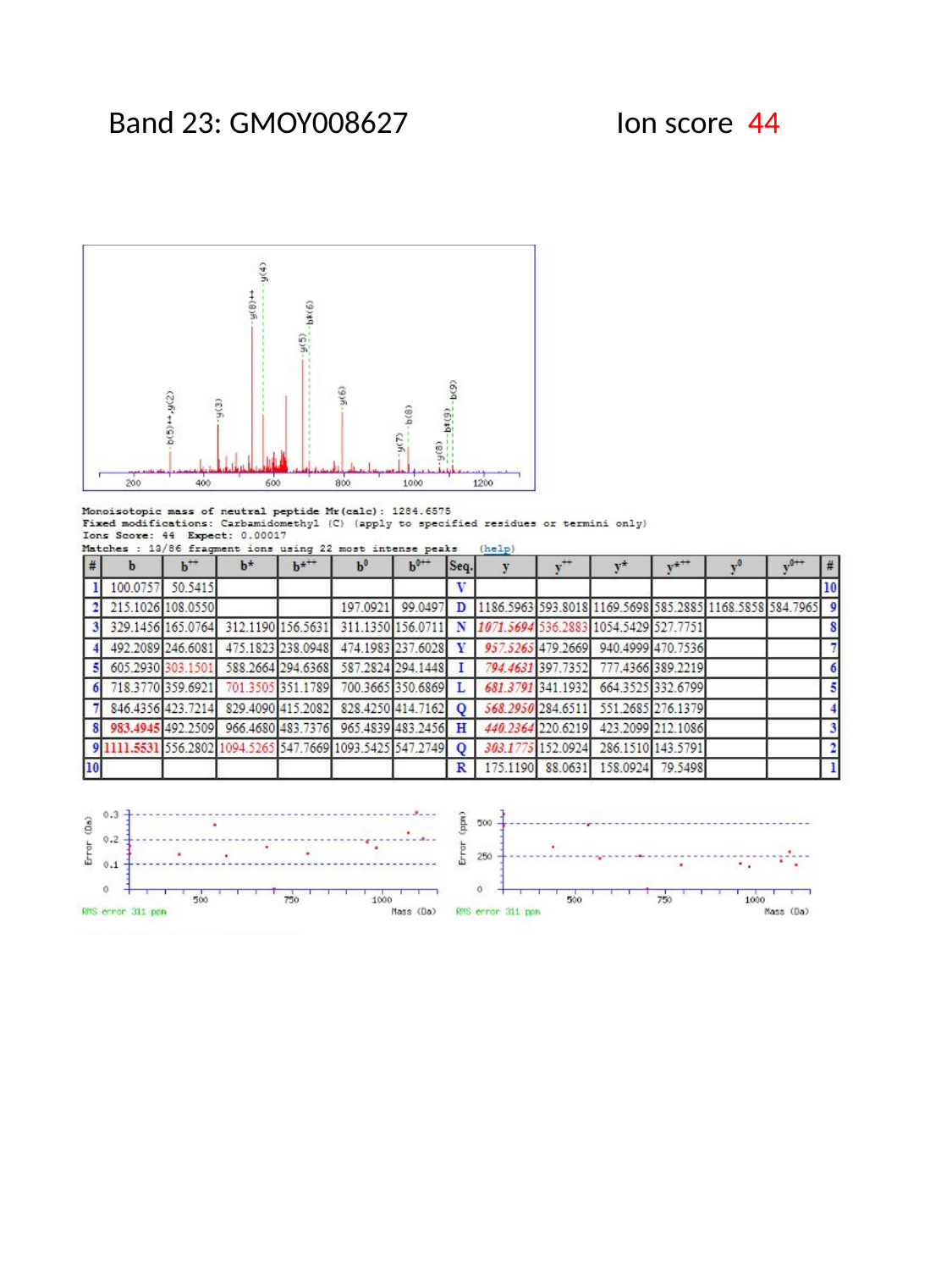

Band 23: GMOY008627 		Ion score 44

## Slide 99
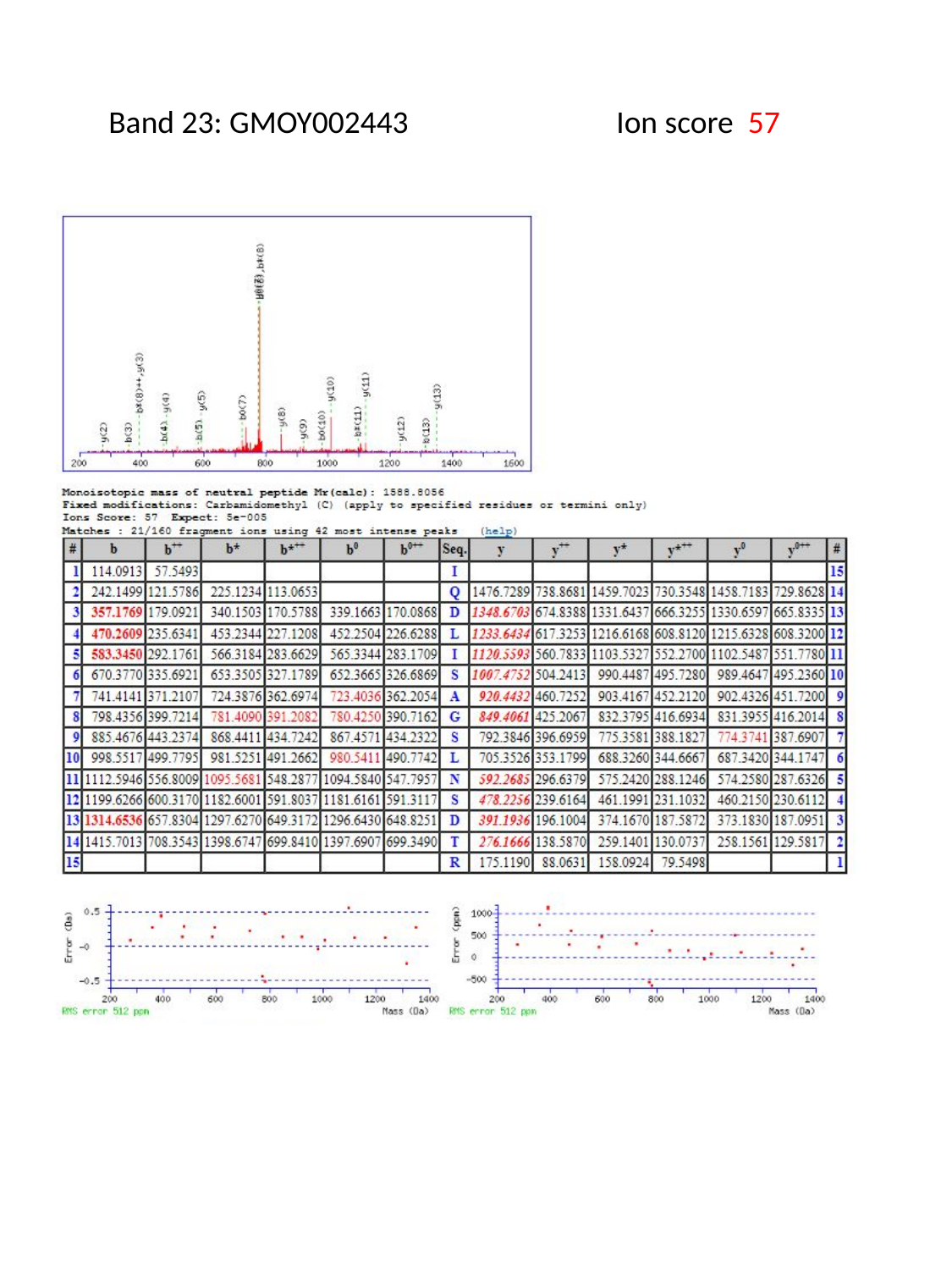

Band 23: GMOY002443 		Ion score 57

## Slide 100
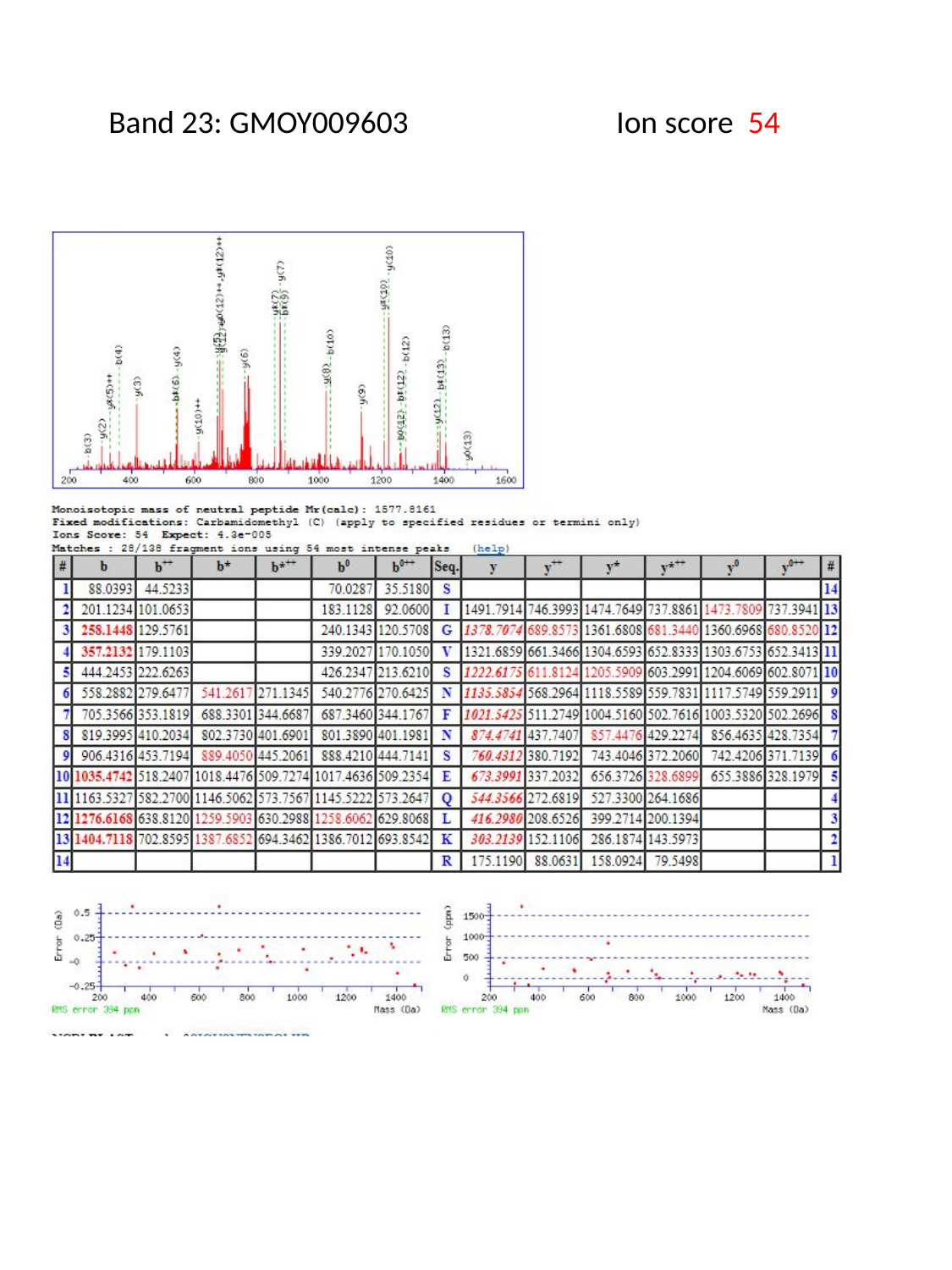

Band 23: GMOY009603 		Ion score 54

## Slide 101
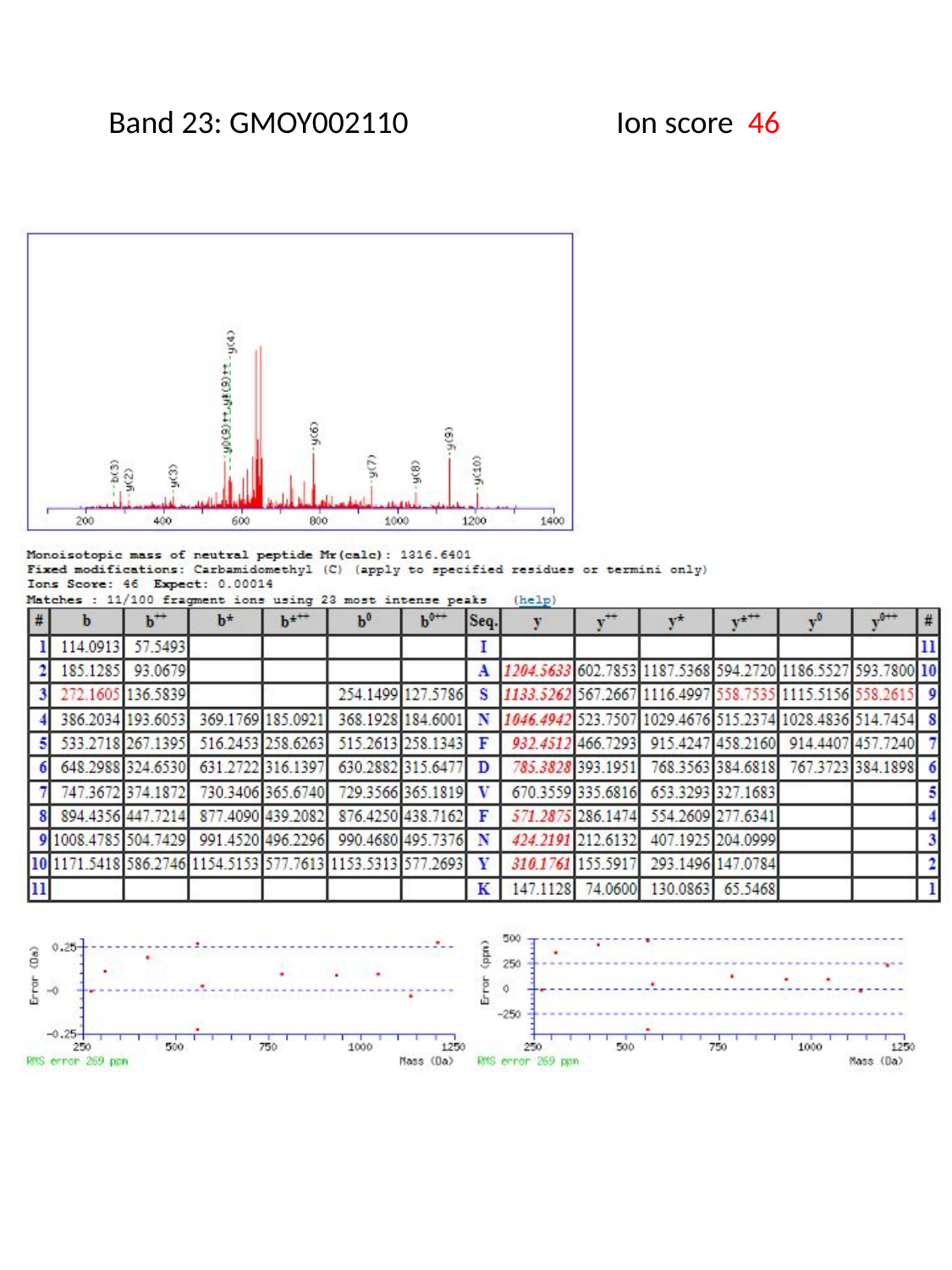

Band 23: GMOY002110 		Ion score 46

## Slide 102
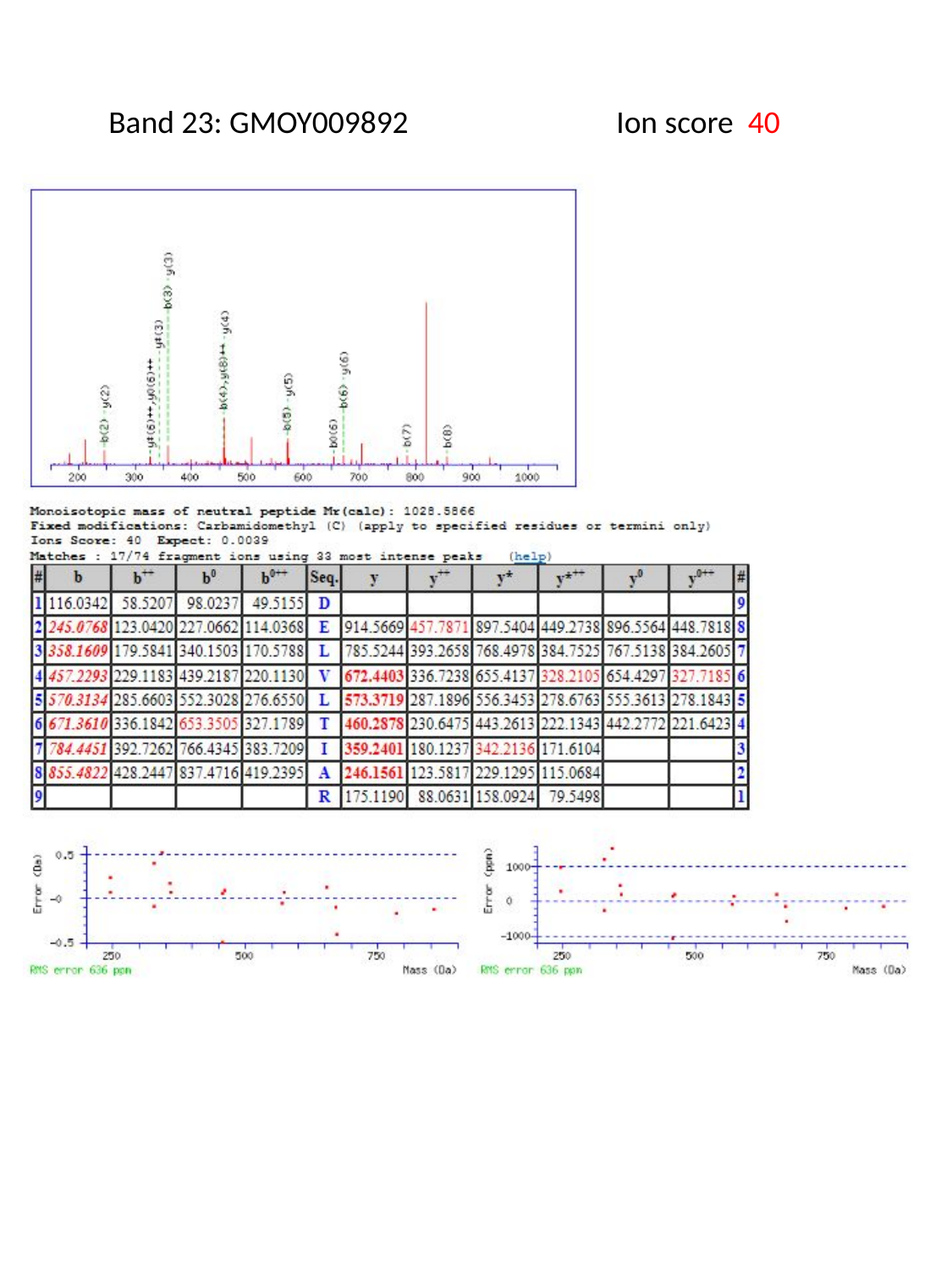

Band 23: GMOY009892 		Ion score 40

## Slide 103
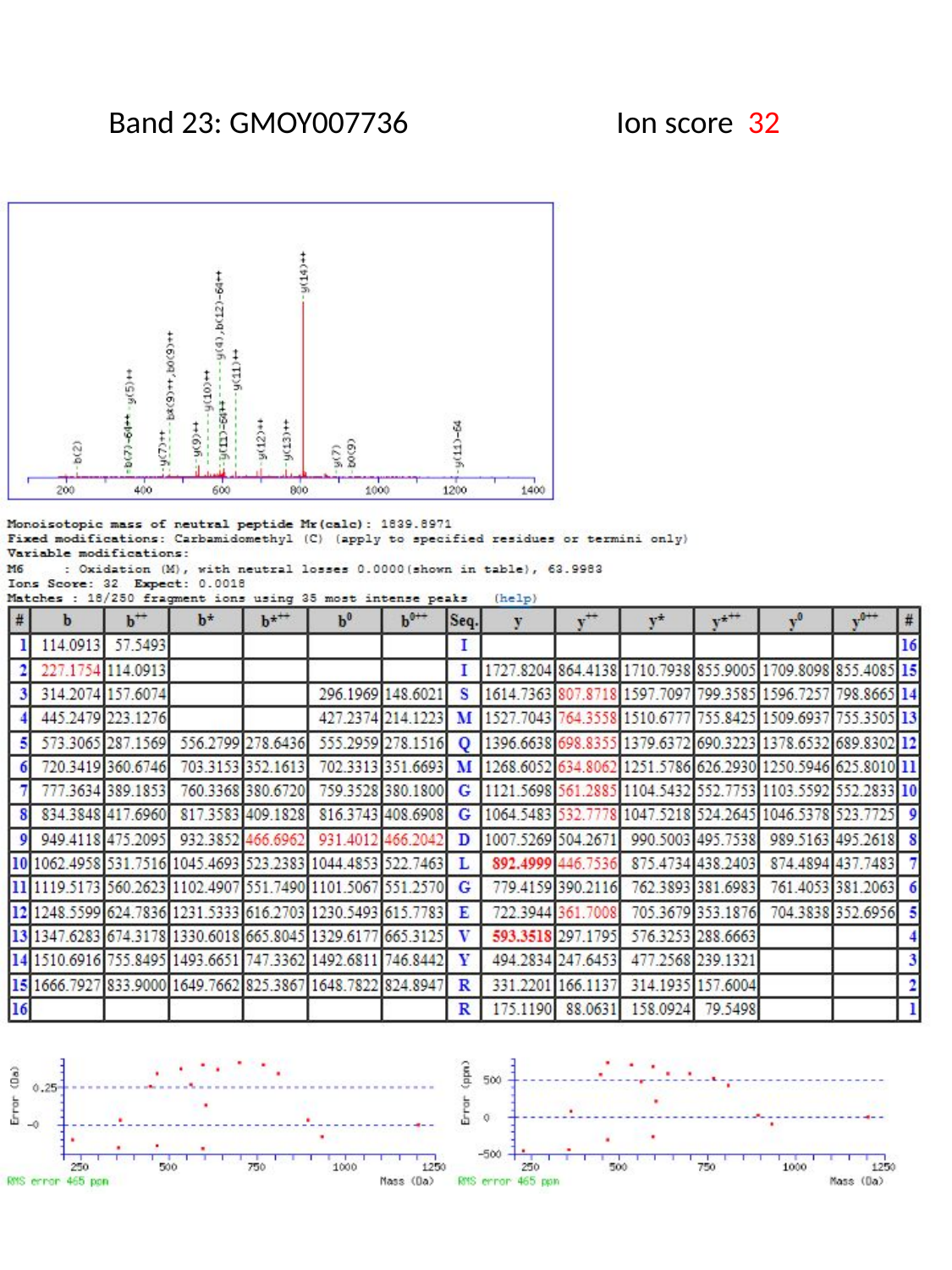

Band 23: GMOY007736 		Ion score 32

## Slide 104
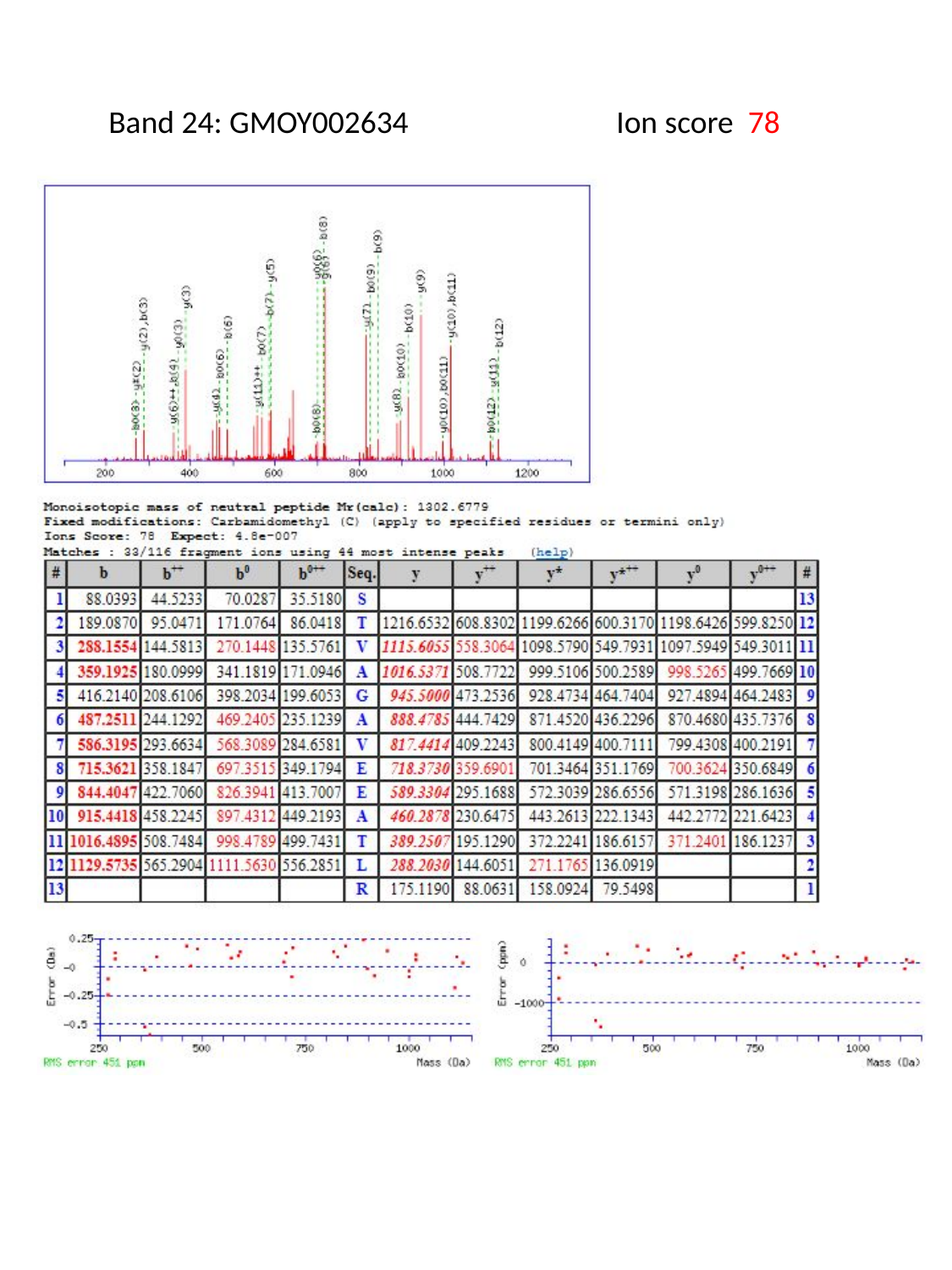

Band 24: GMOY002634 		Ion score 78

## Slide 105
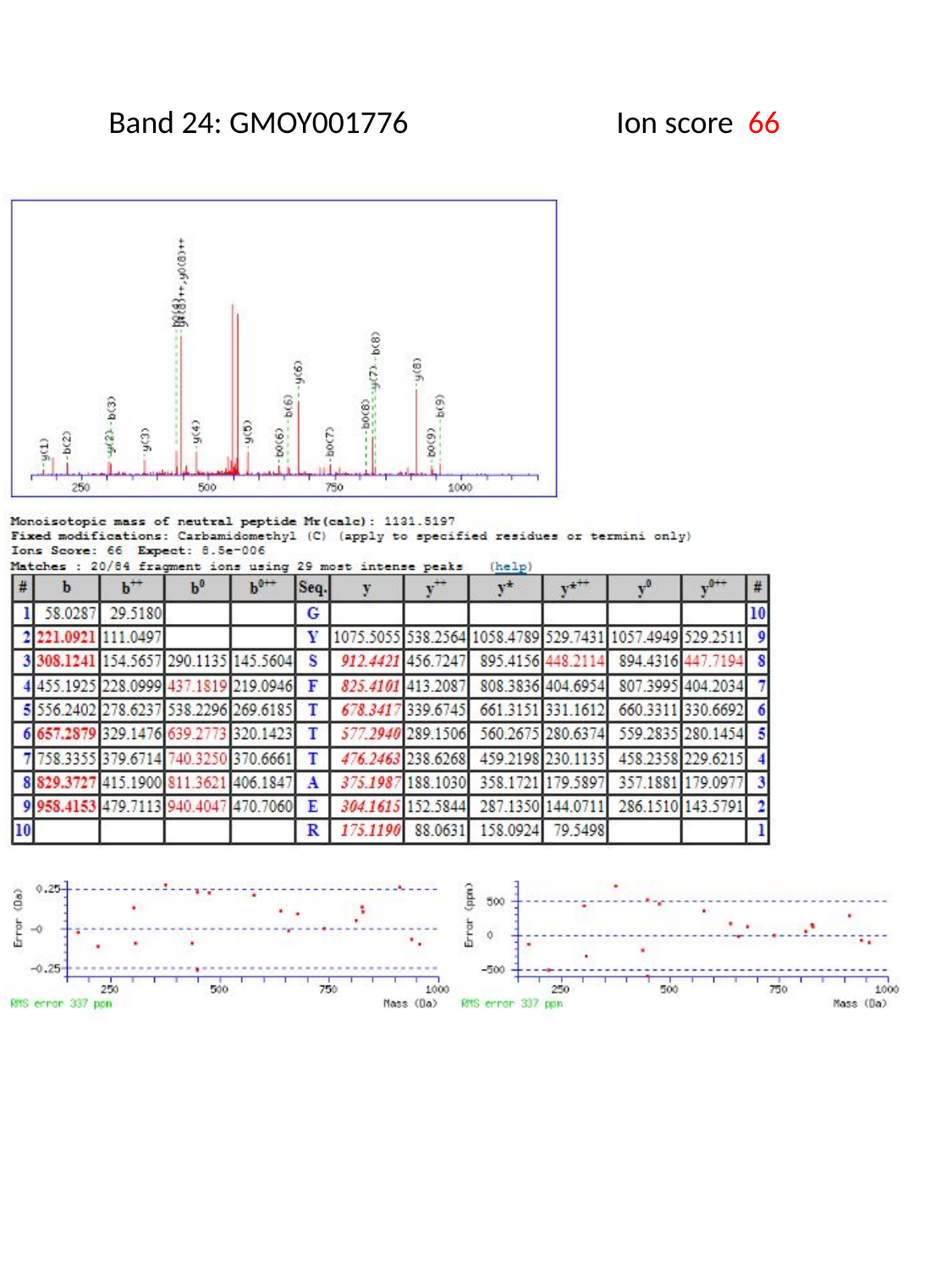

Band 24: GMOY001776 		Ion score 66

## Slide 106
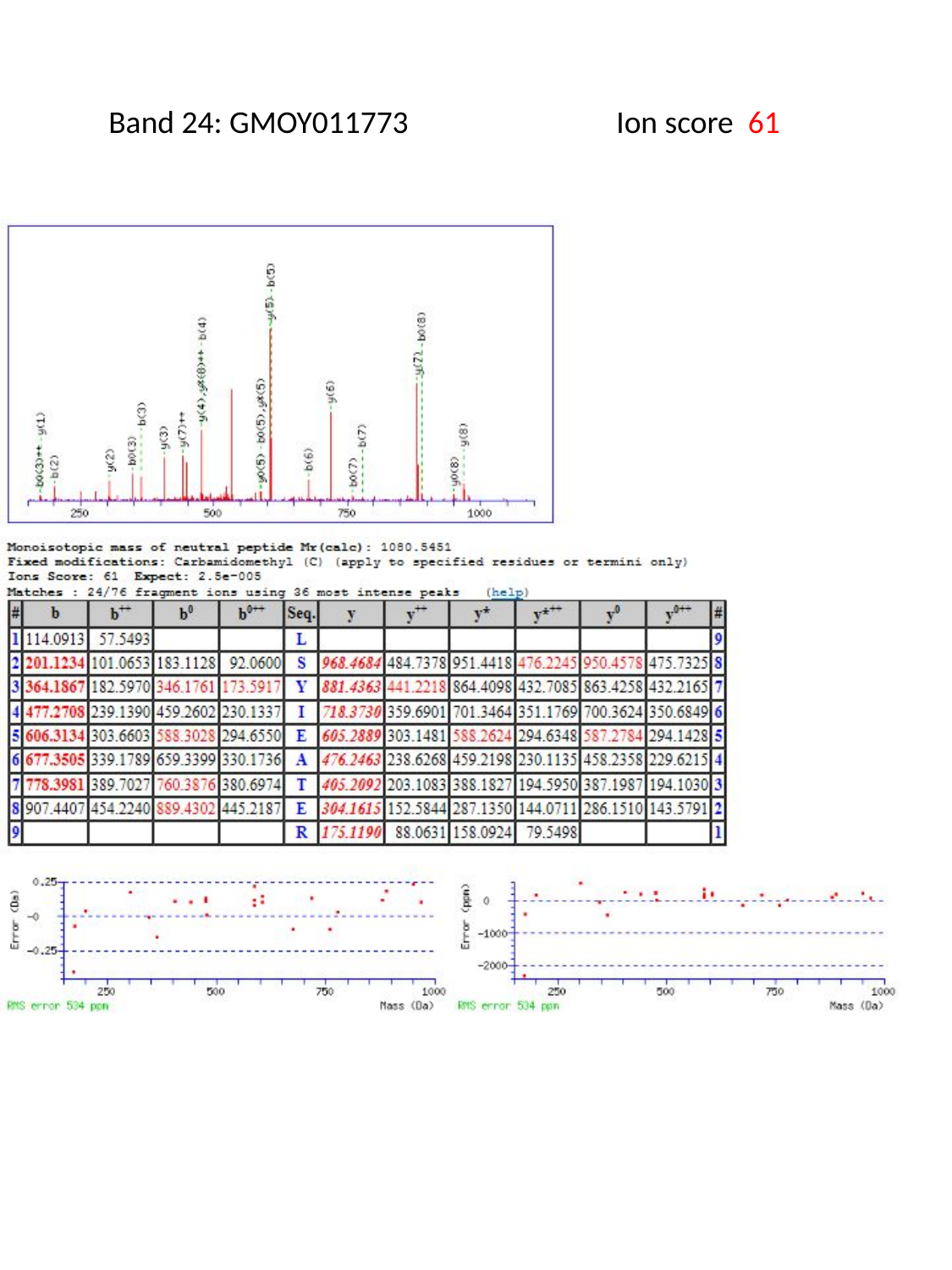

Band 24: GMOY011773 		Ion score 61

## Slide 107
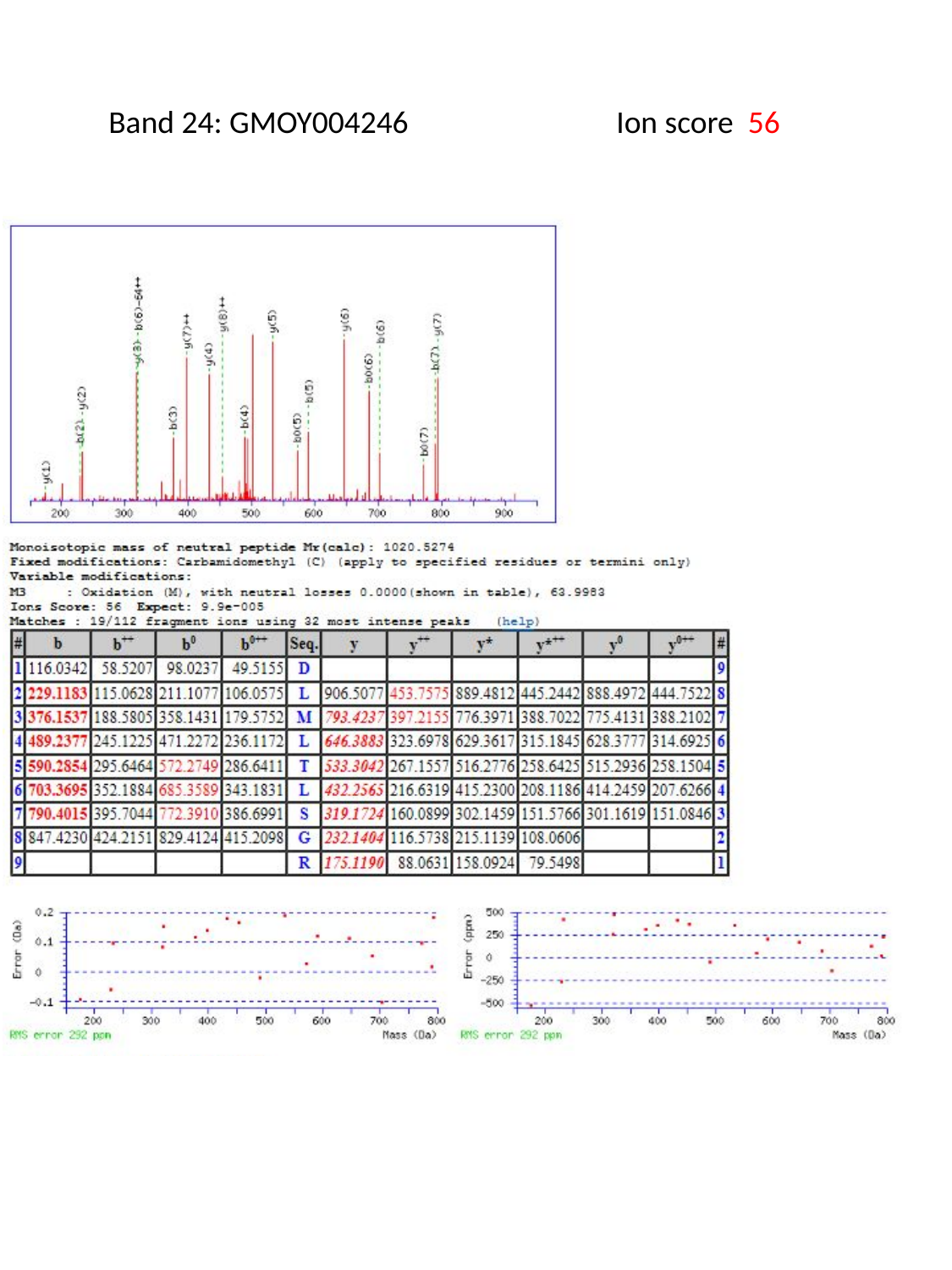

Band 24: GMOY004246 		Ion score 56

## Slide 108
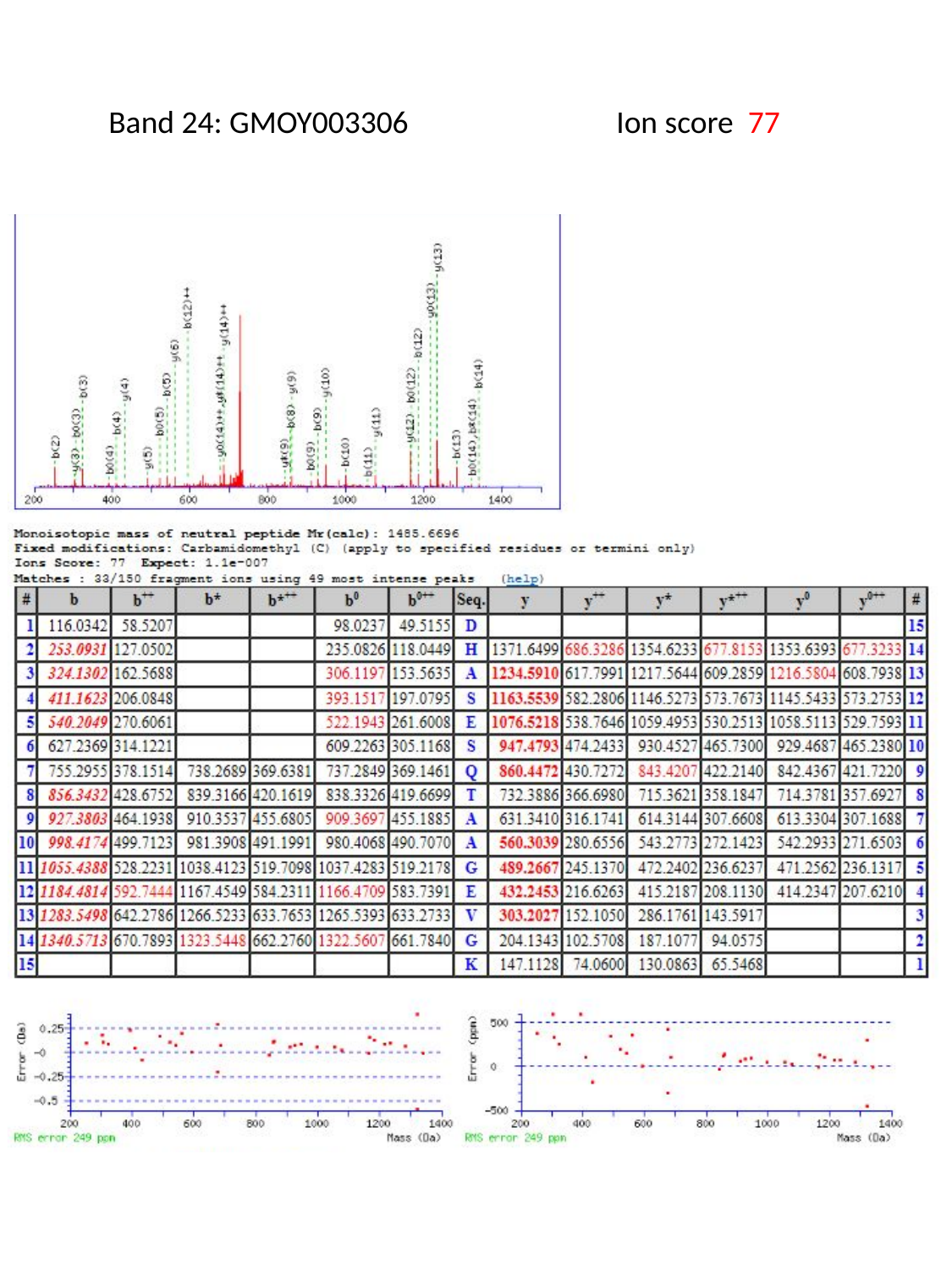

Band 24: GMOY003306 		Ion score 77

## Slide 109
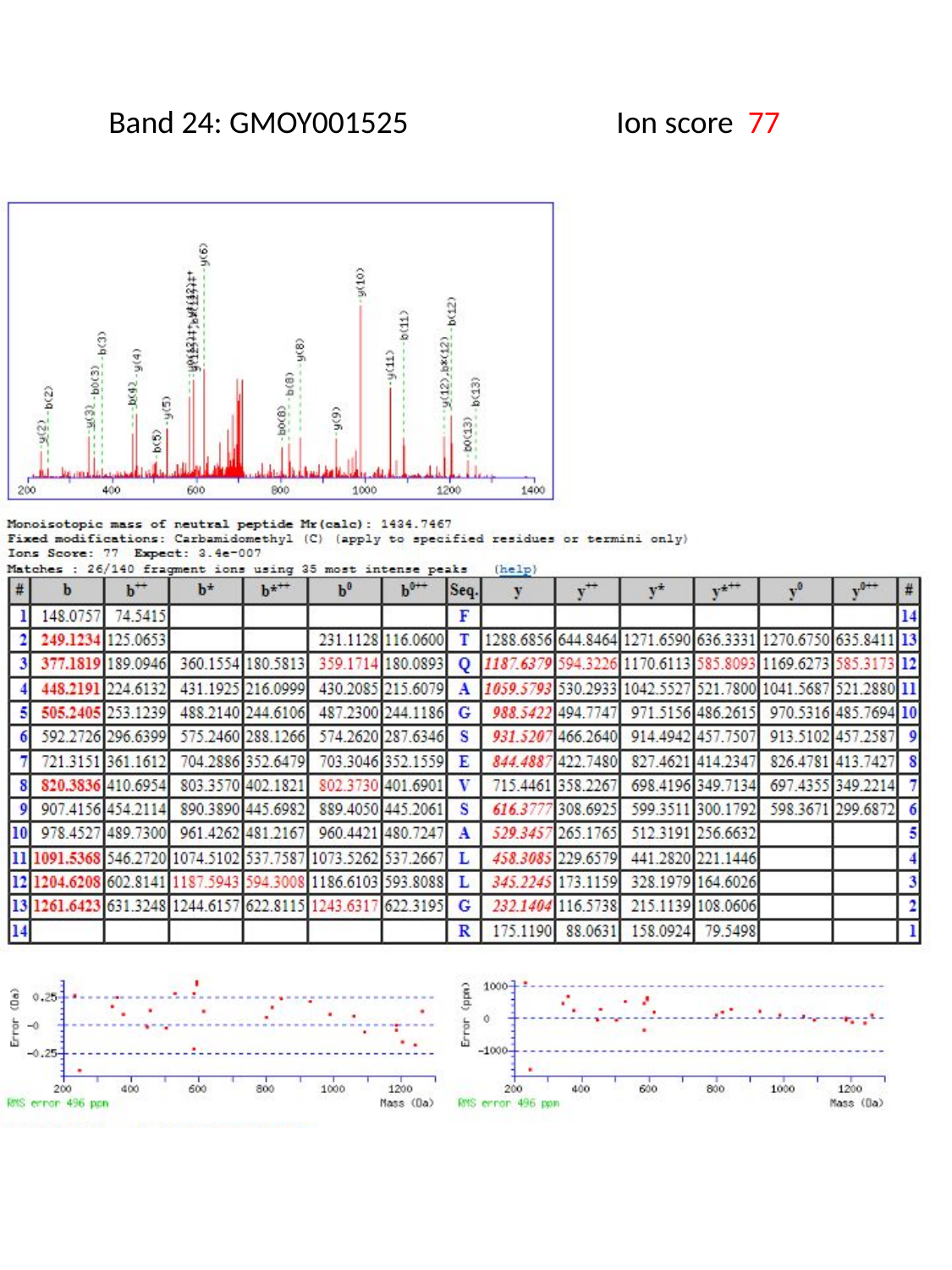

Band 24: GMOY001525 		Ion score 77

## Slide 110
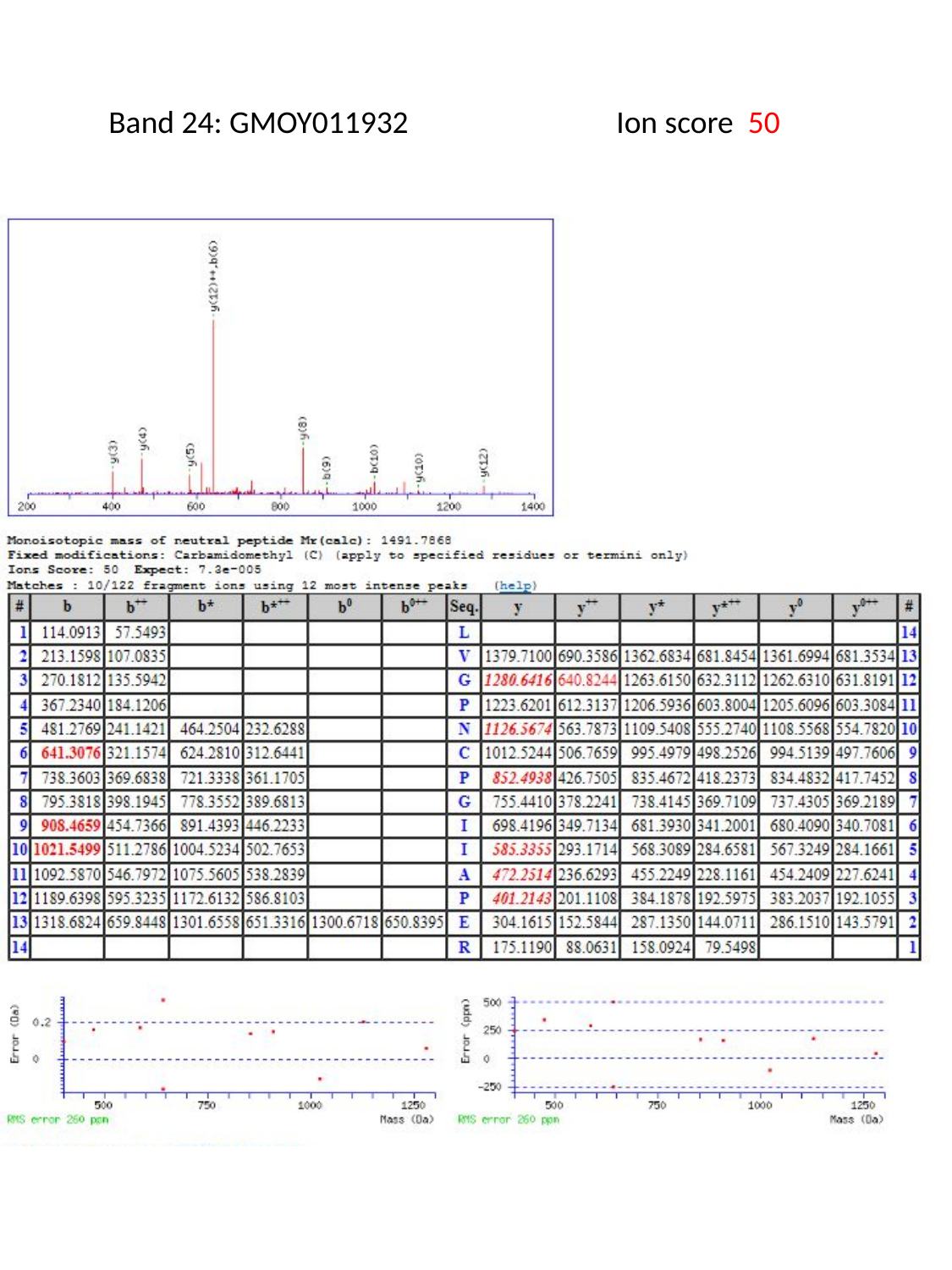

Band 24: GMOY011932 		Ion score 50

## Slide 111
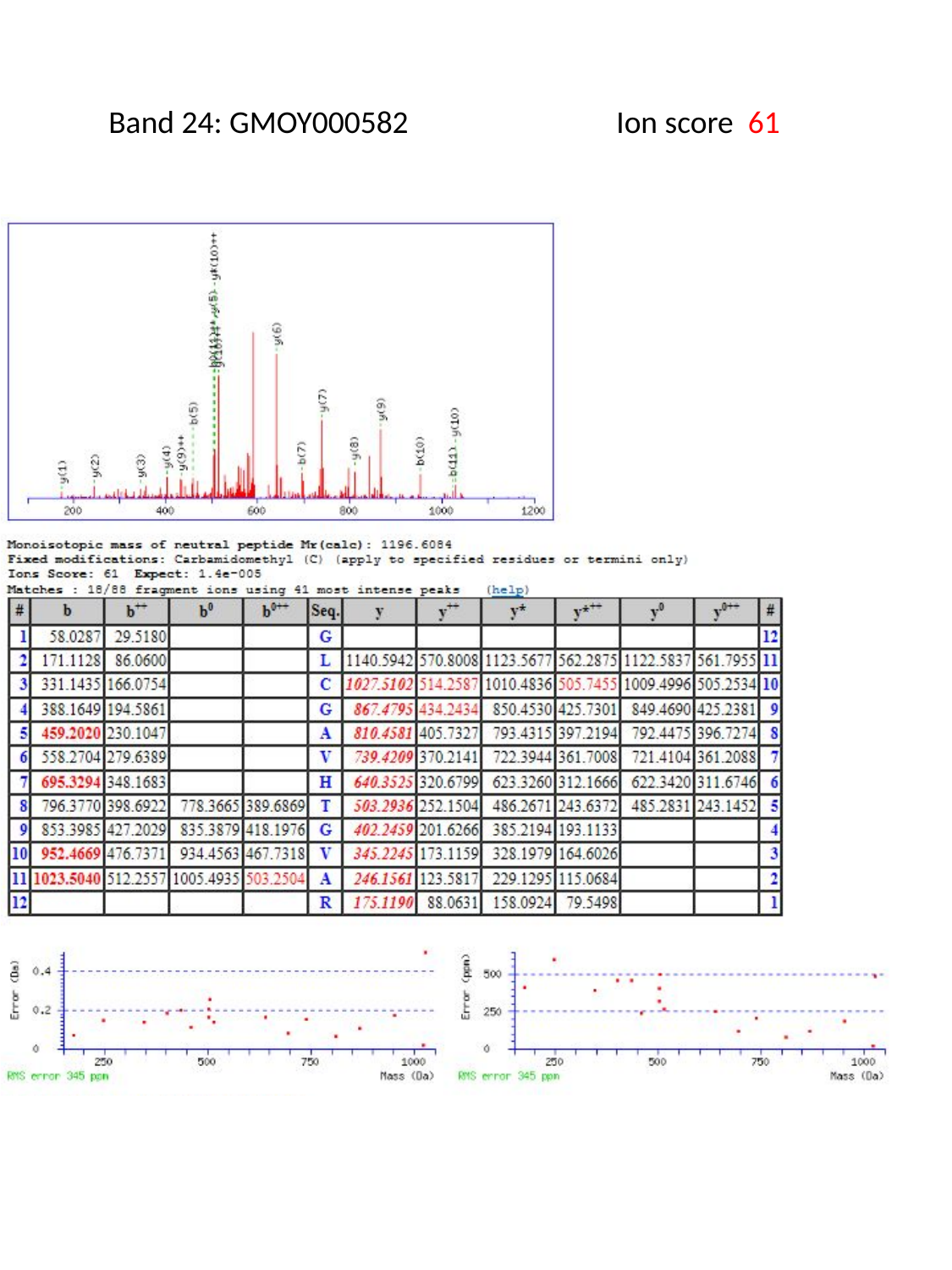

Band 24: GMOY000582 		Ion score 61

## Slide 112
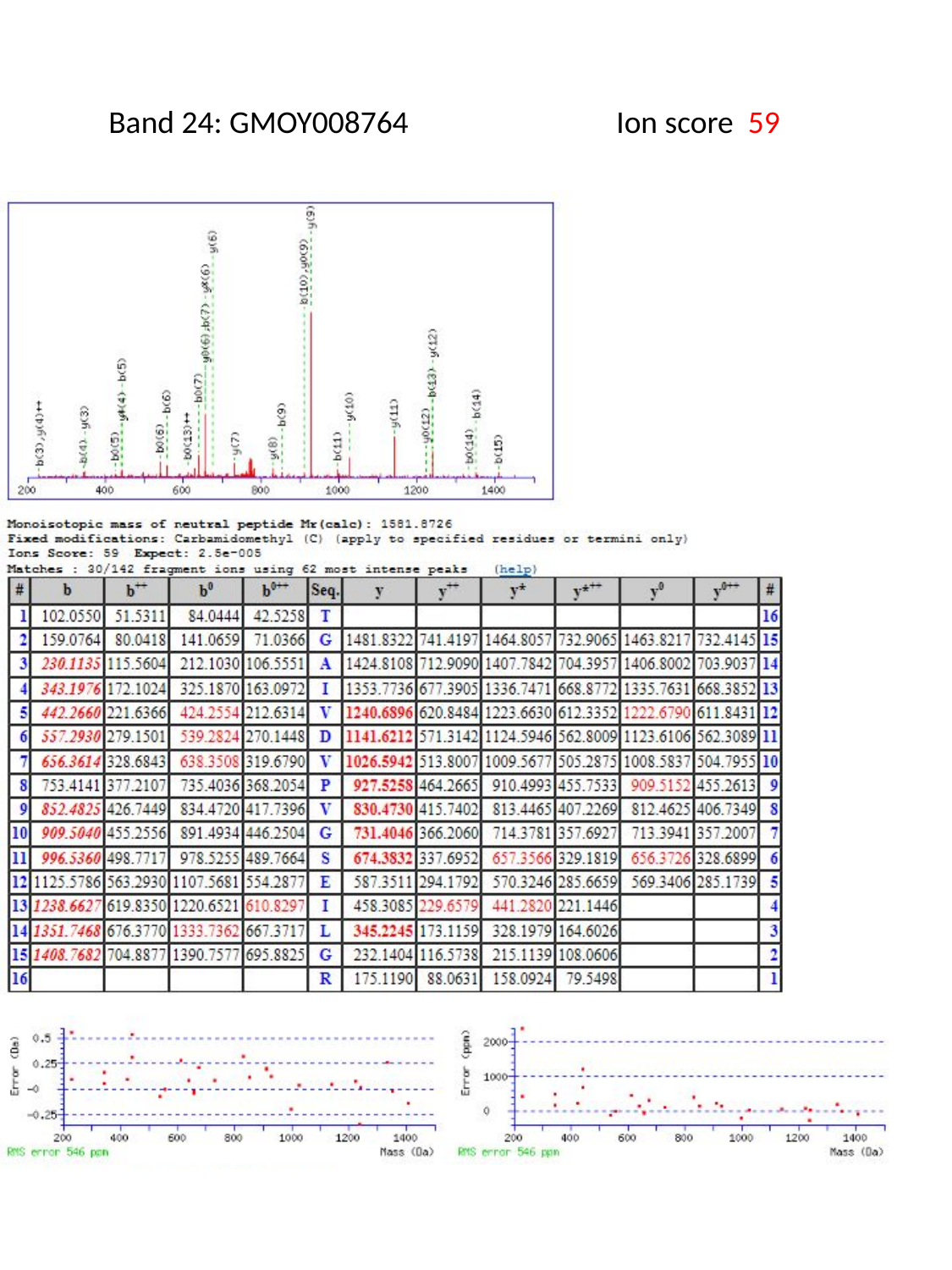

Band 24: GMOY008764 		Ion score 59

## Slide 113
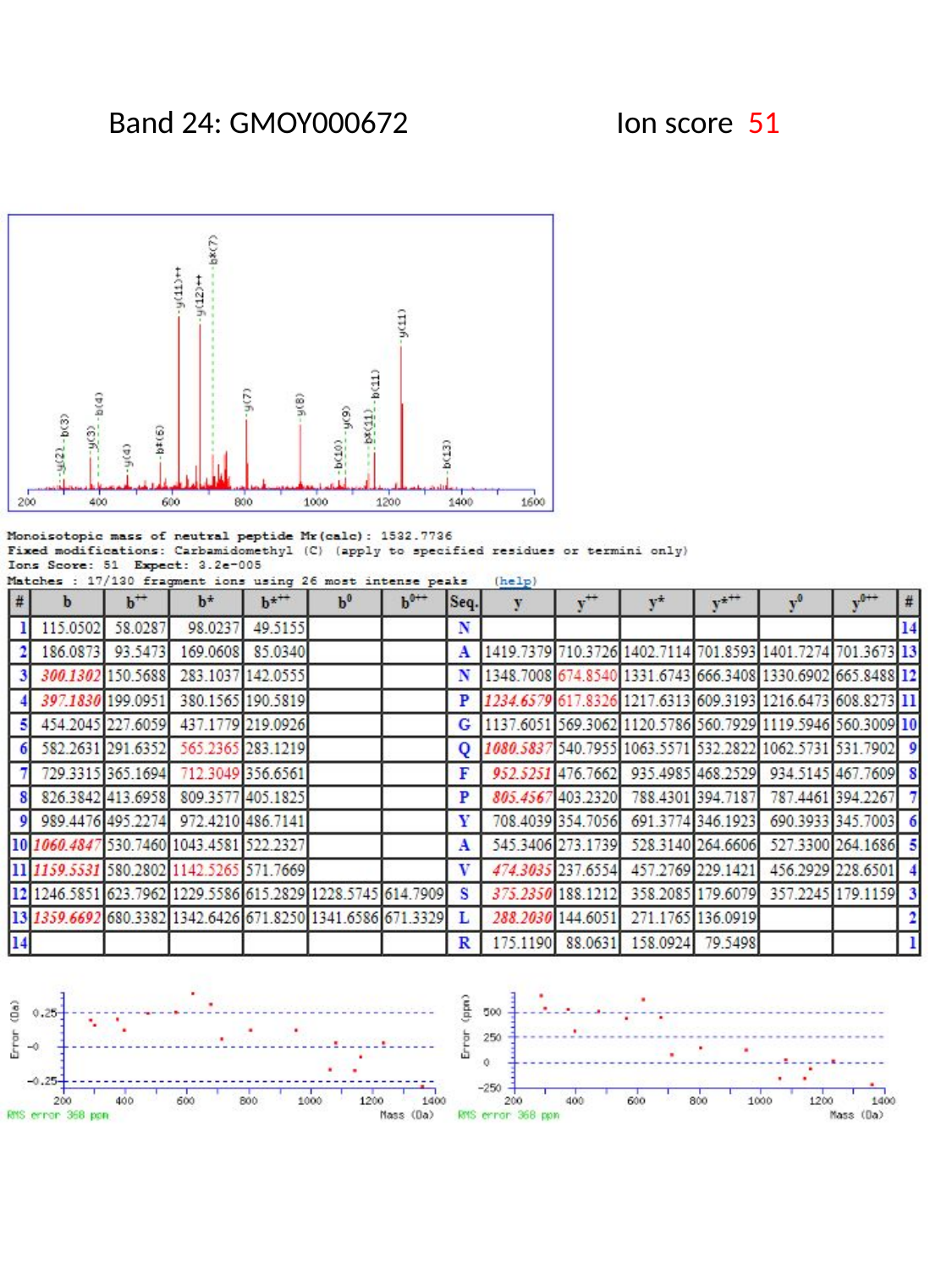

Band 24: GMOY000672 		Ion score 51

## Slide 114
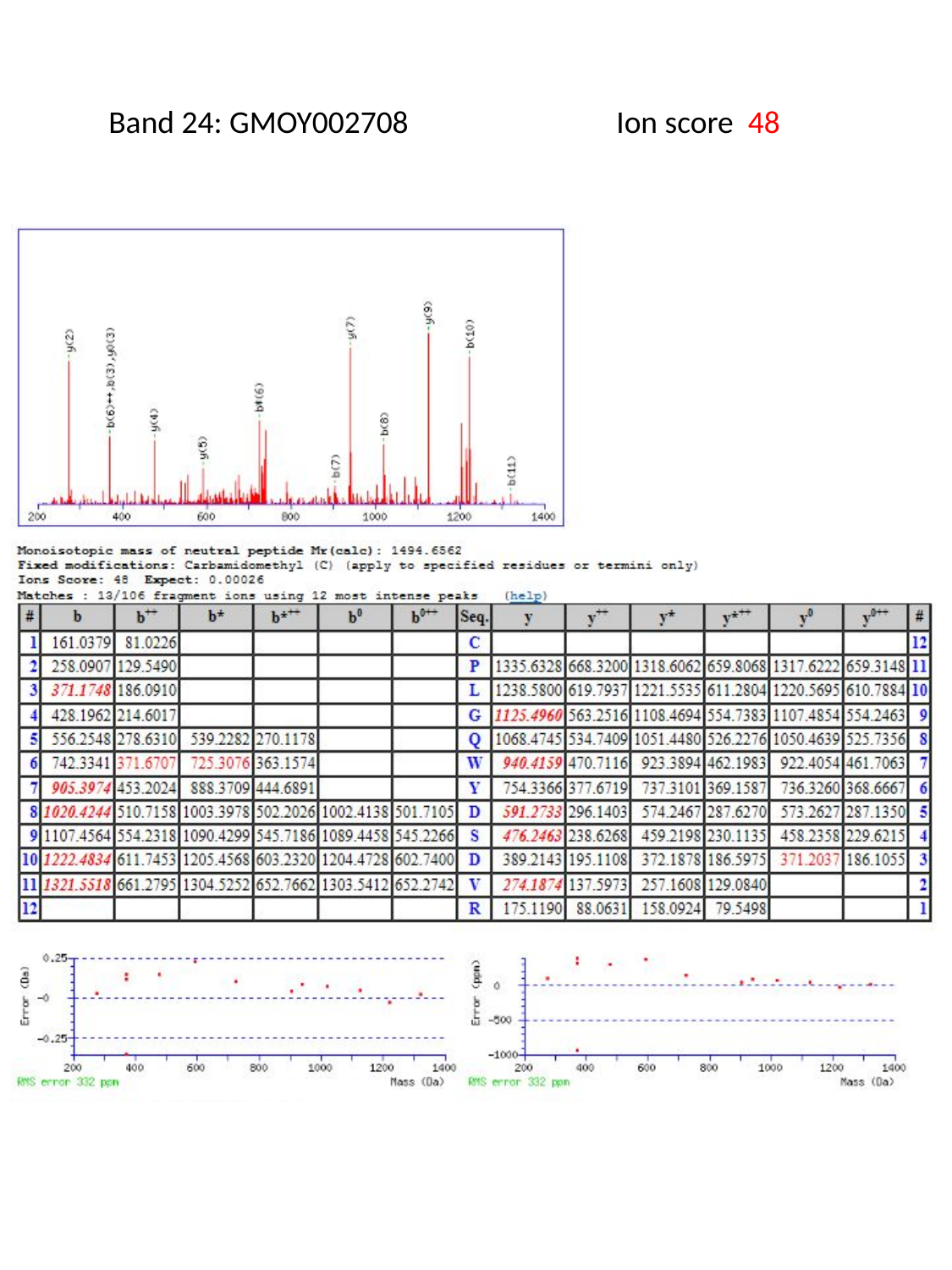

Band 24: GMOY002708 		Ion score 48

## Slide 115
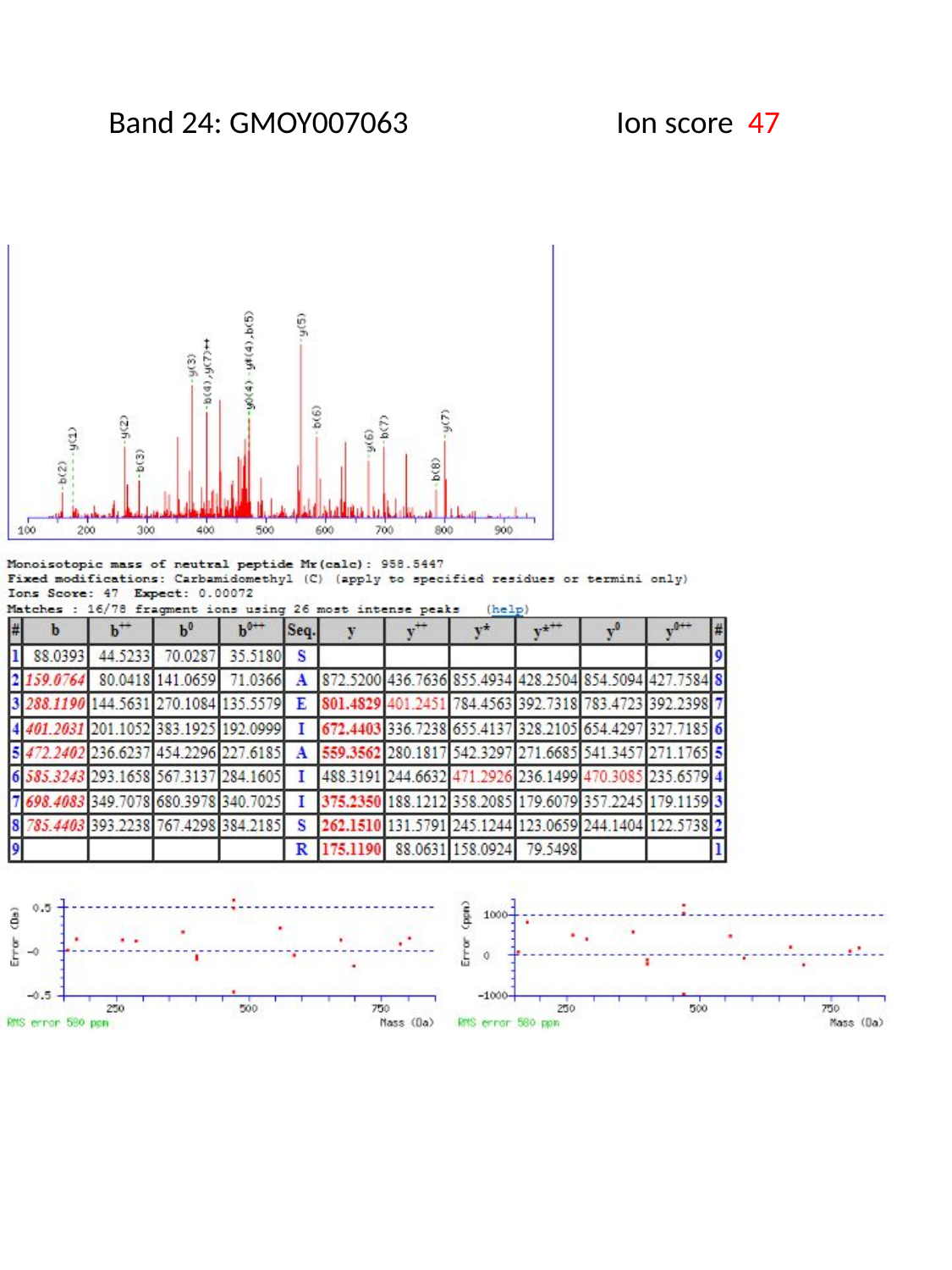

Band 24: GMOY007063 		Ion score 47

## Slide 116
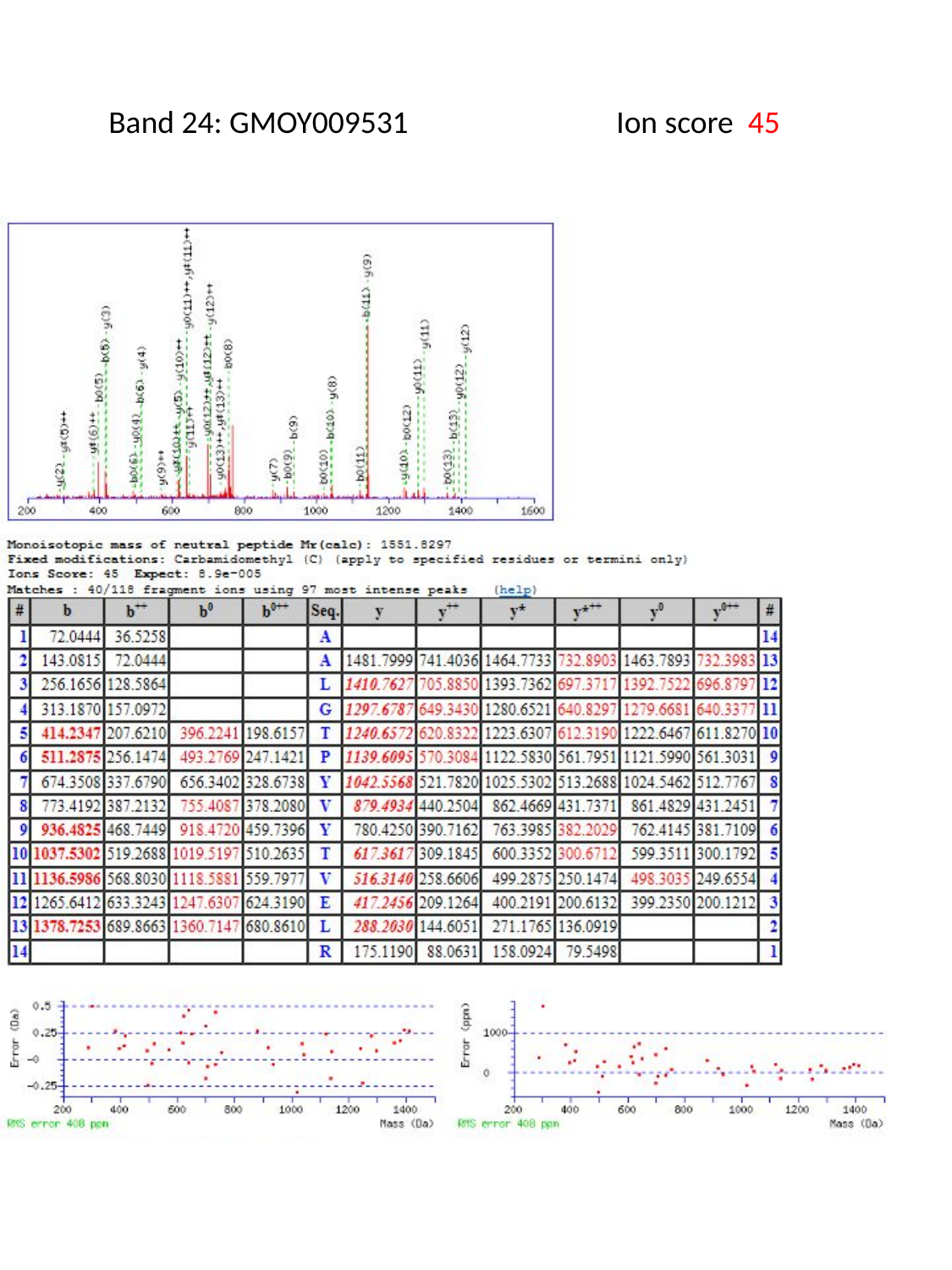

Band 24: GMOY009531 		Ion score 45

## Slide 117
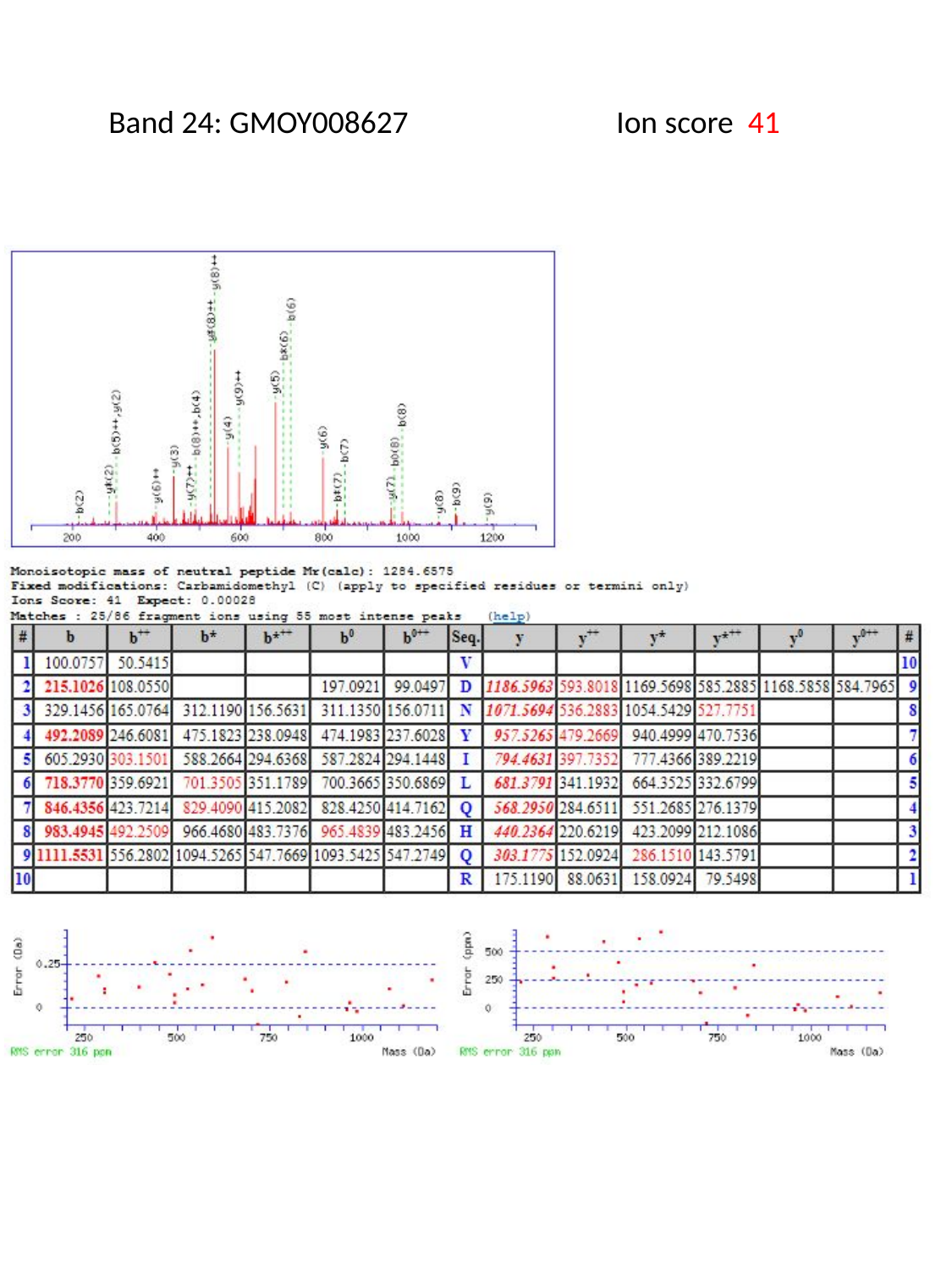

Band 24: GMOY008627 		Ion score 41

## Slide 118
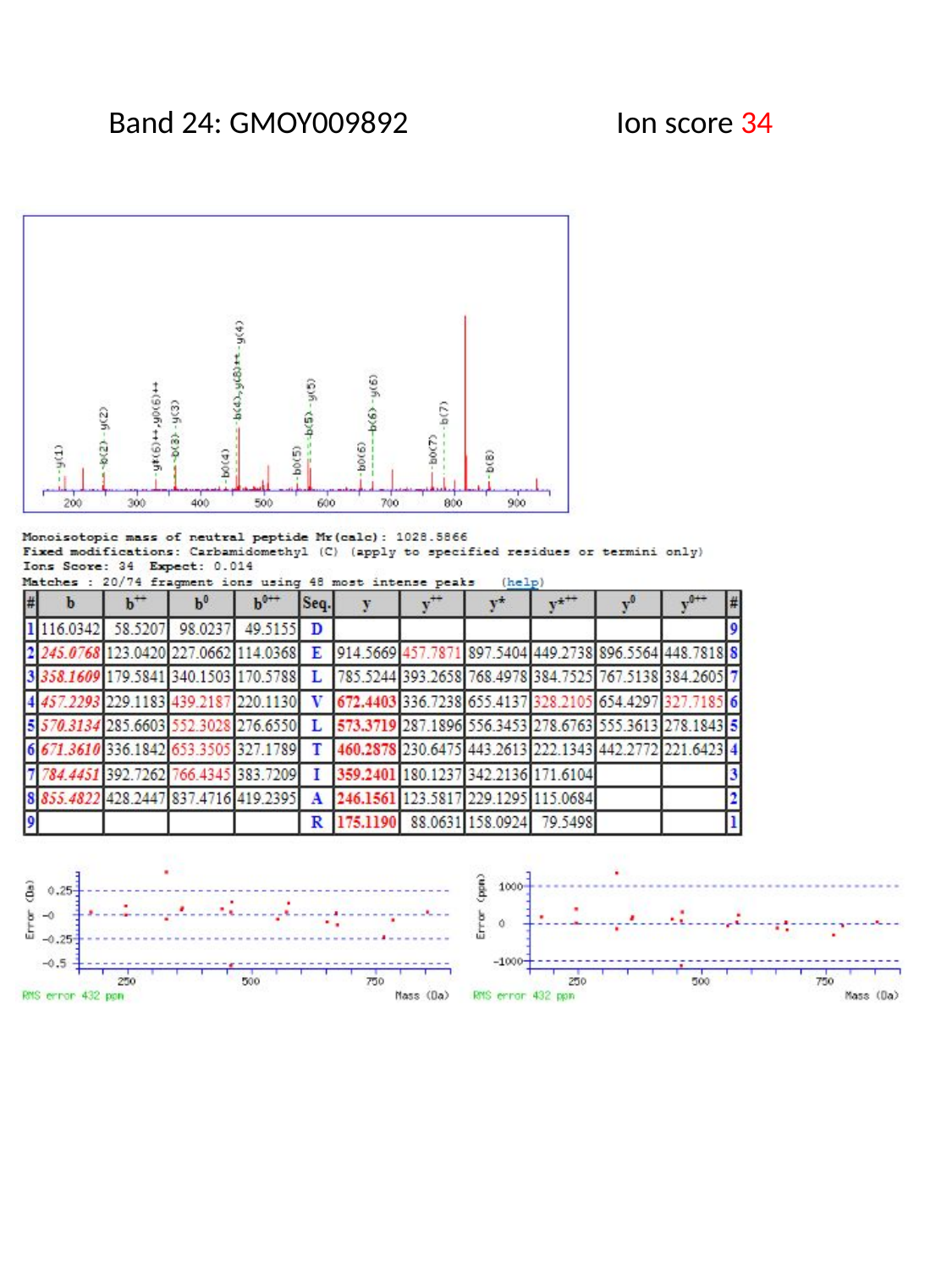

Band 24: GMOY009892 		Ion score 34

## Slide 119
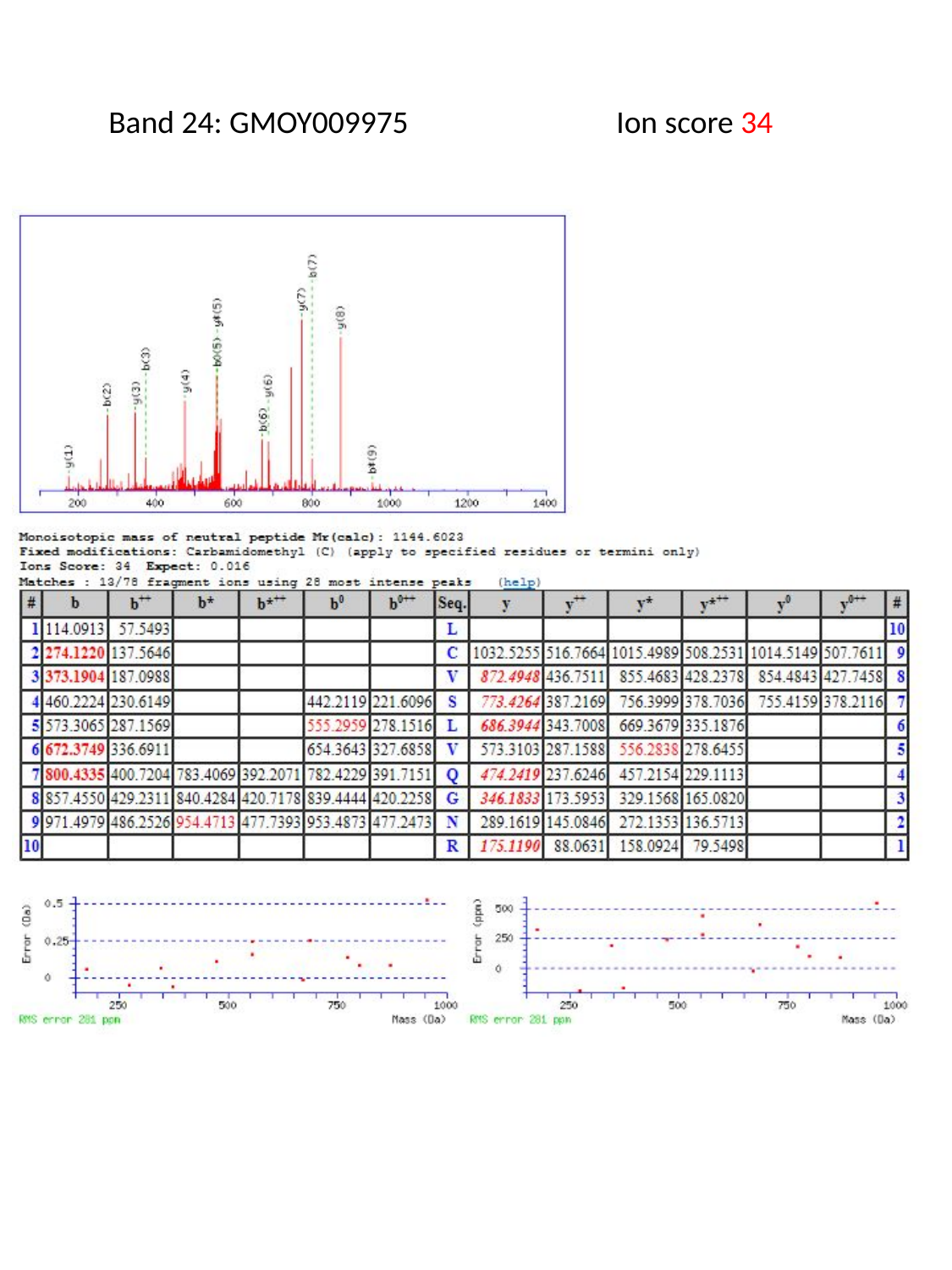

Band 24: GMOY009975 		Ion score 34

## Slide 120
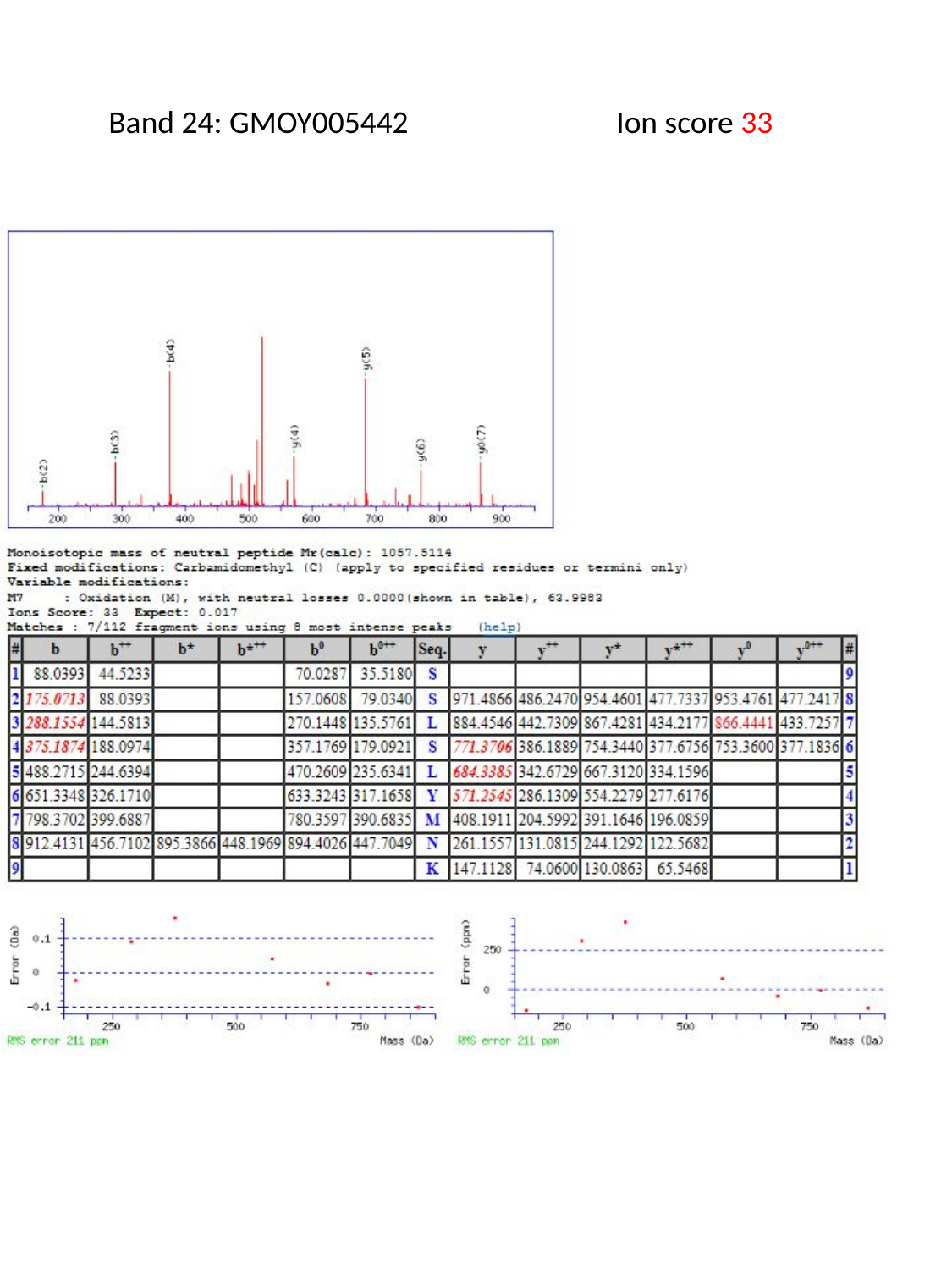

Band 24: GMOY005442 		Ion score 33

## Slide 121
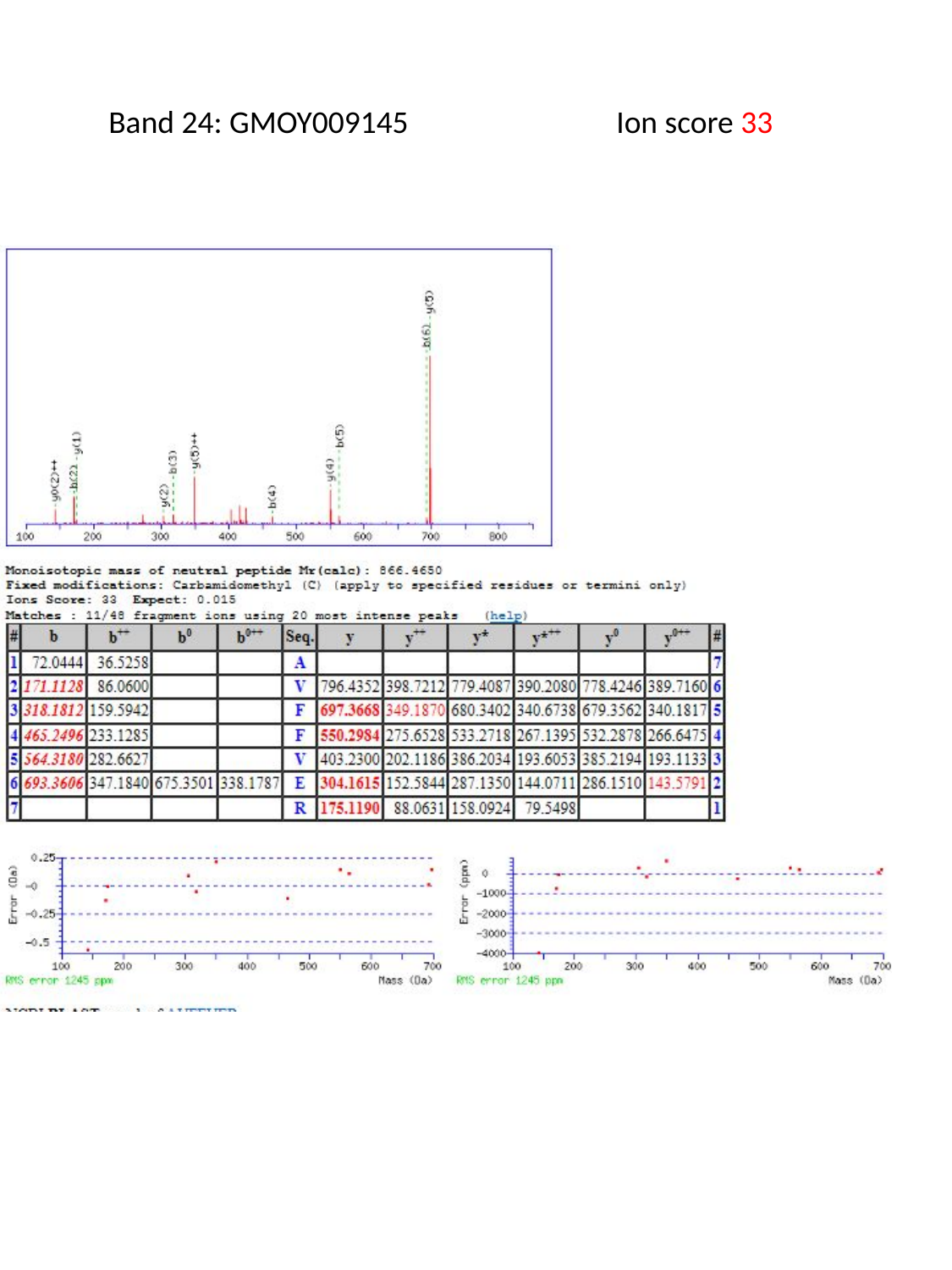

Band 24: GMOY009145 		Ion score 33

## Slide 122
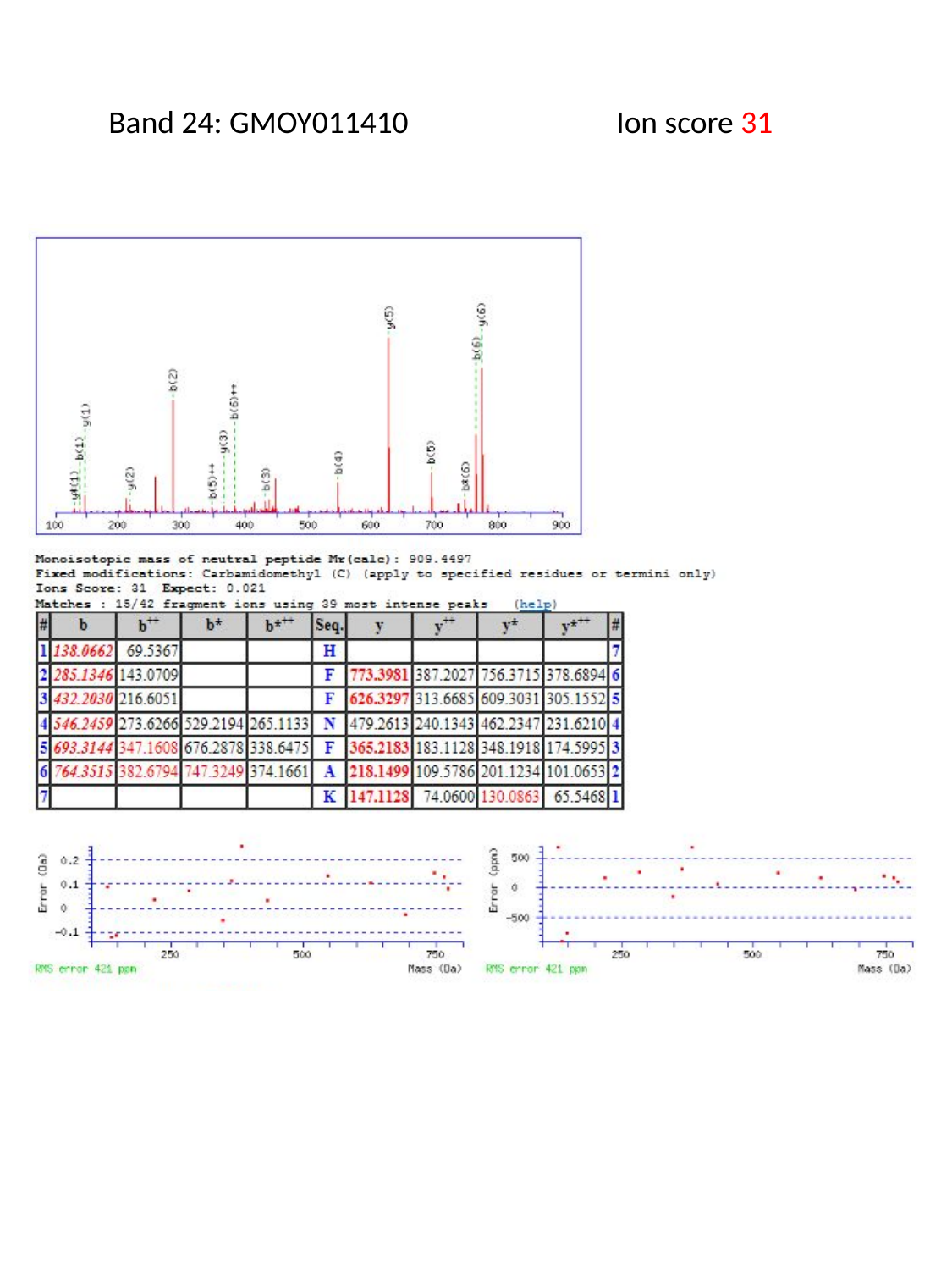

Band 24: GMOY011410 		Ion score 31

## Slide 123
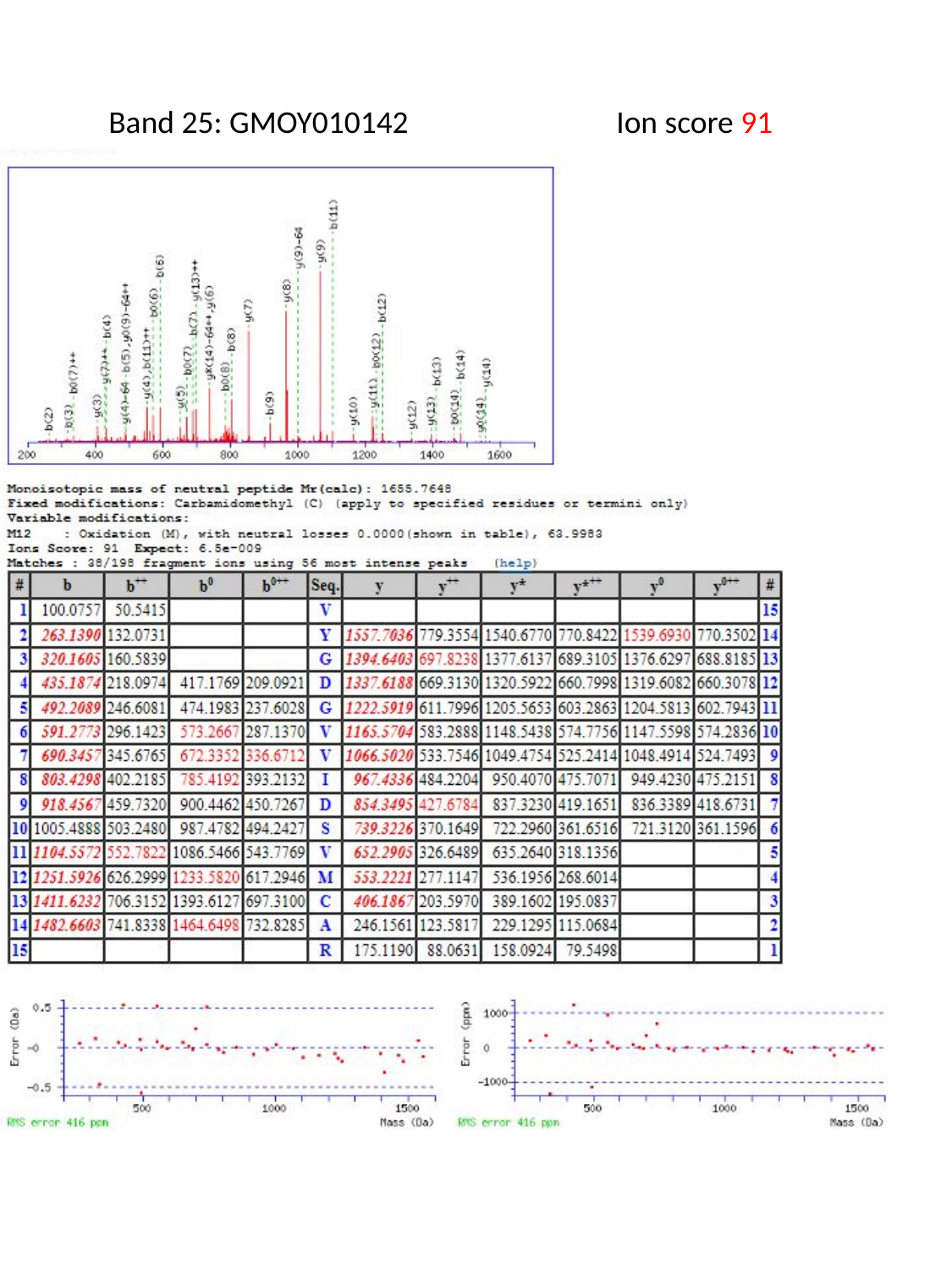

Band 25: GMOY010142 		Ion score 91

## Slide 124
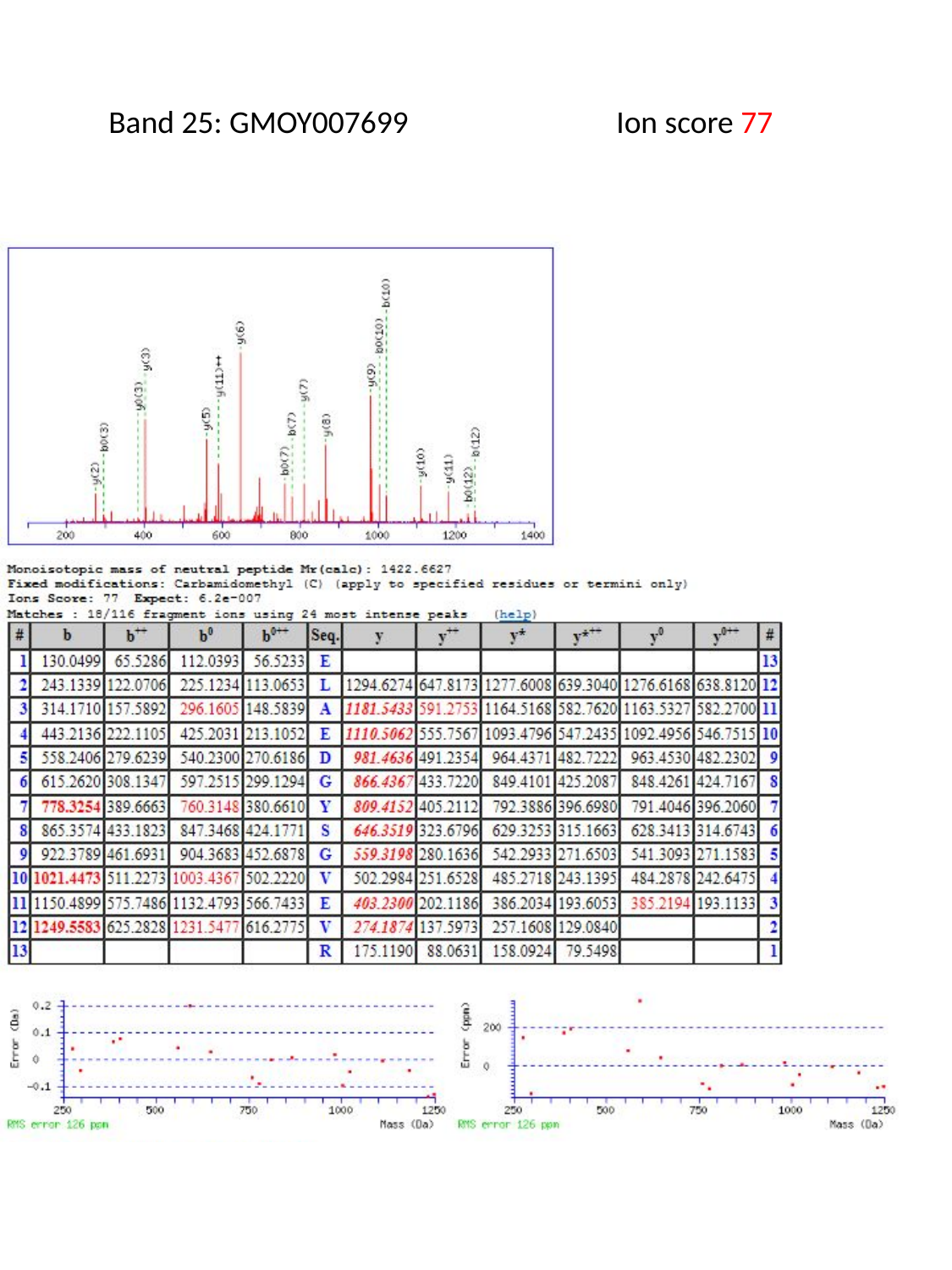

Band 25: GMOY007699 		Ion score 77

## Slide 125
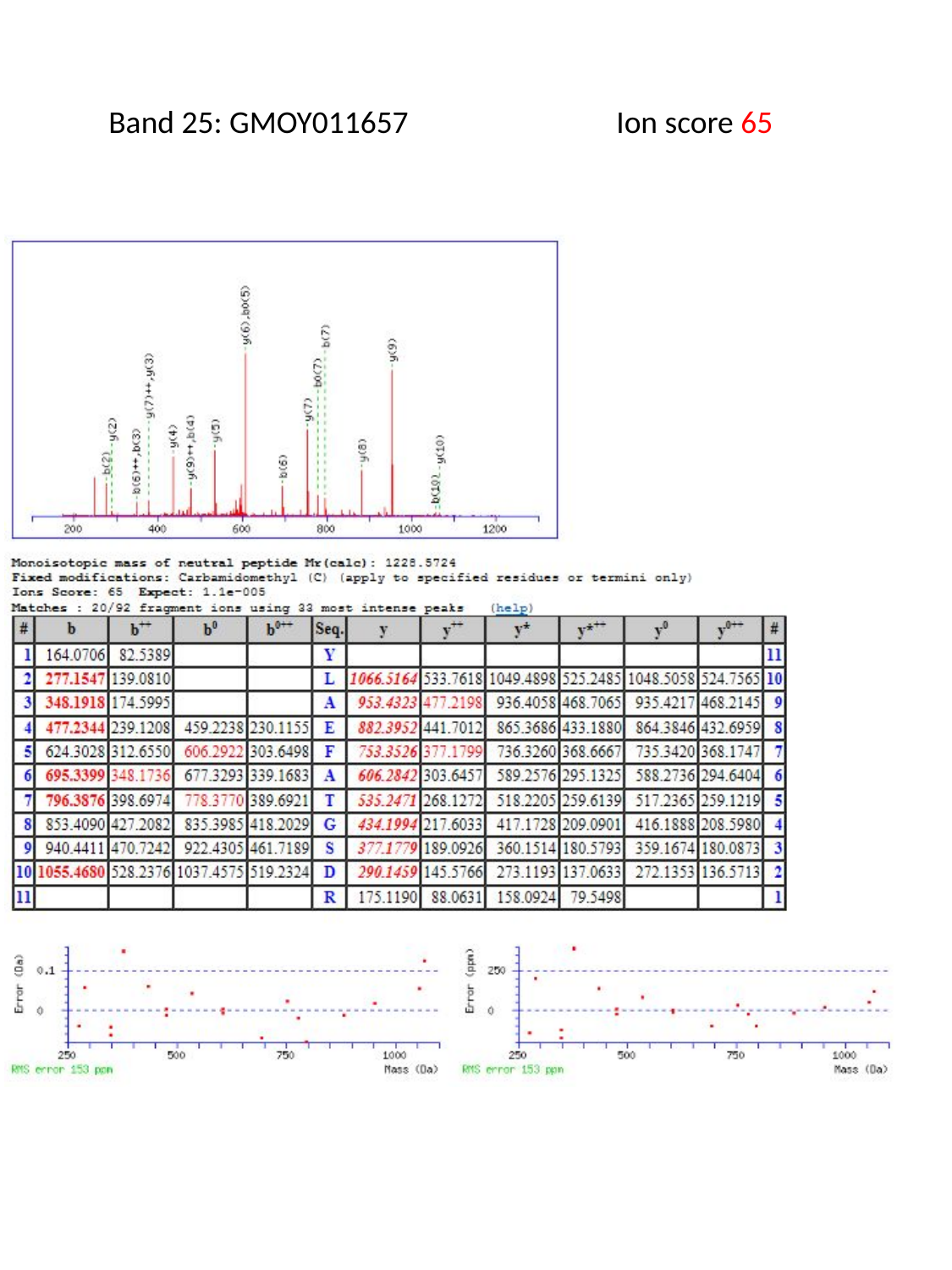

Band 25: GMOY011657 		Ion score 65

## Slide 126
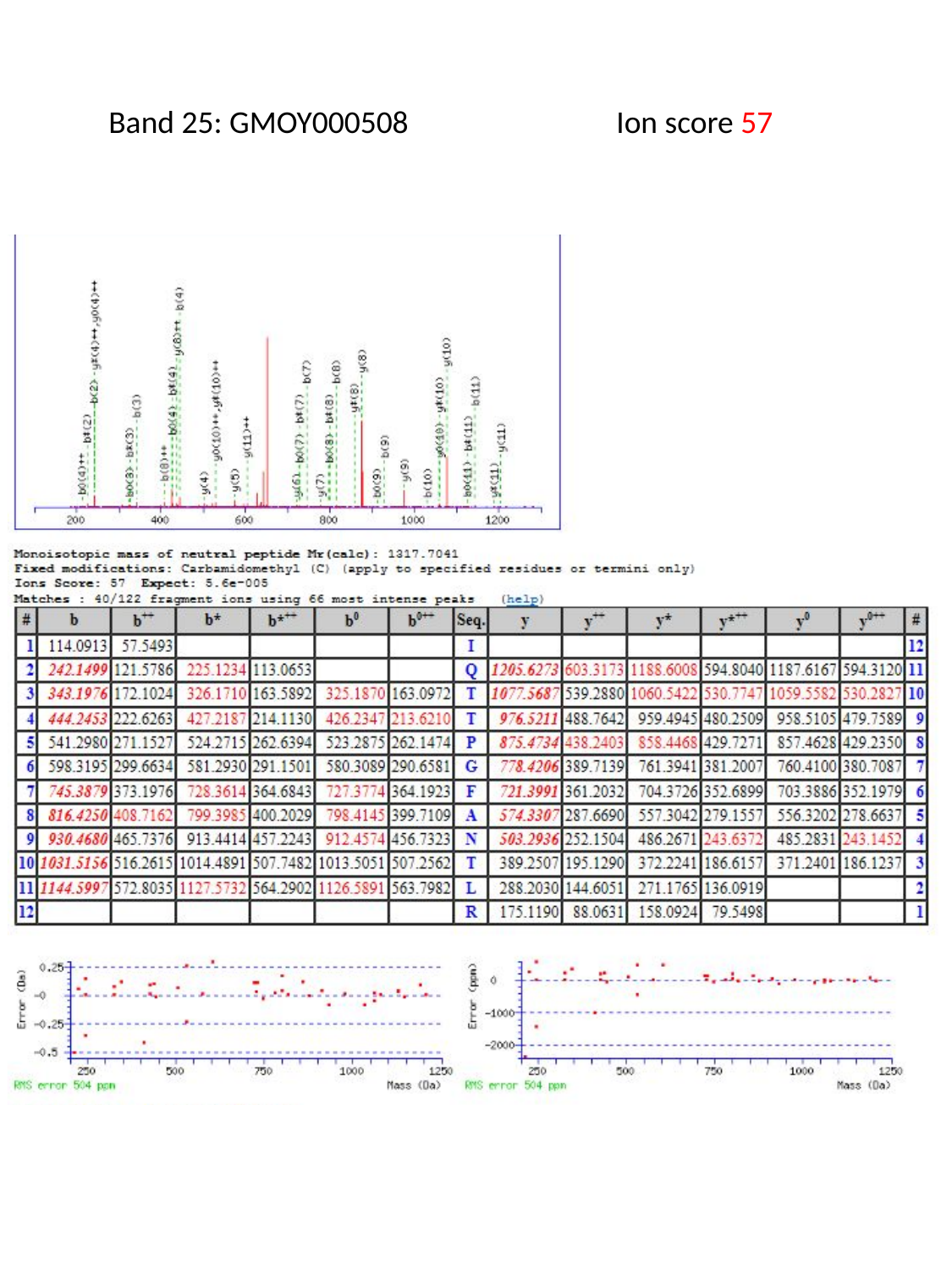

Band 25: GMOY000508 		Ion score 57

## Slide 127
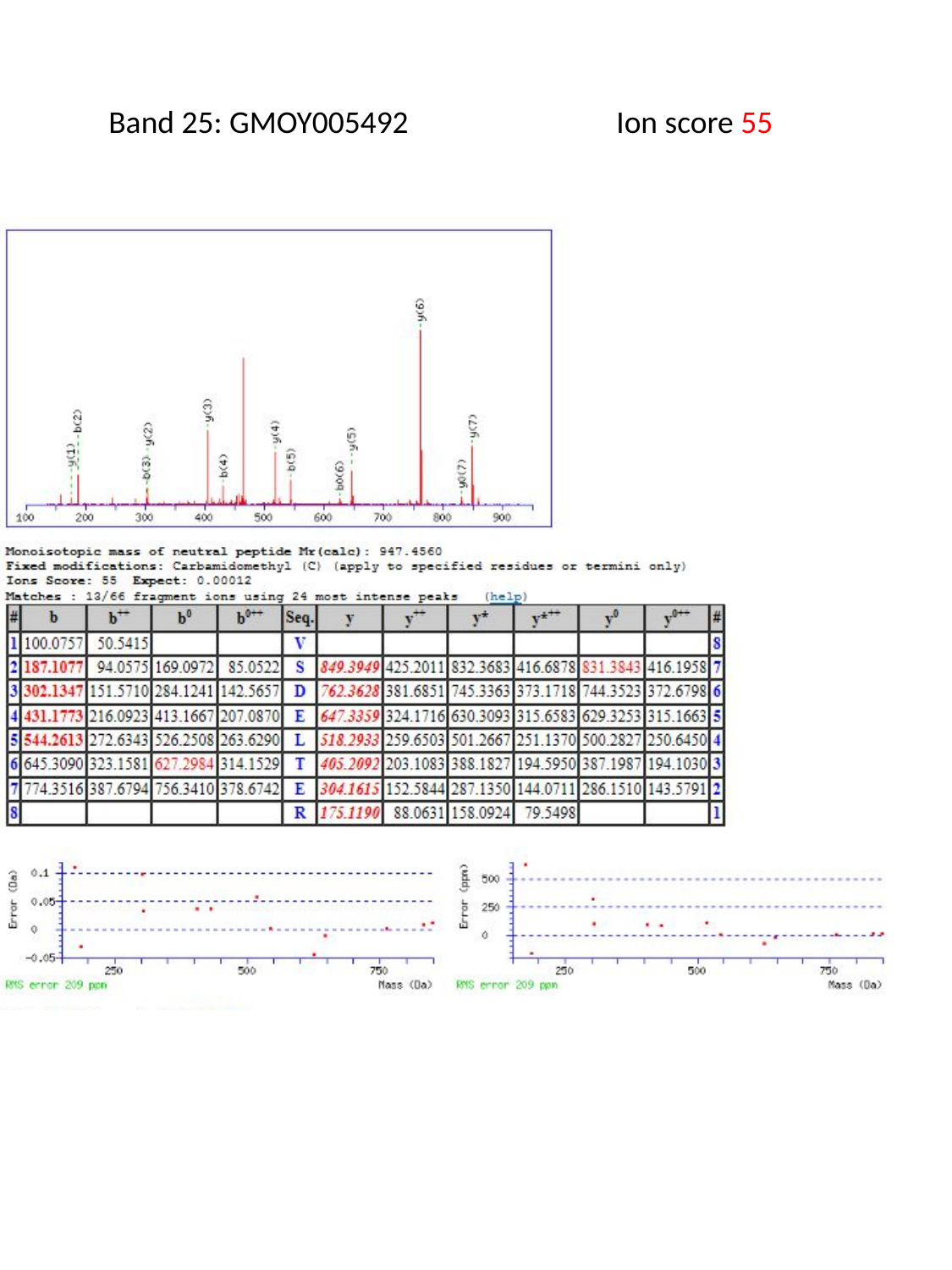

Band 25: GMOY005492 		Ion score 55

## Slide 128
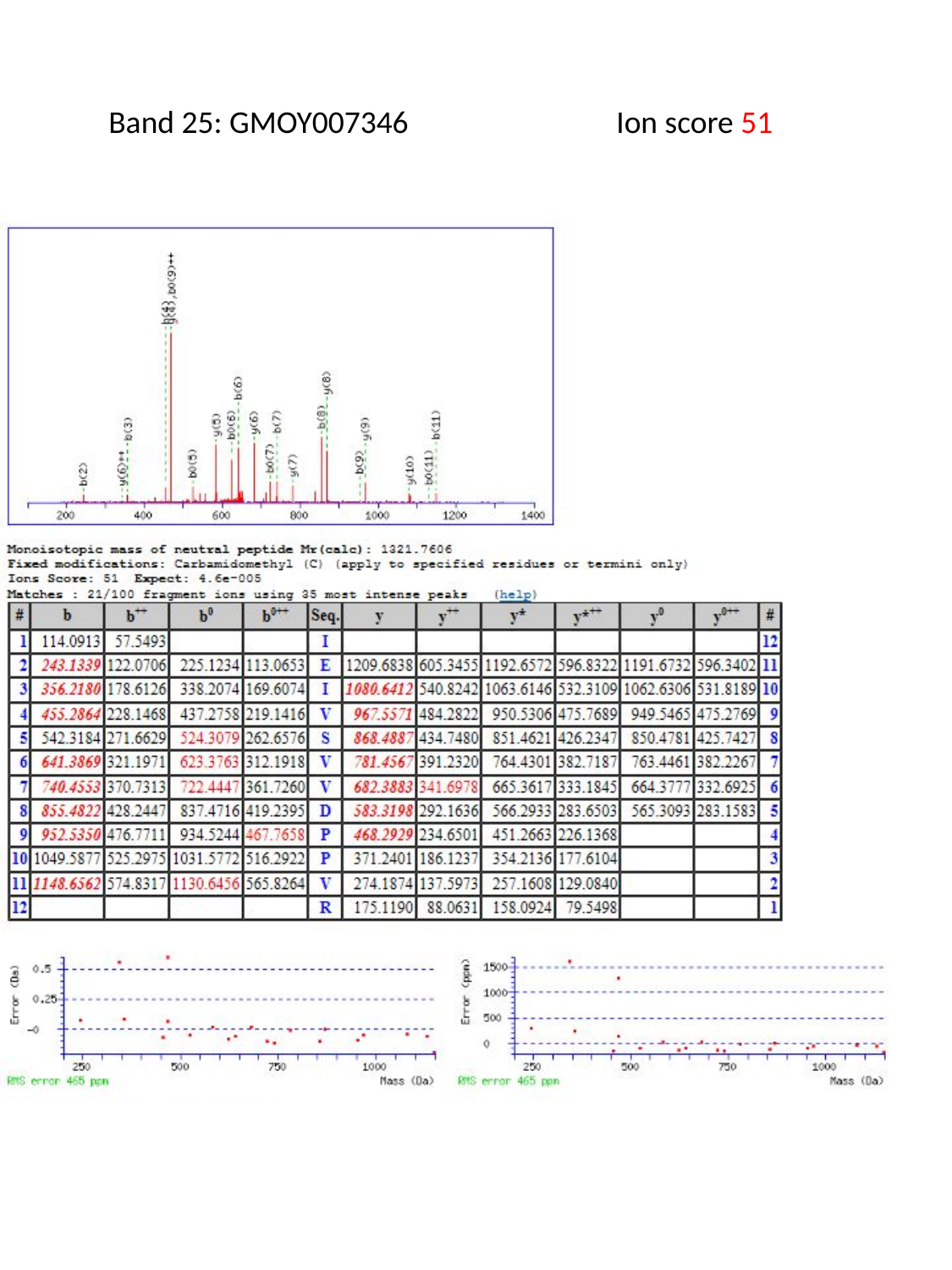

Band 25: GMOY007346 		Ion score 51

## Slide 129
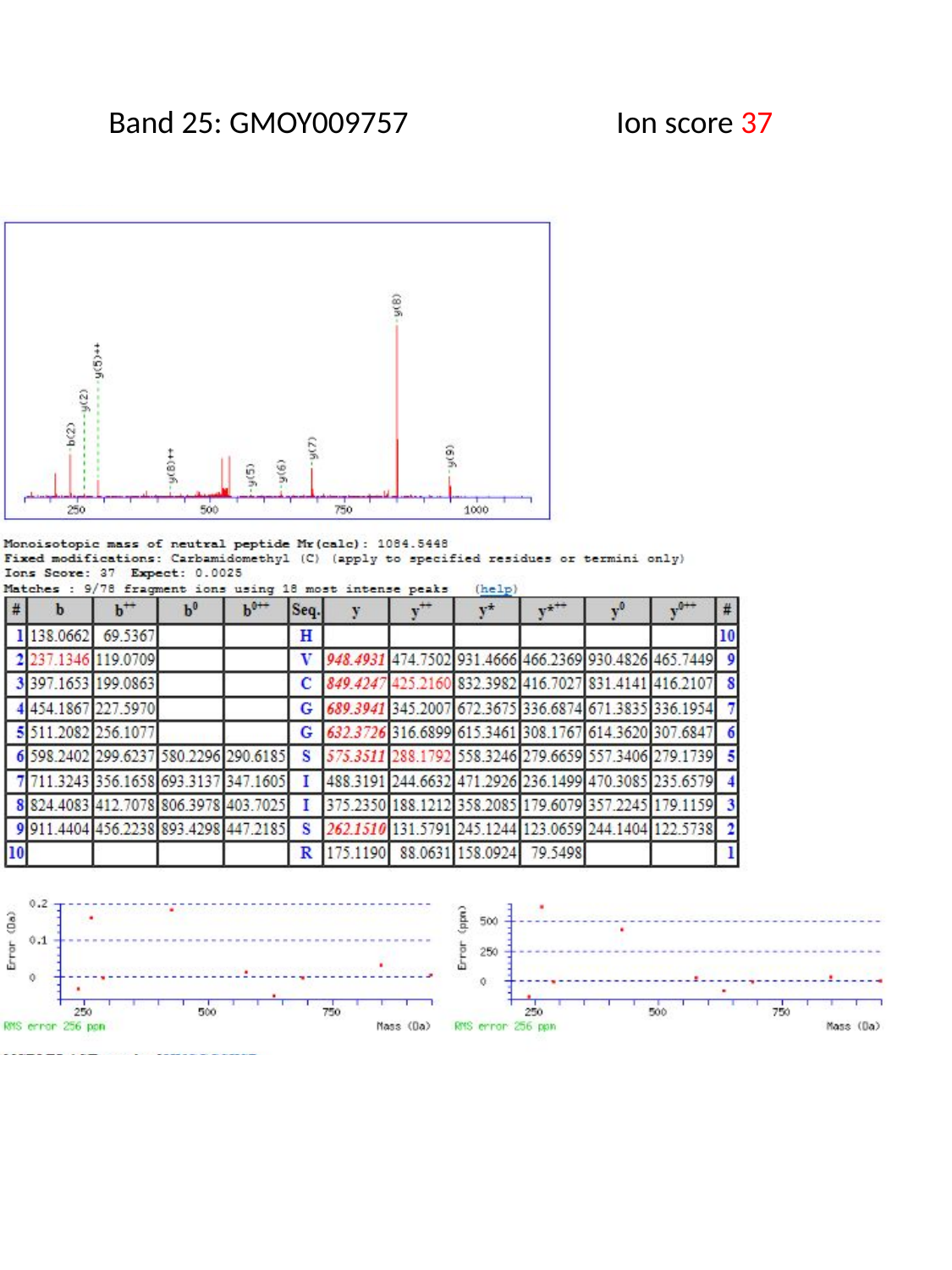

Band 25: GMOY009757 		Ion score 37

## Slide 130
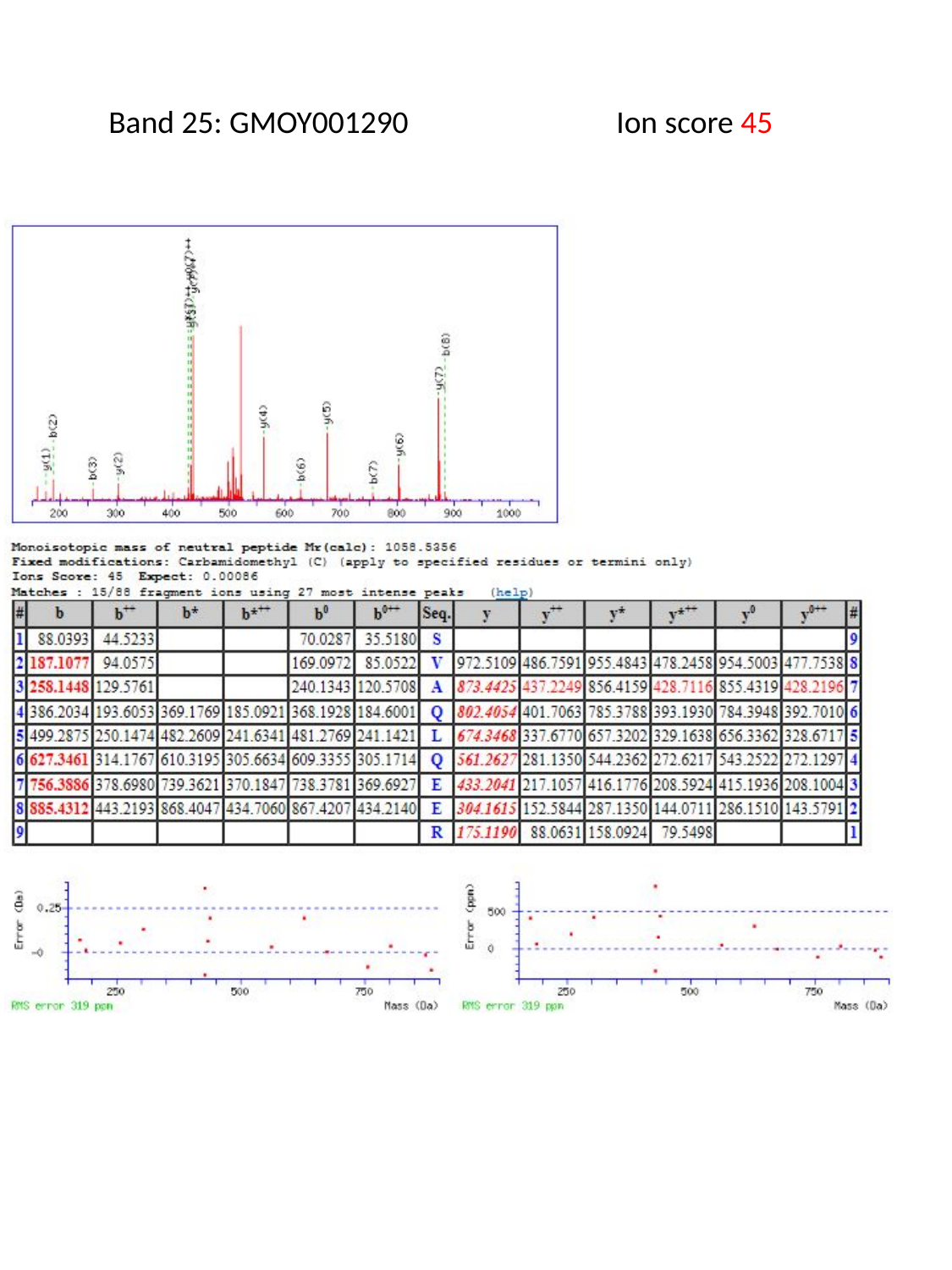

Band 25: GMOY001290 		Ion score 45

## Slide 131
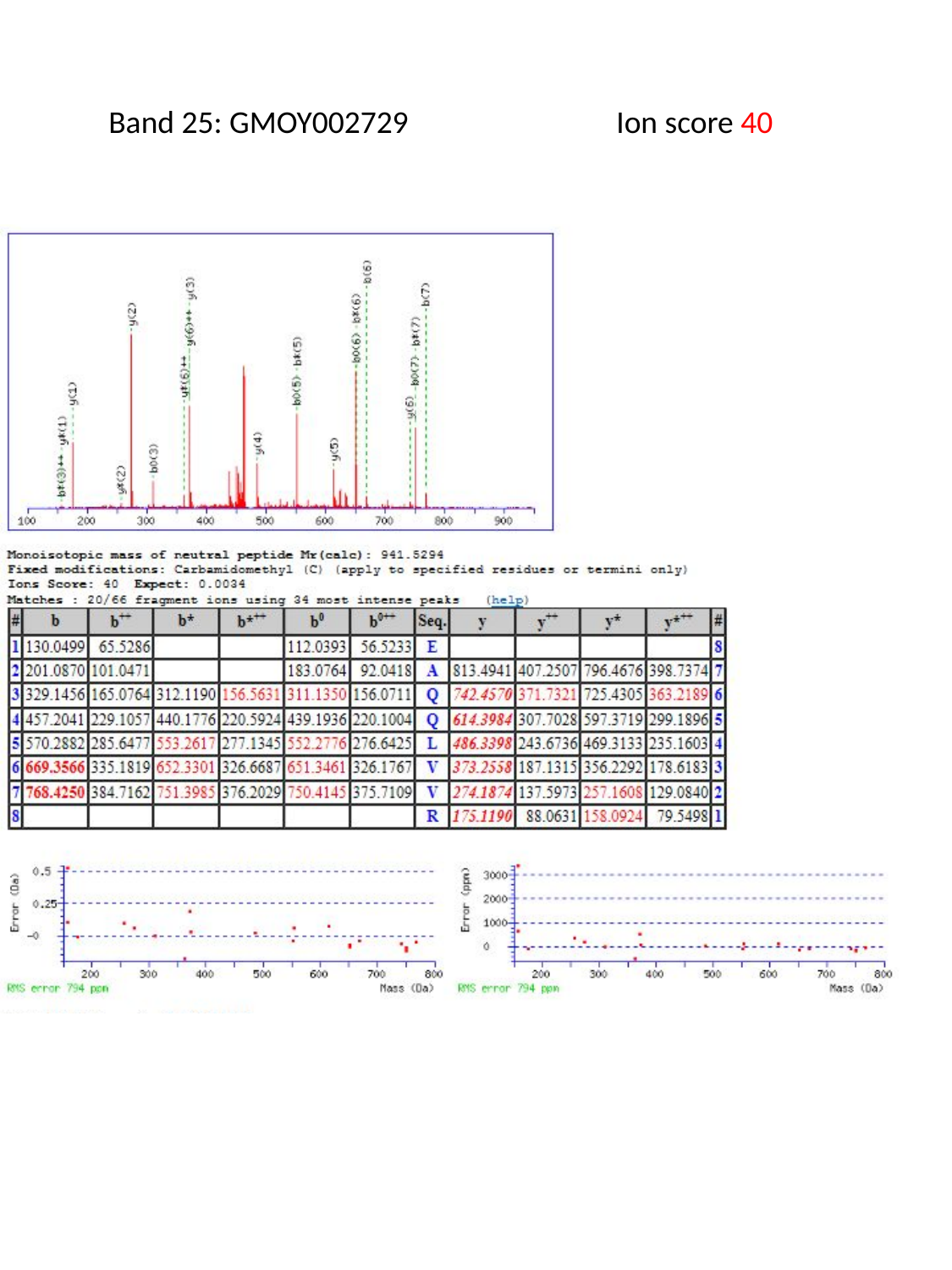

Band 25: GMOY002729 		Ion score 40

## Slide 132
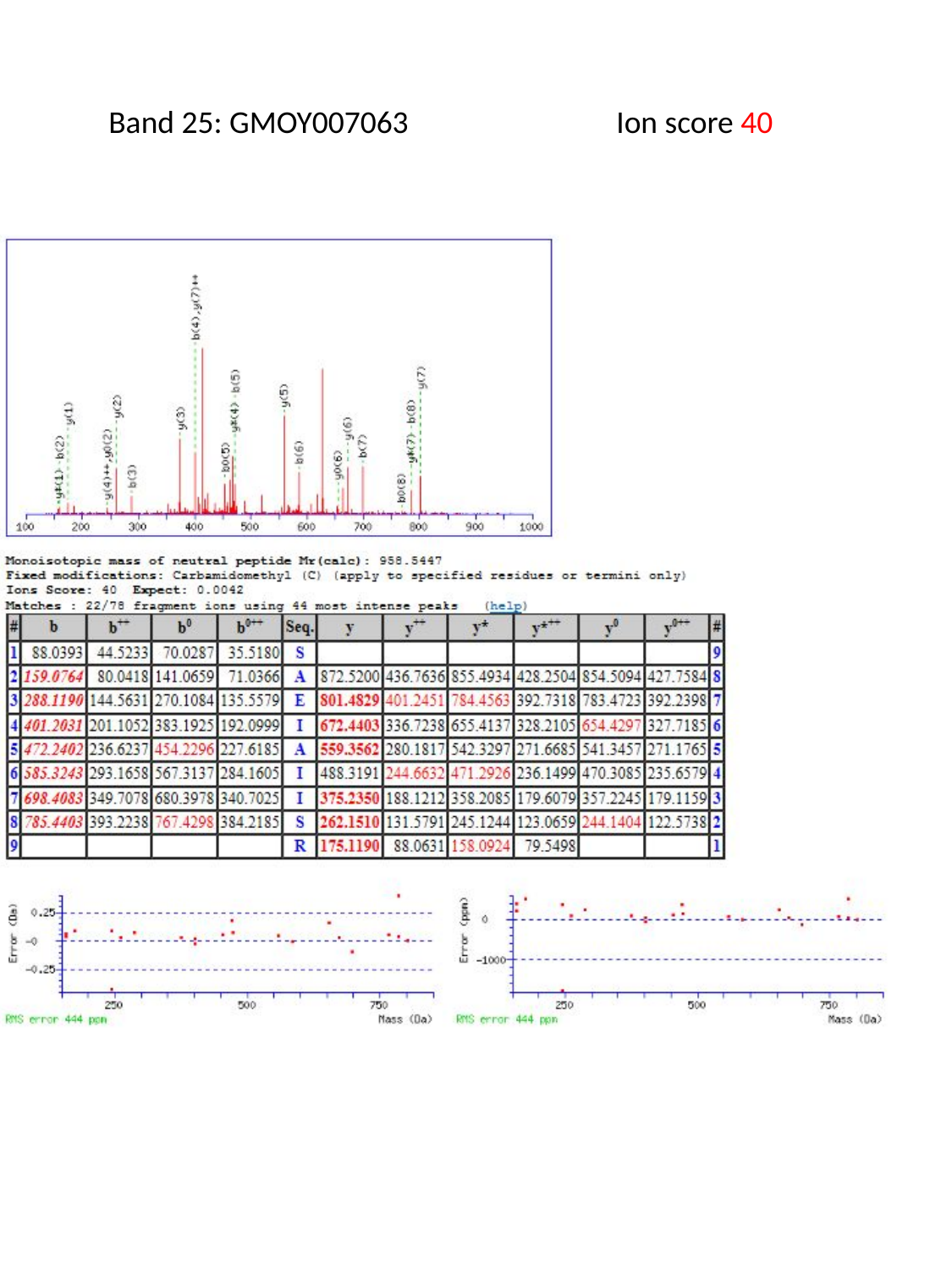

Band 25: GMOY007063 		Ion score 40

## Slide 133
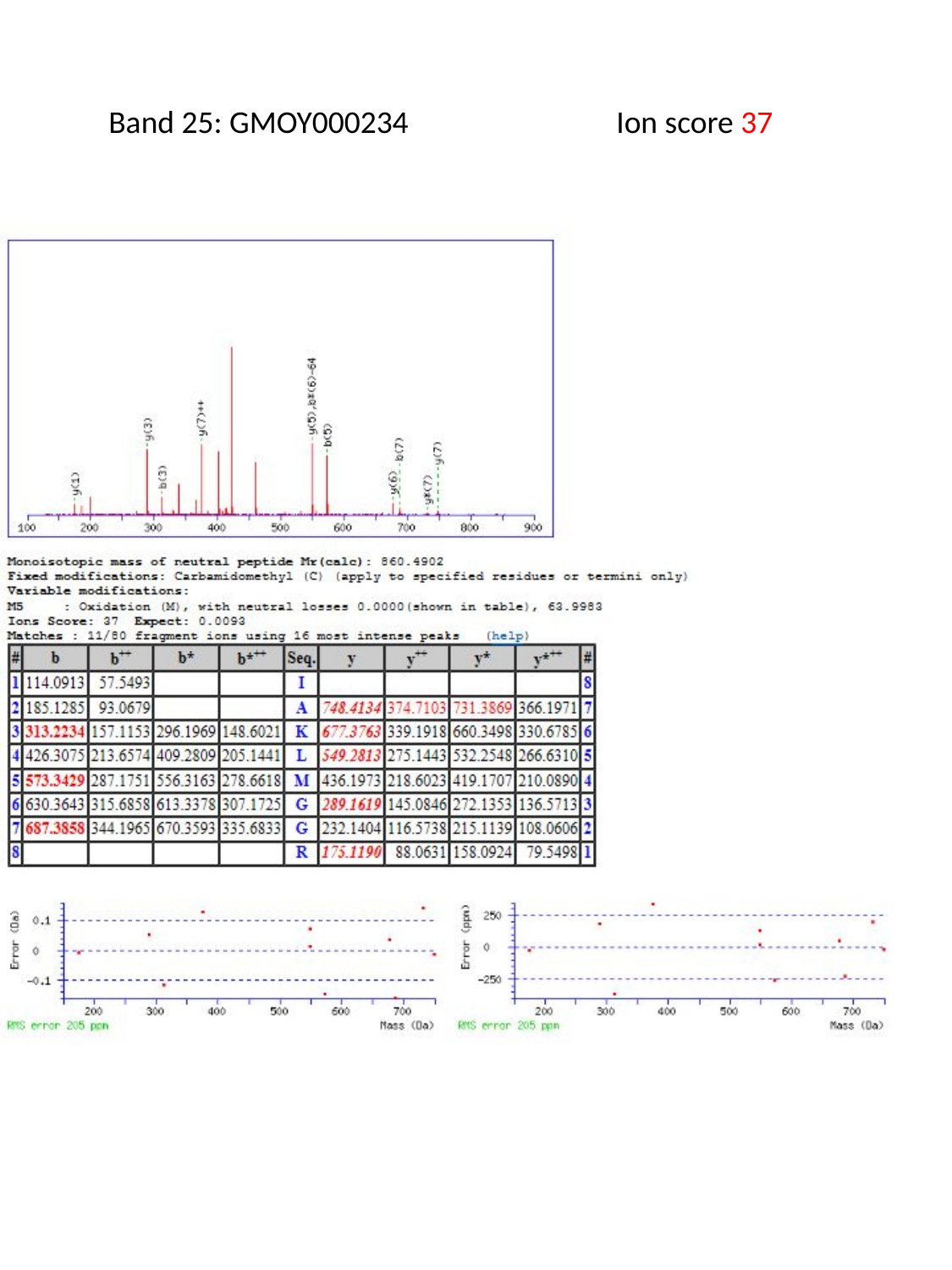

Band 25: GMOY000234 		Ion score 37

## Slide 134
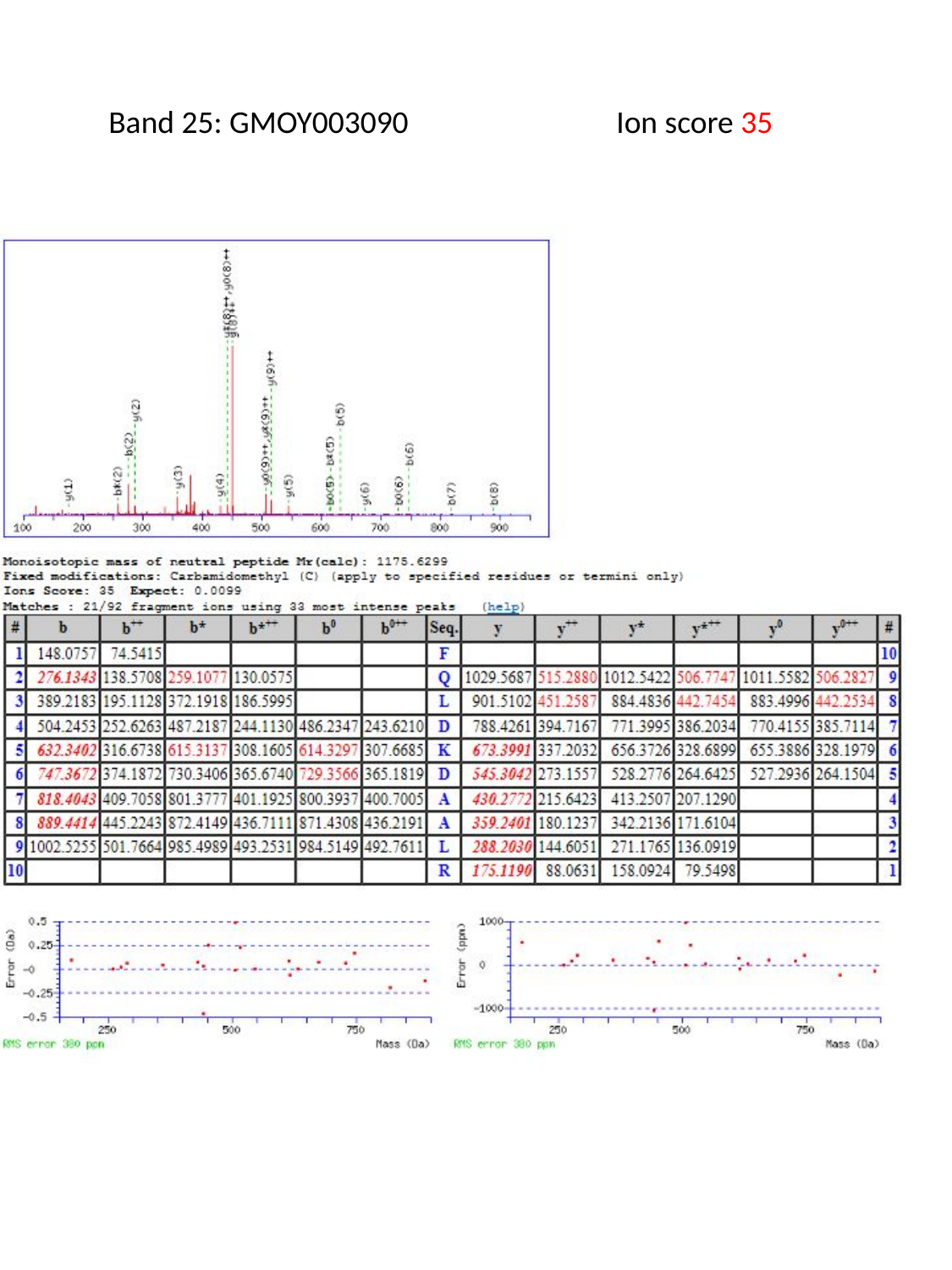

Band 25: GMOY003090 		Ion score 35

## Slide 135
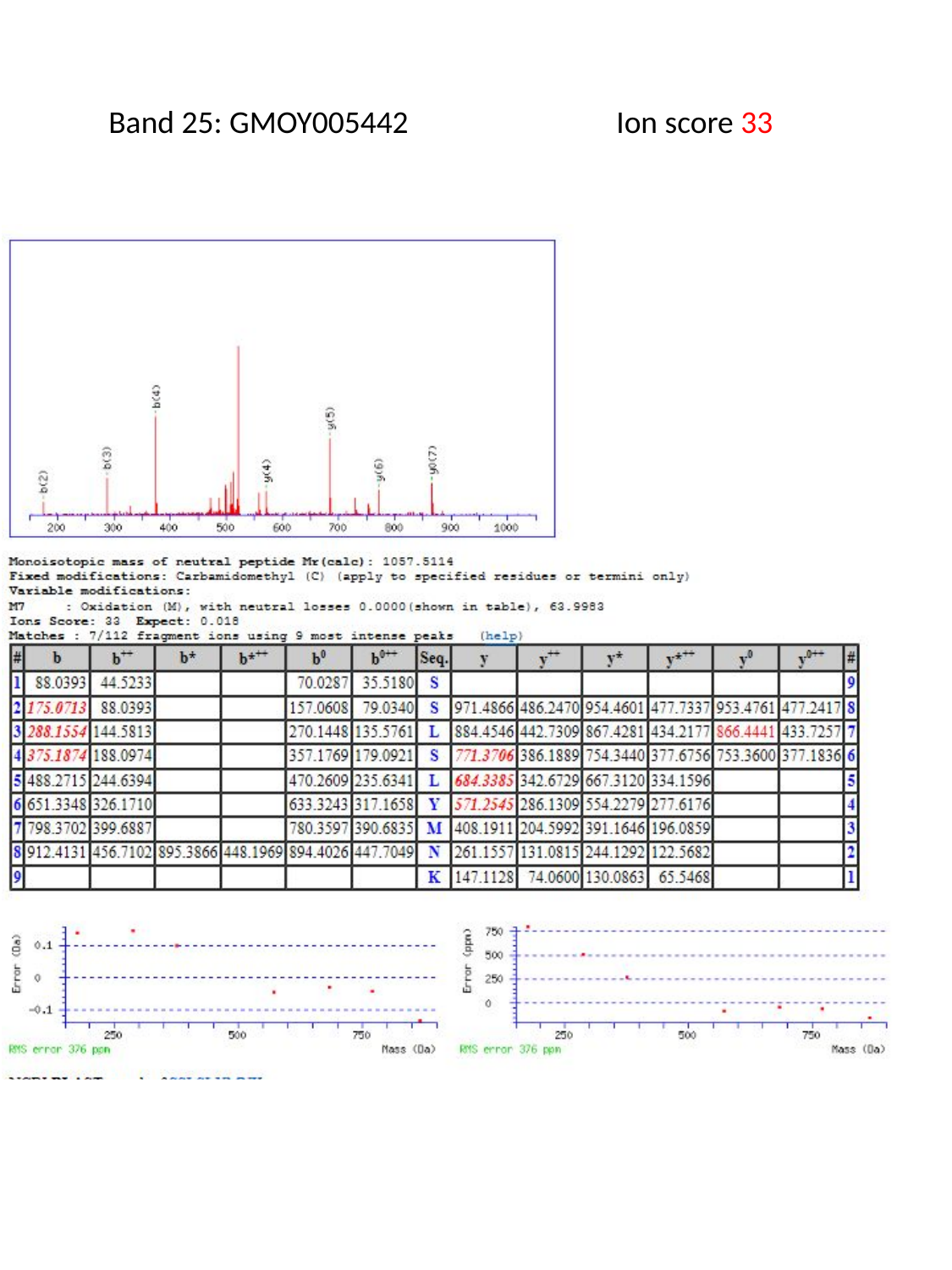

Band 25: GMOY005442 		Ion score 33

## Slide 136
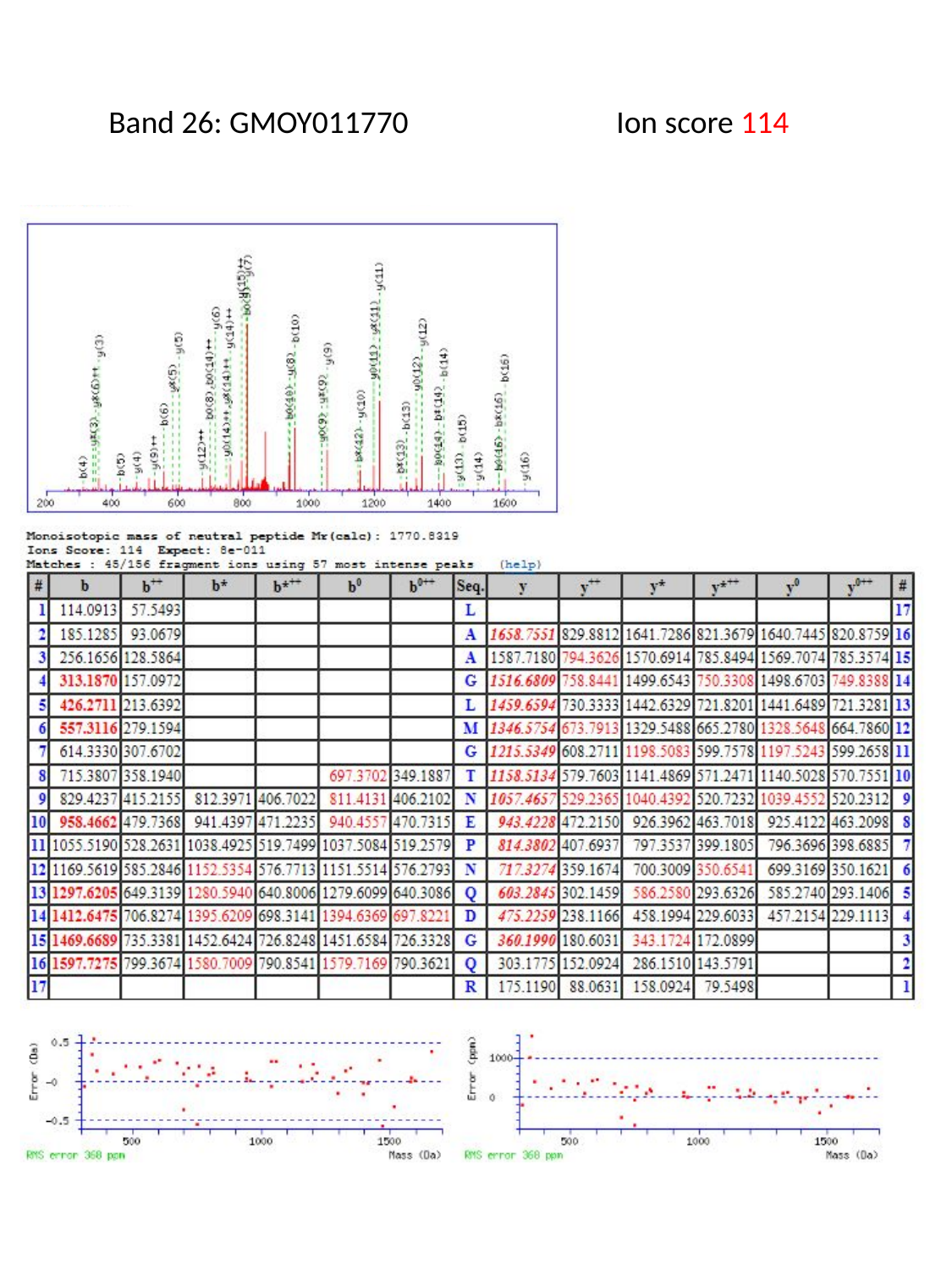

Band 26: GMOY011770 		Ion score 114

## Slide 137
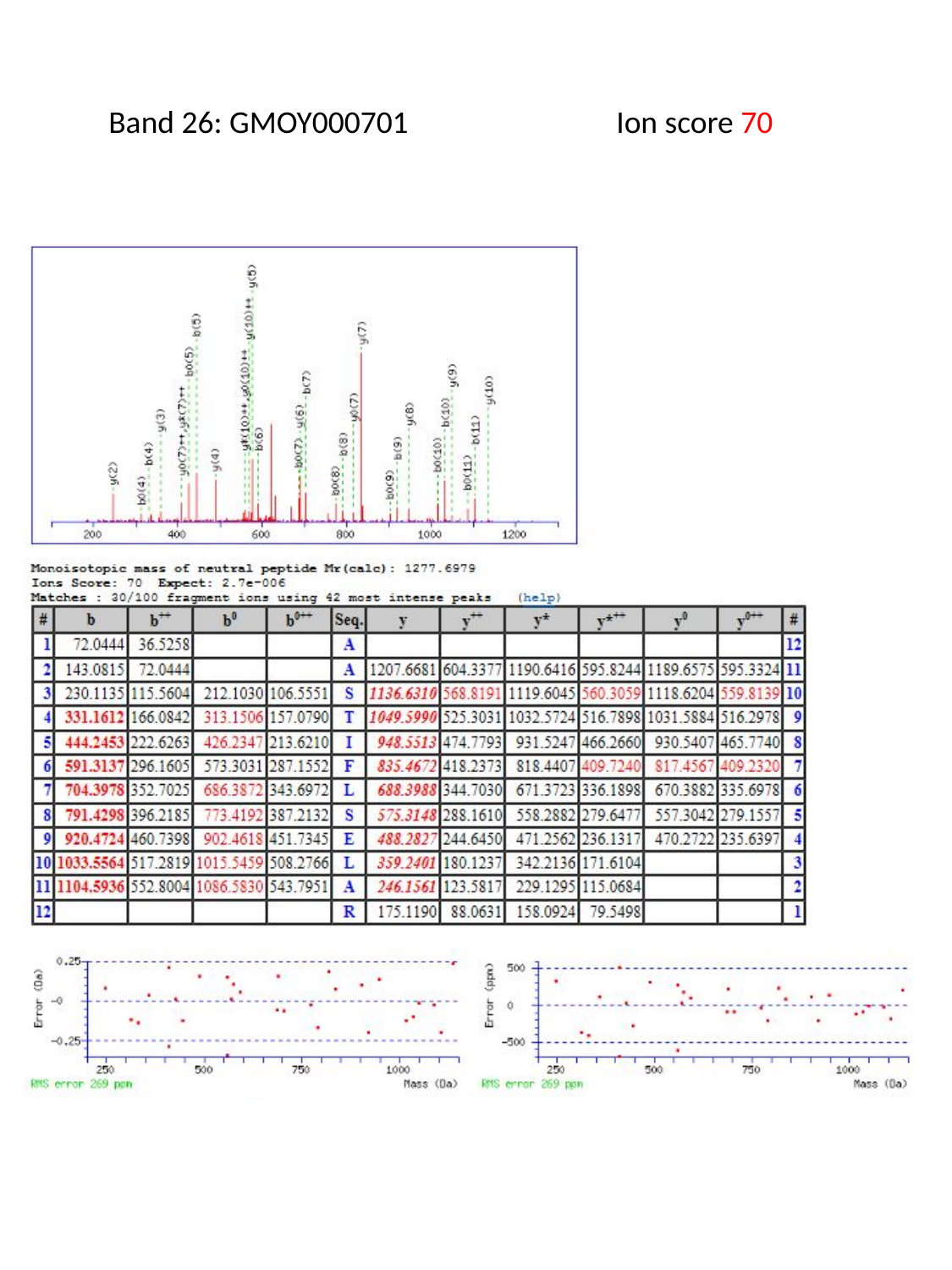

Band 26: GMOY000701 		Ion score 70

## Slide 138
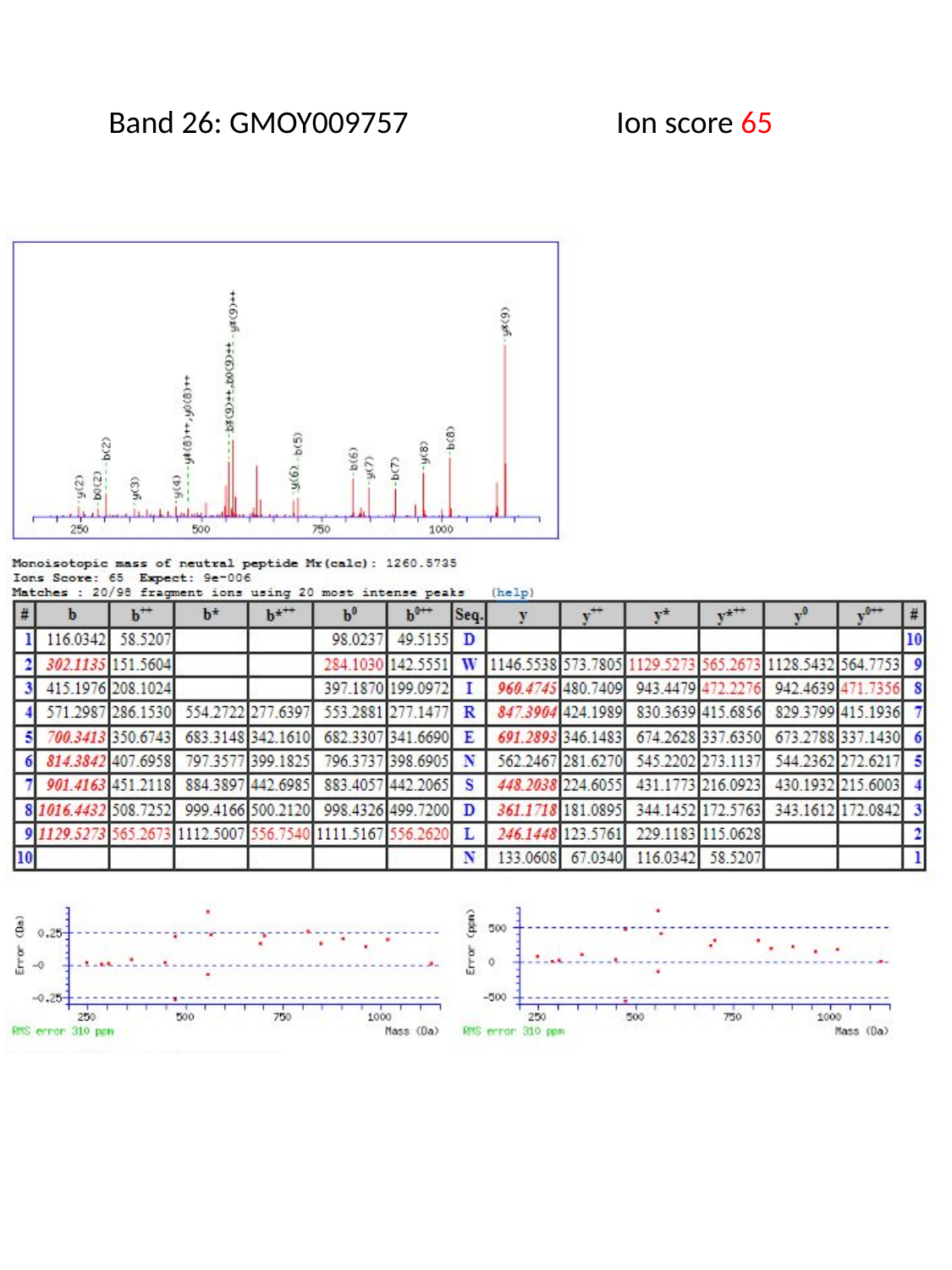

Band 26: GMOY009757 		Ion score 65

## Slide 139
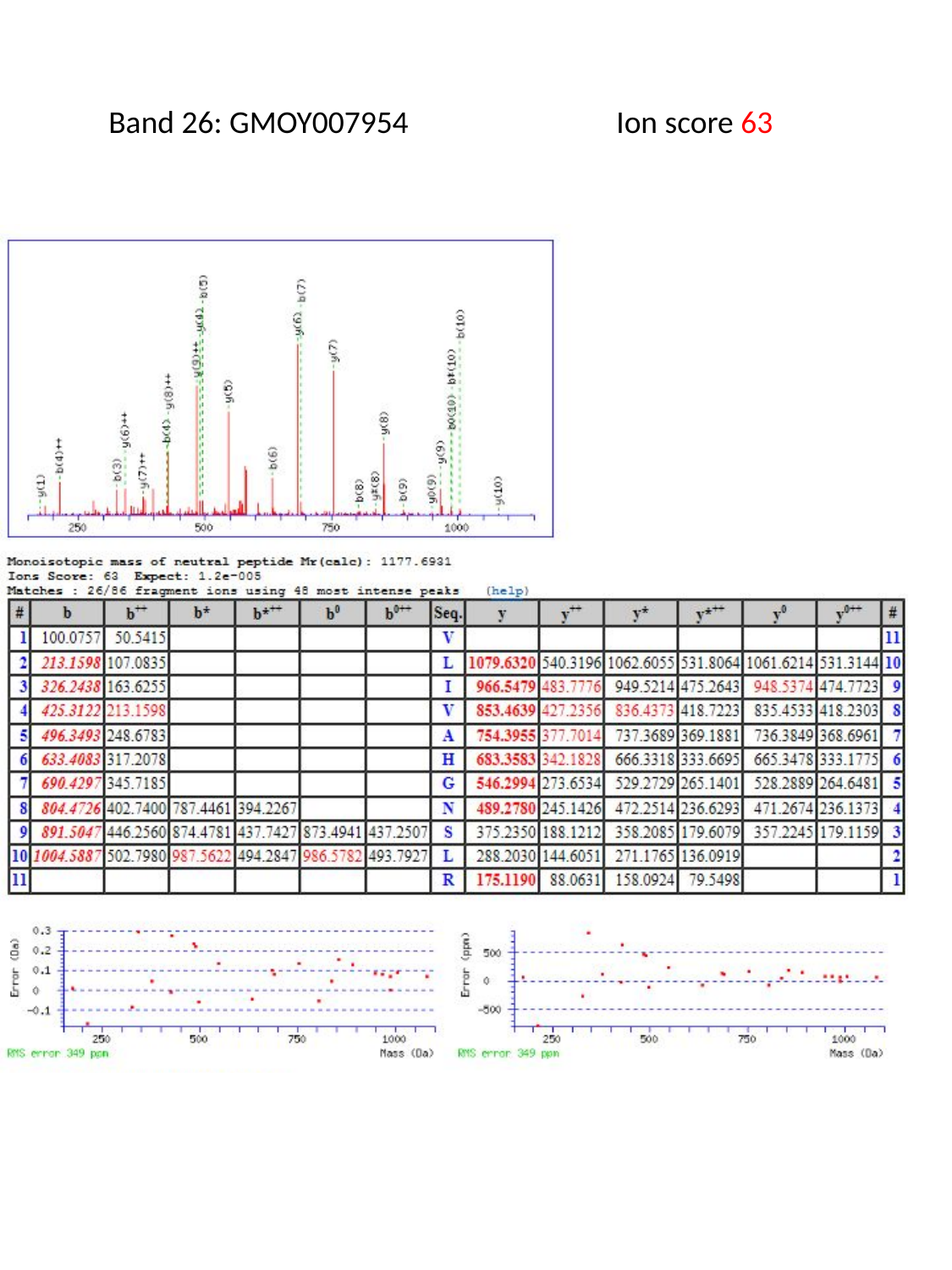

Band 26: GMOY007954 		Ion score 63

## Slide 140
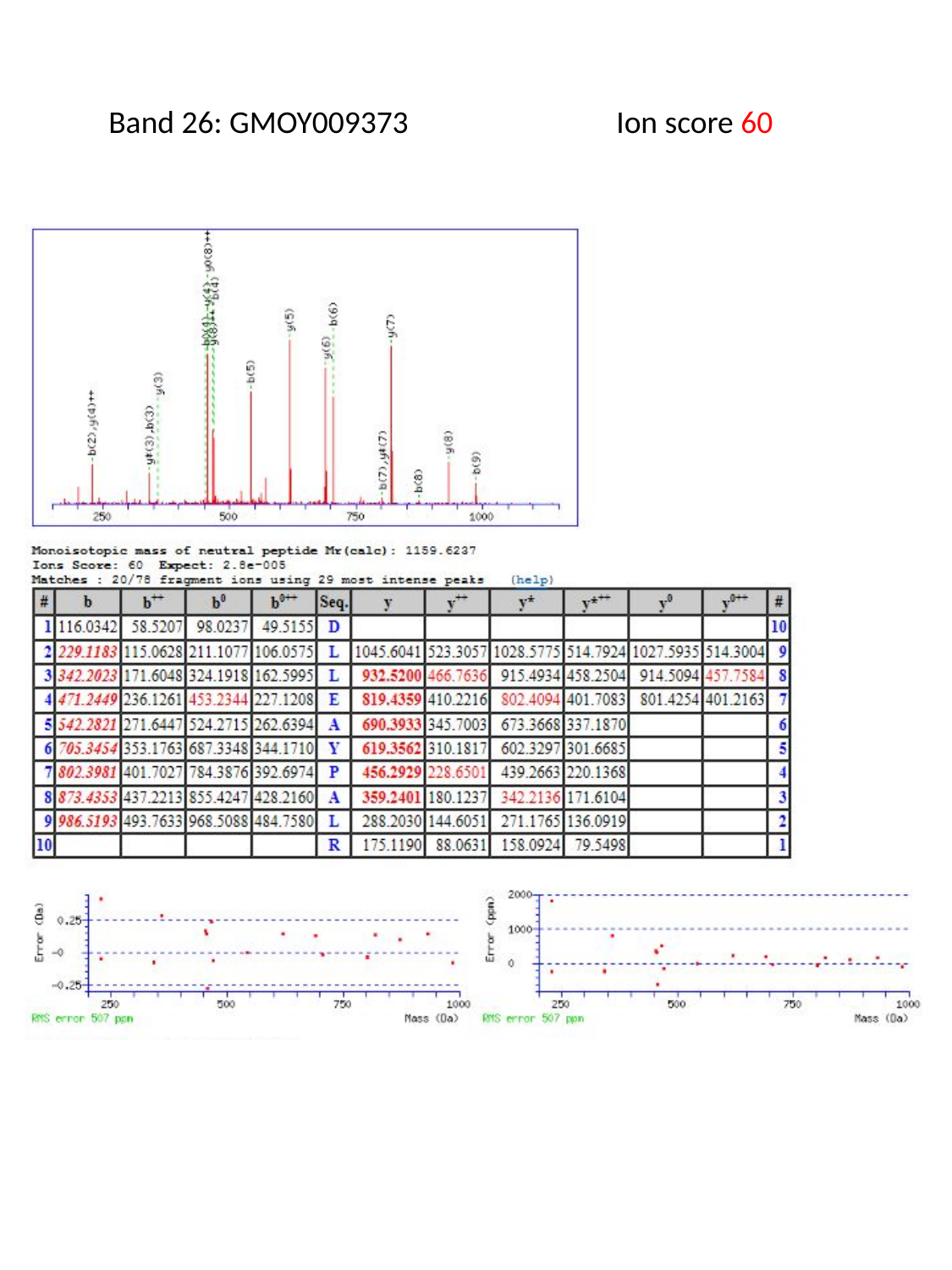

Band 26: GMOY009373 		Ion score 60

## Slide 141
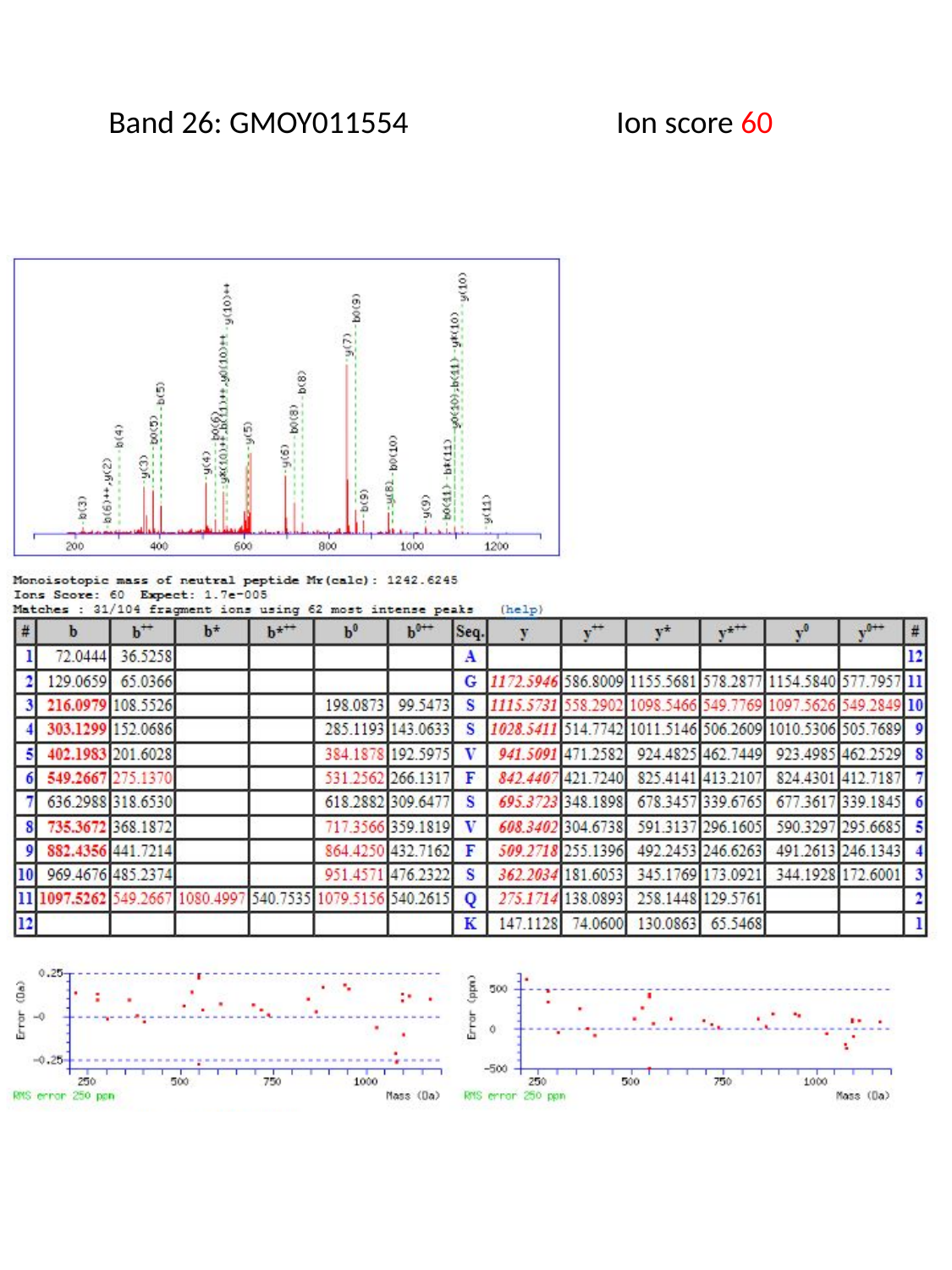

Band 26: GMOY011554 		Ion score 60

## Slide 142
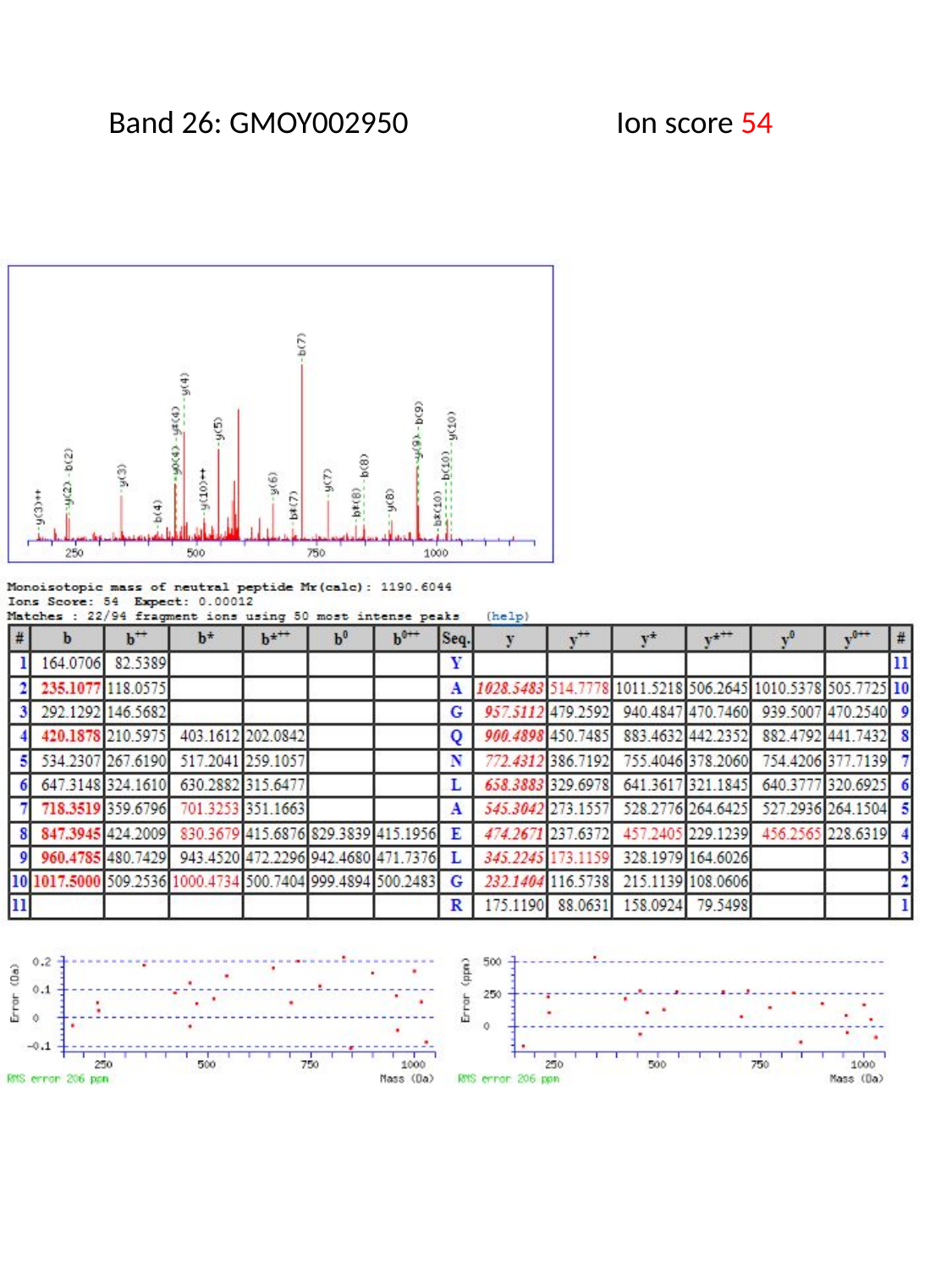

Band 26: GMOY002950 		Ion score 54

## Slide 143
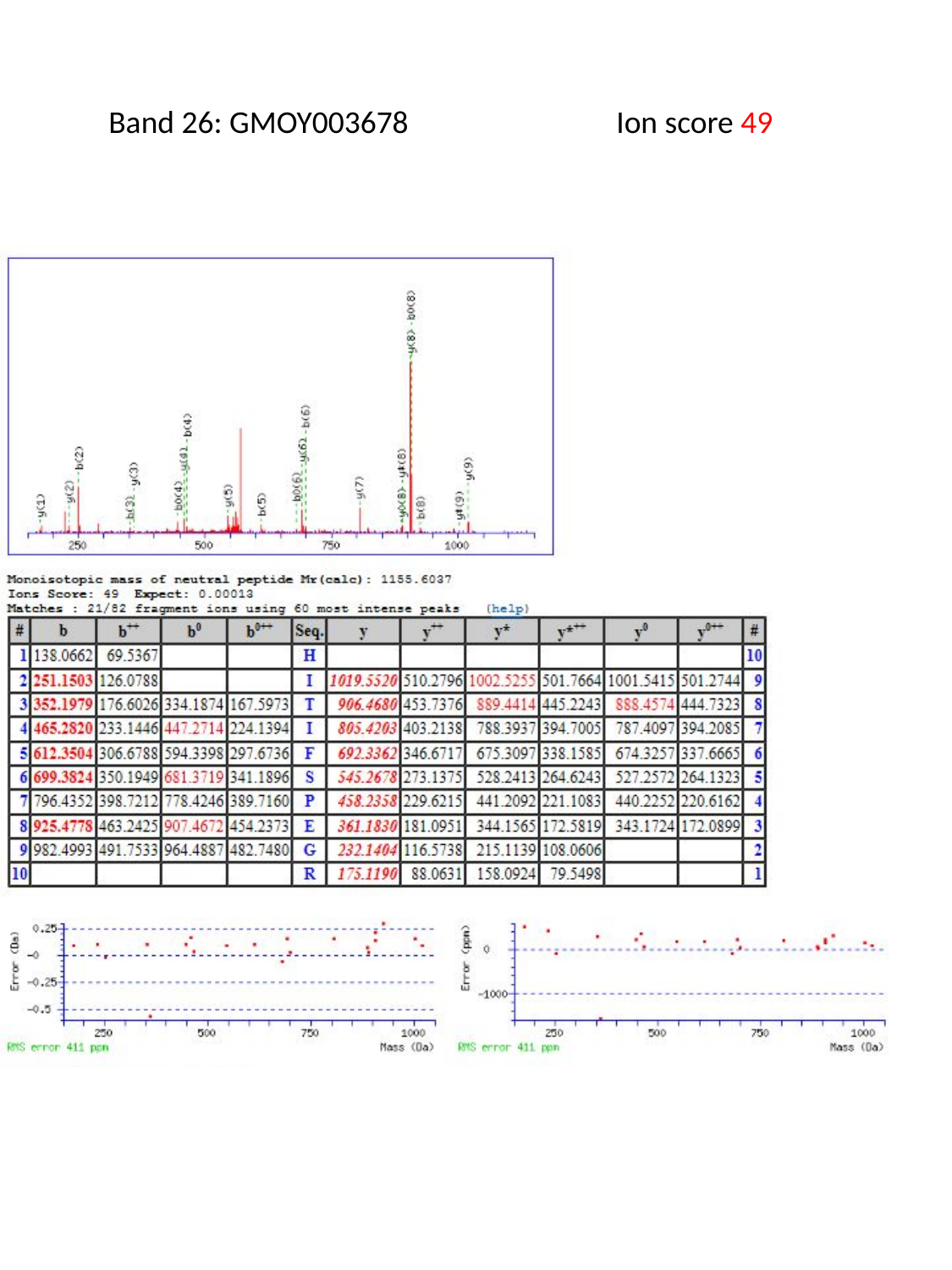

Band 26: GMOY003678 		Ion score 49

## Slide 144
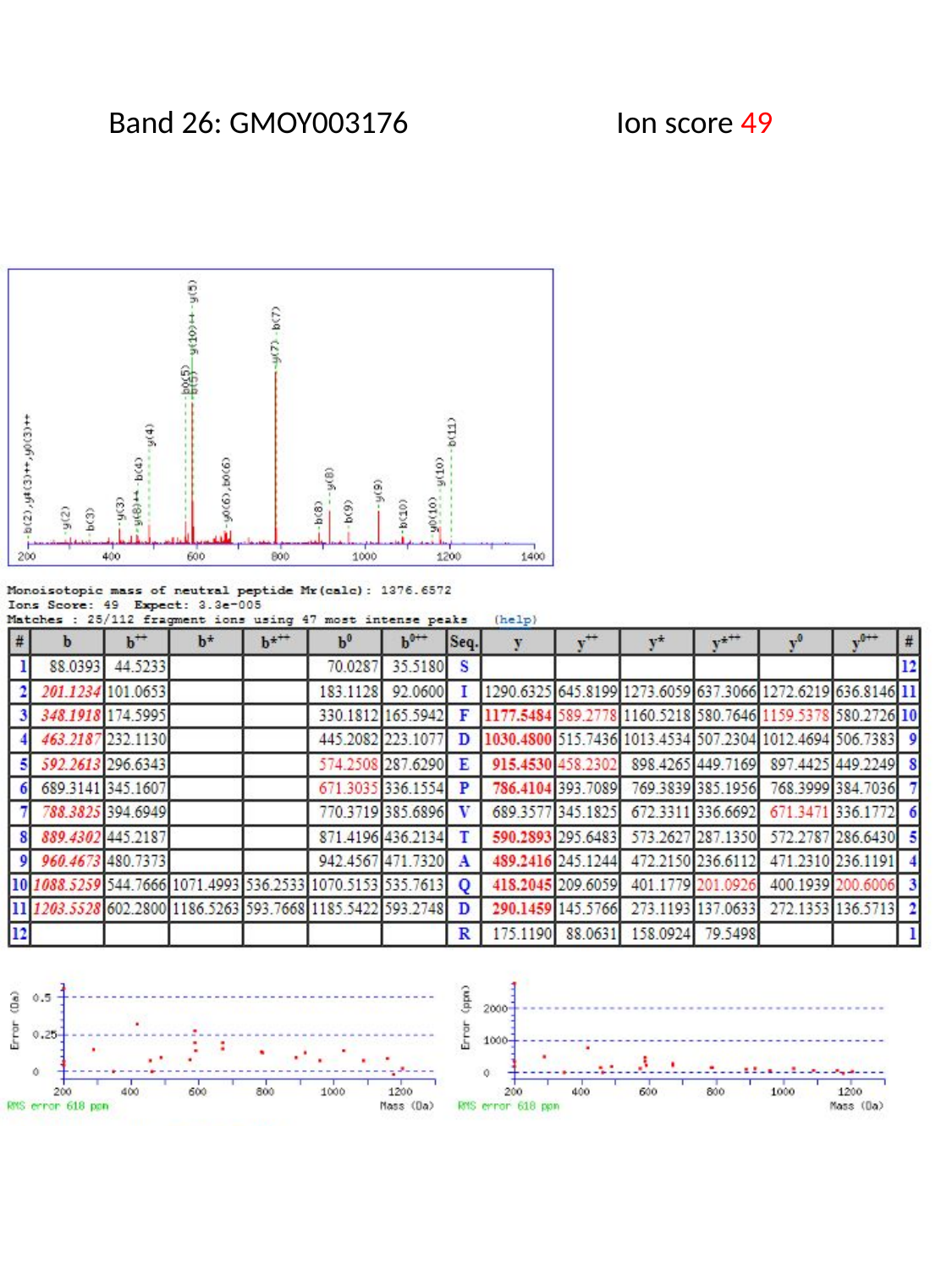

Band 26: GMOY003176 		Ion score 49

## Slide 145
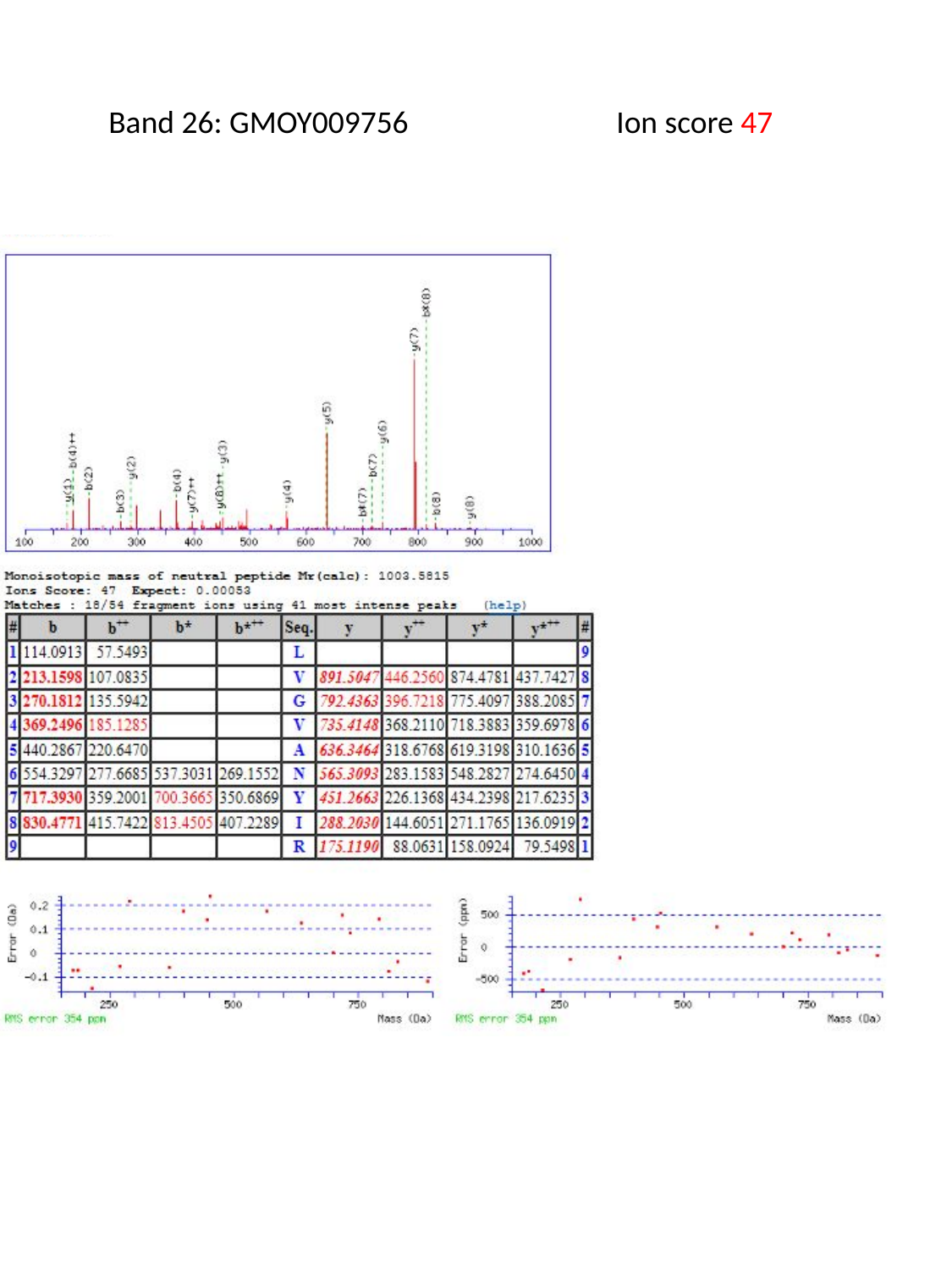

Band 26: GMOY009756 		Ion score 47

## Slide 146
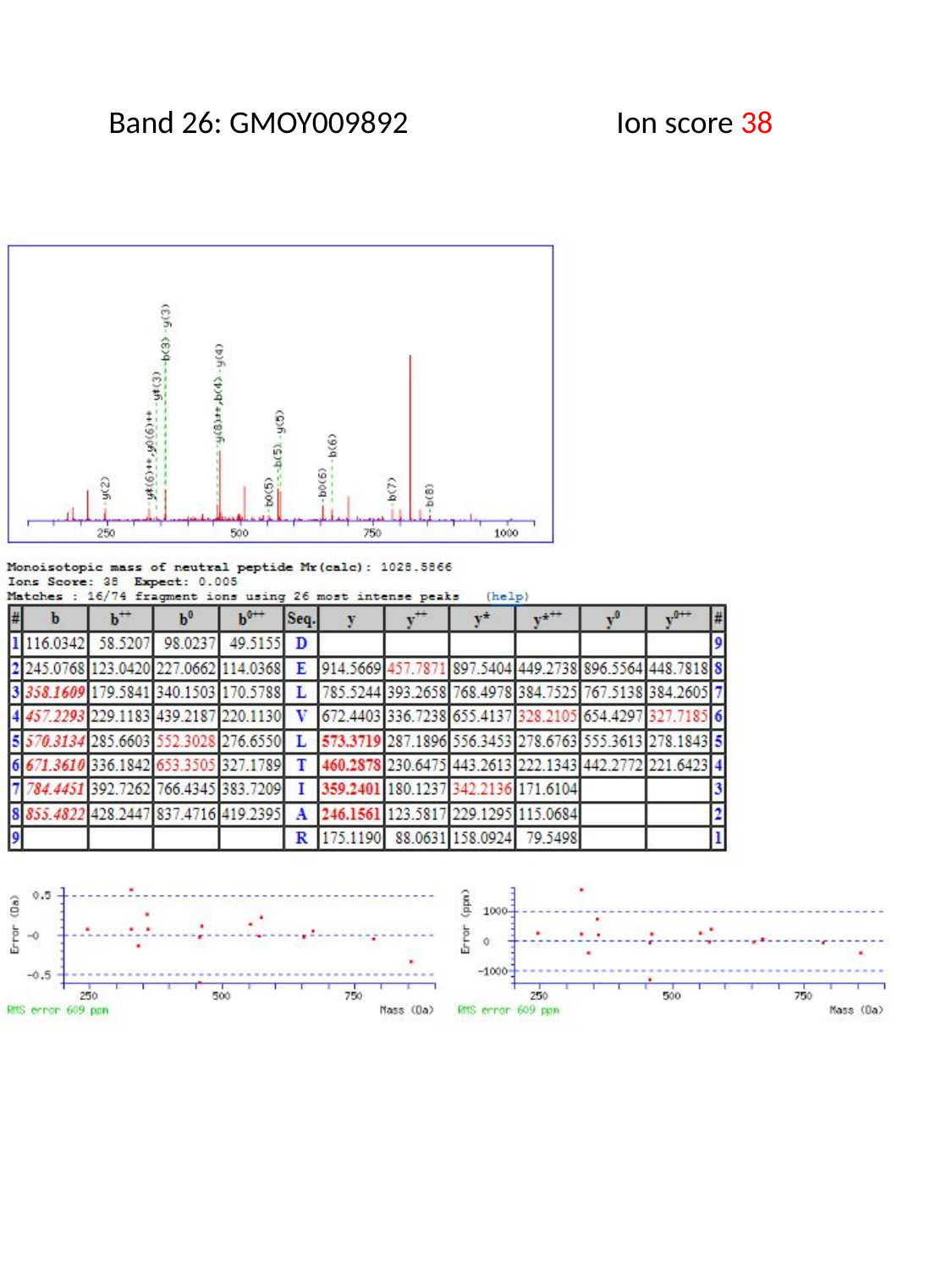

Band 26: GMOY009892 		Ion score 38

## Slide 147
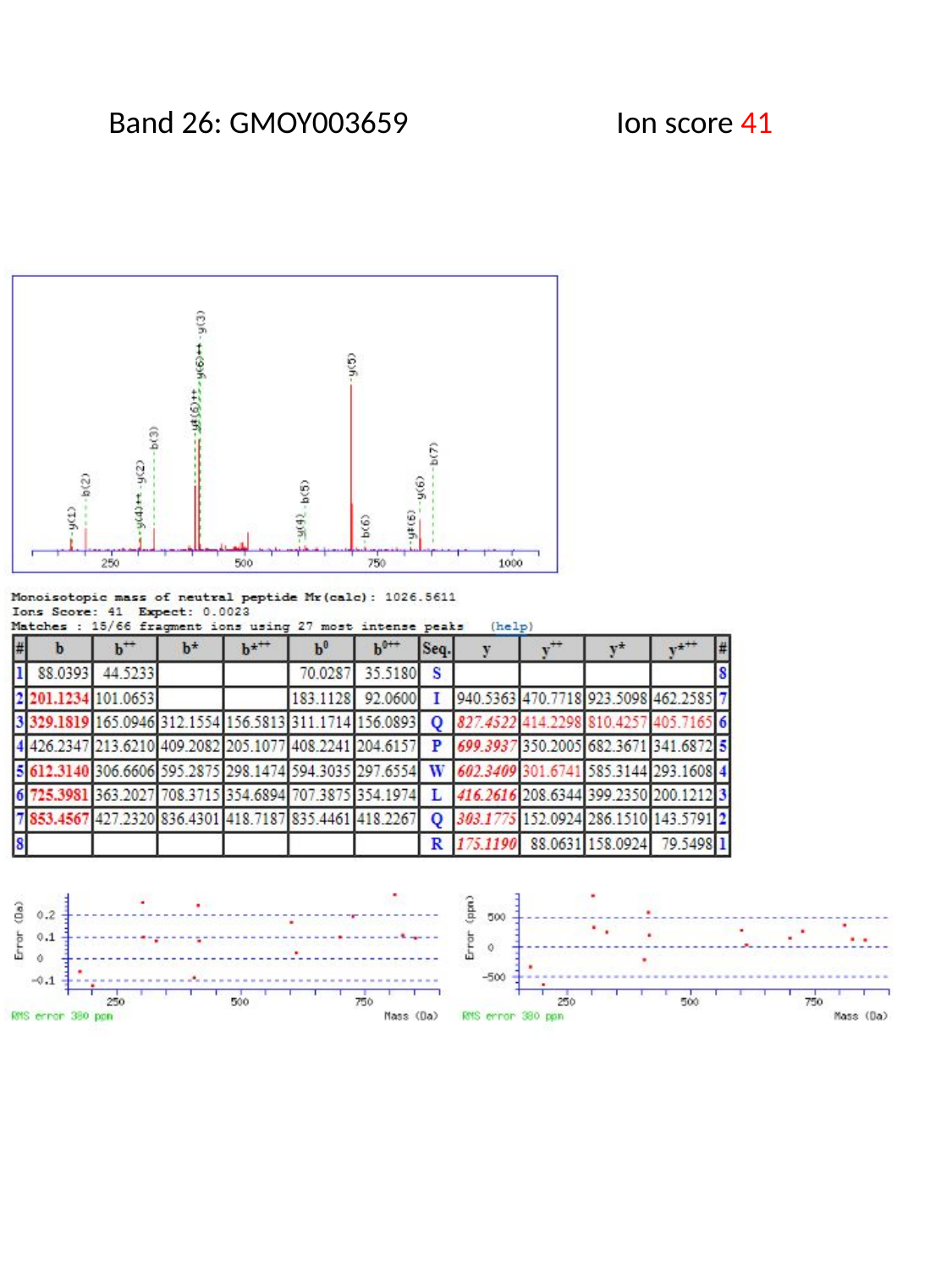

Band 26: GMOY003659 		Ion score 41

## Slide 148
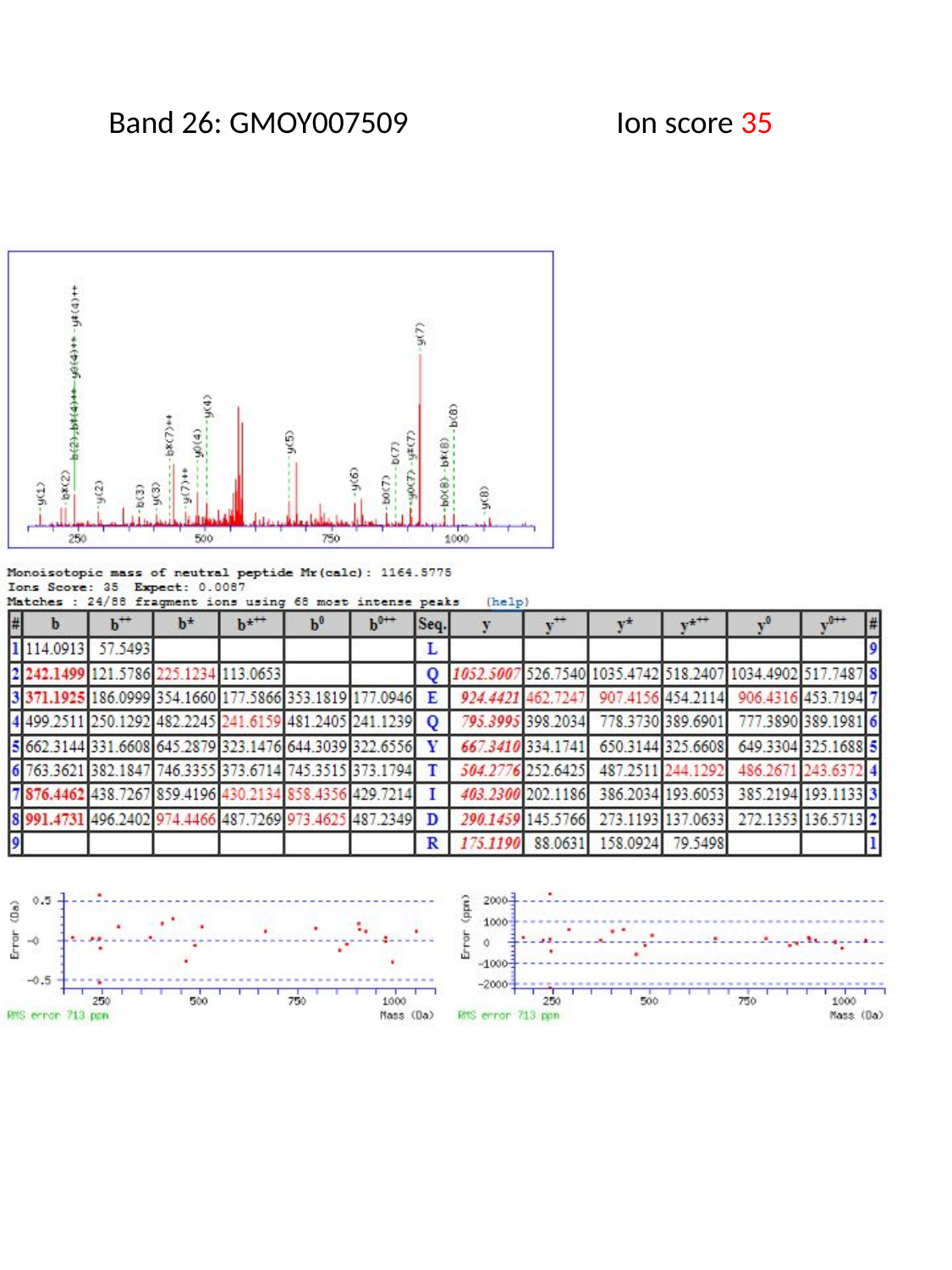

Band 26: GMOY007509 		Ion score 35

## Slide 149
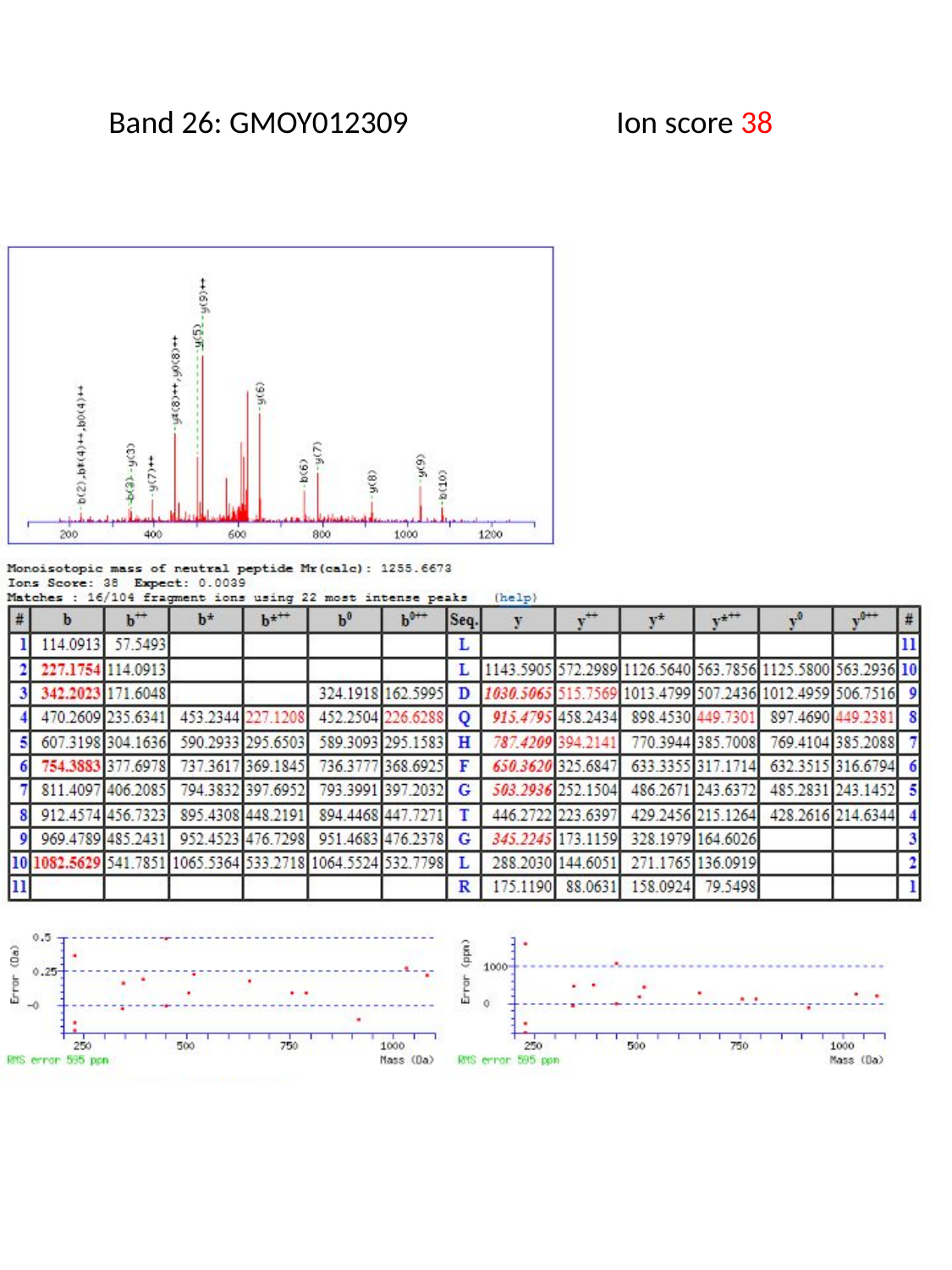

Band 26: GMOY012309 		Ion score 38

## Slide 150
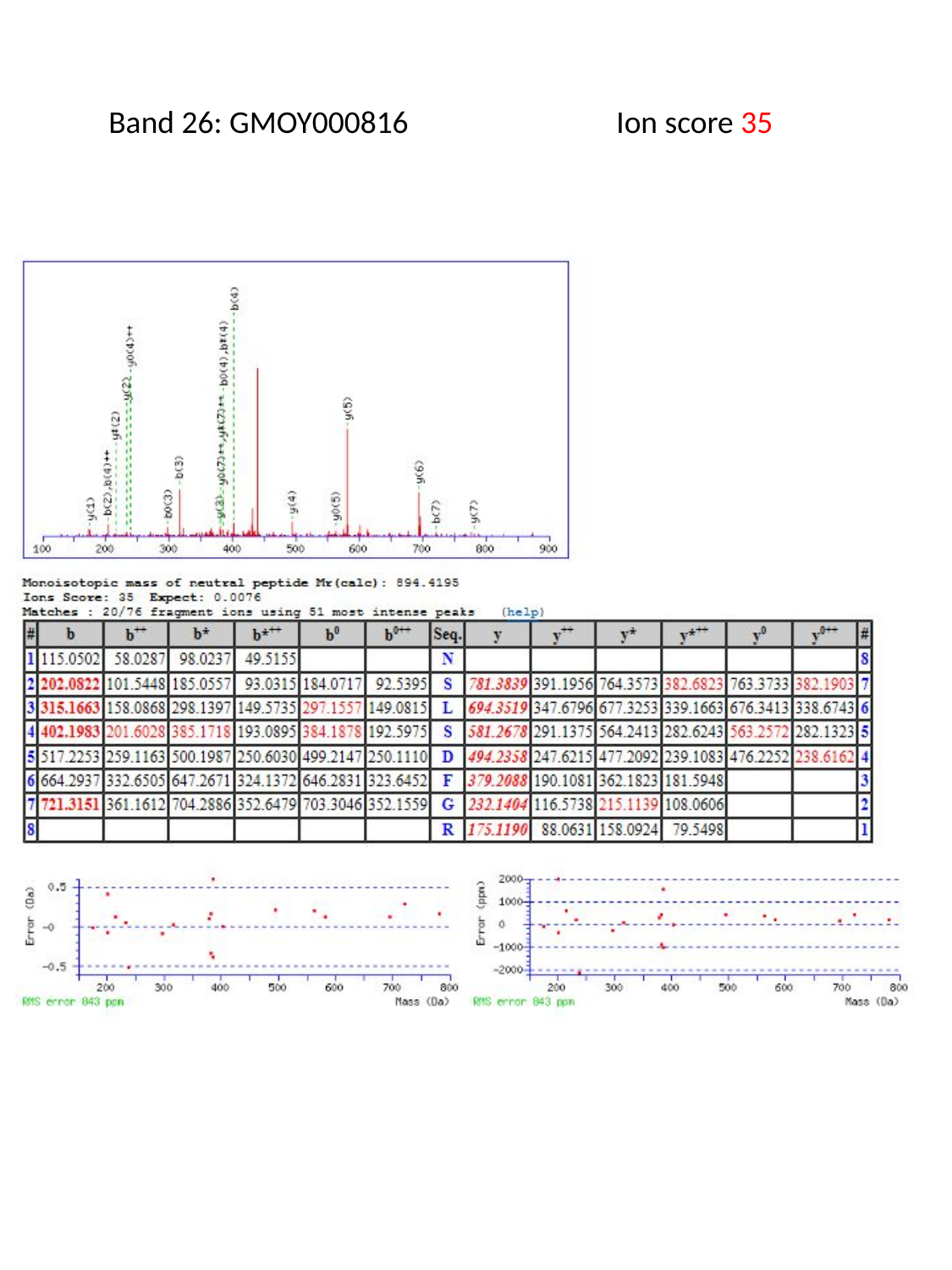

Band 26: GMOY000816 		Ion score 35

## Slide 151
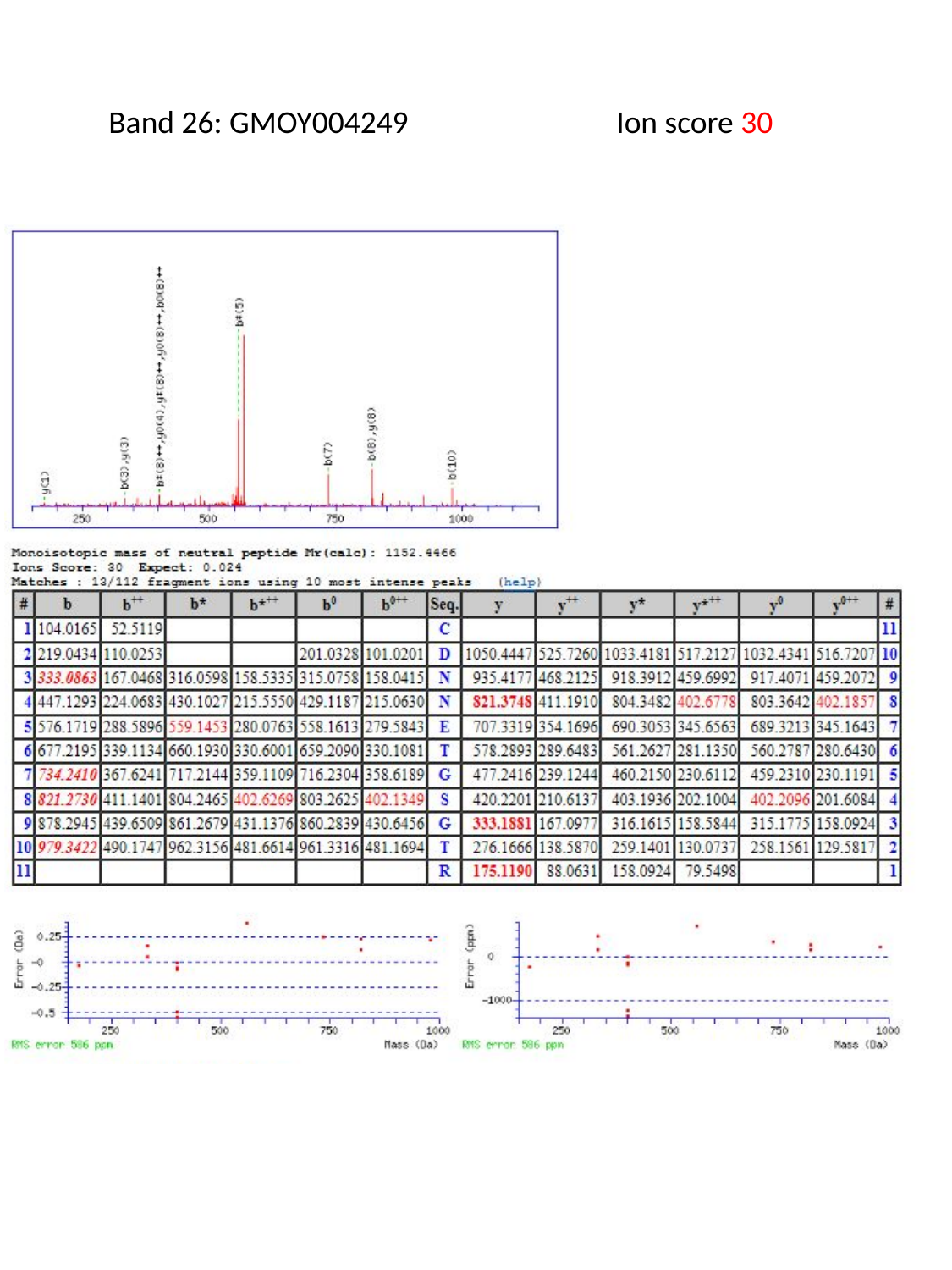

Band 26: GMOY004249 		Ion score 30

## Slide 152
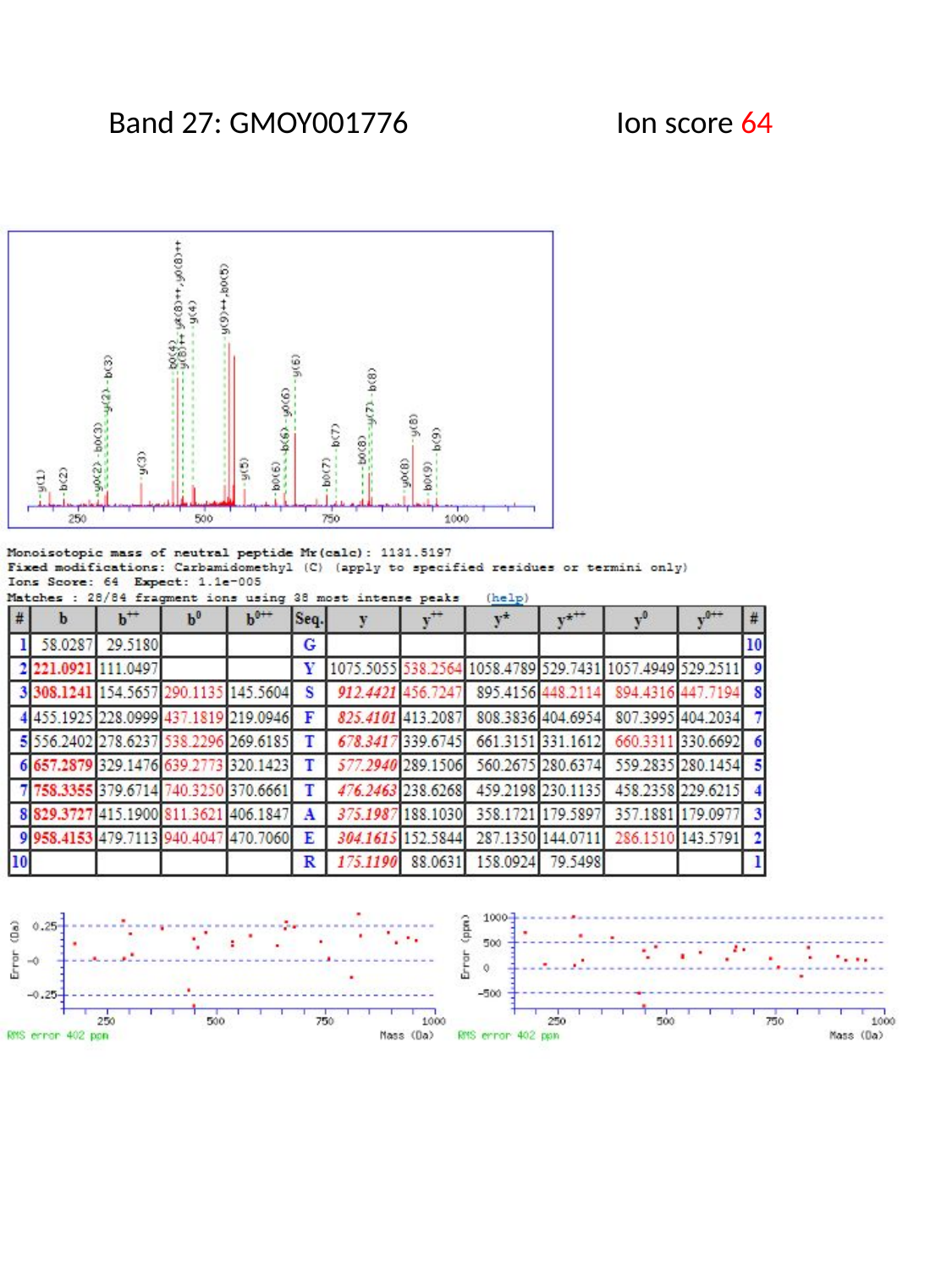

Band 27: GMOY001776 		Ion score 64

## Slide 153
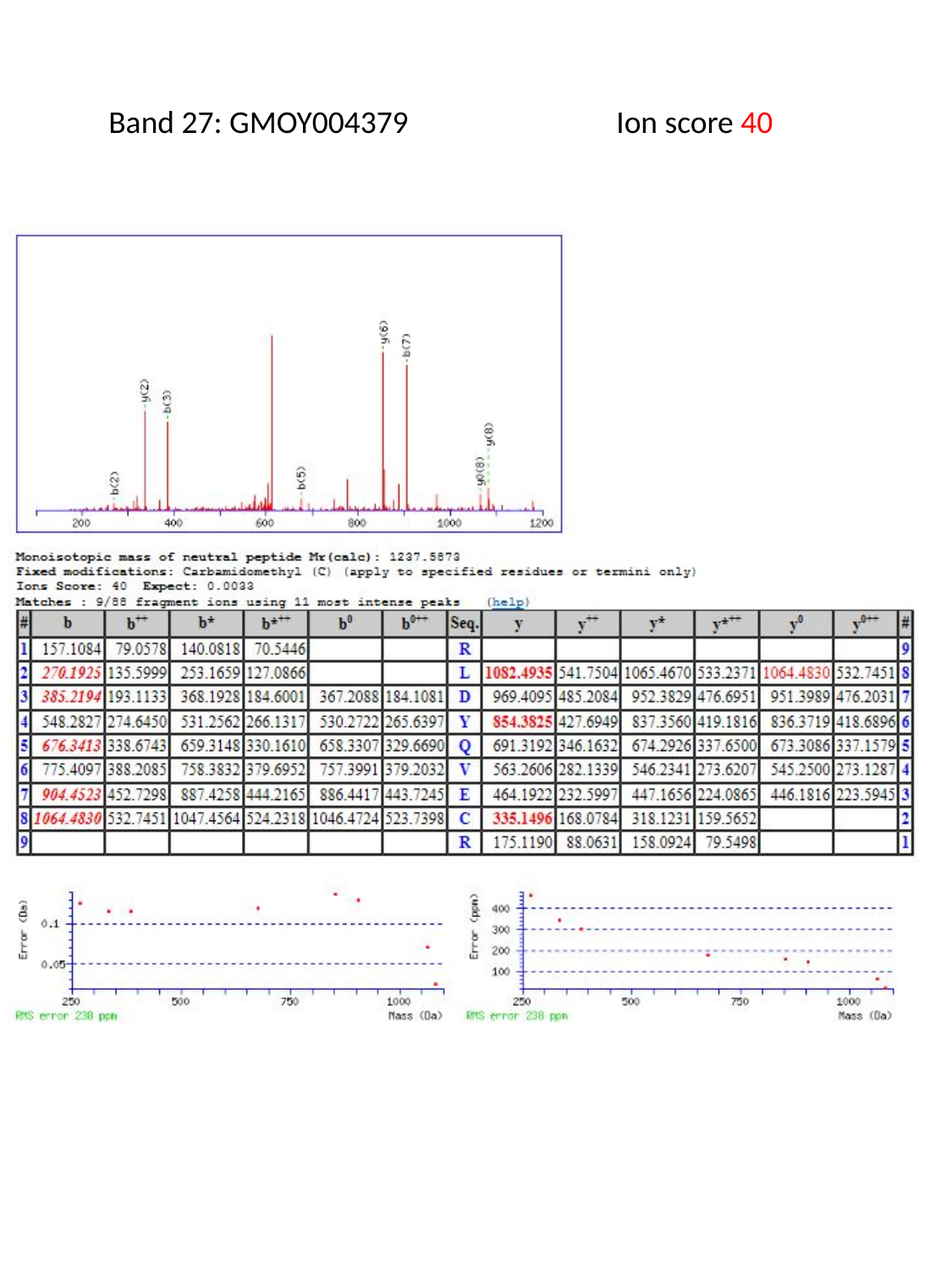

Band 27: GMOY004379 		Ion score 40

## Slide 154
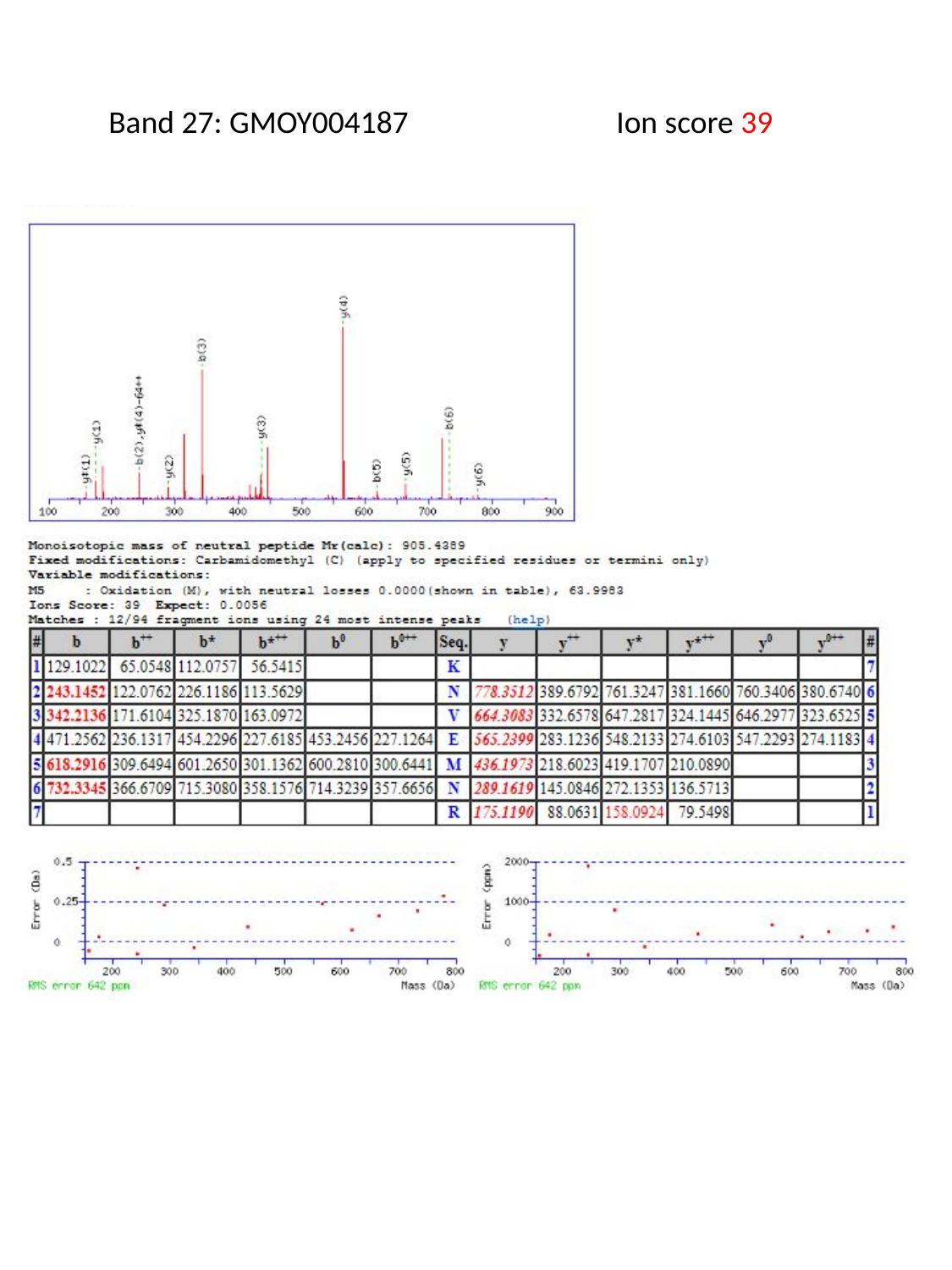

Band 27: GMOY004187 		Ion score 39

## Slide 155
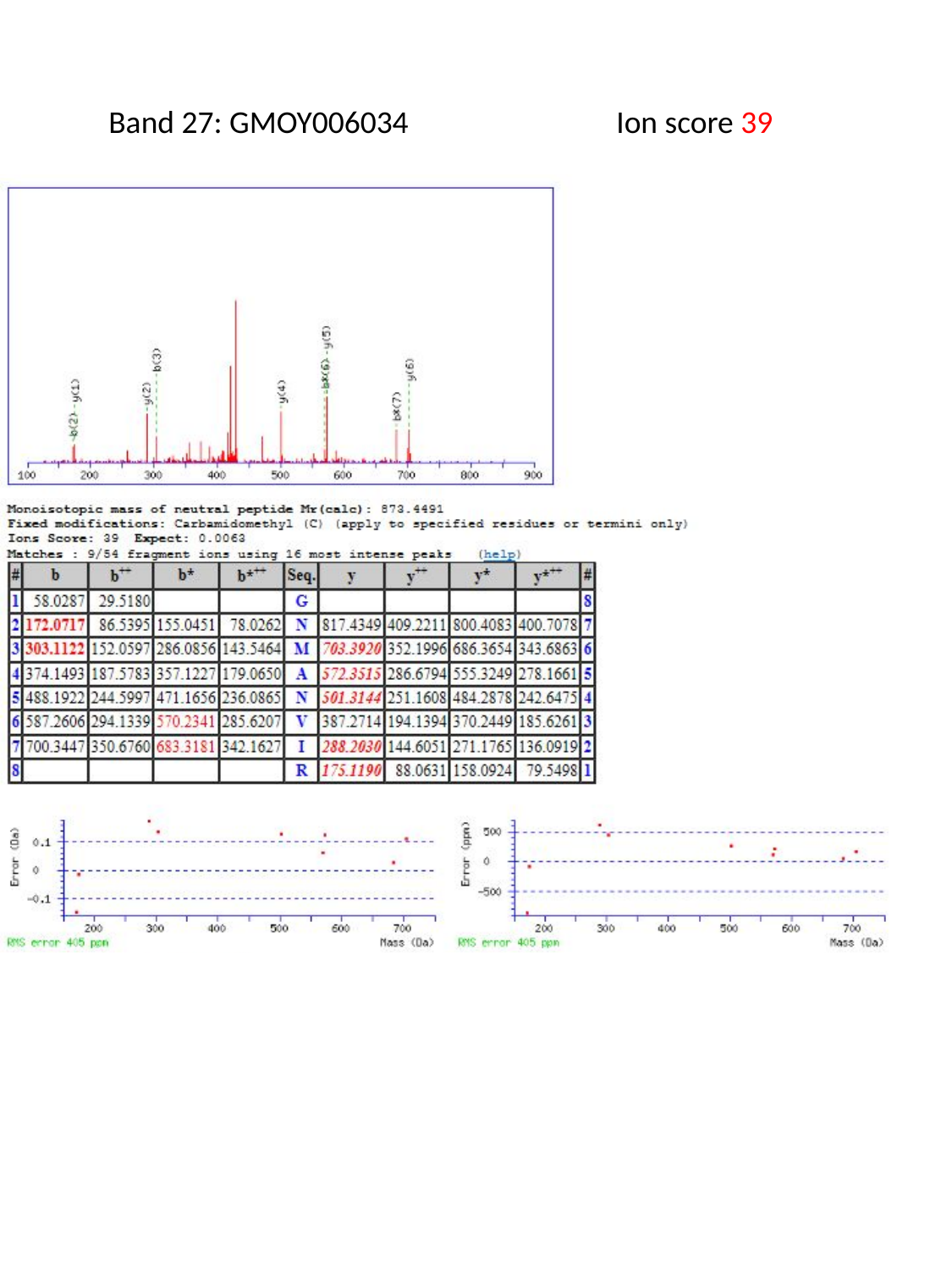

Band 27: GMOY006034 		Ion score 39

## Slide 156
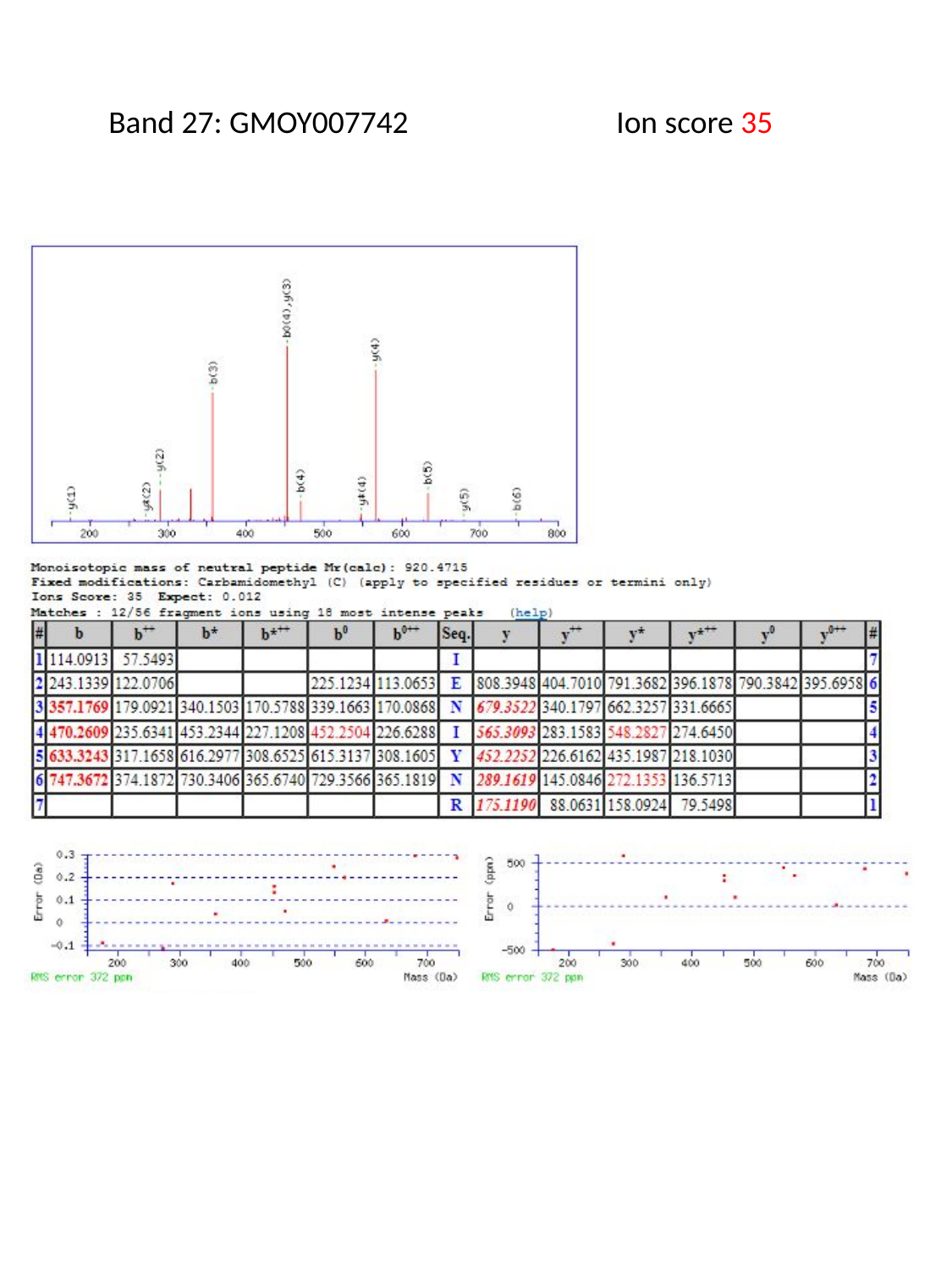

Band 27: GMOY007742 		Ion score 35

## Slide 157
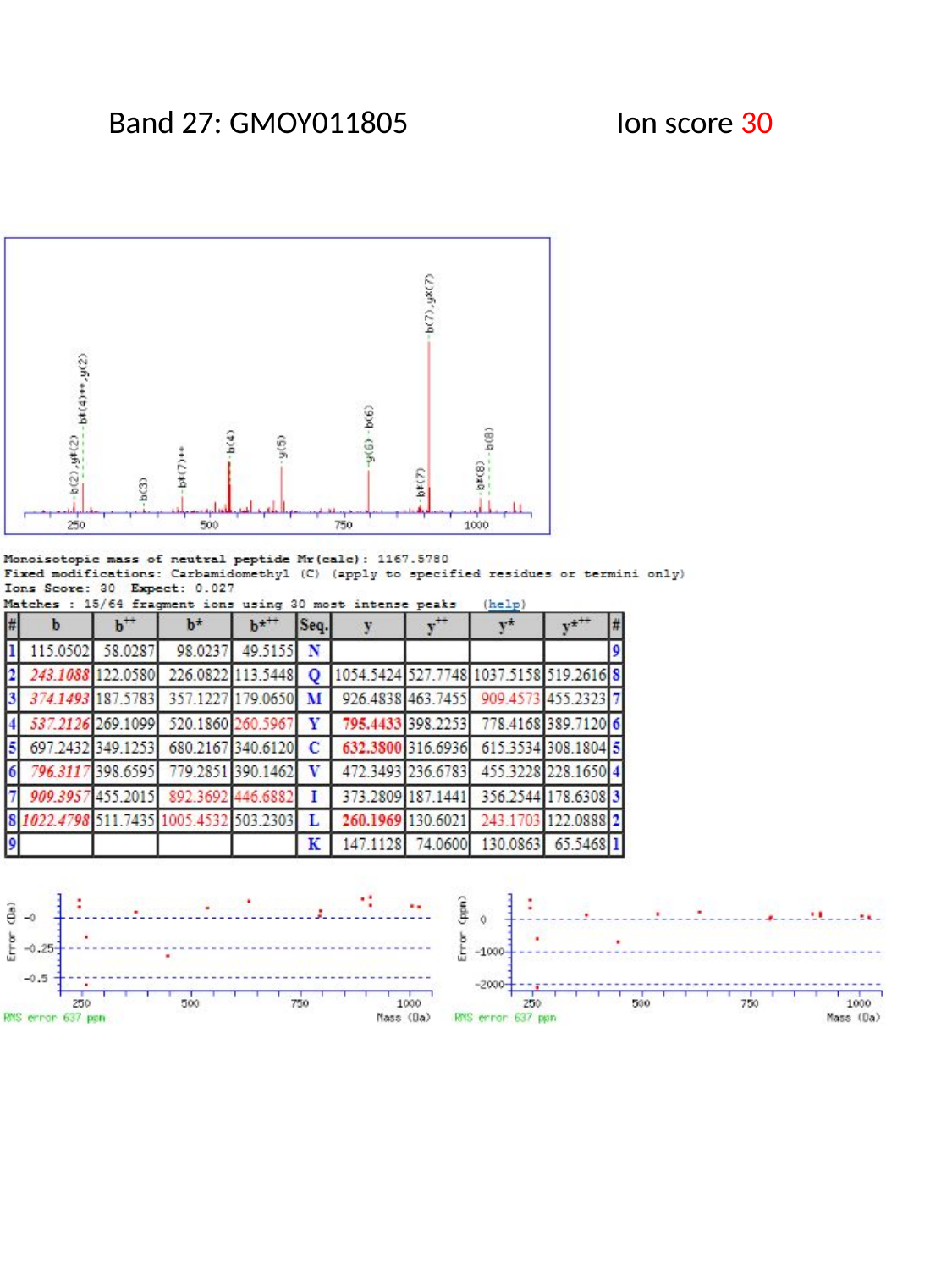

Band 27: GMOY011805 		Ion score 30

## Slide 158
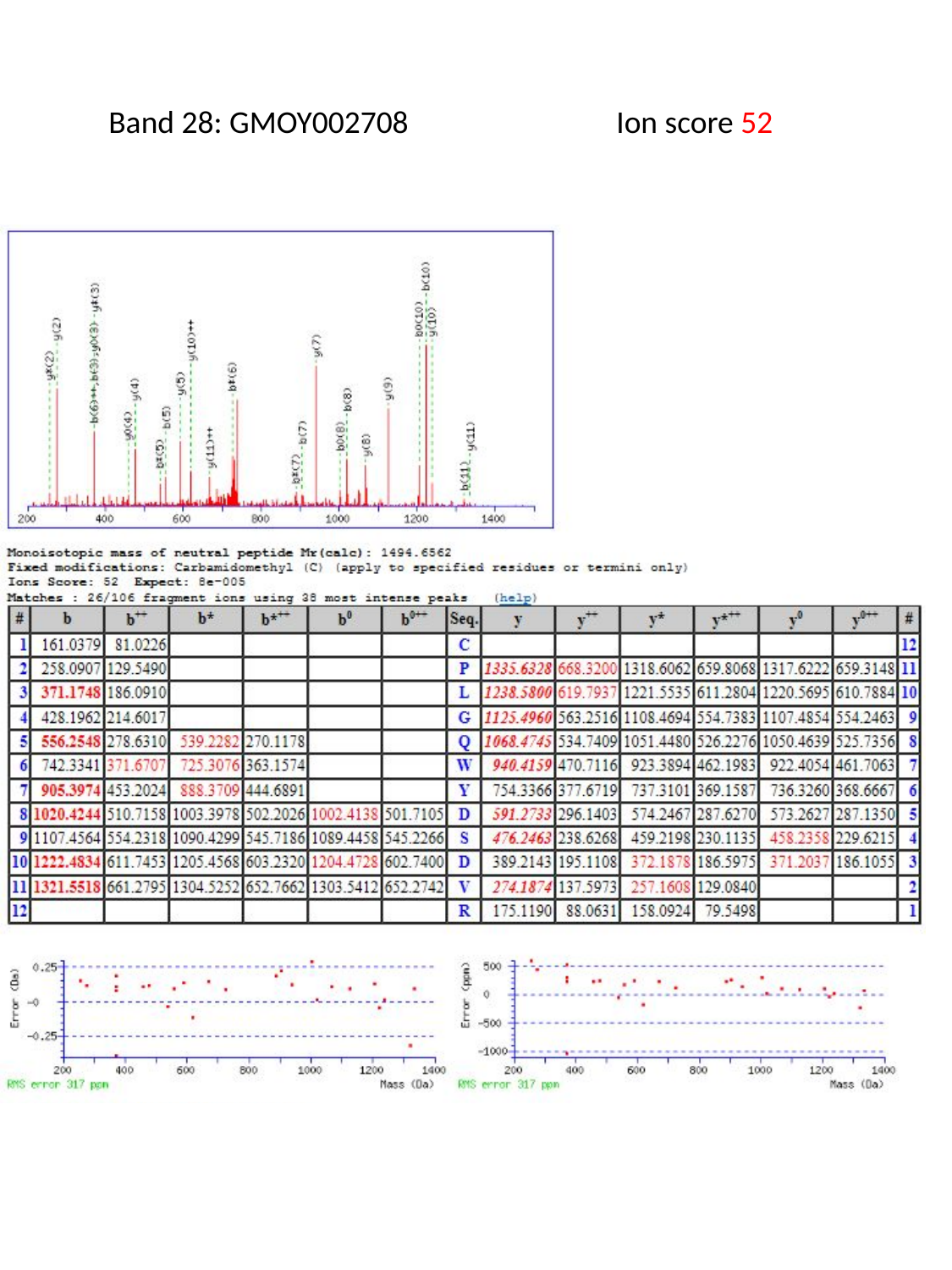

Band 28: GMOY002708 		Ion score 52

## Slide 159
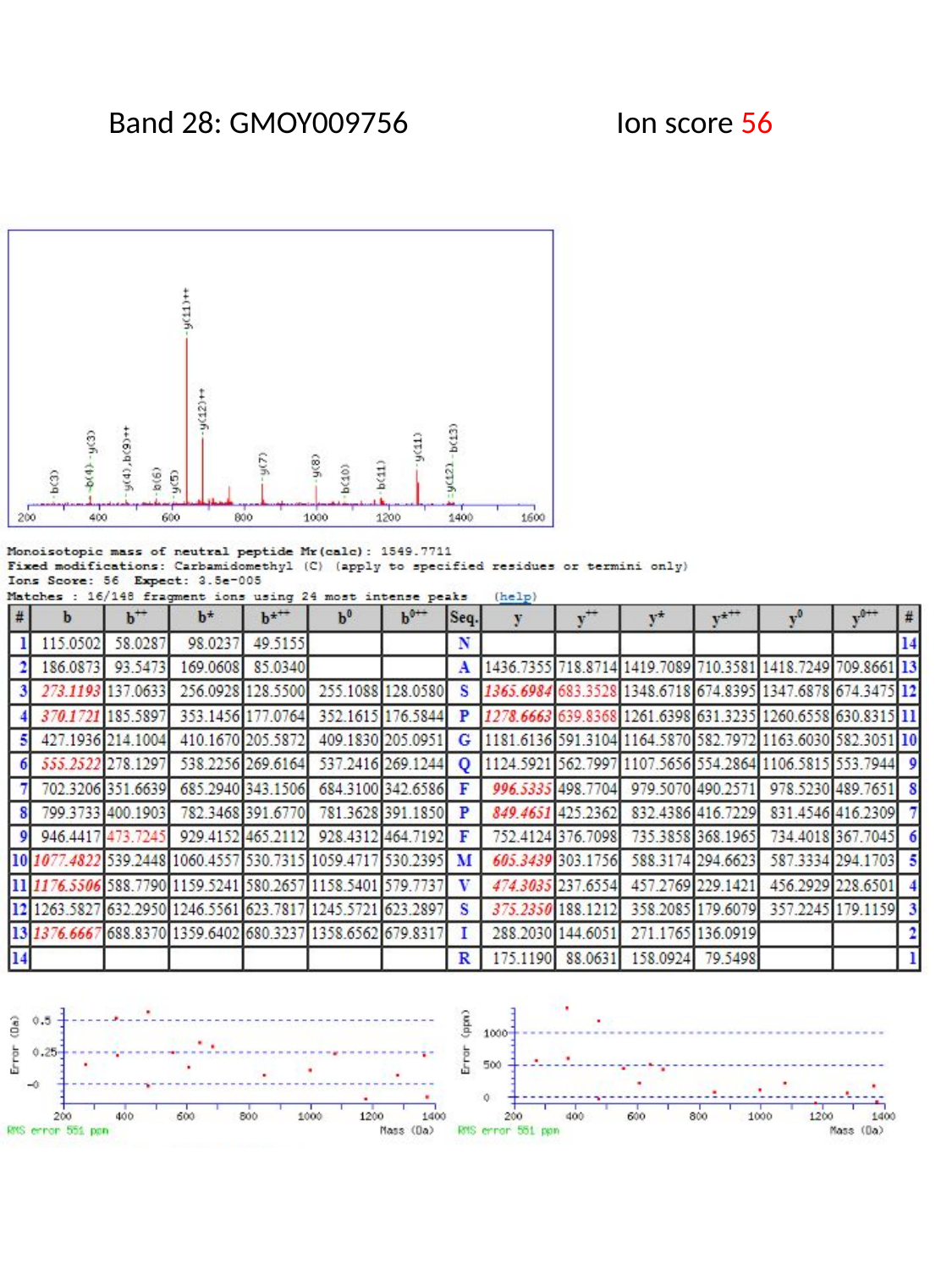

Band 28: GMOY009756 		Ion score 56

## Slide 160
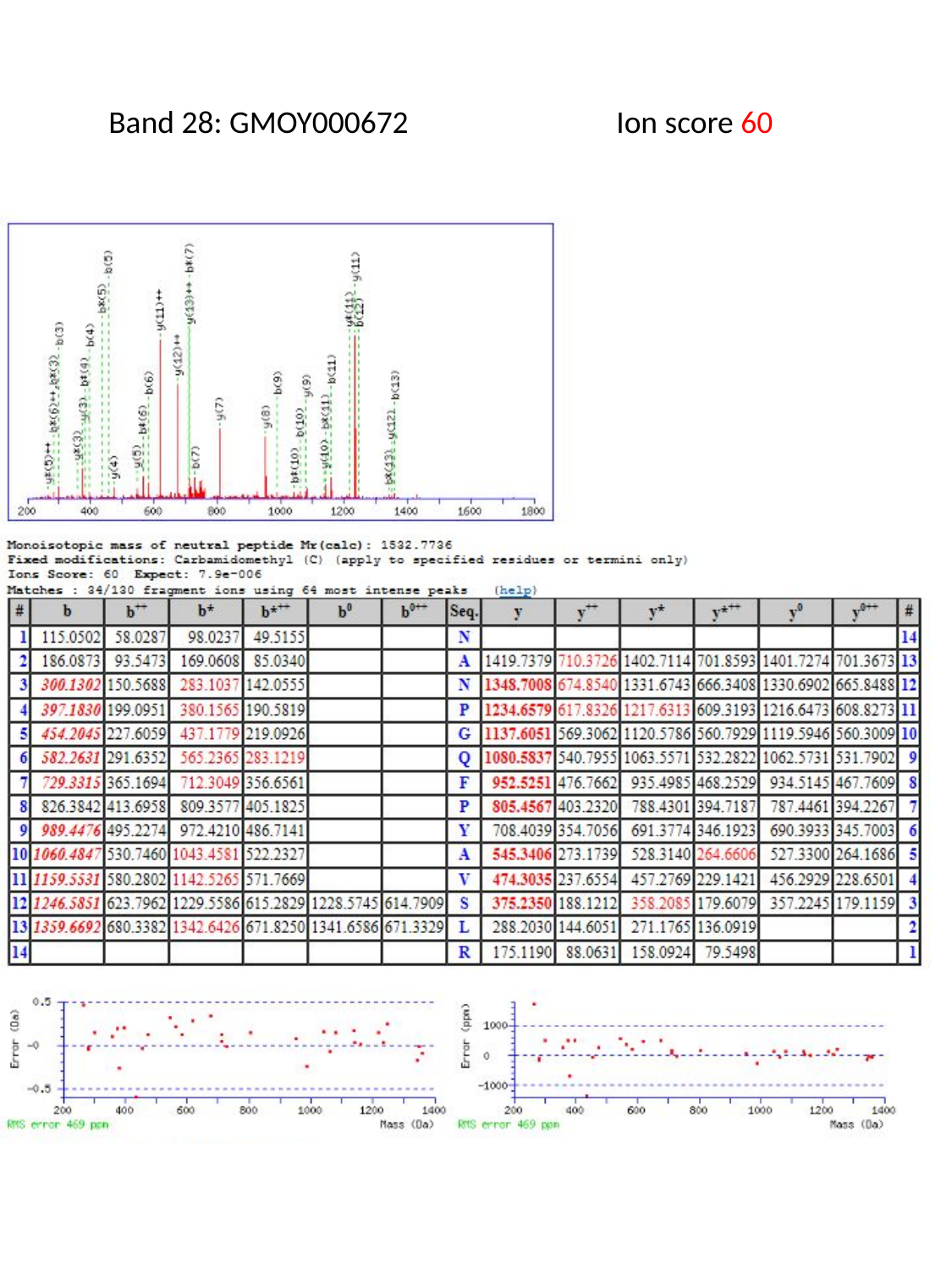

Band 28: GMOY000672 		Ion score 60

## Slide 161
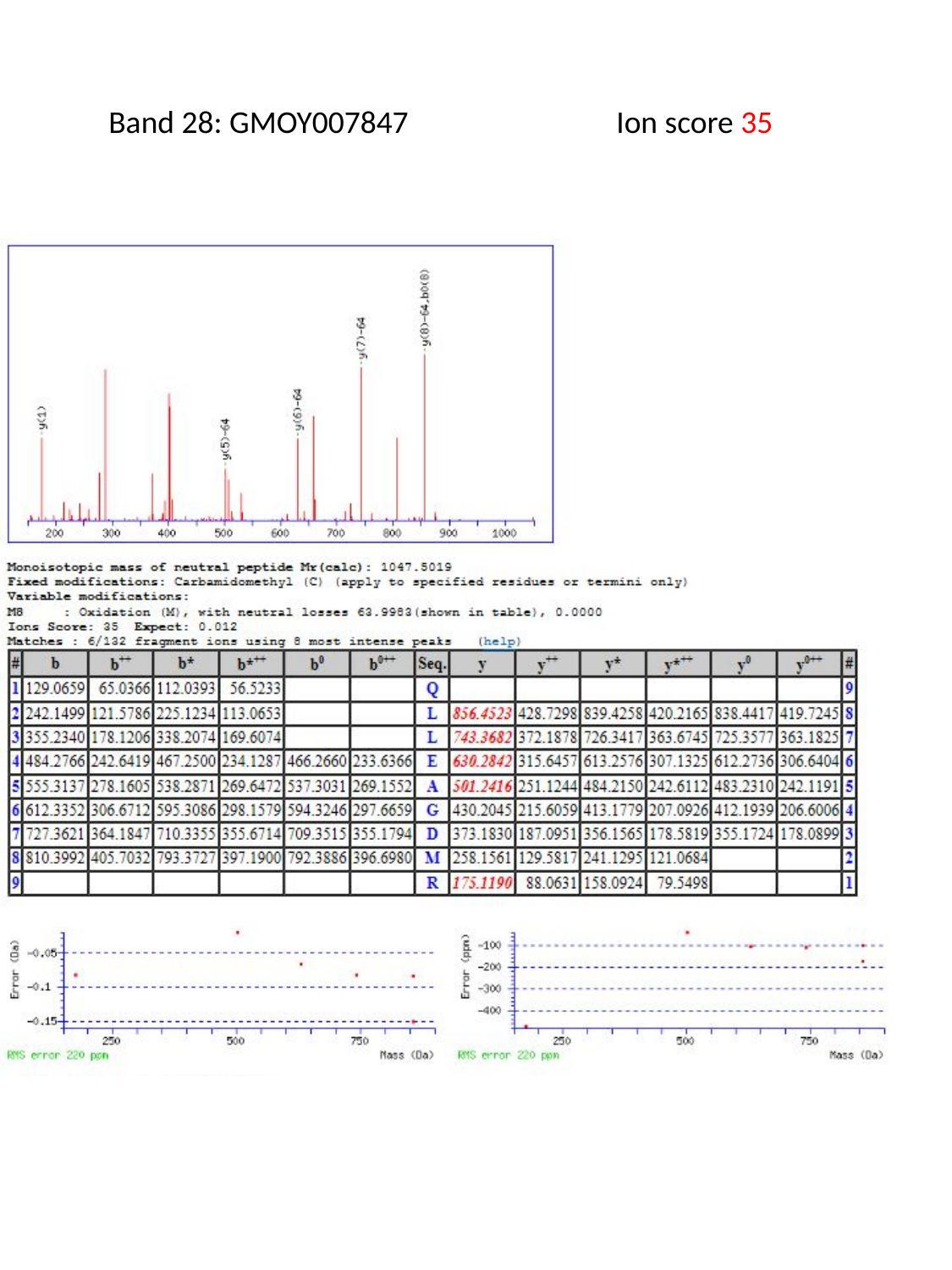

Band 28: GMOY007847 		Ion score 35

## Slide 162
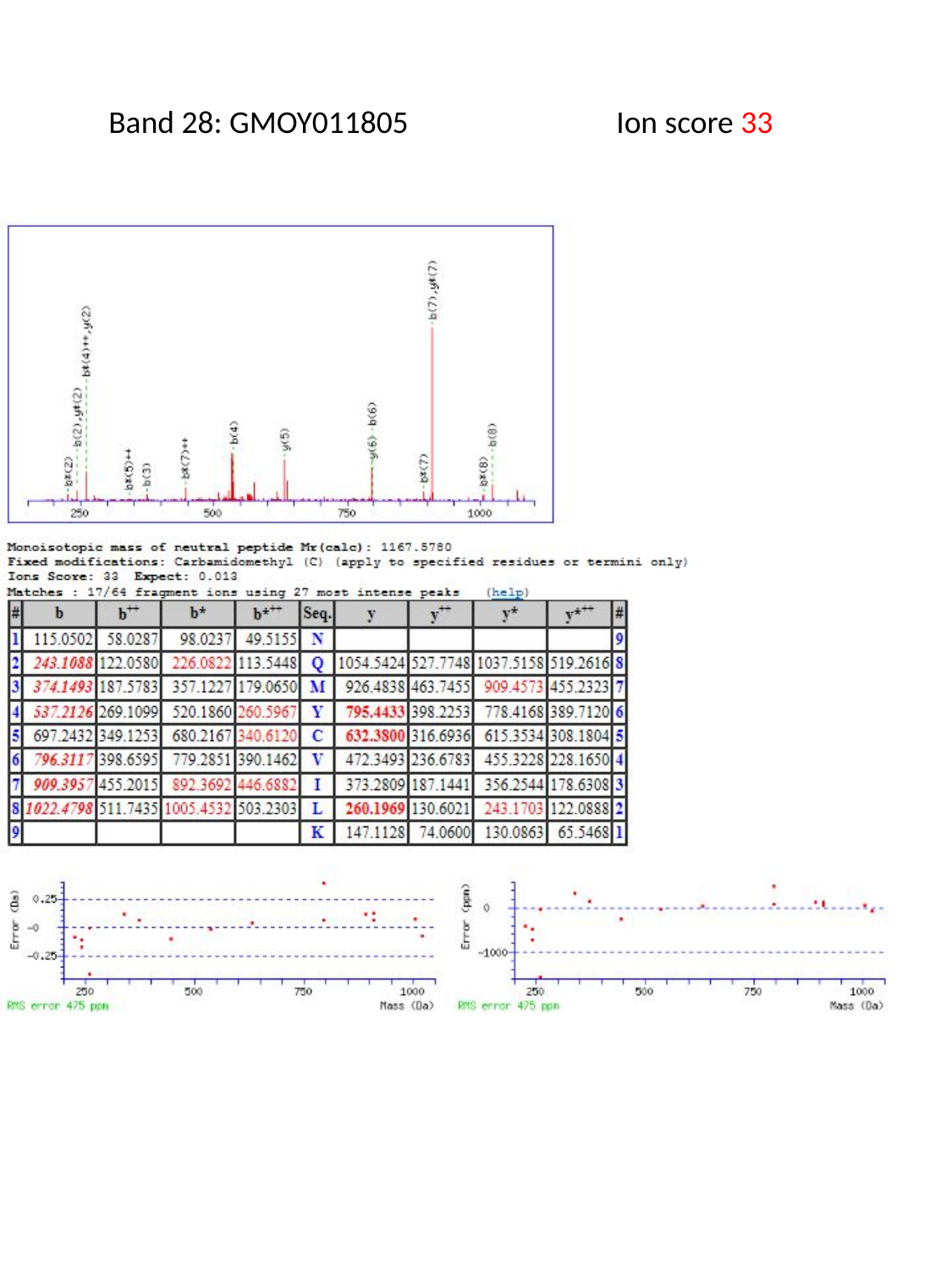

Band 28: GMOY011805 		Ion score 33

## Slide 163
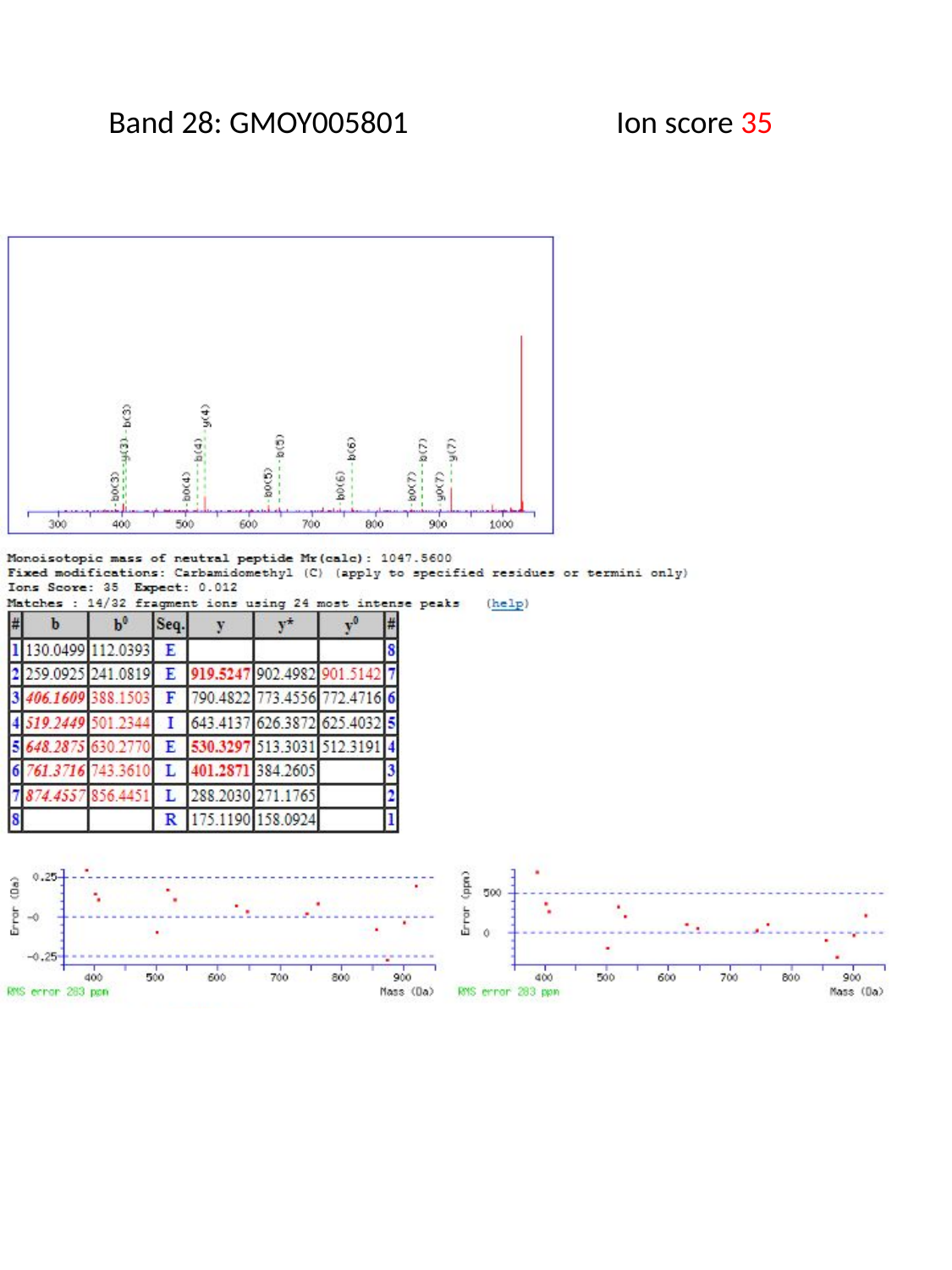

Band 28: GMOY005801 		Ion score 35

## Slide 164
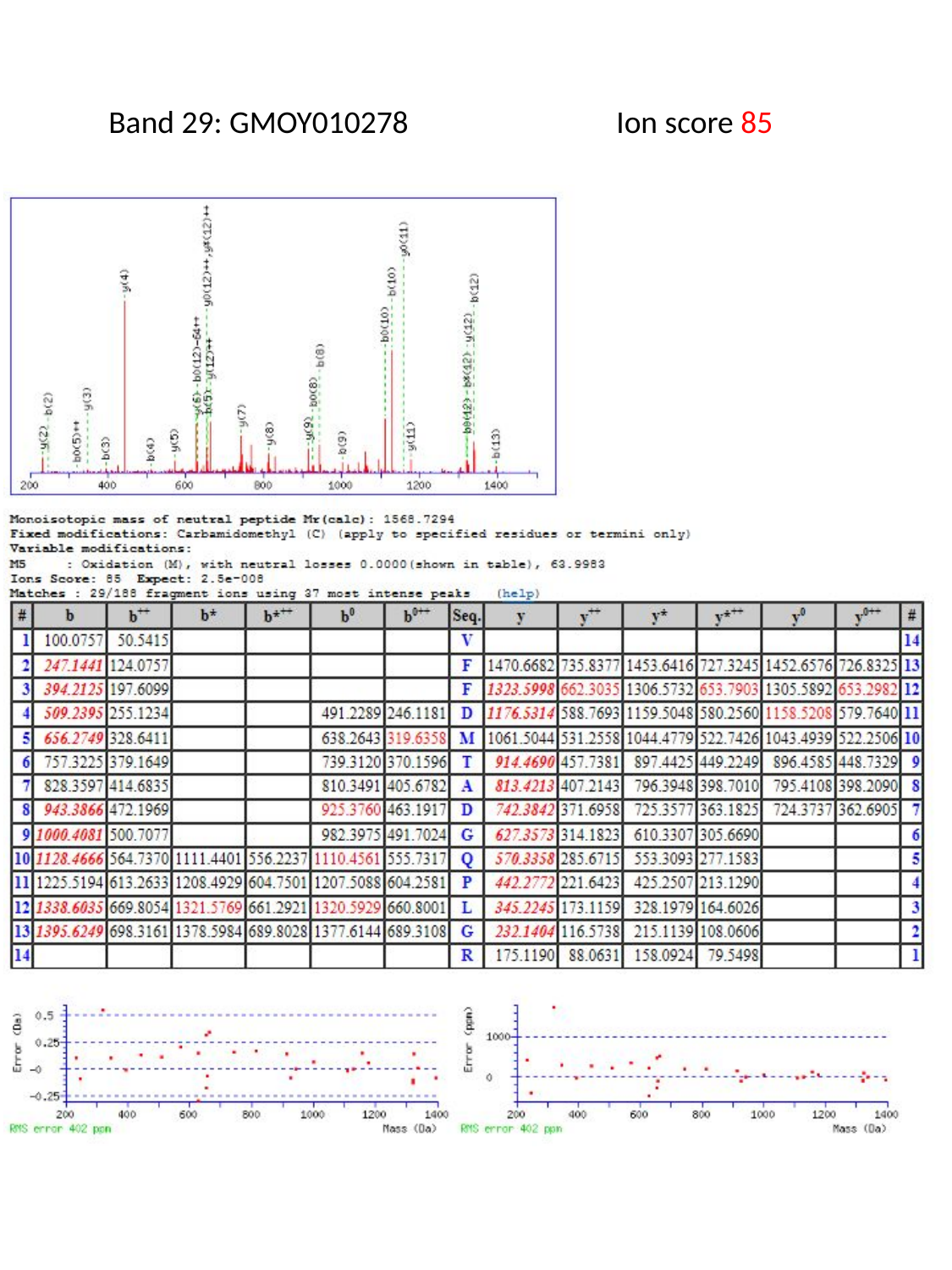

Band 29: GMOY010278 		Ion score 85

## Slide 165
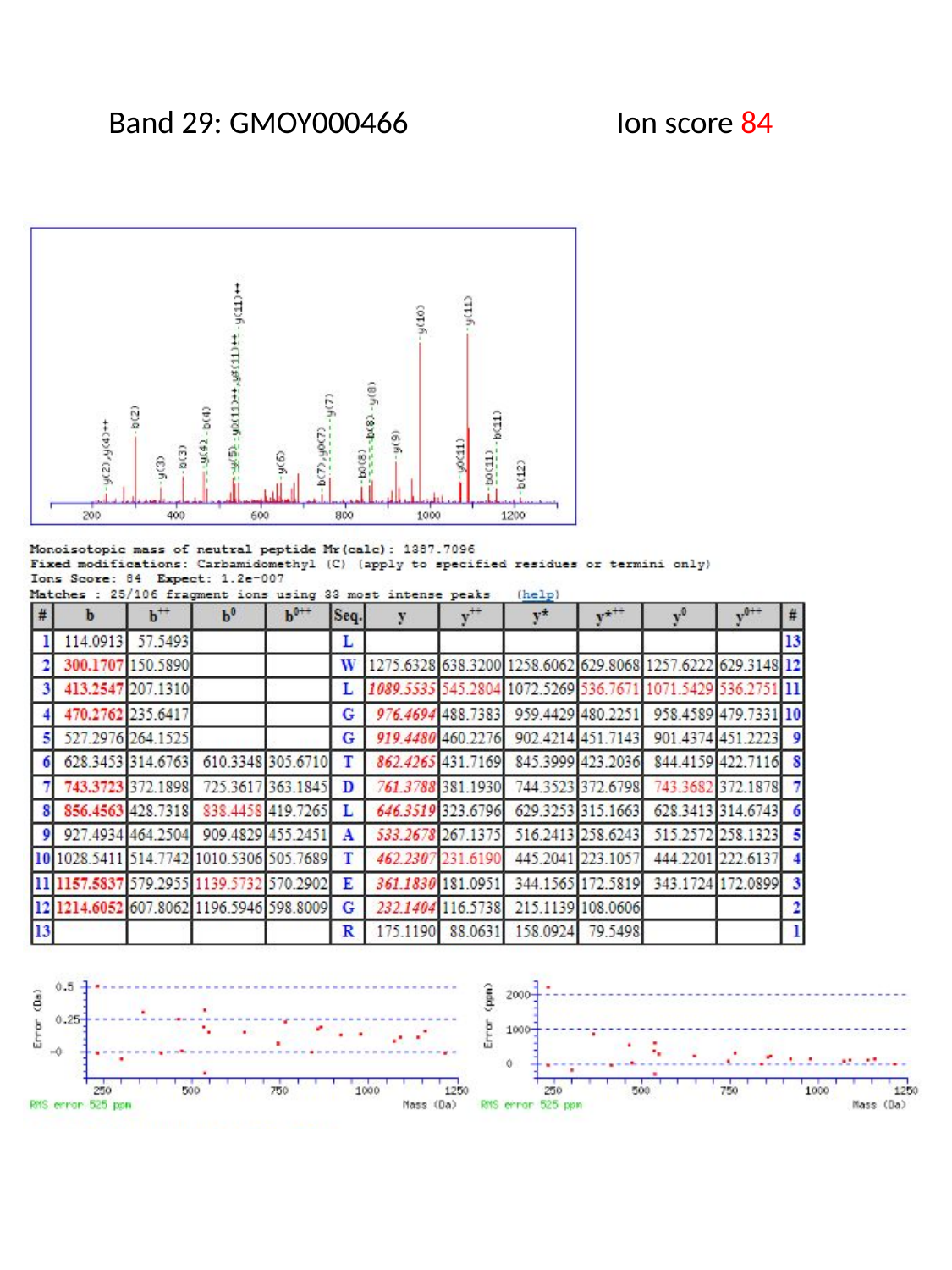

Band 29: GMOY000466 		Ion score 84

## Slide 166
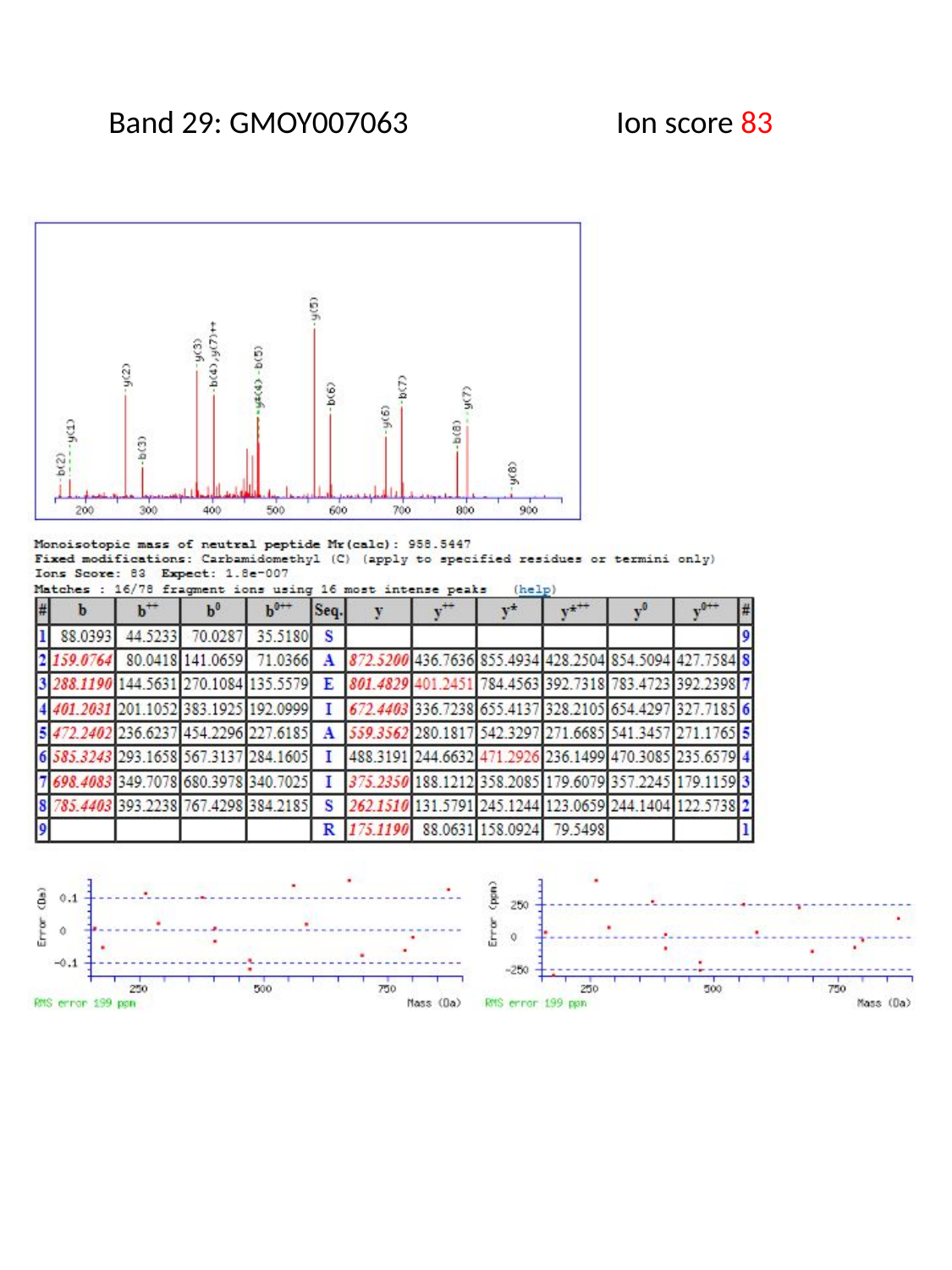

Band 29: GMOY007063 		Ion score 83

## Slide 167
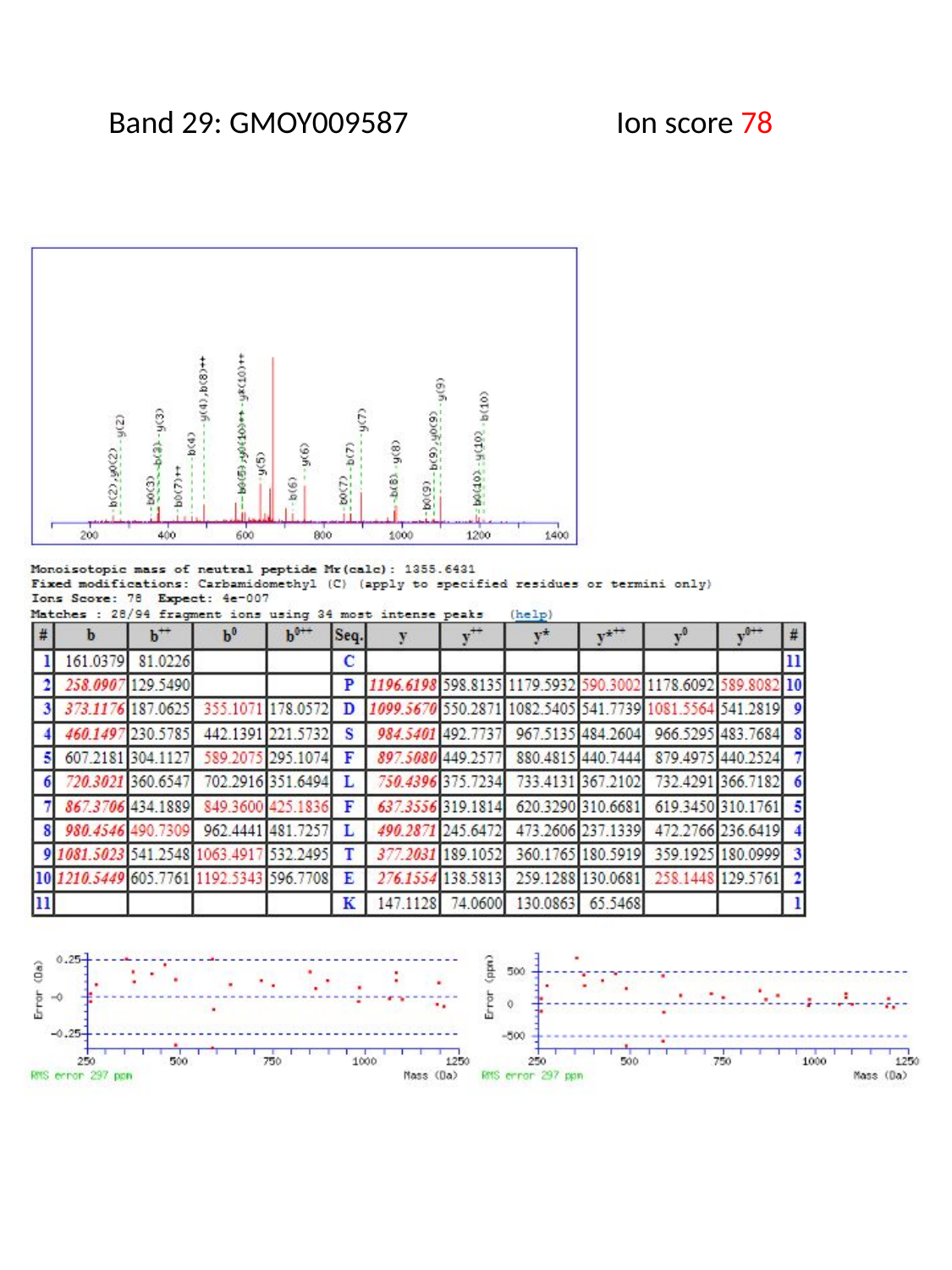

Band 29: GMOY009587 		Ion score 78

## Slide 168
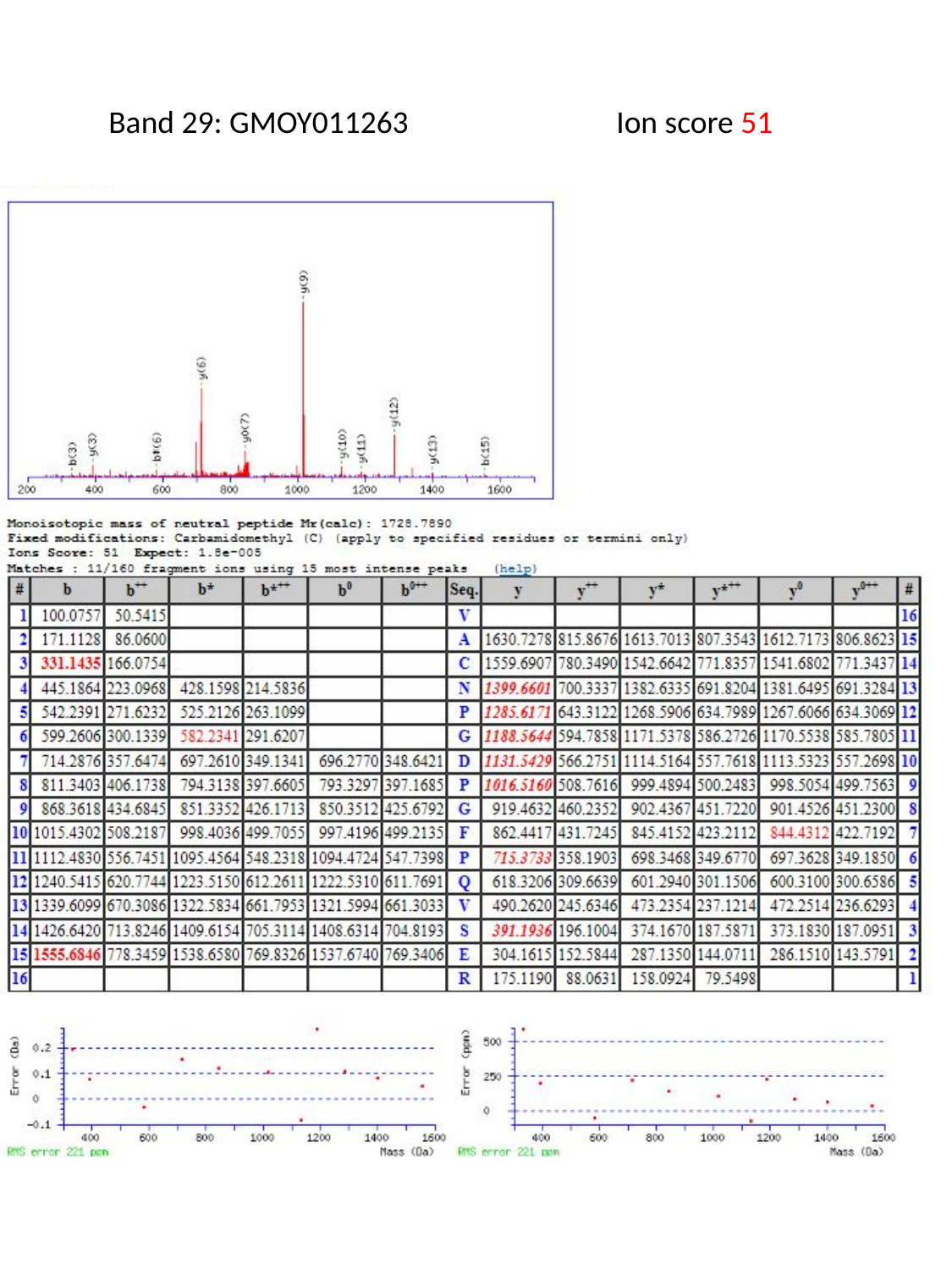

Band 29: GMOY011263 		Ion score 51

## Slide 169
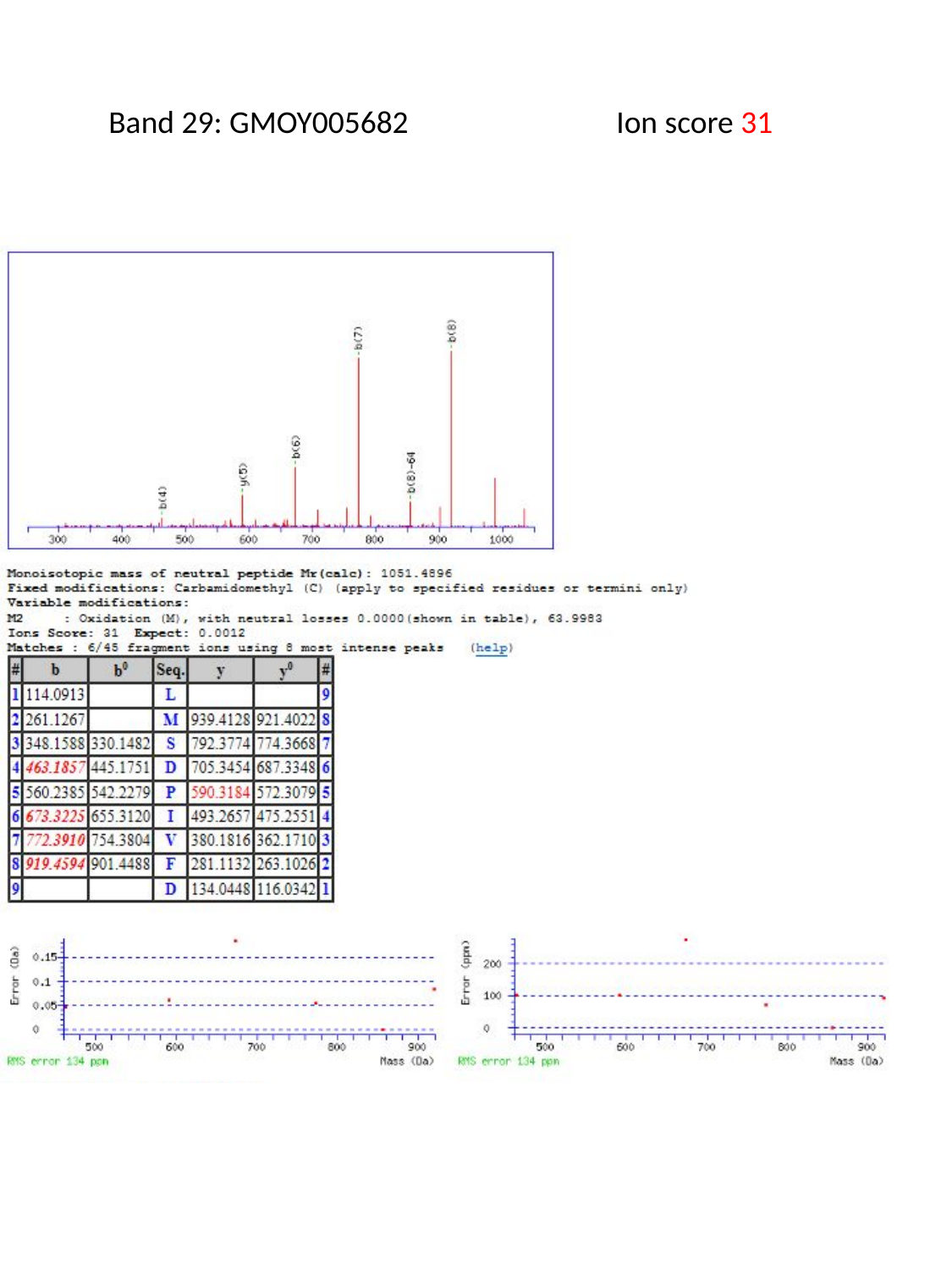

Band 29: GMOY005682 		Ion score 31

## Slide 170
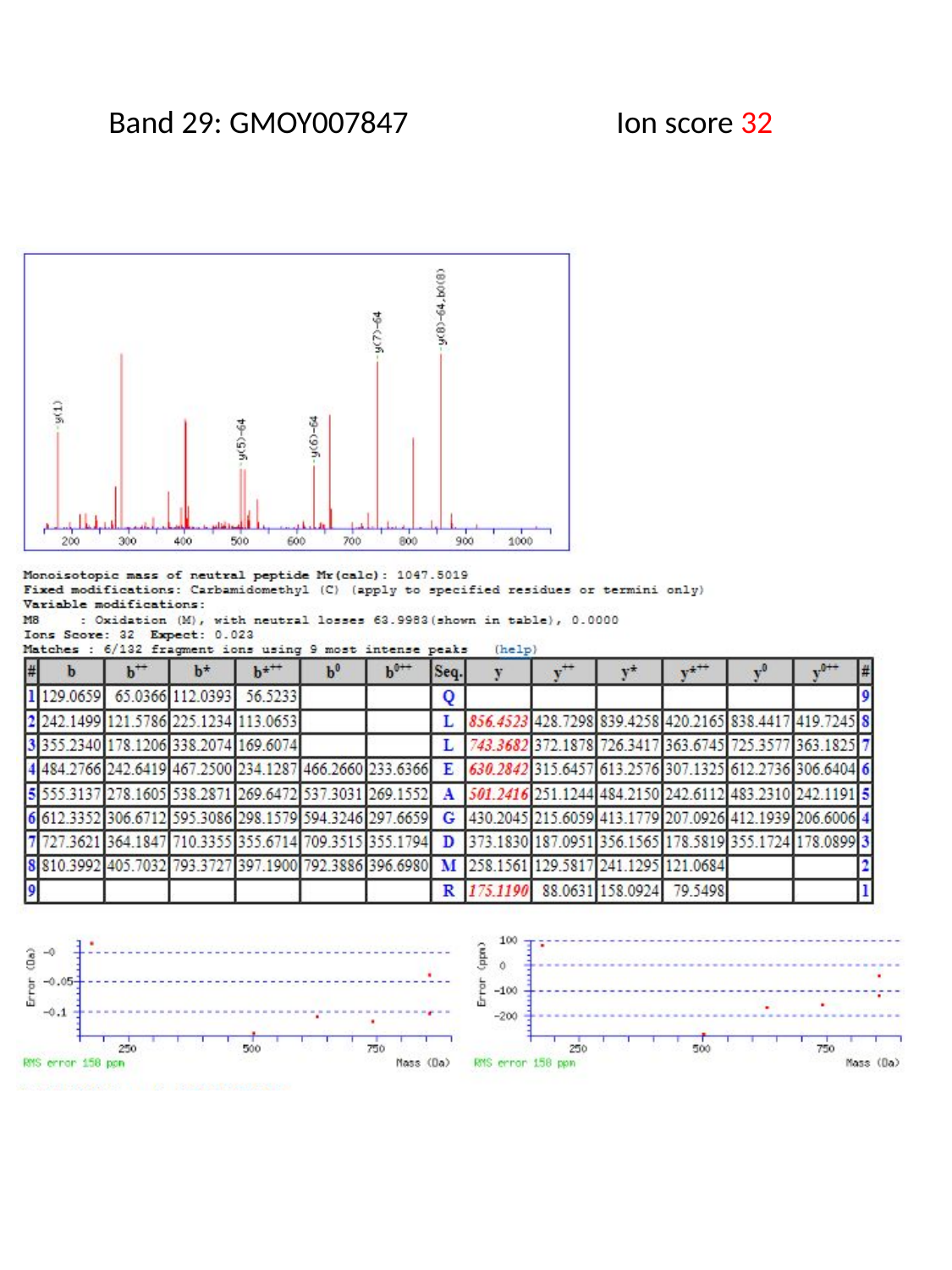

Band 29: GMOY007847 		Ion score 32

## Slide 171
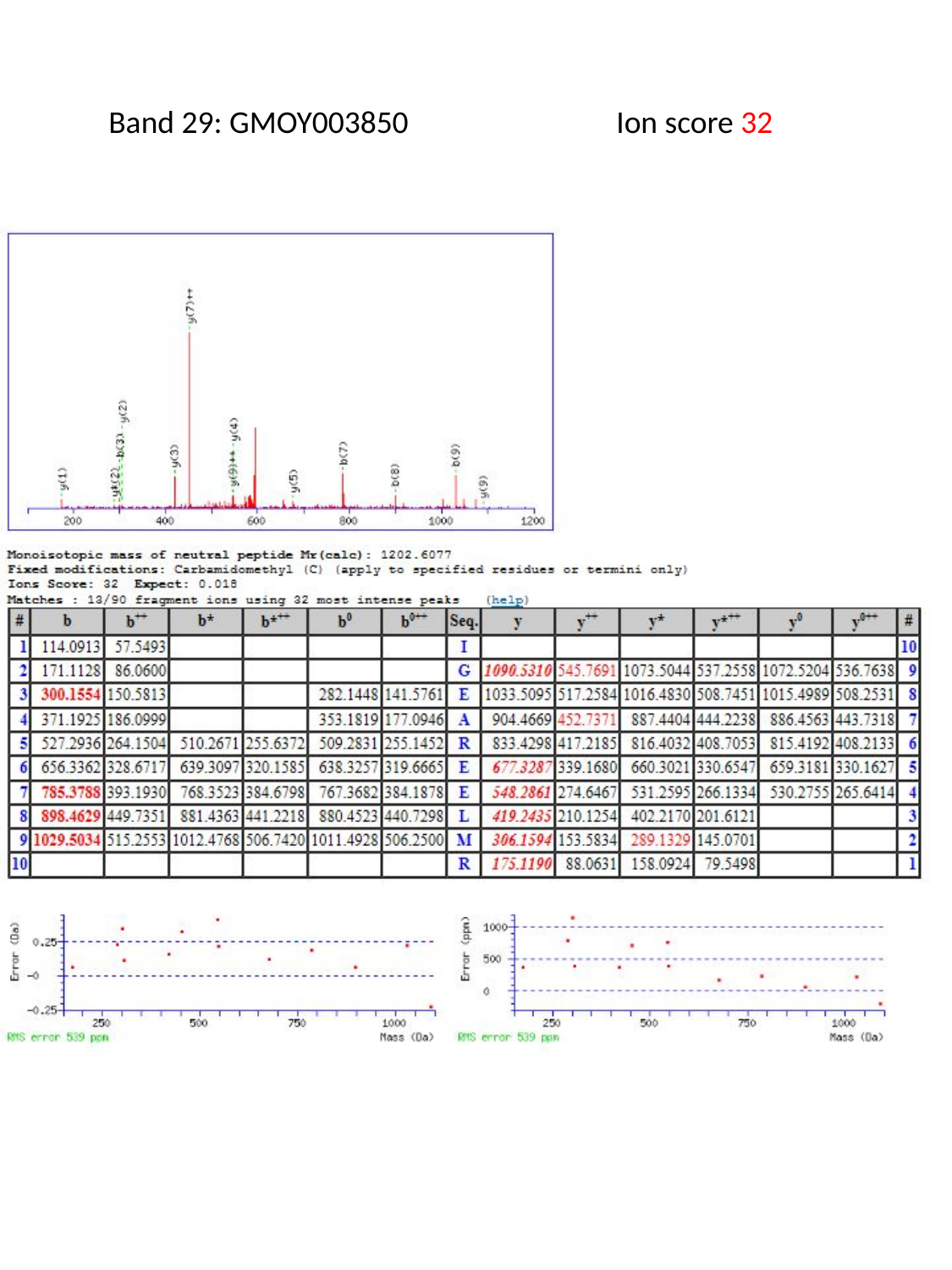

Band 29: GMOY003850 		Ion score 32

## Slide 172
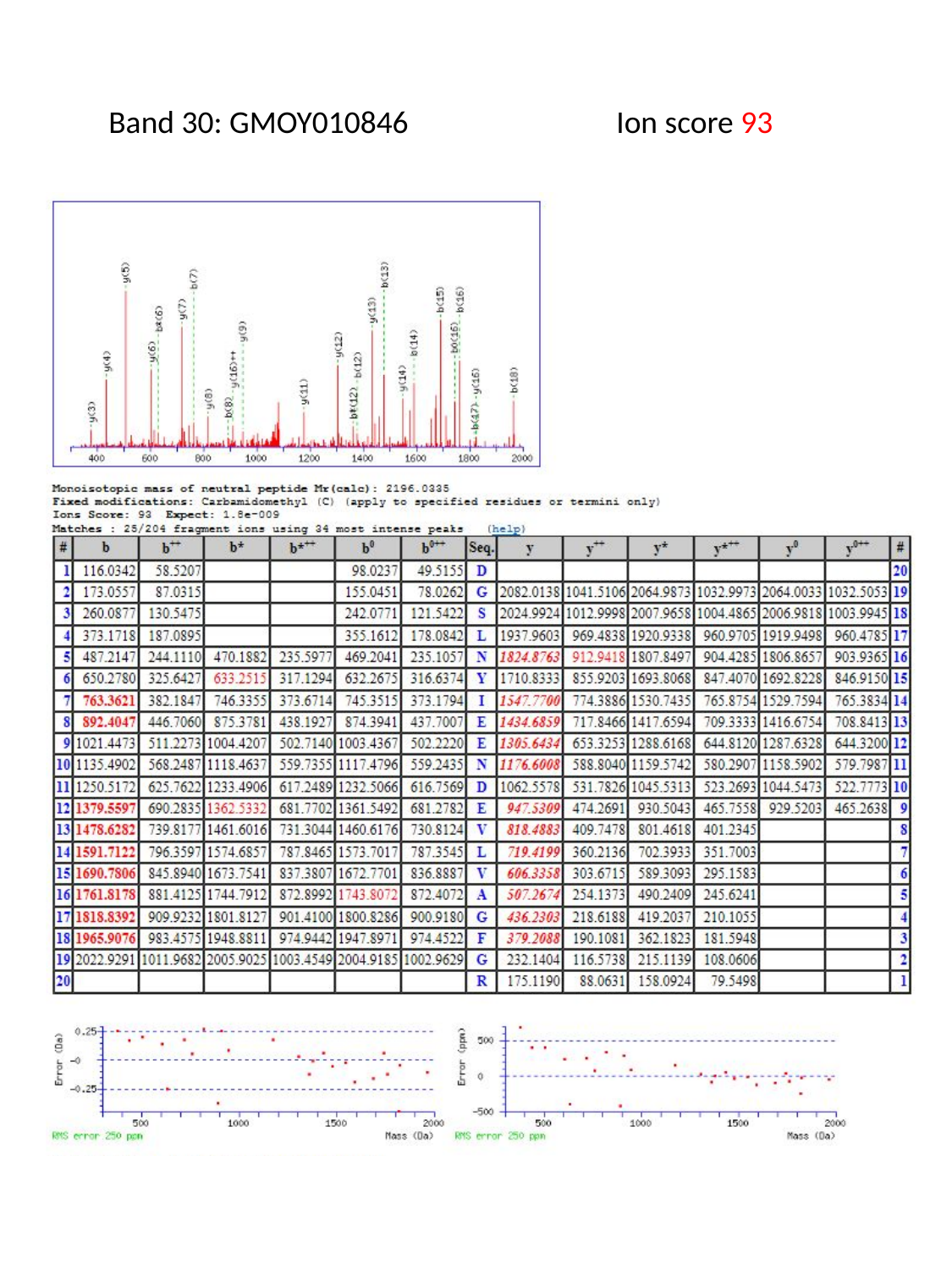

Band 30: GMOY010846 		Ion score 93

## Slide 173
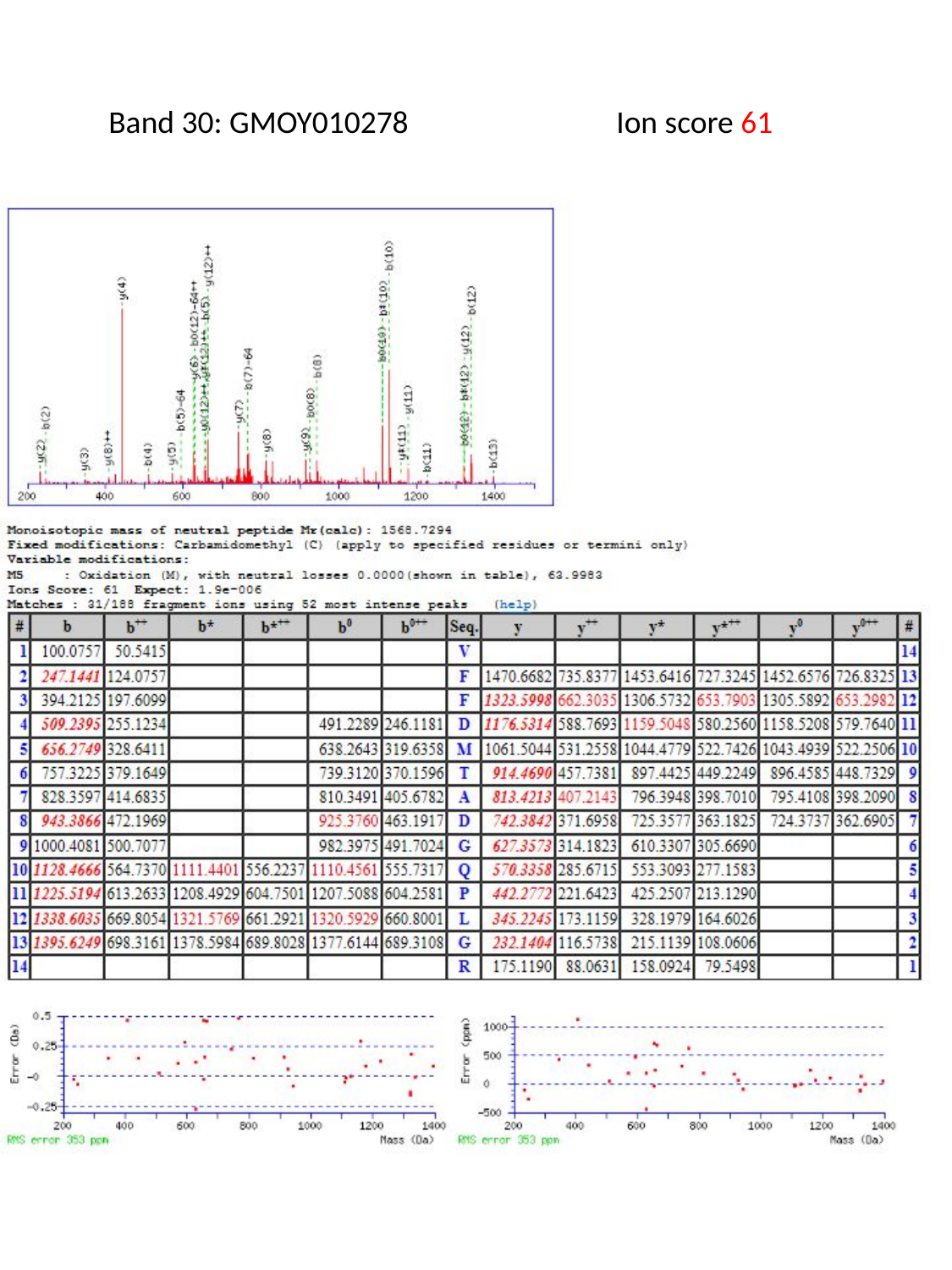

Band 30: GMOY010278 		Ion score 61

## Slide 174
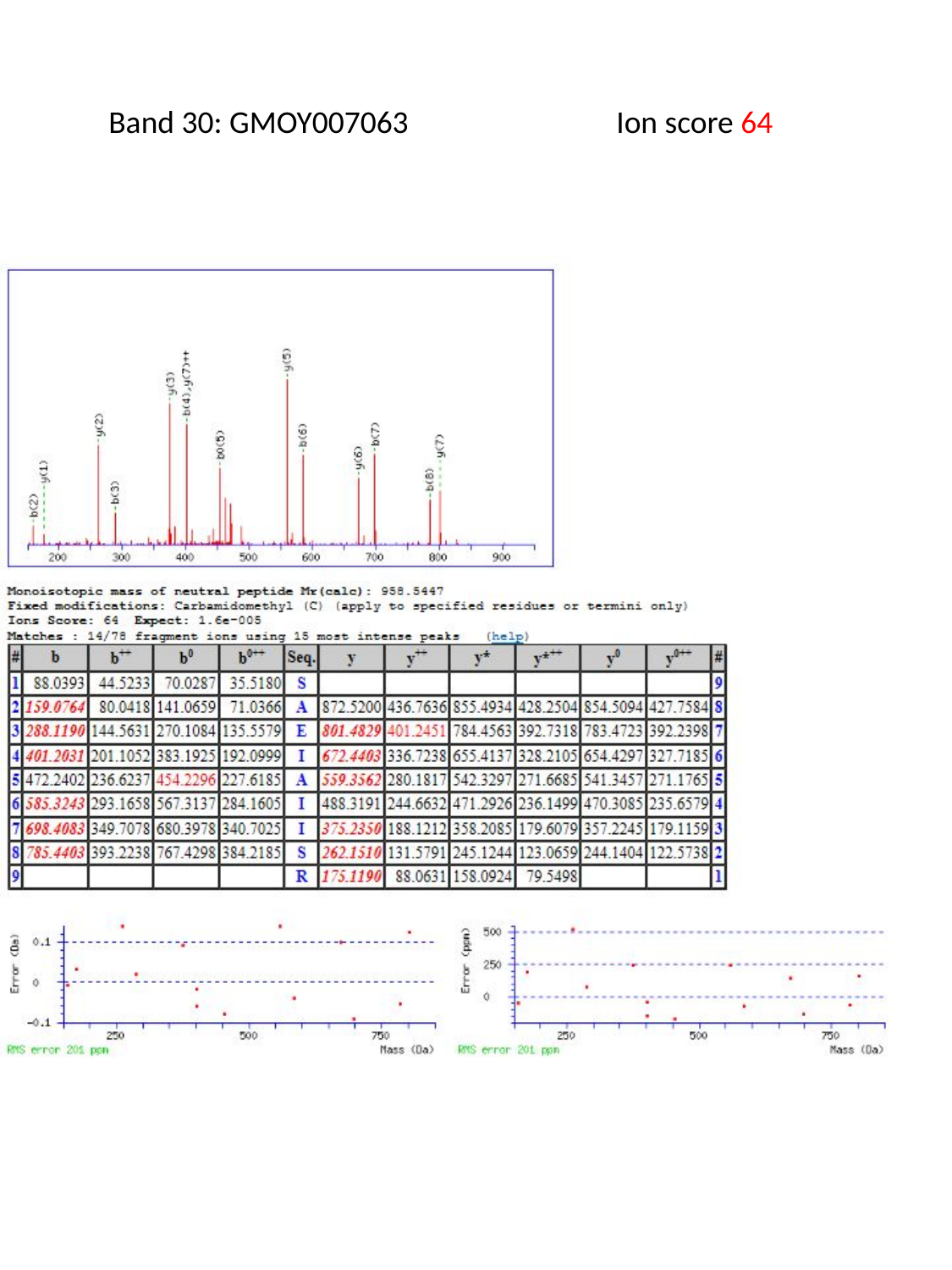

Band 30: GMOY007063 		Ion score 64

## Slide 175
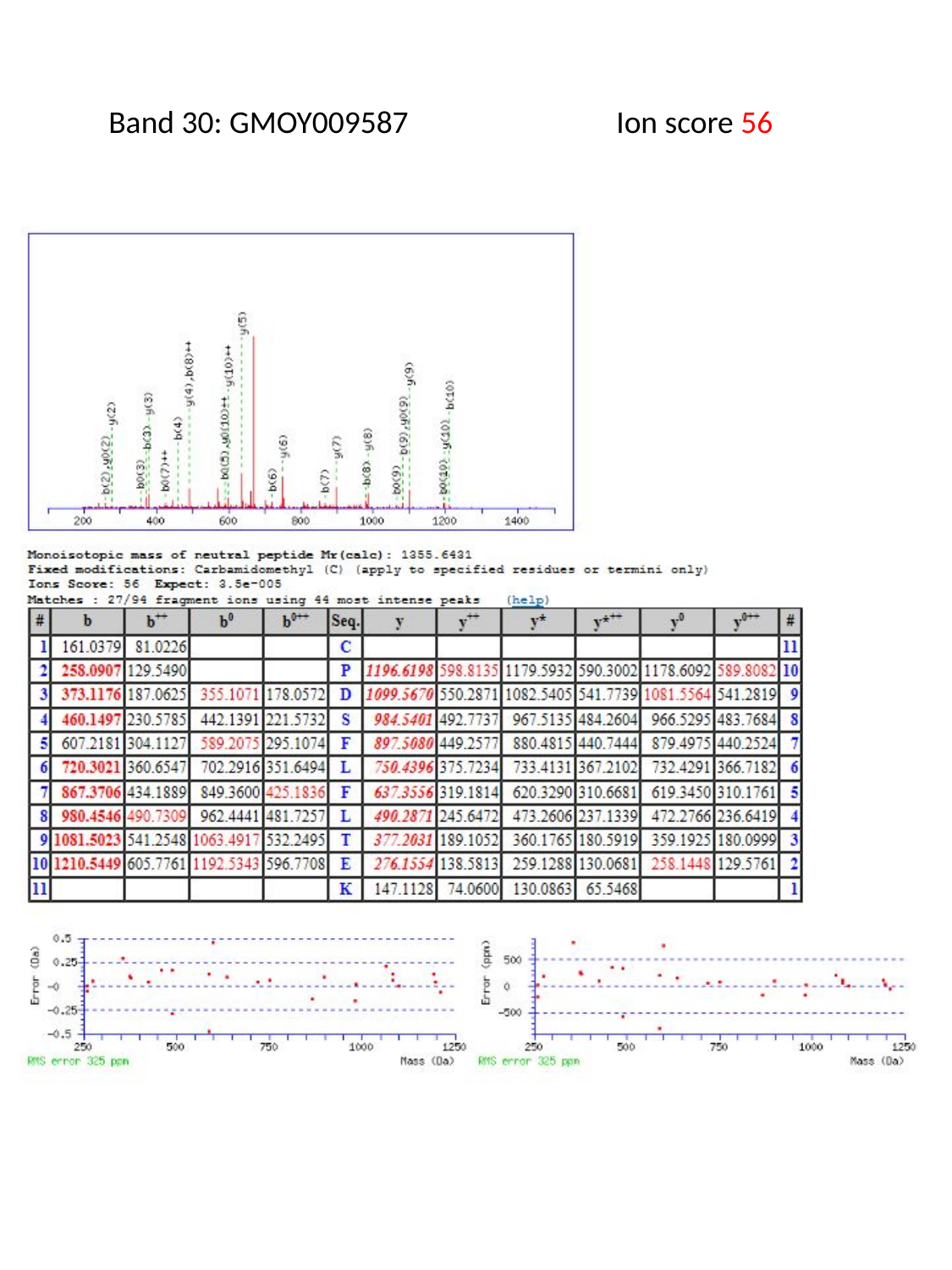

Band 30: GMOY009587 		Ion score 56

## Slide 176
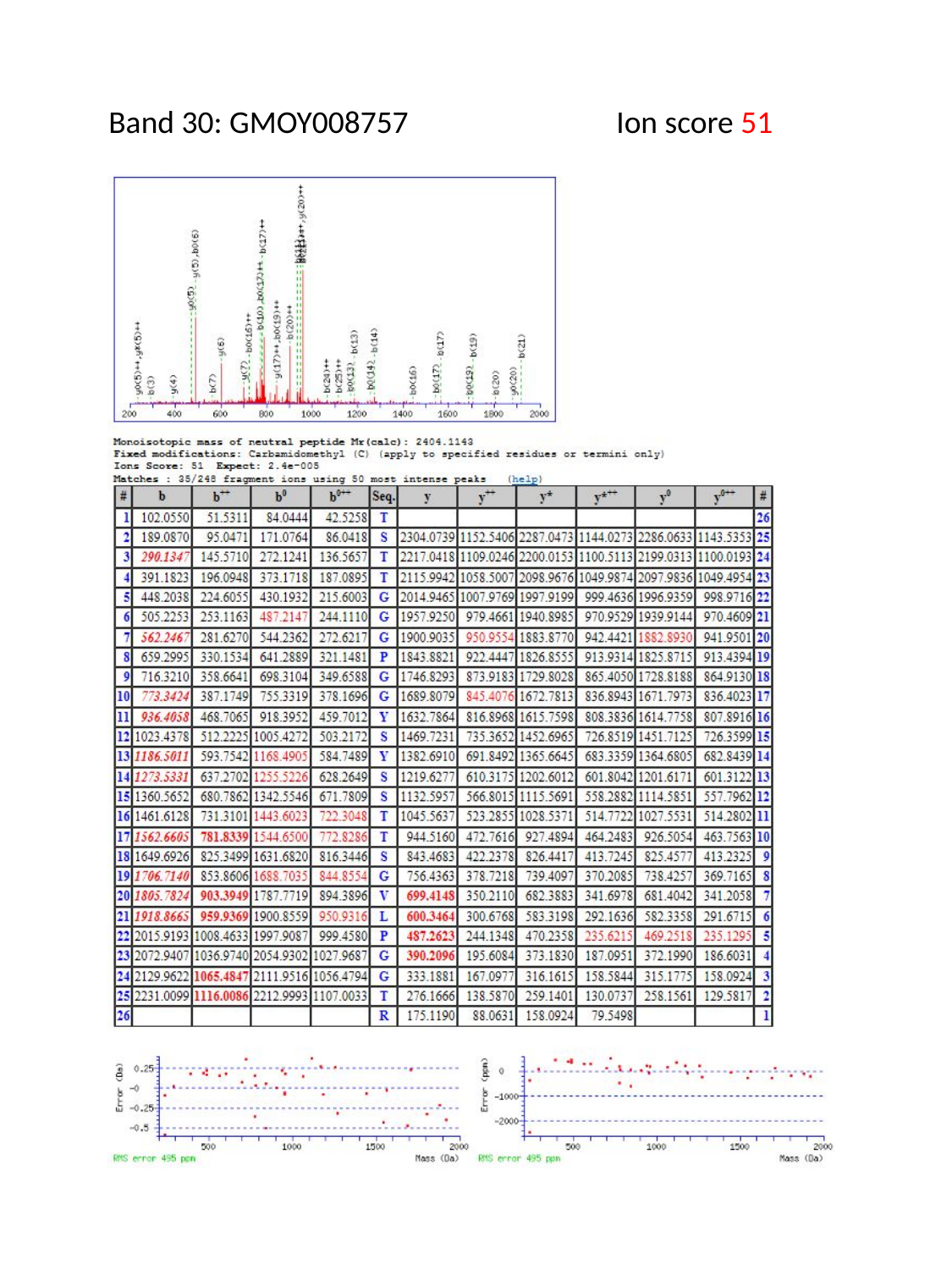

Band 30: GMOY008757 		Ion score 51

## Slide 177
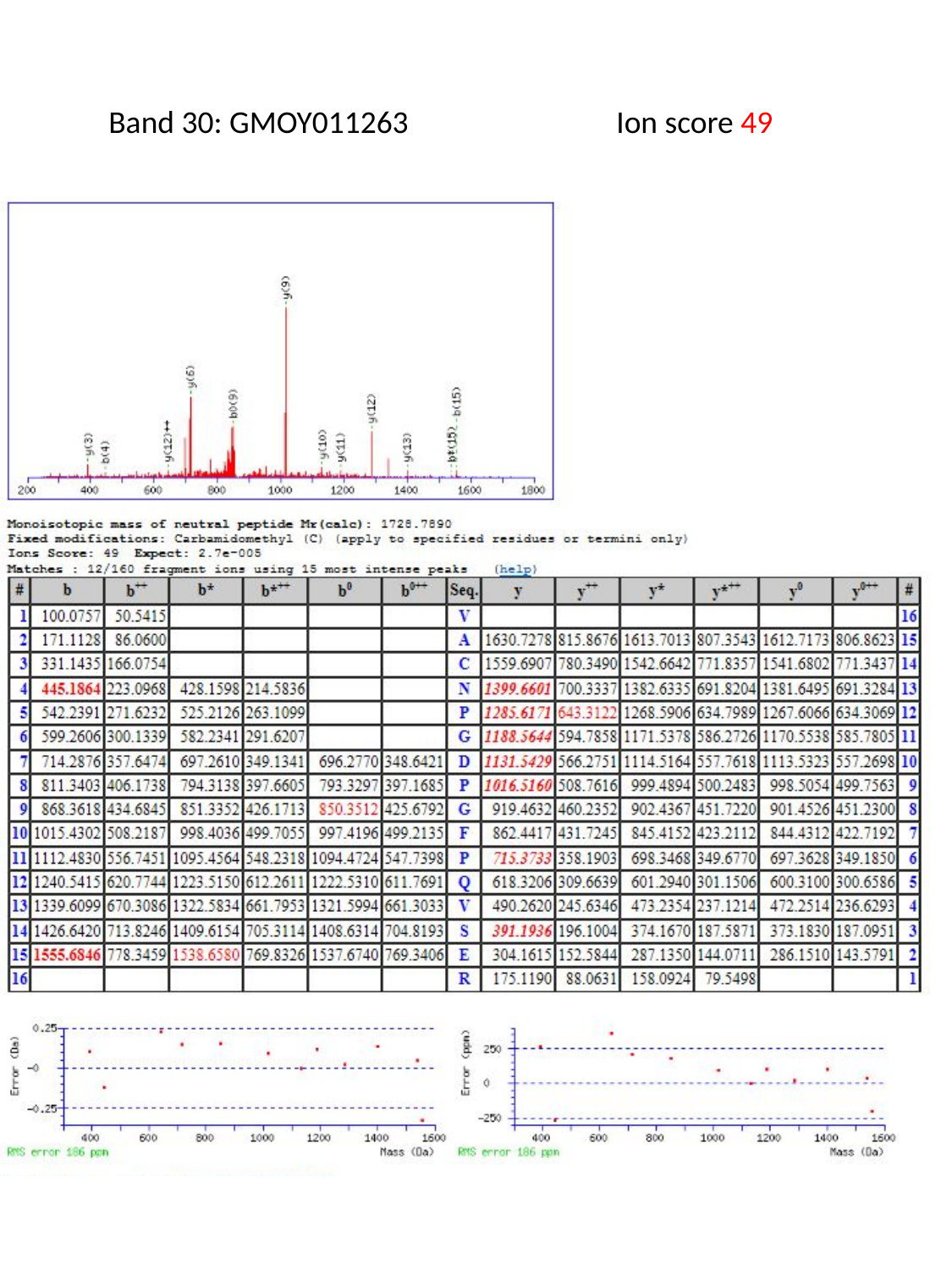

Band 30: GMOY011263 		Ion score 49

## Slide 178
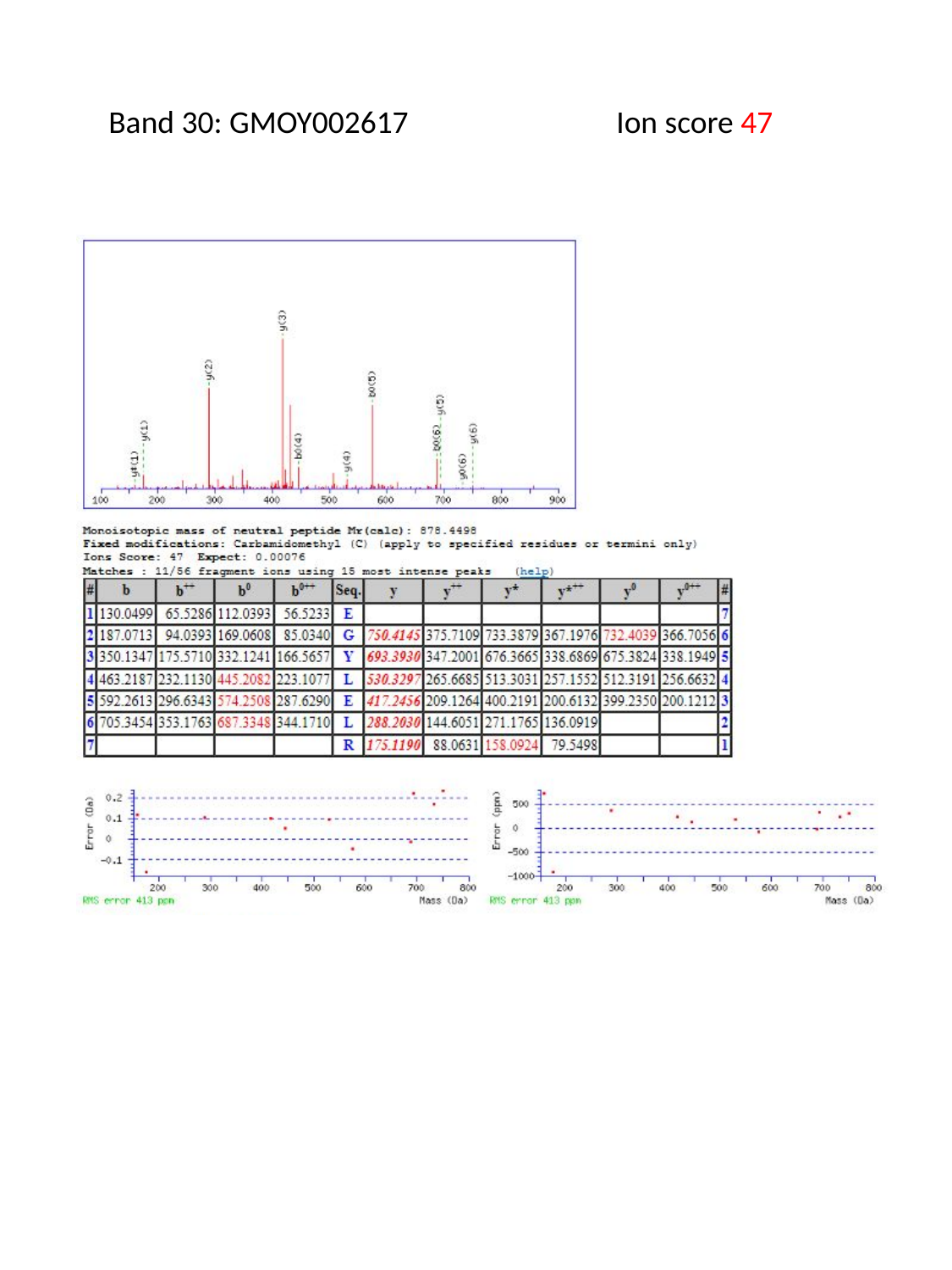

Band 30: GMOY002617 		Ion score 47

## Slide 179
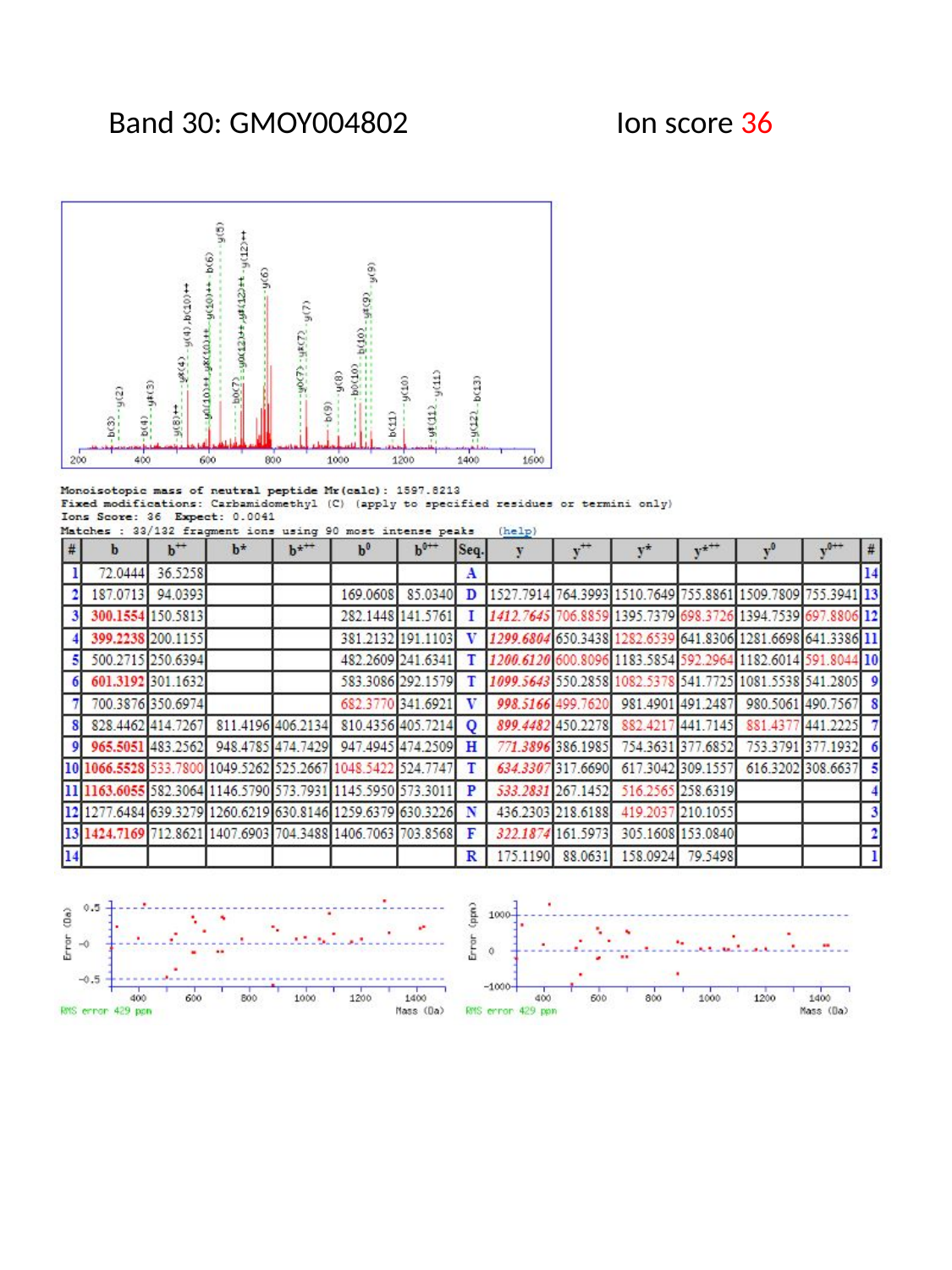

Band 30: GMOY004802 		Ion score 36

## Slide 180
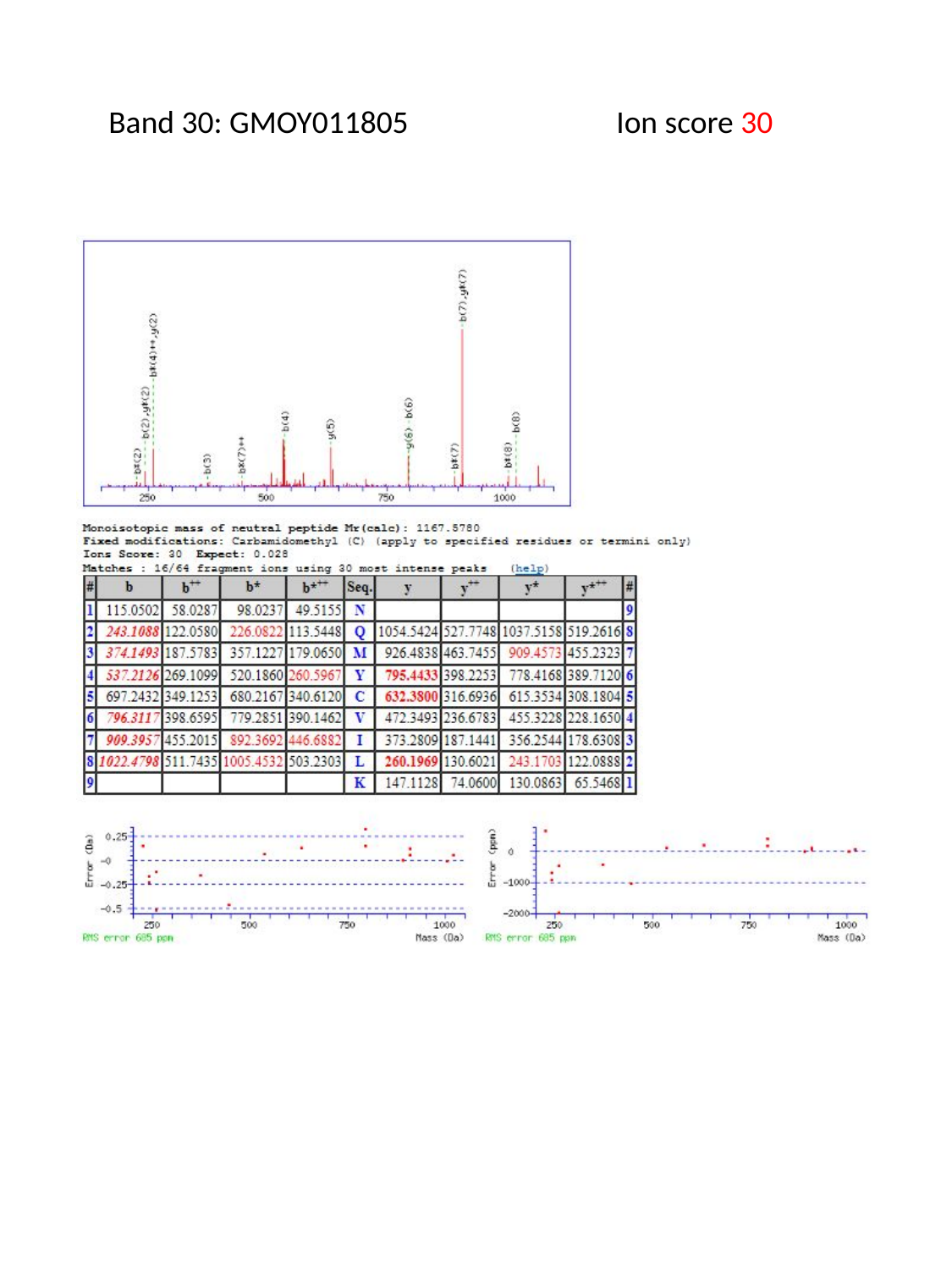

Band 30: GMOY011805 		Ion score 30
