# Supplementary material for: An Investigation into the Protein Composition of the Teneral Glossina morsitans morsitans Peritrophic Matrix
Source: PLoS Negl Trop Dis. 2014 Apr 24;8(4):e2691. doi: 10.1371/journal.pntd.0002691 (PMC3998921; doi:10.1371/journal.pntd.0002691)
Supplement: Figure S2 — In-solution single peptide hit validation. (PPT) [file pntd.0002691.s002.ppt]

## Slide 1
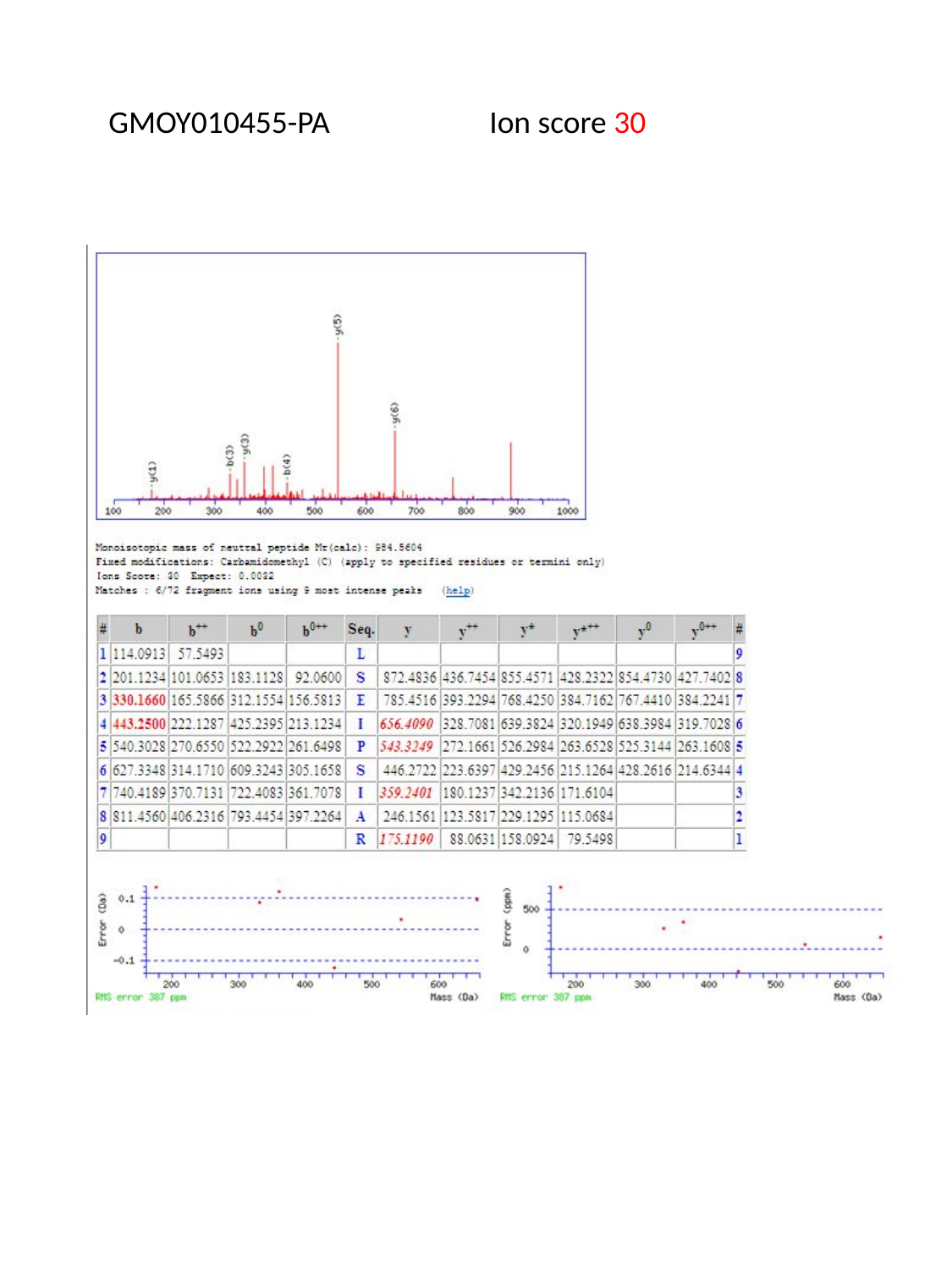

GMOY010455-PA		Ion score 30

## Slide 2
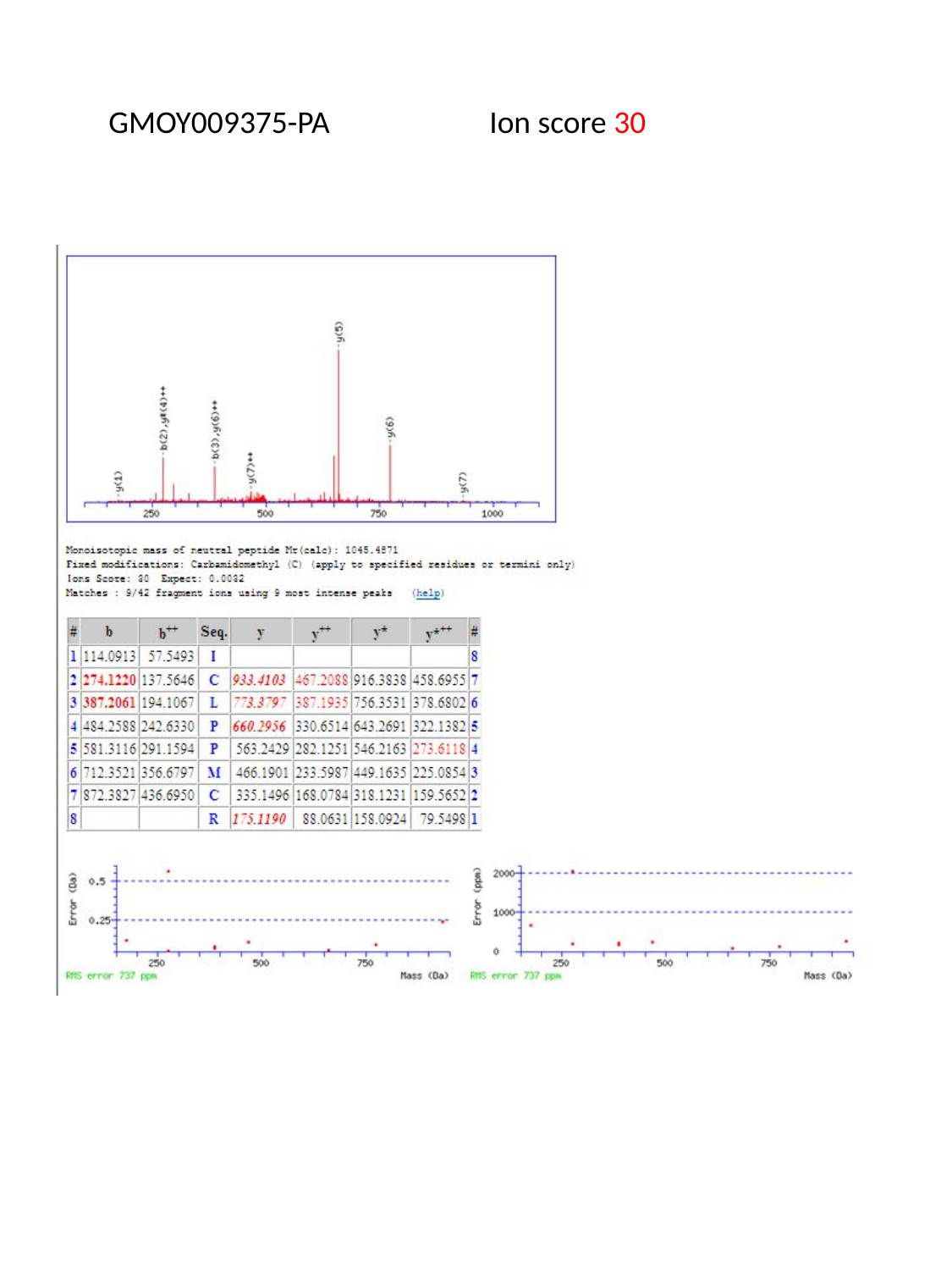

GMOY009375-PA		Ion score 30

## Slide 3
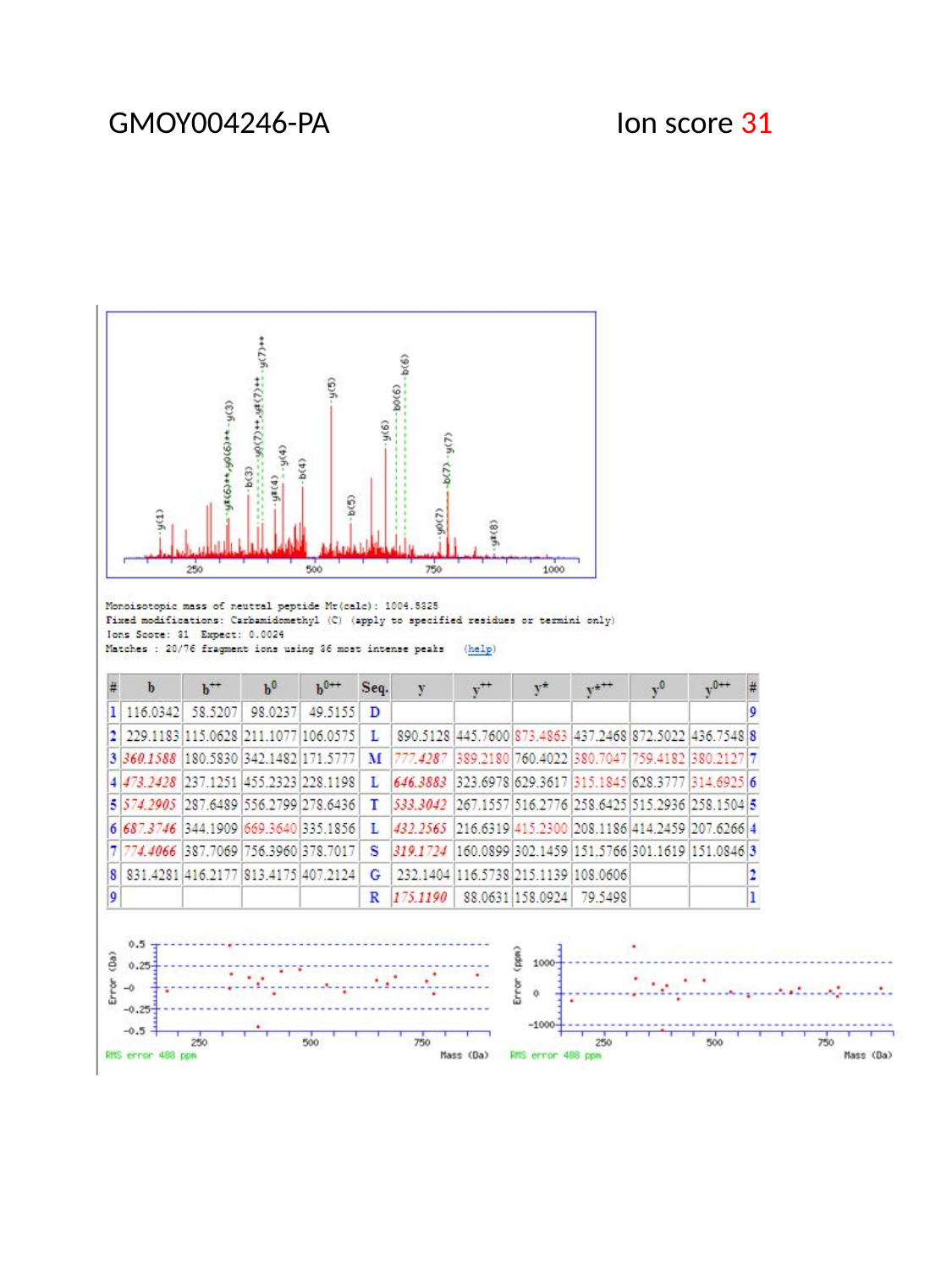

GMOY004246-PA			Ion score 31

## Slide 4
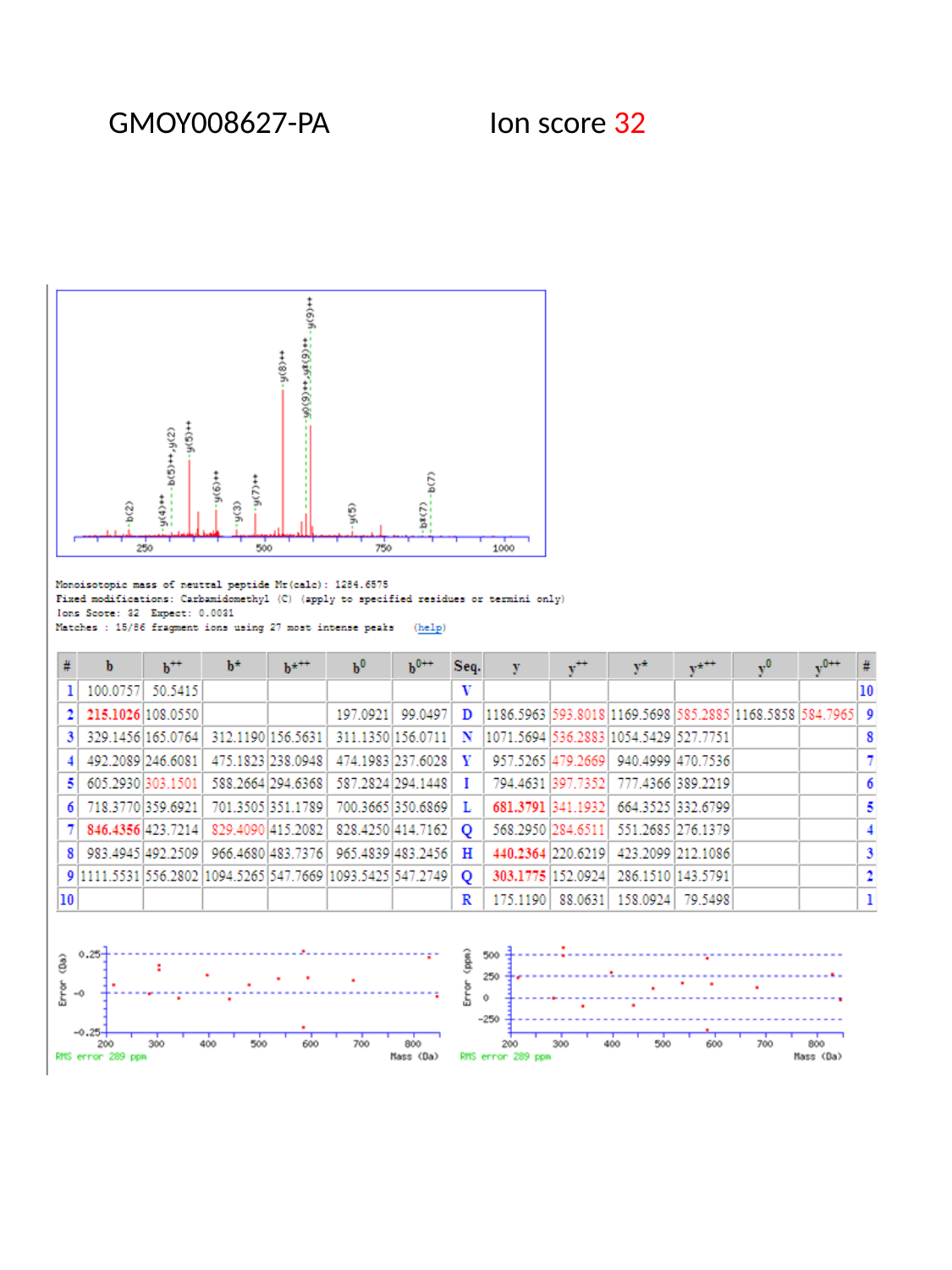

GMOY008627-PA 		Ion score 32

## Slide 5
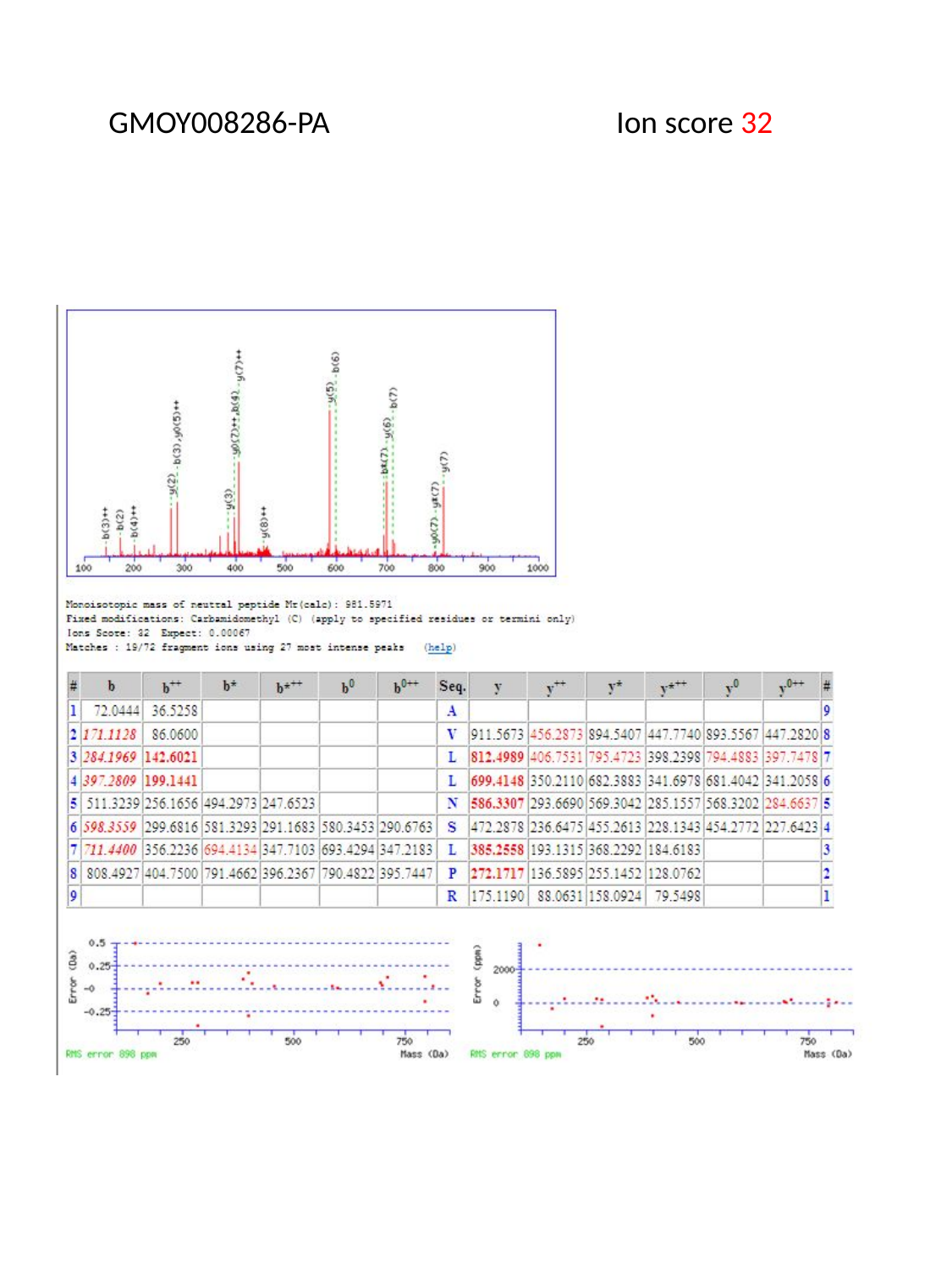

GMOY008286-PA 			Ion score 32

## Slide 6
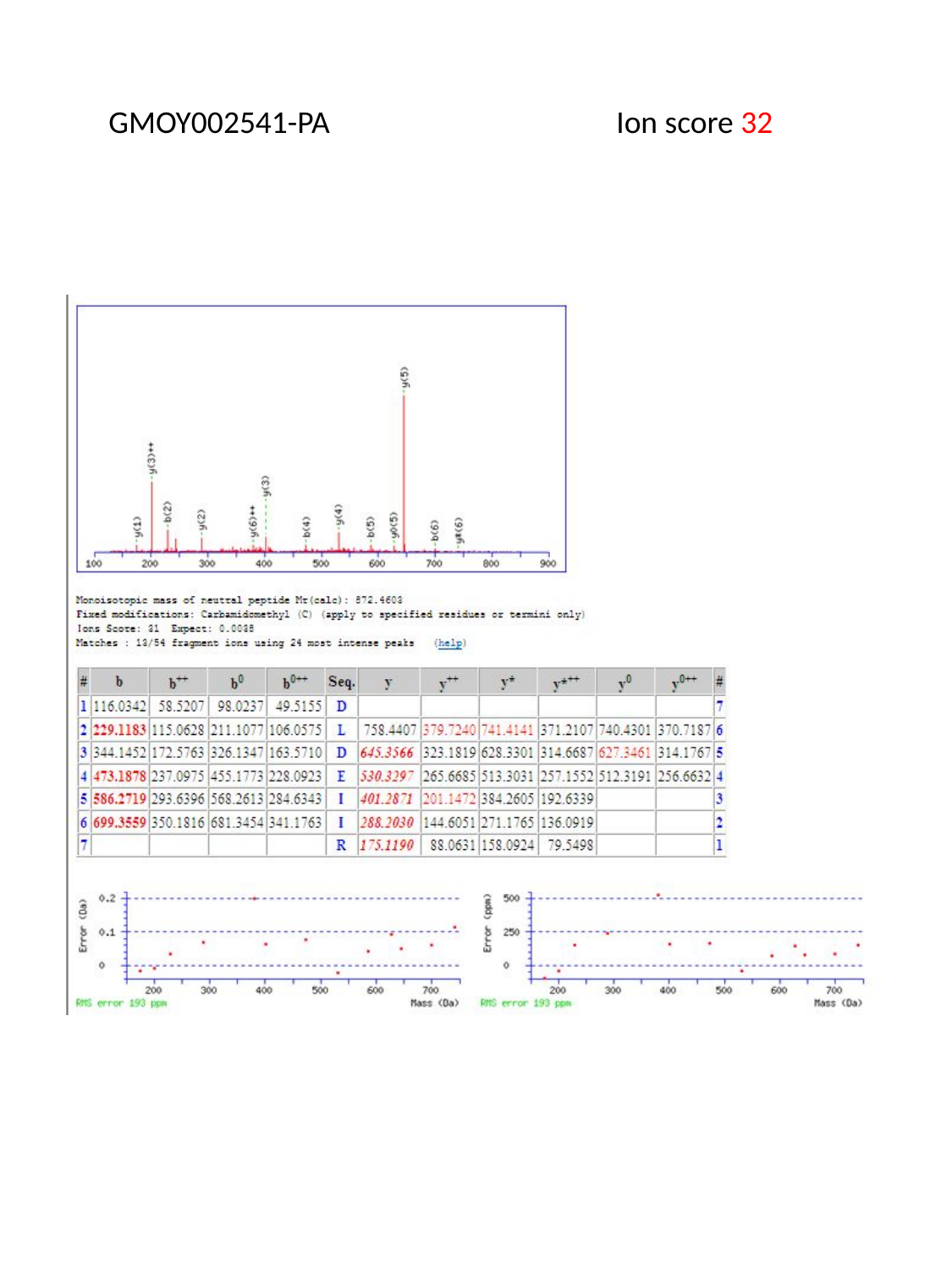

GMOY002541-PA 			Ion score 32

## Slide 7
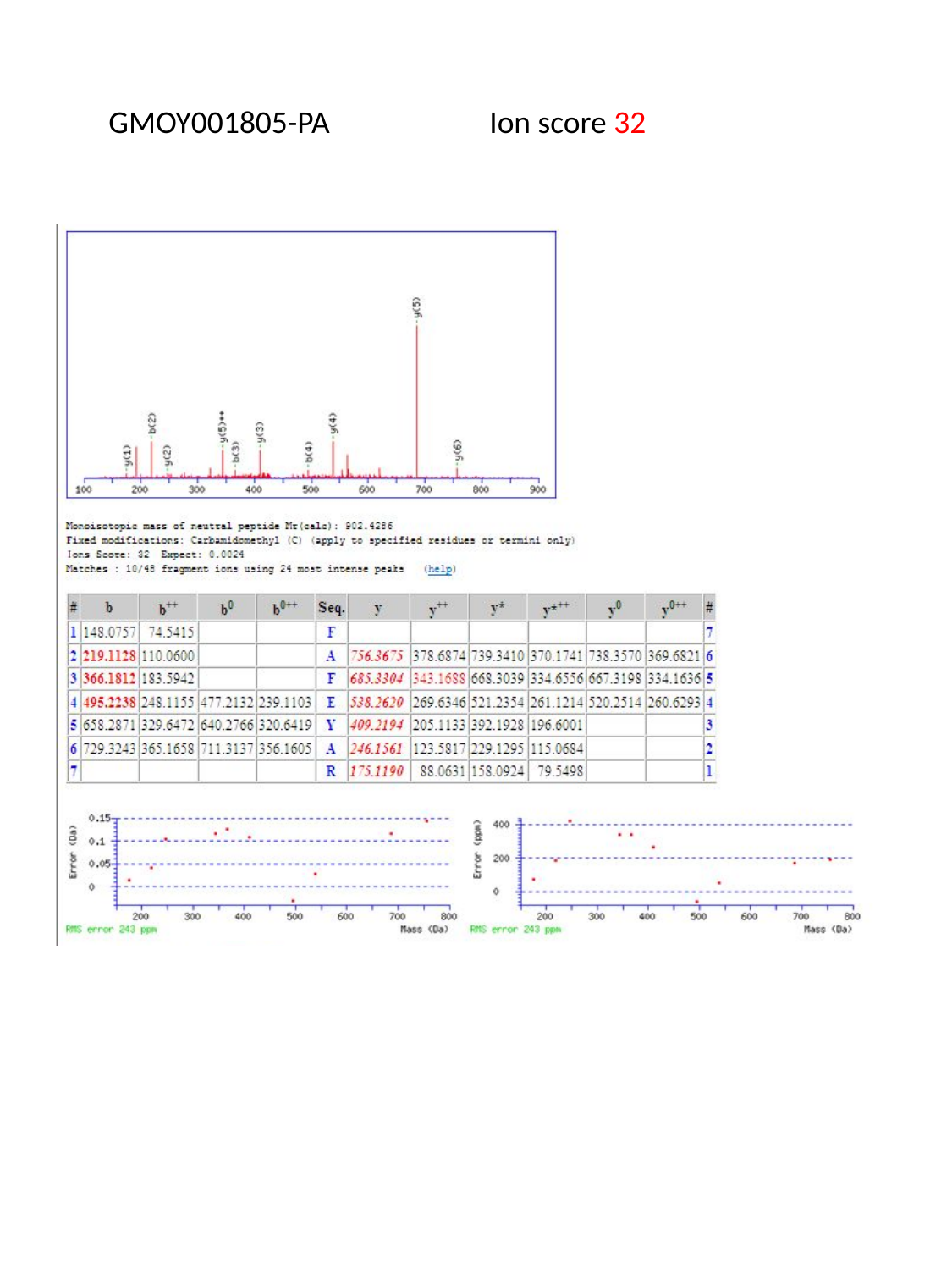

GMOY001805-PA 		Ion score 32

## Slide 8
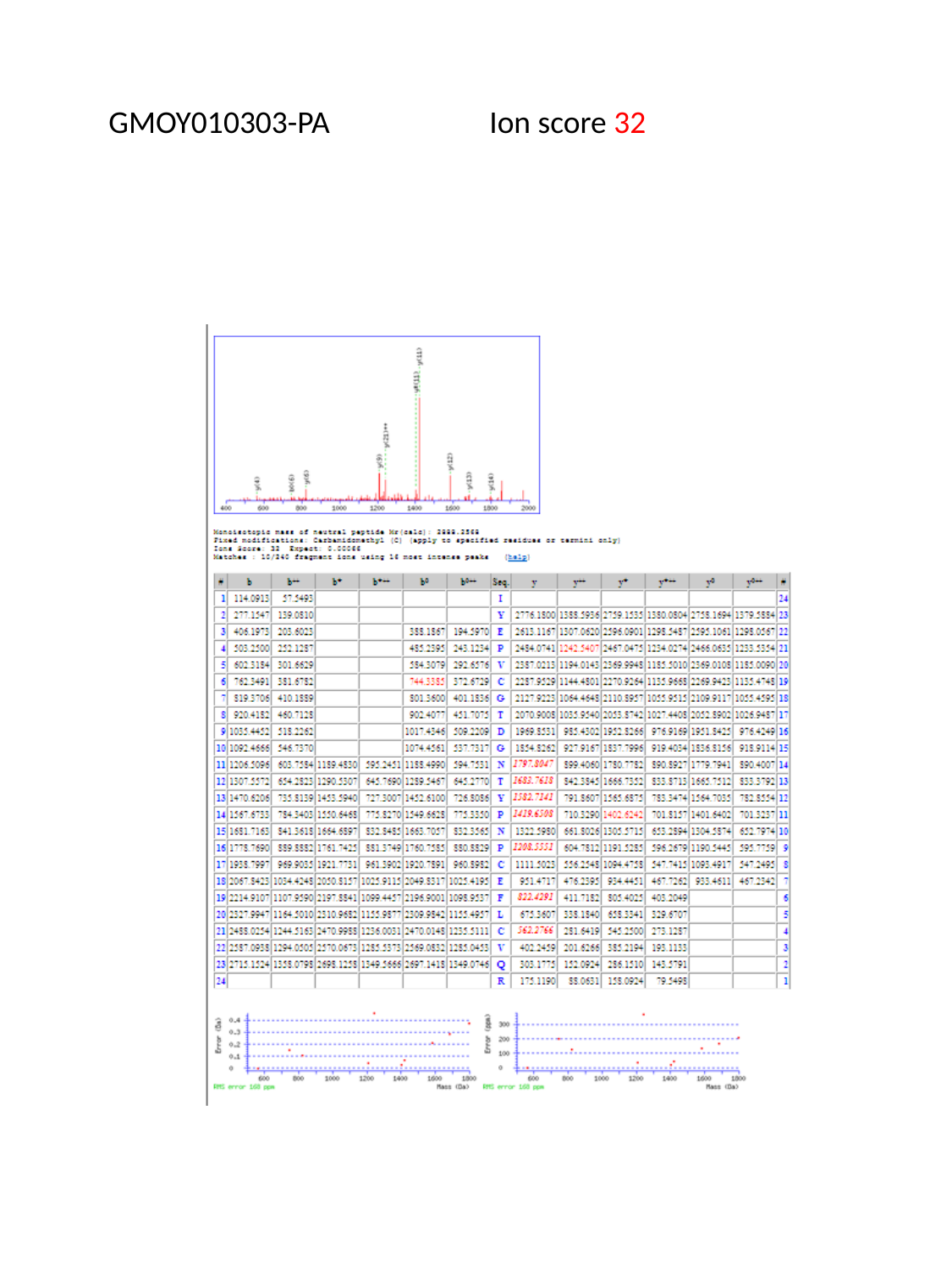

GMOY010303-PA 		Ion score 32

## Slide 9
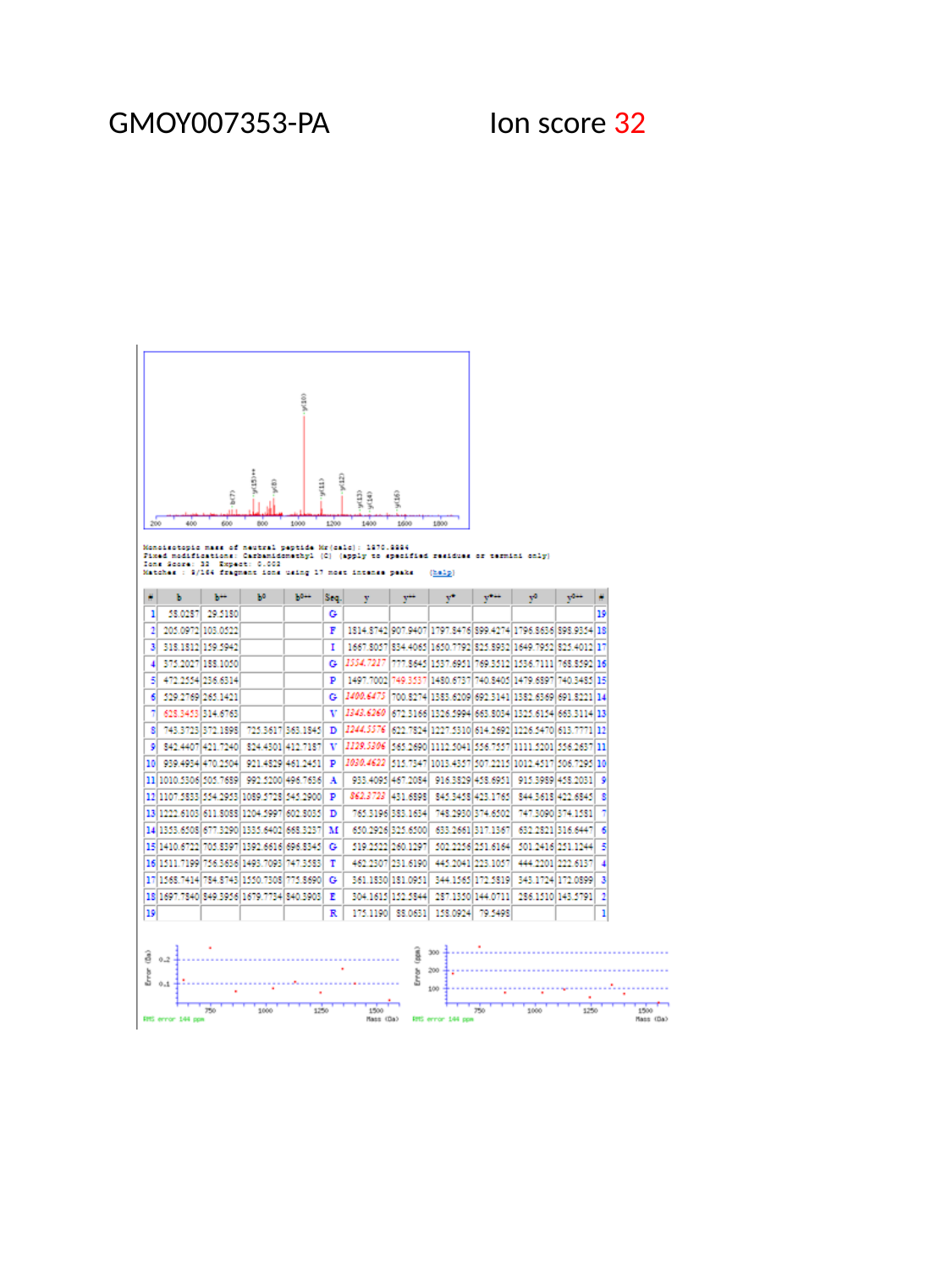

GMOY007353-PA 		Ion score 32

## Slide 10
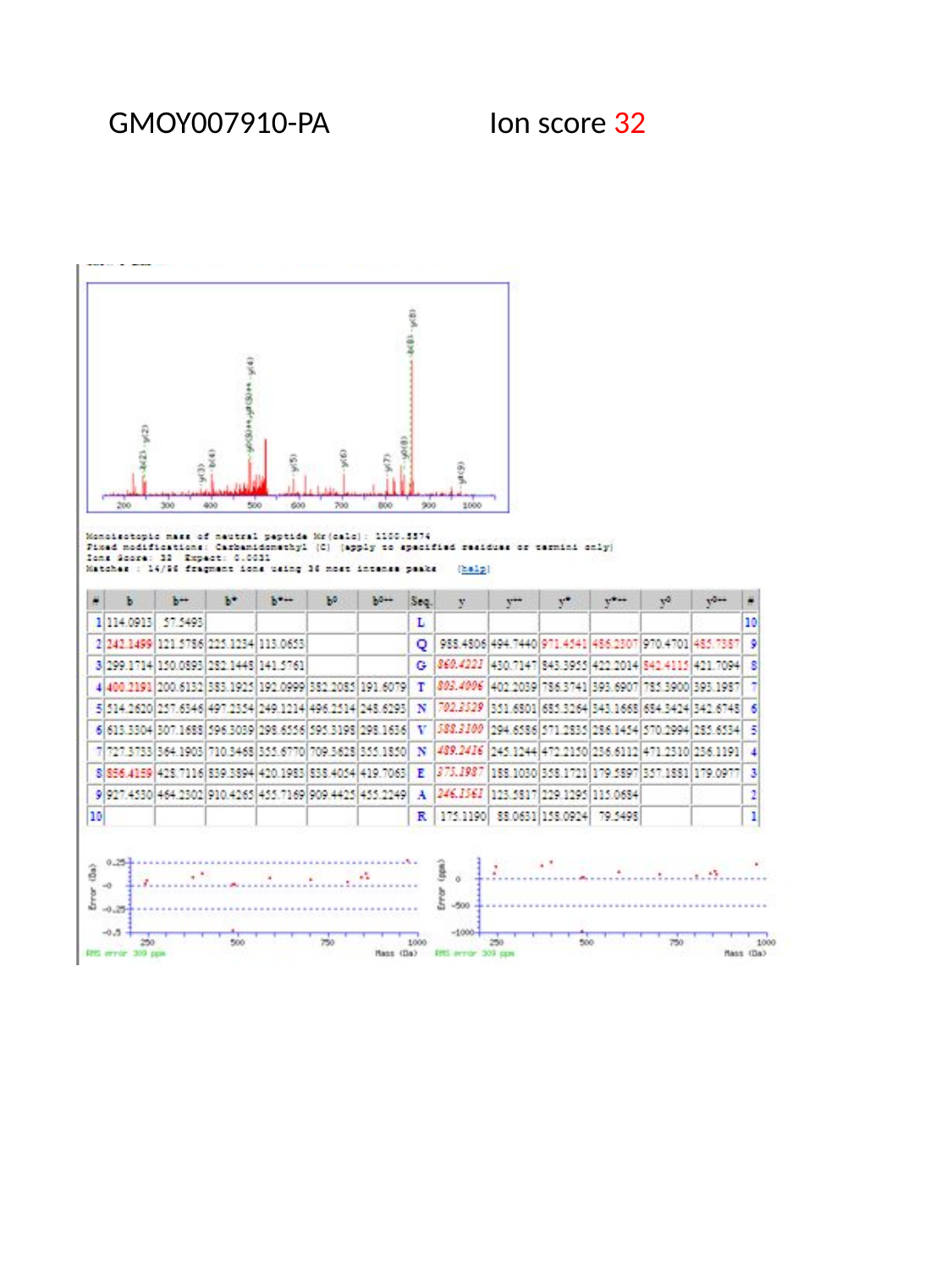

GMOY007910-PA 		Ion score 32

## Slide 11
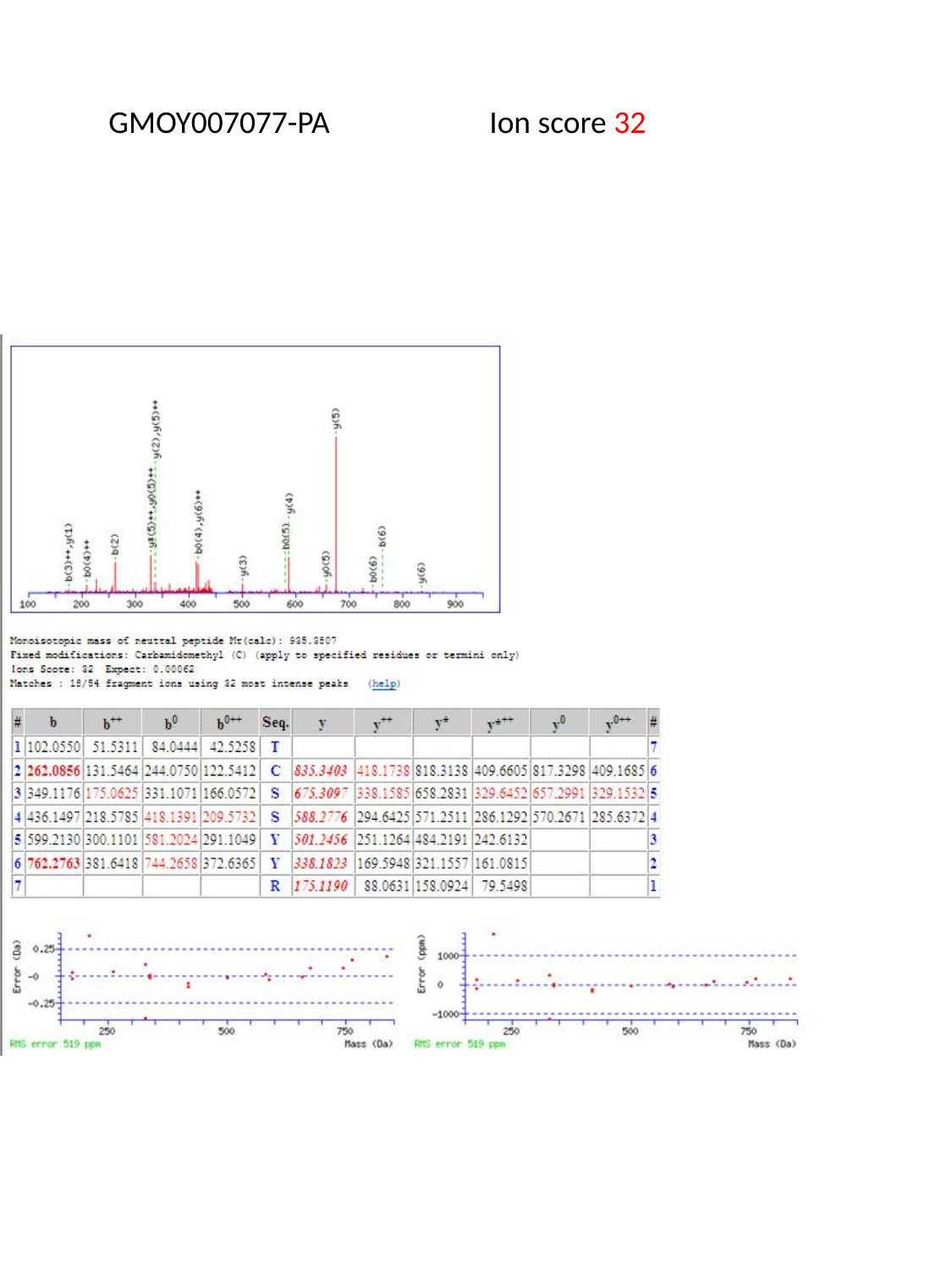

GMOY007077-PA 		Ion score 32

## Slide 12
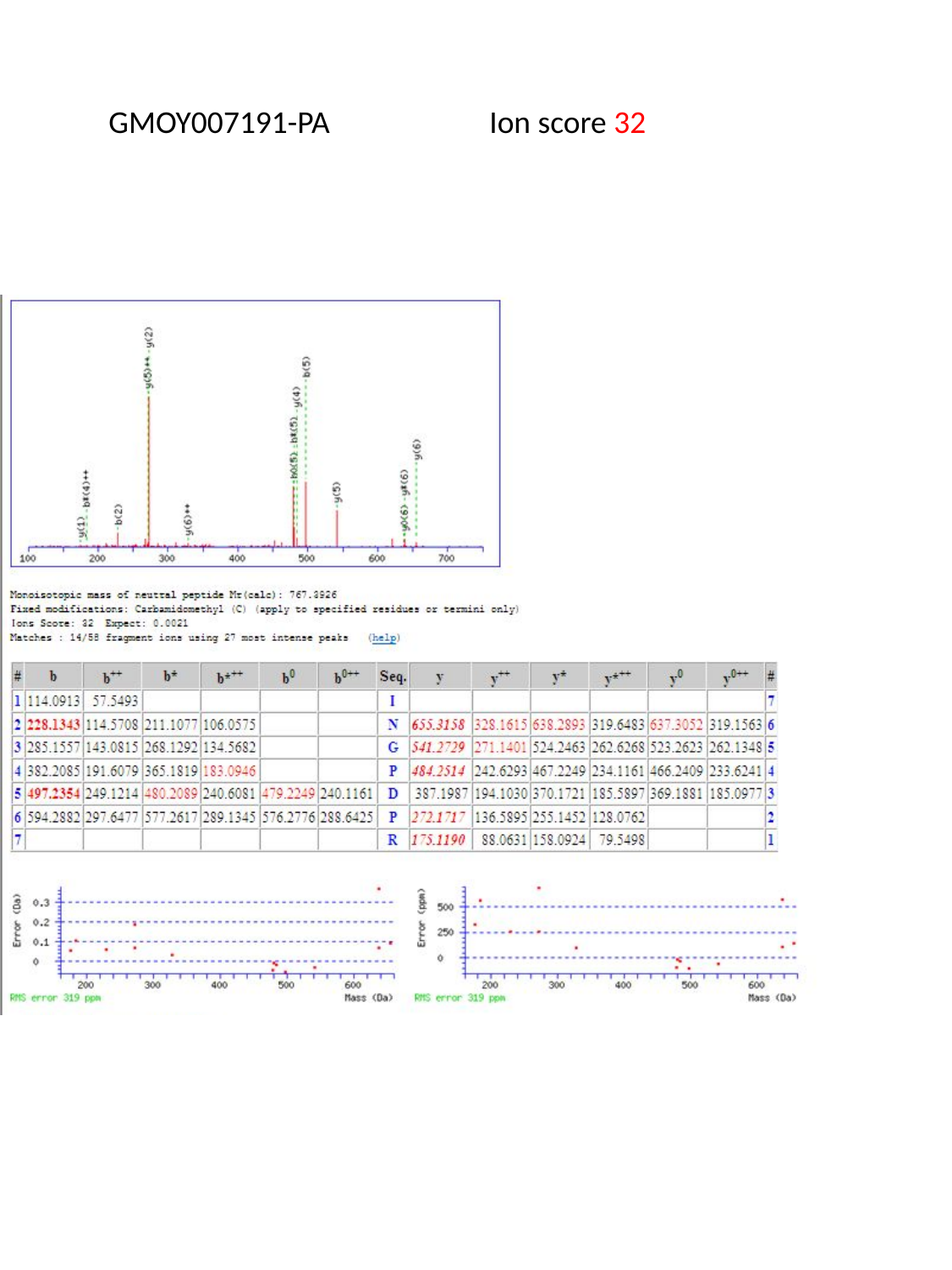

GMOY007191-PA 		Ion score 32

## Slide 13
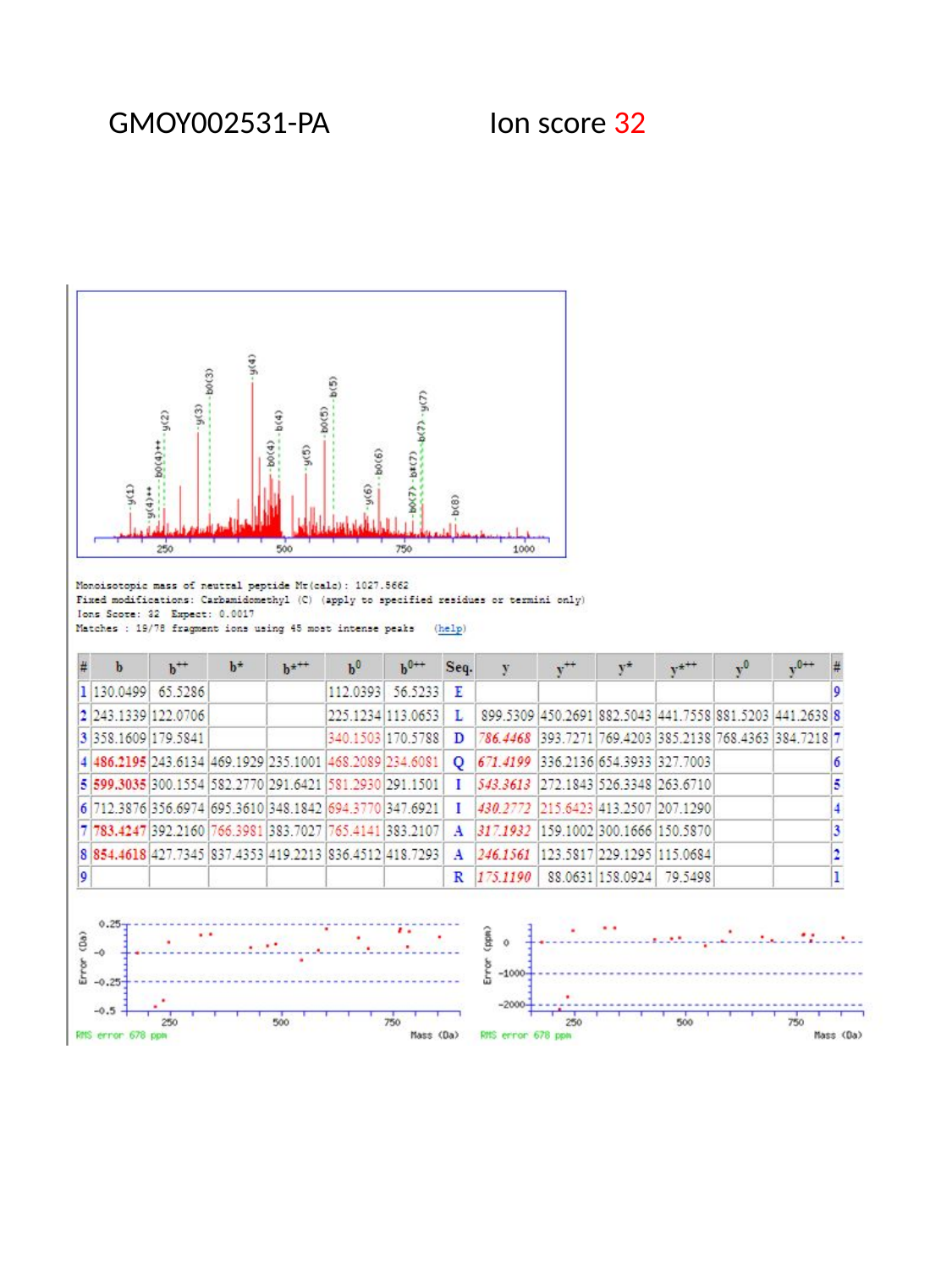

GMOY002531-PA 		Ion score 32

## Slide 14
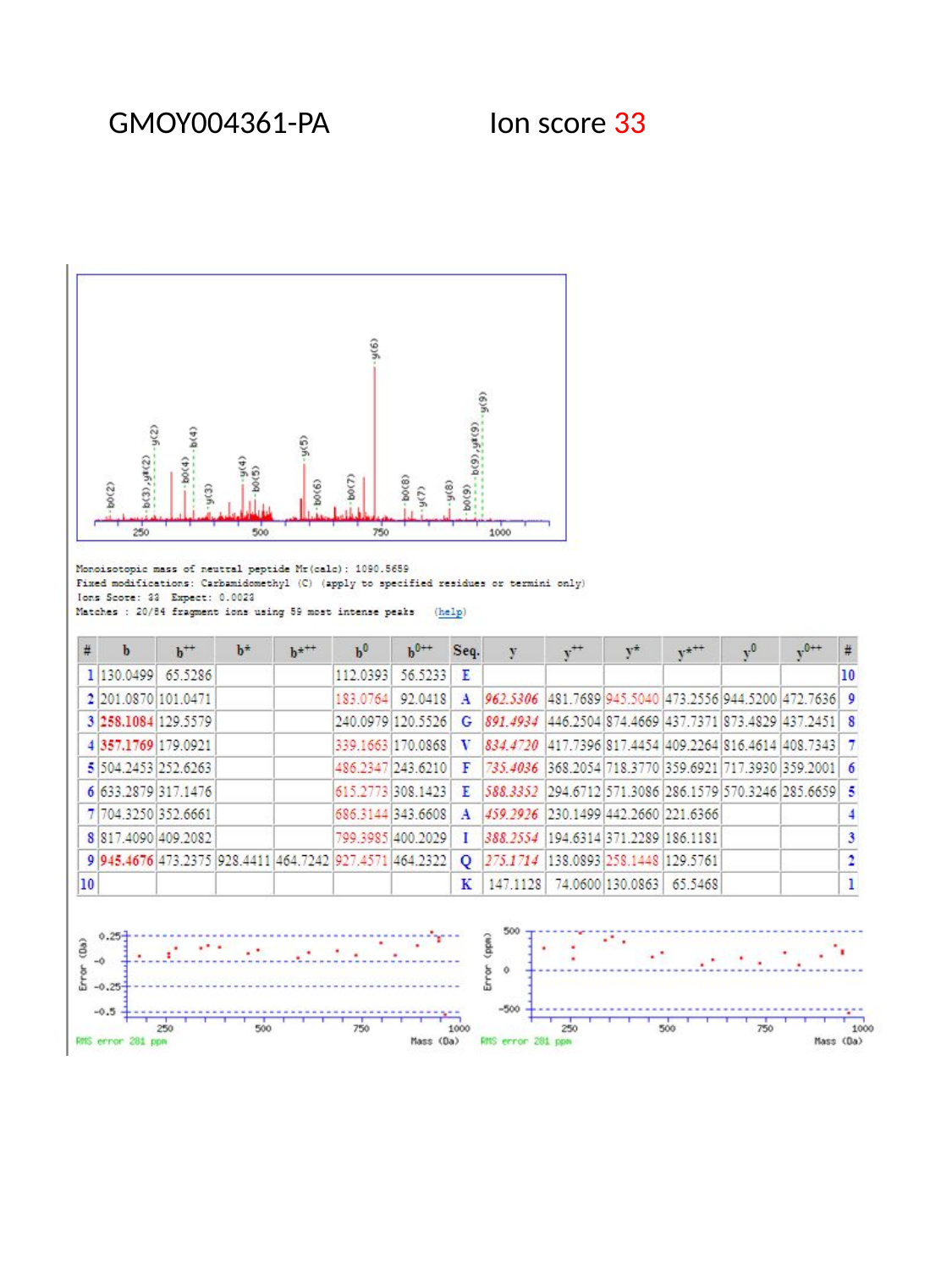

GMOY004361-PA		Ion score 33

## Slide 15
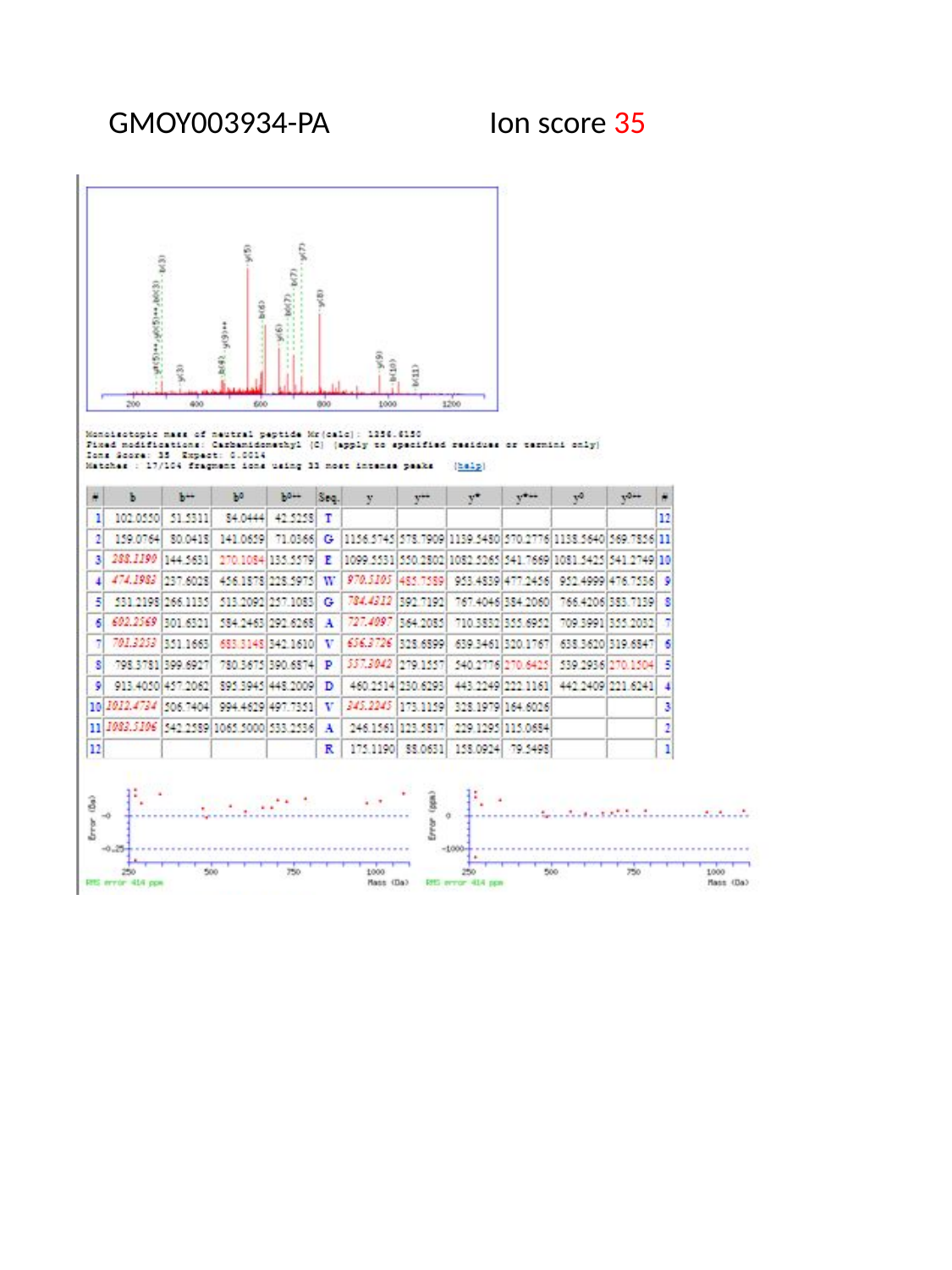

GMOY003934-PA		Ion score 35

## Slide 16
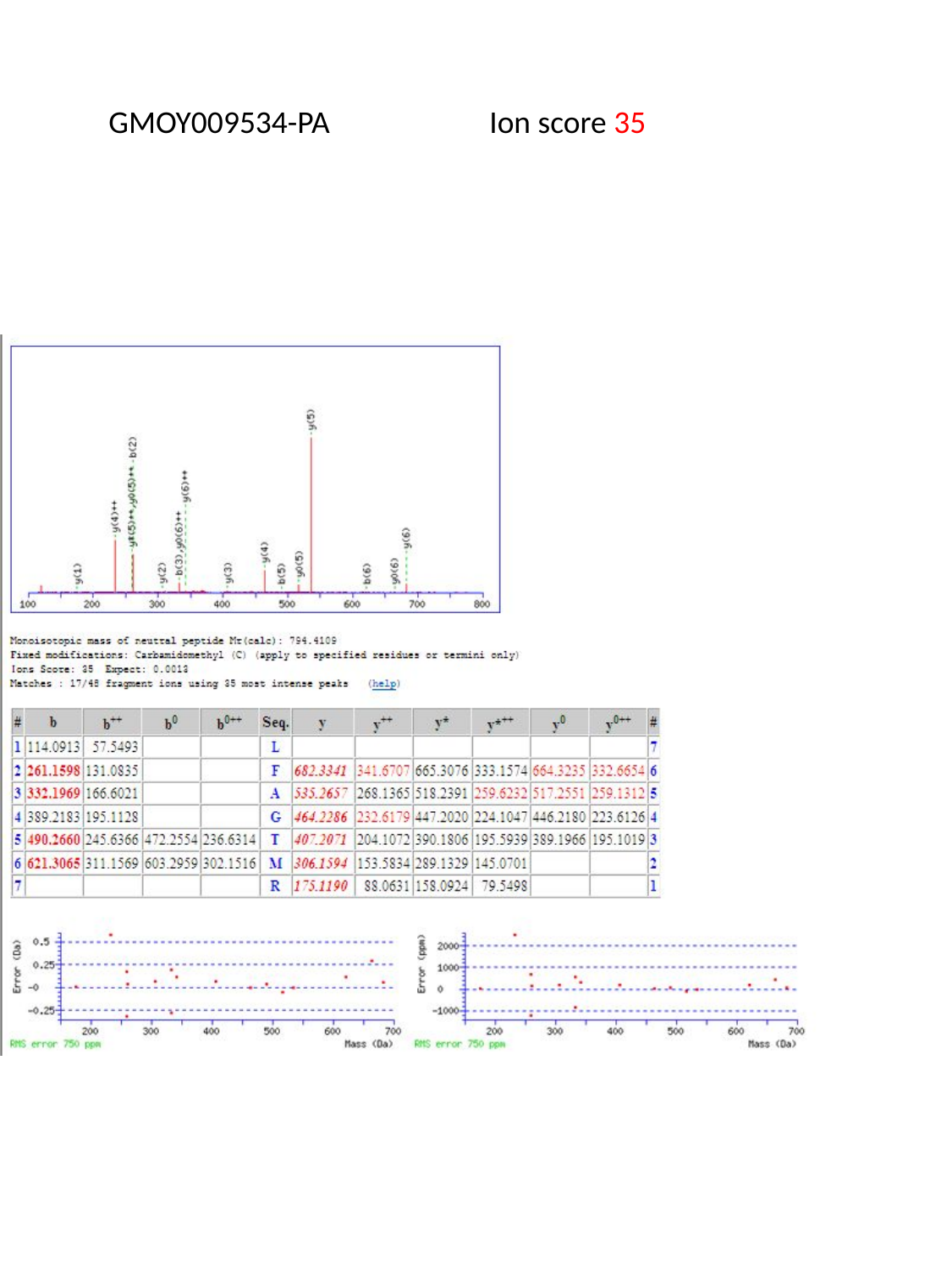

GMOY009534-PA		Ion score 35

## Slide 17
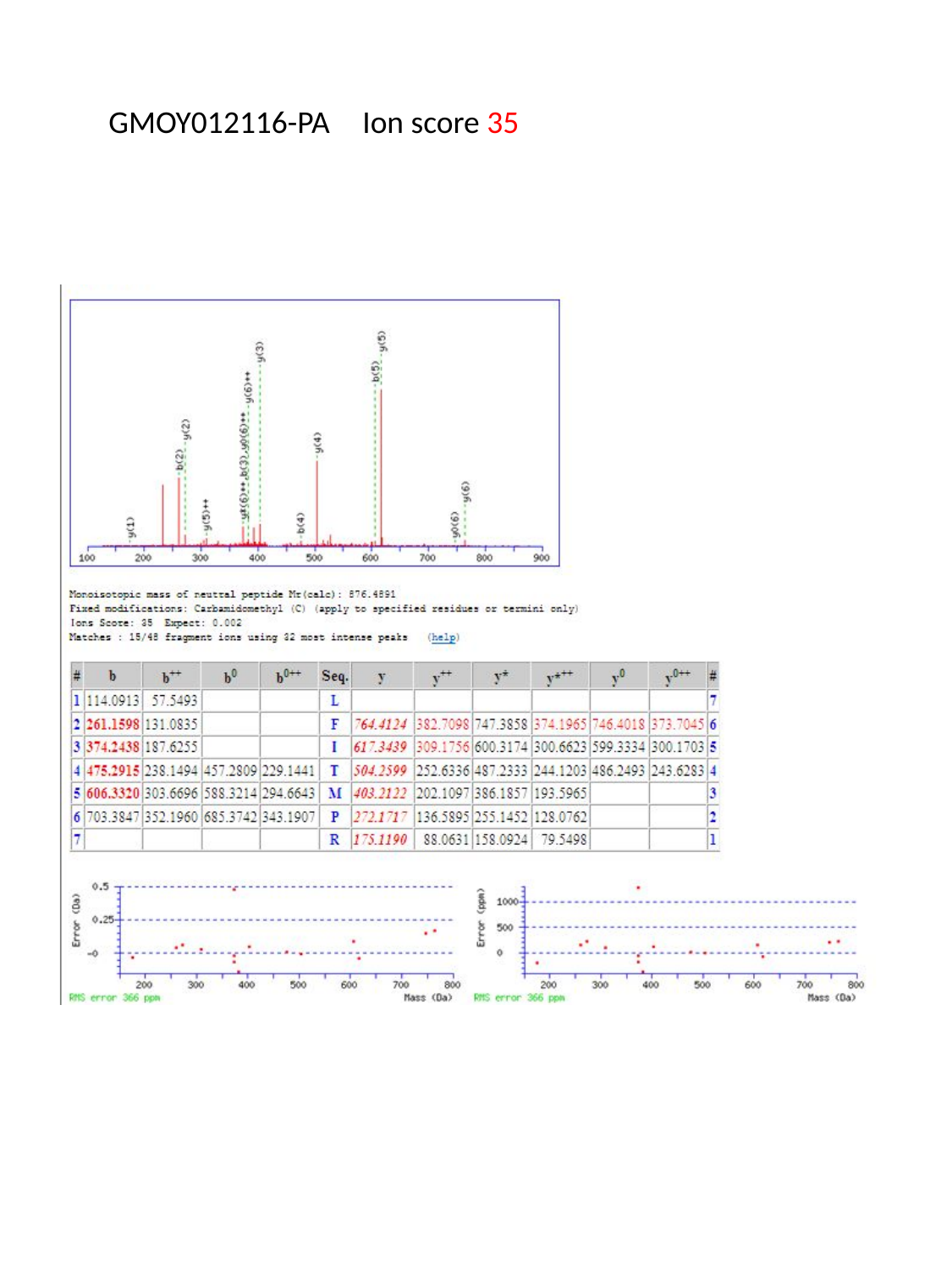

GMOY012116-PA	Ion score 35

## Slide 18
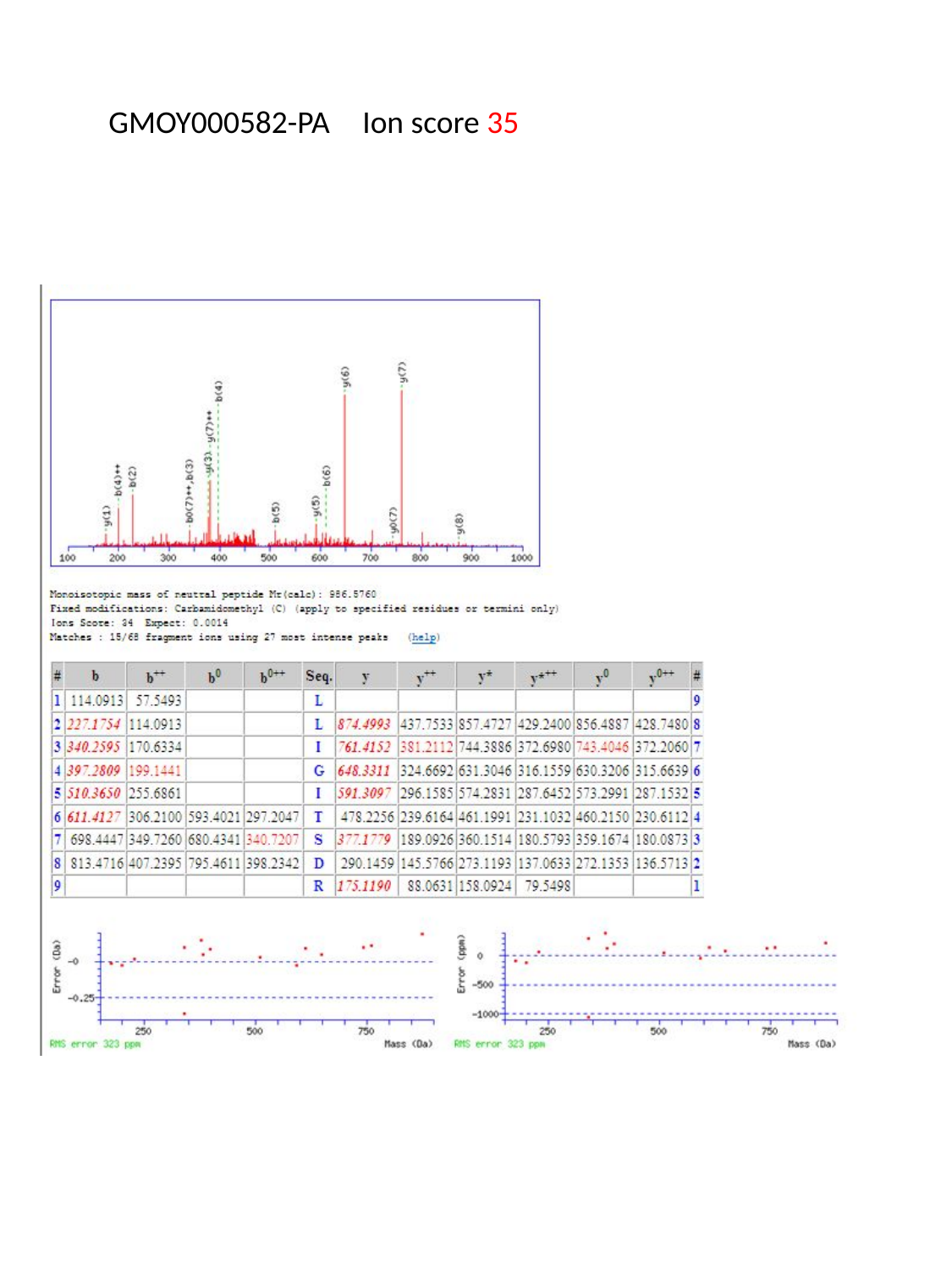

GMOY000582-PA	Ion score 35

## Slide 19
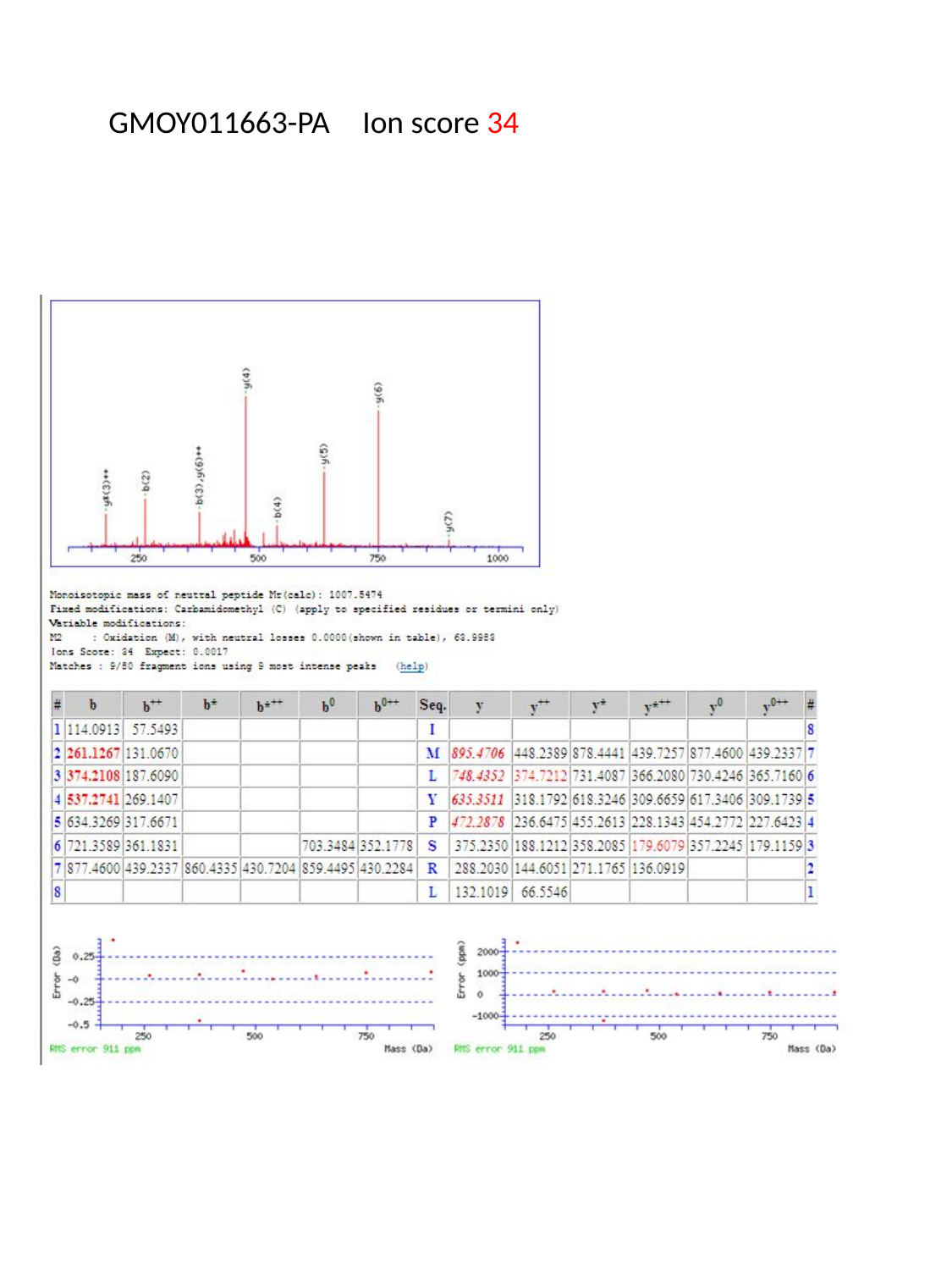

GMOY011663-PA 	Ion score 34

## Slide 20
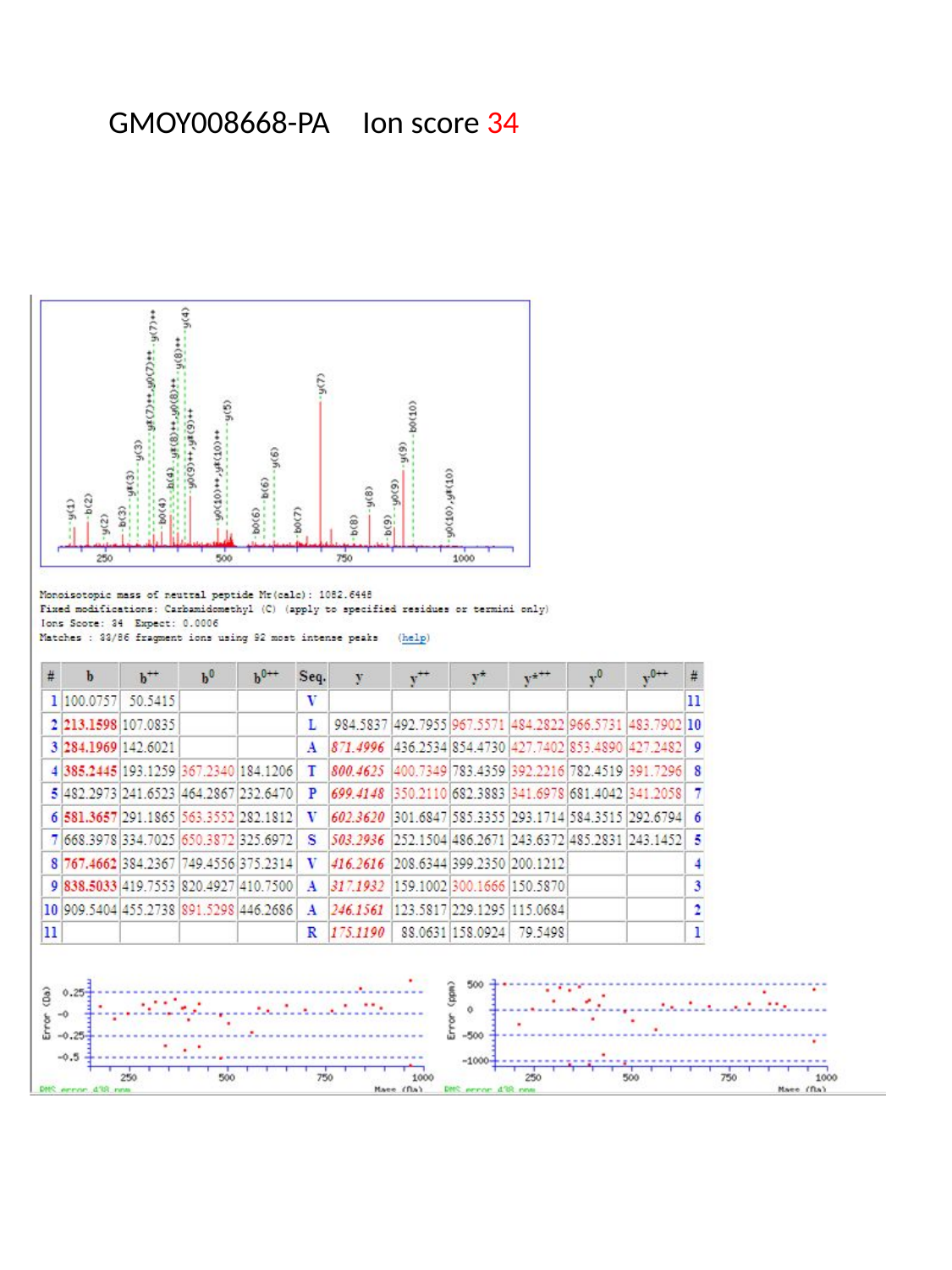

GMOY008668-PA 	Ion score 34

## Slide 21
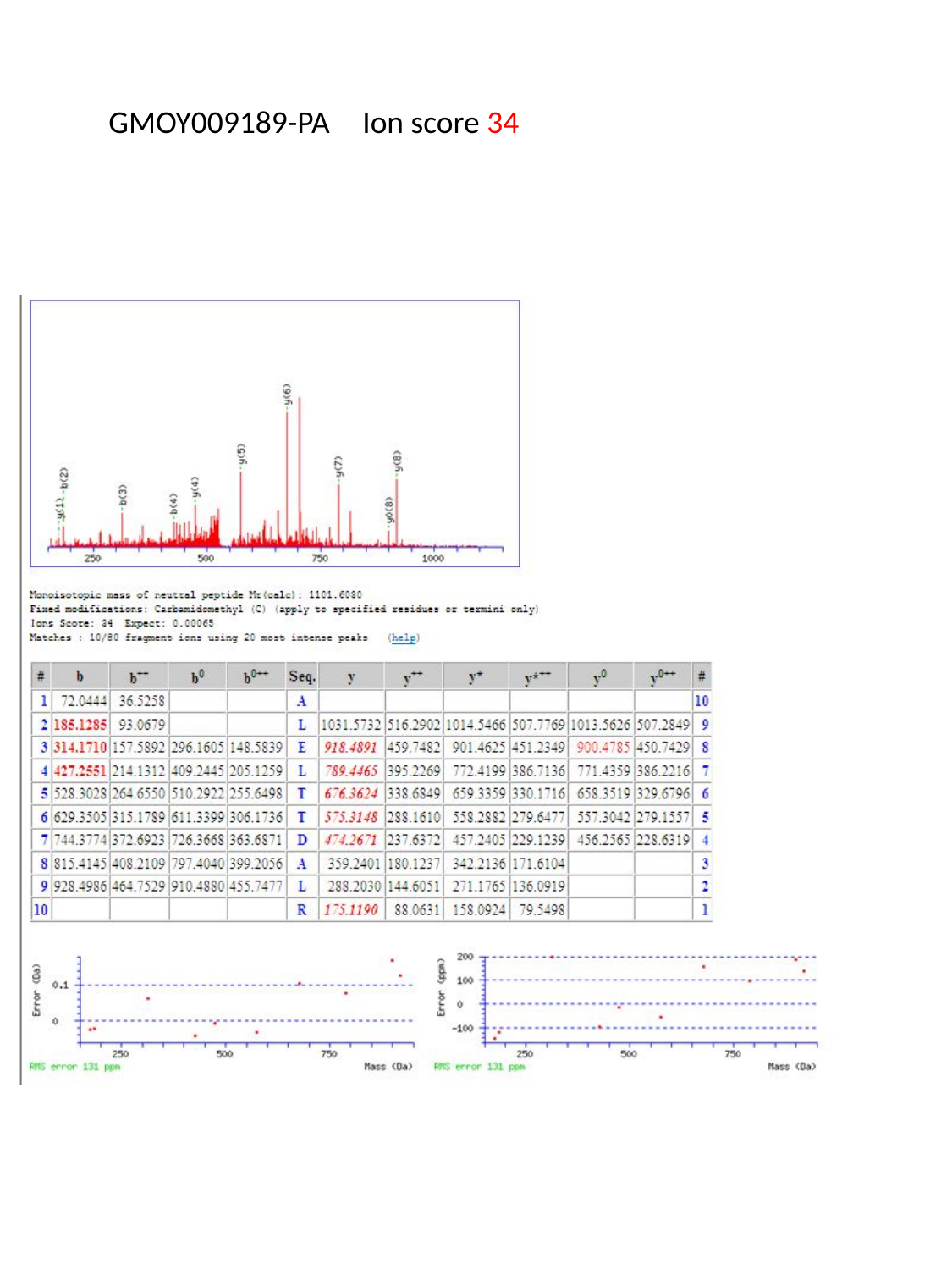

GMOY009189-PA 	Ion score 34

## Slide 22
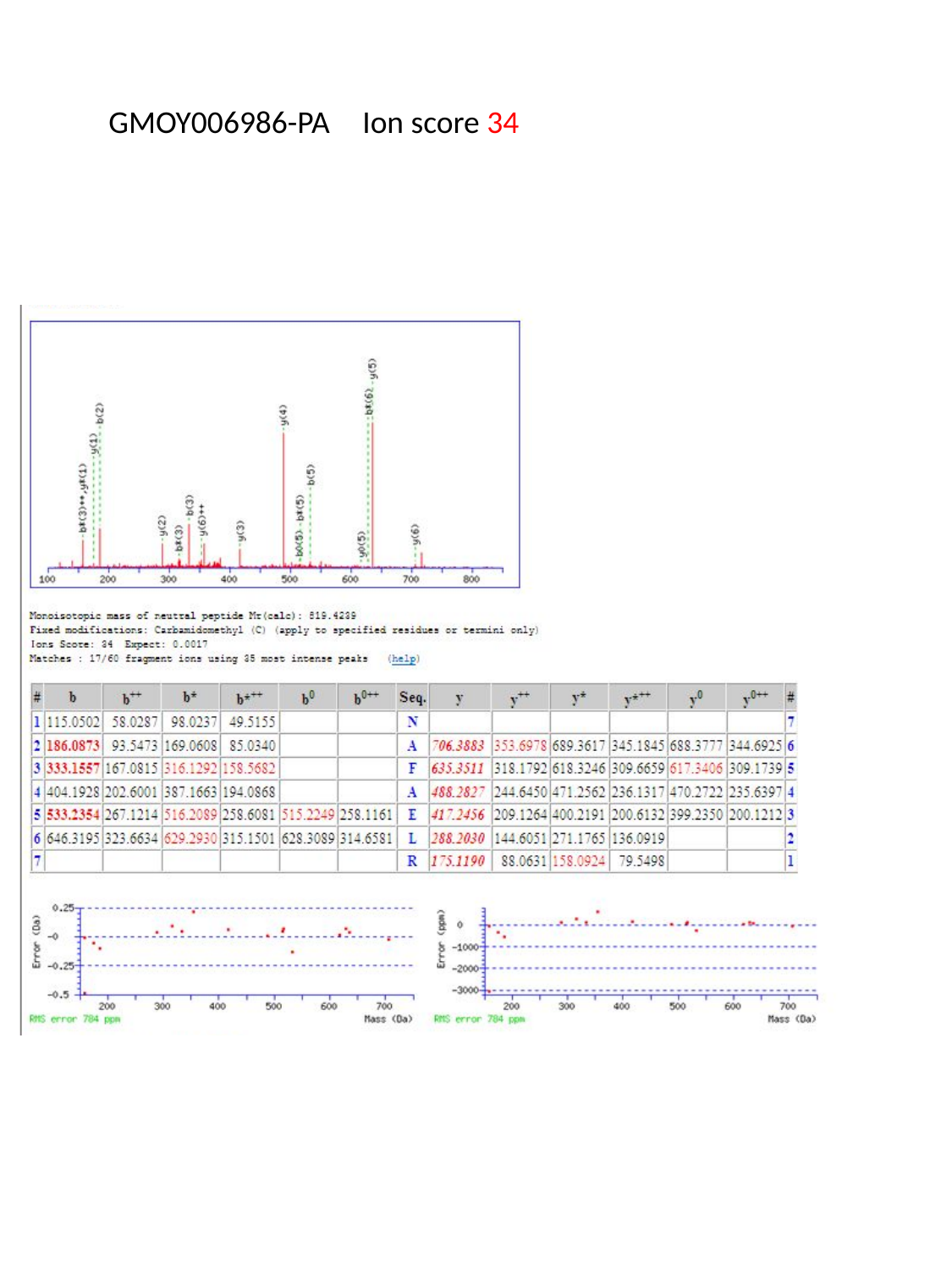

GMOY006986-PA 	Ion score 34

## Slide 23
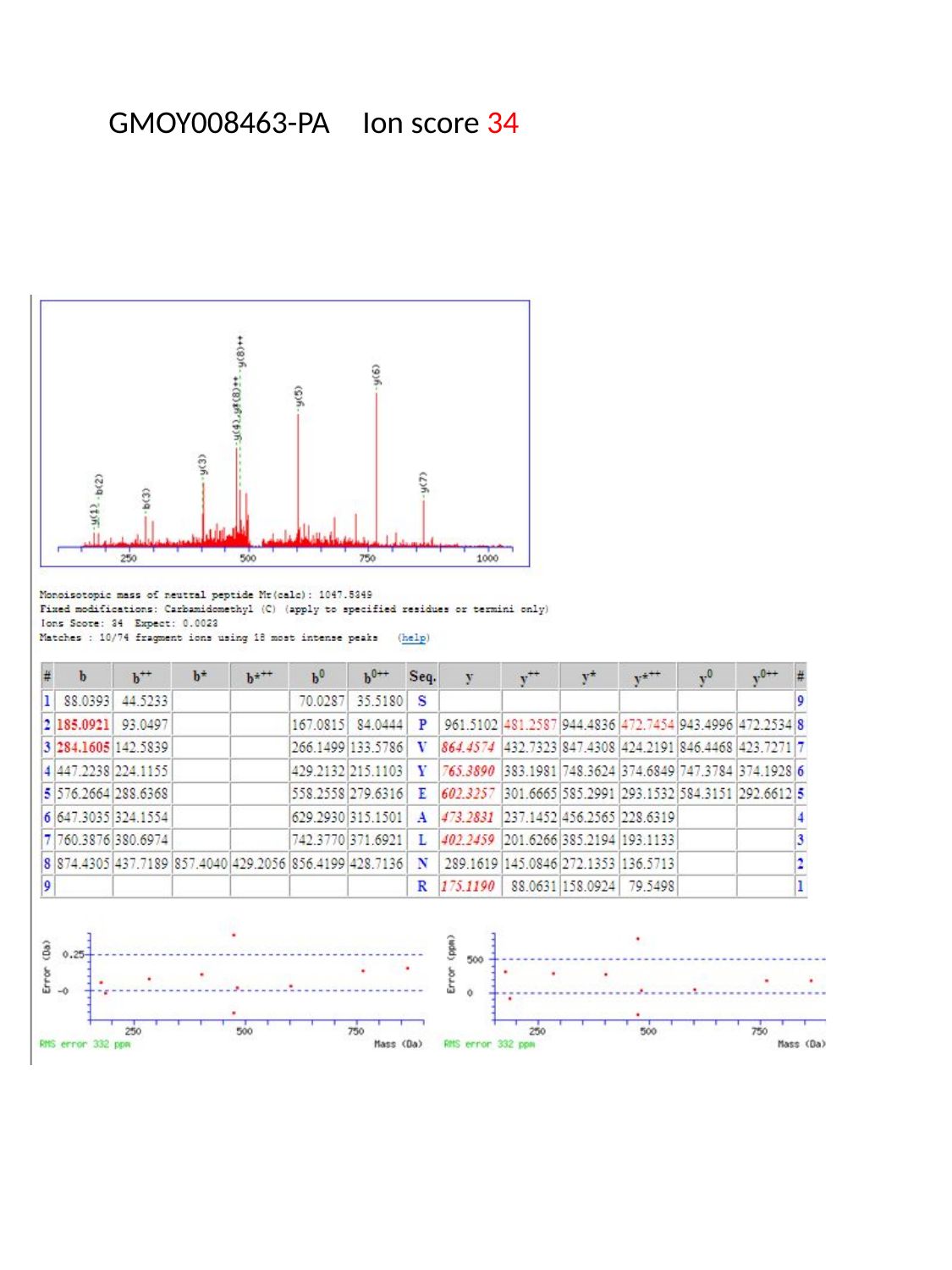

GMOY008463-PA 	Ion score 34

## Slide 24
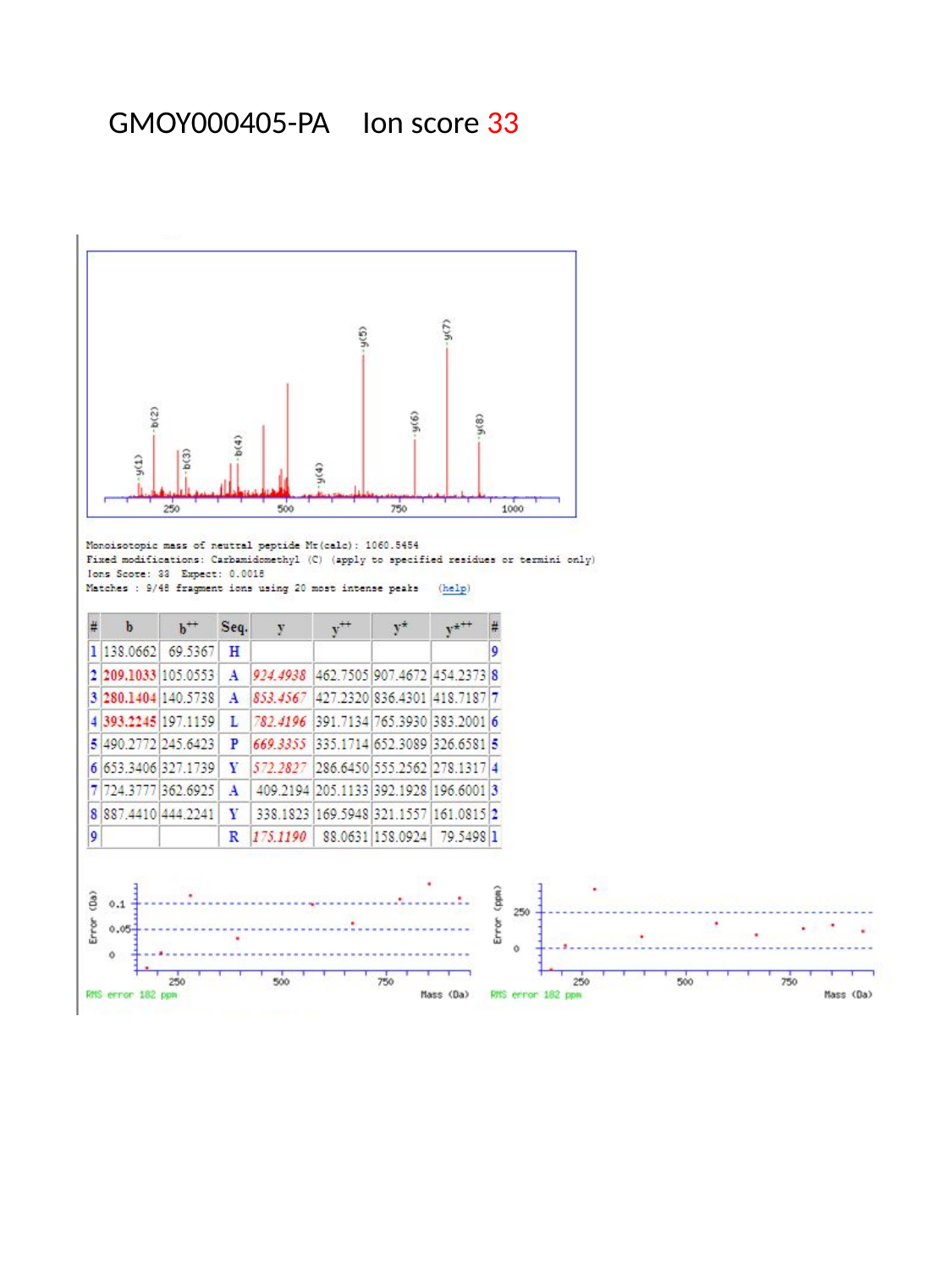

GMOY000405-PA 	Ion score 33

## Slide 25
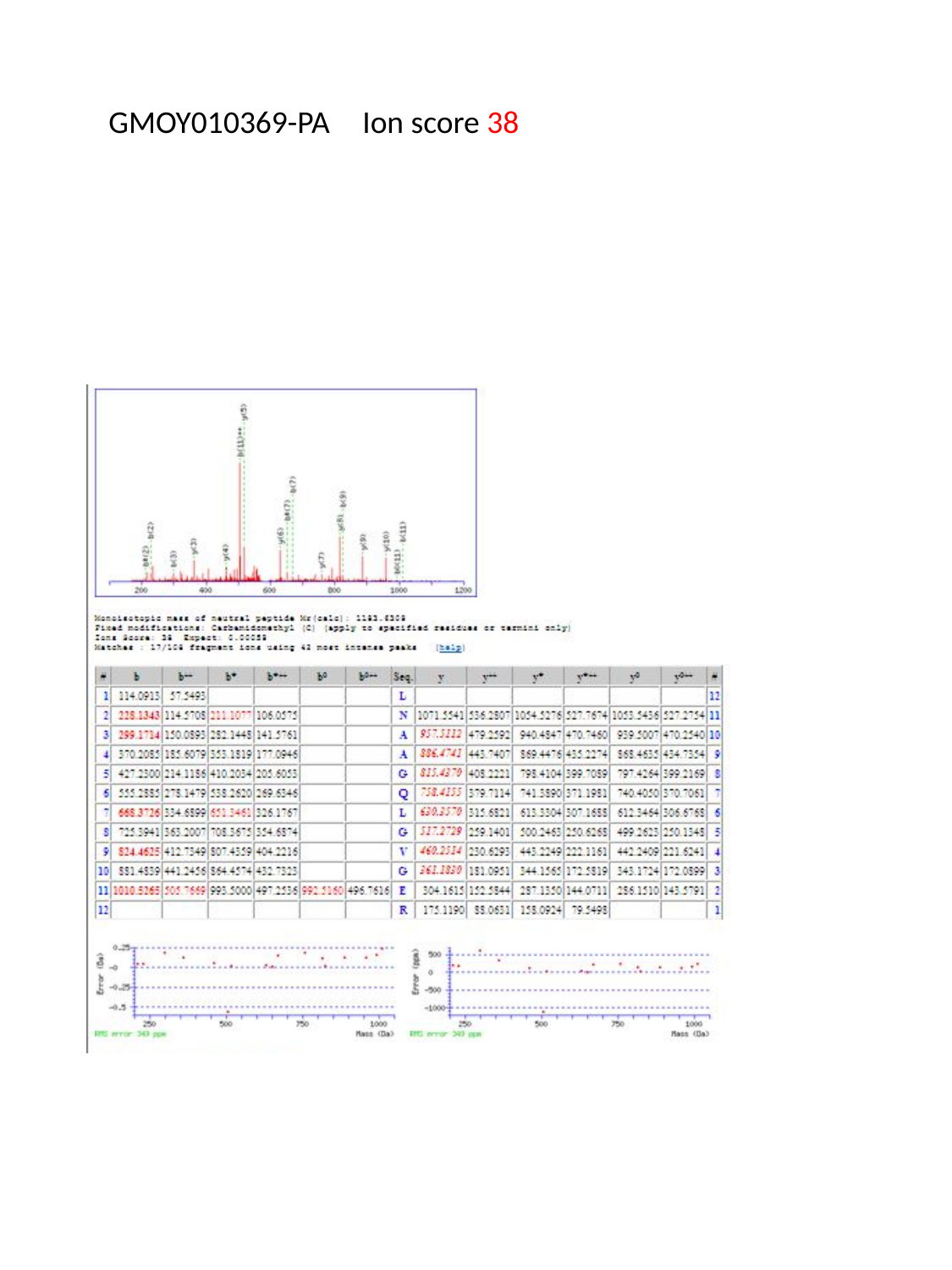

GMOY010369-PA 	Ion score 38

## Slide 26
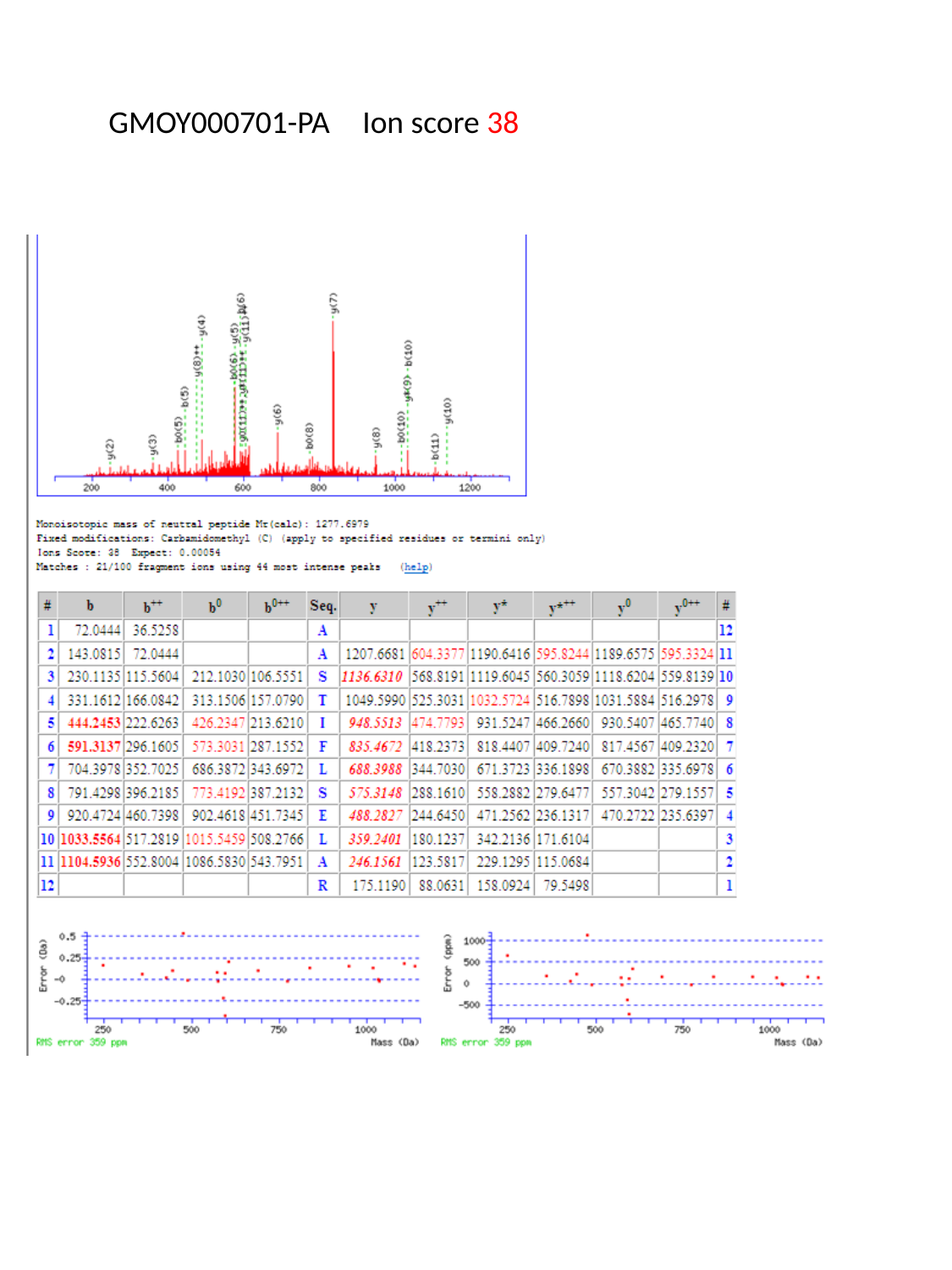

GMOY000701-PA 	Ion score 38

## Slide 27
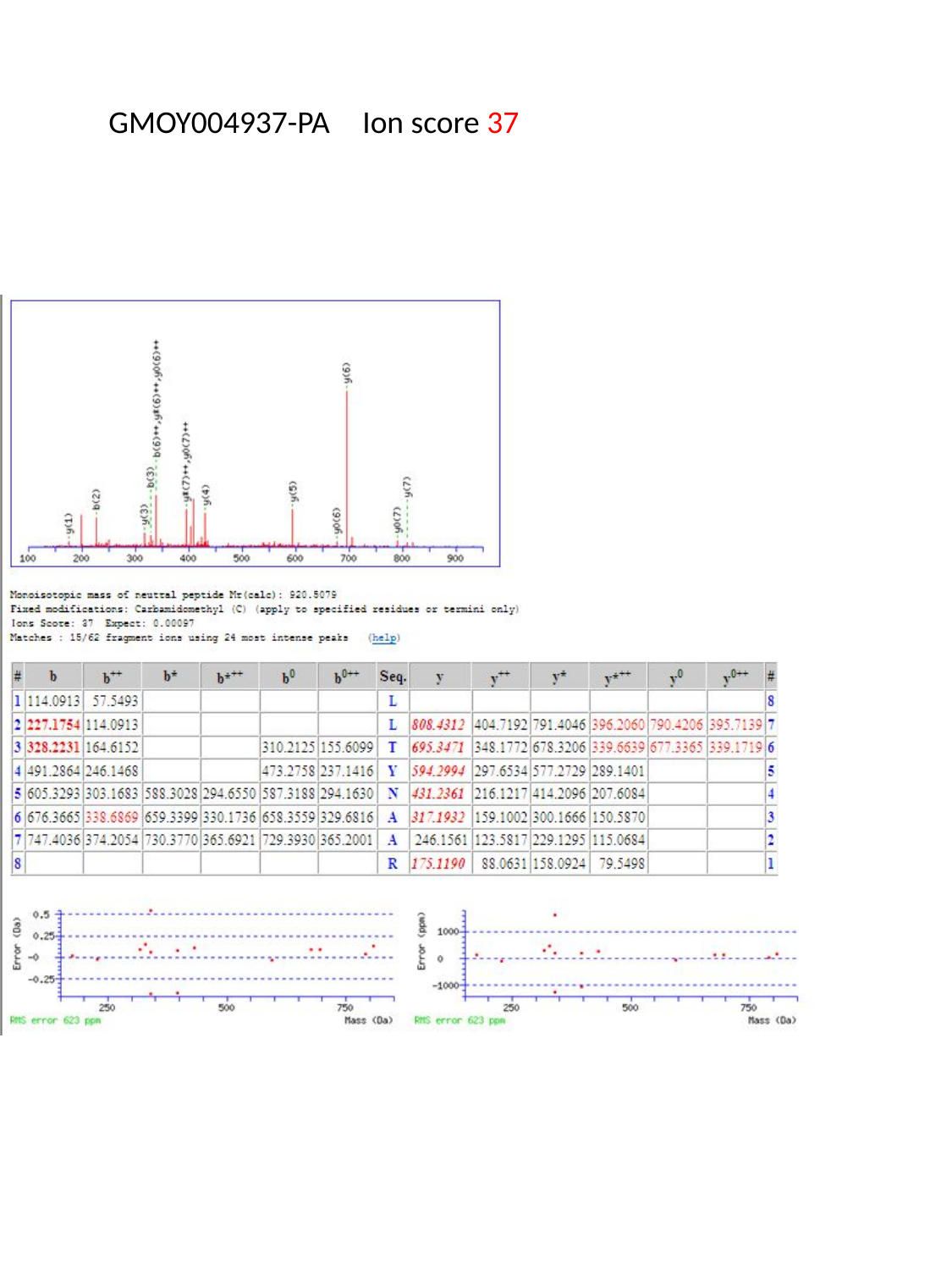

GMOY004937-PA 	Ion score 37

## Slide 28
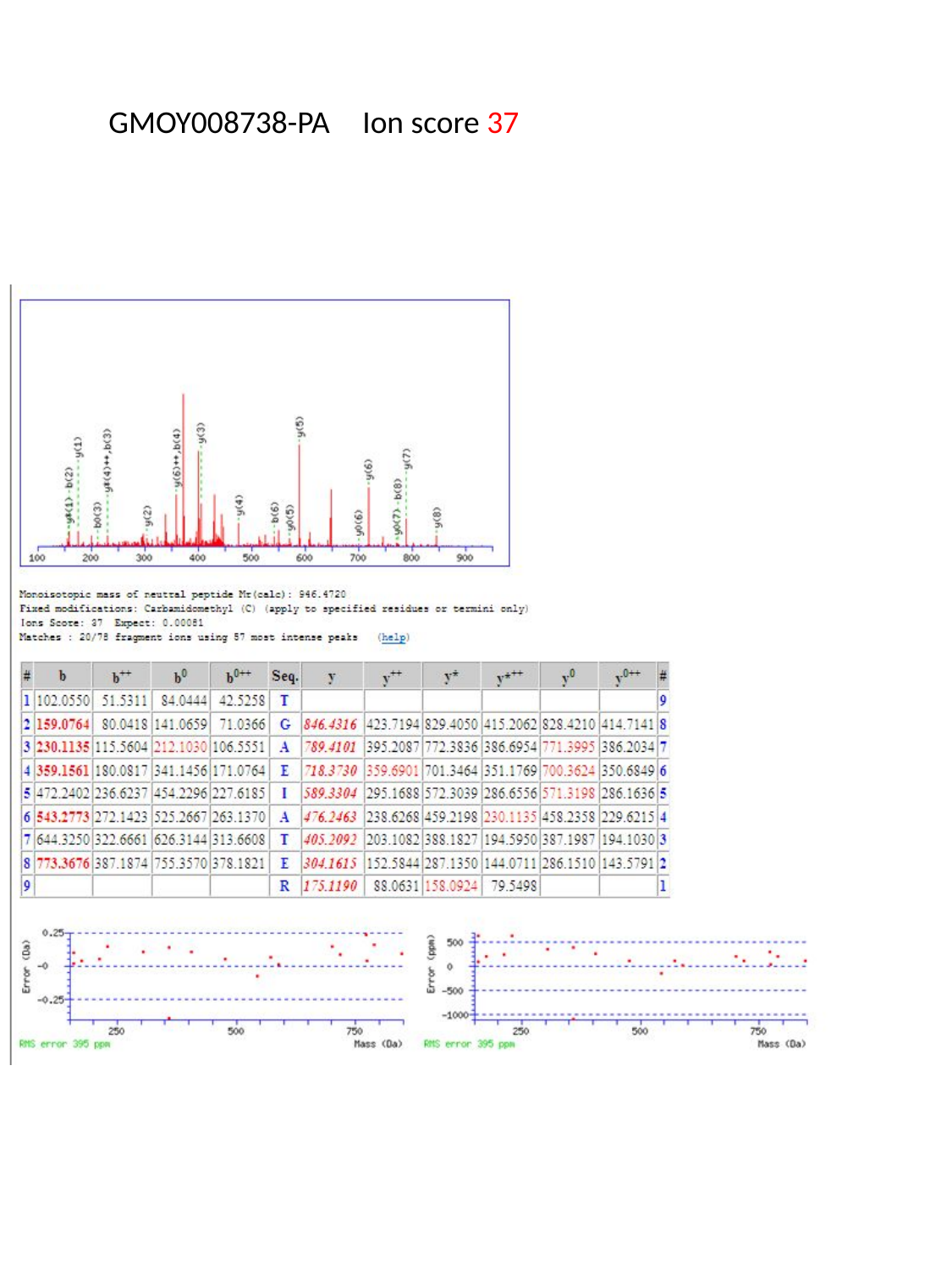

GMOY008738-PA 	Ion score 37

## Slide 29
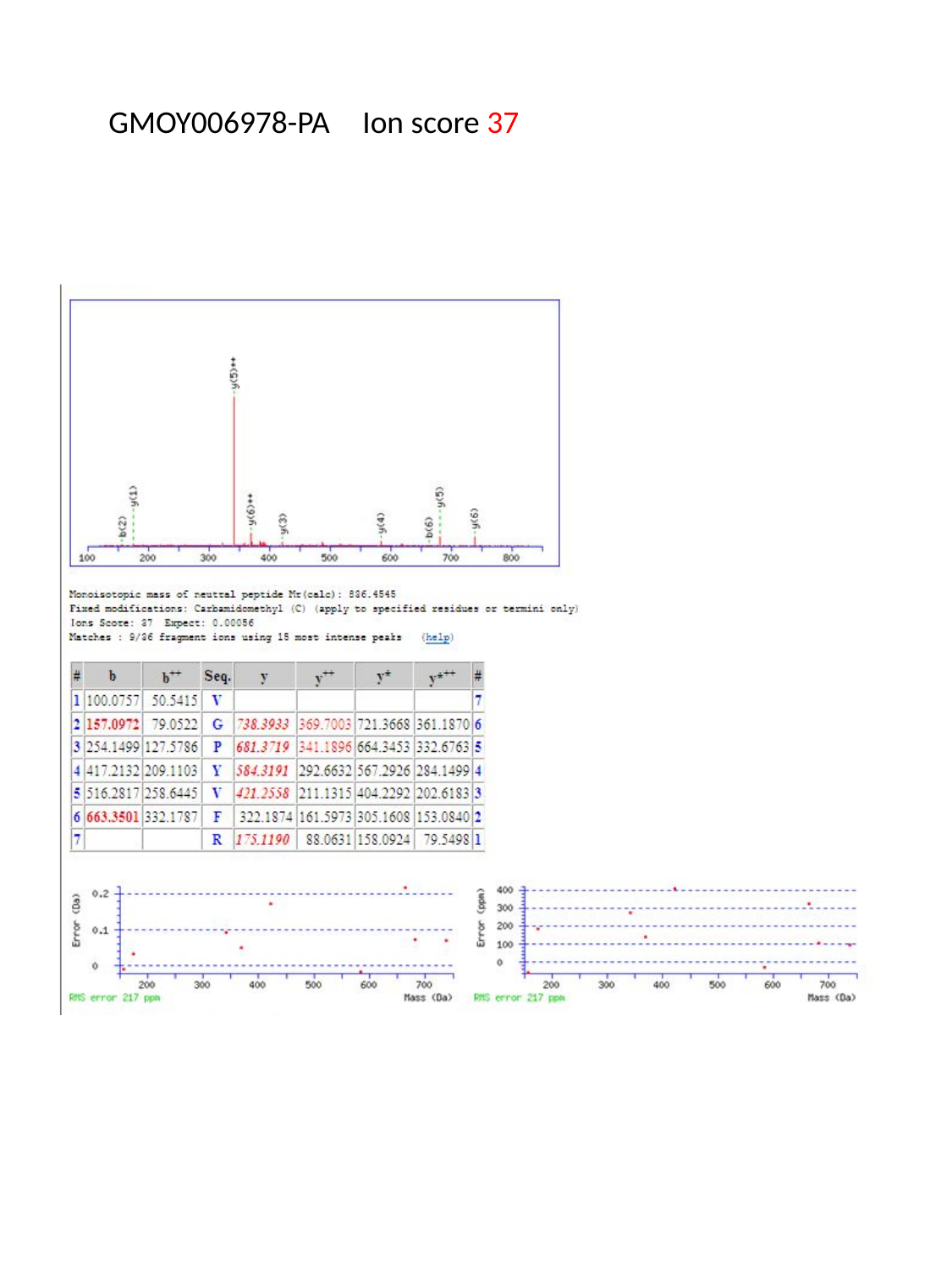

GMOY006978-PA 	Ion score 37

## Slide 30
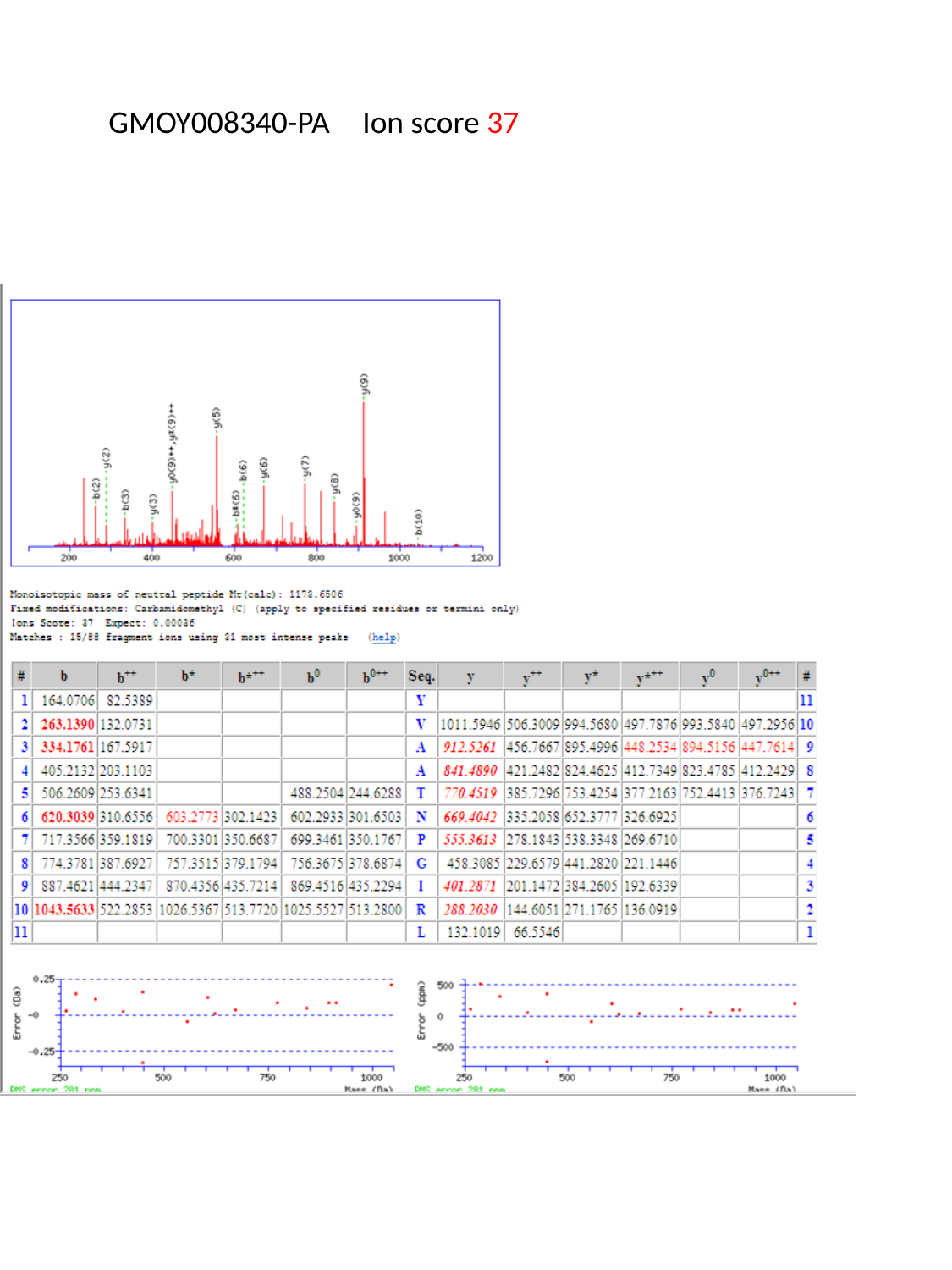

GMOY008340-PA 	Ion score 37

## Slide 31
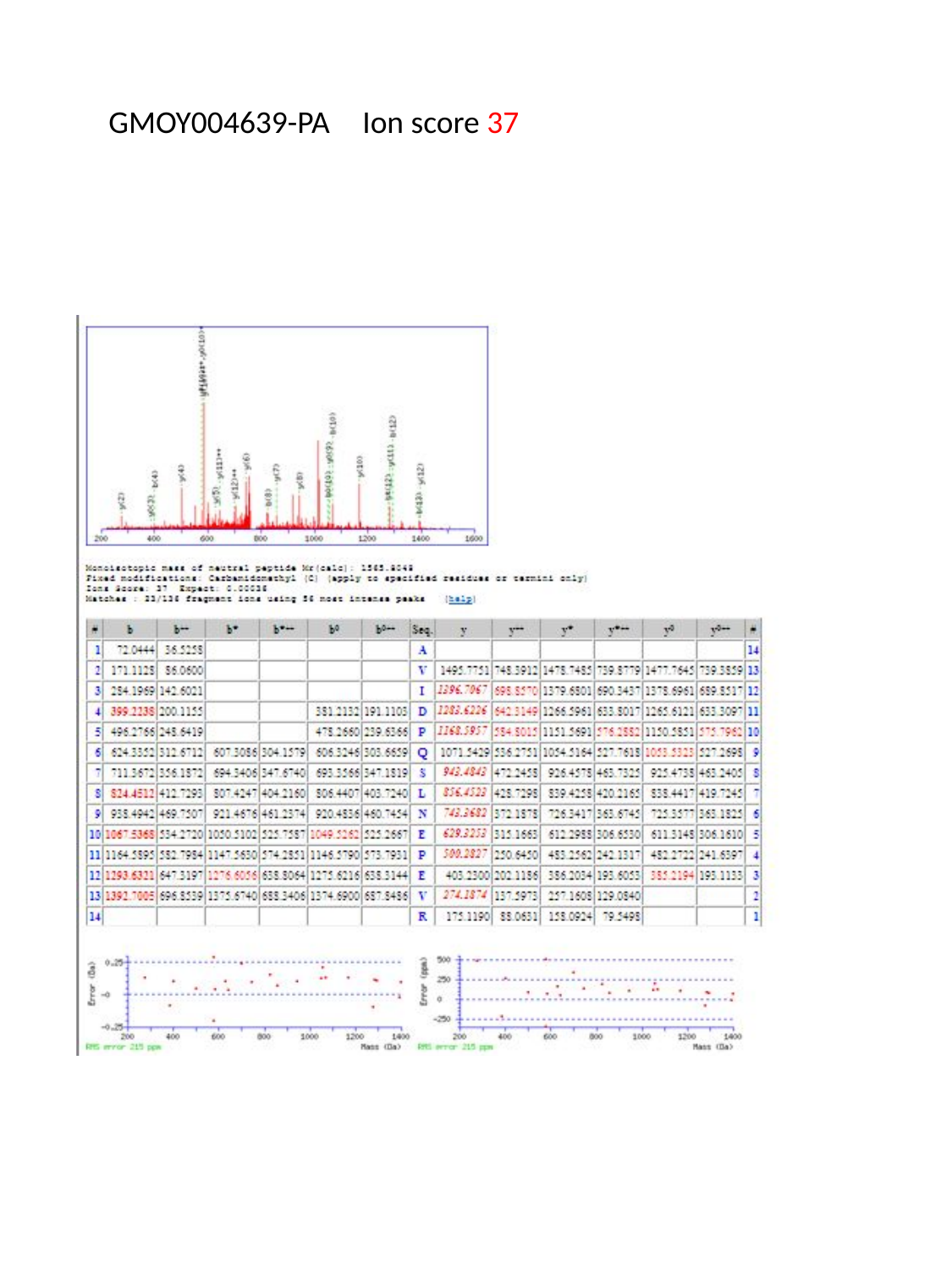

GMOY004639-PA 	Ion score 37

## Slide 32
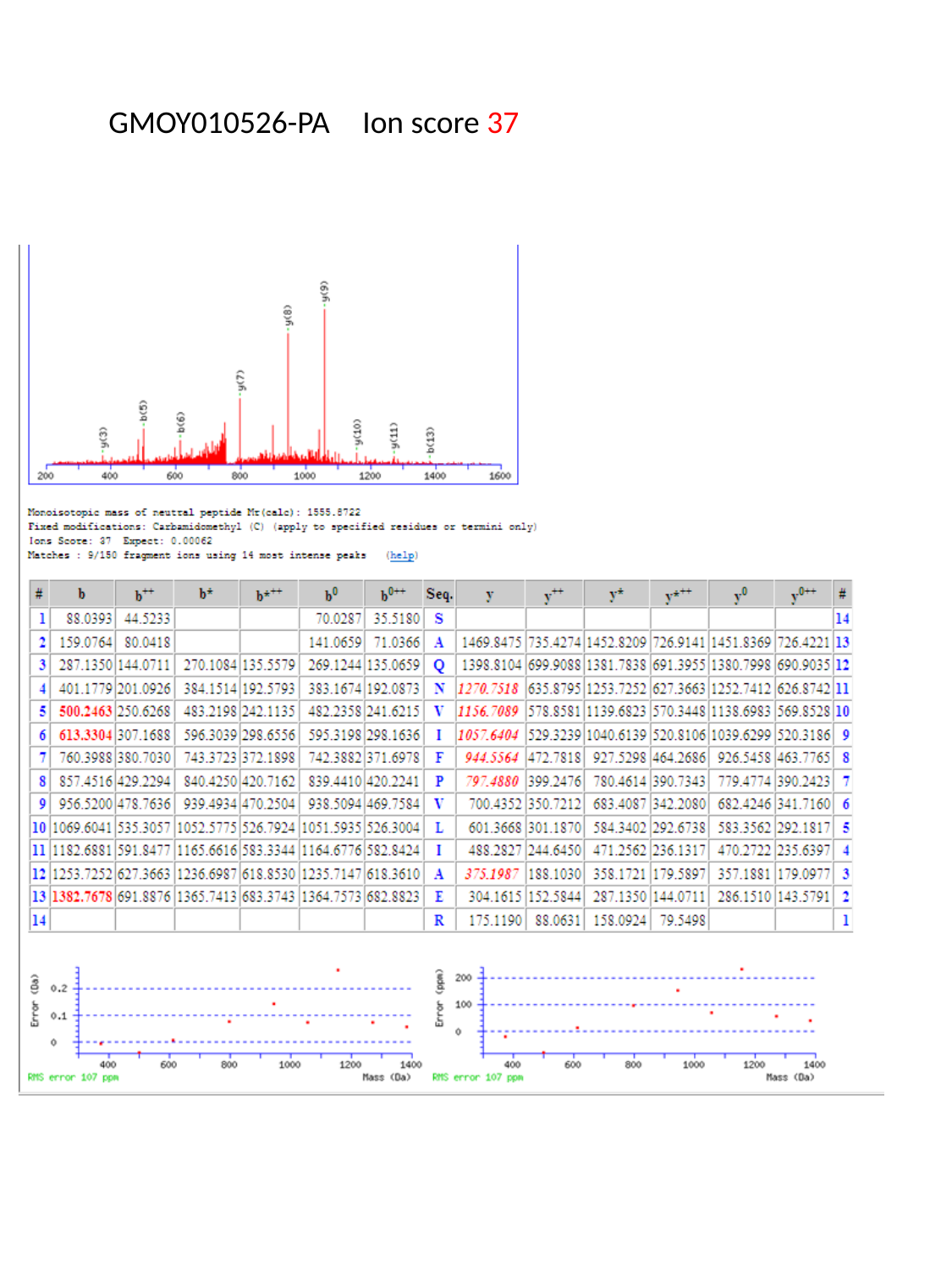

GMOY010526-PA 	Ion score 37

## Slide 33
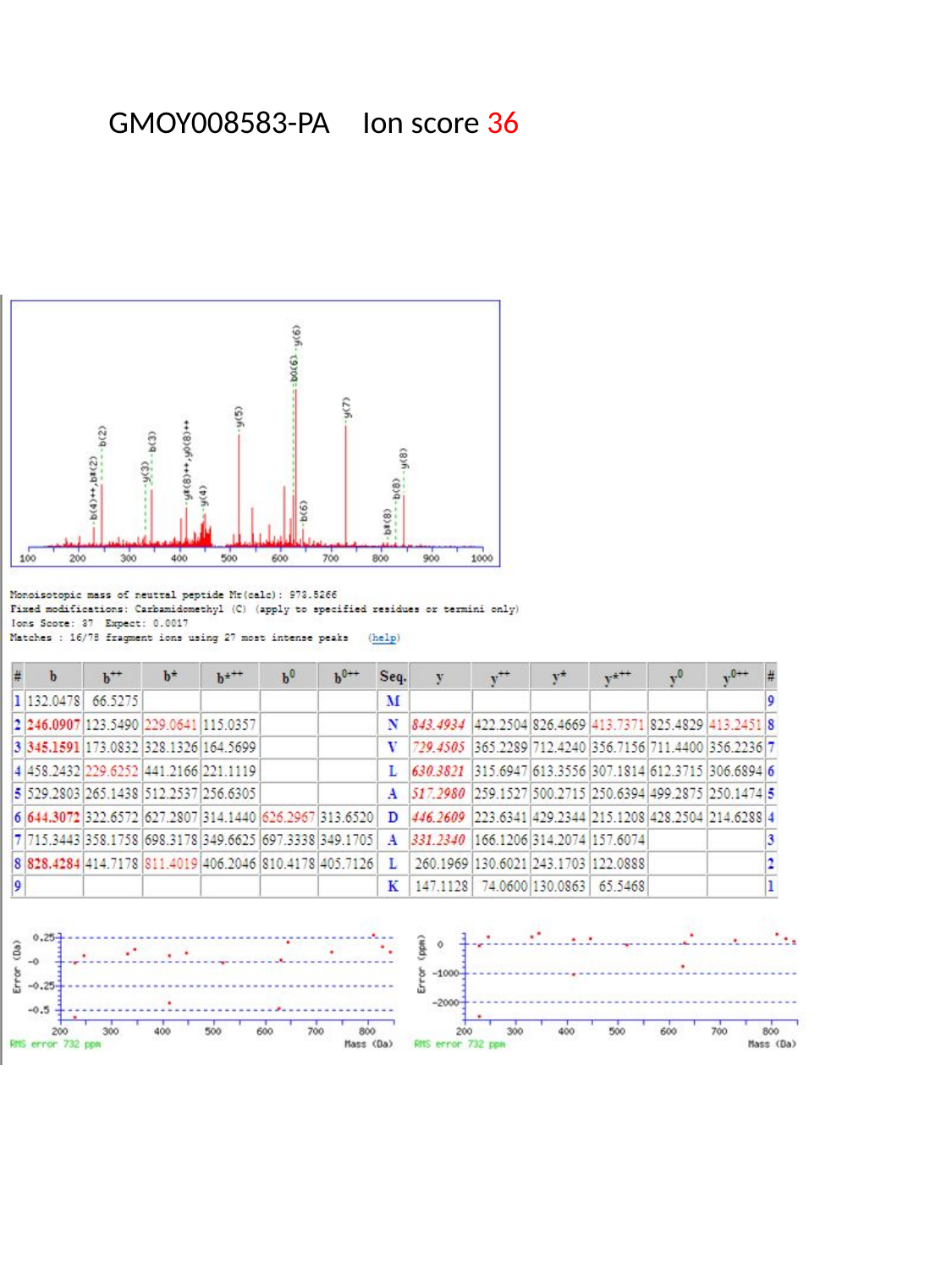

GMOY008583-PA 	Ion score 36

## Slide 34
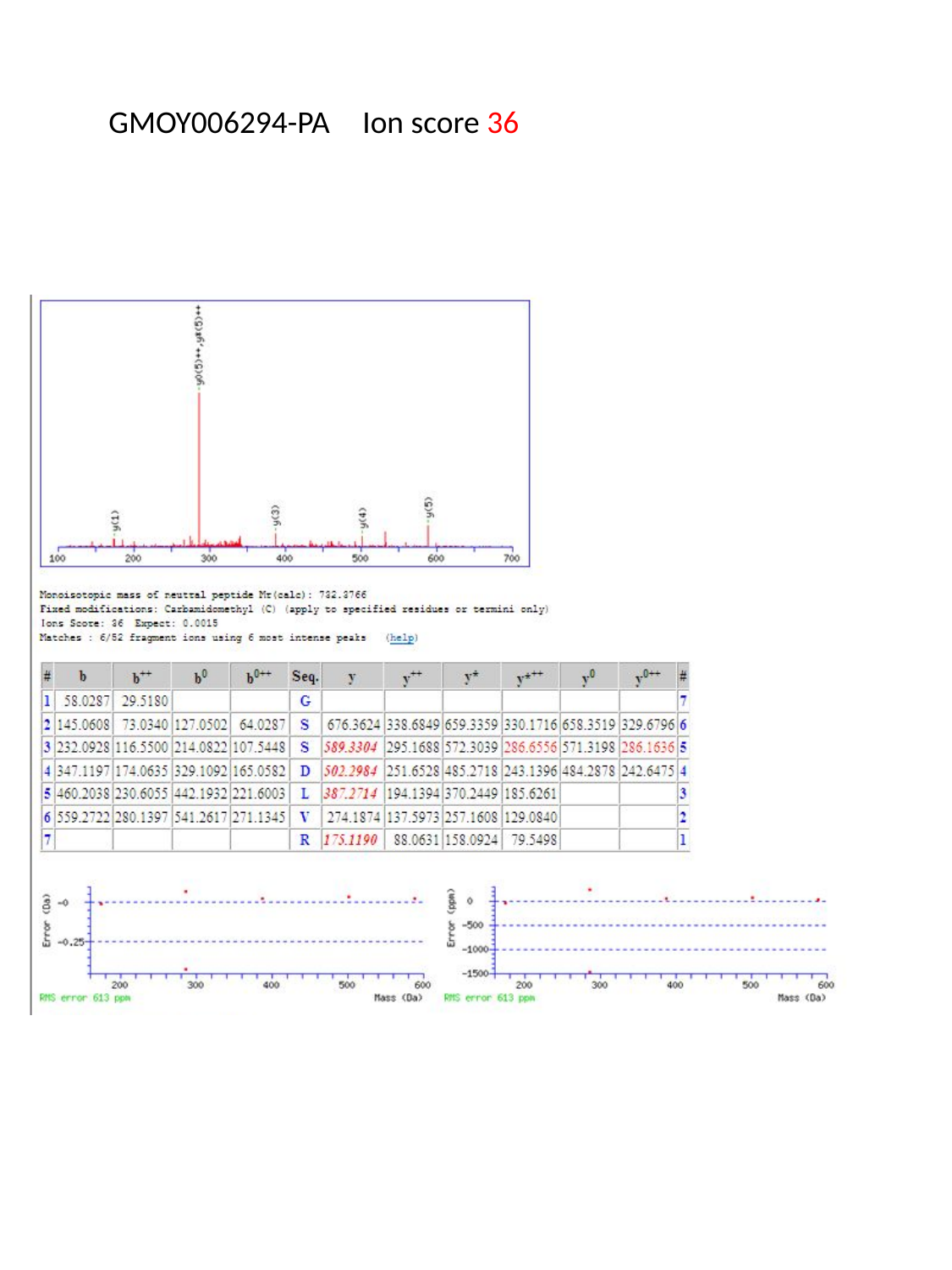

GMOY006294-PA	Ion score 36

## Slide 35
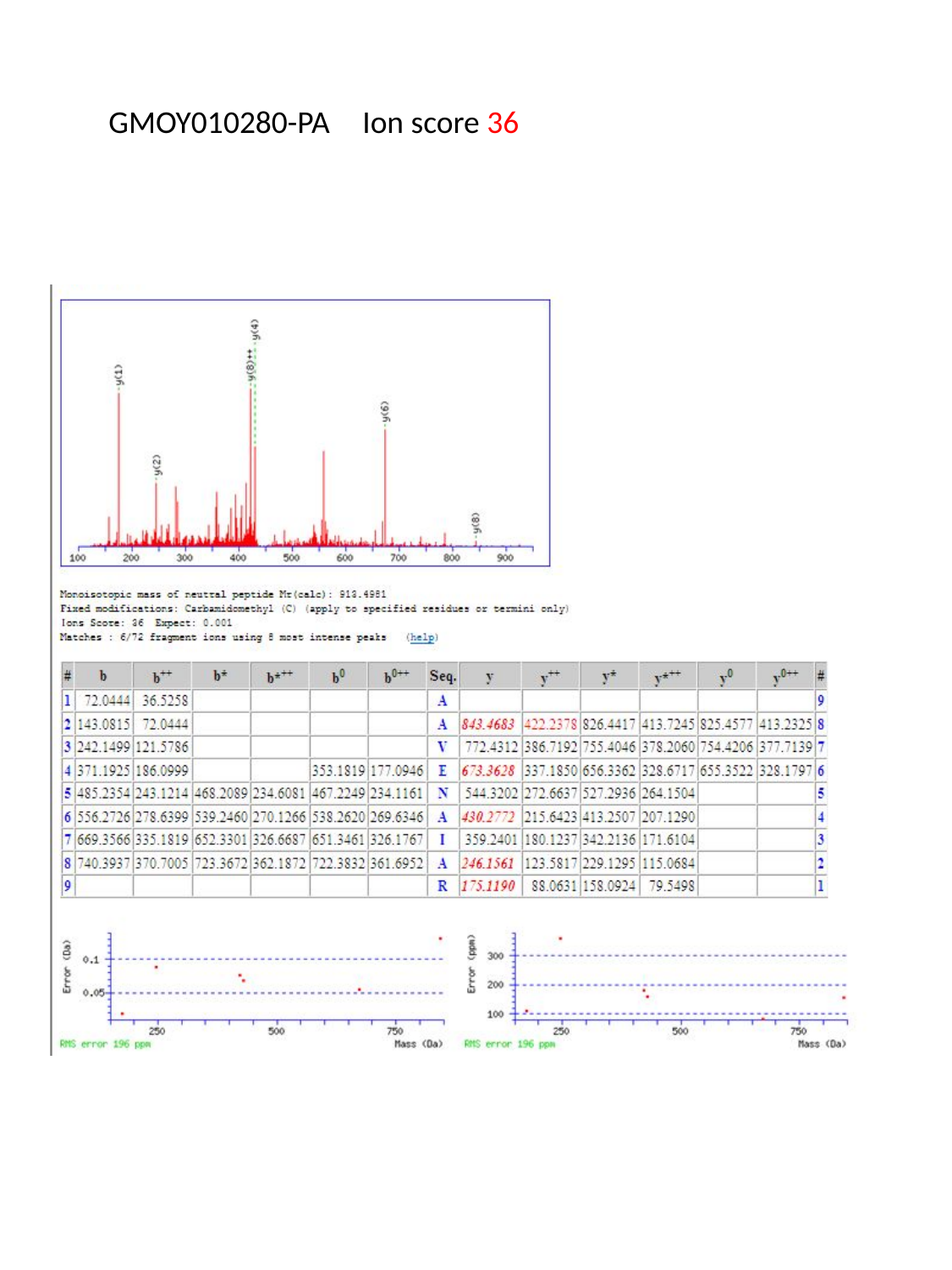

GMOY010280-PA	Ion score 36

## Slide 36
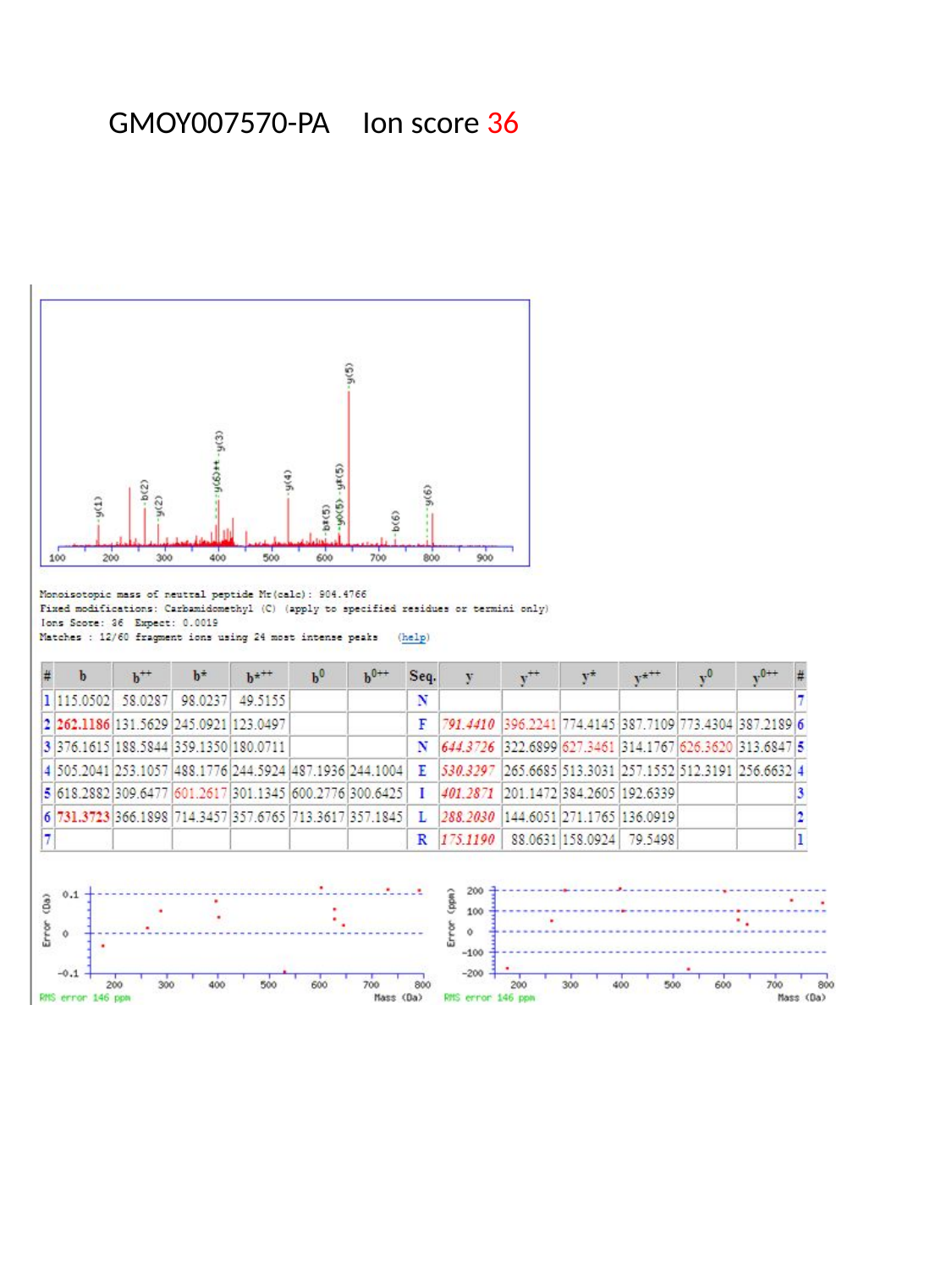

GMOY007570-PA	Ion score 36

## Slide 37
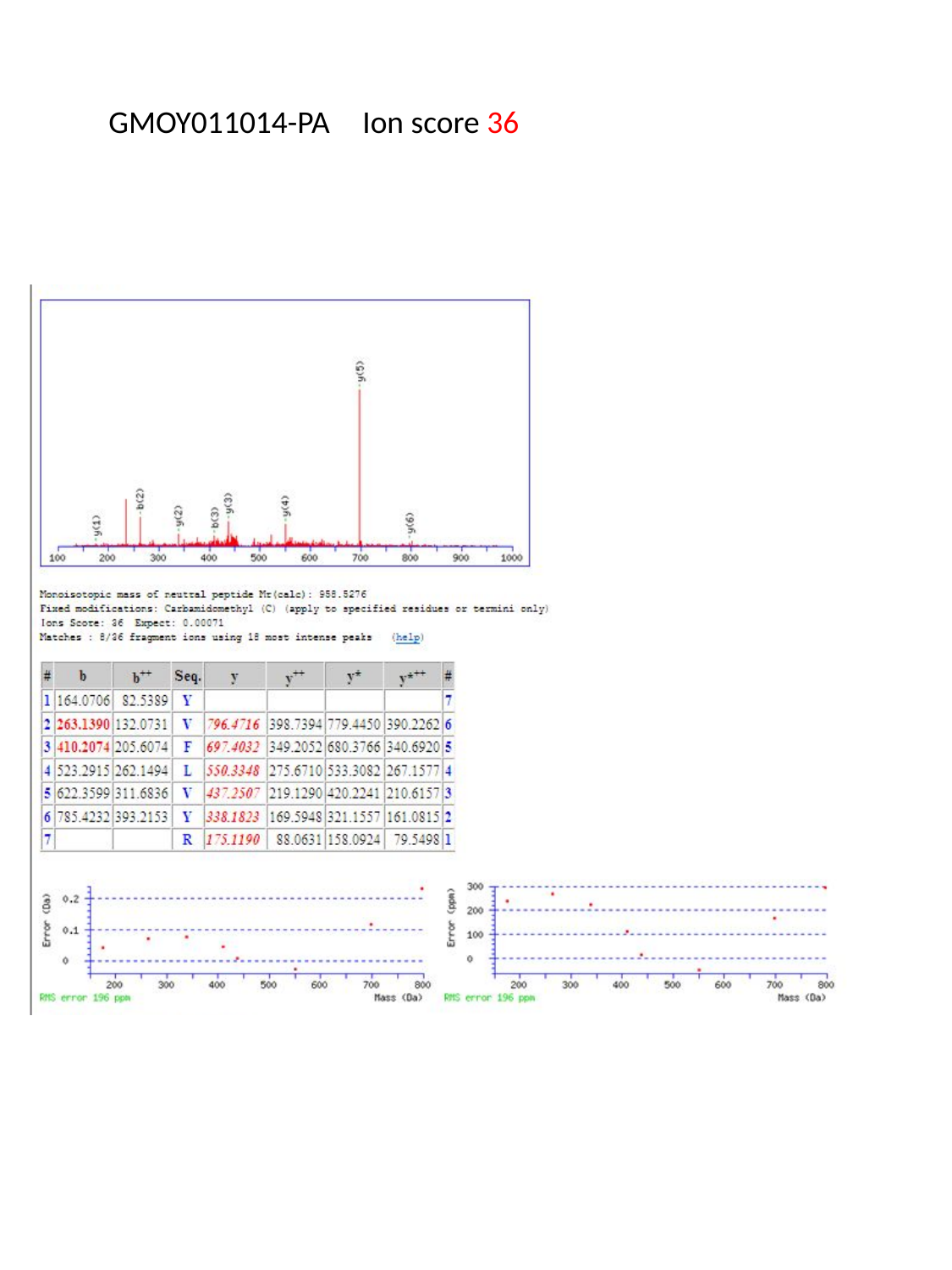

GMOY011014-PA	Ion score 36

## Slide 38
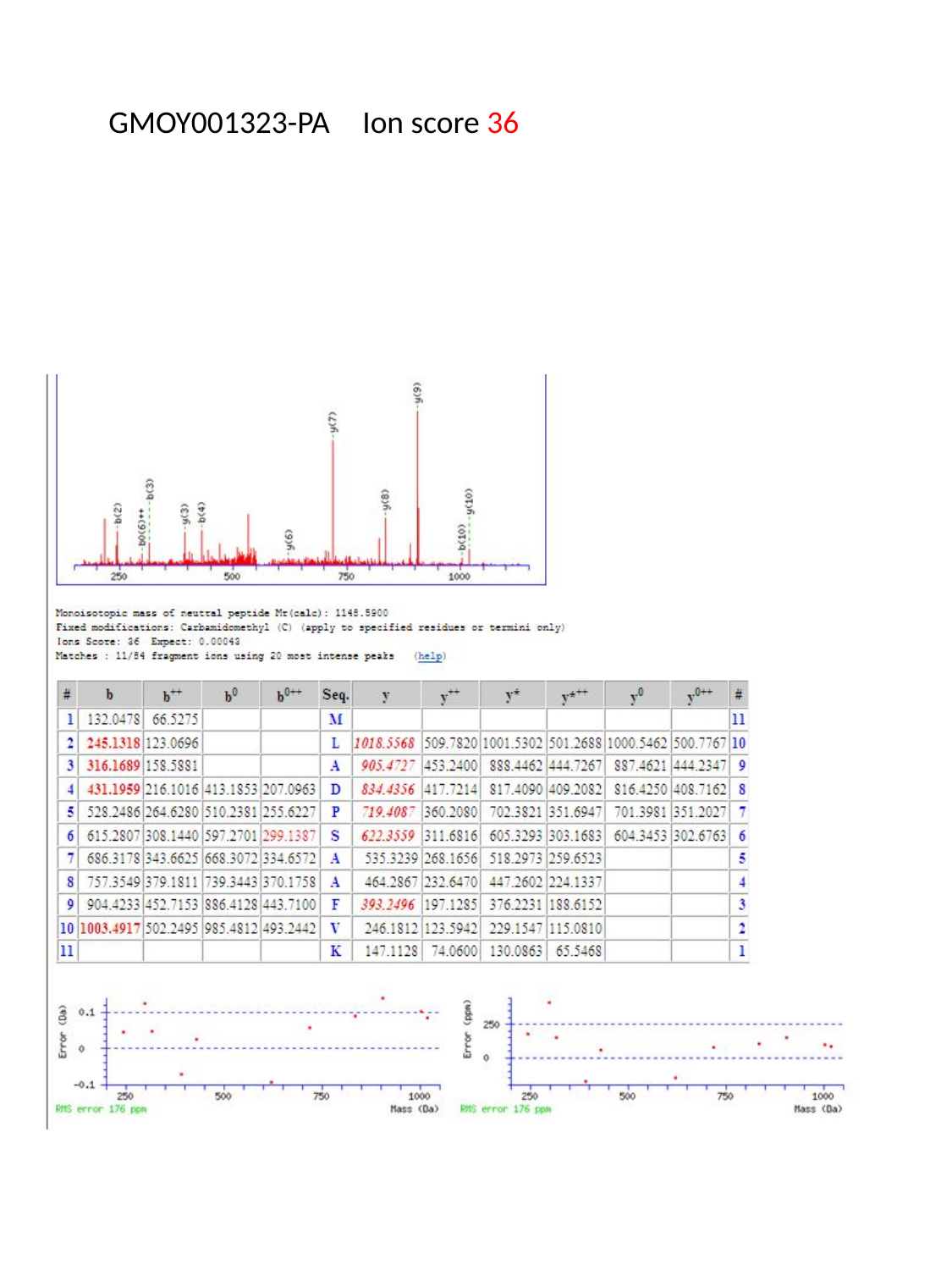

GMOY001323-PA	Ion score 36

## Slide 39
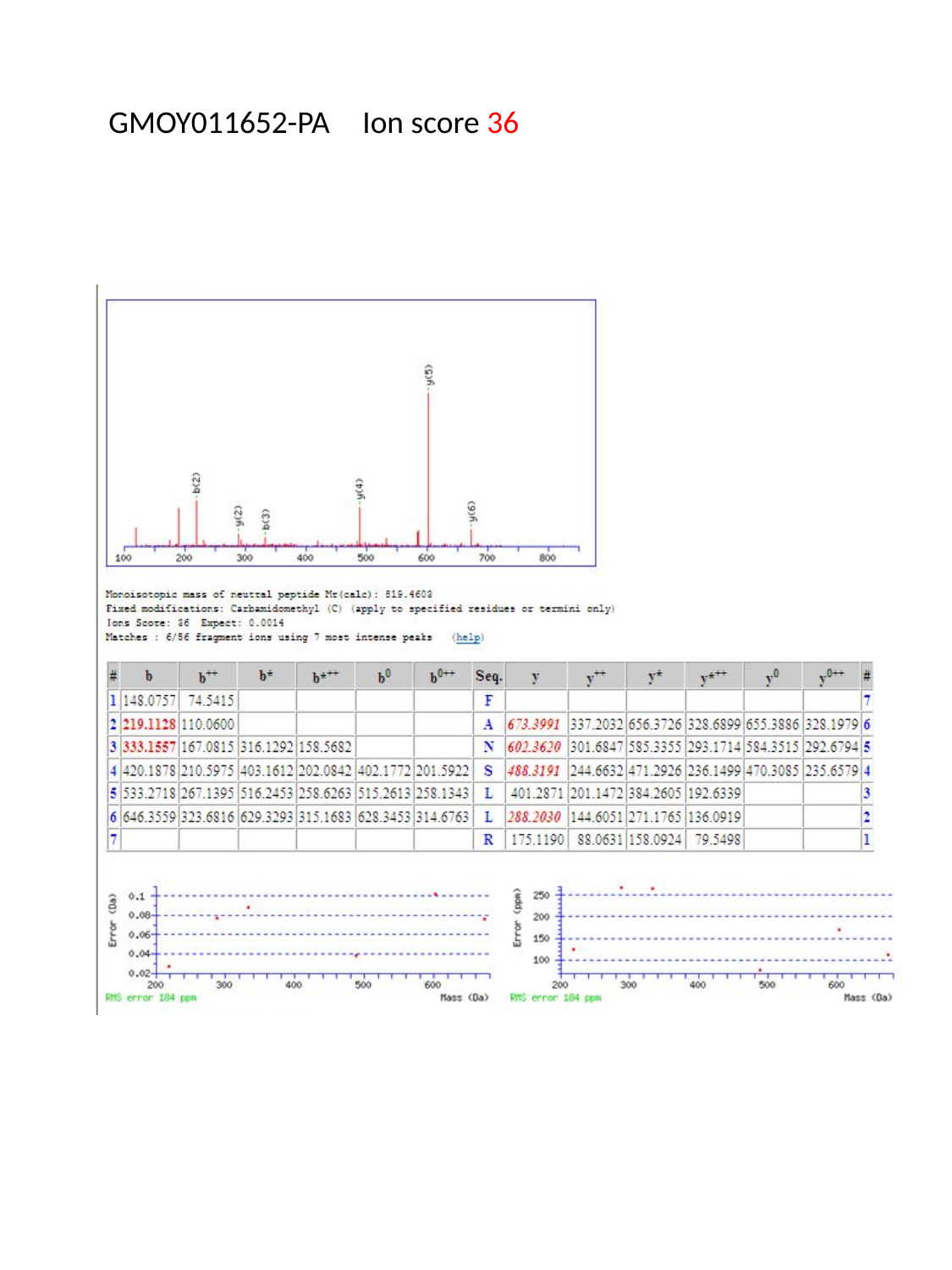

GMOY011652-PA	Ion score 36

## Slide 40
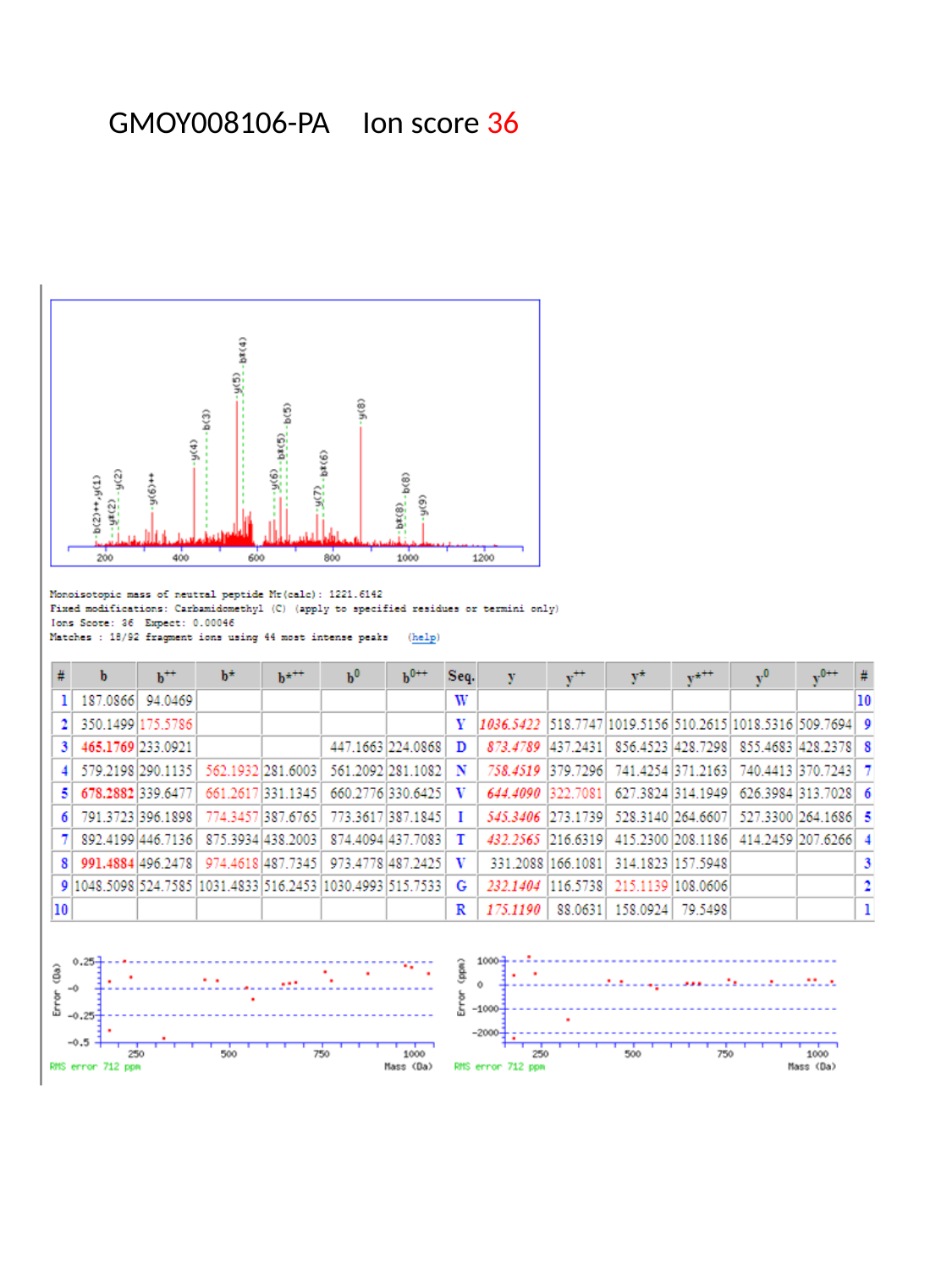

GMOY008106-PA	Ion score 36

## Slide 41
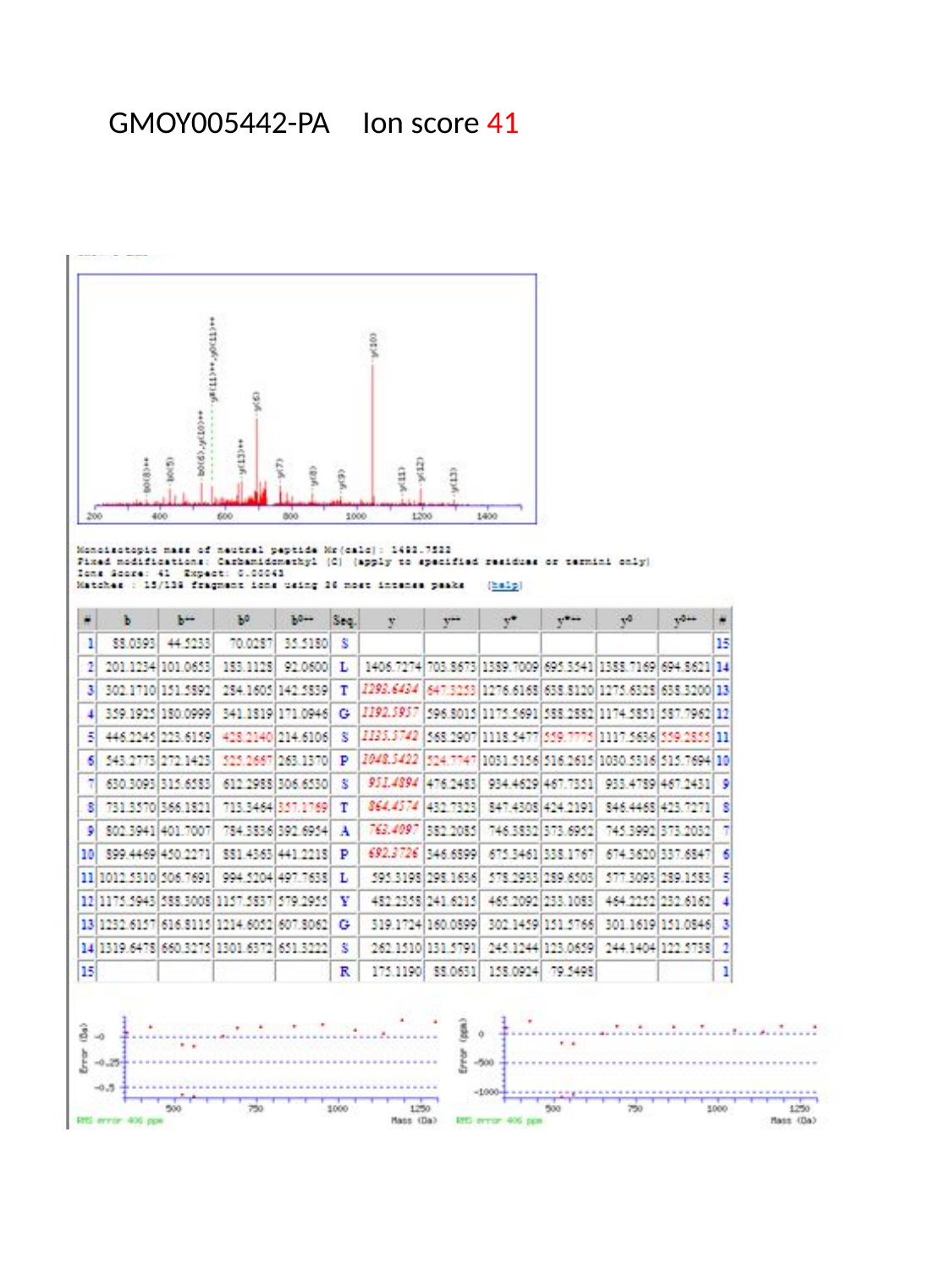

GMOY005442-PA	Ion score 41

## Slide 42
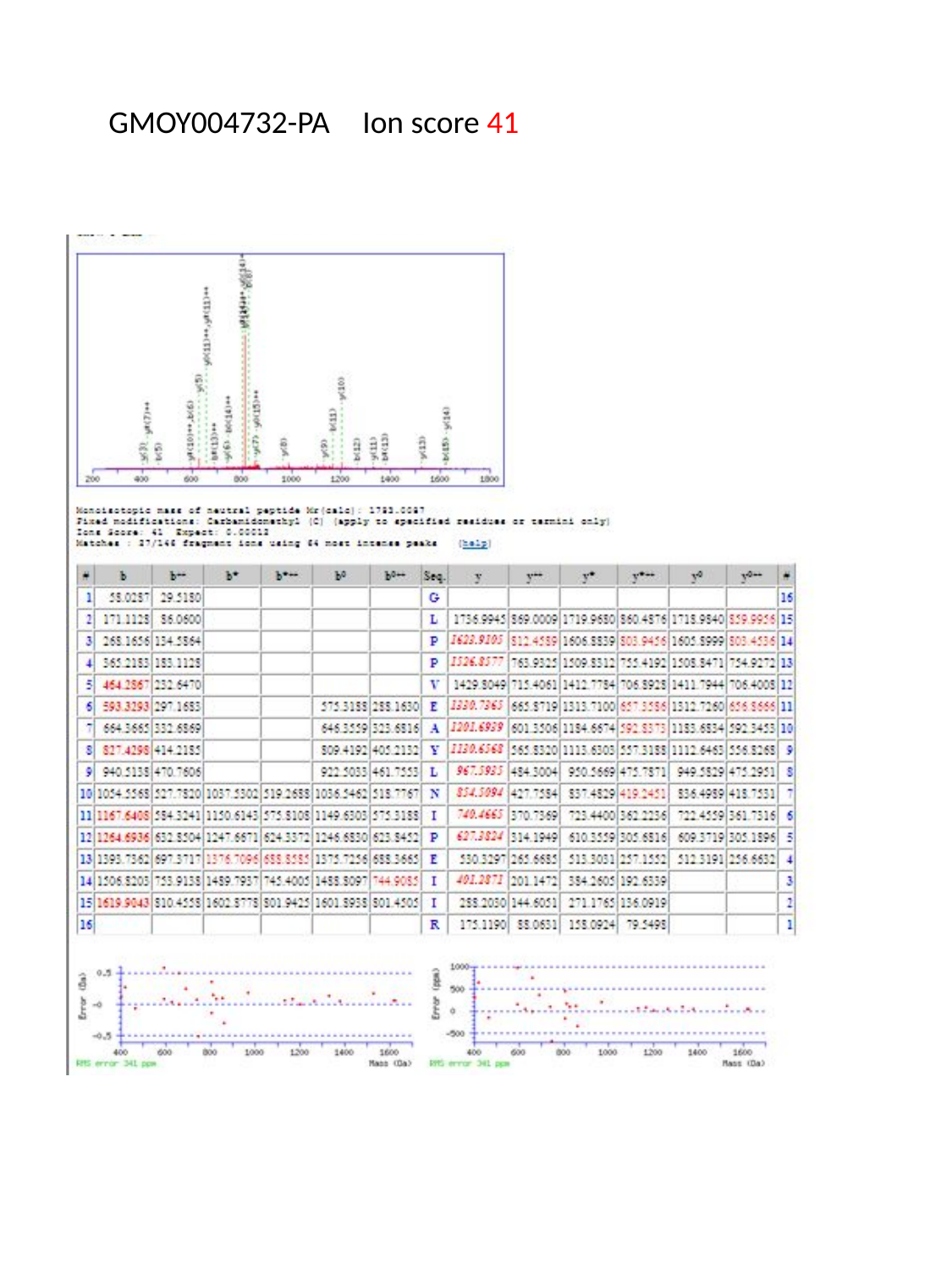

GMOY004732-PA	Ion score 41

## Slide 43
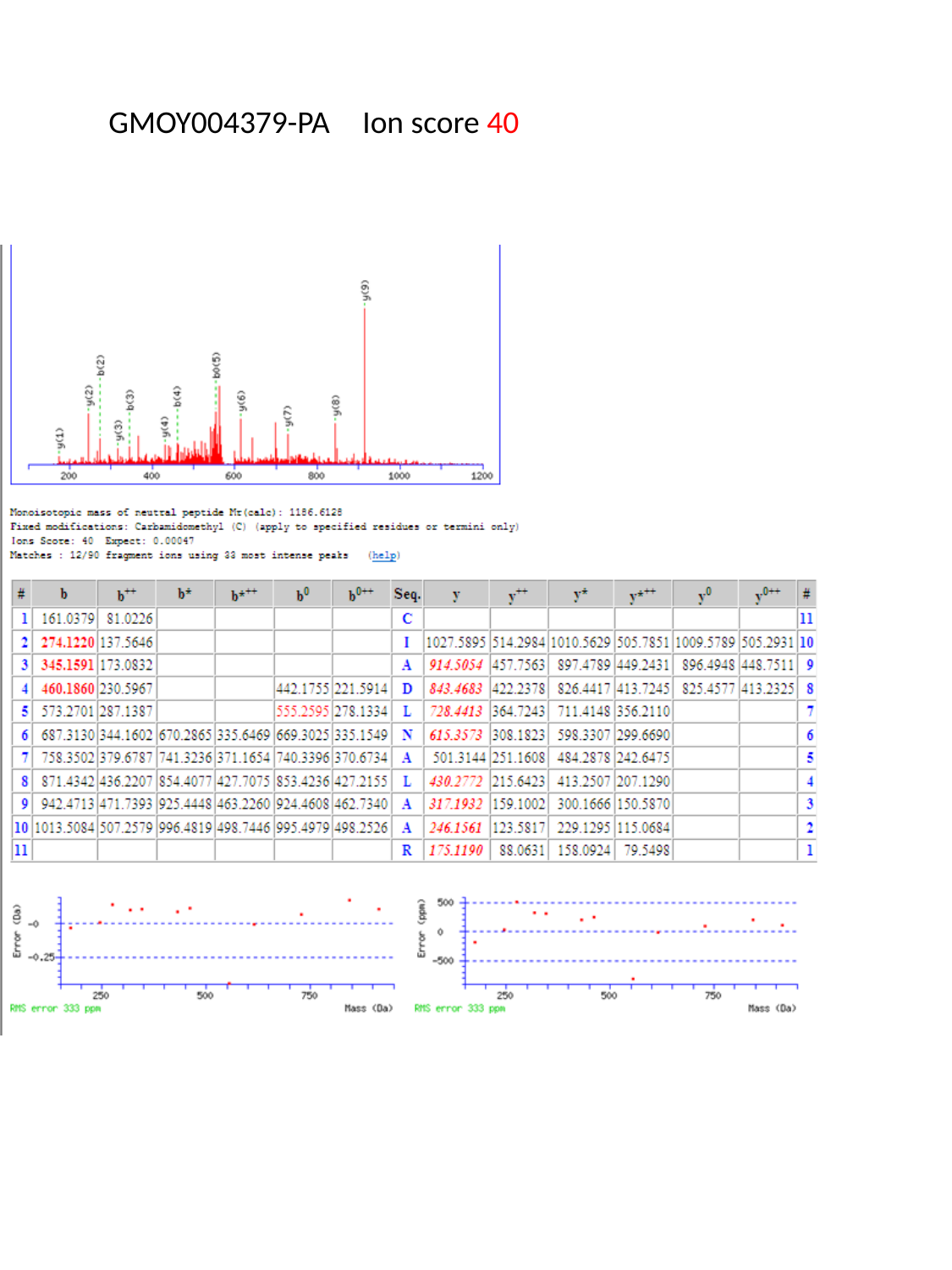

GMOY004379-PA	Ion score 40

## Slide 44
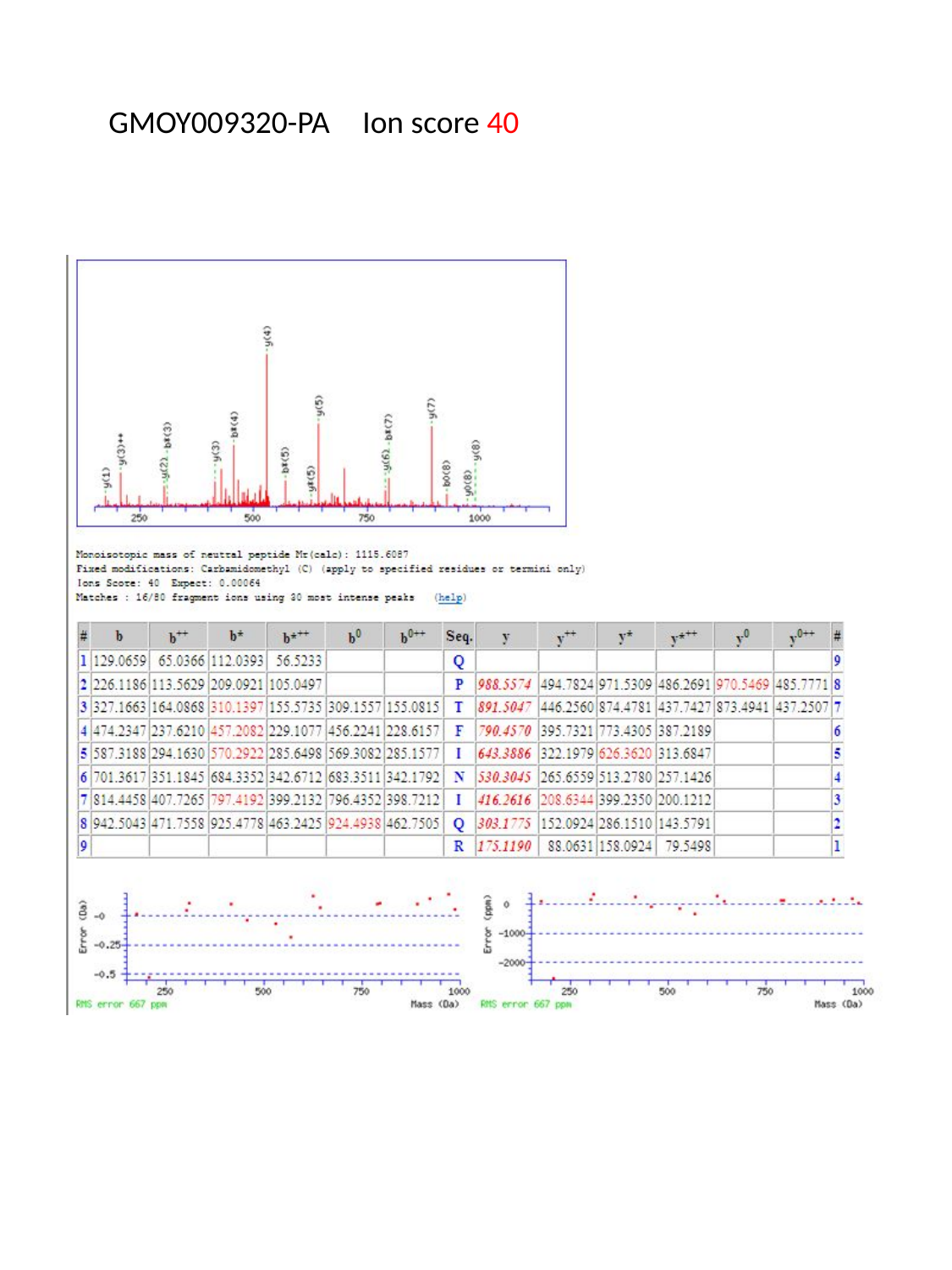

GMOY009320-PA	Ion score 40

## Slide 45
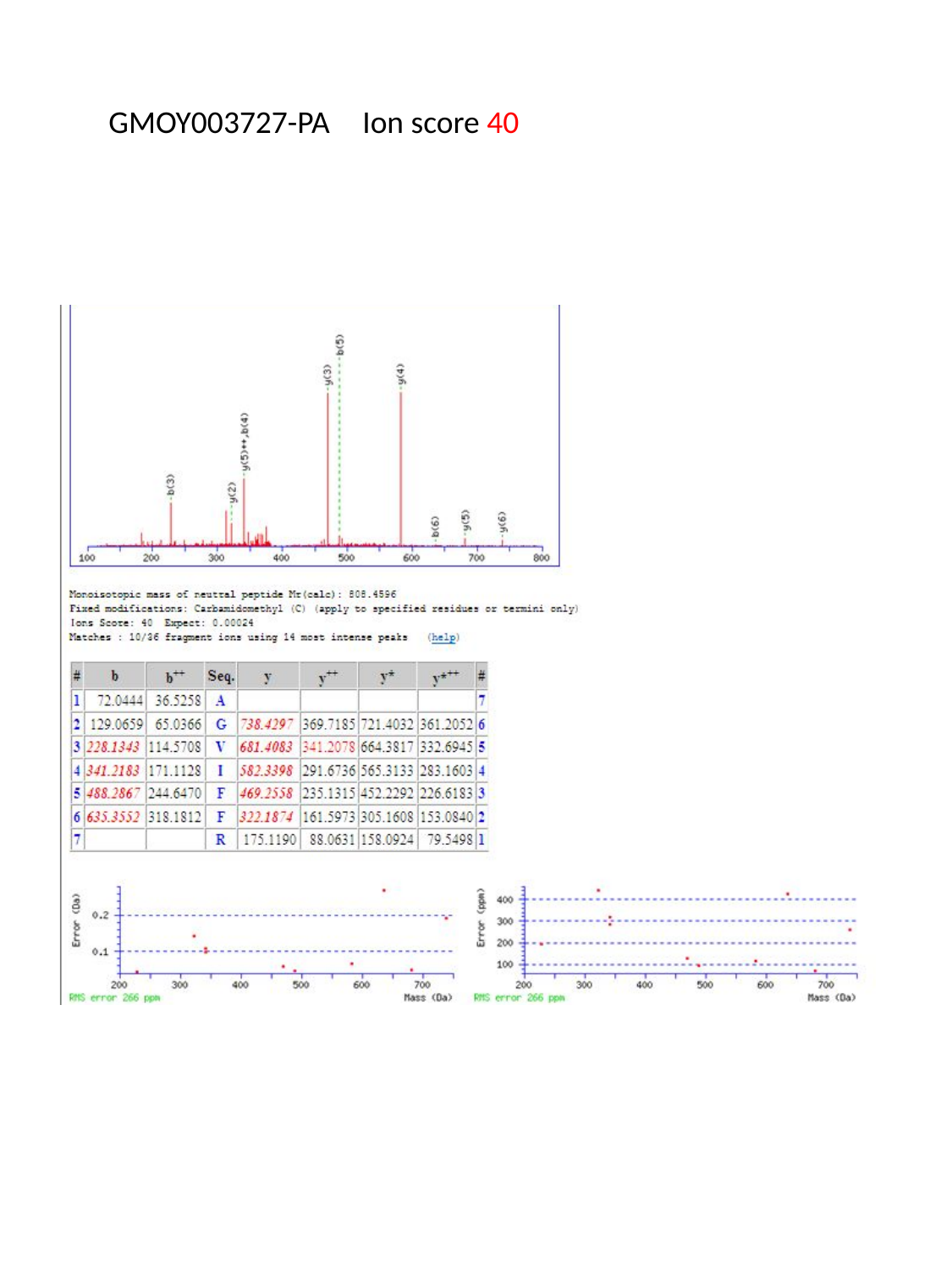

GMOY003727-PA	Ion score 40

## Slide 46
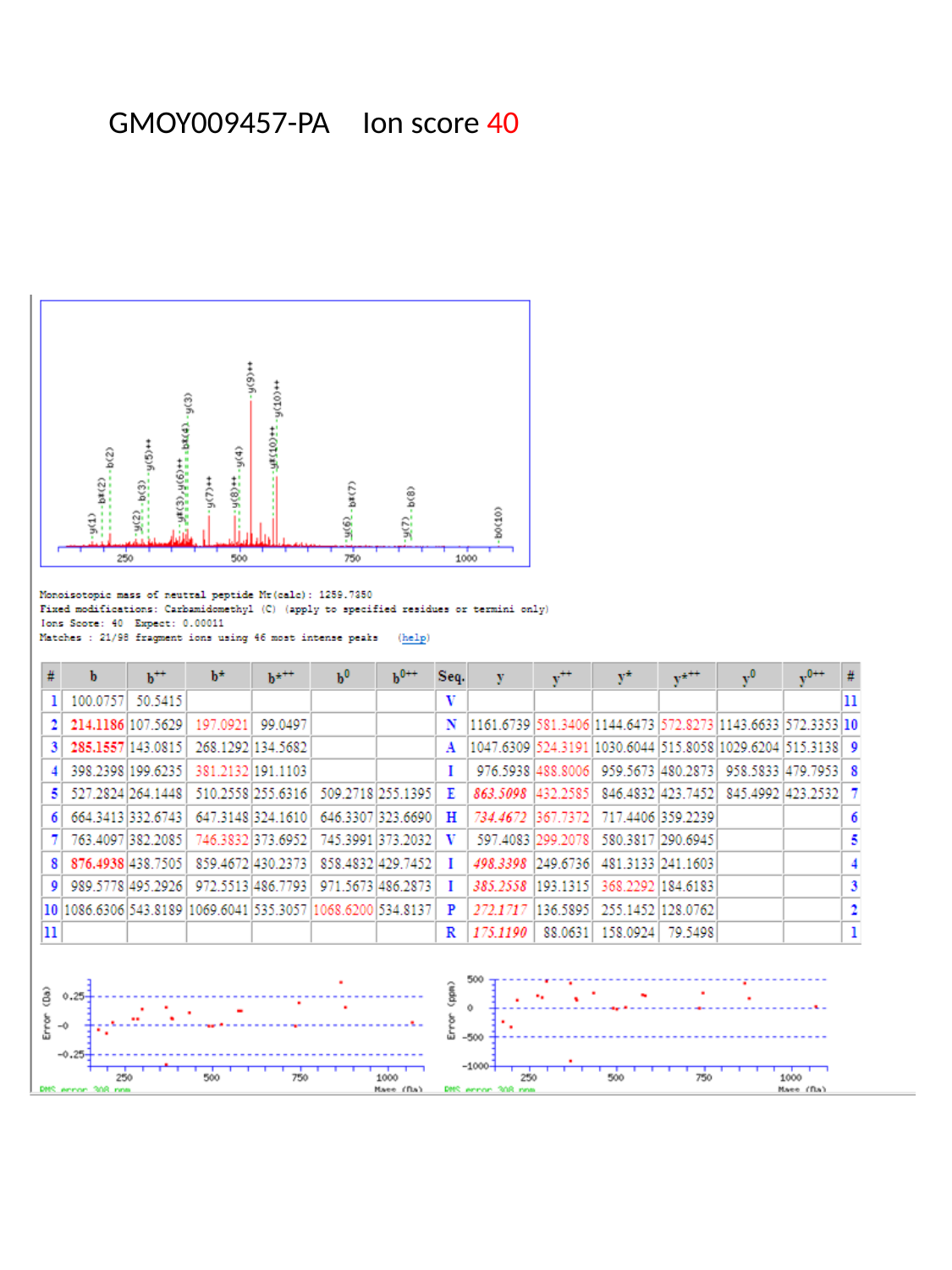

GMOY009457-PA	Ion score 40

## Slide 47
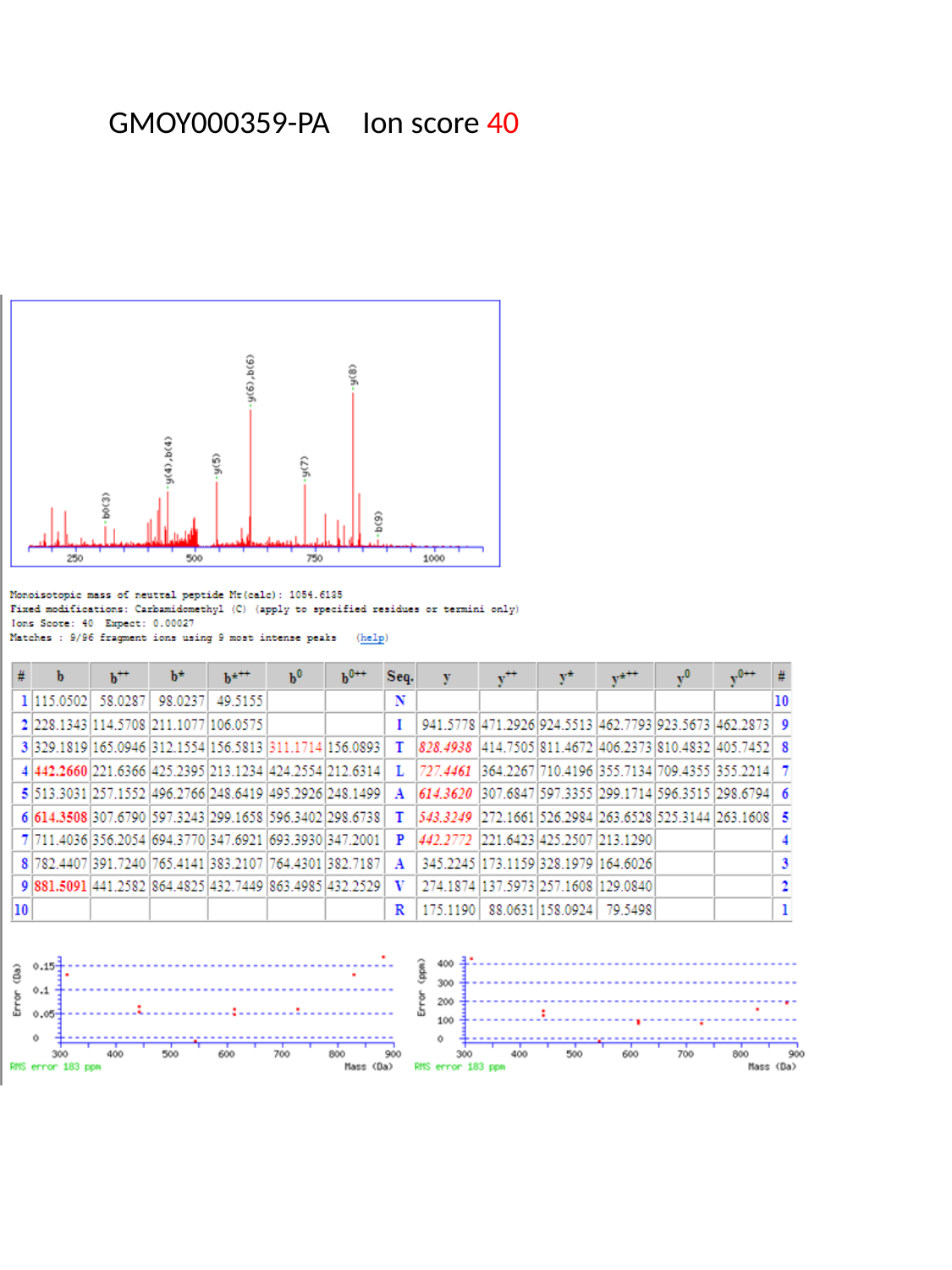

GMOY000359-PA	Ion score 40

## Slide 48
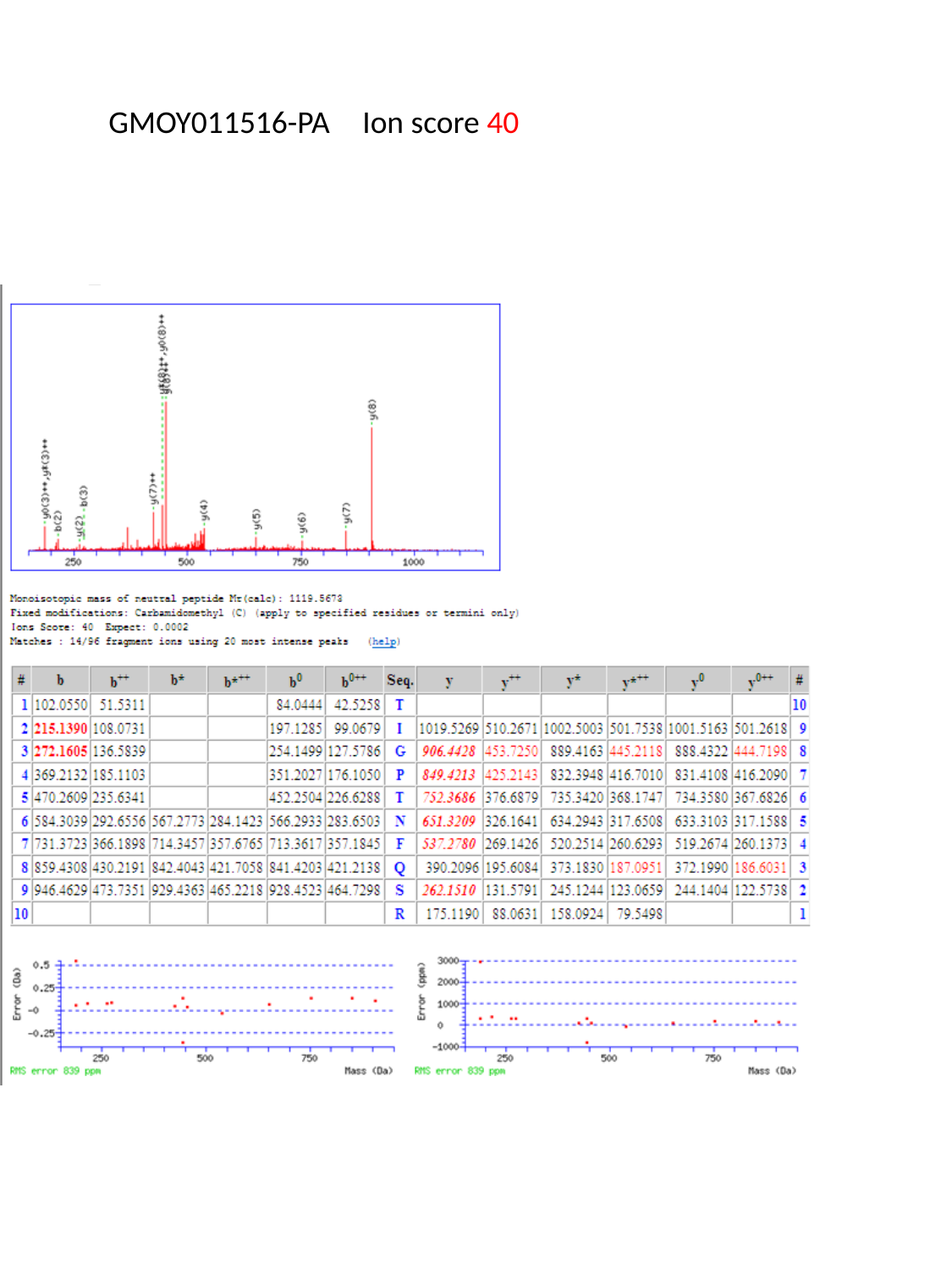

GMOY011516-PA	Ion score 40

## Slide 49
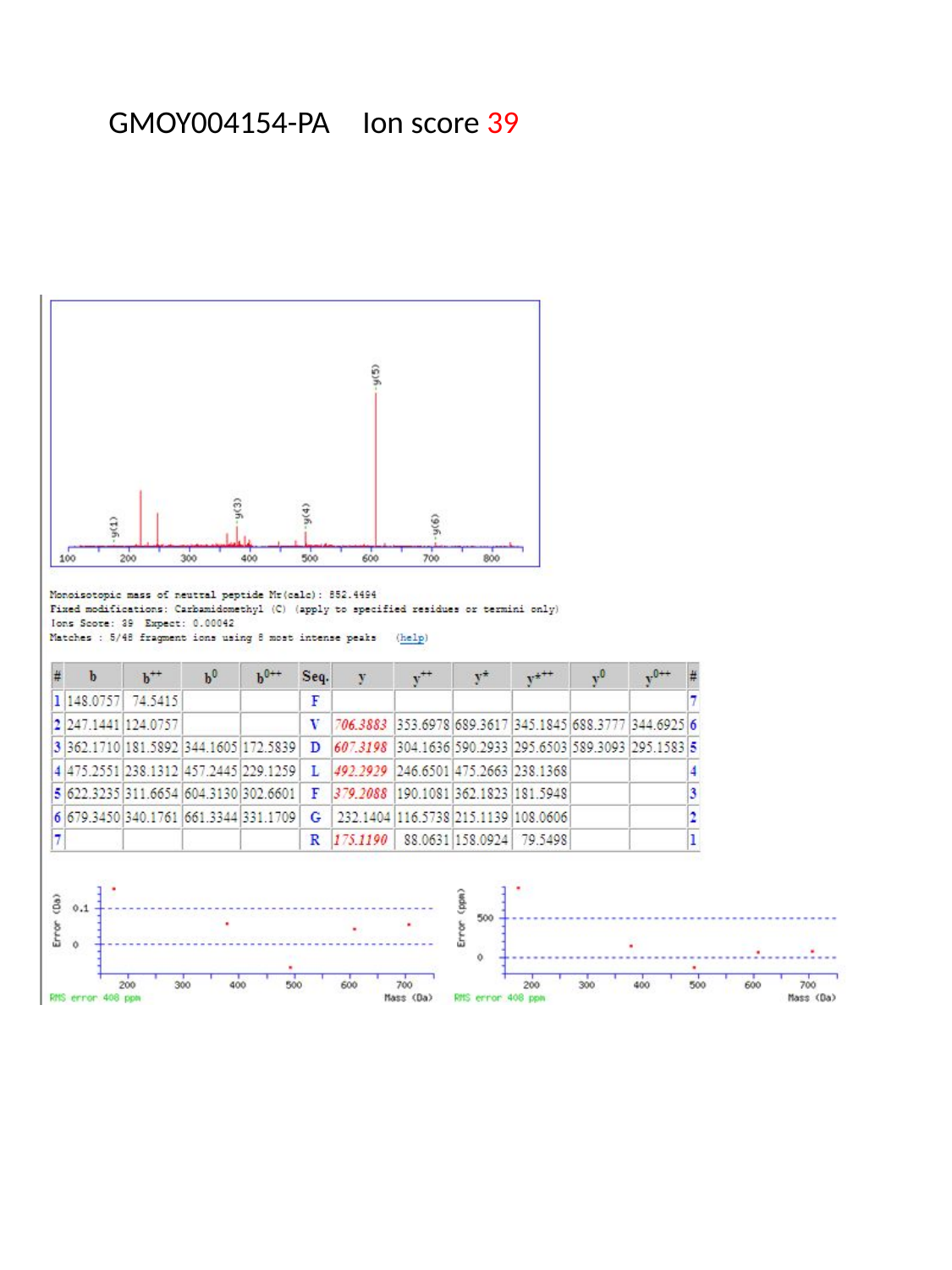

GMOY004154-PA	Ion score 39

## Slide 50
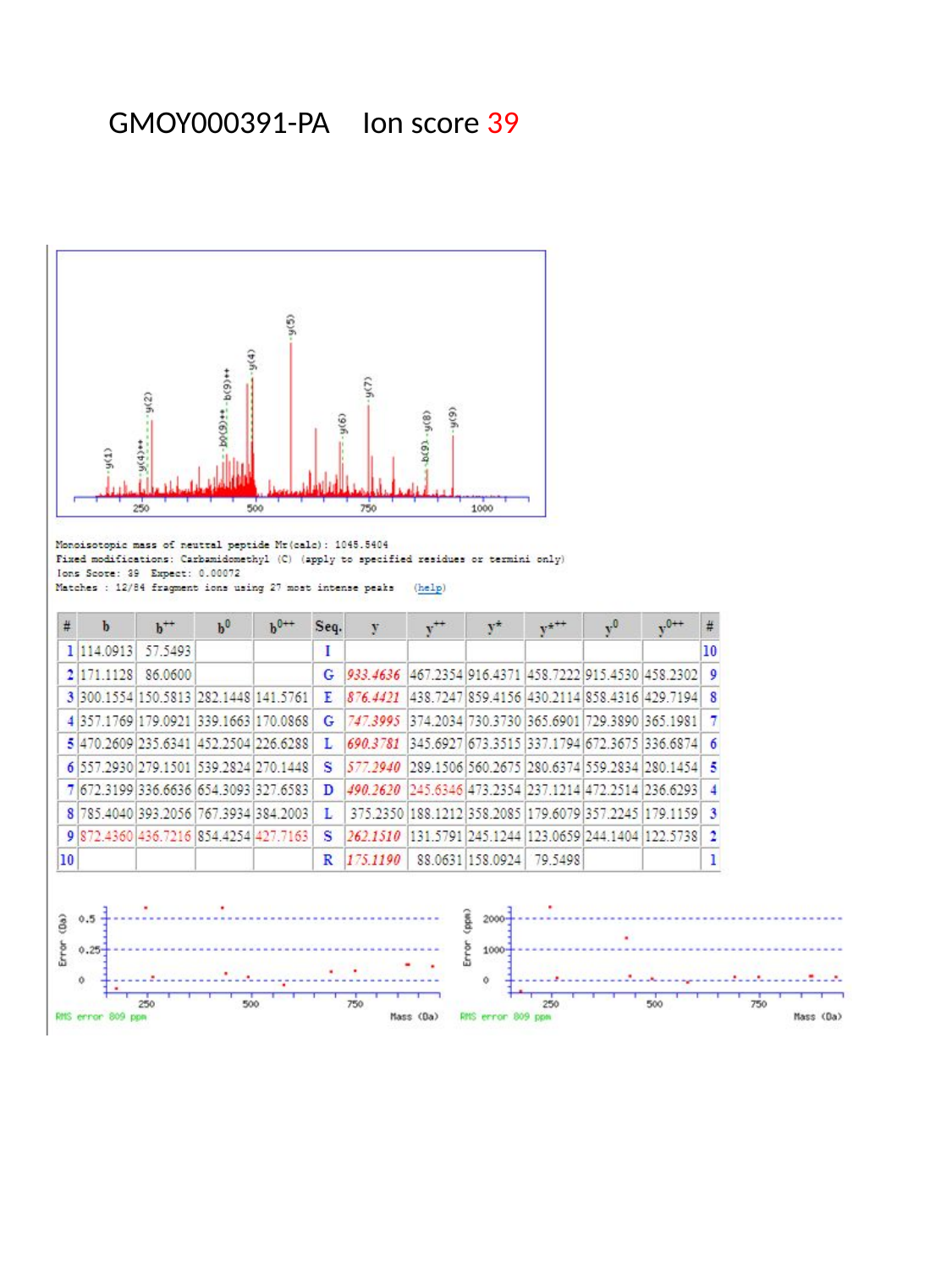

GMOY000391-PA	Ion score 39

## Slide 51
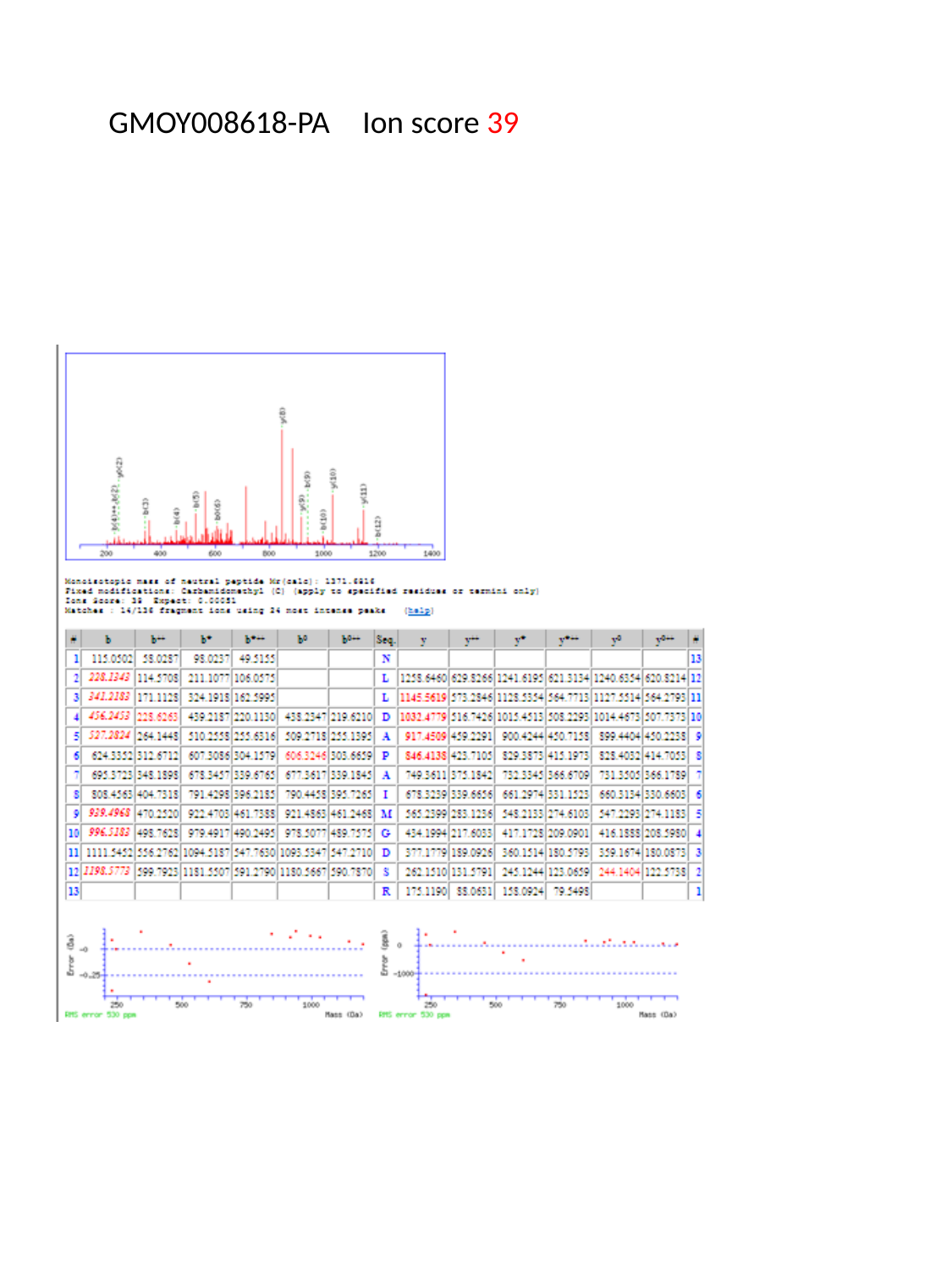

GMOY008618-PA	Ion score 39

## Slide 52
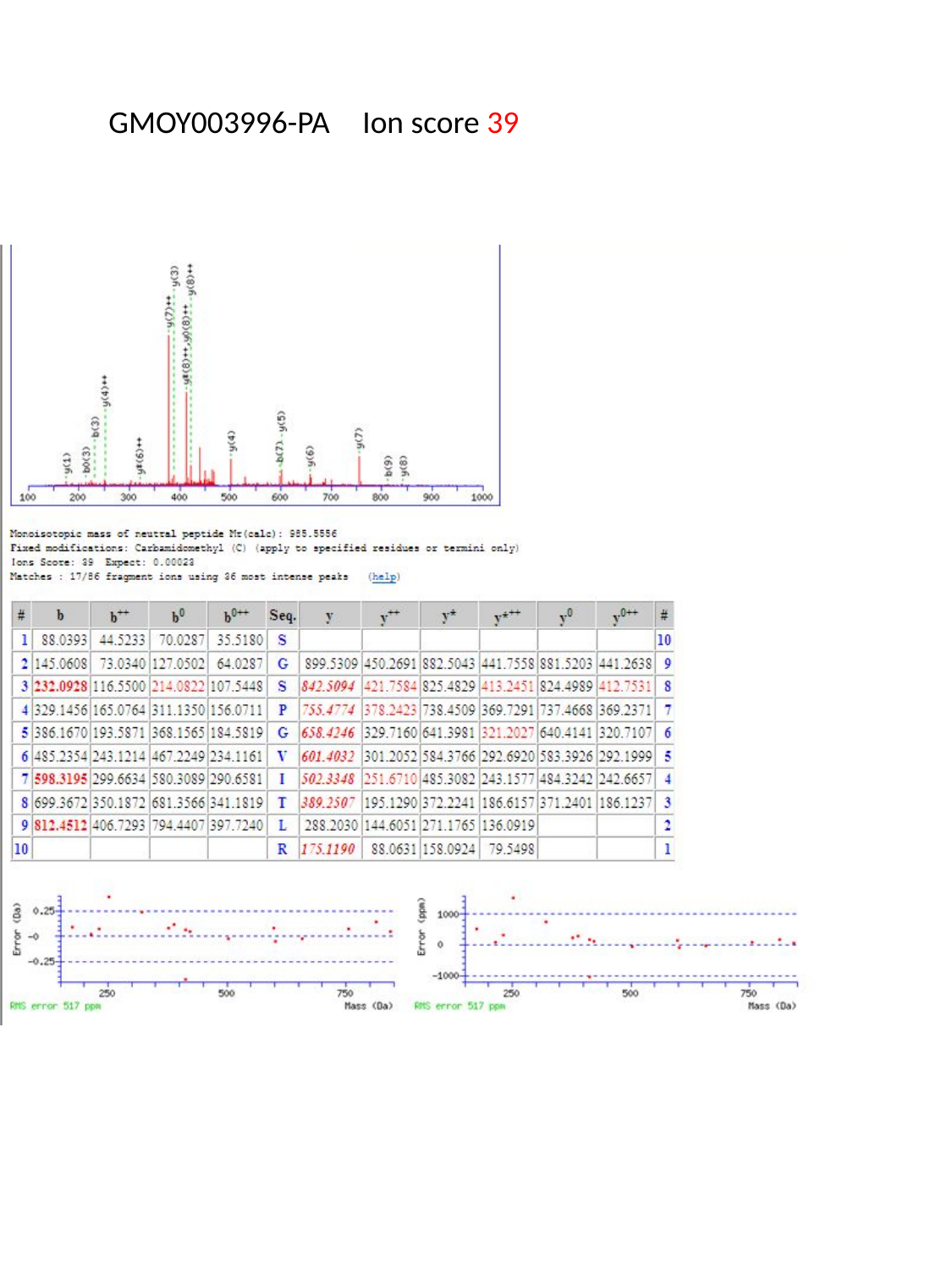

GMOY003996-PA 	Ion score 39

## Slide 53
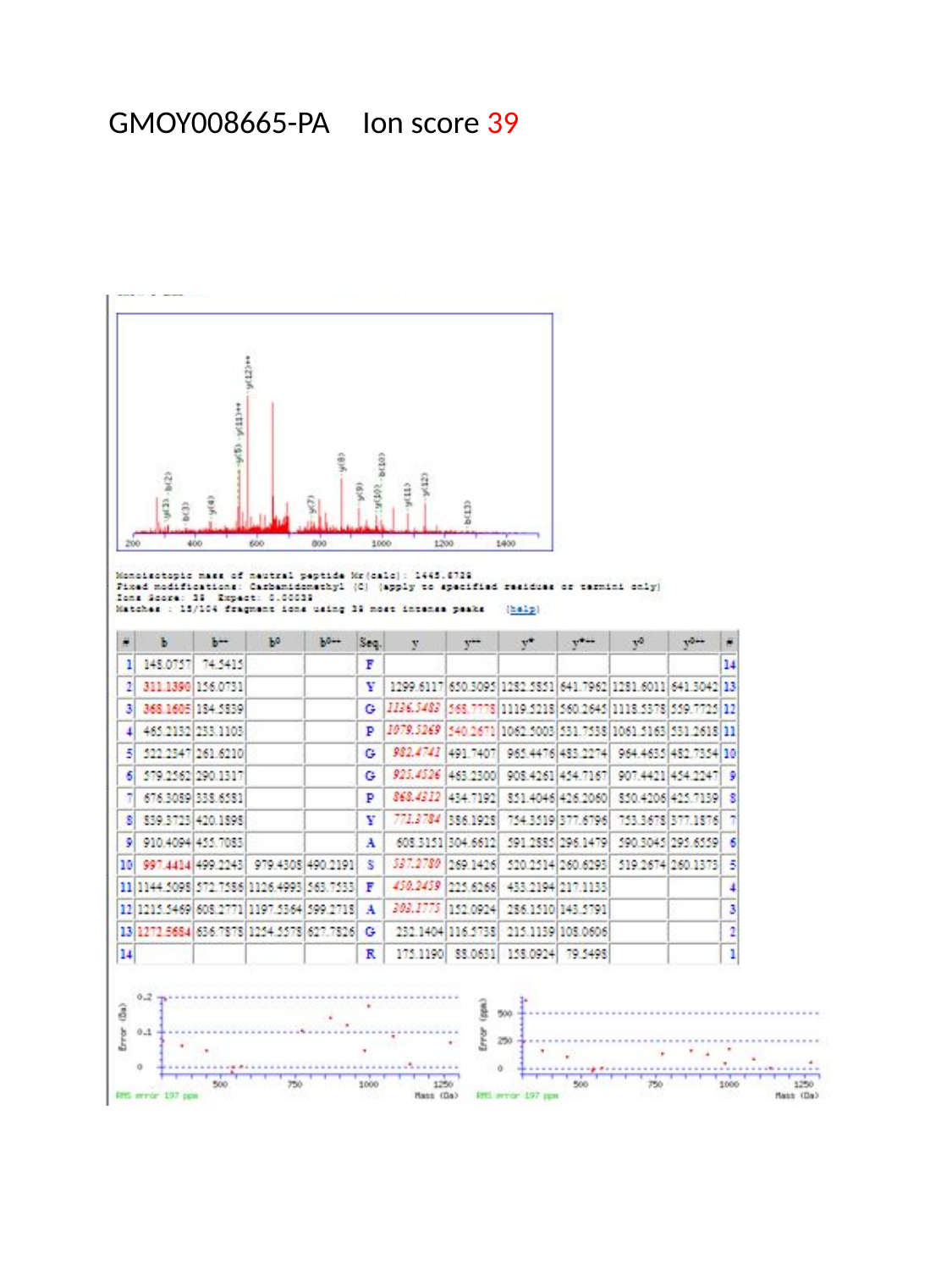

GMOY008665-PA 	Ion score 39

## Slide 54
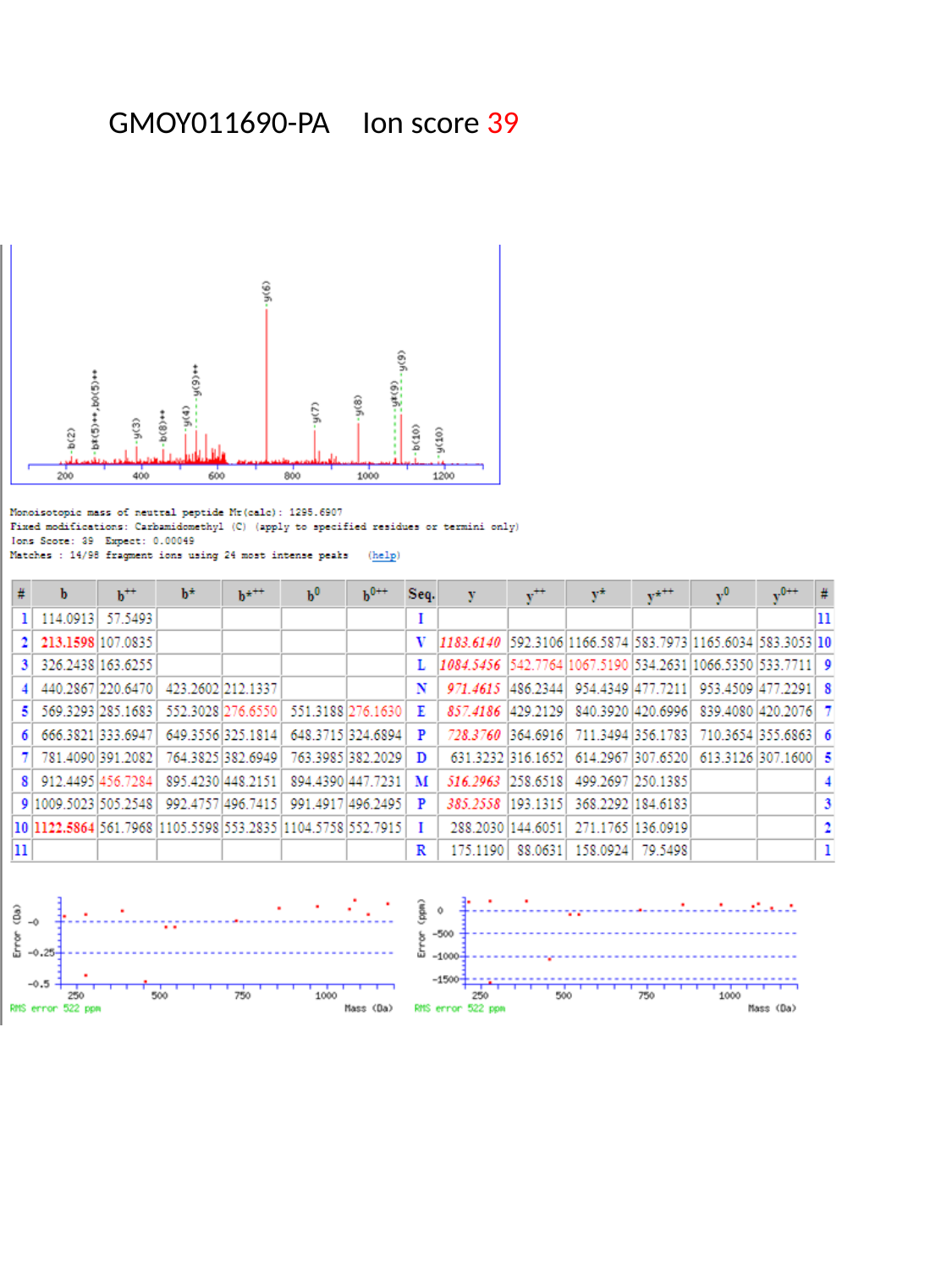

GMOY011690-PA 	Ion score 39

## Slide 55
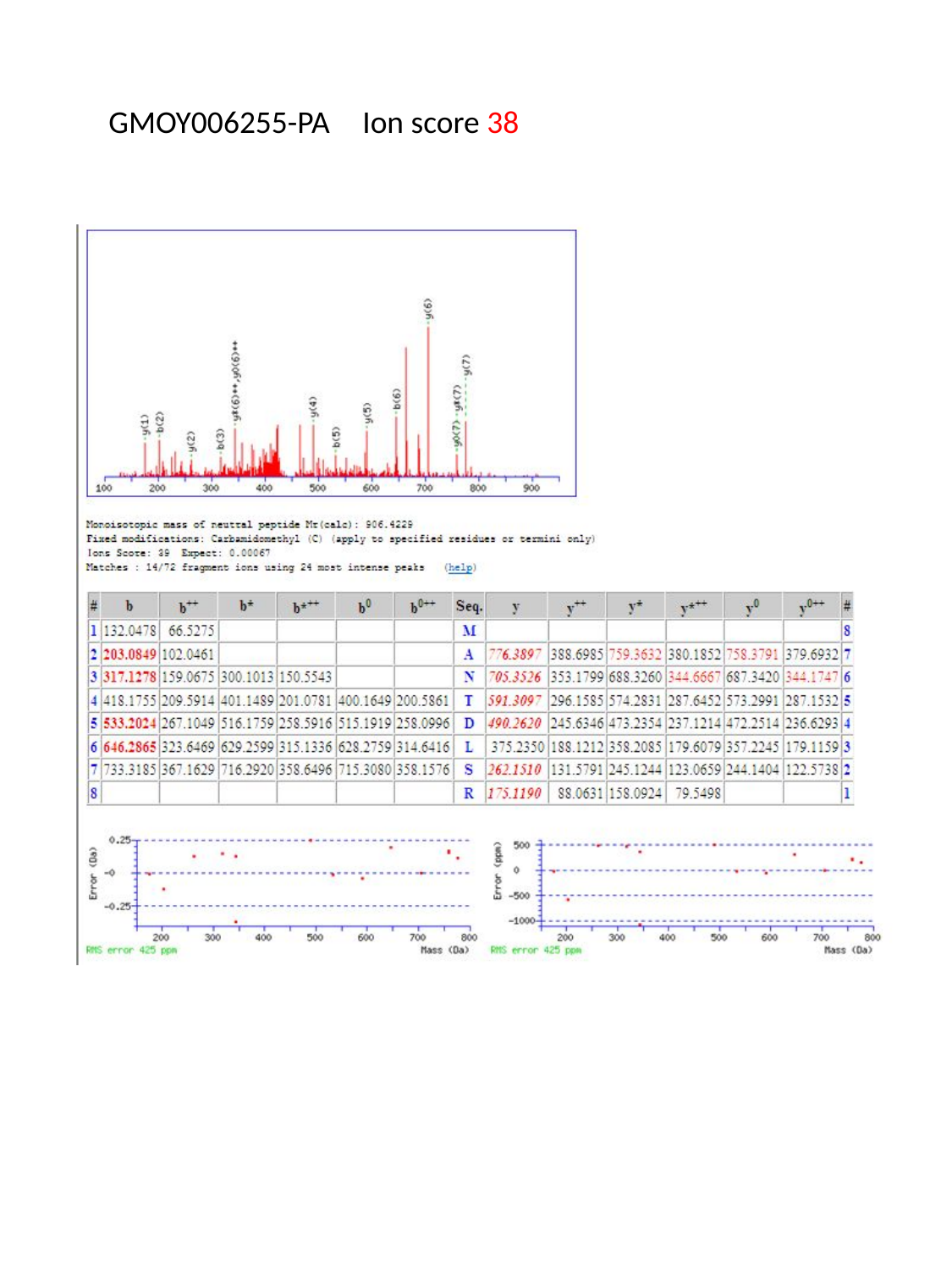

GMOY006255-PA 	Ion score 38

## Slide 56
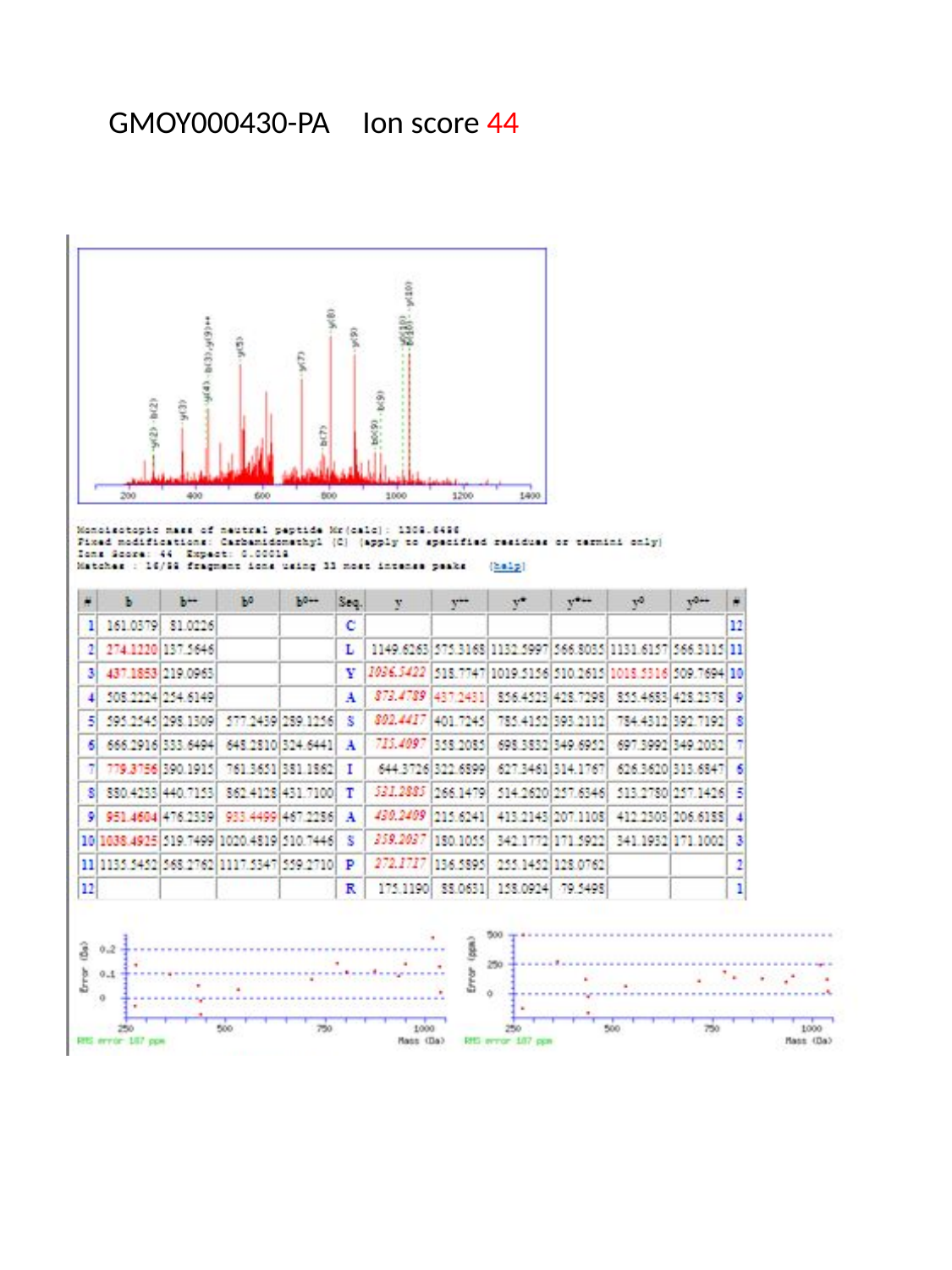

GMOY000430-PA 	Ion score 44

## Slide 57
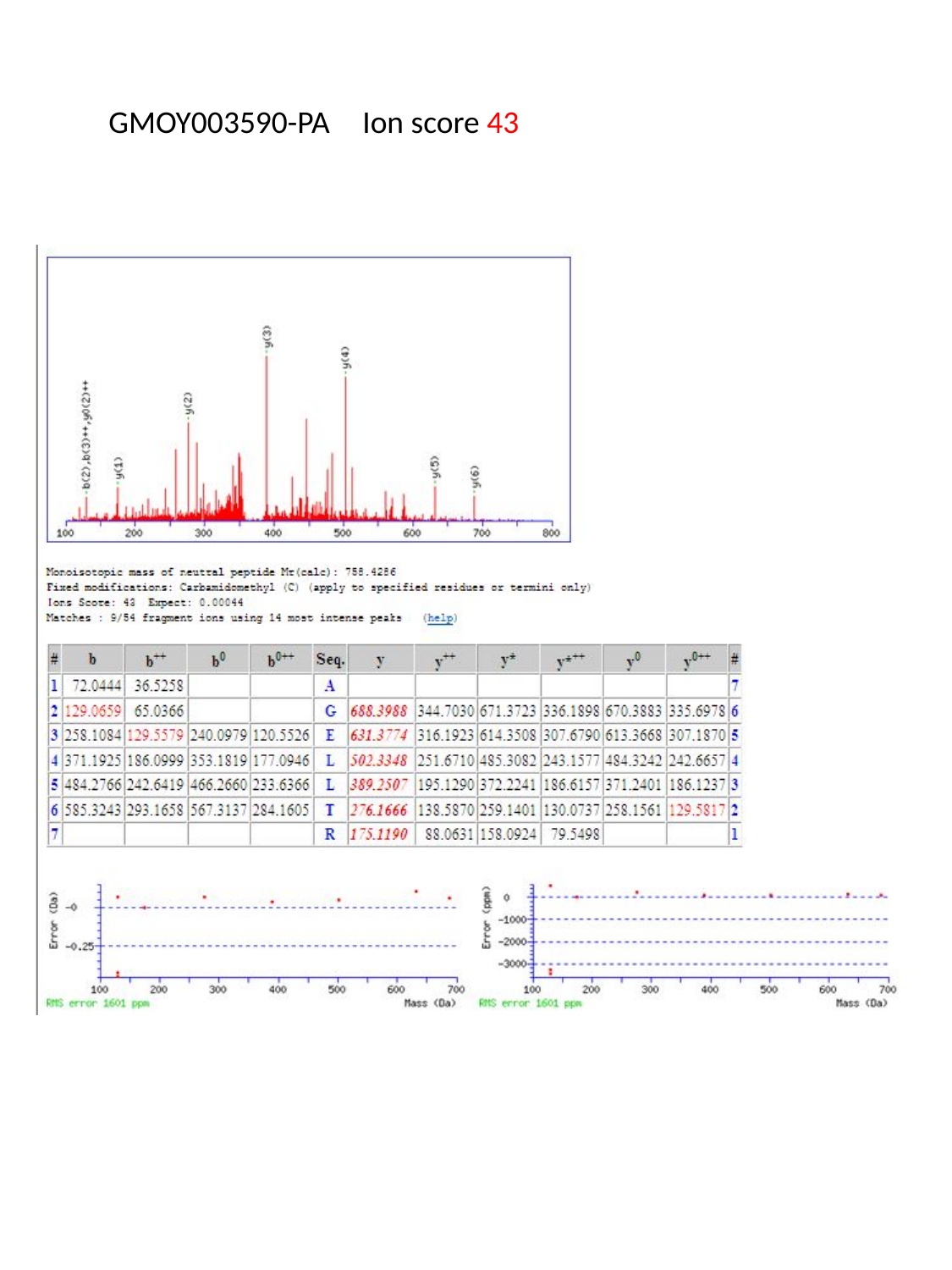

GMOY003590-PA 	Ion score 43

## Slide 58
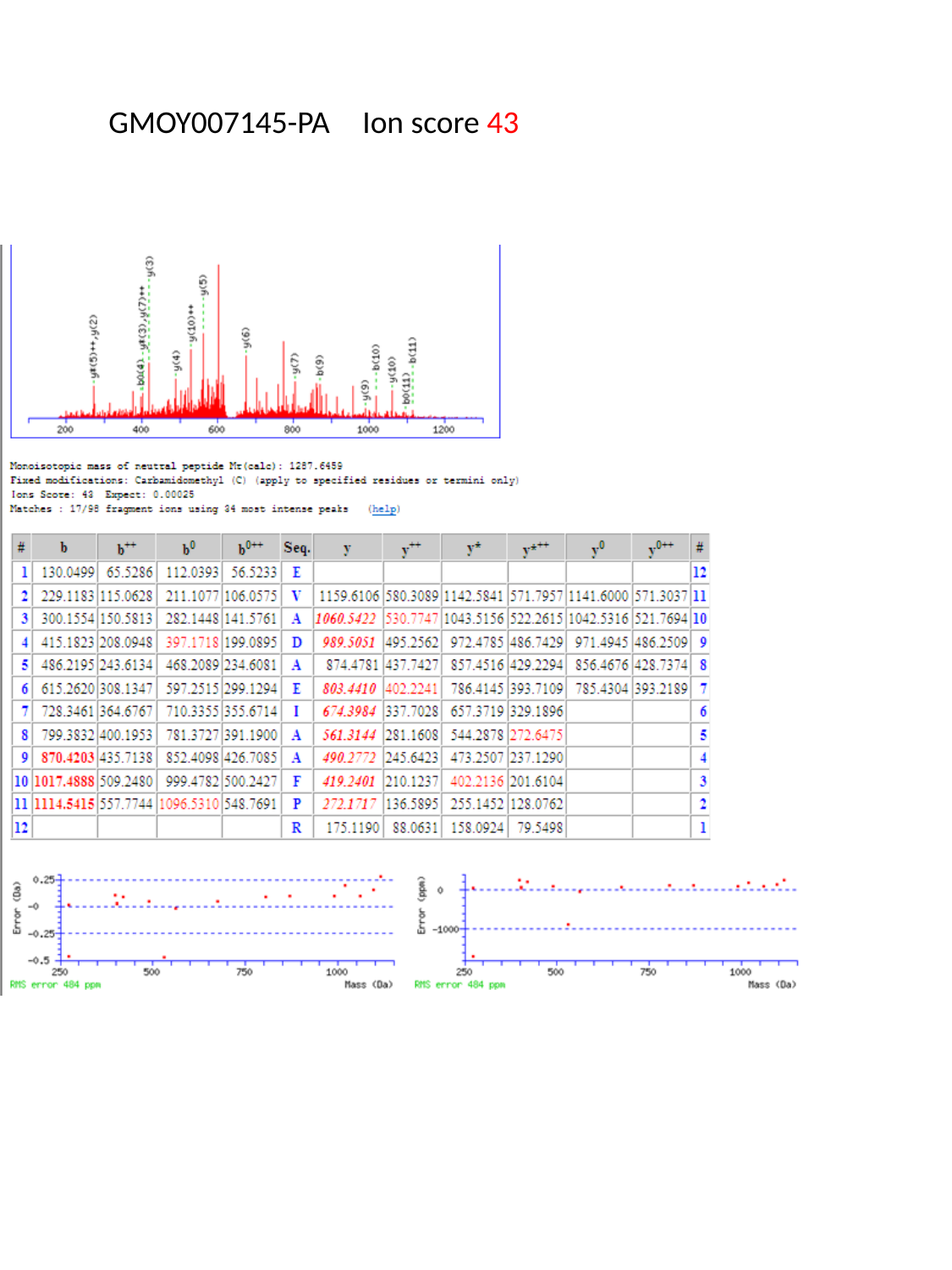

GMOY007145-PA 	Ion score 43

## Slide 59
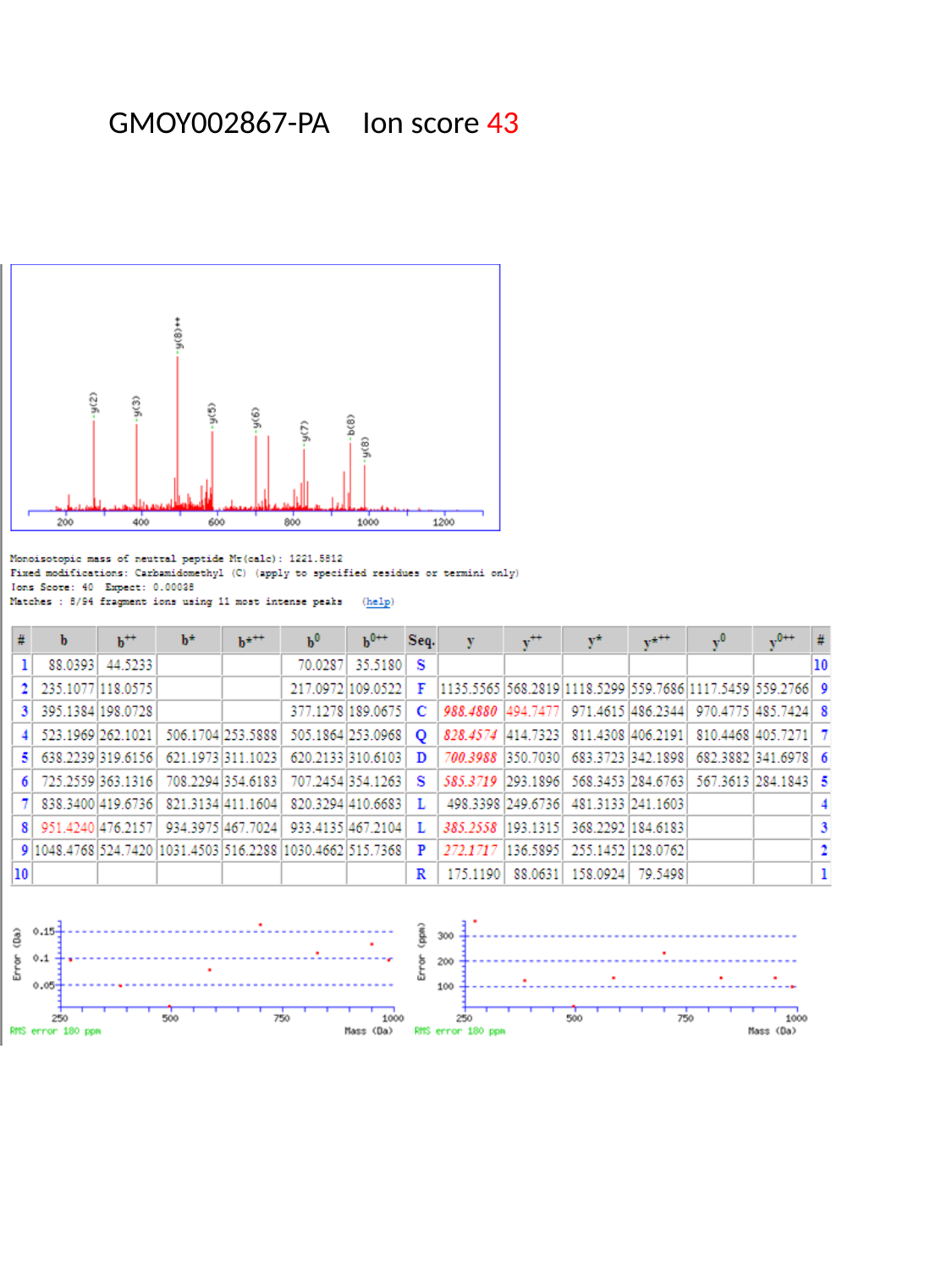

GMOY002867-PA 	Ion score 43

## Slide 60
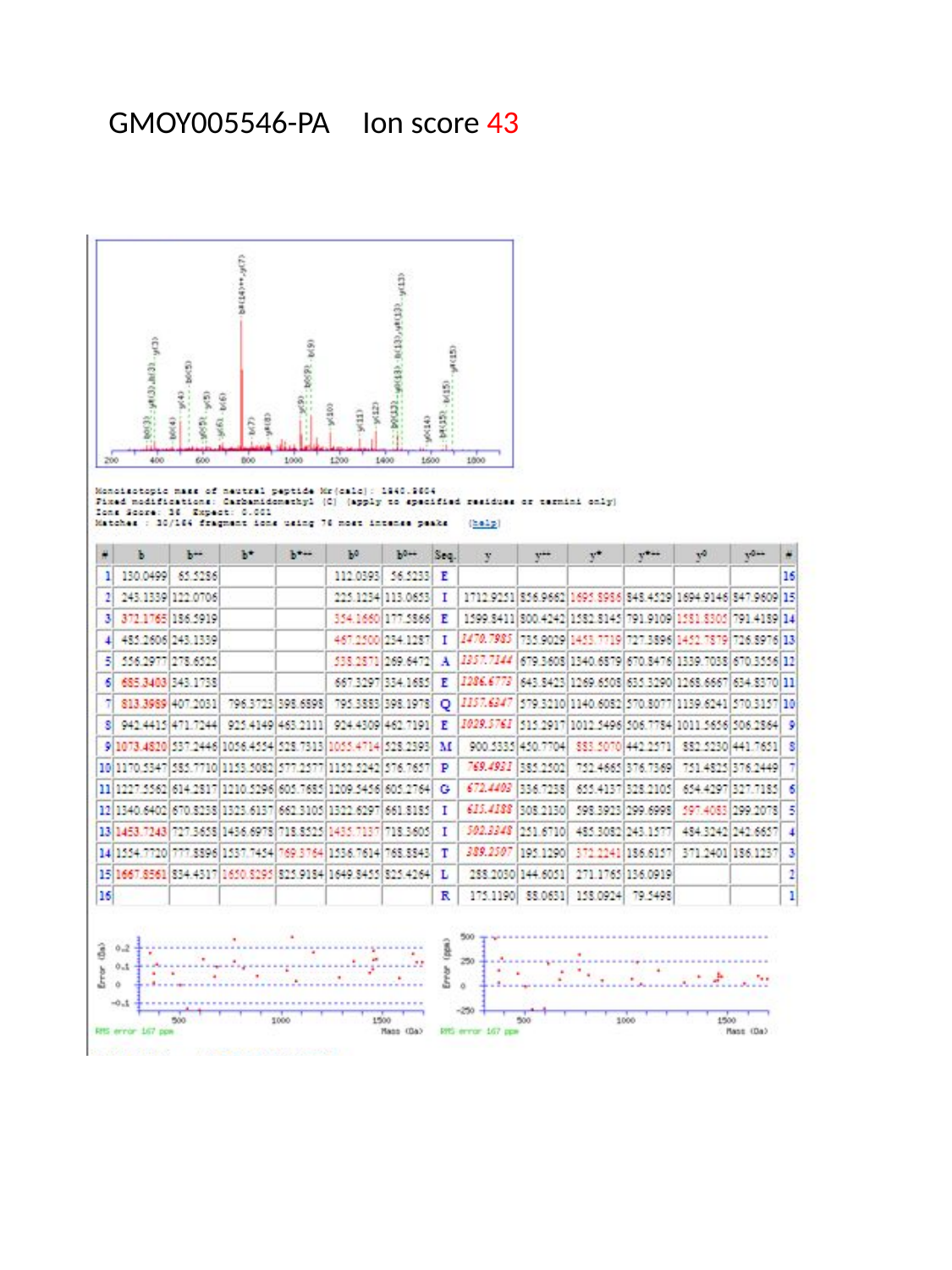

GMOY005546-PA 	Ion score 43

## Slide 61
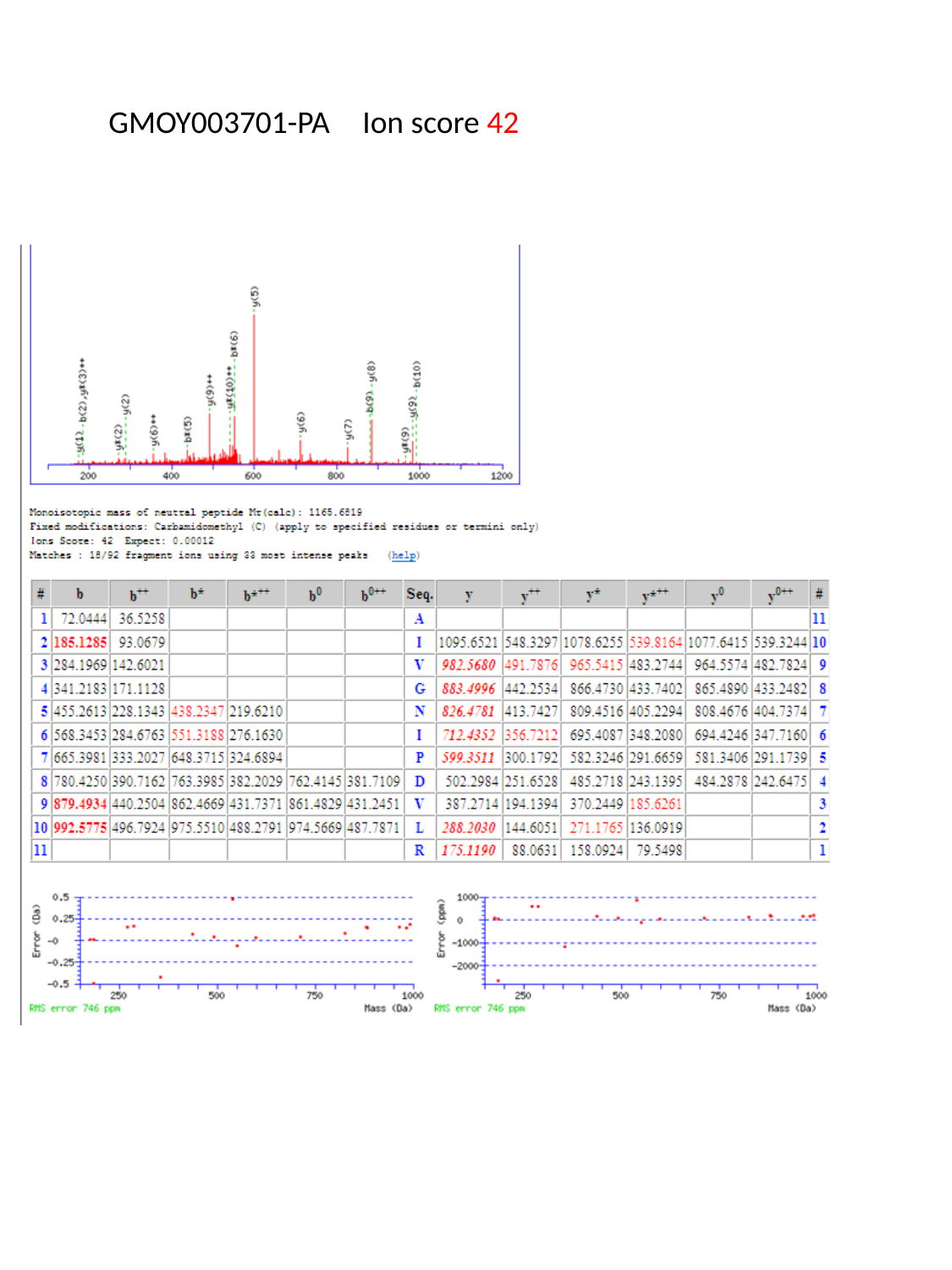

GMOY003701-PA 	Ion score 42

## Slide 62
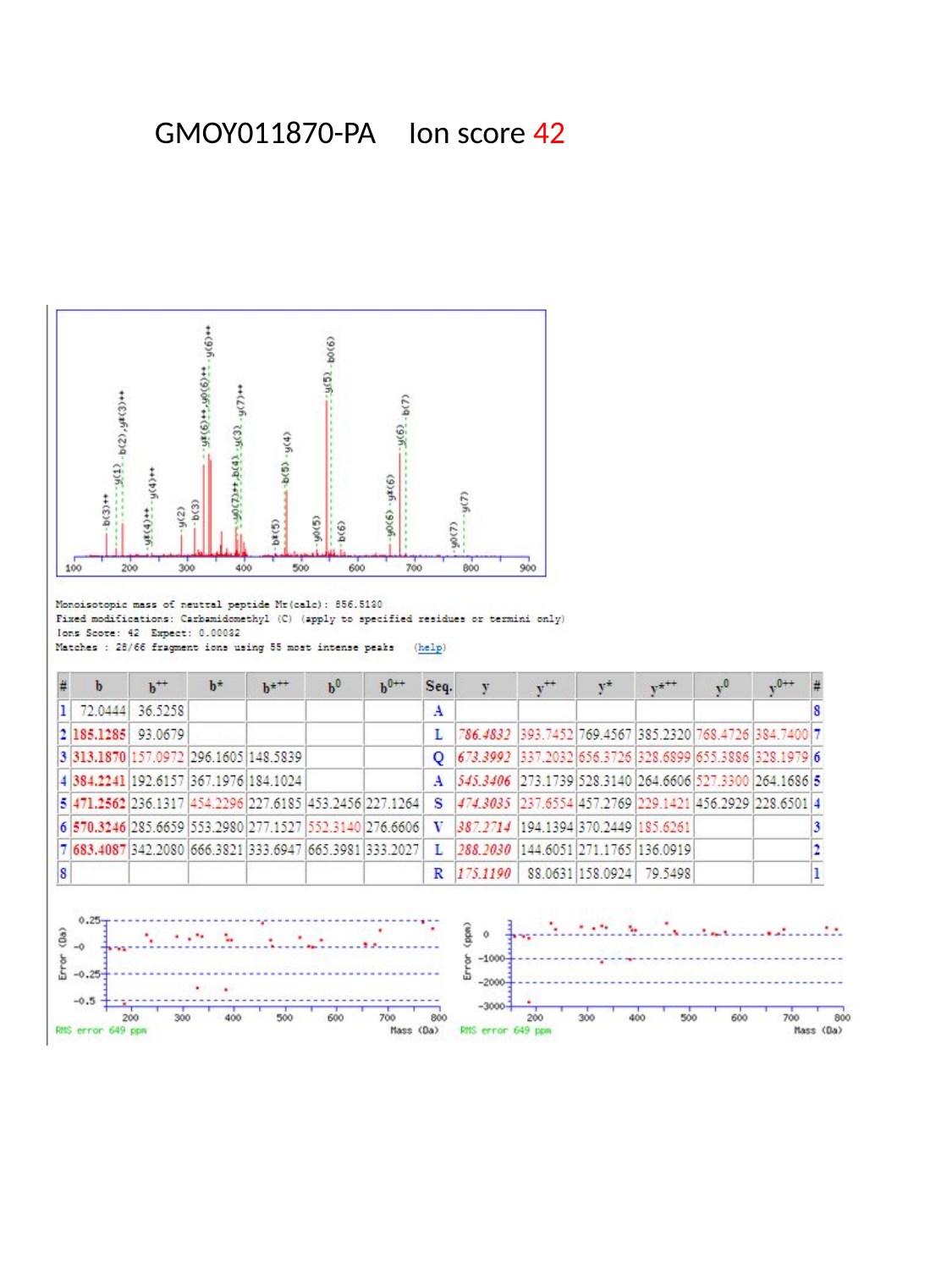

GMOY011870-PA 	Ion score 42

## Slide 63
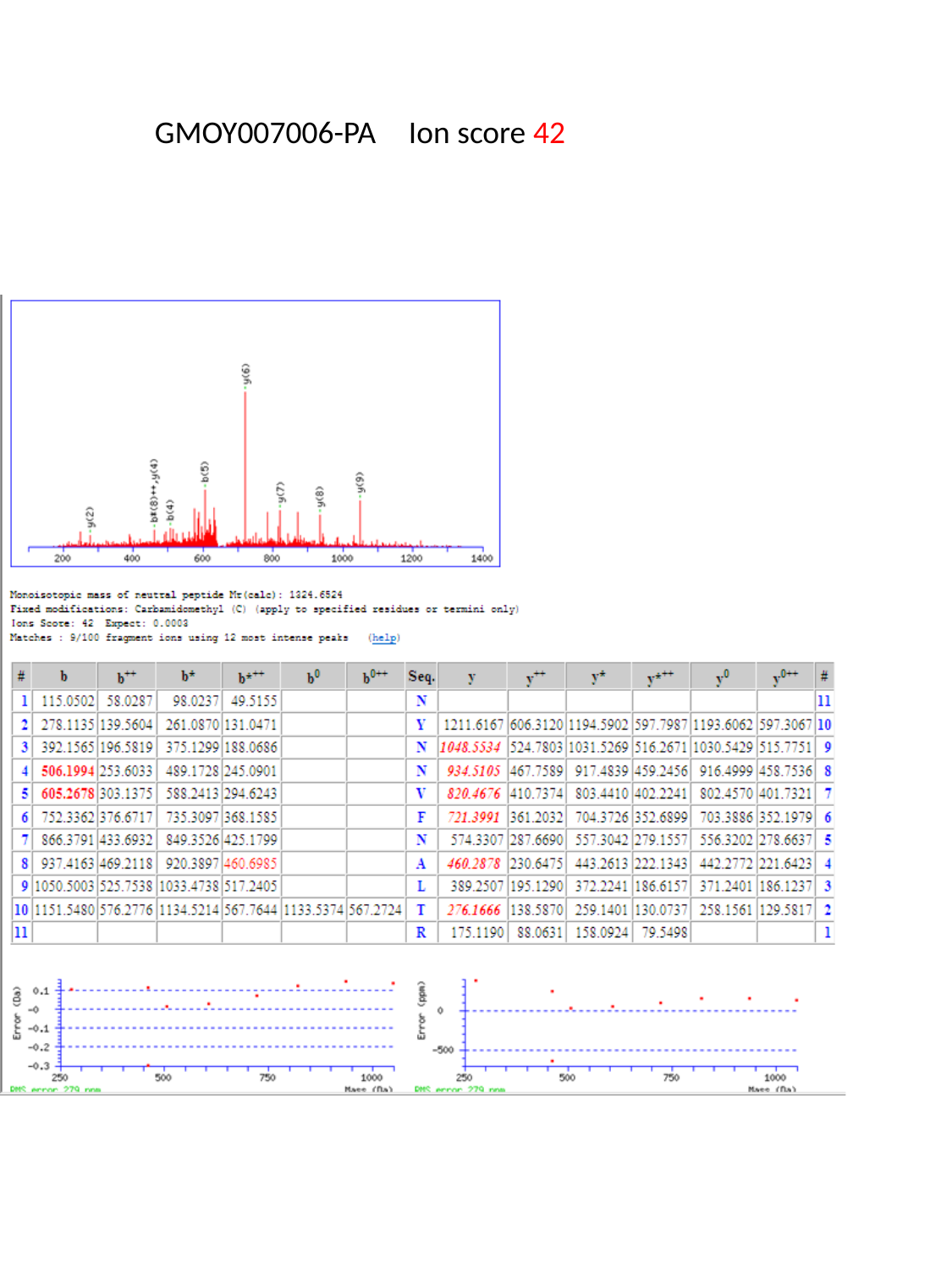

GMOY007006-PA 	Ion score 42

## Slide 64
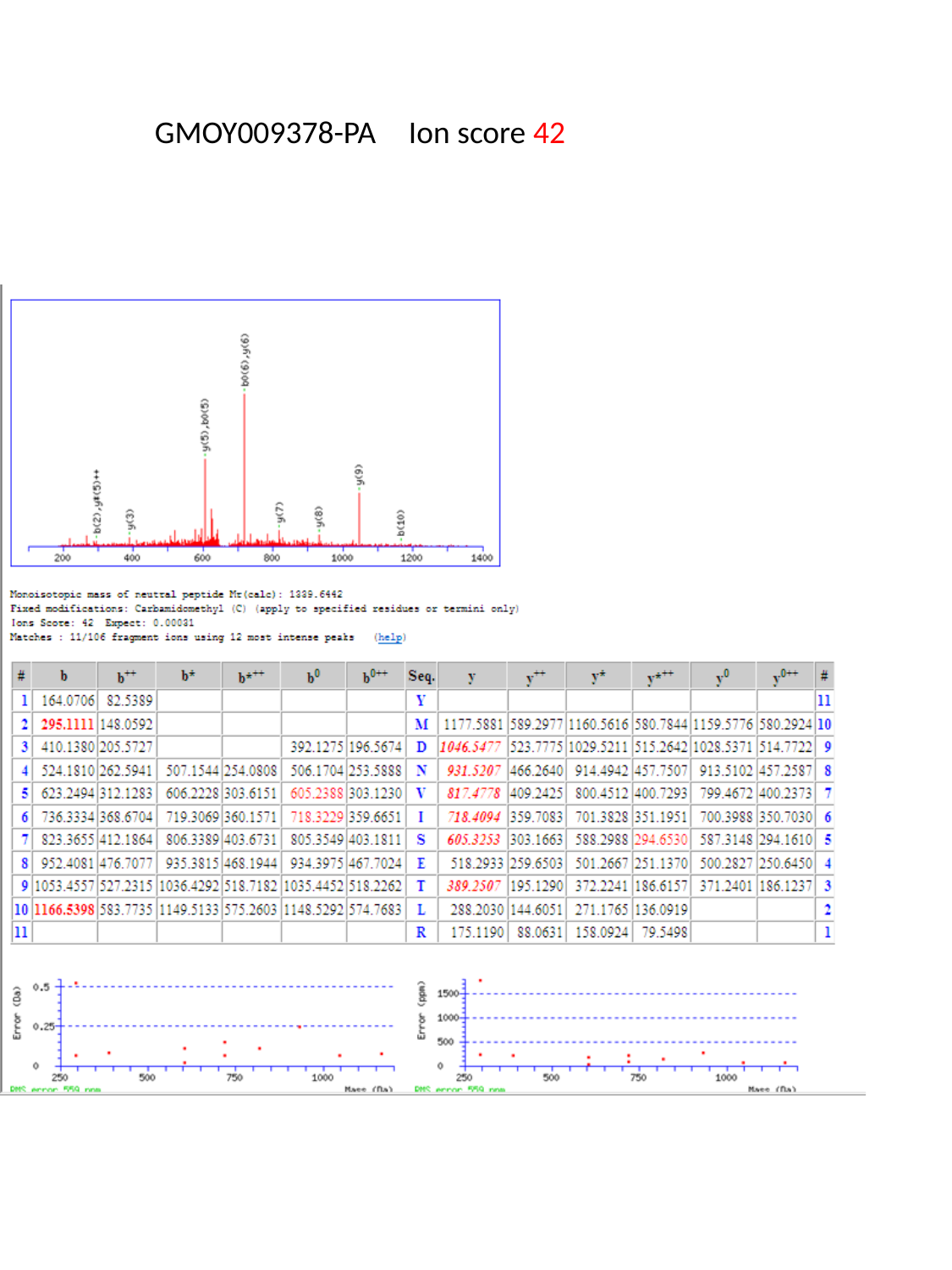

GMOY009378-PA 	Ion score 42

## Slide 65
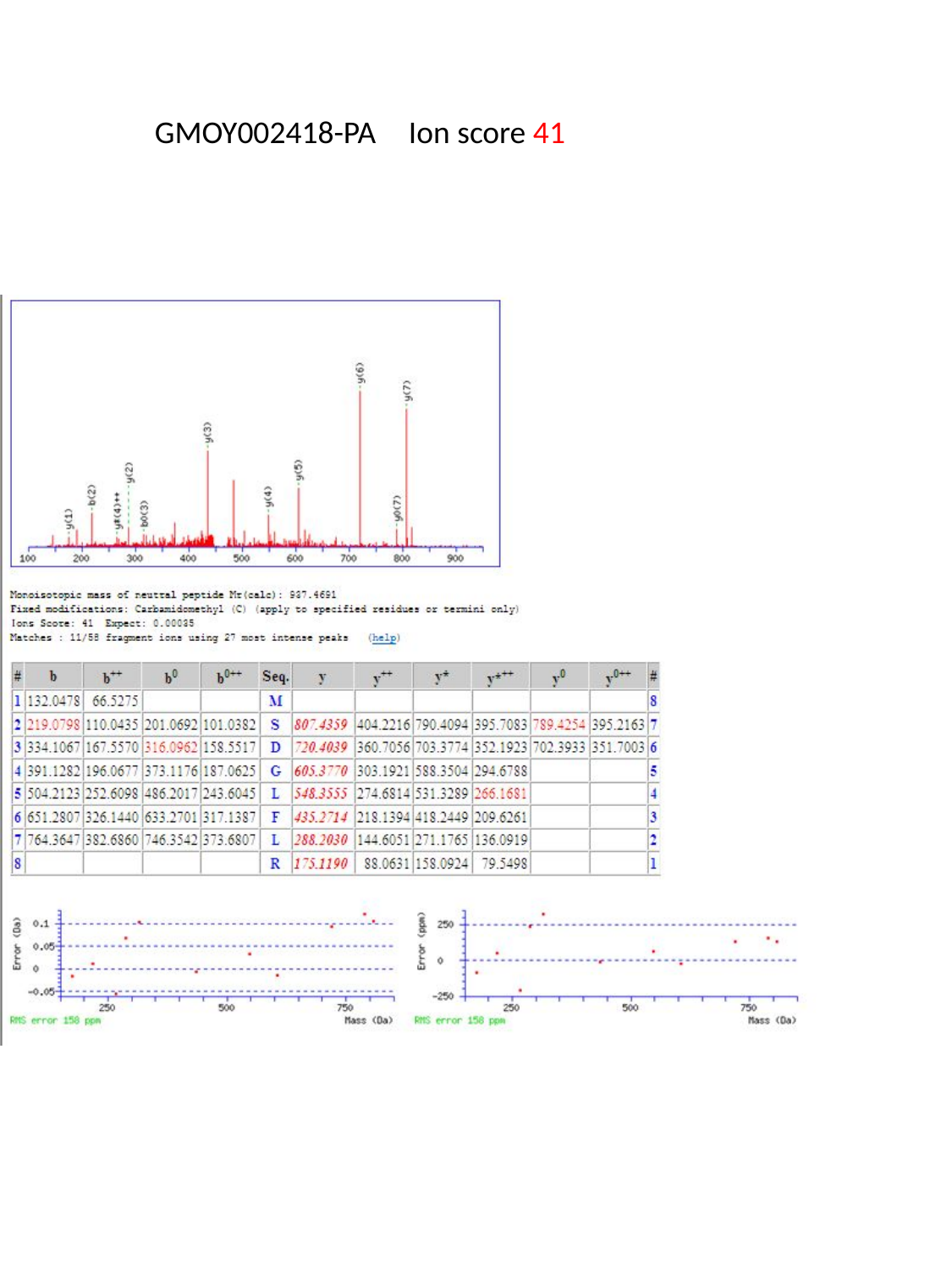

GMOY002418-PA 	Ion score 41

## Slide 66
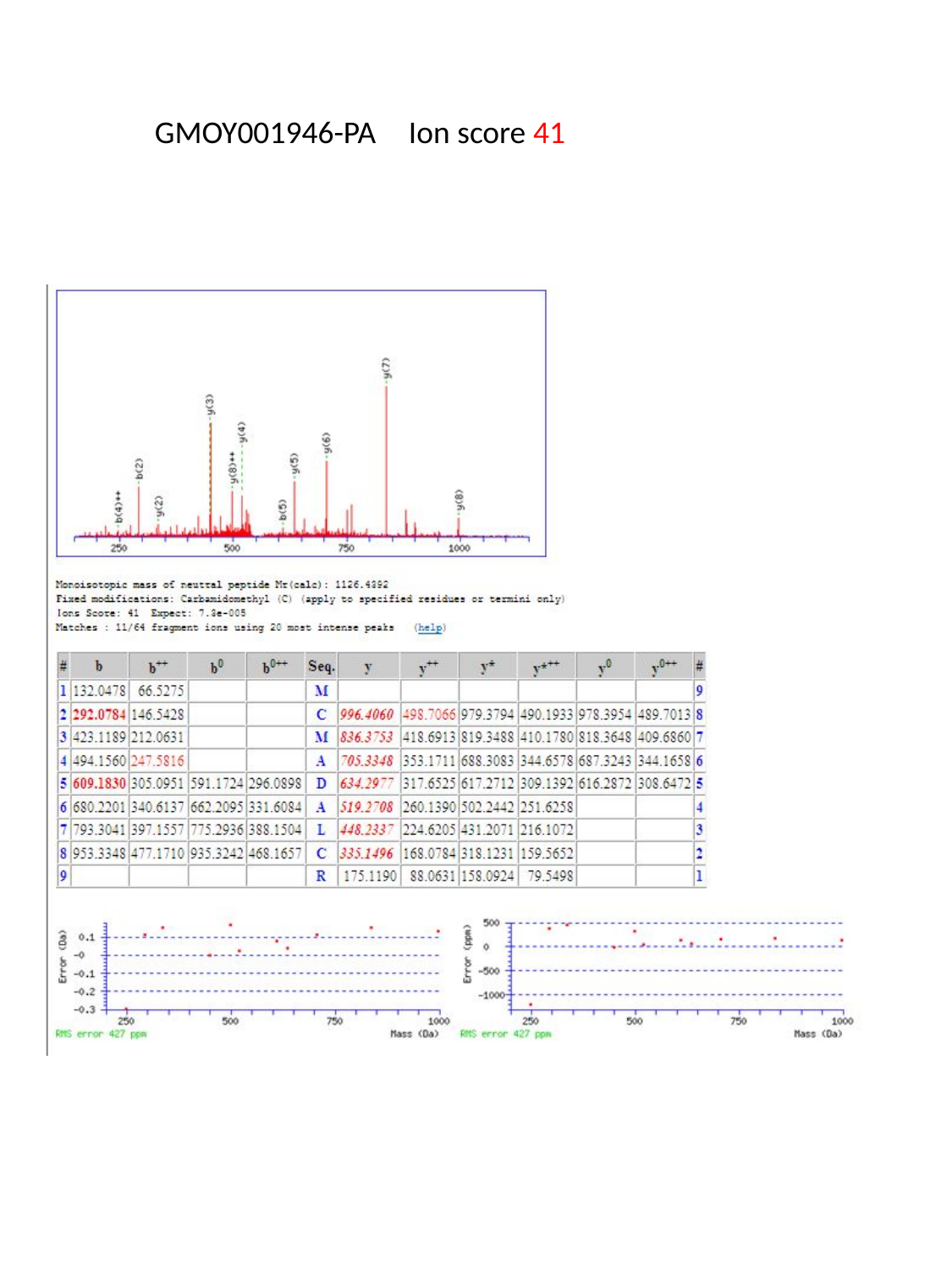

GMOY001946-PA 	Ion score 41

## Slide 67
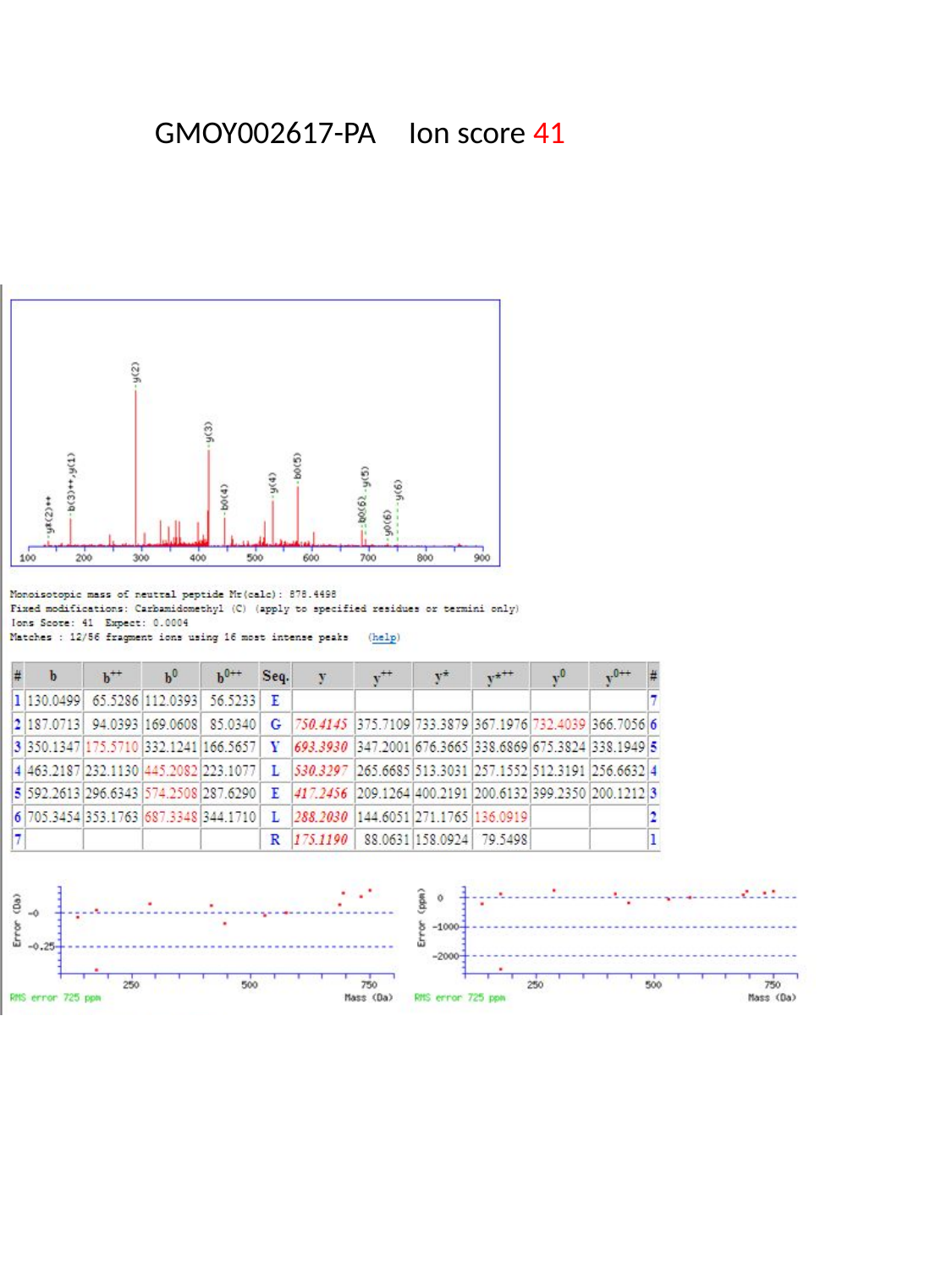

GMOY002617-PA 	Ion score 41

## Slide 68
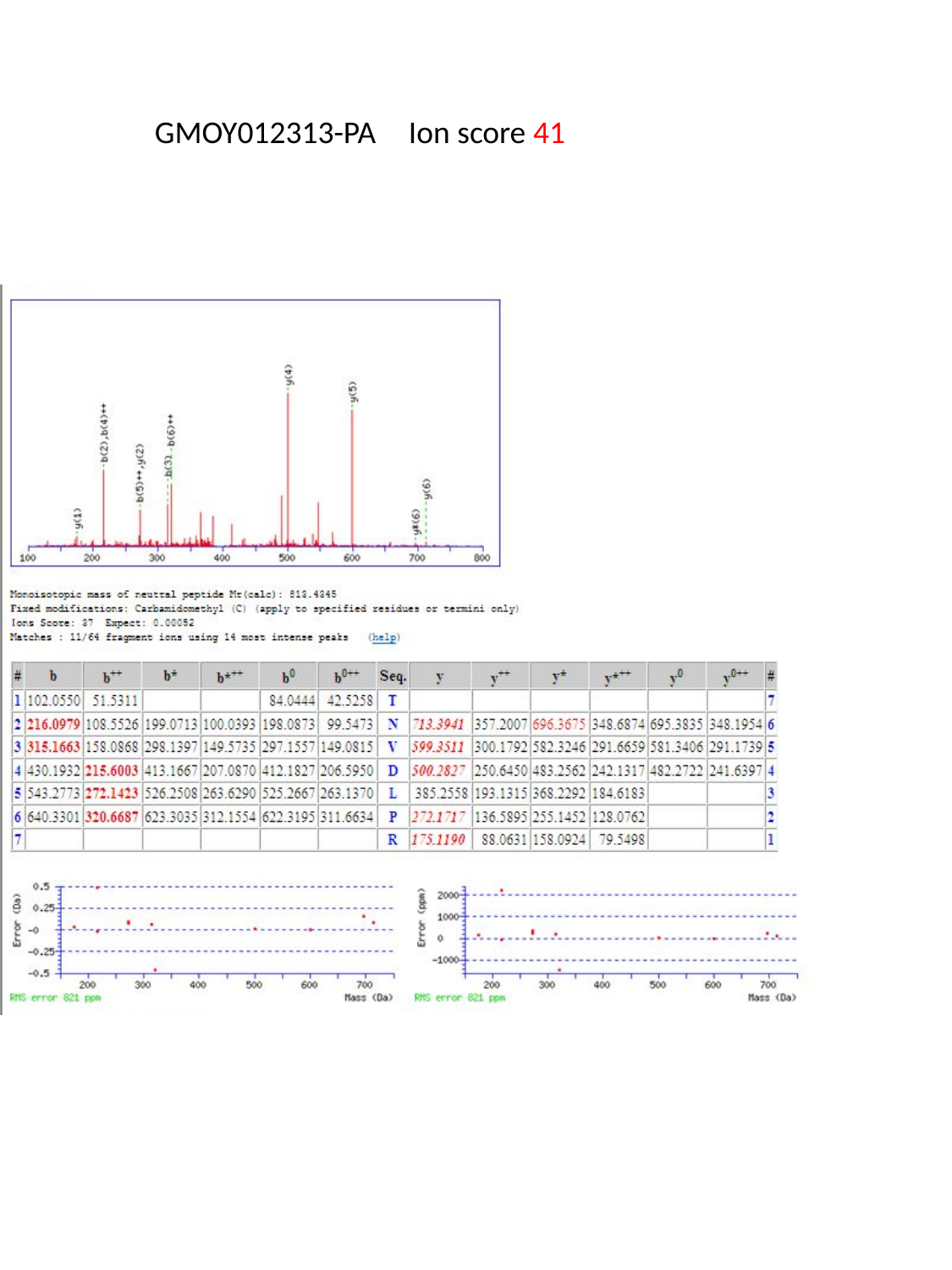

GMOY012313-PA	Ion score 41

## Slide 69
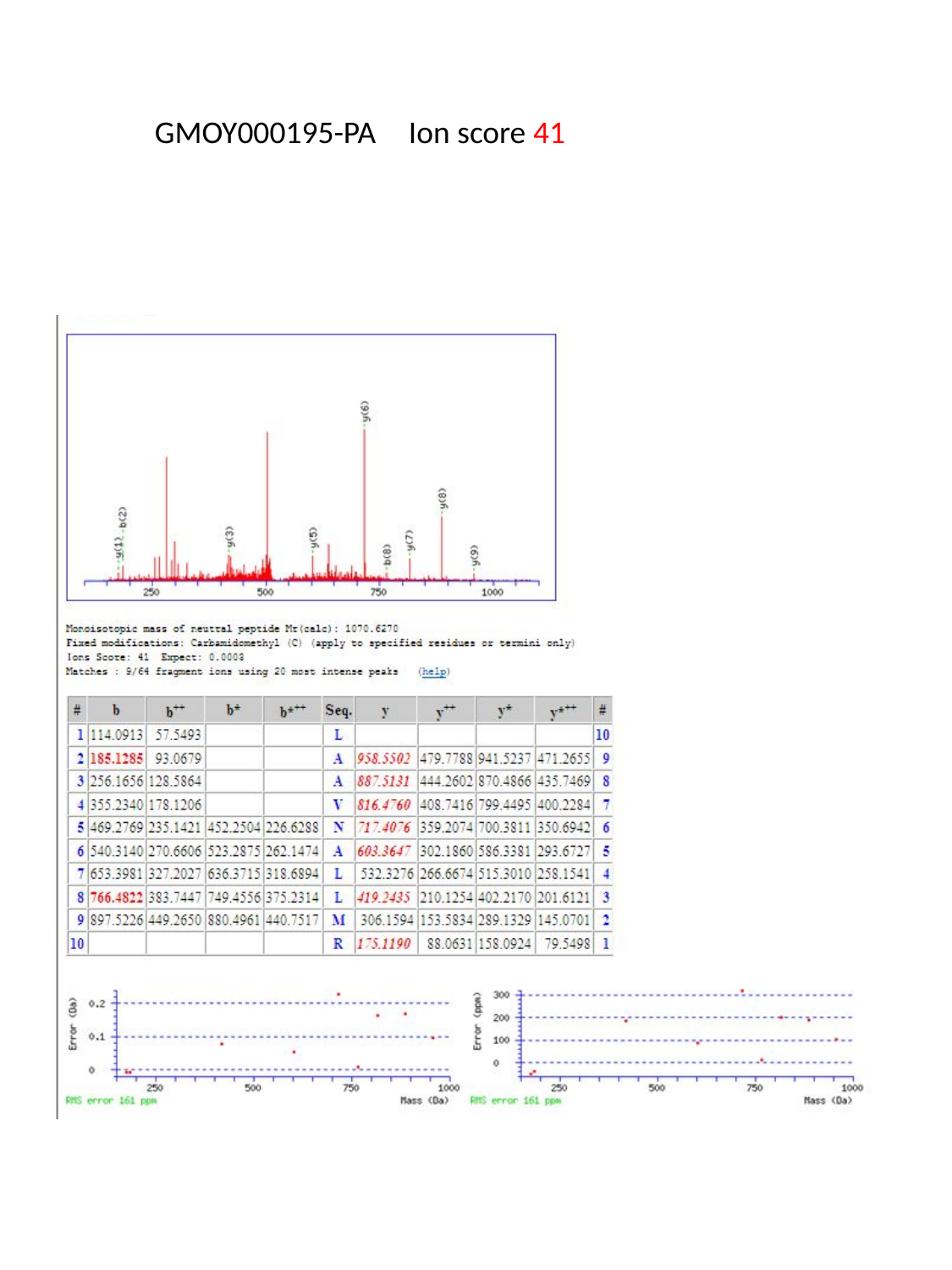

GMOY000195-PA	Ion score 41

## Slide 70
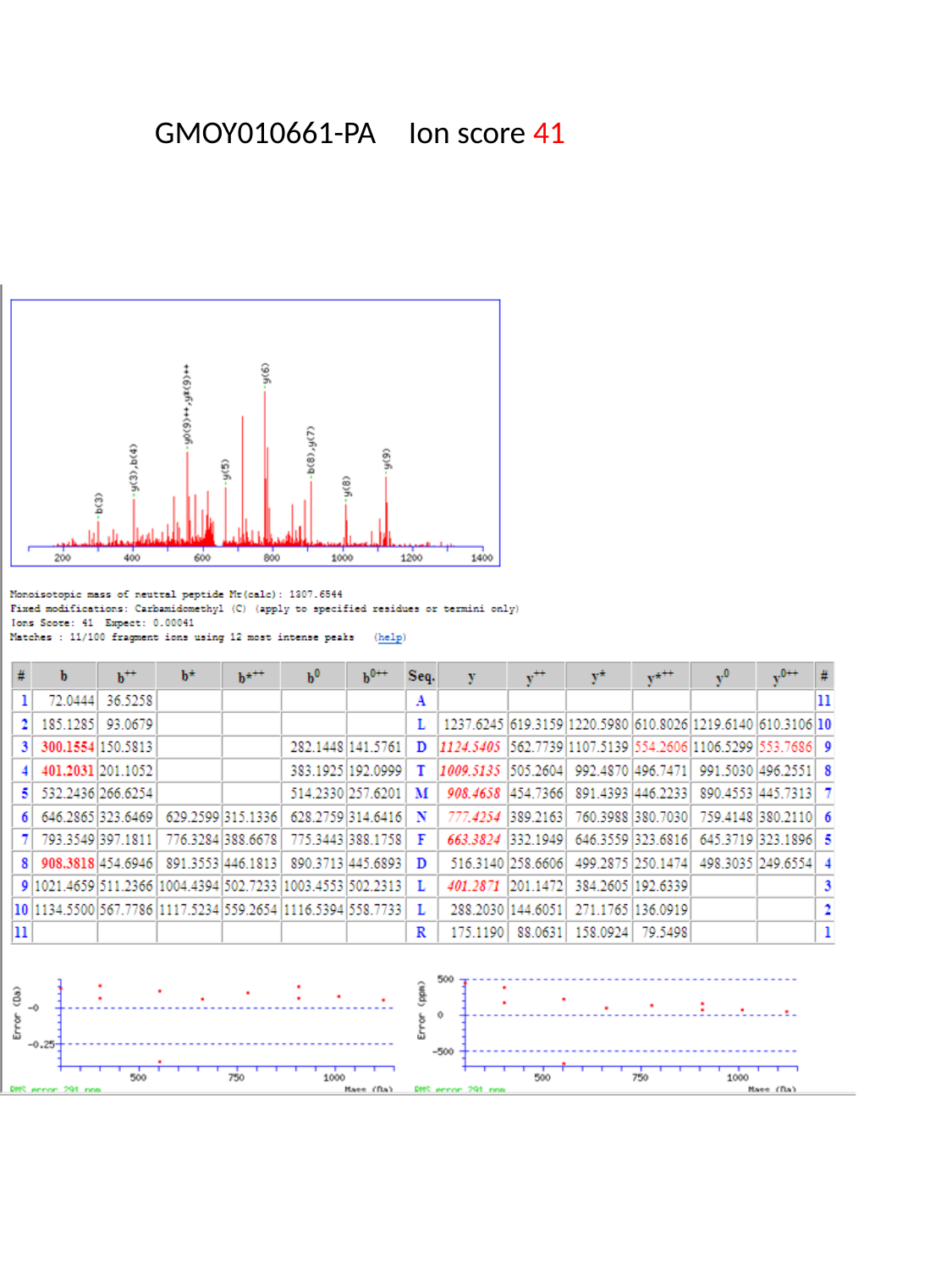

GMOY010661-PA	Ion score 41

## Slide 71
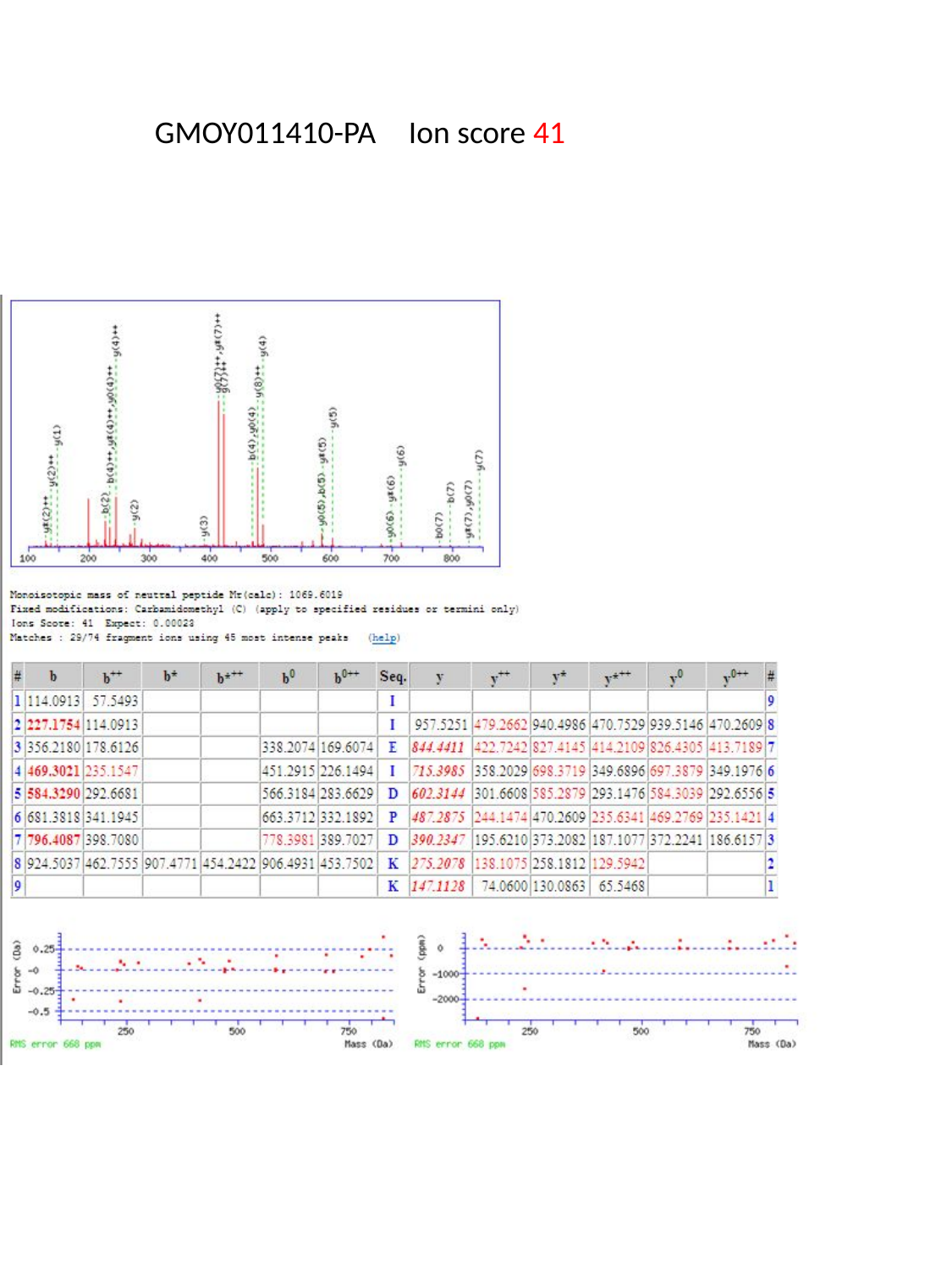

GMOY011410-PA	Ion score 41

## Slide 72
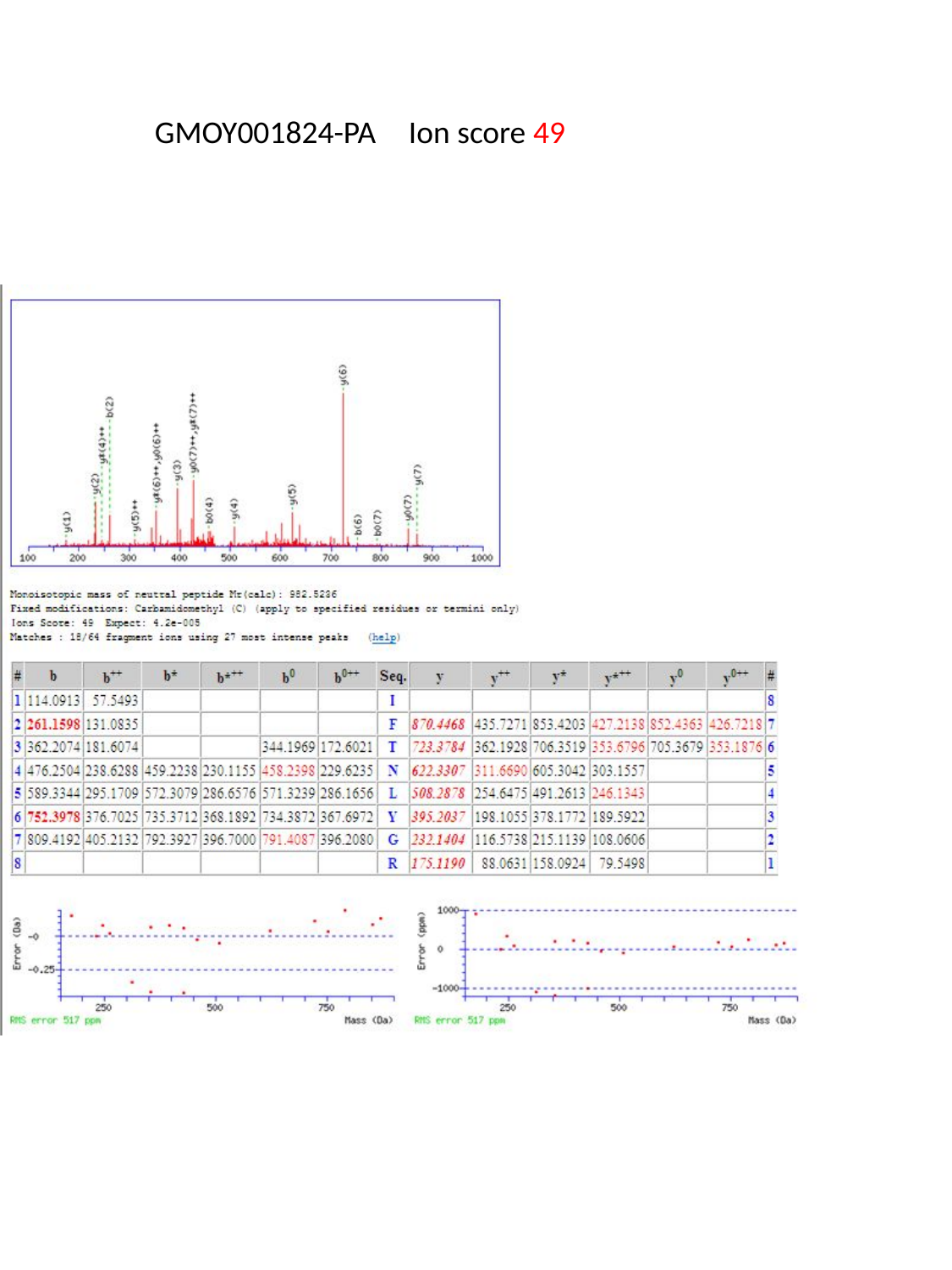

GMOY001824-PA 	Ion score 49

## Slide 73
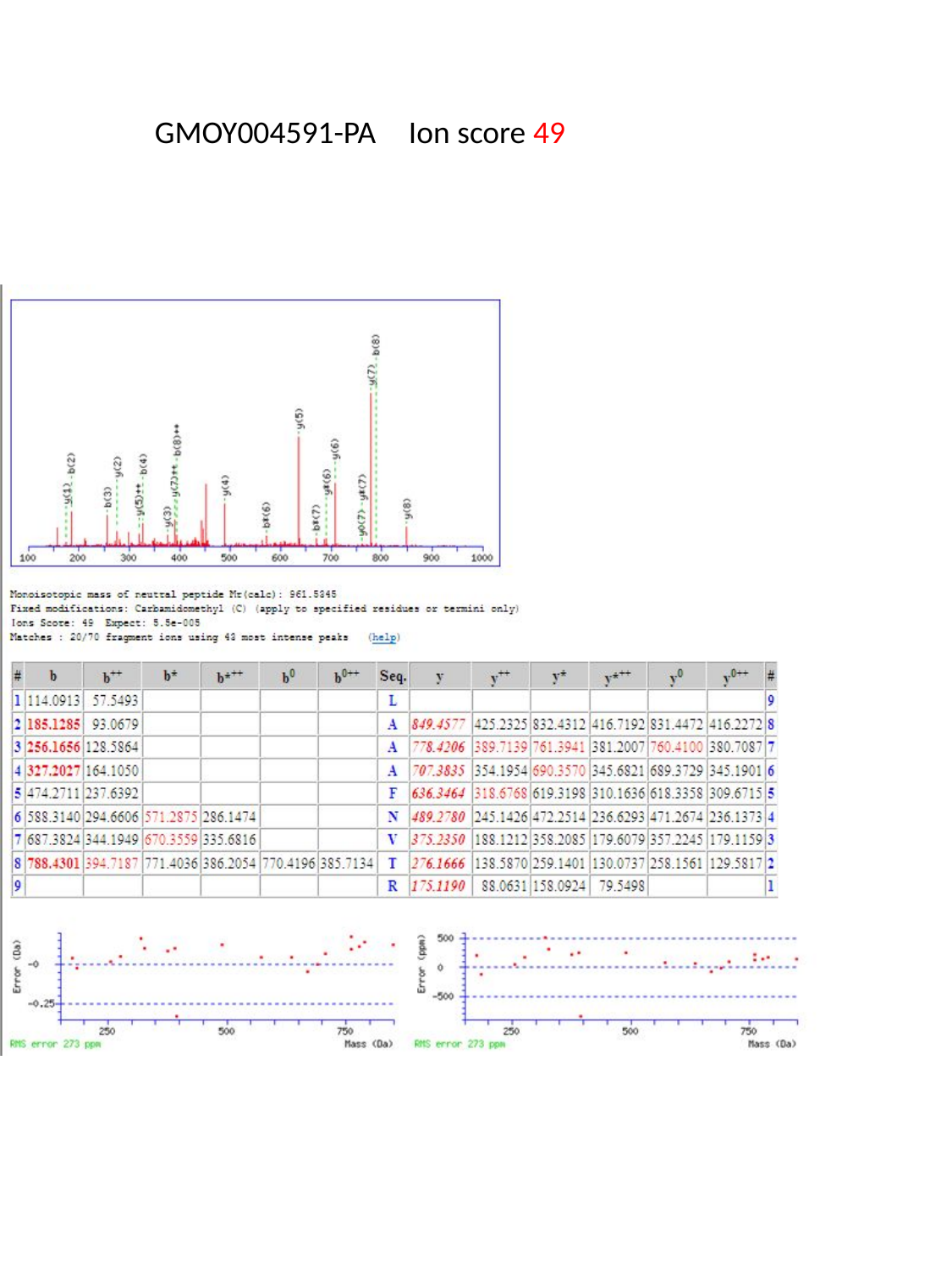

GMOY004591-PA 	Ion score 49

## Slide 74
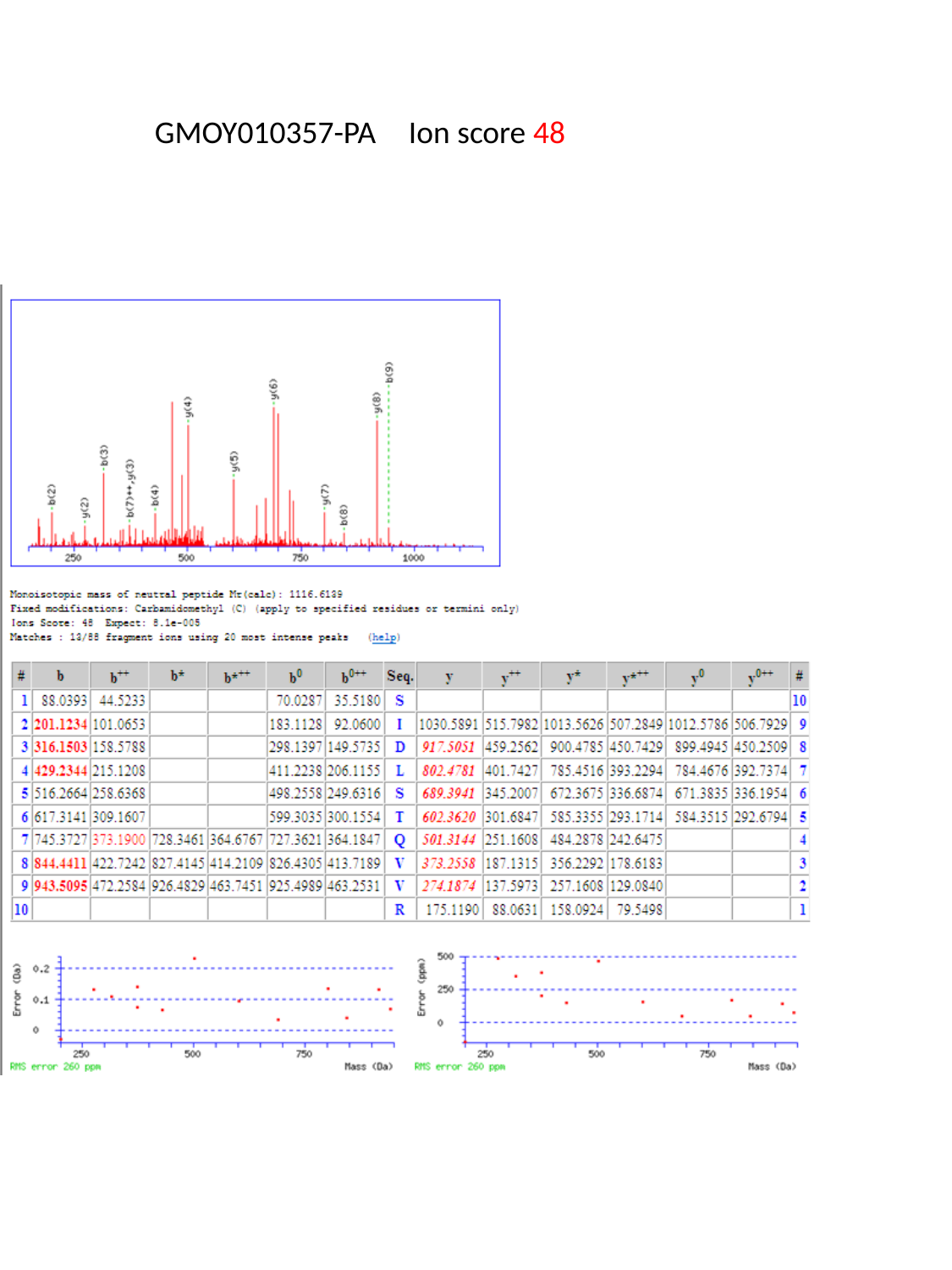

GMOY010357-PA 	Ion score 48

## Slide 75
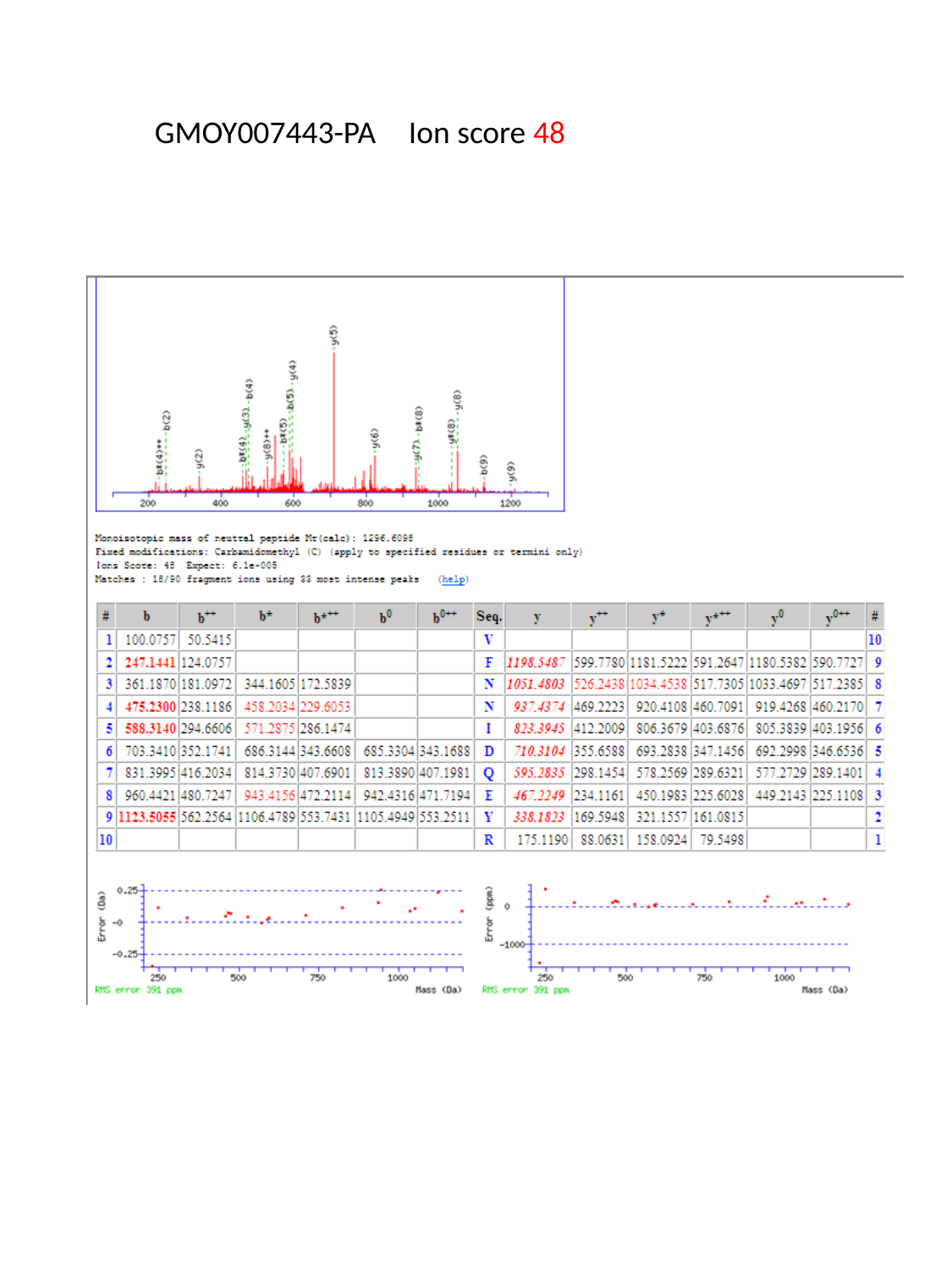

GMOY007443-PA 	Ion score 48

## Slide 76
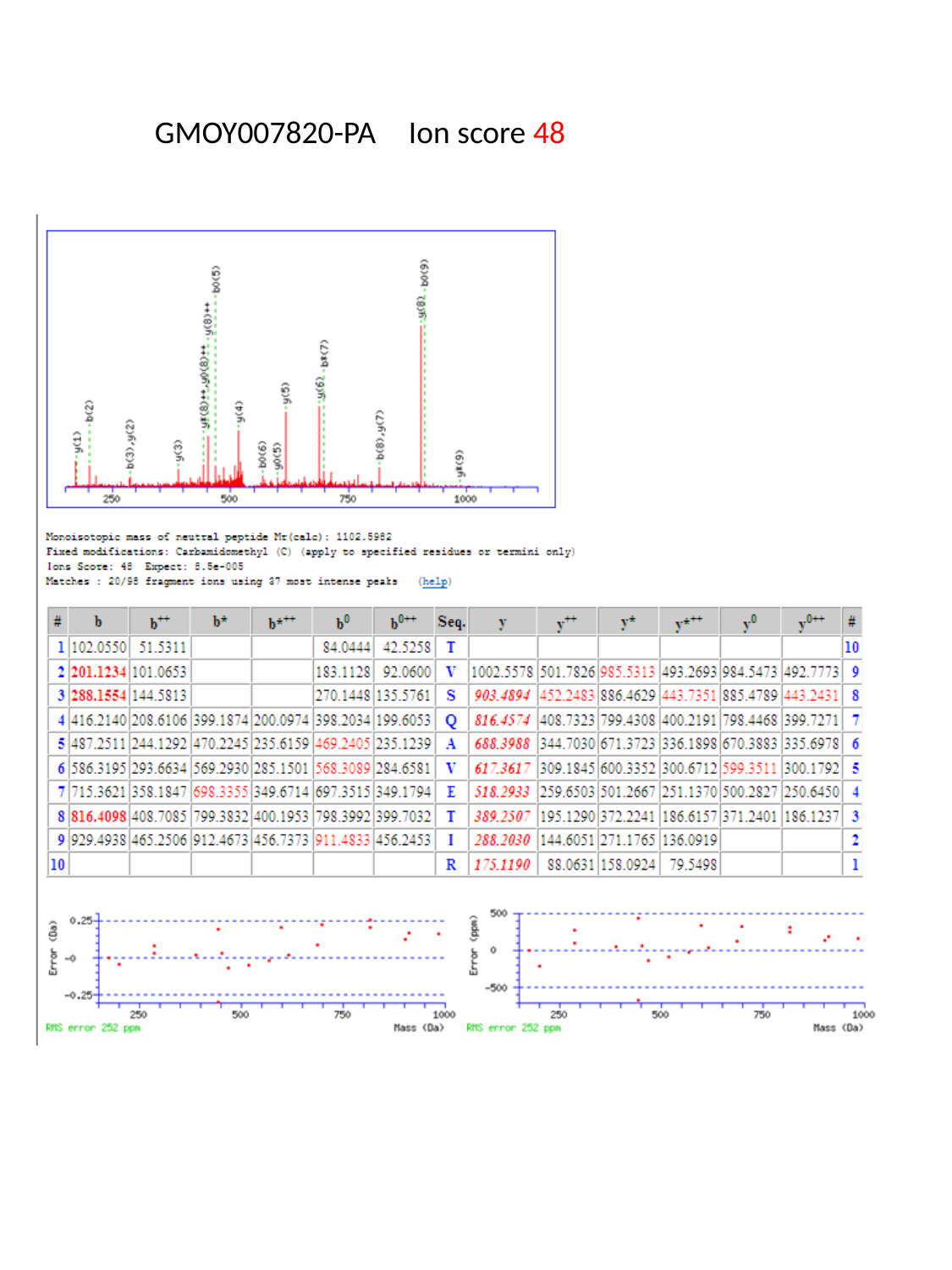

GMOY007820-PA 	Ion score 48

## Slide 77
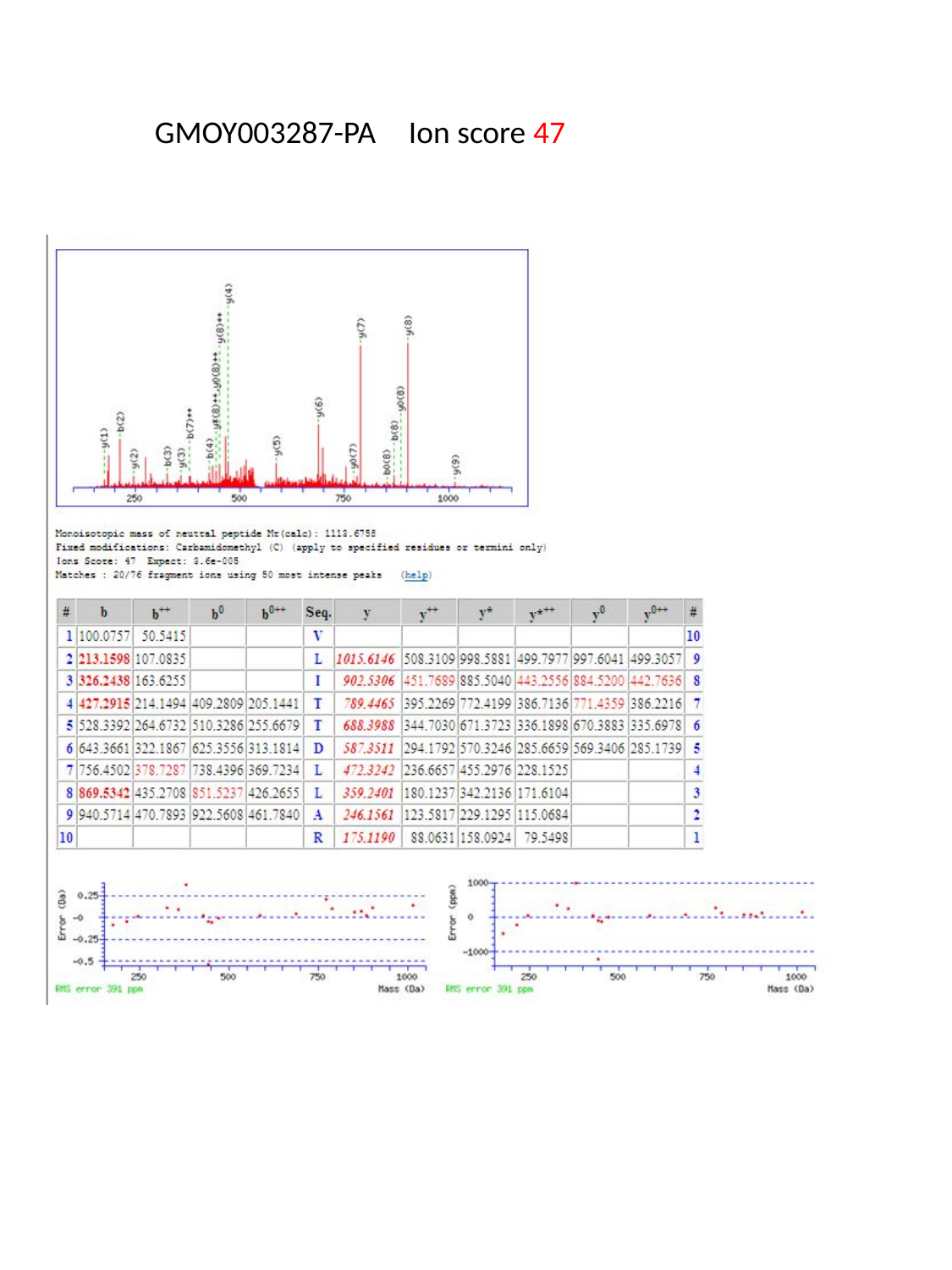

GMOY003287-PA 	Ion score 47

## Slide 78
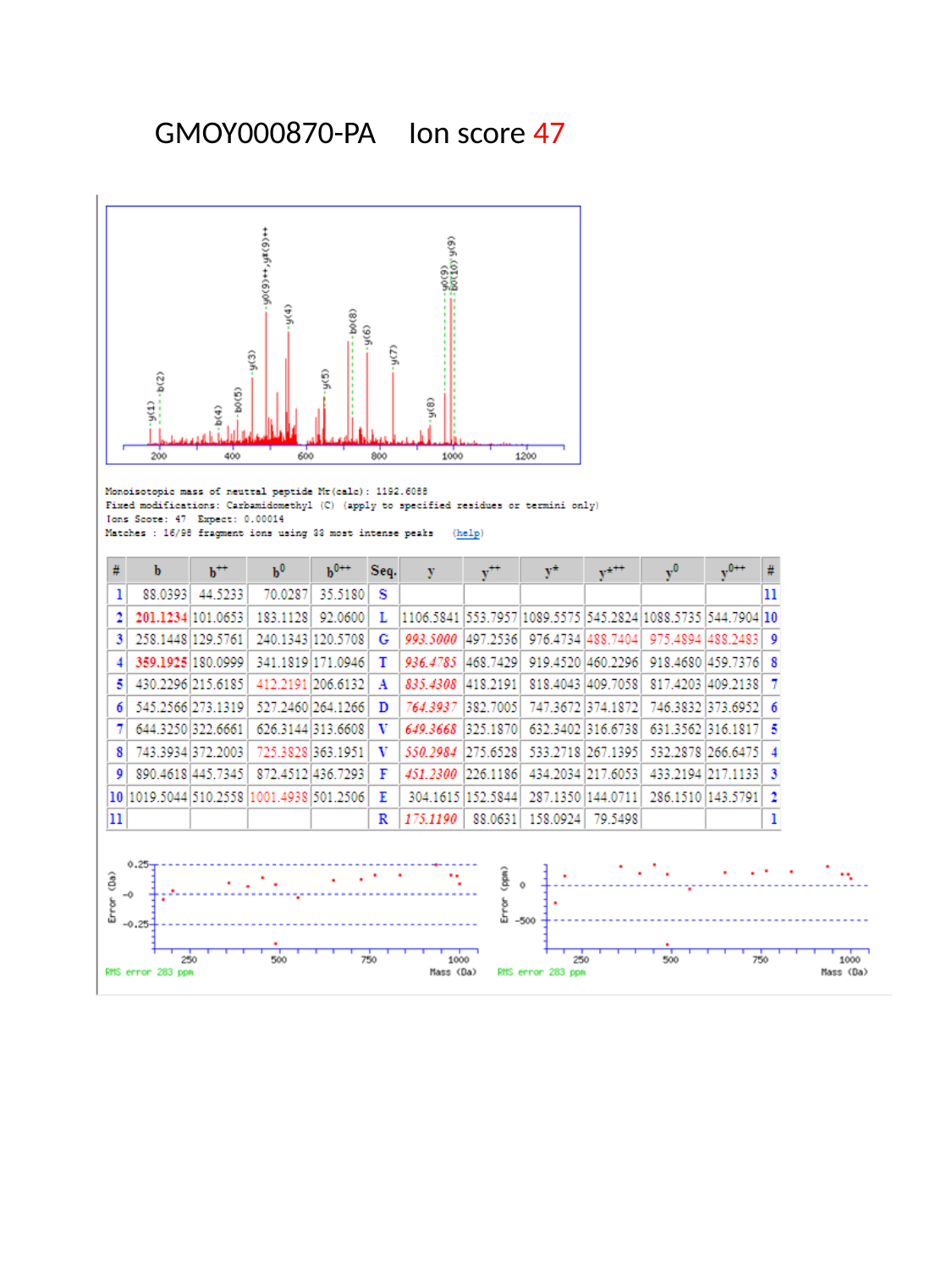

GMOY000870-PA 	Ion score 47

## Slide 79
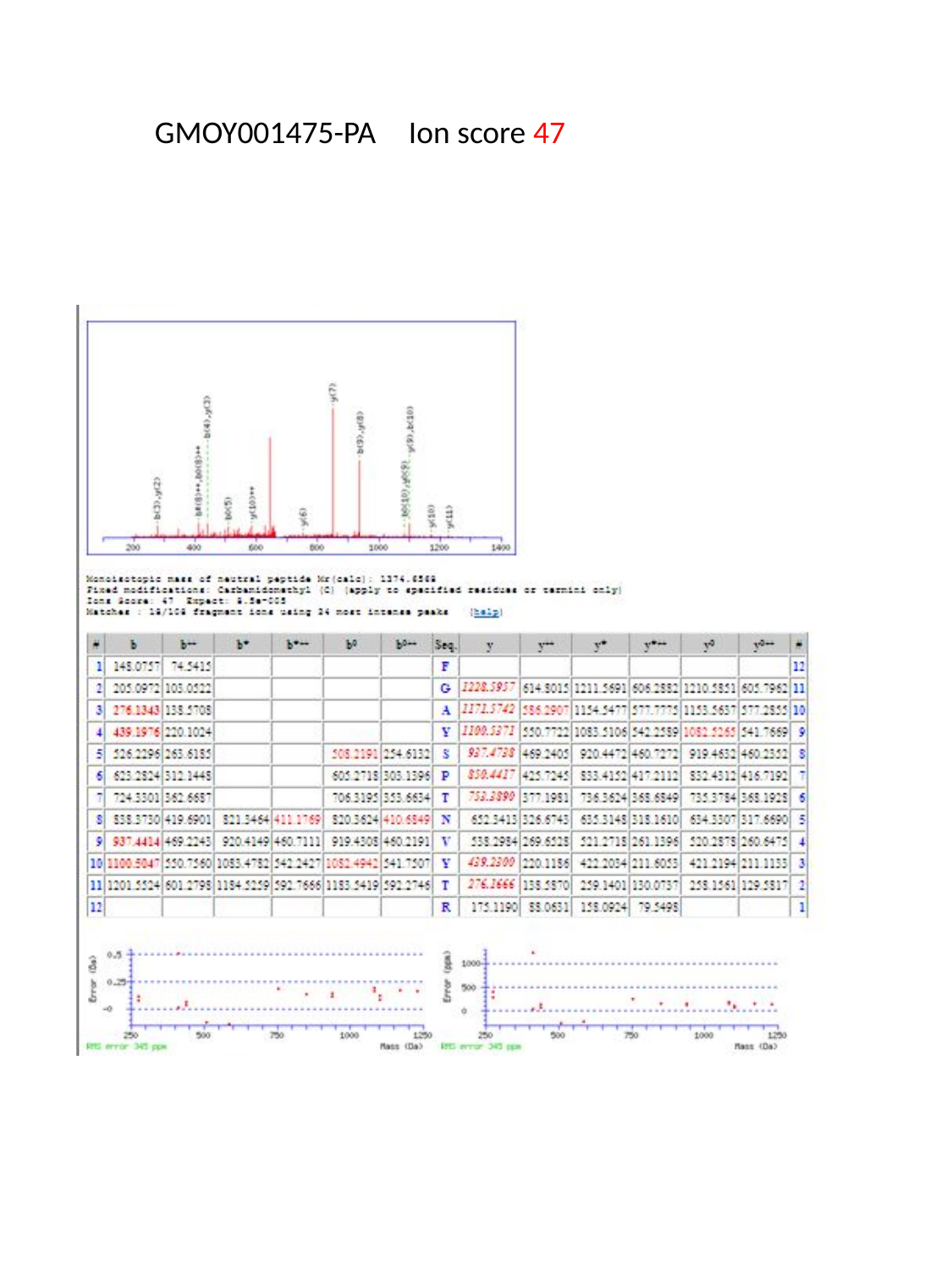

GMOY001475-PA 	Ion score 47

## Slide 80
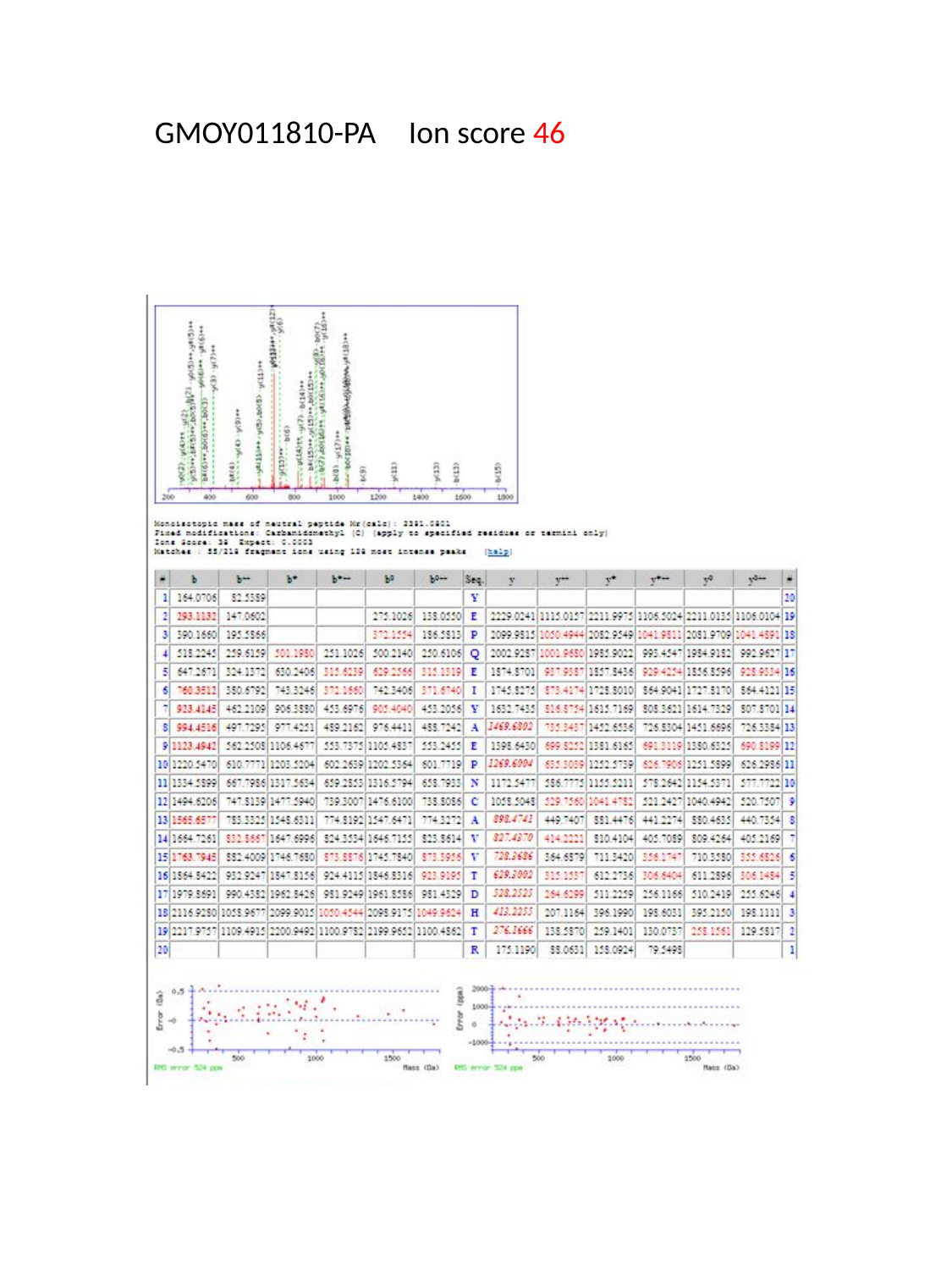

GMOY011810-PA 	Ion score 46

## Slide 81
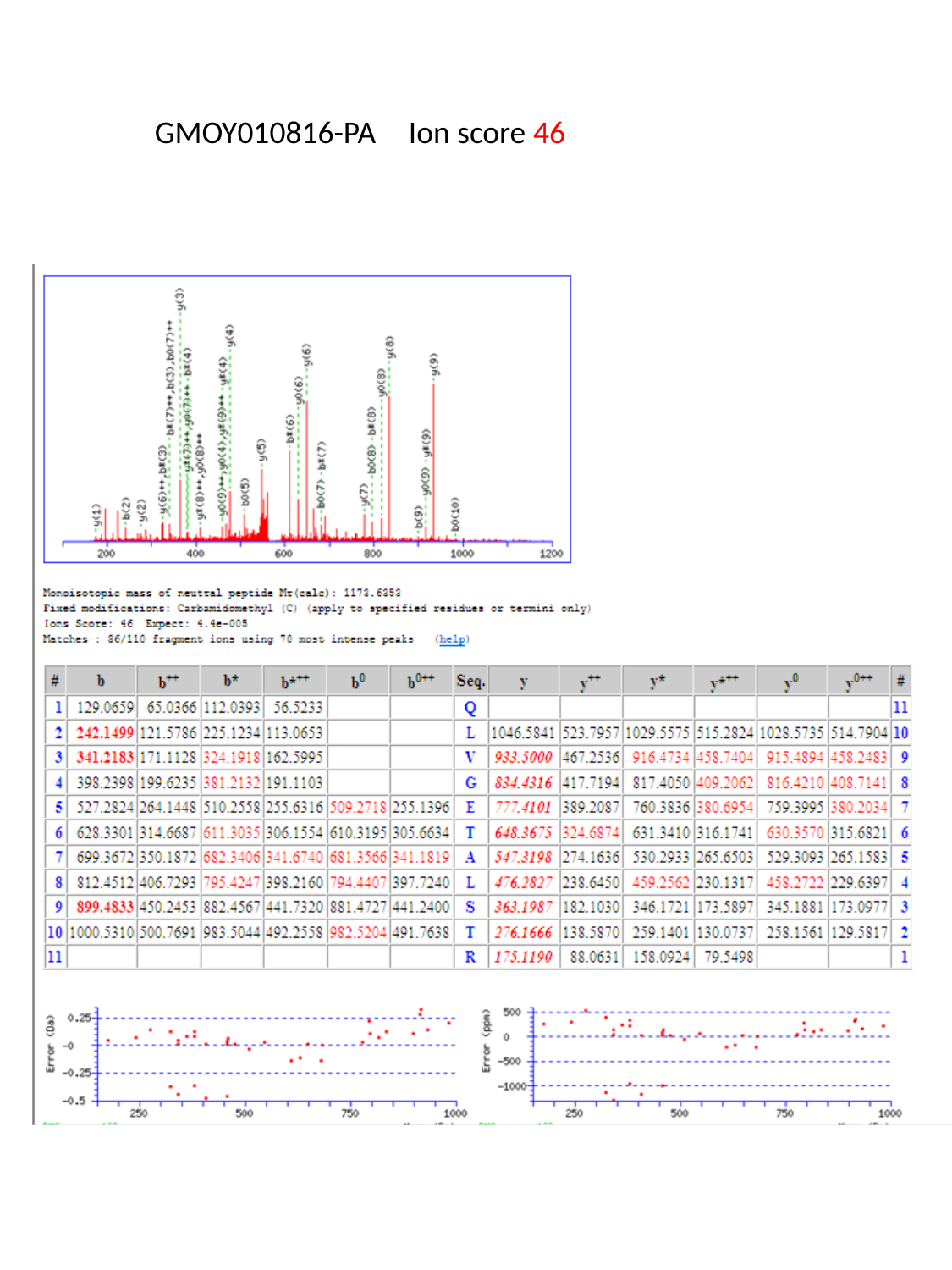

GMOY010816-PA 	Ion score 46

## Slide 82
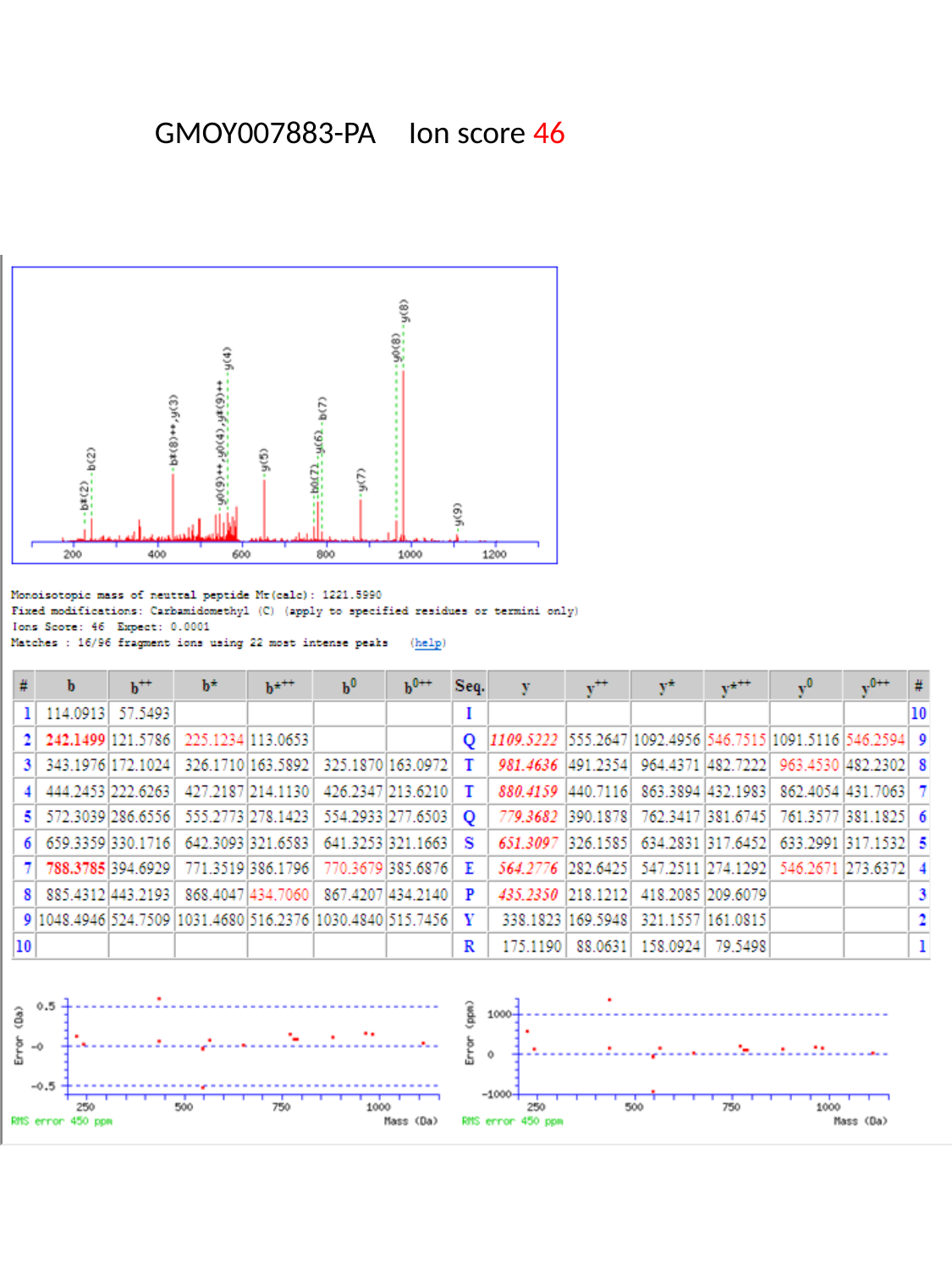

GMOY007883-PA 	Ion score 46

## Slide 83
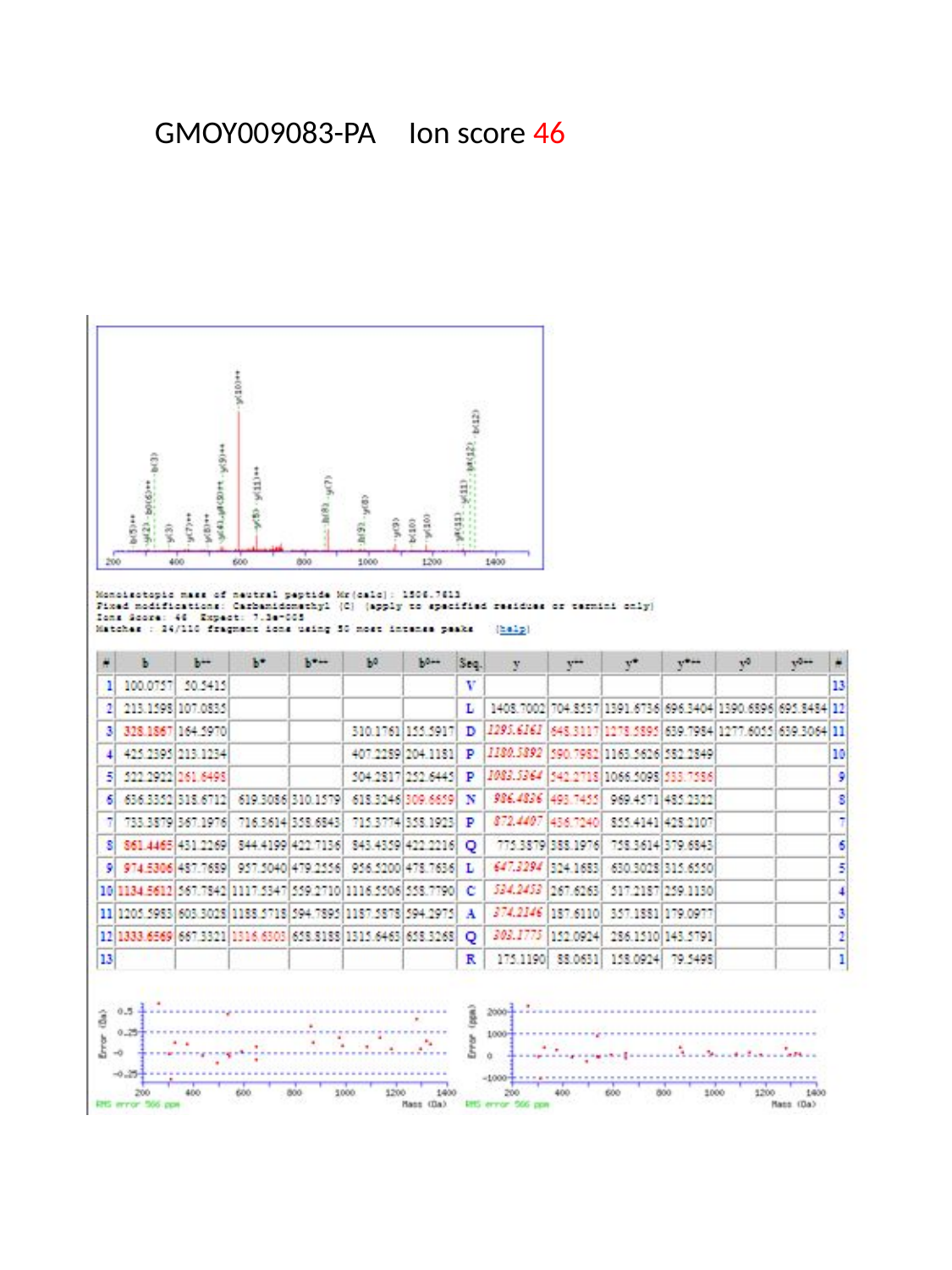

GMOY009083-PA 	Ion score 46

## Slide 84
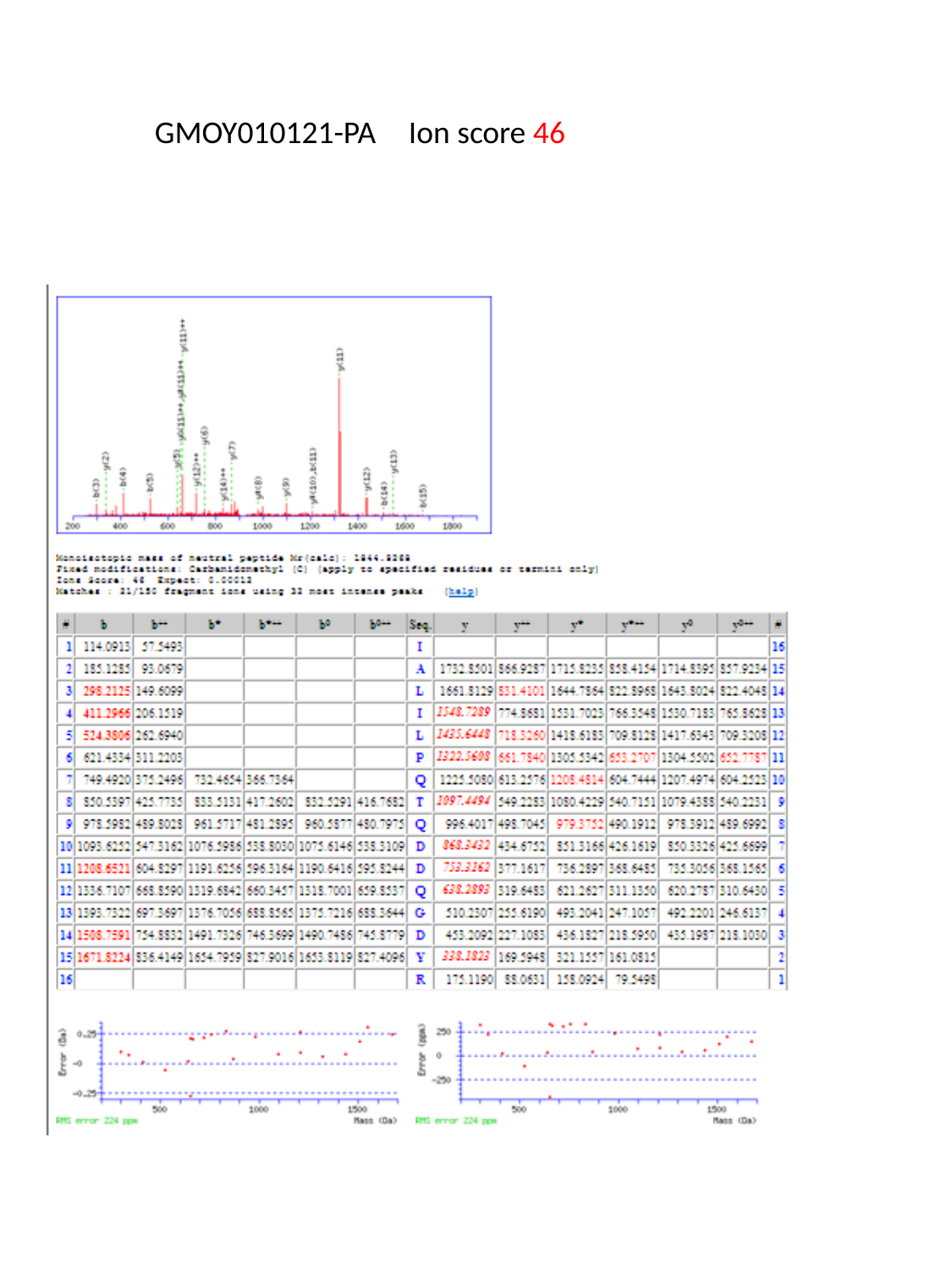

GMOY010121-PA 	Ion score 46

## Slide 85
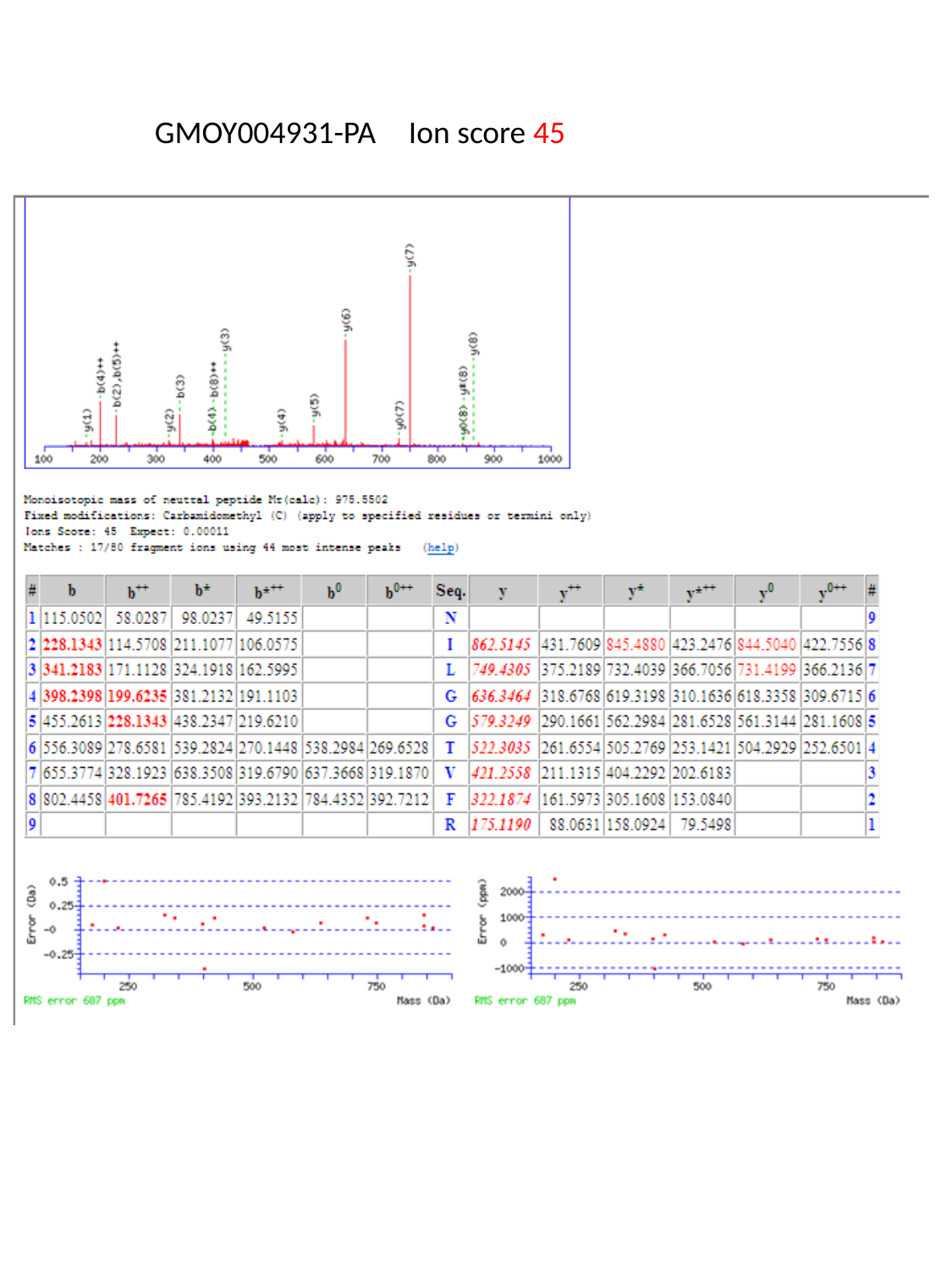

GMOY004931-PA 	Ion score 45

## Slide 86
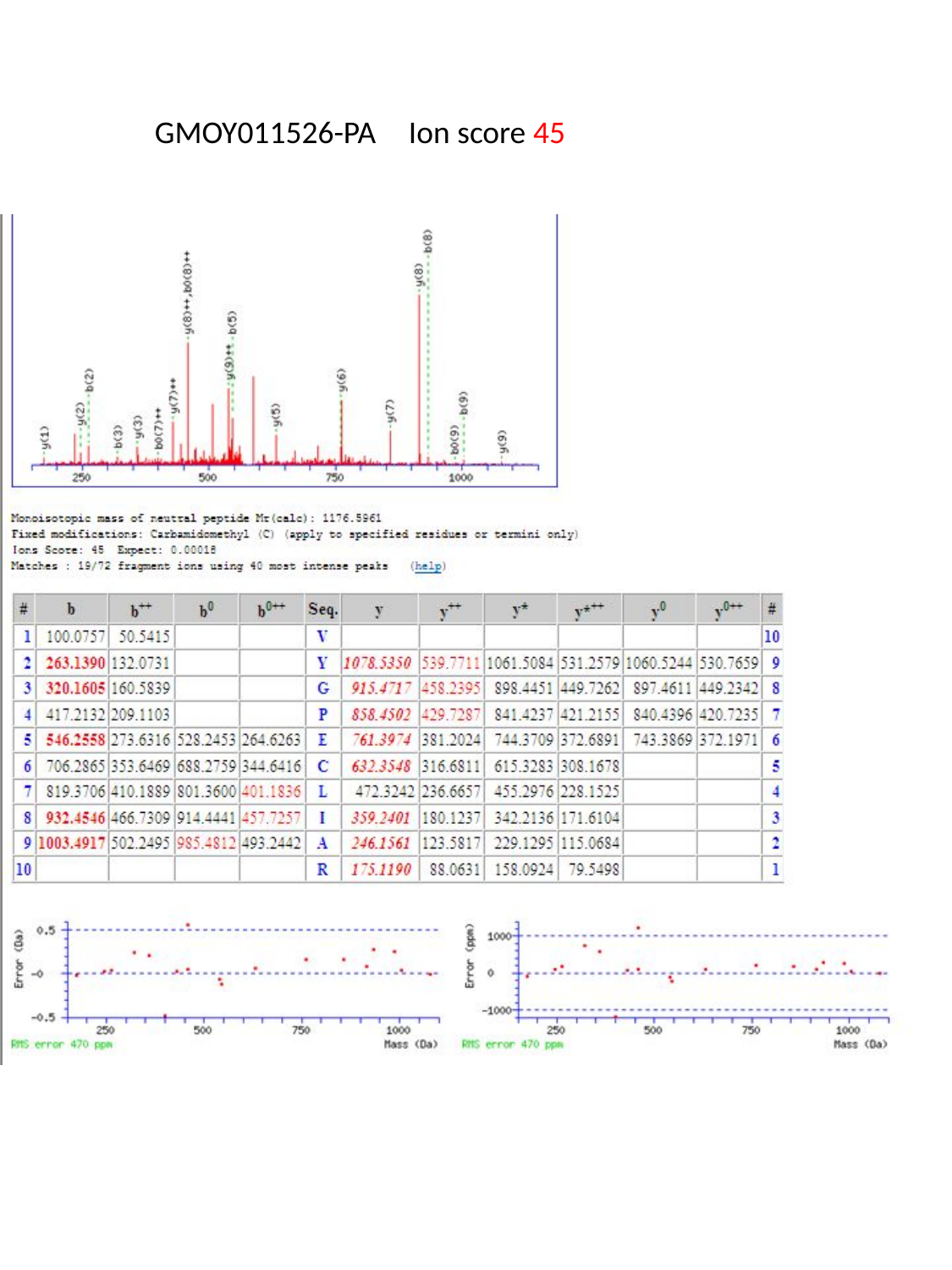

GMOY011526-PA 	Ion score 45

## Slide 87
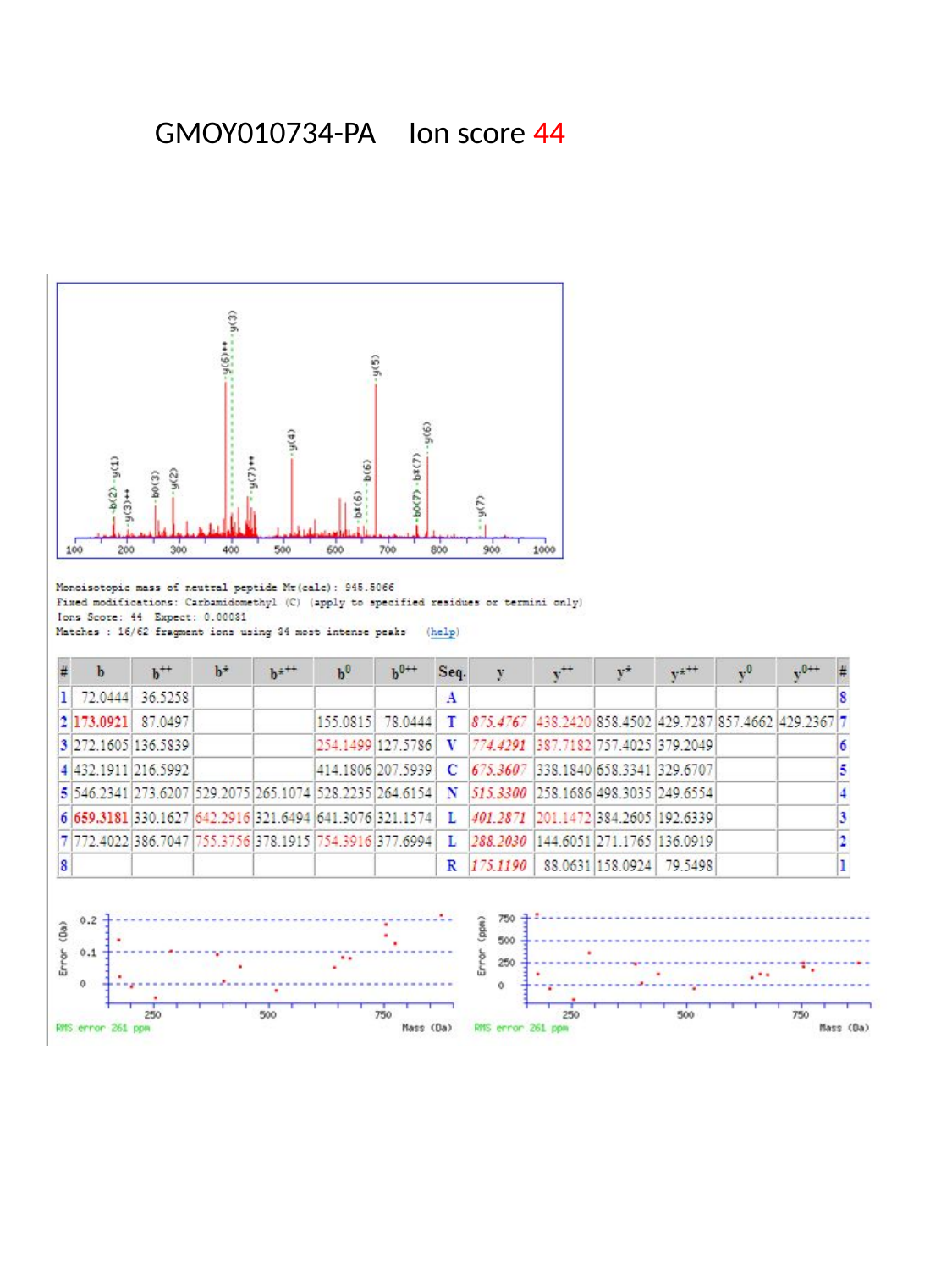

GMOY010734-PA	Ion score 44

## Slide 88
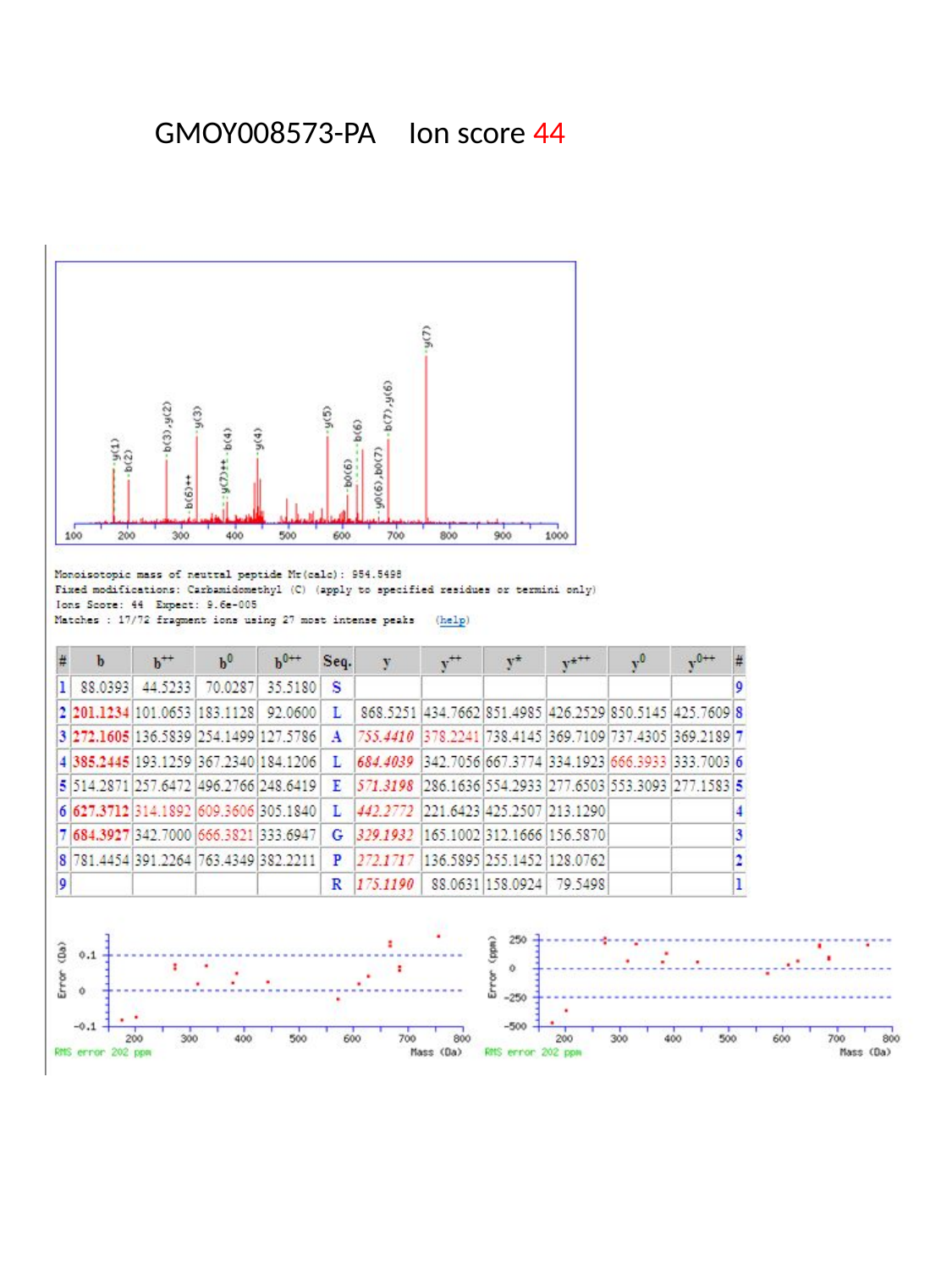

GMOY008573-PA	Ion score 44

## Slide 89
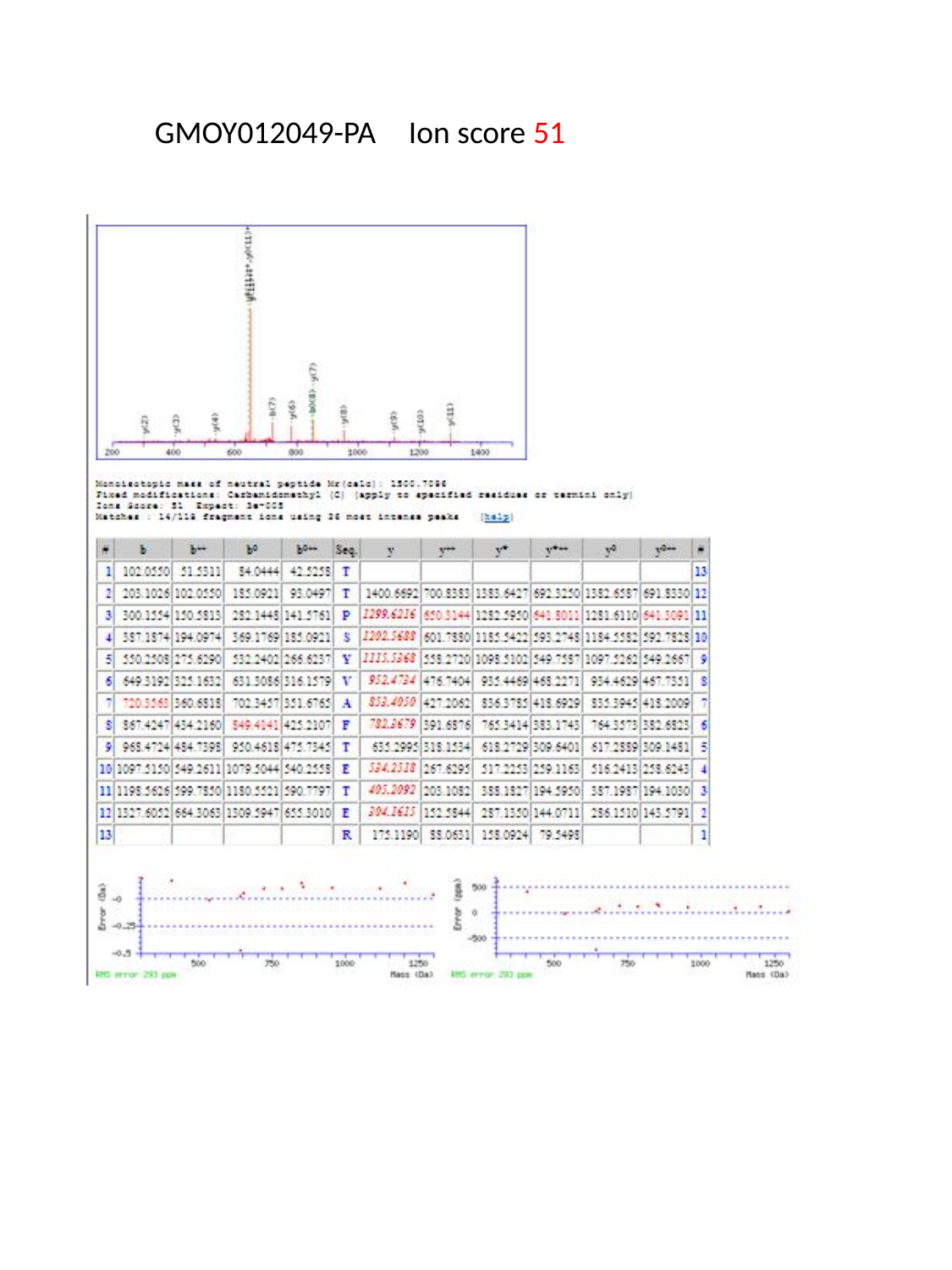

GMOY012049-PA	Ion score 51

## Slide 90
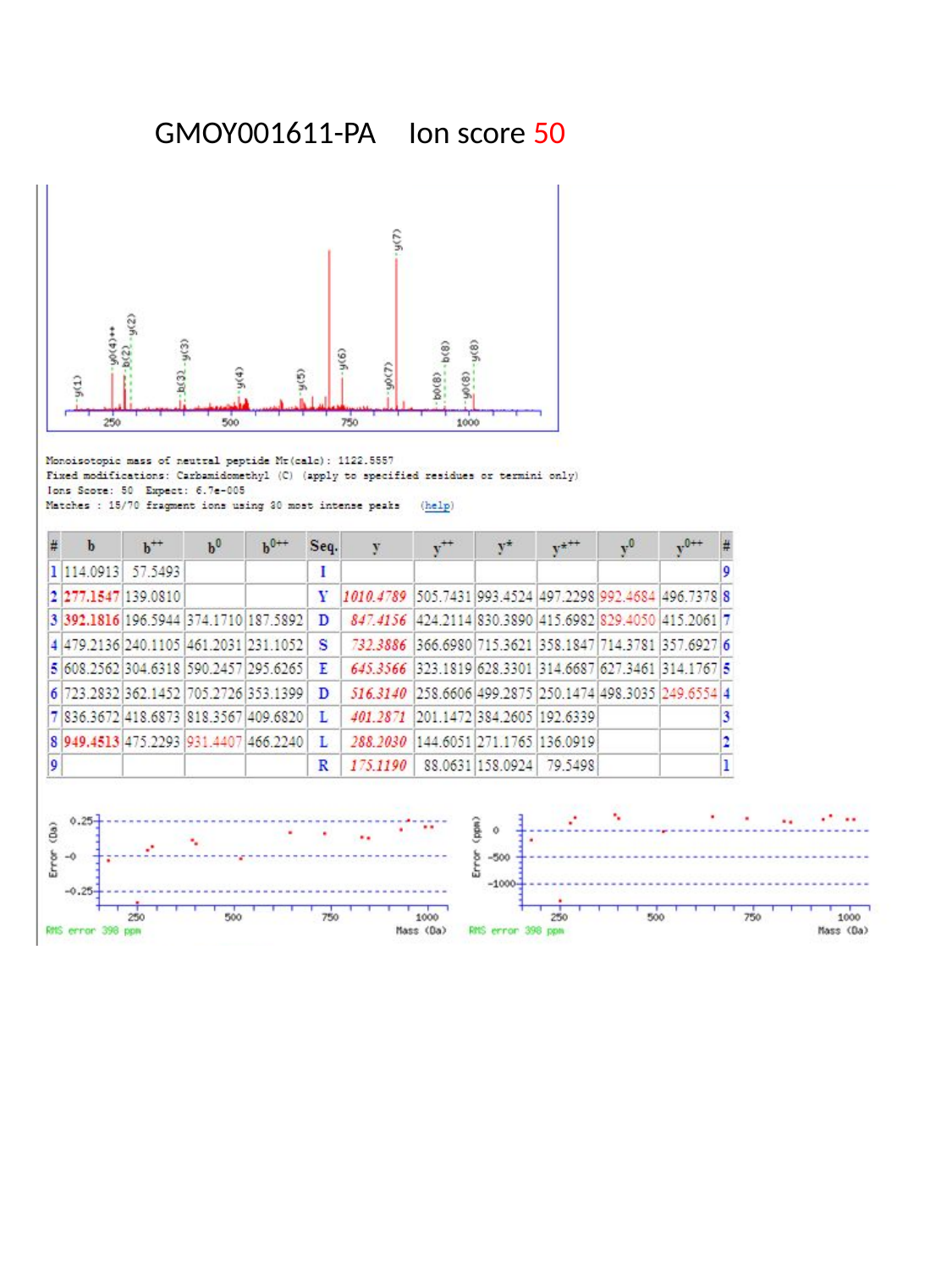

GMOY001611-PA 	Ion score 50

## Slide 91
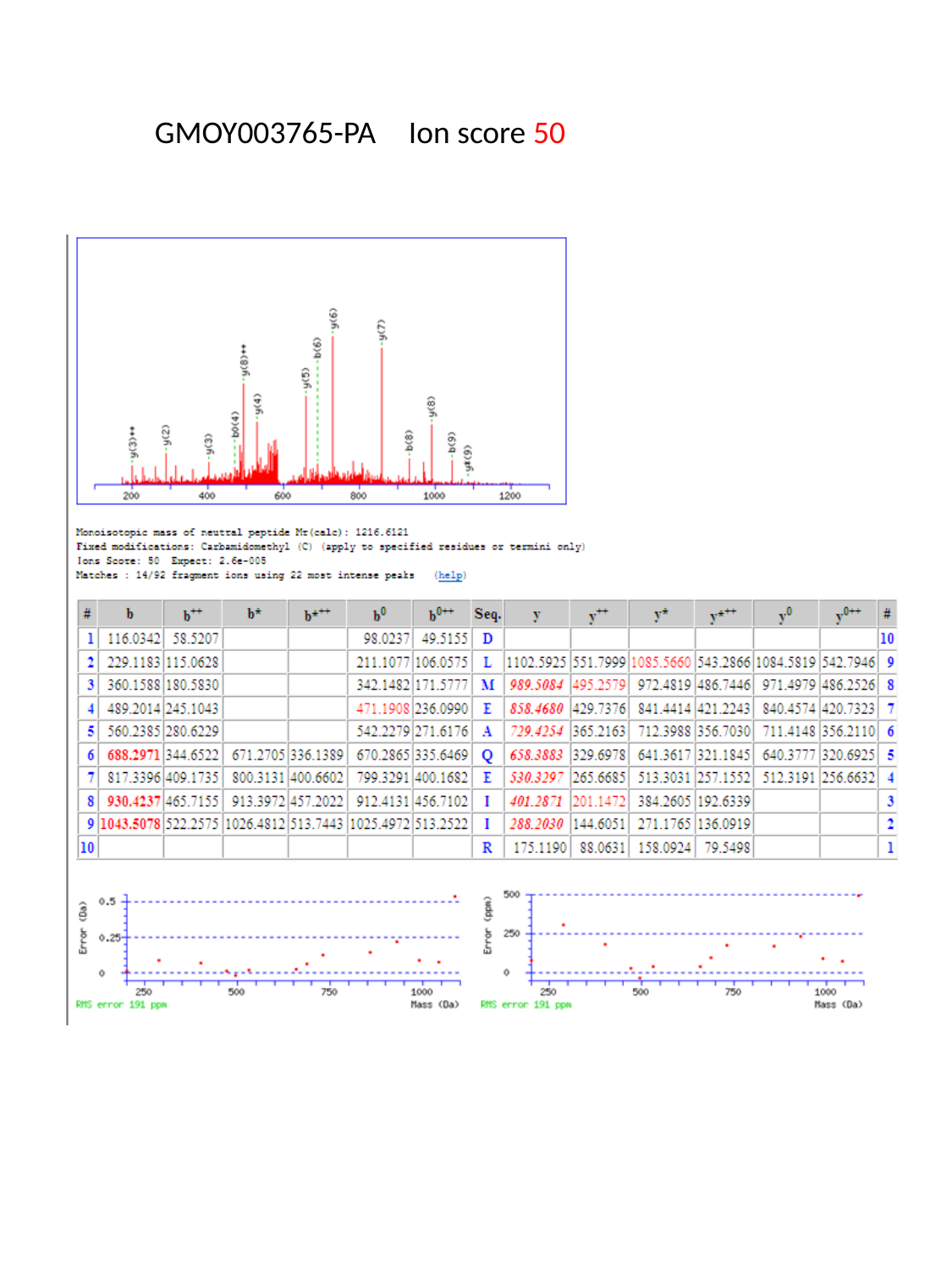

GMOY003765-PA 	Ion score 50

## Slide 92
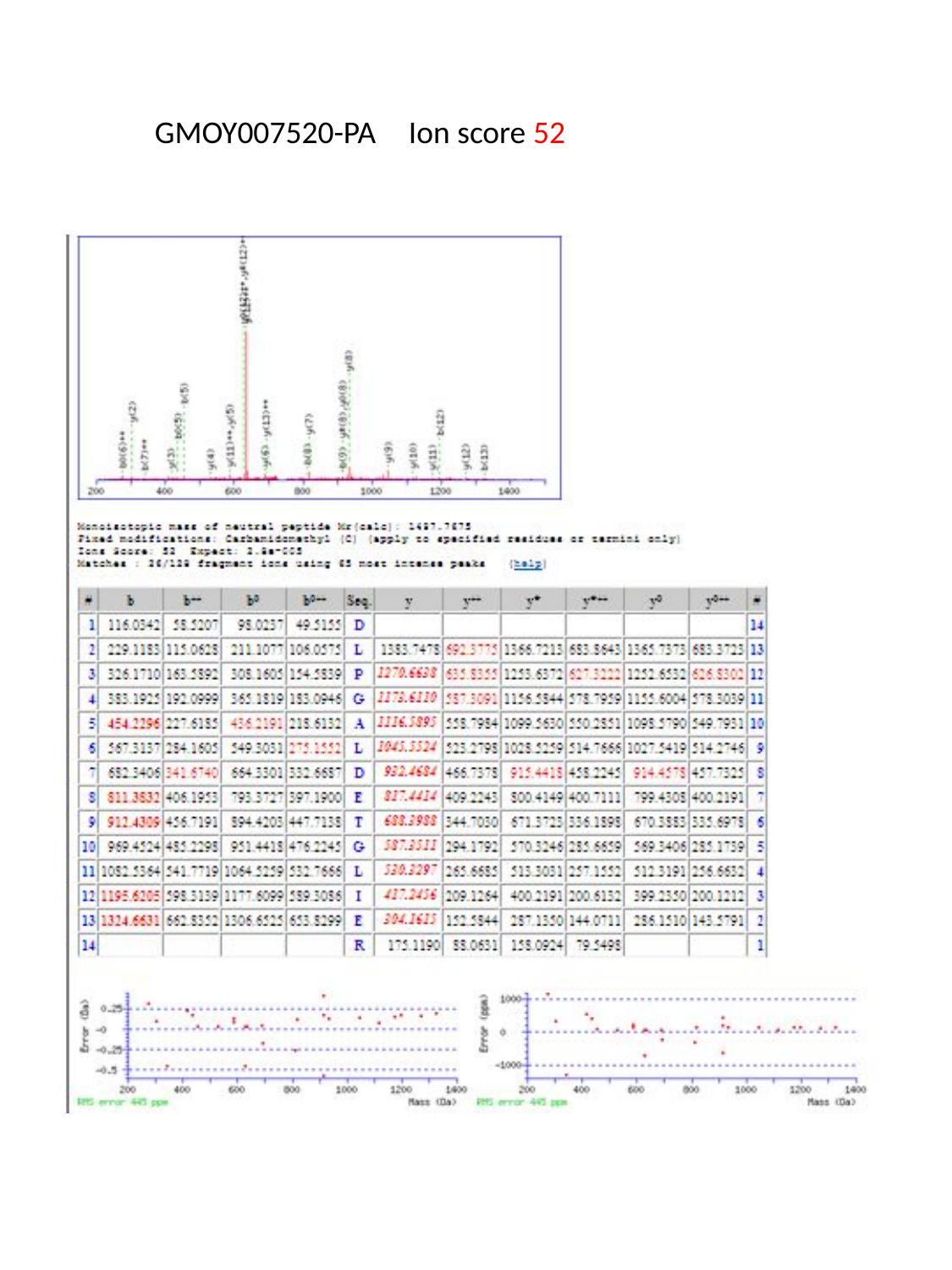

GMOY007520-PA 	Ion score 52

## Slide 93
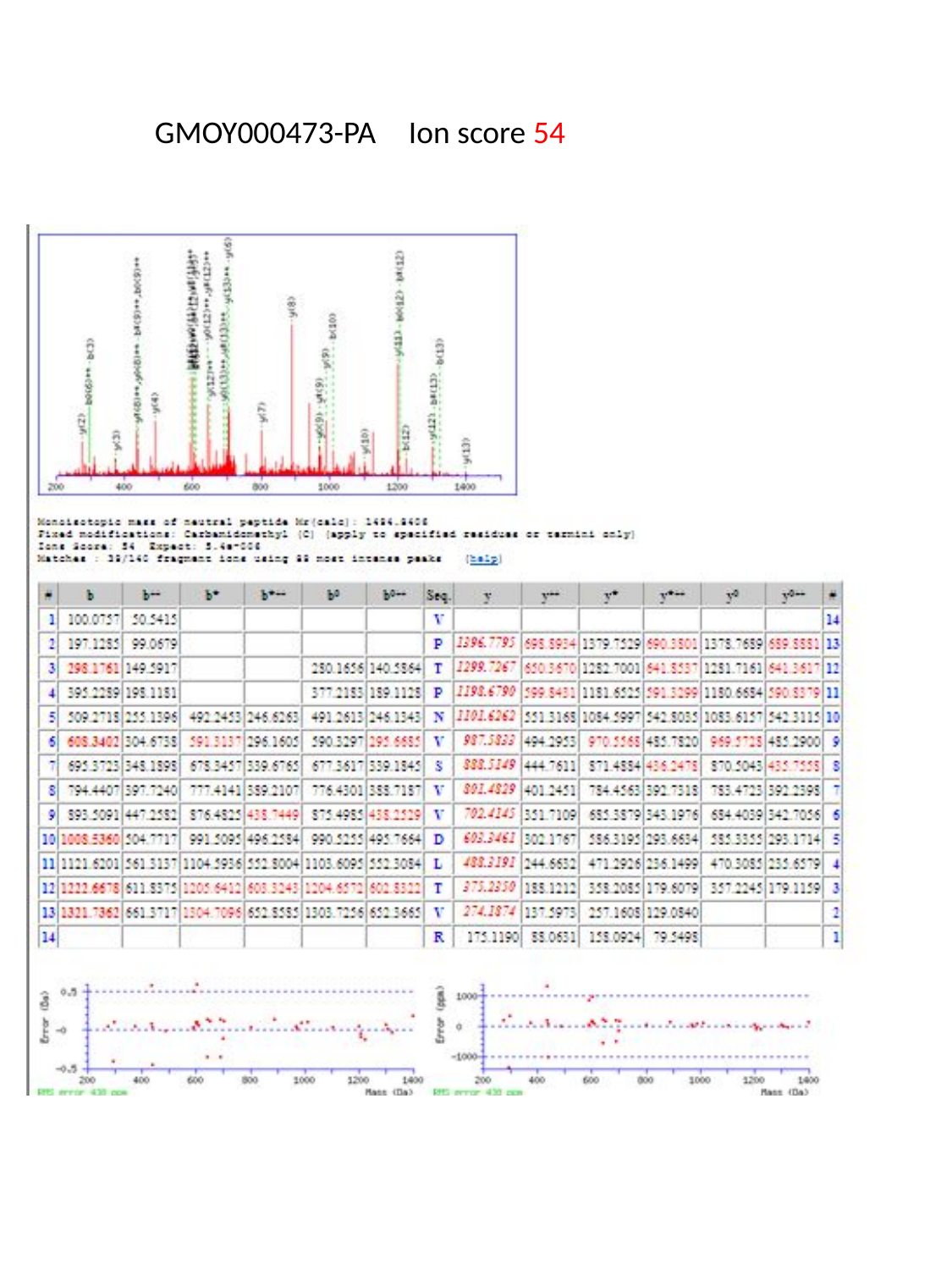

GMOY000473-PA 	Ion score 54

## Slide 94
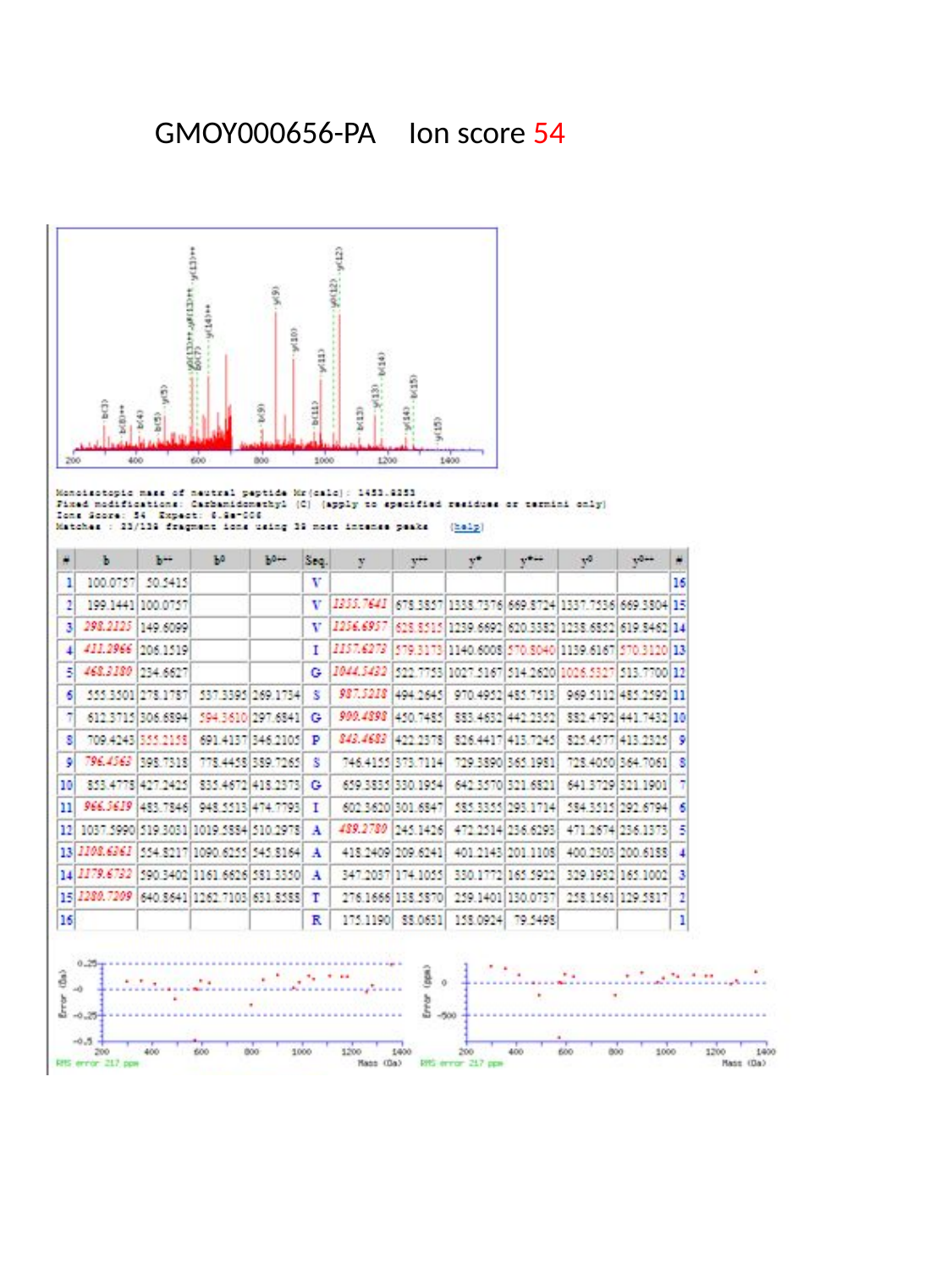

GMOY000656-PA 	Ion score 54

## Slide 95
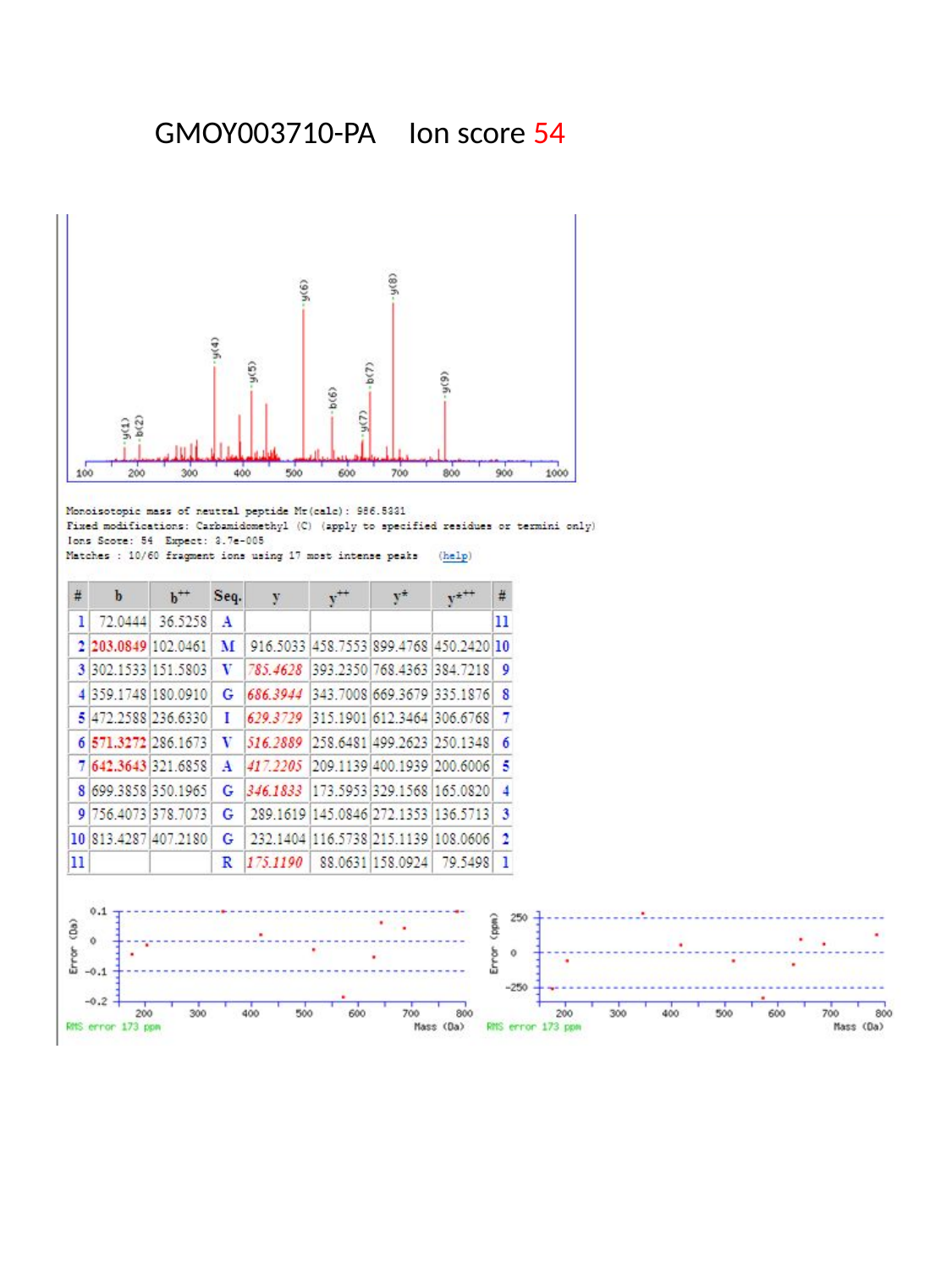

GMOY003710-PA 	Ion score 54

## Slide 96
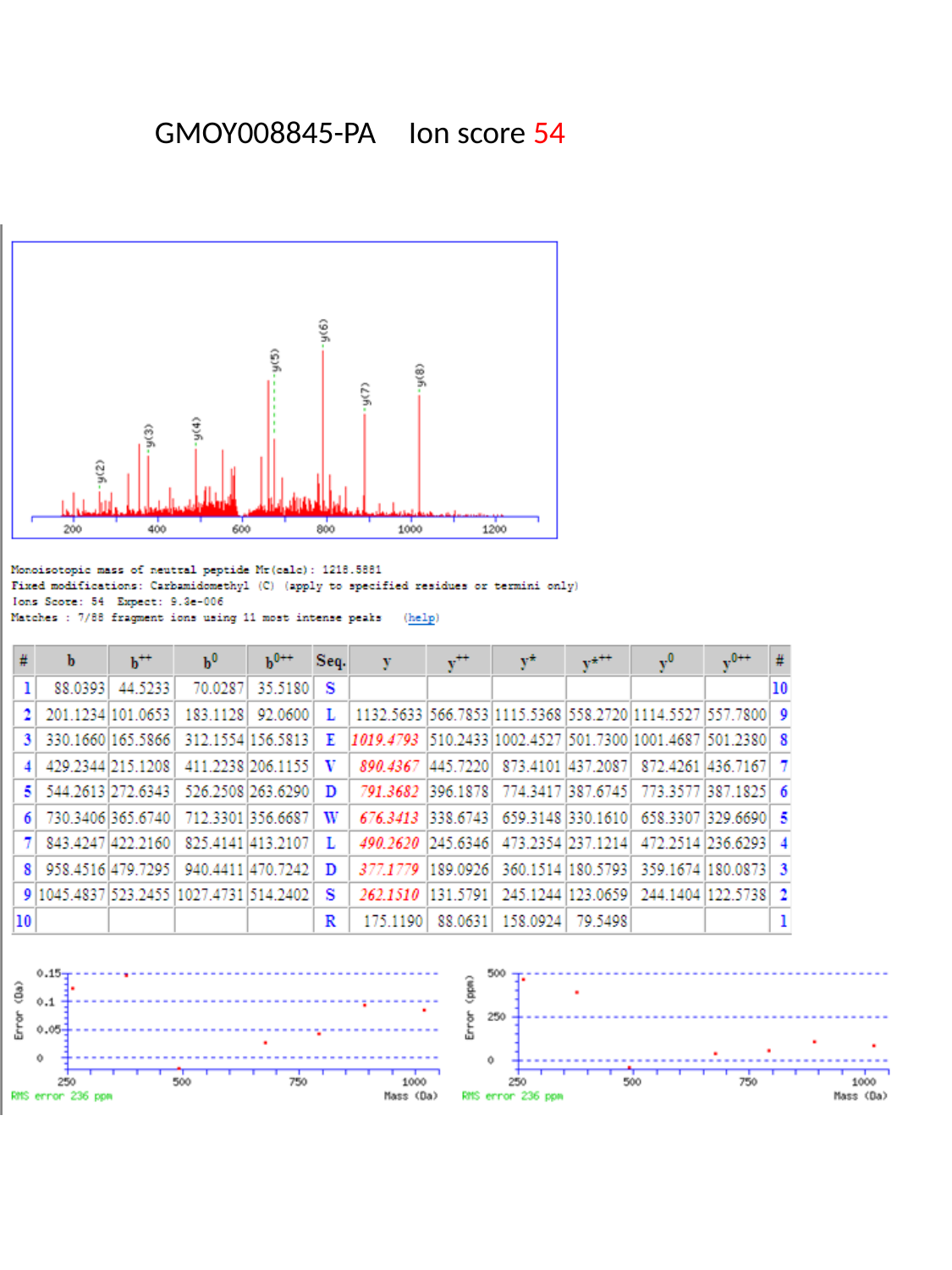

GMOY008845-PA 	Ion score 54

## Slide 97
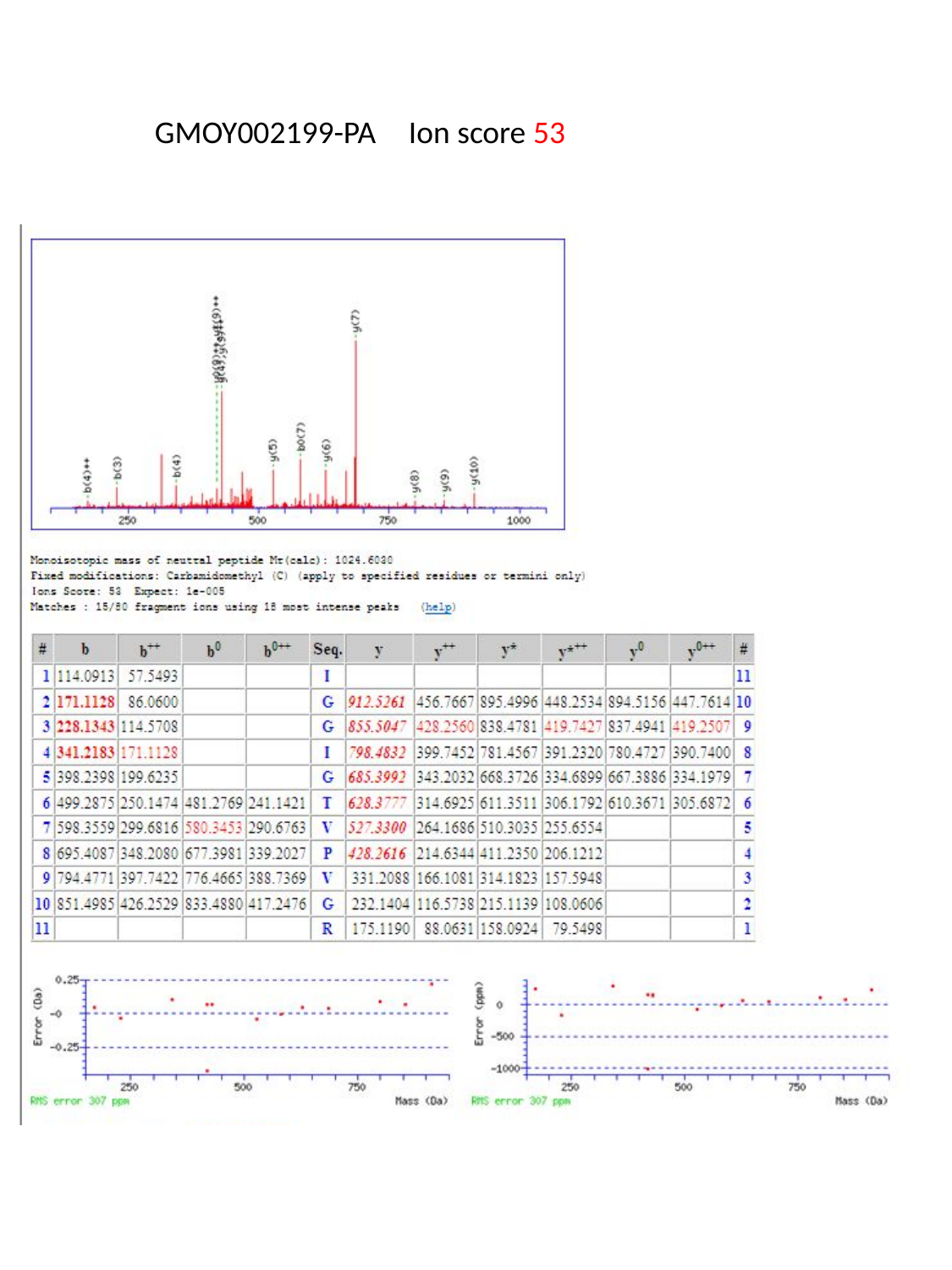

GMOY002199-PA	Ion score 53

## Slide 98
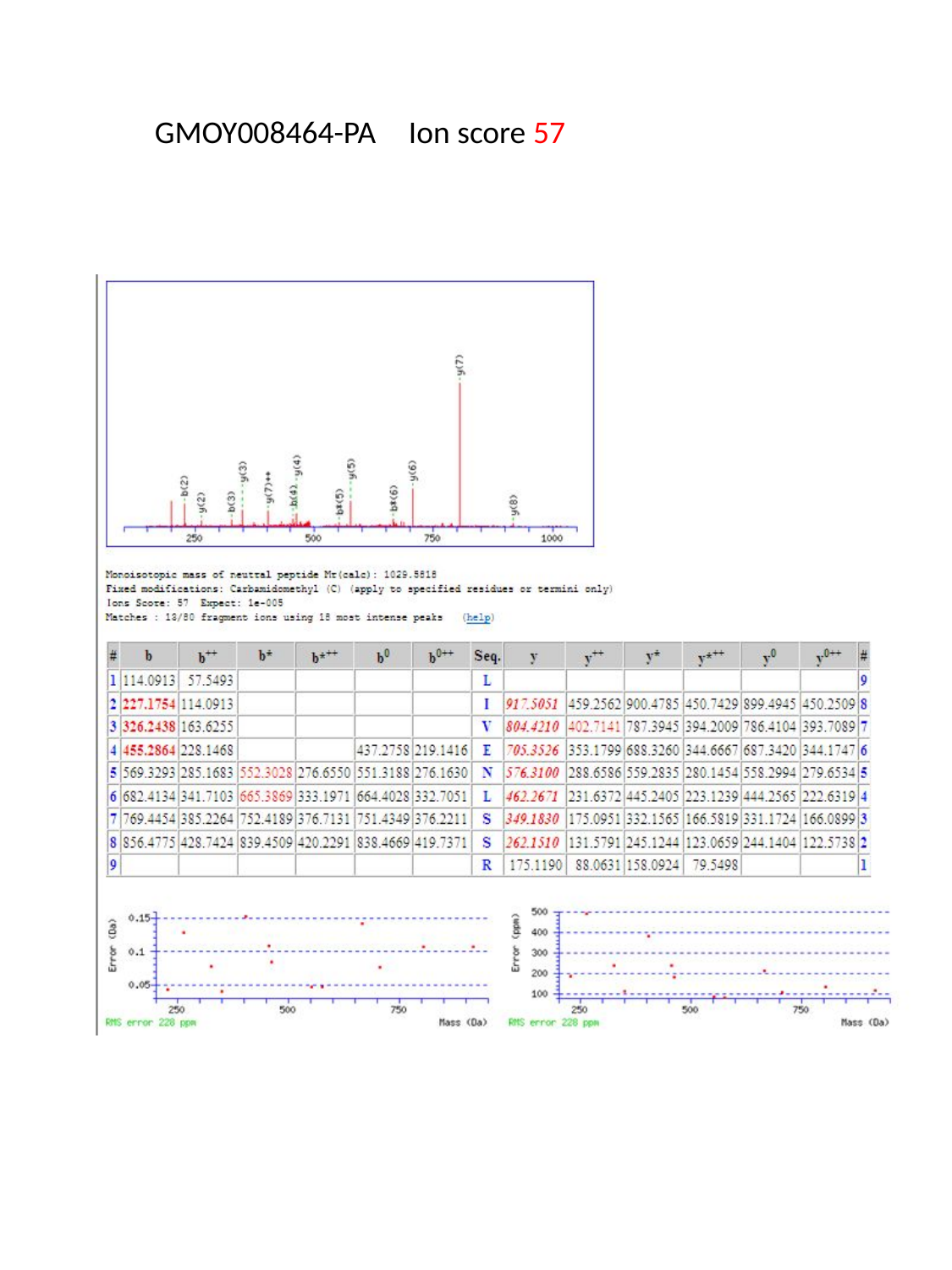

GMOY008464-PA	Ion score 57

## Slide 99
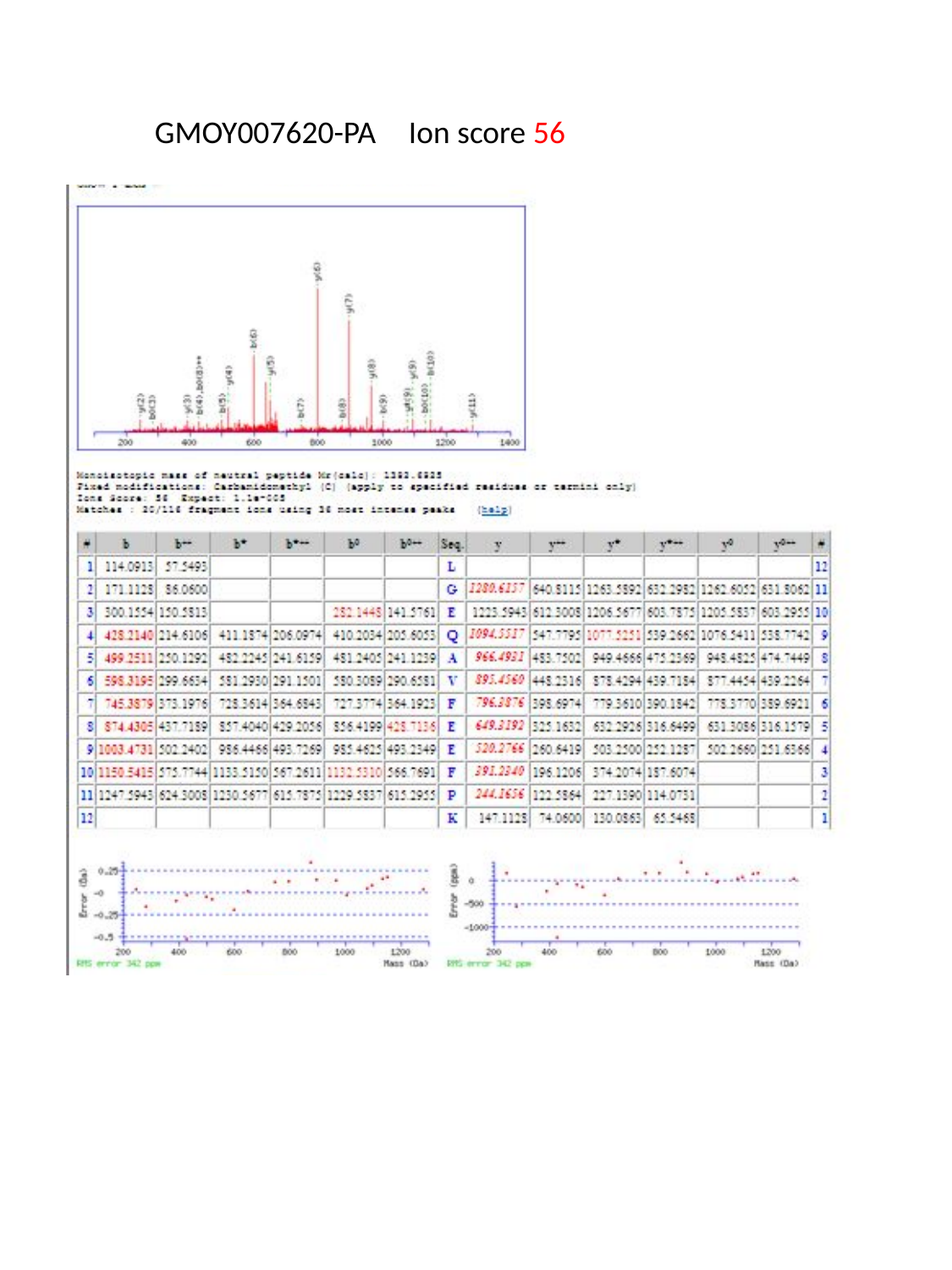

GMOY007620-PA	Ion score 56

## Slide 100
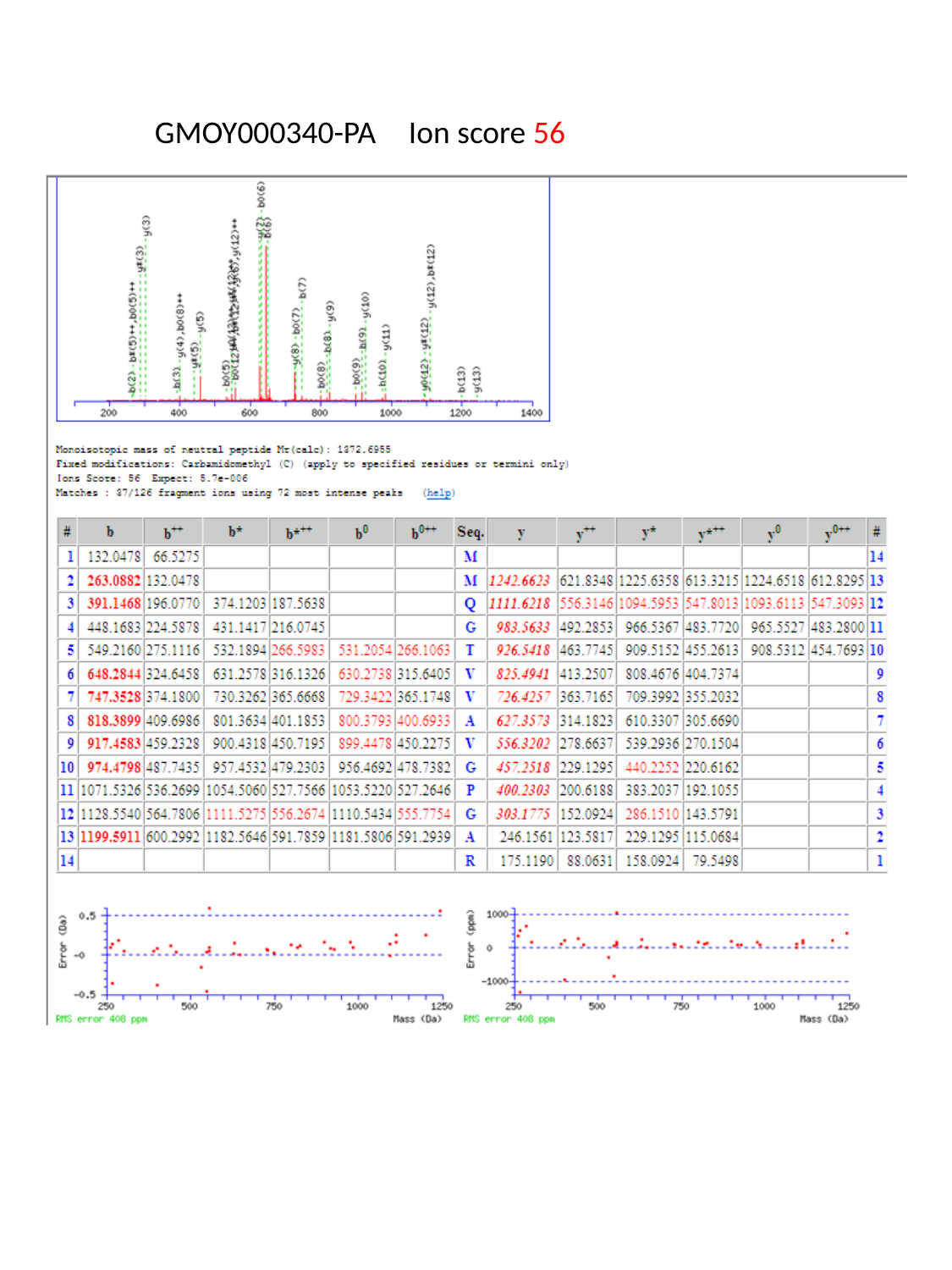

GMOY000340-PA 	Ion score 56

## Slide 101
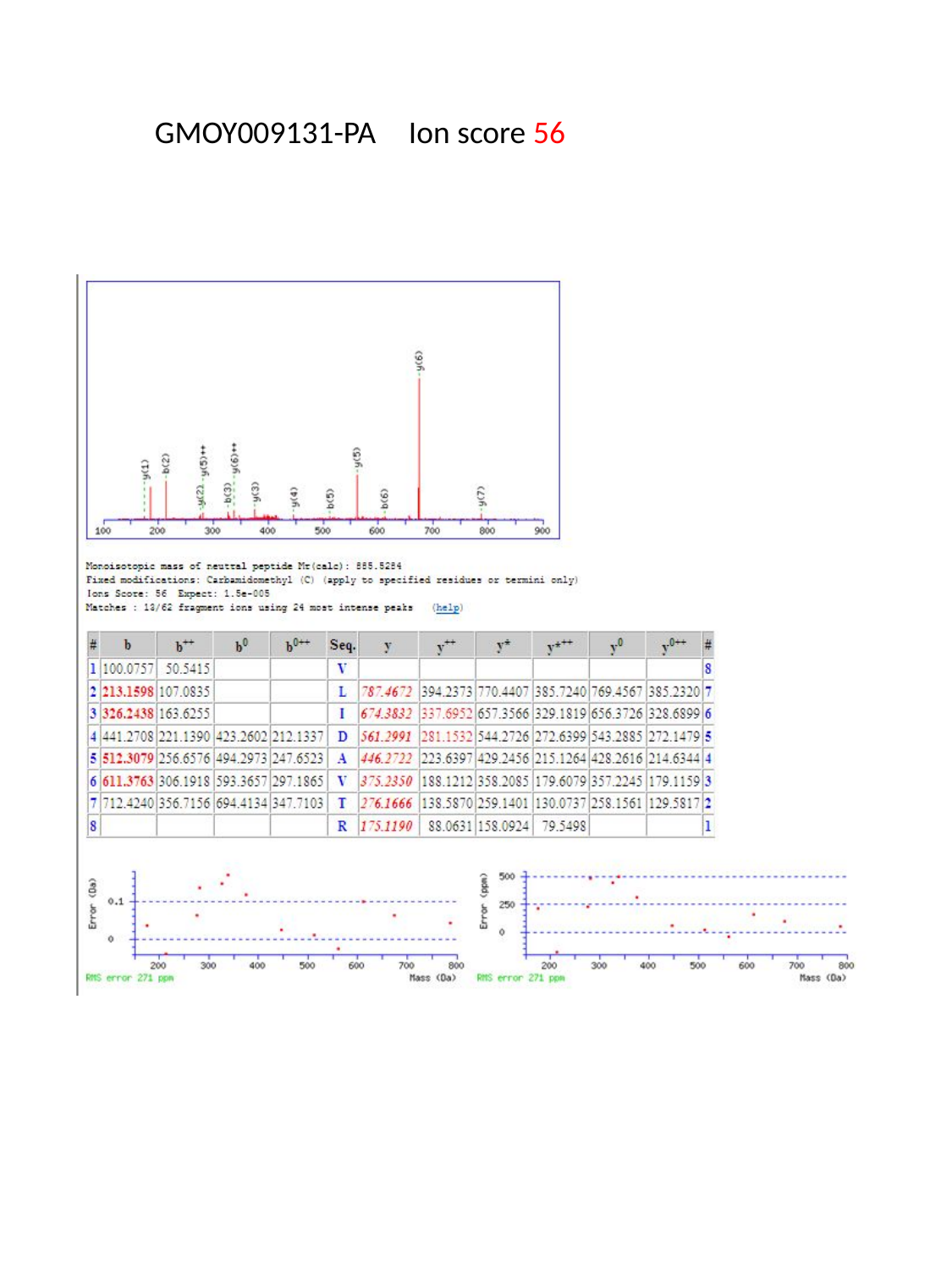

GMOY009131-PA 	Ion score 56

## Slide 102
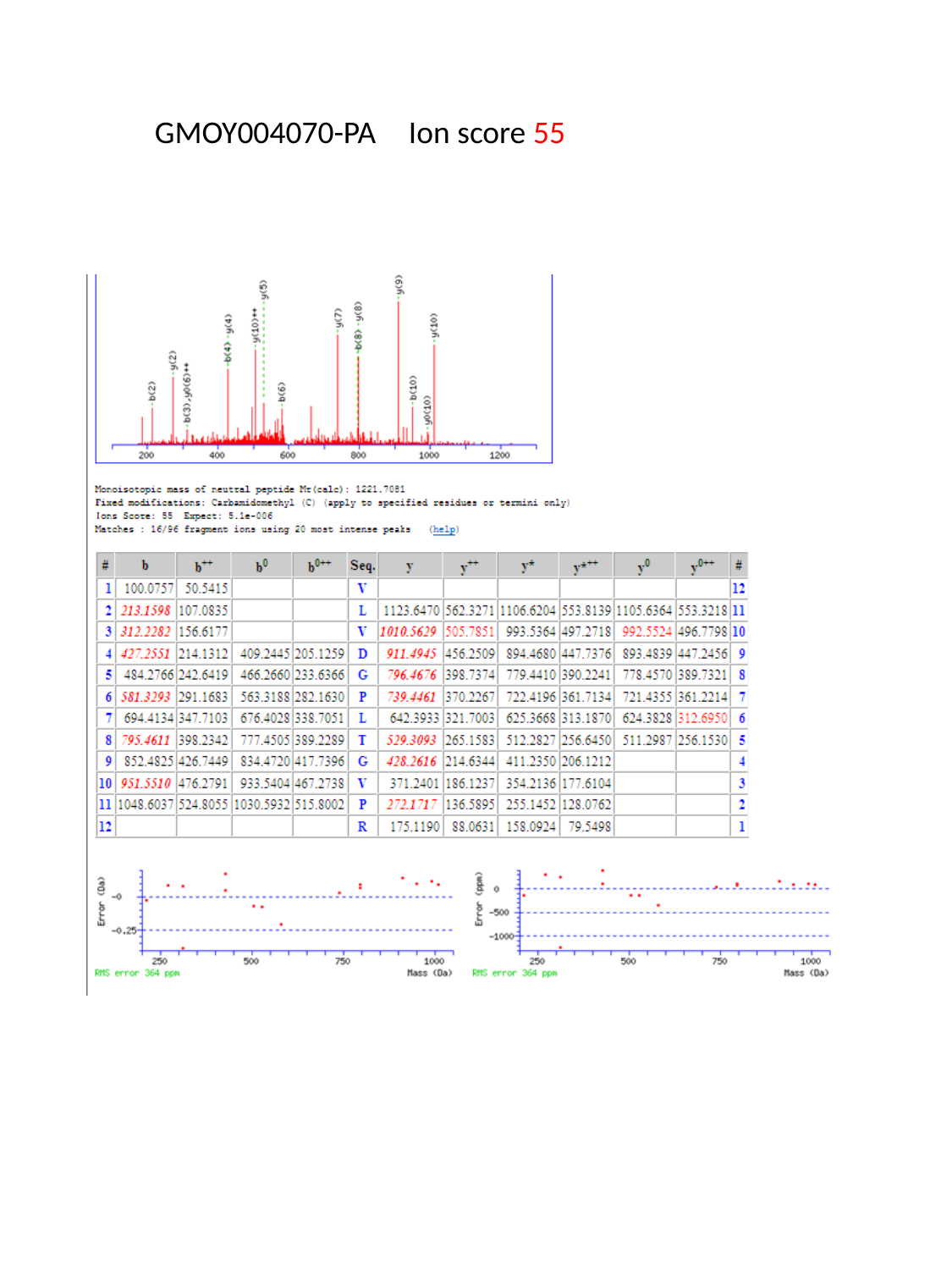

GMOY004070-PA 	Ion score 55

## Slide 103
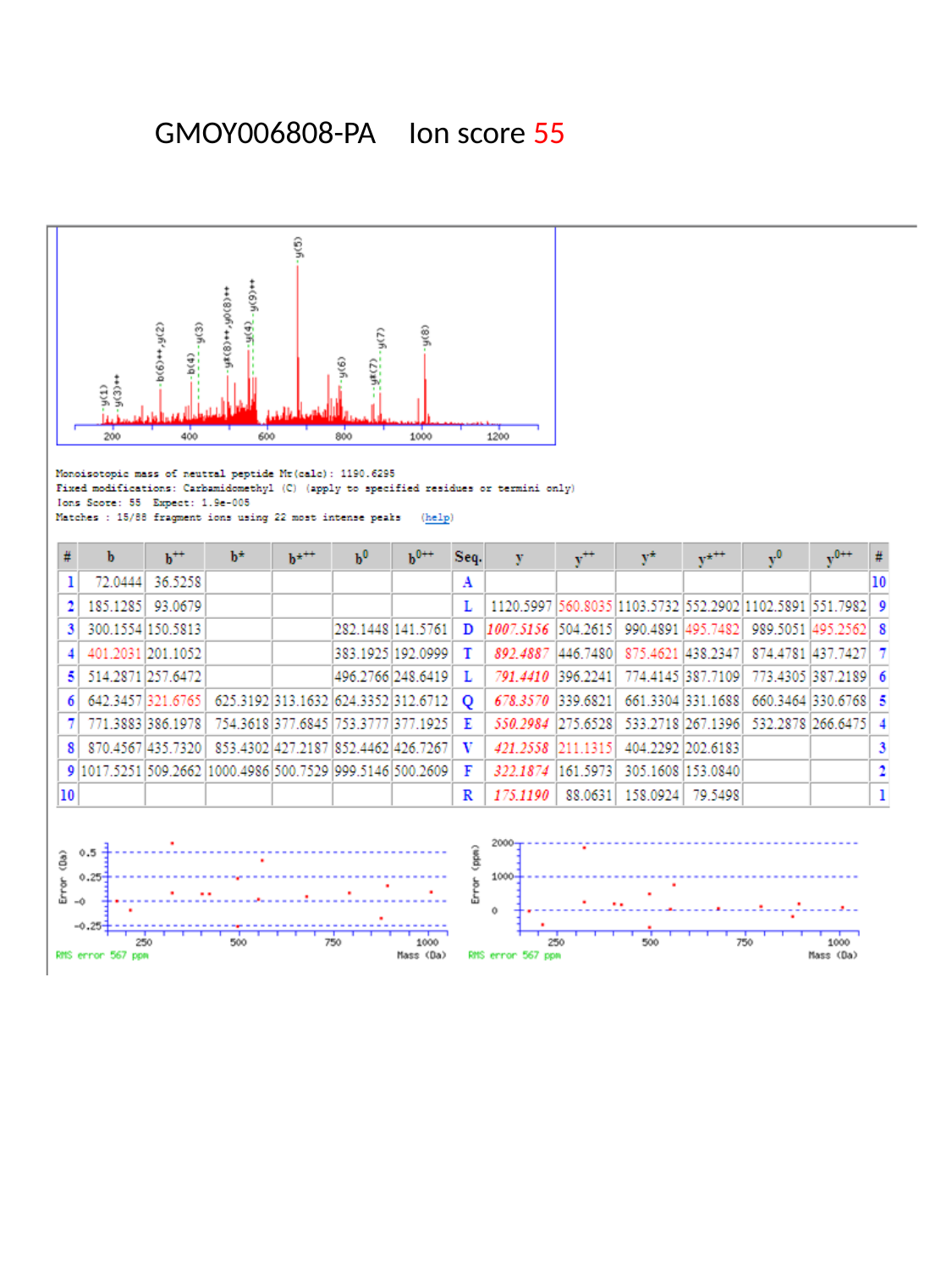

GMOY006808-PA 	Ion score 55

## Slide 104
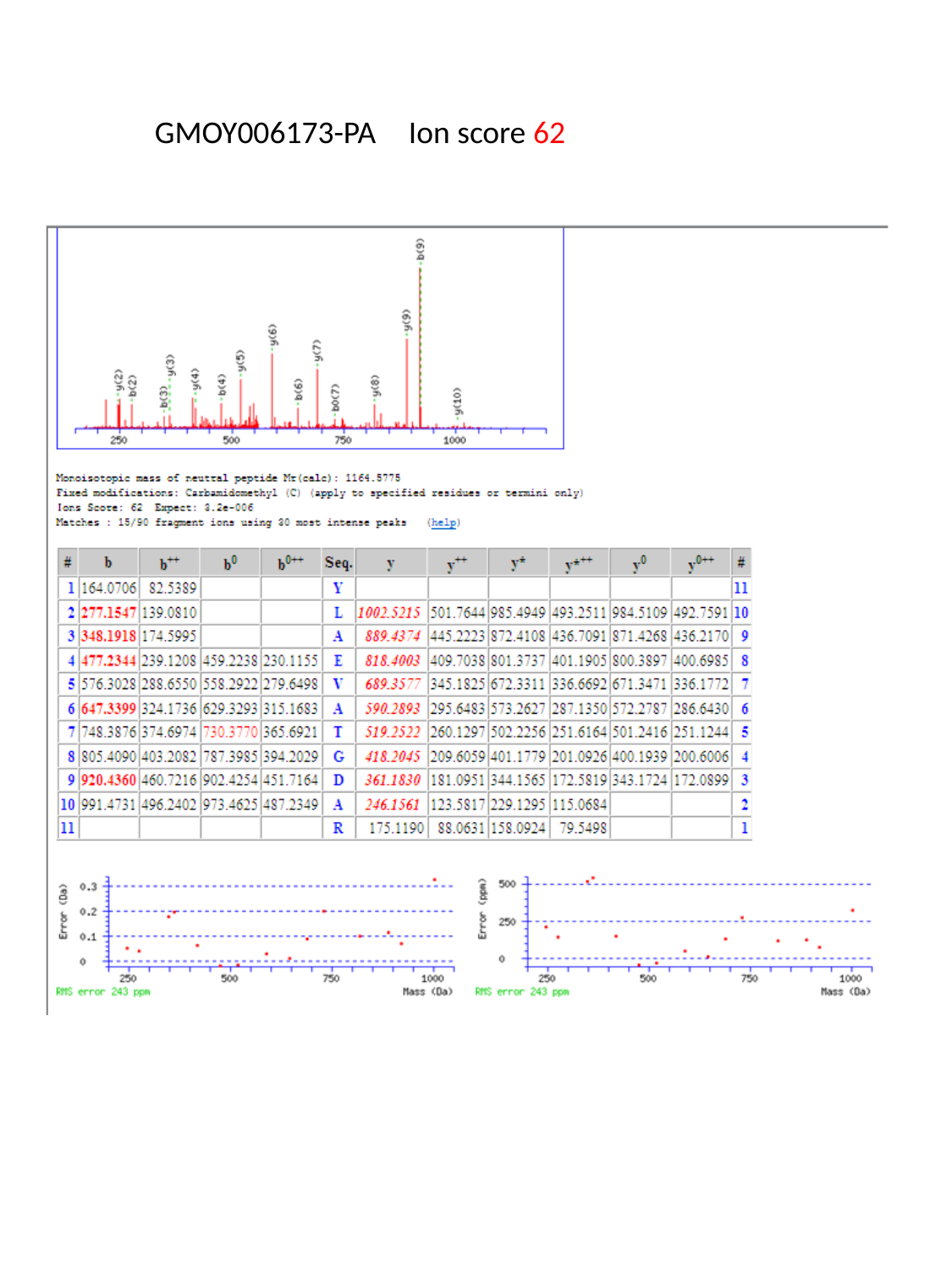

GMOY006173-PA 	Ion score 62

## Slide 105
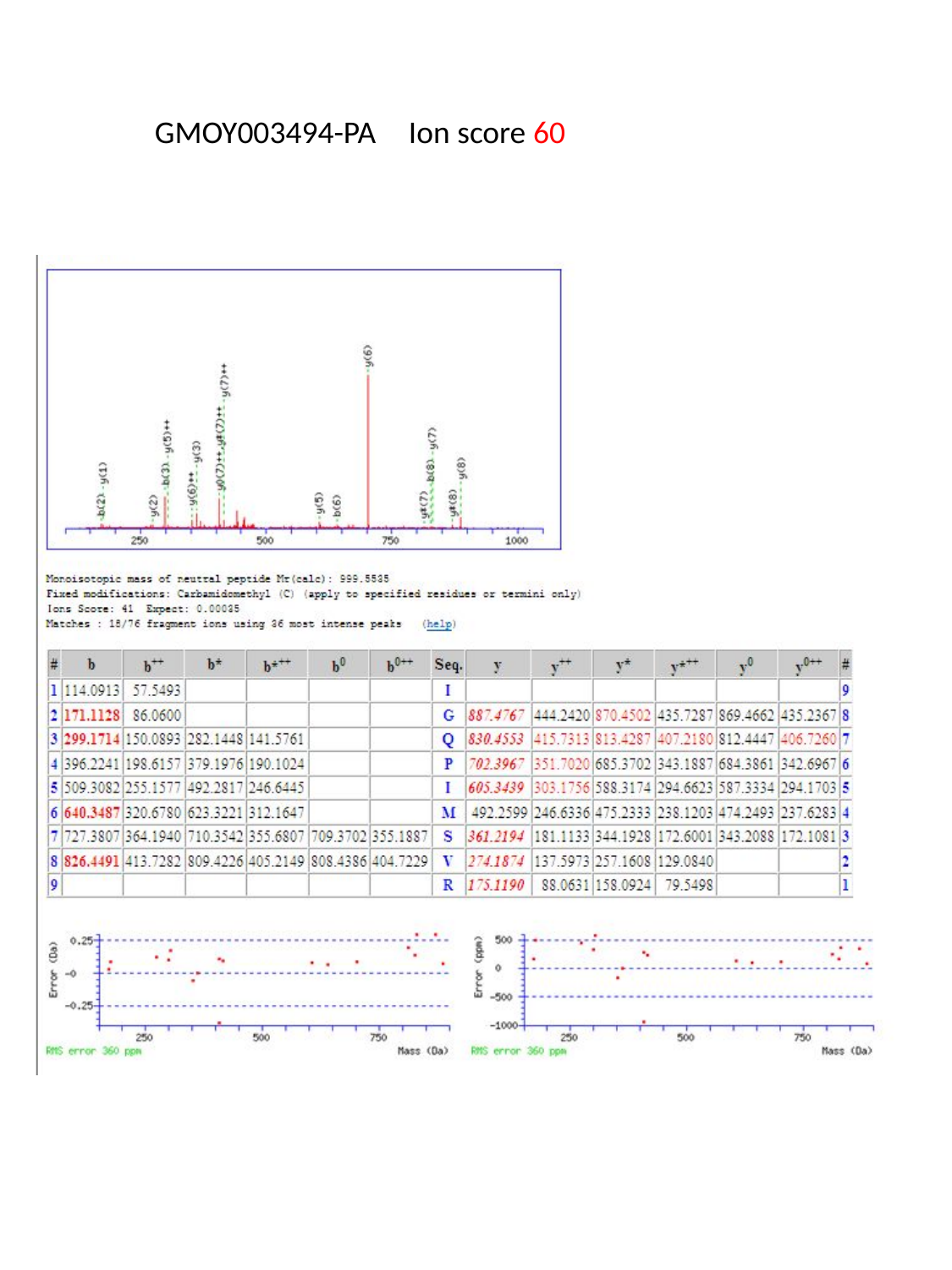

GMOY003494-PA 	Ion score 60

## Slide 106
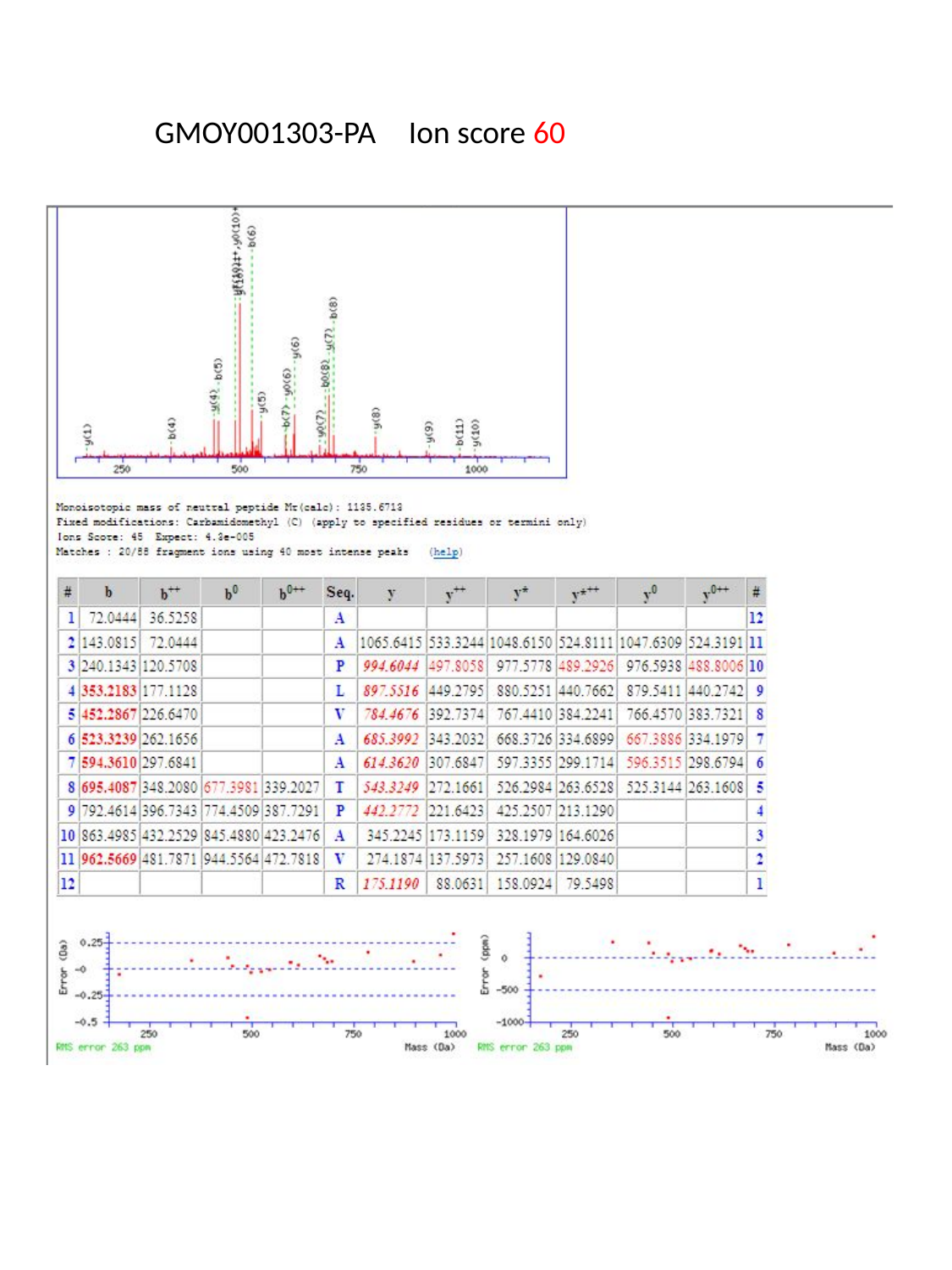

GMOY001303-PA 	Ion score 60

## Slide 107
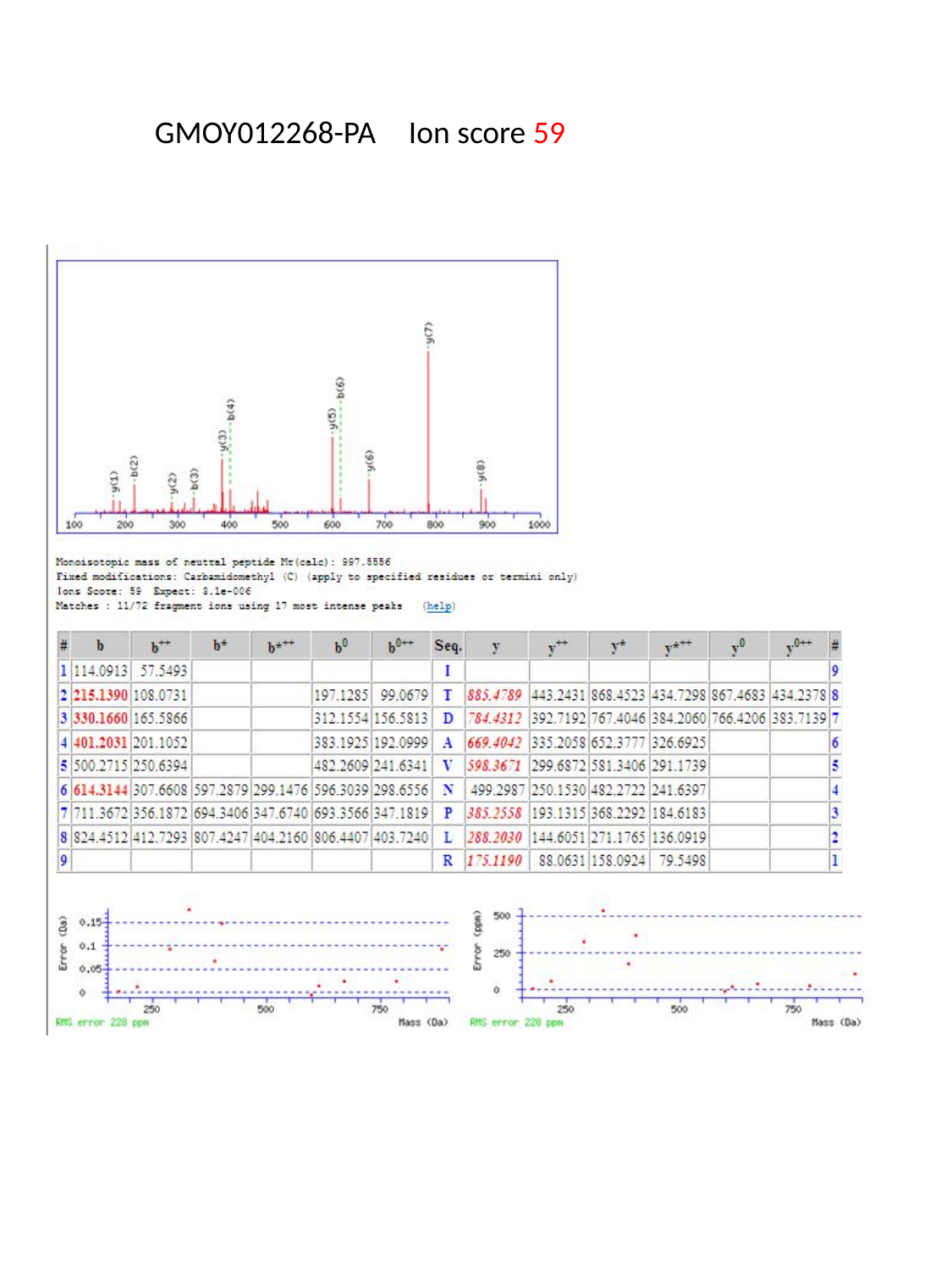

GMOY012268-PA 	Ion score 59

## Slide 108
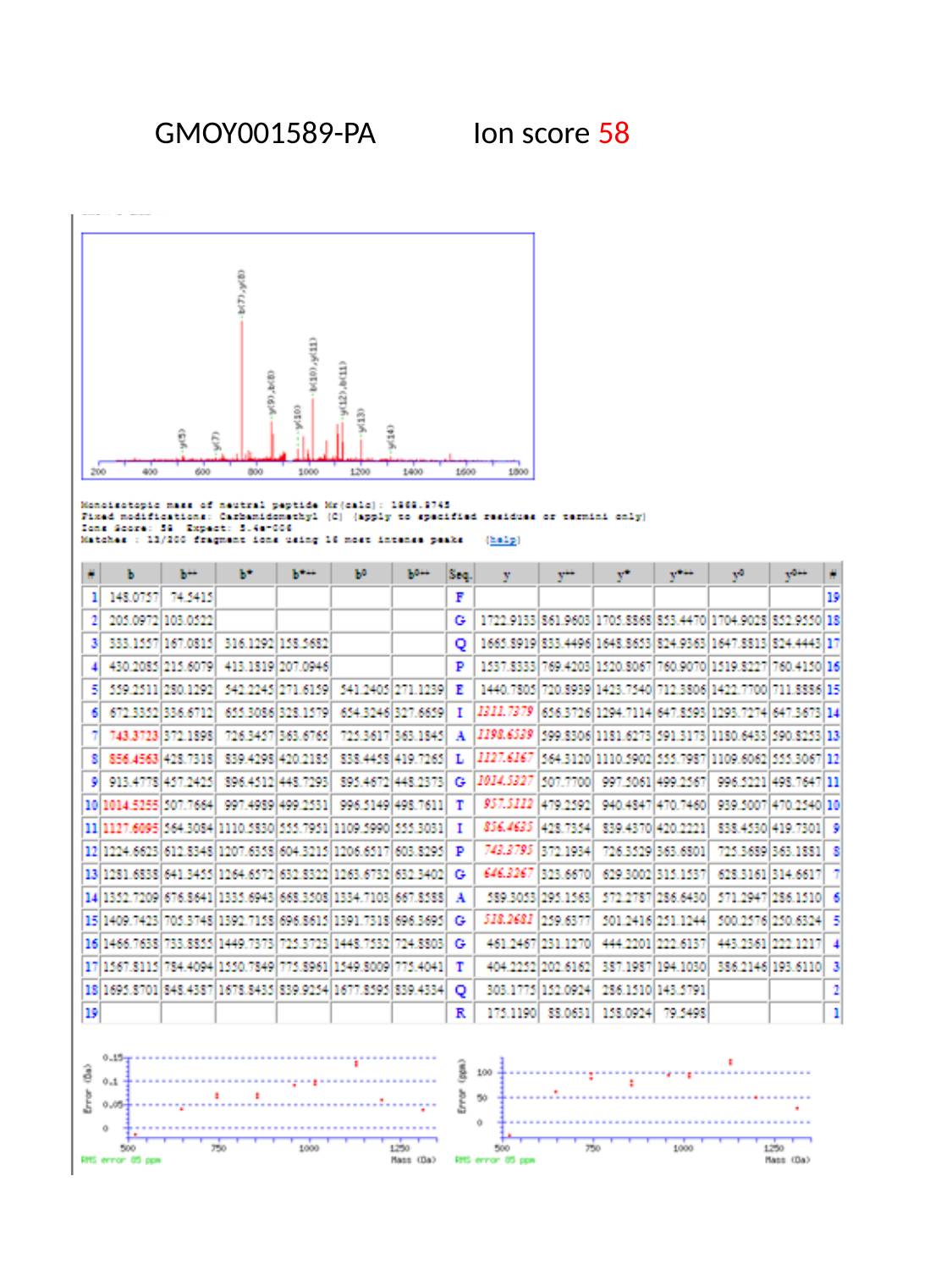

GMOY001589-PA 	 Ion score 58

## Slide 109
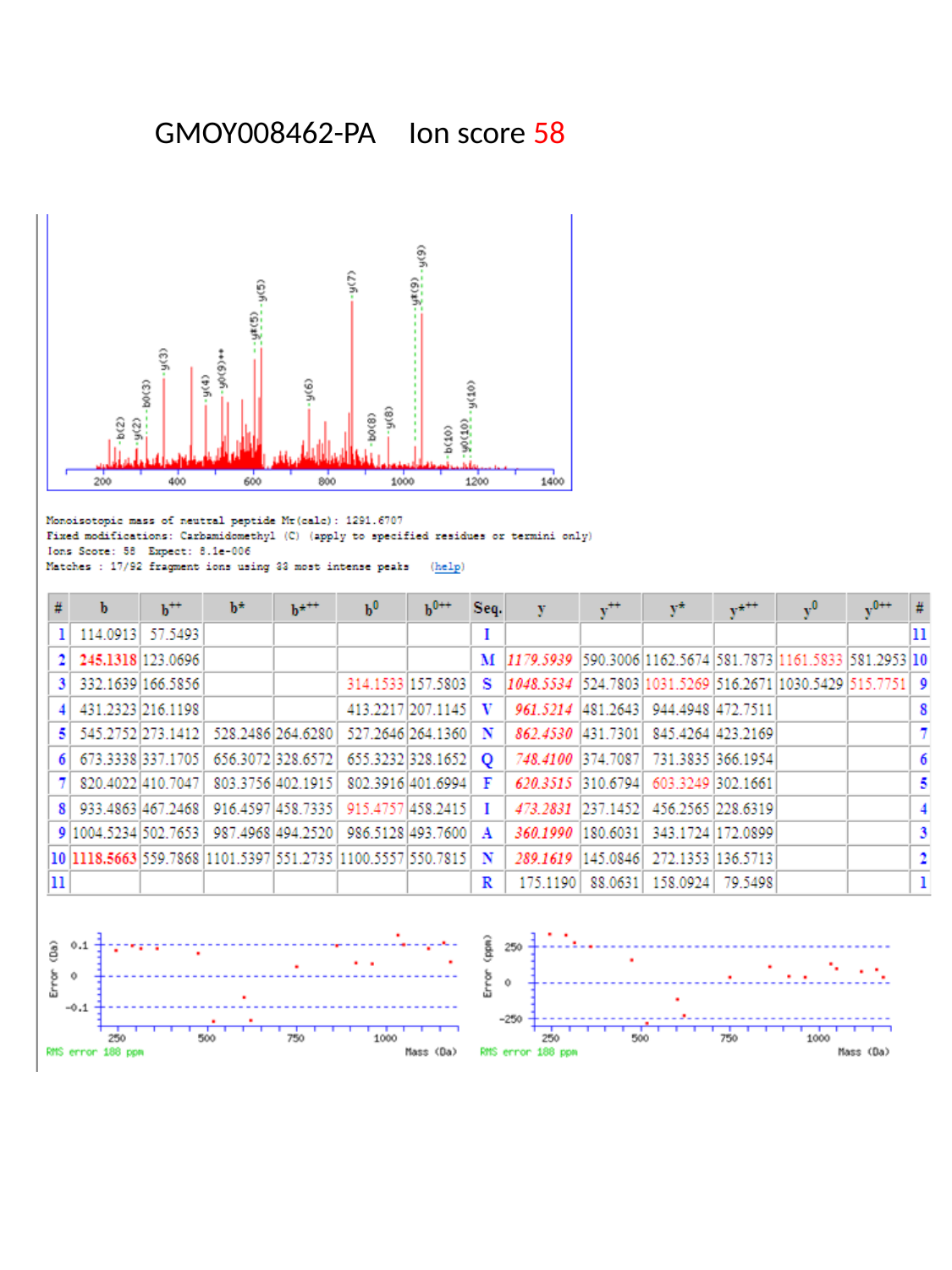

GMOY008462-PA 	Ion score 58

## Slide 110
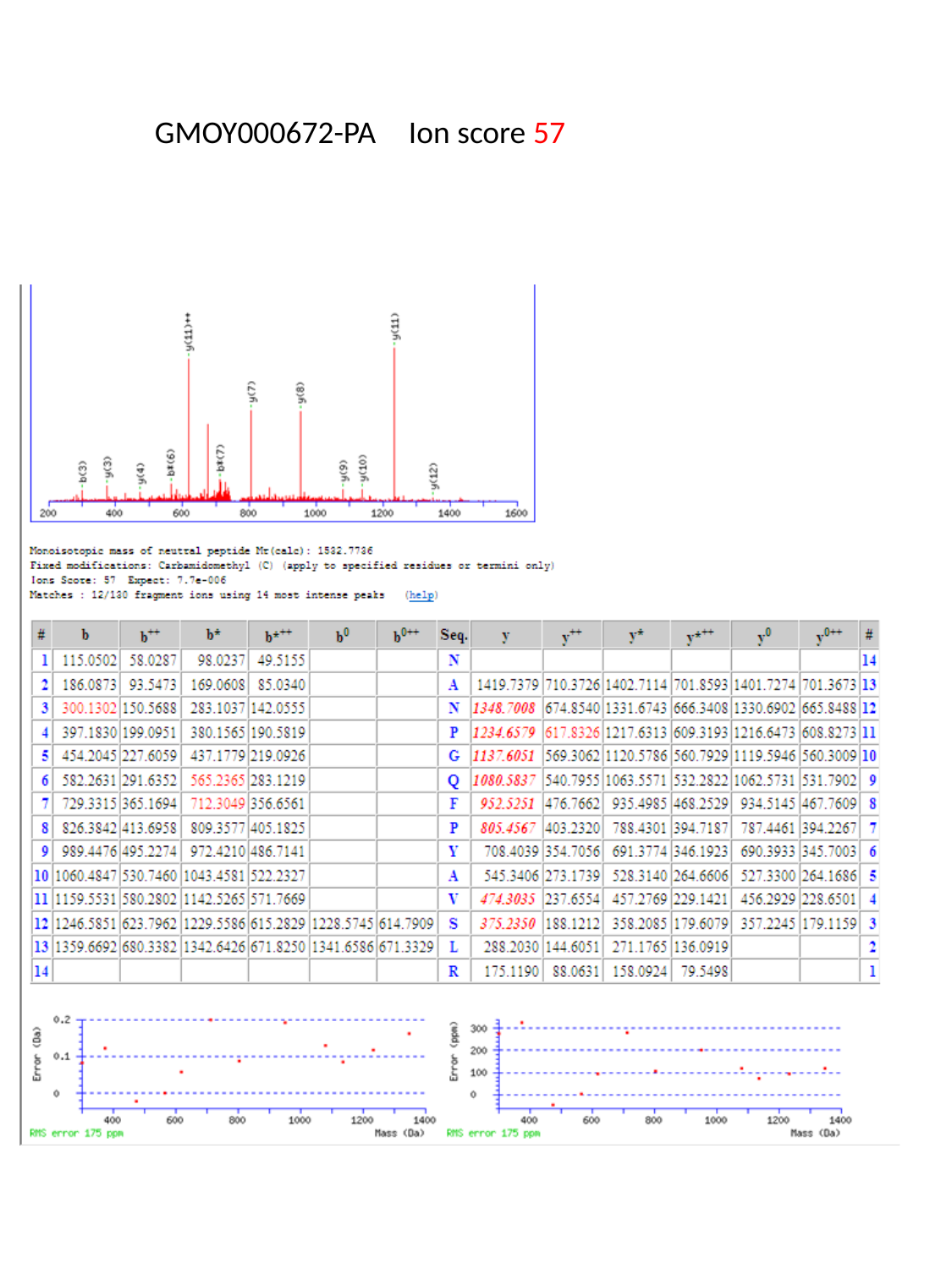

GMOY000672-PA 	Ion score 57

## Slide 111
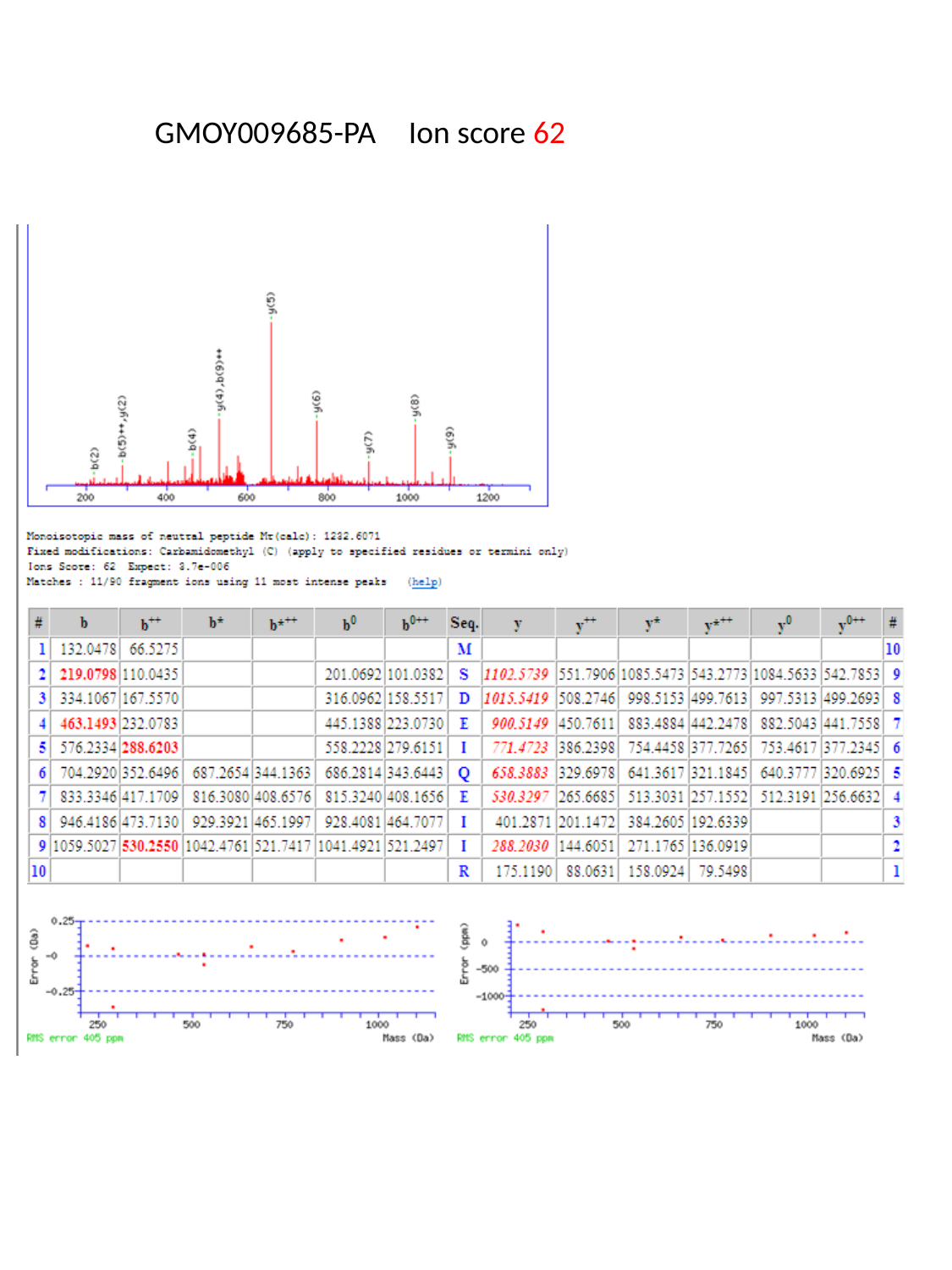

GMOY009685-PA 	Ion score 62

## Slide 112
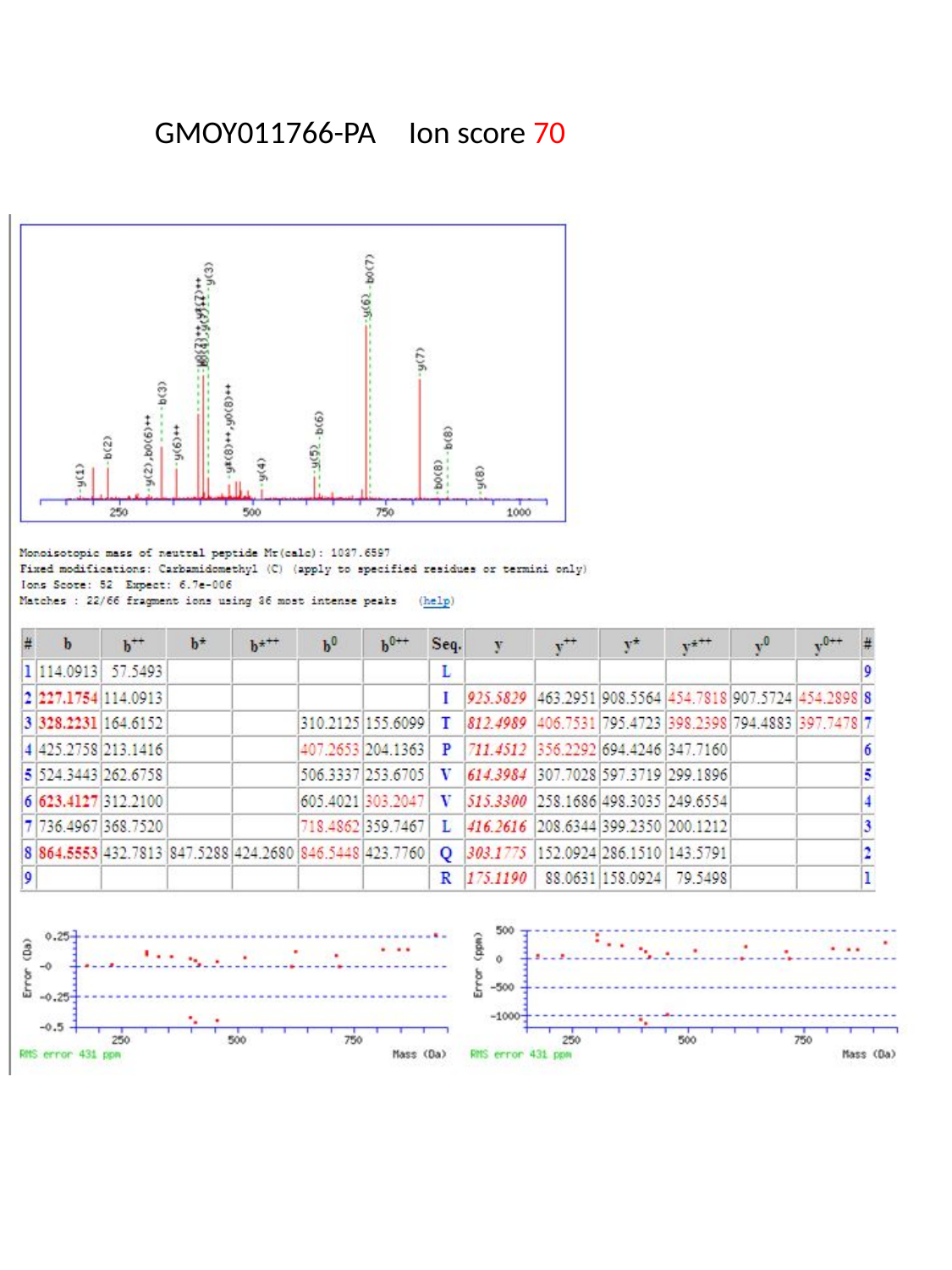

GMOY011766-PA 	Ion score 70

## Slide 113
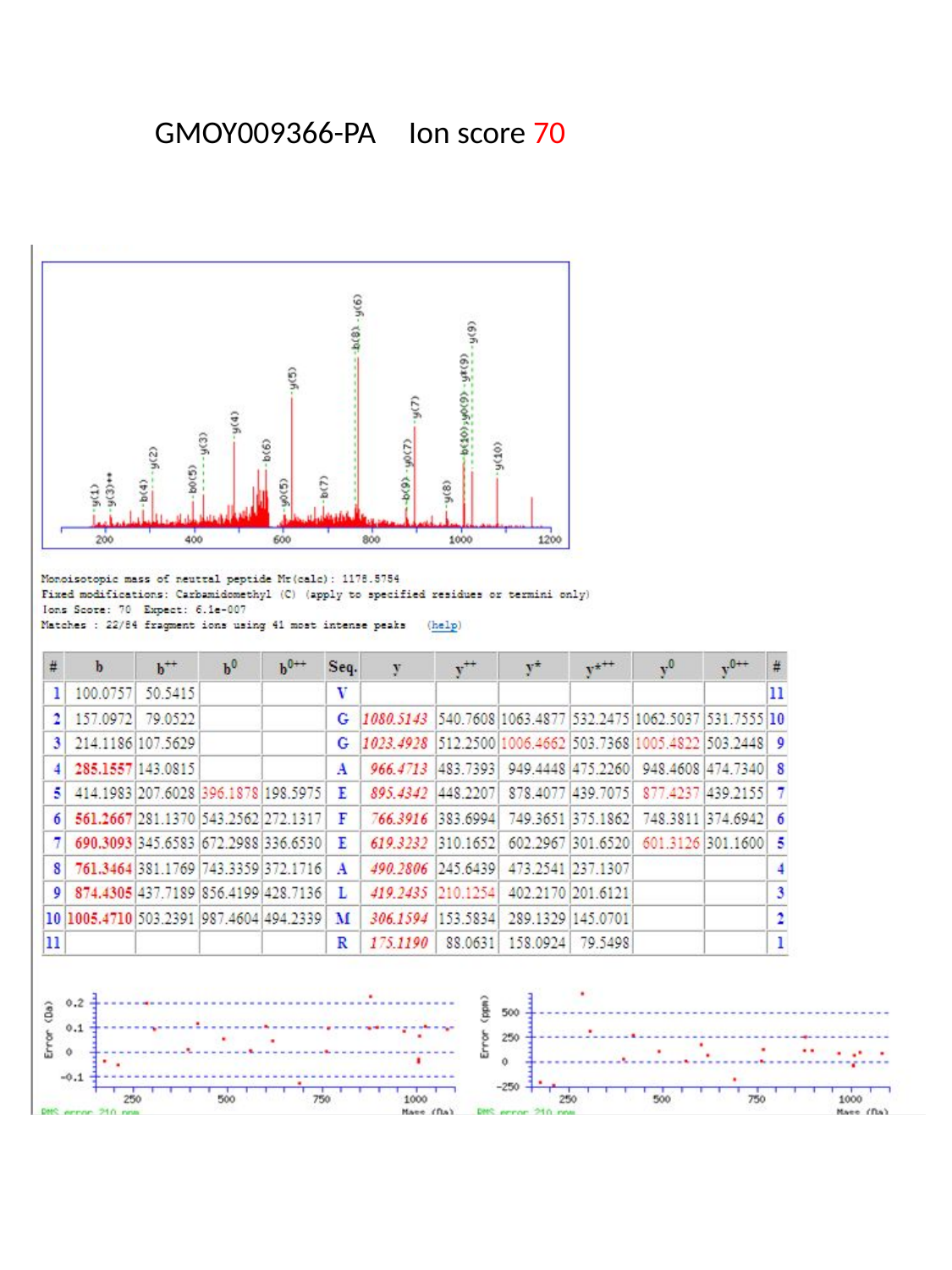

GMOY009366-PA 	Ion score 70

## Slide 114
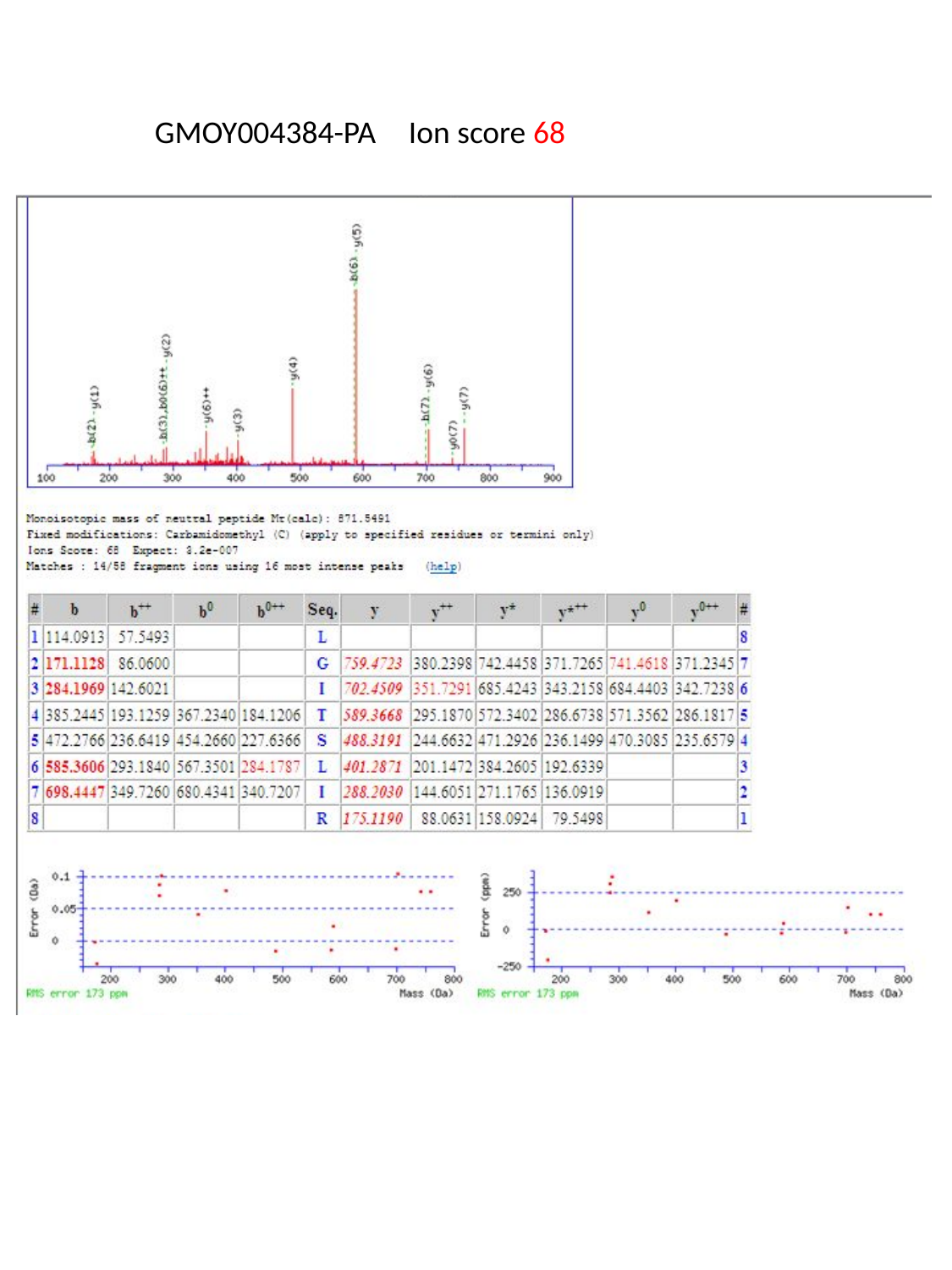

GMOY004384-PA 	Ion score 68

## Slide 115
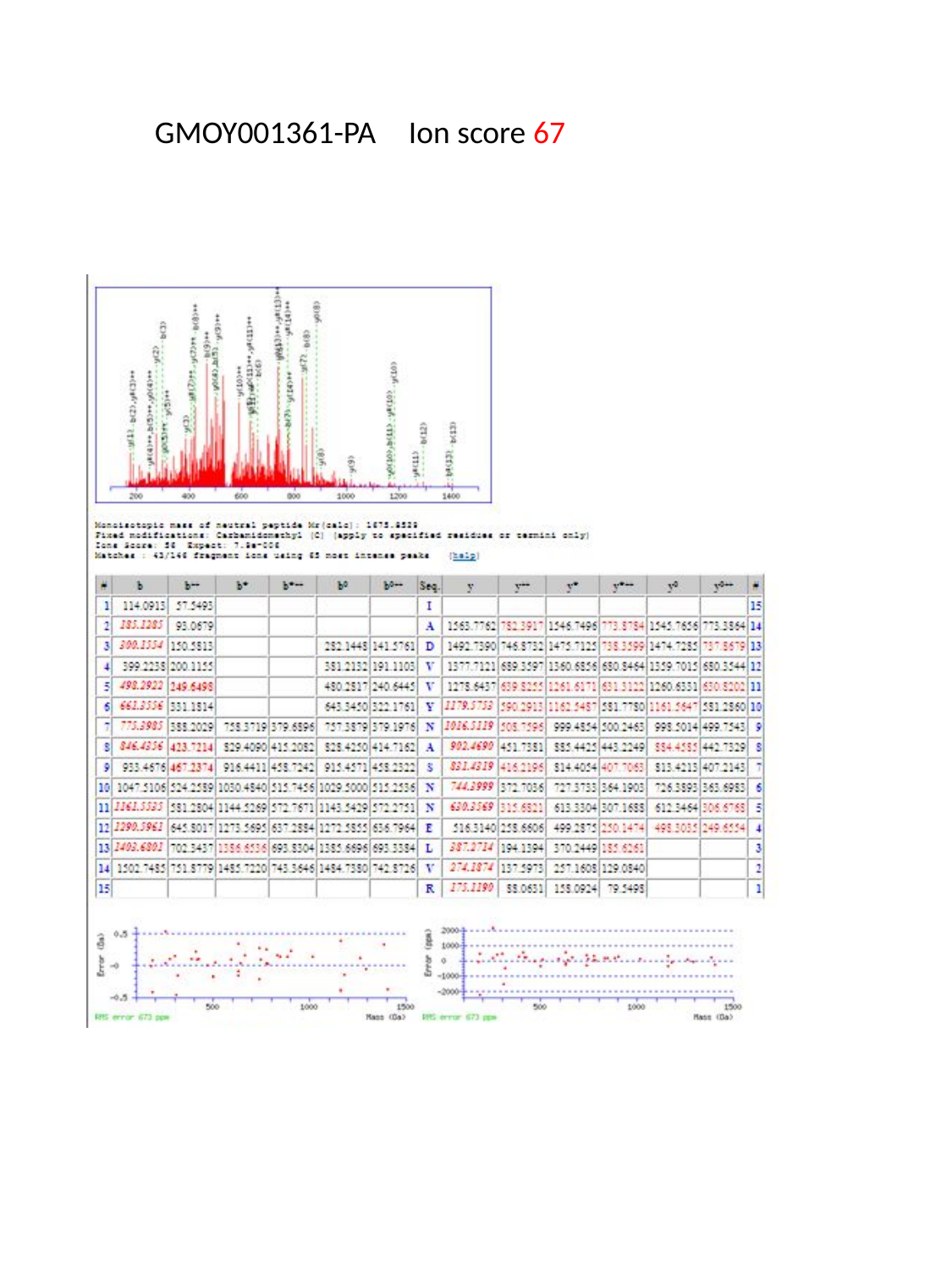

GMOY001361-PA 	Ion score 67

## Slide 116
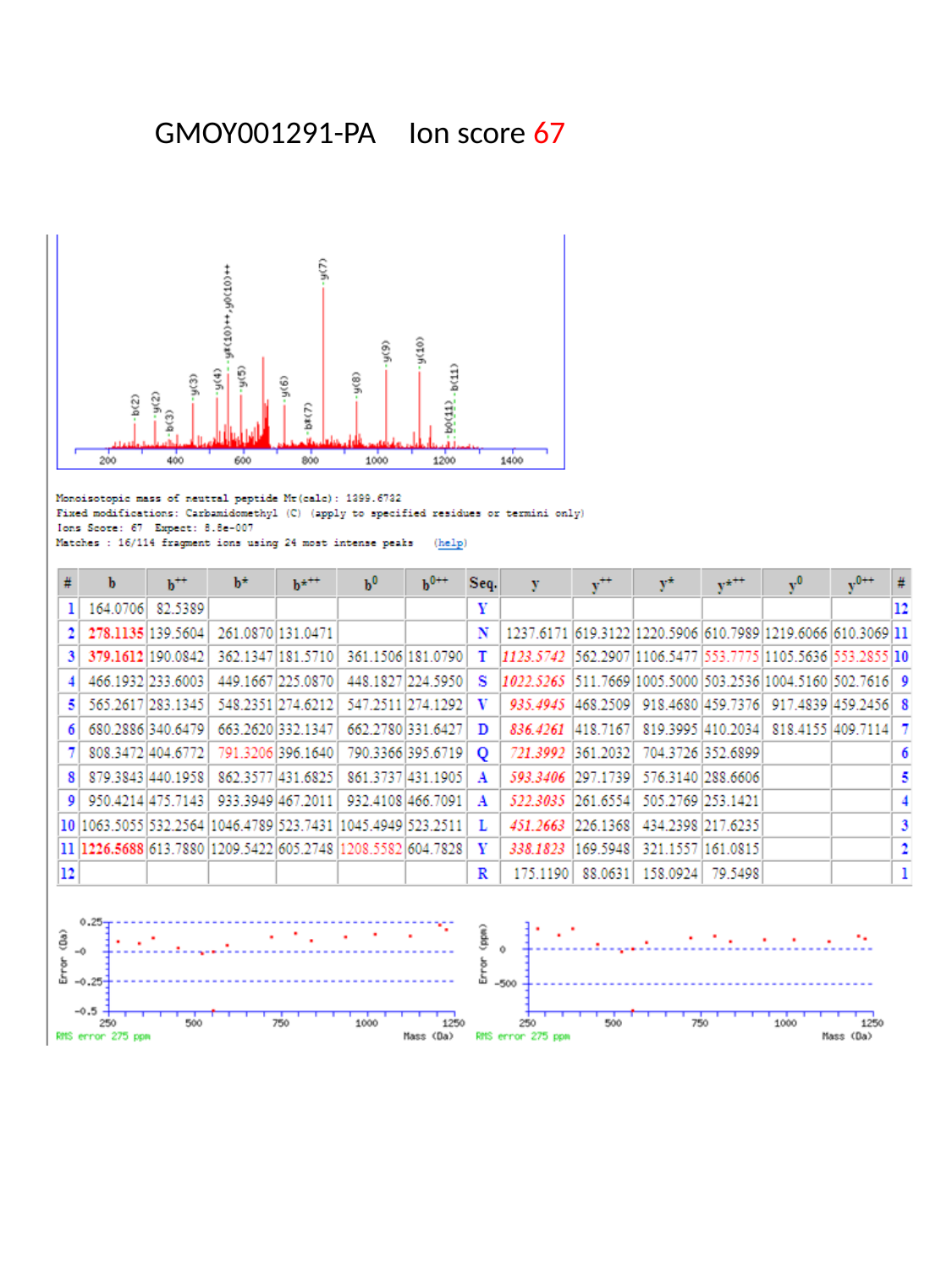

GMOY001291-PA 	Ion score 67

## Slide 117
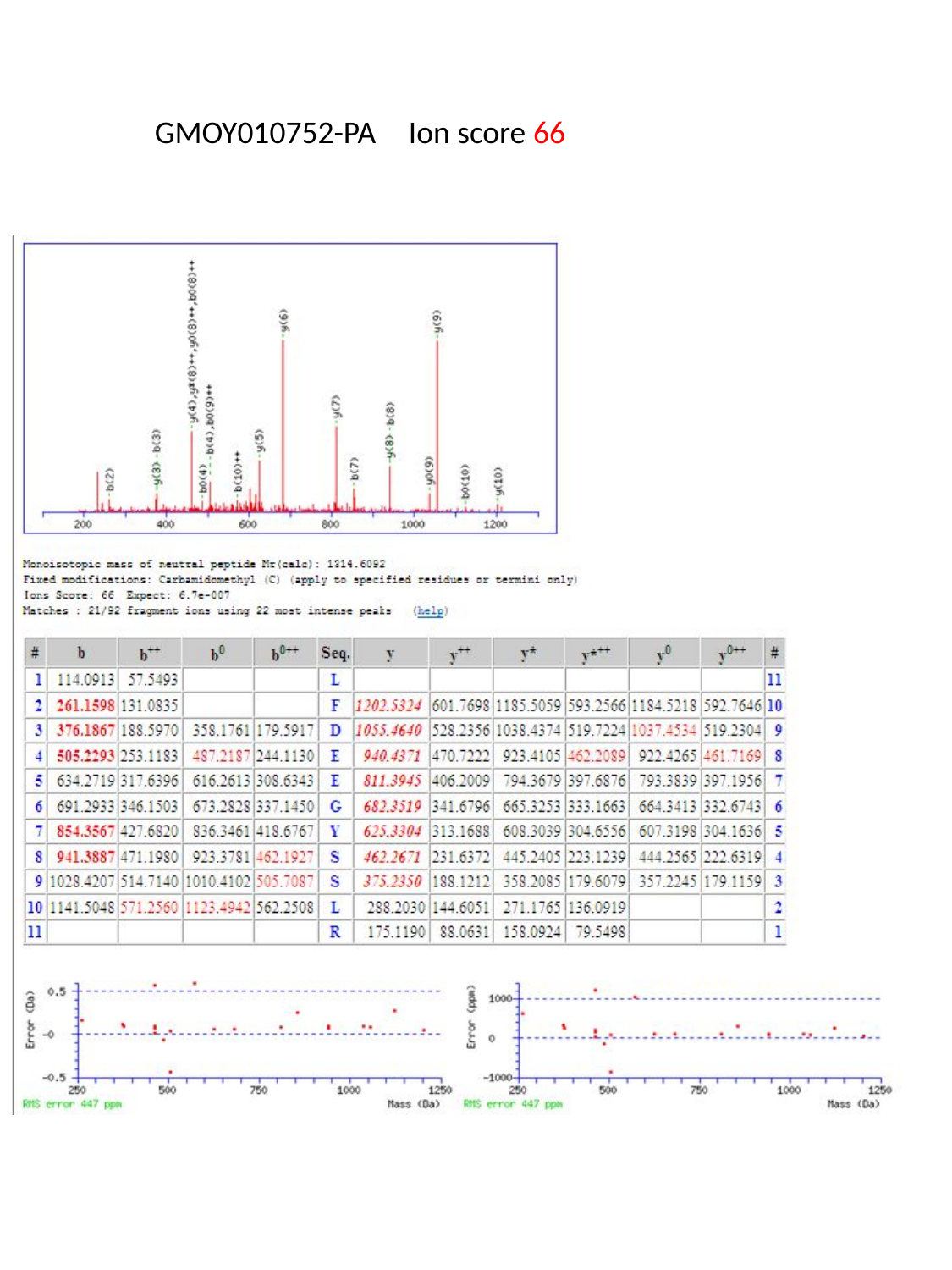

GMOY010752-PA	Ion score 66

## Slide 118
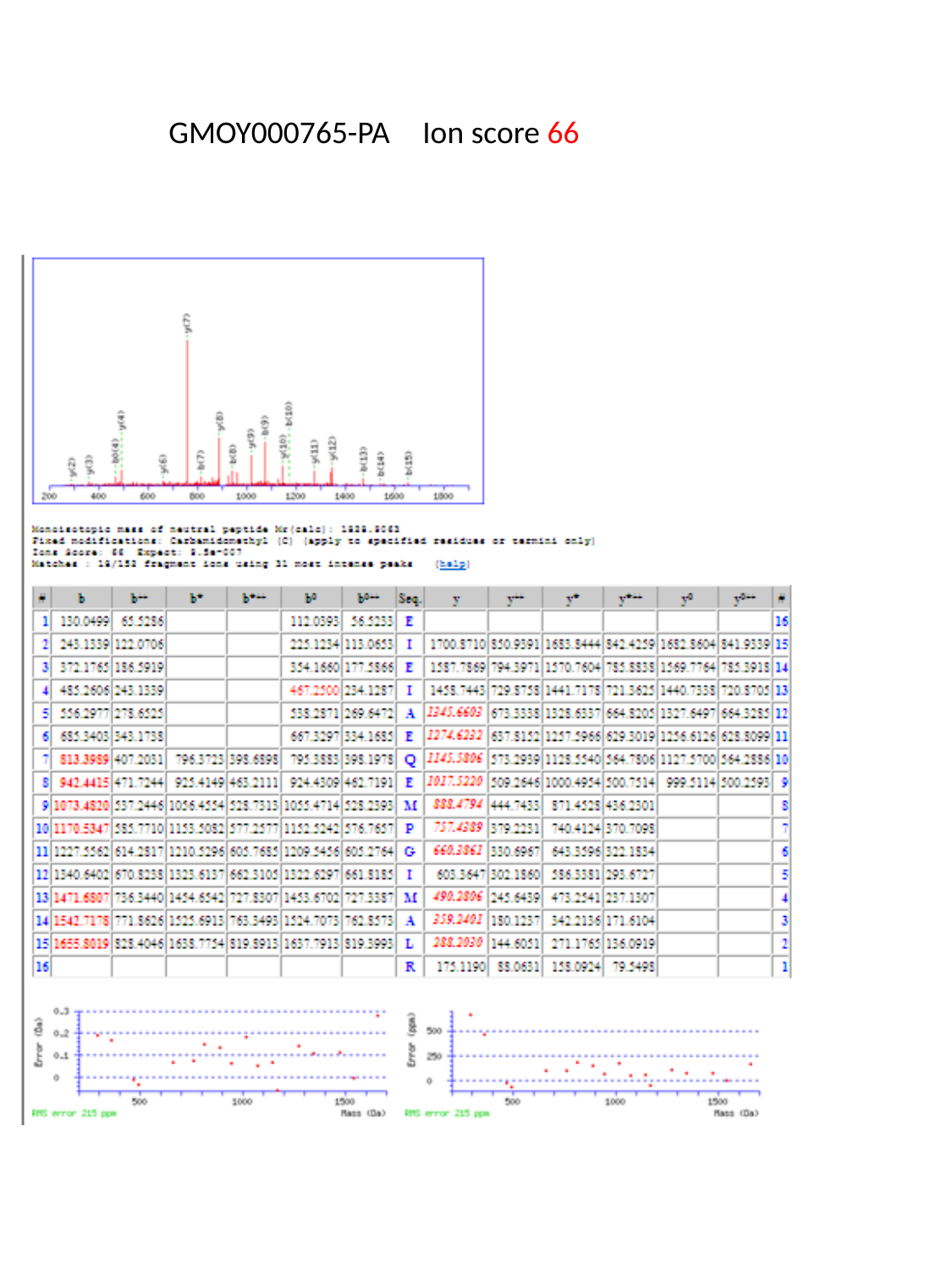

GMOY000765-PA	Ion score 66

## Slide 119
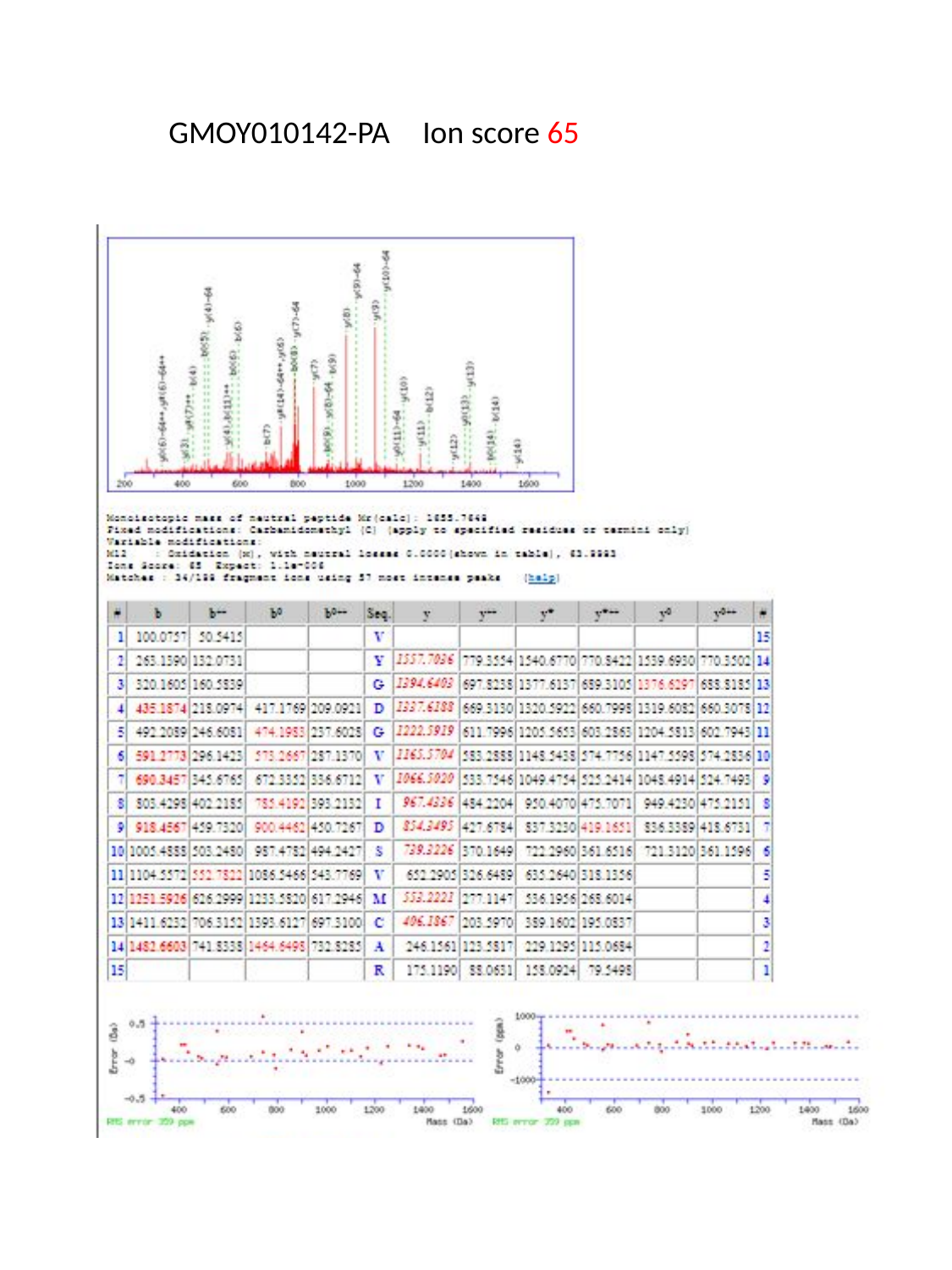

GMOY010142-PA	Ion score 65

## Slide 120
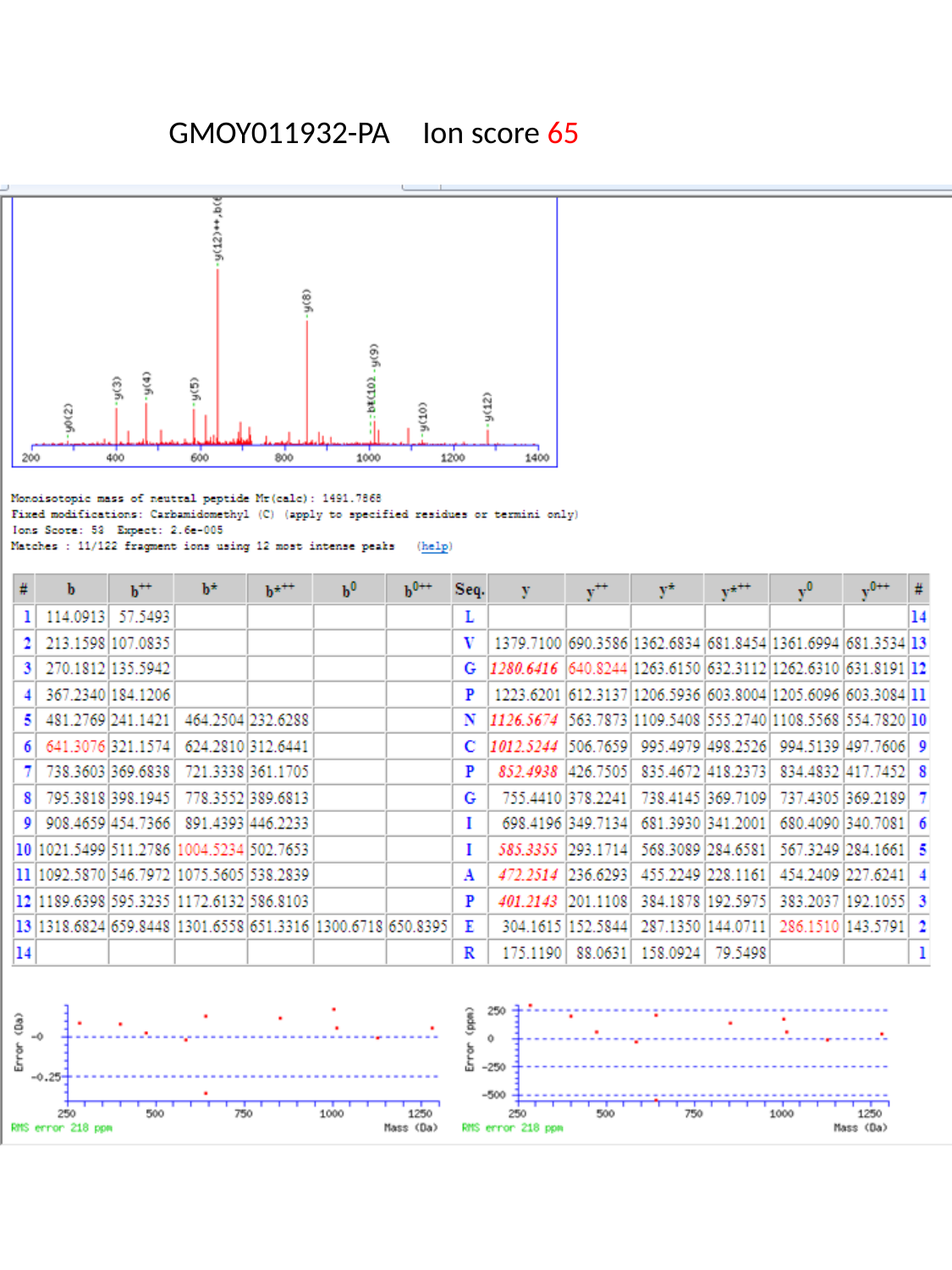

GMOY011932-PA 	Ion score 65

## Slide 121
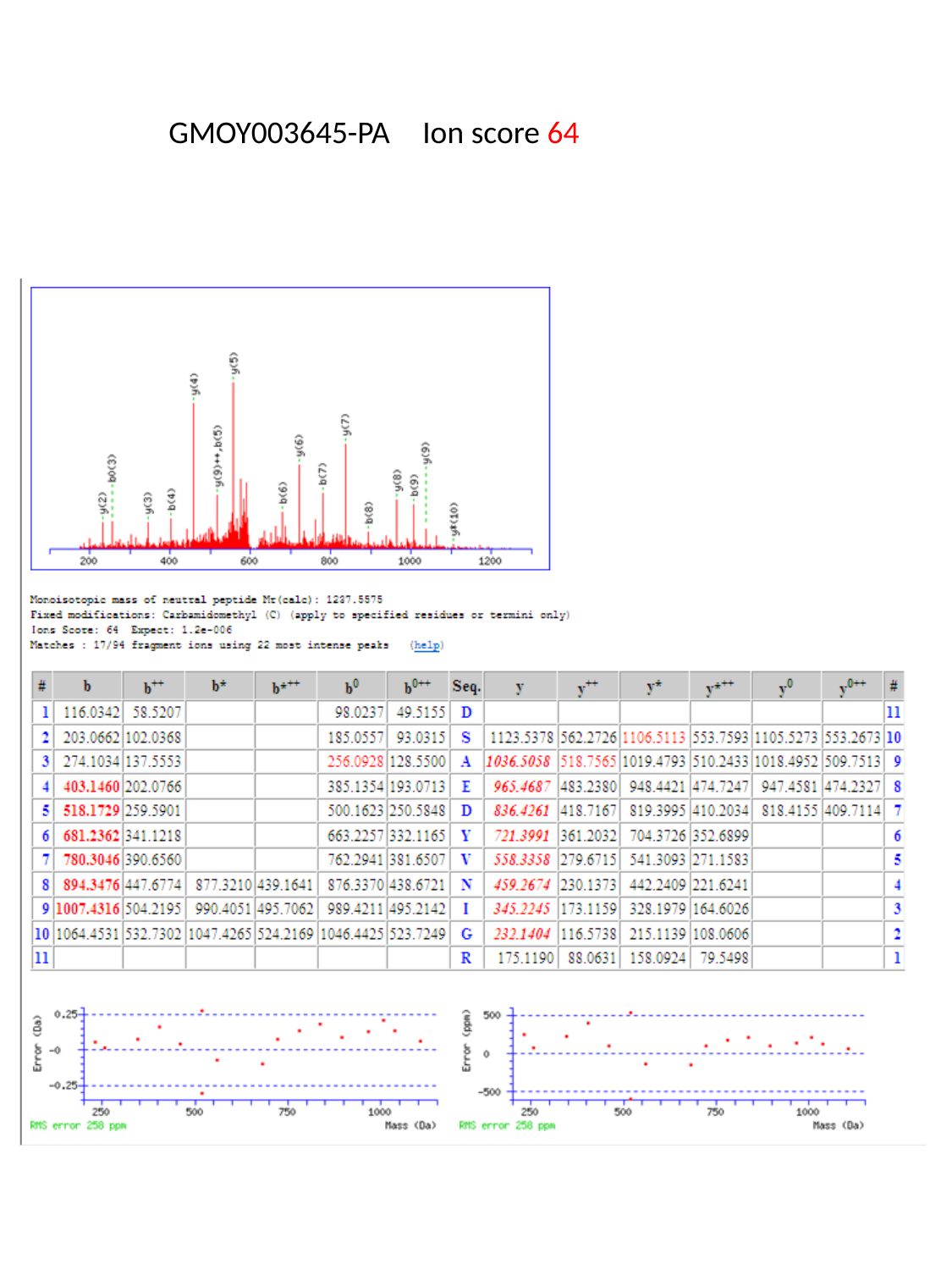

GMOY003645-PA 	Ion score 64

## Slide 122
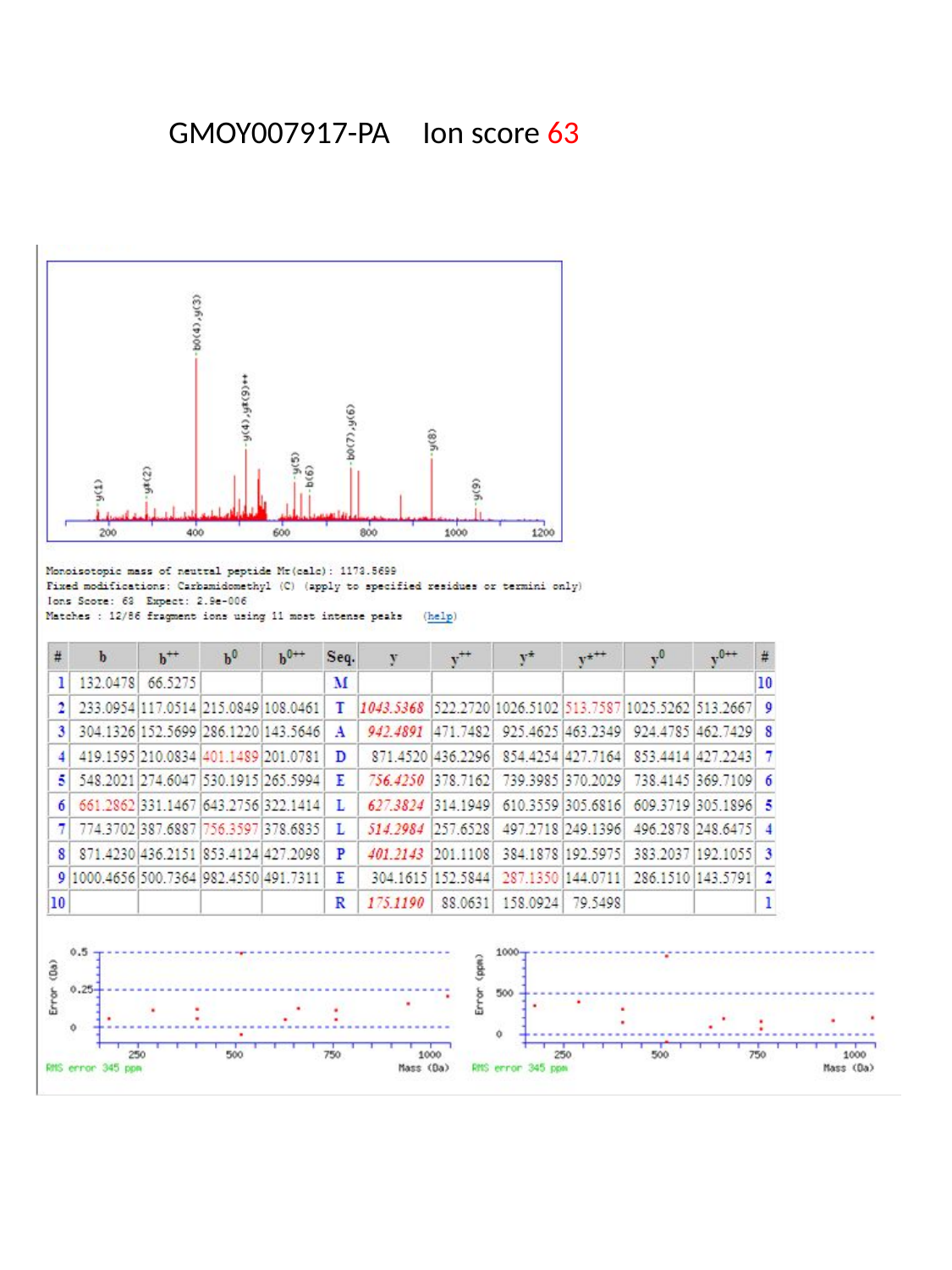

GMOY007917-PA 	Ion score 63

## Slide 123
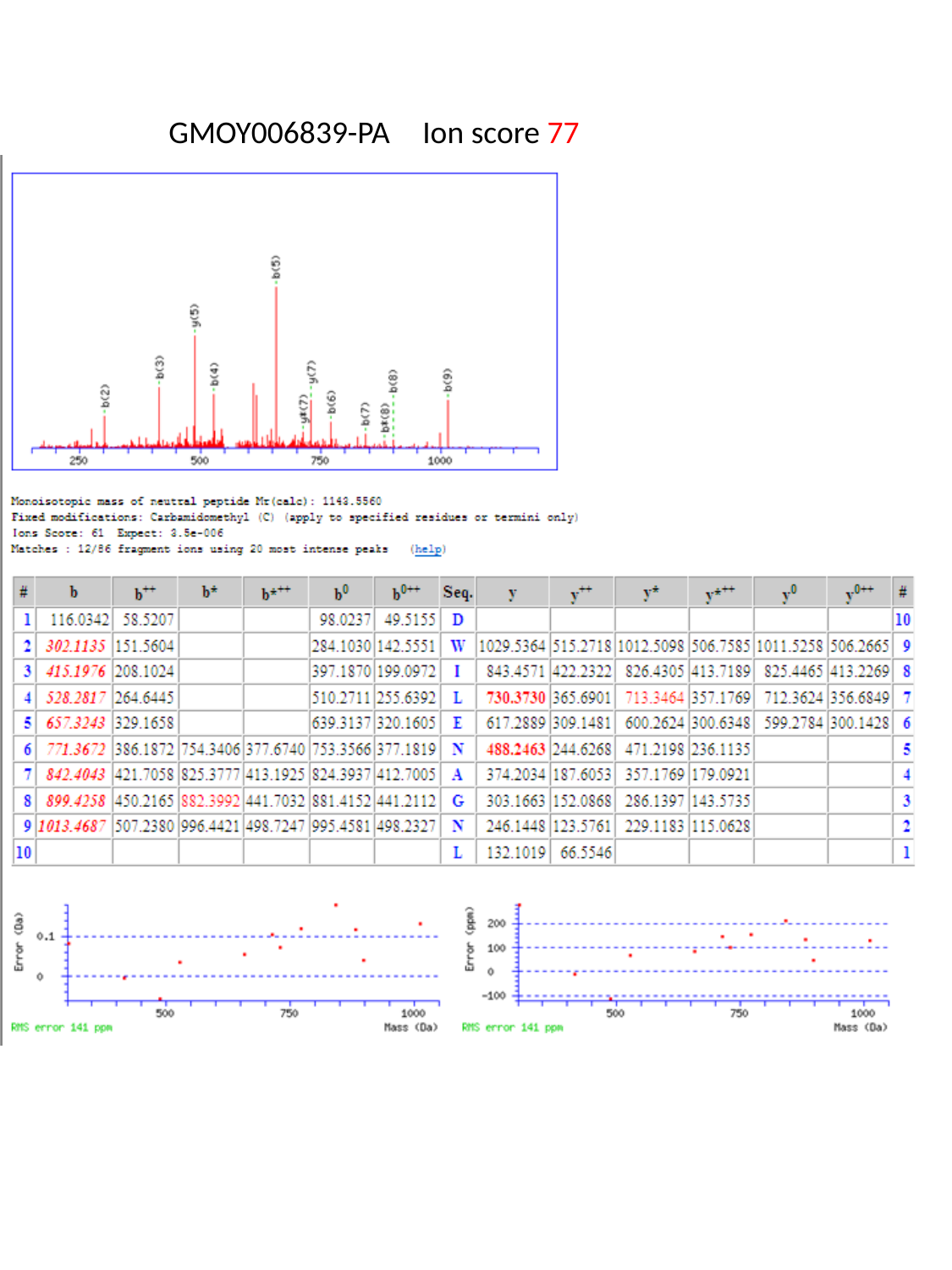

GMOY006839-PA 	Ion score 77

## Slide 124
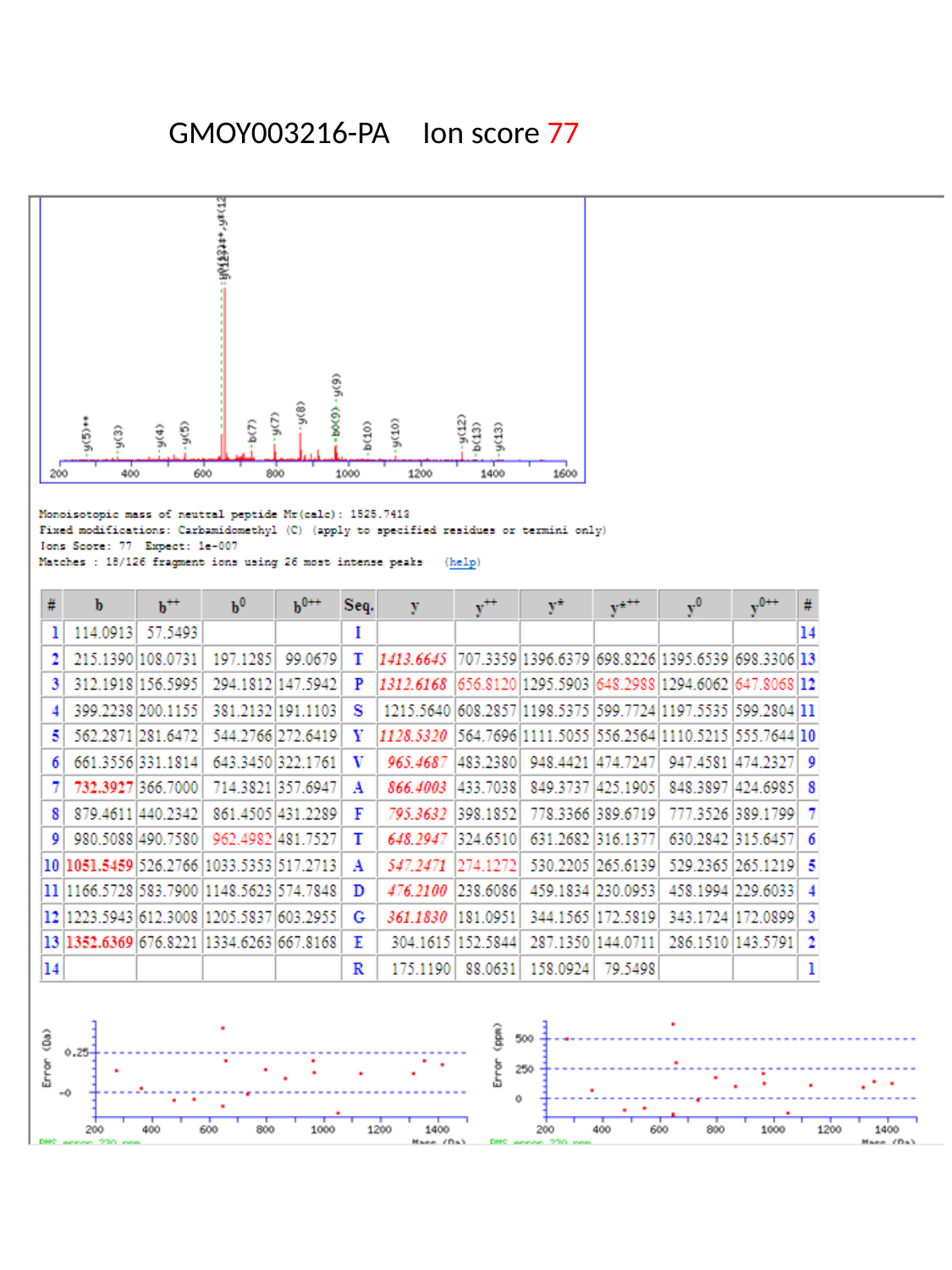

GMOY003216-PA 	Ion score 77

## Slide 125
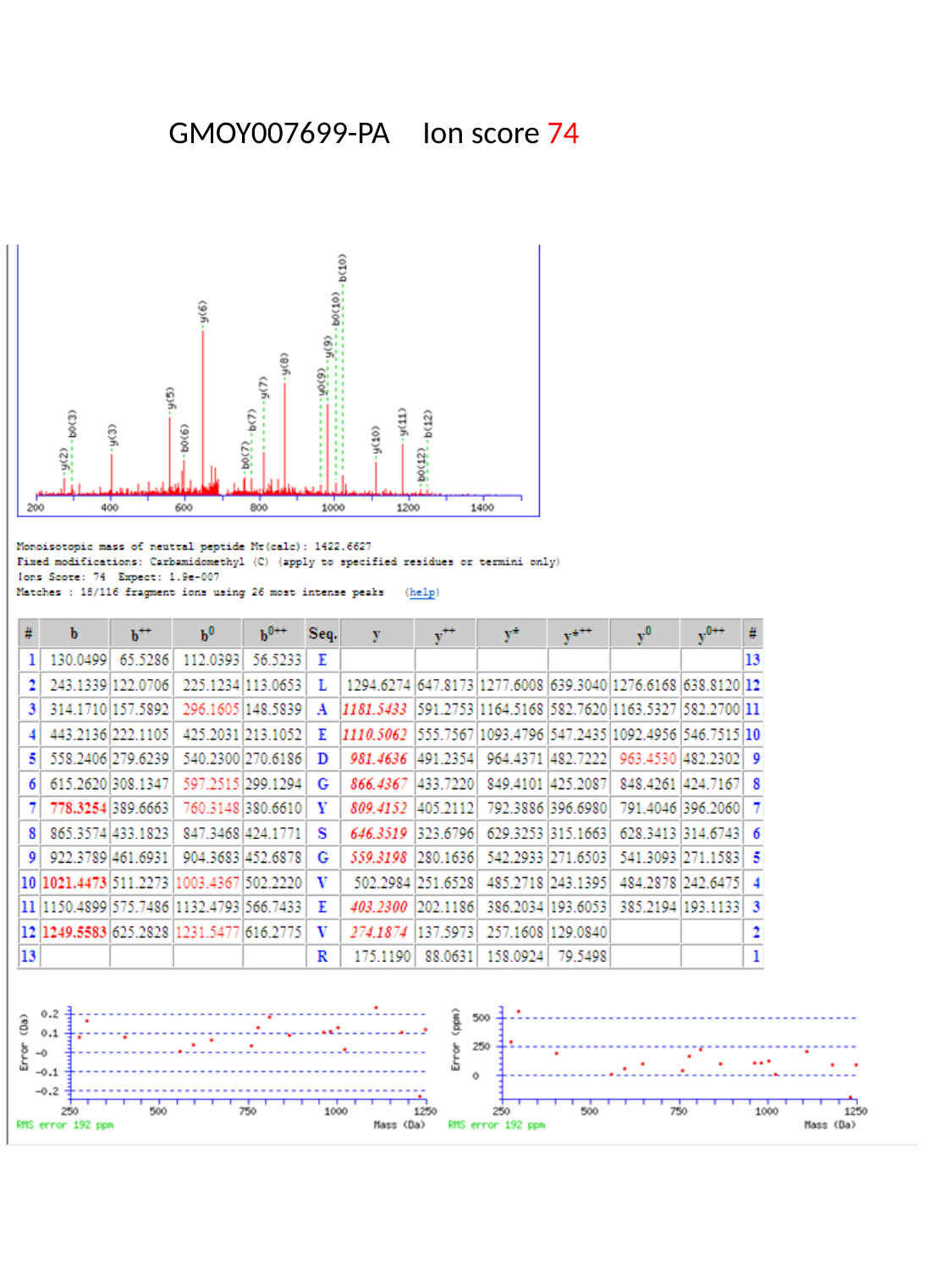

GMOY007699-PA 	Ion score 74

## Slide 126
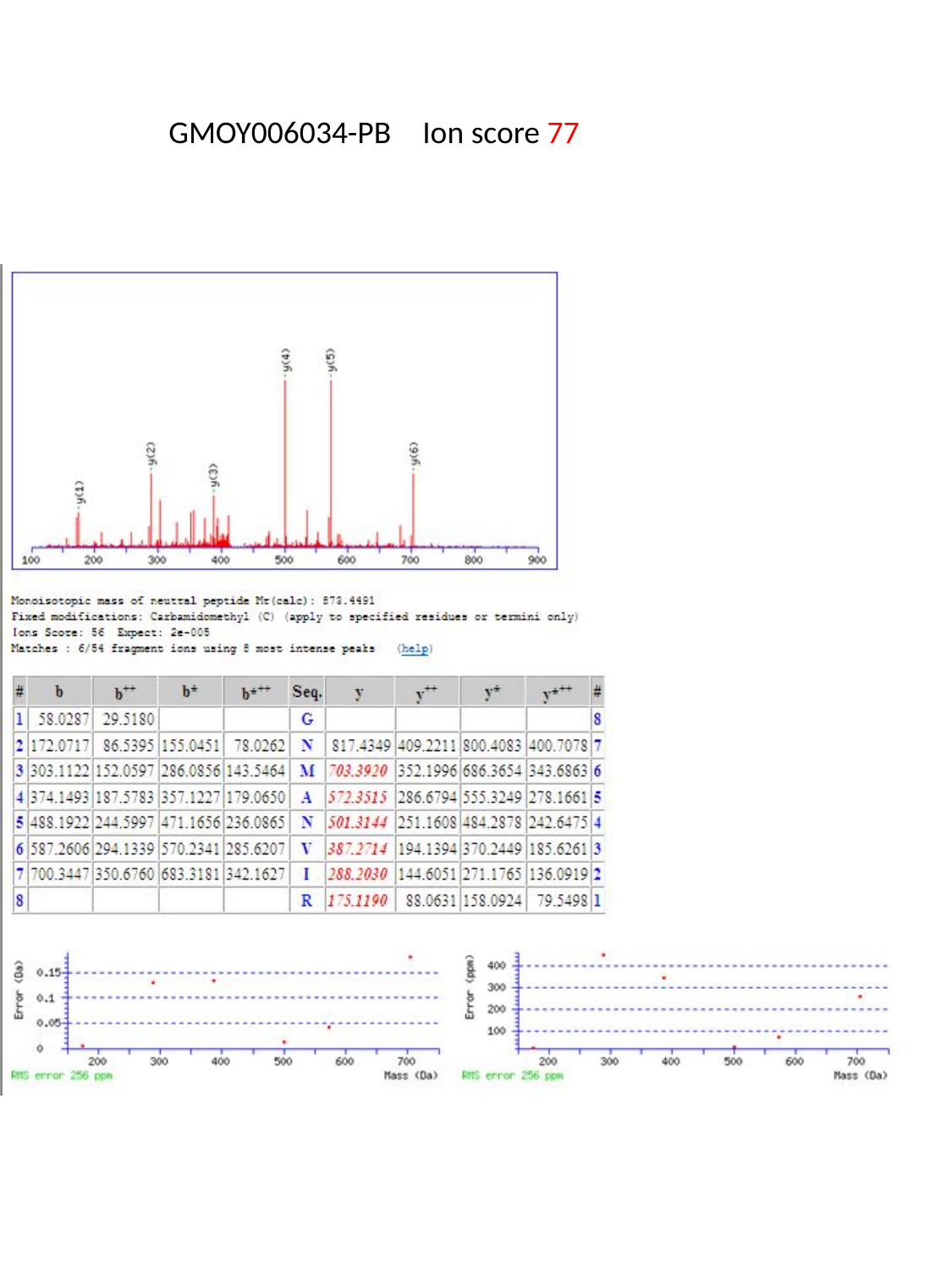

GMOY006034-PB 	Ion score 77

## Slide 127
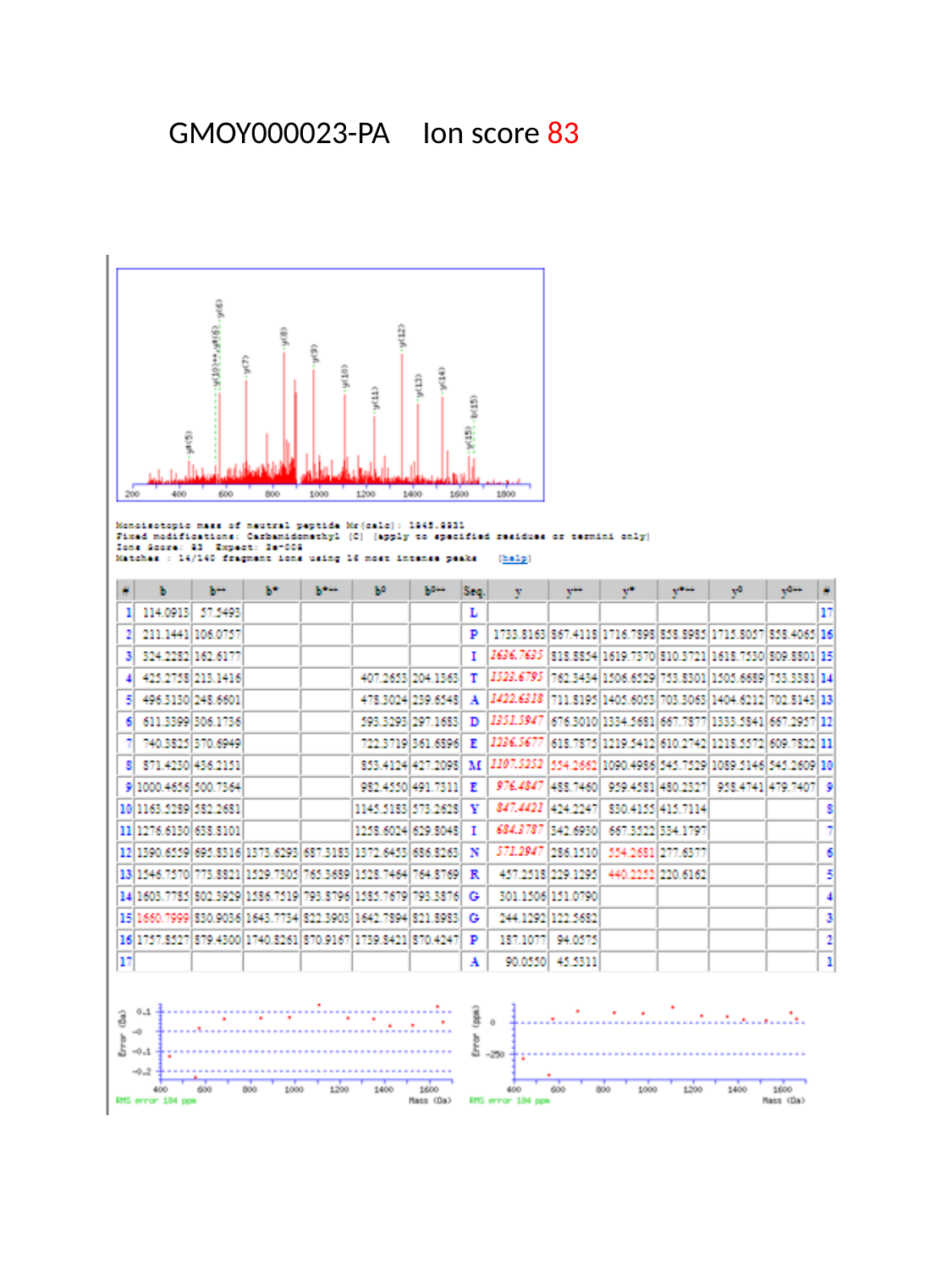

GMOY000023-PA 	Ion score 83

## Slide 128
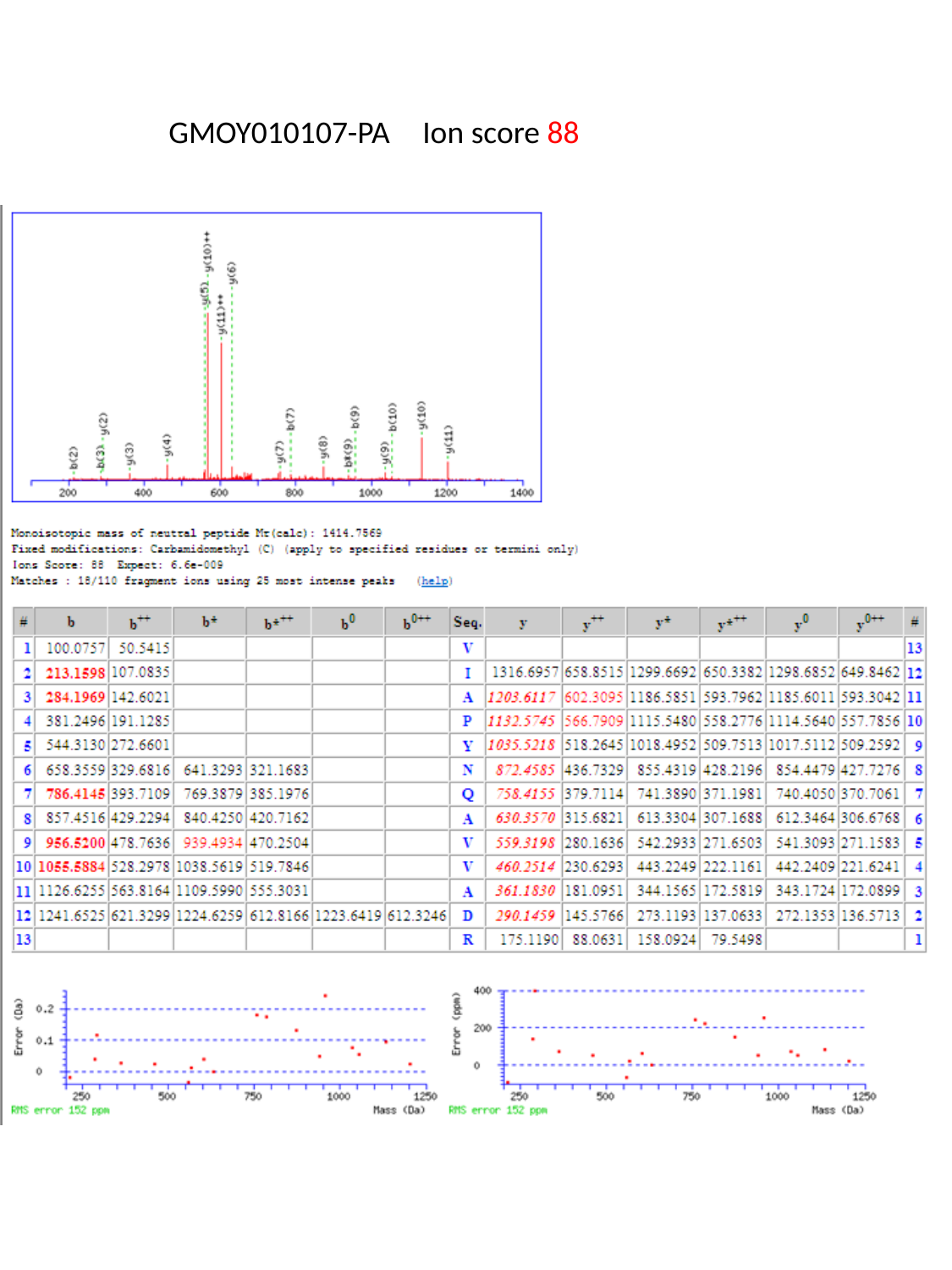

GMOY010107-PA 	Ion score 88

## Slide 129
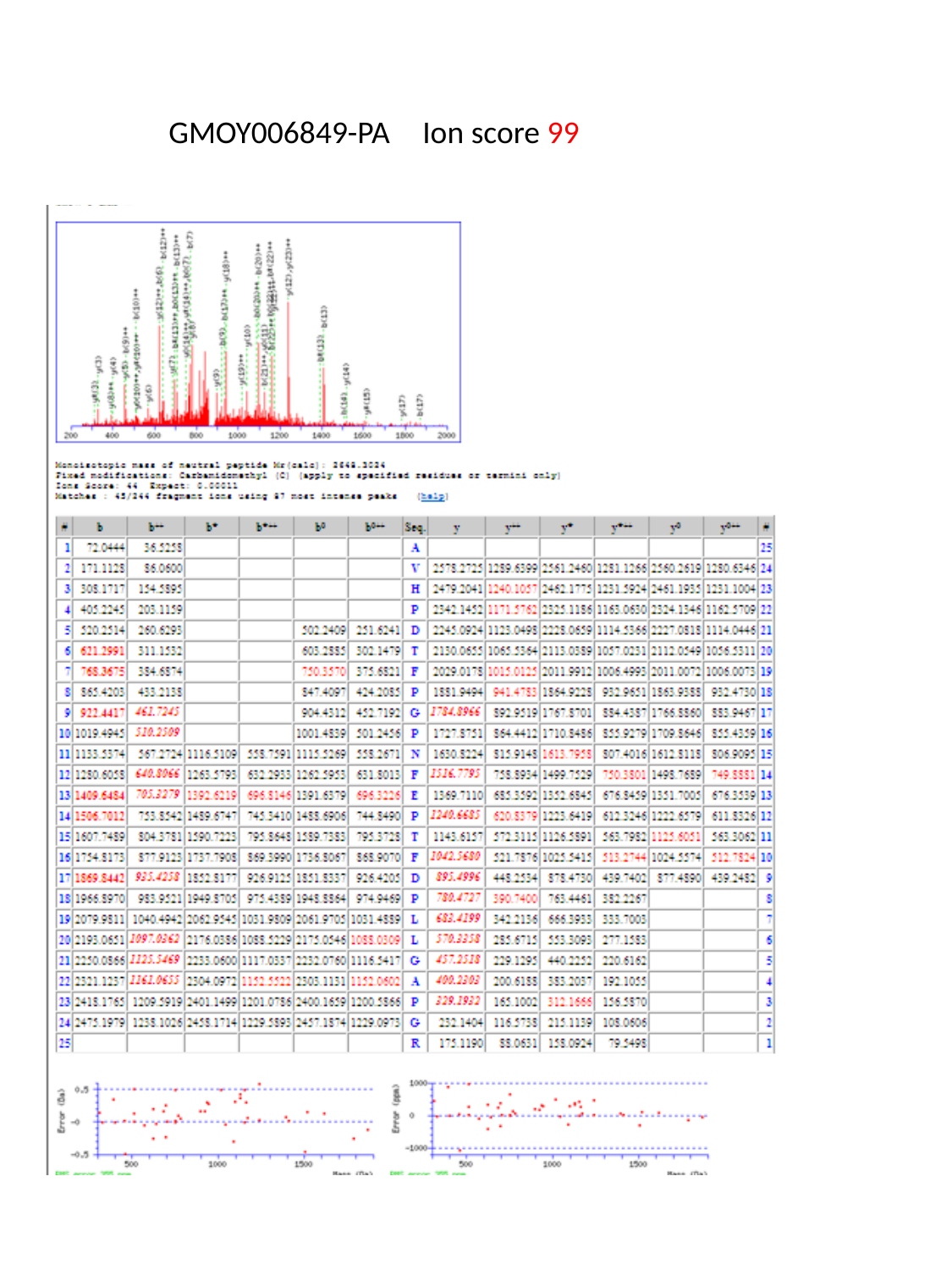

GMOY006849-PA 	Ion score 99

## Slide 130
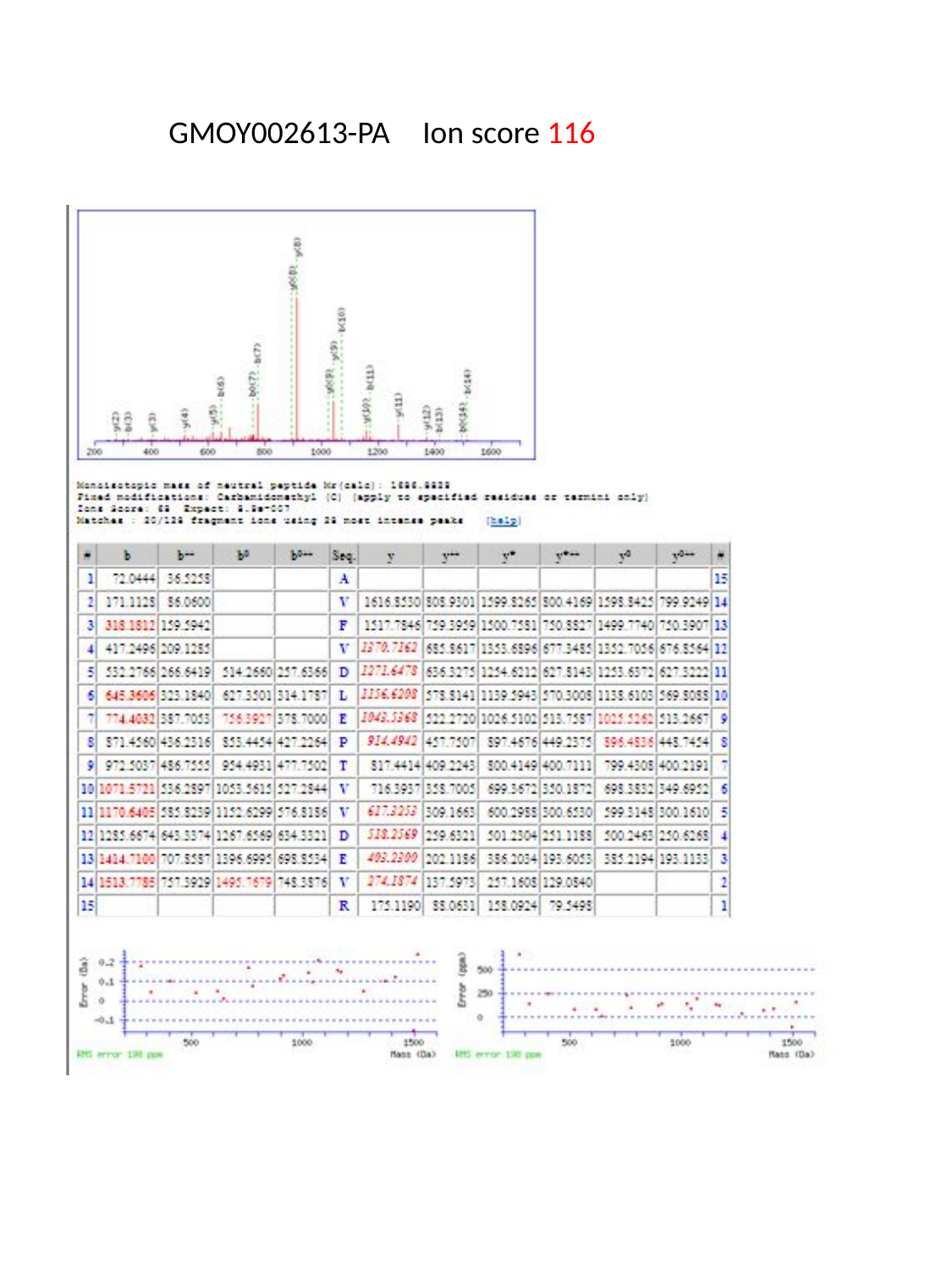

GMOY002613-PA	Ion score 116

## Slide 131
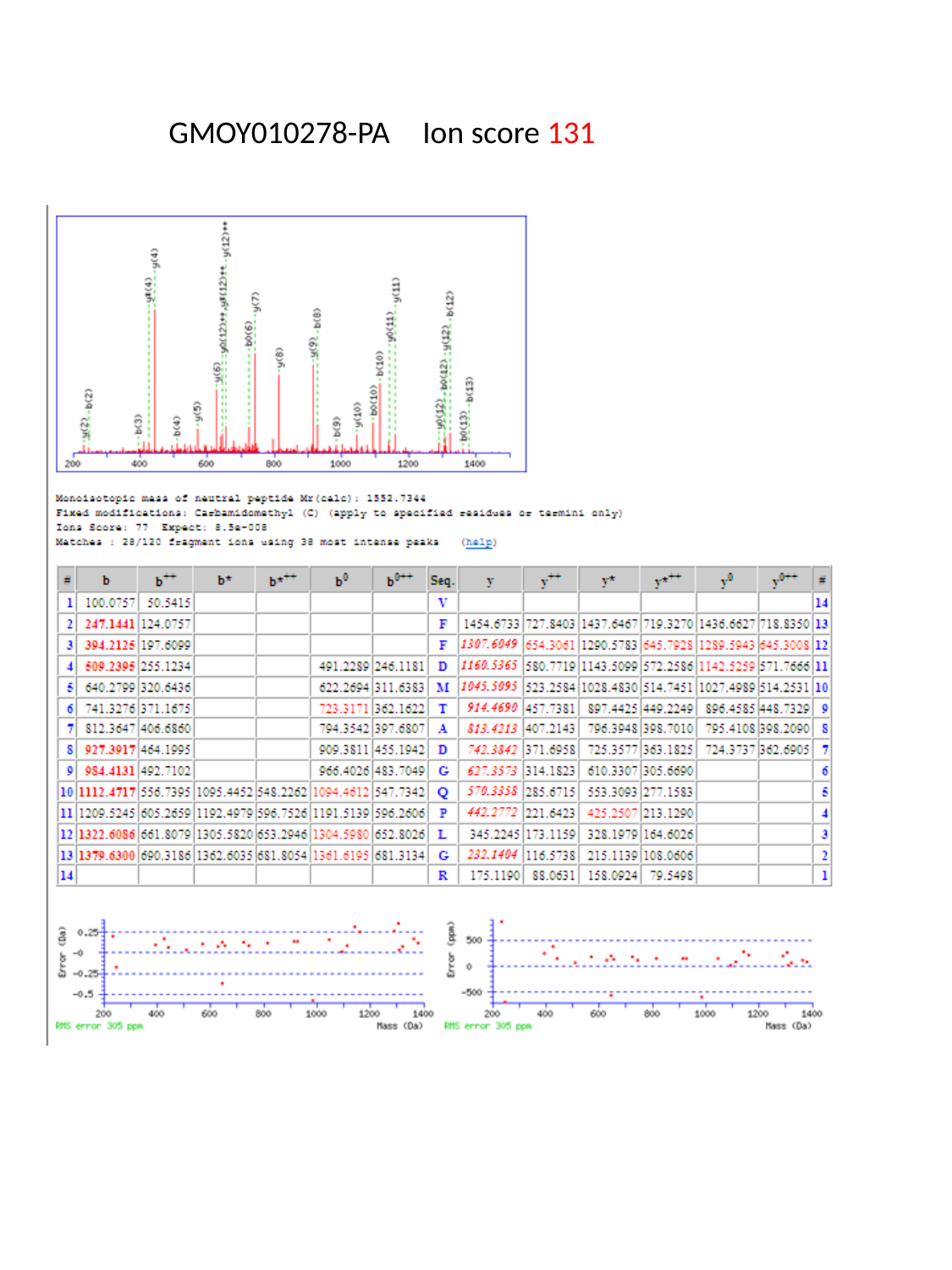

GMOY010278-PA	Ion score 131

## Slide 132
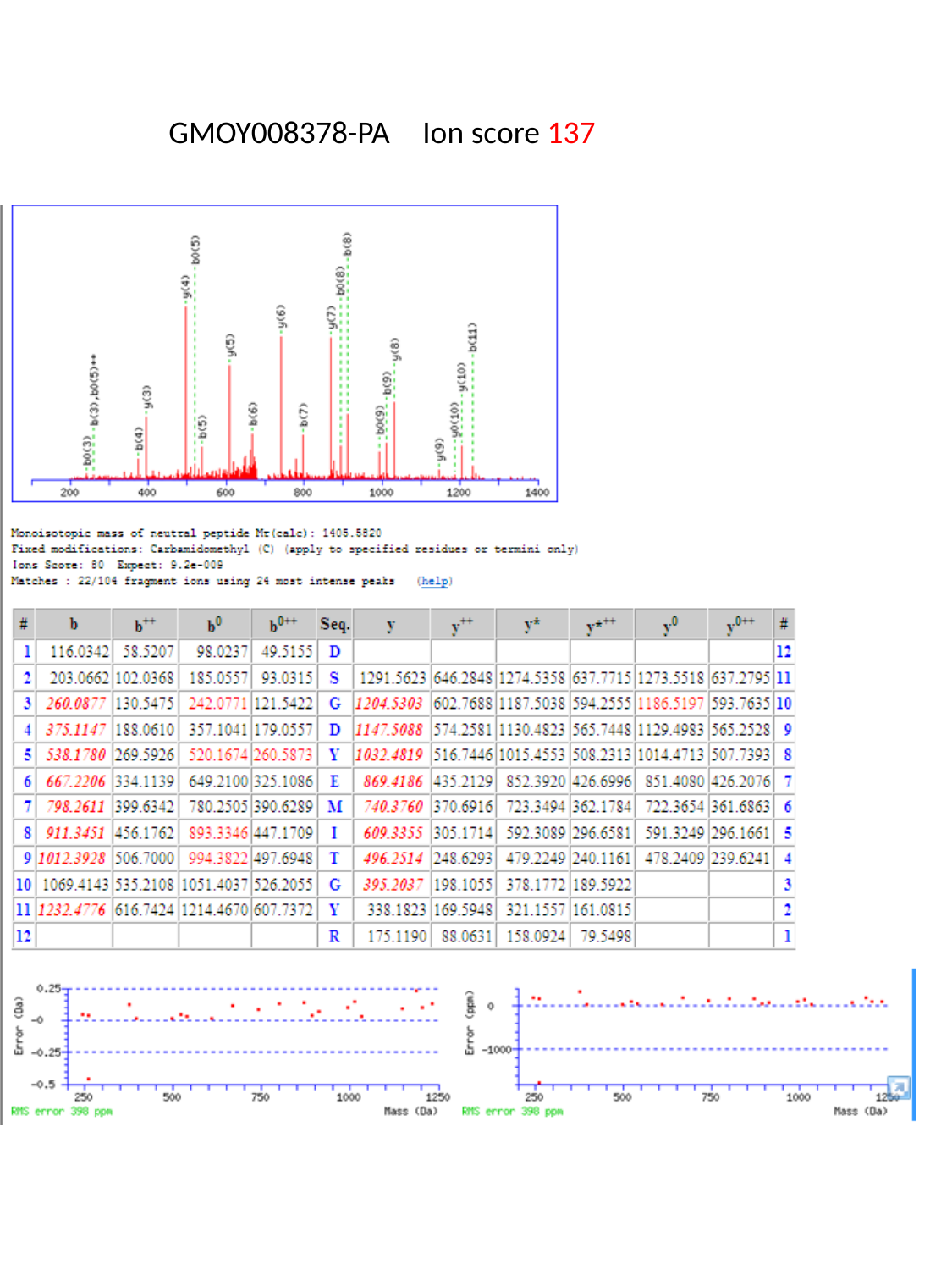

GMOY008378-PA	Ion score 137

## Slide 133
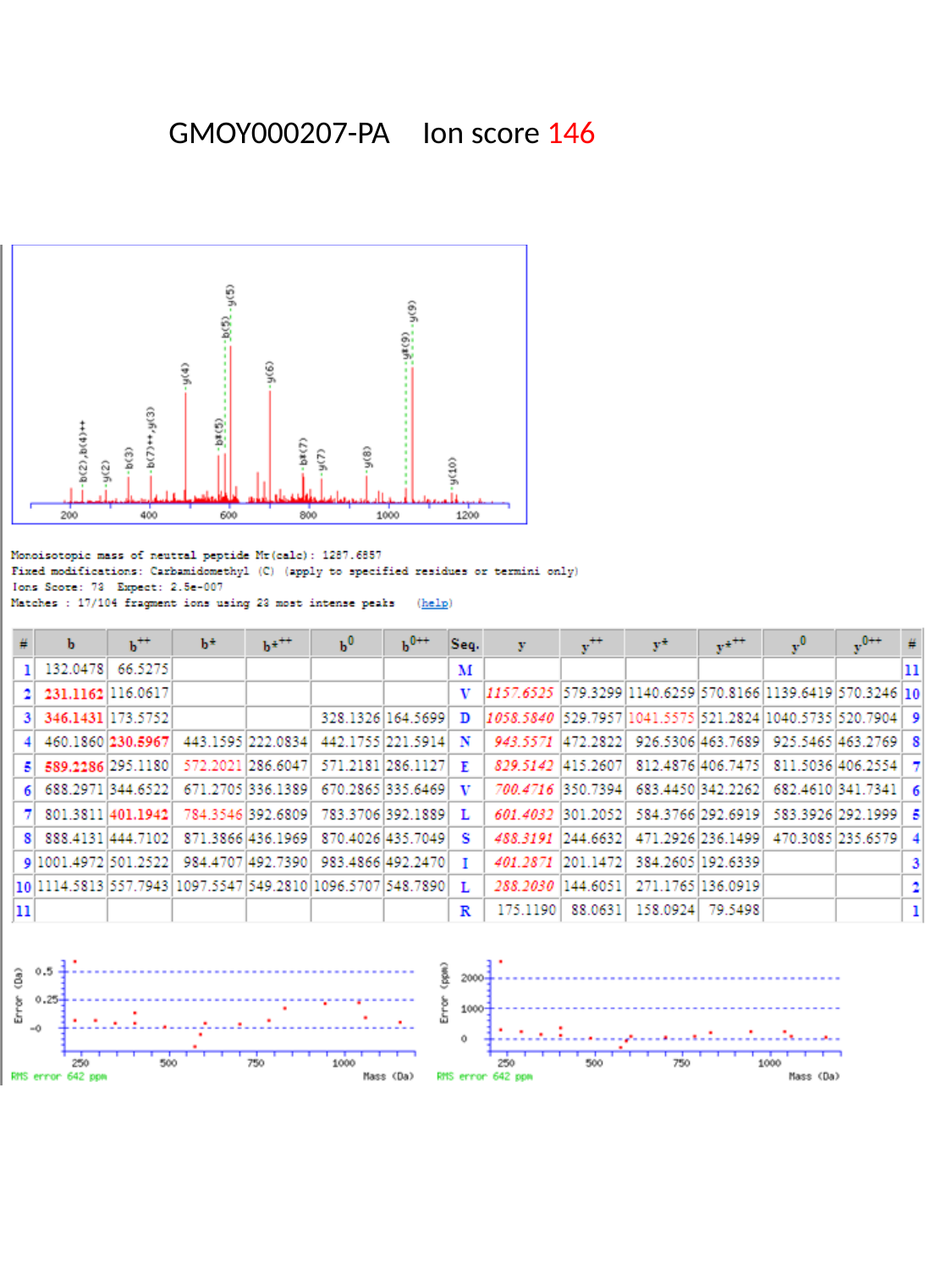

GMOY000207-PA	Ion score 146
