# Supplementary material for: An Investigation into the Protein Composition of the Teneral Glossina morsitans morsitans Peritrophic Matrix
Source: PLoS Negl Trop Dis. 2014 Apr 24;8(4):e2691. doi: 10.1371/journal.pntd.0002691 (PMC3998921; doi:10.1371/journal.pntd.0002691)
Supplement: Table S1 — List of Glossina morsitans morsitans peritrophic matrix proteins from in-gel digestion analysis (ion score cut off of 30). (DOC) [file pntd.0002691.s003.doc]

**Supporting Information**

**An investigation into the protein composition of the teneral *Glossina morsitans morsitans* peritrophic matrix.**

Clair Rose^1^, Rodrigo Belmonte^2,3^, Stuart Armstrong^4^, Gemma Molyneux^1^ Lee Haines^2^, Michael Lehane^2^, Jonathan Wastling^4^ and Alvaro Acosta-Serrano^1,2,*^

Department of ^1^Parasitology and ^2^Vector Biology, Liverpool School of Tropical Medicine, Liverpool, UK, ^4^Institute for Infection Biology, University of Liverpool, Liverpool, UK

^3^Current address: School of Biological Sciences, University of Aberdeen, Aberdeen, UK

**Table S1. List of *Glossina morsitans morsitans* peritrophic matrix proteins from in-gel digestion analysis (ion score cut off of 30)**

| Band # | Accession | Protein score | Description | # peptides | Protein Coverage | emPAI |
| --- | --- | --- | --- | --- | --- | --- |
| 1 | GMOY002708 | 279 | GmmPer66 | 3 | 6.8% | 0.17 |
|  | GMOY009892 | 36 | Dynein AAA+ ATPase | 1 | 0.3% | 0.01 |
| 2 | No sig hits | | | | | |
| 3 | GMOY002708 | 38 | GmmPer66 | 1 | 2% | 0.05 |
|  | GMOY009892 | 37 | Dynein AAA+ ATPase | 1 | 0.3% | 0.01 |
| 4 | GMOY011773 | 98 | Basement membrane-specific heparan sulfate proteoglycan core protein | 1 | 0.2% | 0.01 |
| 5 | No sig hits | | | | | |
| 6 | GMOY001776 | 99 | Actin | 2 | 5.6% | 0.19 |
|  | GMOY001773 | 68 | Basement membrane-specific heparan sulfate proteoglycan core protein | 1 | 0.3% | 0.01 |
| 7 | GMOY003590 | 61 | Collagen type IV | 1 | 0.6% | 0.02 |
|  | GMOY011773 | 60 | Basement membrane-specific heparan sulfate proteoglycan core protein | 1 | 0.2% | 0.01 |
| 8 | GMOY005703 | 748 | Myosin heavy chain | 13 | 6.3% | 0.19 |
|  | GMOY007523 | 202 | Collagen alpha-1 (IV) | 2 | 1.4% | 0.07 |
|  | GMOY011773 | 53 | Basement membrane-specific heparan sulfate proteoglycan core protein | 1 | 0.2% | 0.01 |
|  | GMOY009892 | 33 | Dynein AAA+ ATPase | 1 | 0.3% | 0.01 |
| 9 | GMOY005703 | 421 | Myosin heavy chain | 7 | 2.5% | 0.1 |
|  | GMOY007523 | 107 | Collagen alpha-1 (IV) | 1 | 0.9% | 0.02 |
|  | GMOY003579 | 73 | Sodium/potassium transporting ATPase | 1 | 1.2% | 0.04 |
|  | GMOY011773 | 70 | Basement membrane-specific heparan sulfate proteoglycan core protein | 2 | 0.4% | 0.01 |
|  | GMOY009892 | 37 | Dynein AAA+ ATPase | 1 | 0.3% | 0.01 |
| 10 | GMOY005703 | 247 | Myosin heavy chain | 4 | 1.5% | 0.06 |
|  | GMOY011773 | 35 | Basement membrane-specific heparan sulfate proteoglycan core protein | 1 | 0.2% | 0.01 |
| 11 | GMOY005703 | 342 | Myosin heavy chain | 5 | 2% | 0.07 |
|  | GMOY011773 | 79 | Basement membrane-specific heparan sulfate proteoglycan core protein | 2 | 0.4% | 0.01 |
|  | GMOY004732 | 42 | Acetyl CoA pyruvate carboxylase | 1 | 1.4% | 0.05 |
|  | GMOY009248 | 30 | Lamin | 1 | 1.6% | 0.05 |
| 12 | GMOY003759 | 275 | Sodium/potassium transporting ATPase | 4 | 5.8% | 0.16 |
|  | GMOY002708 | 157 | GmmPer66 | 2 | 4.8% | 0.11 |
|  | GMOY005703 | 118 | Myosin heavy chain | 2 | 0.7% | 0.03 |
|  | GMOY011773 | 115 | Basement membrane-specific heparan sulfate proteoglycan core protein | 3 | 0.6% | 0.02 |
|  | GMOY011520 | 112 | Alanyl aminopeptidase M1 | 3 | 1.9% | 0.05 |
|  | GMOY006600 | 103 | ATP-citrate synthase | 1 | 0.5% | 0.01 |
|  | GMOY009300 | 70 | Glutamyl aminopeptidase | 1 | 0.4% | 0.01 |
|  | GMOY001369 | 67 | Ubiquitin activating enzyme E1 | 1 | 1.2% | 0.03 |
|  | GMOY009892 | 50 | Dynein AAA+ ATPase | 1 | 0.3% | 0.01 |
|  | GMOY007294 | 45 | 2-oxoglutarate dehydrogenase | 1 | 0.9% | 0.03 |
| 13 | GMOY002377 | 518 | Paramyosin | 10 | 9.1% | 0.33 |
|  | GMOY003579 | 274 | Sodium/potassium transporting ATPase | 3 | 3.9% | 0.12 |
|  | GMOY007294 | 193 | 2-oxoglutarate dehydrogenase | 3 | 2.6% | 0.09 |
|  | GMOY011520 | 141 | Alanyl aminopeptidase M1 | 3 | 1.9% | 0.05 |
|  | GMOY003173 | 92 | Cathepsin B | 2 | 2.8% | 0.09 |
|  | GMOY005703 | 91 | Myosin heavy chain | 2 | 0.8% | 0.03 |
|  | GMOY011773 | 79 | Basement membrane-specific heparan sulfate proteoglycan core protein | 2 | 0.4% | 0.01 |
|  | GMOY004419 | 78 | Ebna-2 binding protein | 2 | 2% | 0.07 |
|  | GMOY009685 | 70 | Vinculin | 1 | 1% | 0.03 |
|  | GMOY001722 | 58 | Myosin 1B | 1 | 1% | 0.03 |
|  | GMOY001277 | 48 | Midline fasciclin | 1 | 1.2% | 0.04 |
|  | GMOY009892 | 46 | Dynein AAA+ ATPase | 1 | 0.32% | 0.01 |
|  | GMOY006959 | 39 | Carboxlyesterase | 1 | 2.4% | 0.06 |
|  | GMOY010537 | 32 | Double stranded binding protein | 1 | 1% | 0.05 |
| 14 | GMOY012011 | 176 | Hexamerin 2 | 4 | 3.5% | 0.15 |
|  | GMOY006372 | 172 | Endoplasmin | 1 | 1.7% | 0.04 |
|  | GMOY003579 | 156 | Sodium/potassium transporting ATPase | 3 | 3.9% | 0.12 |
|  | GMOY005703 | 153 | Myosin heavy chain | 4 | 1.3% | 0.06 |
|  | GMOY011773 | 106 | Basement membrane-specific heparan sulfate proteoglycan core protein | 3 | 0.6% | 0.02 |
|  | GMOY000430 | 88 | Translation elongation factor | 2 | 2.6% | 0.08 |
|  | GMOY006294 | 73 | Glutamate semialdehyde dehydrogenase | 2 | 2.9% | 0.09 |
|  | GMOY007820 | 52 | puromycin-sensitive aminopeptidase | 1 | 1% | 0.03 |
|  | GMOY002377 | 44 | Paramyosin | 1 | 1% | 0.03 |
|  | GMOY009892 | 44 | Dynein AAA+ ATPase | 1 | 0.3% | 0.01 |
|  | GMOY006871 | 34 | Dipeptidyl-peptidase | 1 | 1.8% | 0.05 |
|  | GMOY002227 | 34 | Uncharacterised/conserved/  hypothetical | 1 | 1.9% | 0.06 |
| 15 | GMOY004743 | 239 | 3-hydroxyacyl-coa dehyrogenase | 3 | 3.9% | 0.14 |
|  | GMOY003216 | 209 | Heat shock protein HSP-70 | 2 | 3.3% | 0.16 |
|  | GMOY006294 | 177 | Glutamate semialdehyde dehydrogenase | 3 | 5% | 0.14 |
|  | GMOY004375 | 145 | Heat shock protein HSP-83 | 3 | 5% | 0.14 |
|  | GMOY012139 | 124 | Heat shock protein HSP-90 | 2 | 4.1% | 0.14 |
|  | GMOY003934 | 108 | Aconitase hydratase | 3 | 3.9% | 0.11 |
|  | GMOY005703 | 102 | Myosin heavy chain | 2 | 0.7% | 0.03 |
|  | GMOY009071 | 101 | Protein transport protein (Sec23) | 1 | 2.1% | 0.04 |
|  | GMOY005958 | 72 | Prolyl endopeptidase (prolyl oligopeptidase) | 1 | 1.6% | 0.04 |
|  | GMOY008757 | 67 | Uncharacterised/conserved/  hypothetical | 2 | 1.3% | 0.04 |
|  | GMOY011773 | 62 | Basement membrane-specific heparan sulfate proteoglycan core protein | 1 | 0.2% | 0.01 |
|  | GMOY012011 | 58 | Hexamerin 2 | 1 | 1% | 0.04 |
|  | GMOY011587 | 39 | Uncharacterised/conserved/  hypothetical | 1 | 1.4% | 0.04 |
| 16 | GMOY003765 | 139 | Moesin/ezrin/radixin | 1 | 1.7% | 0.11 |
|  | GMOY009723 | 132 | Angiotensin converting enzyme | 2 | 1.2% | 0.03 |
|  | GMOY012049 | 121 | Hsc70-interacting protein | 1 | 2% | 0.05 |
|  | GMOY009493 | 105 | Heat shock protein HSP-70 | 1 | 2% | 0.05 |
|  | GMOY009357 | 97 | Succinate dehydrogenase | 1 | 3.9% | 0.12 |
|  | GMOY000743 | 76 | V-type proton ATPase catalytic subunit A (V-ATPase subunit A) | 1 | 2.4% | 0.05 |
|  | GMOY005703 | 67 | Myosin heavy chain | 2 | 0.8% | 0.03 |
|  | GMOY004228 | 63 | Transferrin | 1 | 1.8% | 0.05 |
|  | GMOY011691 | 63 | NADH-ubiquinone oxidoreductase | 1 | 1.2% | 0.04 |
|  | GMOY005442 | 55 | Lipophorin | 1 | 0.5% | 0.01 |
|  | GMOY009248 | 44 | Lamin | 1 | 1.6% | 0.05 |
|  | GMOY006294 | 40 | Glutamate semialdehyde dehydrogenase | 1 | 1.3% | 0.04 |
|  | GMOY011805 | 30 | Choline o-acyltransferase | 1 | 1.1% | 0.04 |
|  | GMOY002708 | 30 | GmmPer66 | 1 | 2% | 0.05 |
| 17 | GMOY007524 | 46 | Uncharacterised/conserved/  hypothetical | 1 | 2.1% | 0.06 |
|  | GMOY002421 | 44 | Chaperonin-60kD, ch60 | 1 | 3% | 0.06 |
|  | GMOY001034 | 30 | multiple epidermal growth factor-like domains protein 8 | 1 | 0.3% | 0.01 |
| 18 | GMOY008378 | 194 | Glutamate dehydrogenase | 1 | 20% | 1.55 |
|  | GMOY002421 | 119 | Chaperonin-60kD, ch60 | 2 | 4.6% | 0.13 |
|  | GMOY006640 | 71 | Chaperonin-60kD, ch60 | 1 | 1.6% | 0.06 |
|  | GMOY006959 | 63 | Carboxlyesterase | 1 | 2.4% | 0.09 |
|  | GMOY007353 | 60 | Glutamate dehydrogenase | 1 | 4.9% | 0.05 |
|  | GMOY002708 | 40 | GmmPer66 | 1 | 2% | 0.02 |
|  | GMOY009723 | 38 | Angiotensin converting enzyme | 1 | 0.6% | 0.04 |
|  | GMOY004611 | 36 | Vesicular transport factor dp115 | 1 | 1.1% | 0.02 |
|  | GMOY002973 | 35 | Salivary gland growth factor - 1 | 1 | 0.6% | 0.45 |
|  | GMOY005110 | 33 | Glucose-6-phosphate isomerase | 1 | 13.9% | 0.01 |
|  | GMOY003671 | 32 | Uncharacterised/conserved/  hypothetical | 1 | 0.2% | 0.05 |
|  | GMOY009248 | 30 | Lamin | 1 | 1.6% | 0.08 |
| 19 | GMOY001525 | 1212 | ATP synthase beta subunit | 7 | 21.1% | 1.88 |
|  | GMOY007817 | 141 | ATP synthase beta subunit | 1 | 2.6% | 0.10 |
|  | GMOY008764 | 703 | ATP synthase beta subunit | 10 | 20.1% | 1.2 |
|  | GMOY001949 | 61 | ATP synthase alpha subunit | 2 | 1.7% | 0.09 |
|  | GMOY002029 | 286 | ATP synthase beta subunit | 3 | 7.4% | 0.3 |
|  | GMOY012131 | 217 | Aldehyde dehydrogenase | 3 | 6.2% | 0.2 |
|  | GMOY000176 | 183 | Tubulin | 3 | 4.6% | 0.16 |
|  | GMOY000148 | 149 | Tubulin | 2 | 5.6% | 0.16 |
|  | GMOY000119 | 72 | Tubulin | 2 | 5.6% | 0.16 |
|  | GMOY011788 | 73 | Tubulin | 2 | 1.5% | 0.16 |
|  | GMOY009393 | 40 | Tubulin | 1 | 2.3% | 0.16 |
|  | GMOY002613 | 166 | Tubulin | 2 | 6.4% | 0.15 |
|  | GMOY007063 | 160 | Midgut trypsin | 1 | 5.8% | 0.29 |
|  | GMOY000153 | 139 | Chitinase Chit1 precursor | 1 | 1.7% | 0.04 |
|  | GMOY006294 | 61 | Gamma-glutamyl phosphate reductase | 1 | 1.3% | 0.04 |
|  | GMOY002358 | 47 | 80 kDa nuclear cap-binding protein | 1 | 0.9% | 0.04 |
|  | GMOY001198 | 33 | Uncharacterised/conserved/  hypothetical | 1 | 0.2% | 0.01 |
| 20 | GMOY003481 | 253 | Mitochondrial processing peptidase beta subunit | 5 | 14.1% | 0.62 |
|  | GMOY000148 | 135 | Tubulin | 1 | 3.4% | 0.07 |
|  | GMOY001525 | 126 | ATP synthase beta subunit | 1 | 5.3% | 0.14 |
|  | GMOY007817 | 0 | ATP synthase beta subunit | 1 | 2.8% | 0.14 |
|  | GMOY002708 | 121 | GmmPer66 | 2 | 4% | 0.11 |
|  | GMOY010555 | 110 | Prolylcarboxypeptidase | 2 | 9.9% | 0.14 |
|  | GMOY005872 | 108 | NADH-ubiquinone oxidoreductase fe-s protein 2 | 1 | 5.2% | 0.07 |
|  | GMOY002199 | 100 | Translation elongation factor EF-1 alpha/Tu | 1 | 2.4% | 0.07 |
|  | GMOY001831 | 84 | Bleomycin hydrolase | 1 | 2.9% | 0.05 |
|  | GMOY005793 | 79 | Aspartate ammonia lyase | 1 | 2.8% | 0.07 |
|  | GMOY001776 | 53 | Actin | 1 | 2.9% | 0.09 |
|  | GMOY008484 | 42 | cAMP-dependent protein kinase type ii regulatory subunit | 2 | 3.2% | 0.22 |
|  | GMOY009892 | 42 | Dynein AAA+ ATPase | 1 | 1.1% | 0.01 |
|  | GMOY005026 | 0 | Dynein AAA+ ATPase | 0 | 0.4% | 0.01 |
|  | GMOY001824 | 36 | NADH-ubiquinone oxidoreductase flavoprotein 1 | 1 | 4.9% | 0.07 |
|  | GMOY007398 | 33 | Small GTPase | 1 | 1.8% | 0.09 |
|  | GMOY008764 | 33 | ATP synthase beta subunit | 1 | 3.3% | 0.06 |
|  | GMOY007524 | 31 | Uncharacterised/conserved/  hypothetical | 1 | 2.1% | 0.06 |
|  | GMOY011805 | 30 | Choline o-acyltransferase | 1 | 1.1% | 0.04 |
|  | GMOY009459 | 28 | Uncharacterised/conserved/  hypothetical | 1 | 2.9% | 0.09 |
|  | GMOY001198 | 22 | Uncharacterised/conserved/  hypothetical | 1 | 1.9% | 0.01 |
|  | GMOY005840 | 22 | Uncharacterised/conserved/  hypothetical | 1 | 8% | 0.24 |
|  | GMOY006209 | 18 | Gustatory receptor Gr19 | 1 | 3.2% | 0.08 |
|  | GMOY003284 | 14 | Hsp70-interacting protein, putative | 1 | 5.9% | 0.11 |
| 21 | GMOY002708 | 137 | GmmPer66 | 1 | 2% | 0.05 |
|  | GMOY006991 | 94 | Tubulointerstitial nephritis antigen | 2 | 4.6% | 0.24 |
|  | GMOY003513 | 89 | Citrate synthase 1 | 1 | 3% | 0.07 |
|  | GMOY004931 | 71 | NADP-dependent isocitrate dehydrogenase | 1 | 0.6% | 0.02 |
|  | GMOY003390 | 55 | NADH-ubiquinone oxidoreductase NDUFA10/42kDa subunit | 2 | 4.8% | 0.17 |
|  | GMOY003287 | 49 | Initiation factor 4a | 1 | 1.9% | 0.06 |
|  | GMOY000153 | 46 | Chitinase Chit1 precursor | 1 | 1.3% | 0.04 |
|  | GMOY001776 | 46 | Actin | 1 | 2.9% | 0.09 |
|  | GMOY009892 | 41 | Dynein AAA+ ATPase | 1 | 0.3% | 0.01 |
|  | GMOY000849 | 40 | Uncharacterised/conserved/  hypothetical | 1 | 0.9% | 0.03 |
|  | GMOY003934 | 37 | Aconitase | 1 | 1.3% | 0.04 |
|  | GMOY010103 | 30 | Aspartyl protease | 1 | 1.8% | 0.09 |
| 22 | GMOY003315 | 964 | Actin 87e | 9 | 28.7% | 1.78 |
|  | GMOY001776 | 890 | Actin | 8 | 25.3% | 1.78 |
|  | GMOY007085 | 900 | Actin 5c | 8 | 22.6% | 1.09 |
|  | GMOY007620 | 189 | NADH-ubiquinone oxidoreductase | 2 | 4.8% | 0.15 |
|  | GMOY007118 | 100 | NADH-ubiquinone oxidoreductase | 1 | 2.4% | 0.15 |
|  | GMOY000153 | 160 | Chitinase Chit1 precursor | 2 | 3% | 0.08 |
|  | GMOY002708 | 116 | GmmPer66 | 2 | 4% | 0.11 |
|  | GMOY003176 | 100 | Myofilin | 1 | 3.3% | 0.09 |
|  | GMOY002792 | 95 | Acetyl-CoA acyltransferase 2 | 2 | 4.4% | 0.16 |
|  | GMOY008764 | 83 | ATP synthase alpha subunit | 1 | 2.9% | 0.06 |
|  | GMOY001238 | 45 | Gelsolin | 1 | 1% | 0.04 |
|  | GMOY002867 | 42 | Glutaryl-CoA dehydrogenase | 2 | 4.8% | 0.17 |
|  | GMOY006490 | 40 | Ionotropic glutamate receptor | 1 | 0.9% | 0.03 |
|  | GMOY009892 | 38 | Dynein AAA+ ATPase | 1 | 0.3% | 0.01 |
|  | GMOY003929 | 30 | Uncharacterised/conserved/  hypothetical | 1 | 1.1% | 0.03 |
| 23 | GMOY002708 | 209 | GmmPer66 | 2 | 4.8% | 0.11 |
|  | GMOY001776 | 153 | Actin | 2 | 5.6% | 0.19 |
|  | GMOY011652 | 124 | NAD-dependent malate dehydrogenase | 2 | 6.8% | 0.22 |
|  | GMOY003176 | 120 | Uncharacterised/conserved/  hypothetical | 2 | 7% | 0.19 |
|  | GMOY000473 | 115 | Glyceraldehyde-3-phosphate dehydrogenase | 1 | 4.2% | 0.1 |
|  | GMOY003306 | 103 | TsetseEP | 1 | 3.5% | 0.11 |
|  | GMOY001525 | 80 | ATP synthase beta subunit | 1 | 3.7% | 0.07 |
|  | GMOY008627 | 64 | Uncharacterised/conserved/  hypothetical | 1 | 5.4% | 0.17 |
|  | GMOY002443 | 57 | Serpin 2 | 1 | 2.8% | 0.06 |
|  | GMOY009603 | 54 | Aldehyde reductase | 1 | 1.6% | 0.04 |
|  | GMOY002110 | 46 | Aldehyde reductase | 1 | 3.9% | 0.12 |
|  | GMOY009892 | 40 | Dynein AAA+ ATPase | 1 | 0.3% | 0.01 |
|  | GMOY007736 | 32 | Arginine kinase | 1 | 1.55% | 0.03 |
| 24 | GMOY009756 | 286 | Trypsin/PRO3 | 5 | 6.1% | 0.28 |
|  | GMOY011652 | 157 | NAD-dependent malate dehydrogenase | 3 | 8.9% | 0.35 |
|  | GMOY002634 | 120 | Pyrroline-5-carboxylate reductase | 1 | 4.8% | 0.13 |
|  | GMOY001776 | 104 | Actin | 1 | 2.7% | 0.09 |
|  | GMOY011773 | 92 | Basement membrane-specific heparan sulfate proteoglycan core protein | 1 | 0.2% | 0.01 |
|  | GMOY004246 | 78 | Zinc carboxypeptidase | 1 | 2.2% | 0.08 |
|  | GMOY003306 | 77 | TsetseEP | 1 | 4.8% | 0.11 |
|  | GMOY001525 | 77 | ATP synthase beta subunit | 1 | 2.8% | 0.07 |
|  | GMOY011932 | 68 | Glycosyltransferase | 1 | 1.6% | 0.04 |
|  | GMOY000582 | 61 | ATP synthase gamma subunit | 1 | 3.6% | 0.1 |
|  | GMOY008764 | 59 | ATP synthase alpha subunit | 1 | 2.9% | 0.06 |
|  | GMOY000672 | 51 | Serine protease 6 | 1 | 4.9% | 0.12 |
|  | GMOY002708 | 48 | GmmPer66 | 1 | 2% | 0.05 |
|  | GMOY007063 | 47 | Midgut trypsin | 1 | 3.5% | 0.14 |
|  | GMOY009531 | 45 | Uncharacterised/conserved/  hypothetical | 1 | 2.9% | 0.07 |
|  | GMOY008627 | 41 | Uncharacterised/conserved/  hypothetical | 1 | 5.4% | 0.17 |
|  | GMOY009892 | 34 | Dynein AAA+ ATPase | 1 | 0.3% | 0.01 |
|  | GMOY009575 | 34 | Annexin x | 1 | 2.8% | 0.11 |
|  | GMOY009975 | 34 | Annexin | 1 | 3.1% | 0.1 |
|  | GMOY005442 | 33 | Lipophorin | 1 | 0.3% | 0.01 |
|  | GMOY009145 | 33 | Prohibitin-like protein | 1 | 2.1% | 0.1 |
|  | GMOY011410 | 31 | Secreted protein | 1 | 3.1% | 0.15 |
| 25 | GMOY008707 | 302 | Uncharacterised/conserved/  hypothetical | 3 | 6.8% | 0.33 |
|  | GMOY002634 | 265 | Pyrroline-5-carboxylate reductase | 2 | 9.9% | 0.45 |
|  | GMOY009756 | 236 | Trypsin/PRO3 | 3 | 3.7% | 0.18 |
|  | GMOY006034 | 204 | ADP/ATP carrier protein | 7 | 20% | 1.06 |
|  | GMOY010142 | 151 | Serine protease | 1 | 4.9% | 0.11 |
|  | GMOY001776 | 150 | Actin | 2 | 5.6% | 0.19 |
|  | GMOY006173 | 136 | 14-3-3 protein zeta | 2 | 10.1% | 0.29 |
|  | GMOY007699 | 124 | 40S ribosomal protein S3 | 1 | 4.5% | 0.11 |
|  | GMOY011657 | 100 | 14-3-3 protein | 1 | 4.3% | 0.13 |
|  | GMOY000582 | 83 | ATP synthase gamma subunit | 2 | 6.5% | 0.33 |
|  | GMOY010218 | 72 | Ribosomal protein L7 | 2 | 8.2% | 0.27 |
|  | GMOY000508 | 76 | Phosphate carrier protein | 1 | 3.4% | 0.1 |
|  | GMOY005492 | 55 | Prohibitin | 1 | 2.9% | 0.12 |
|  | GMOY007346 | 51 | Electron transfer flavoprotein beta subunit | 1 | 4.7% | 0.14 |
|  | GMOY009757 | 48 | Serine type endopeptidase | 1 | 3.6% | 0.12 |
|  | GMOY001290 | 45 | Proteasome alpha subunit | 1 | 3.6% | 0.13 |
|  | GMOY002729 | 40 | Serine protease 1 | 1 | 0.8% | 0.03 |
|  | GMOY007063 | 40 | Midgut trypsin | 1 | 3.5% | 0.14 |
|  | GMOY000234 | 37 | Acyl-CoA synthetase | 1 | 1.1% | 0.04 |
|  | GMOY003090 | 35 | Voltage-dependent anion-selective channel | 1 | 3.5% | 0.12 |
|  | GMOY005442 | 33 | Lipophorin | 1 | 0.3% | 0.01 |
| 26 | GMOY006839 | 245 | Porin | 3 | 23.7% | 0.8 |
|  | GMOY011770 | 226 | Hydroxyacyl dehydrogenase | 1 | 6.7% | 0.3 |
|  | GMOY007063 | 162 | Midgut trypsin | 2 | 7.8% | 0.29 |
|  | GMOY001776 | 129 | Actin | 2 | 8.5% | 0.19 |
|  | GMOY009757 | 123 | Serine type endopeptidase | 2 | 7.2% | 0.26 |
|  | GMOY007346 | 104 | Electron transfer flavoprotein beta subunit | 2 | 10.7% | 0.3 |
|  | GMOY011979 | 95 | Vacuolar H+-ATPase v1 sector subunit E | 2 | 11.9% | 0.31 |
|  | GMOY010142 | 94 | Midgut trypsin | 1 | 4.9% | 0.11 |
|  | GMOY007954 | 88 | Phosphoglycerate mutase | 1 | 3.6% | 0.11 |
|  | GMOY001901 | 86 | Fibrinogen-related/angiopoietin-like protein | 2 | 6.0% | 0.2 |
|  | GMOY000701 | 85 | NADH:ubiquinone reductase | 1 | 5.6% | 0.15 |
|  | GMOY000816 | 78 | NADH dehydrogenase iron-sulfur protein 3 | 2 | 12.3% | 0.27 |
|  | GMOY002708 | 63 | GmmPer66 | 1 | 2.0% | 0.05 |
|  | GMOY009373 | 56 | Glutathione S transferase S1 | 1 | 9.8% | 0.14 |
|  | GMOY002950 | 52 | Antigen-5 precursor | 1 | 4.2% | 0.13 |
|  | GMOY009756 | 50 | Trypsin/PRO3 | 1 | 1.1% | 0.04 |
|  | GMOY011554 | 50 | Myosin light chain 2 | 1 | 10.4% | 0.14 |
|  | GMOY003176 | 48 | Myofilin | 1 | 3.3% | 0.09 |
|  | GMOY003678 | 43 | Proteasome alpha subunit | 1 | 4.1% | 0.14 |
|  | GMOY003659 | 39 | Succinate dehydrogenase | 2 | 10.5% | 0.22 |
|  | GMOY012309 | 38 | Lethal 2 essential for life/Hsp 20 | 1 | 8.4% | 0.25 |
|  | GMOY009892 | 35 | Dynein AAA+ ATPase | 1 | 1.4% | 0.01 |
|  | GMOY004379 | 32 | ATP synthase subunit b | 1 | 10.6% | 0.14 |
|  | GMOY011805 | 32 | Choline o-acyltransferase | 1 | 1.1% | 0.04 |
| 27 | GMOY007063 | 690 | Midgut trypsin | 7 | 23.6% | 3.07 |
|  | GMOY006839 | 157 | Porin | 2 | 8% | 0.42 |
|  | GMOY001776 | 97 | Actin | 1 | 2.7% | 0.09 |
|  | GMOY004379 | 40 | ATP synthase subunit b | 1 | 3.8% | 0.14 |
|  | GMOY004187 | 39 | Uncharacterised/conserved/  hypothetical | 1 | 1% | 0.05 |
|  | GMOY006034 | 39 | ADP/ATP carrier protein | 1 | 2.5% | 0.11 |
|  | GMOY007742 | 35 | Uncharacterised/conserved/  hypothetical | 1 | 0.3% | 0.01 |
|  | GMOY011805 | 30 | Choline o-acyltransferase | 1 | 1.1% | 0.04 |
| 28 | GMOY007063 | 327 | Midgut trypsin | 5 | 19% | 1.44 |
|  | GMOY001195 | 321 | Uncharacterised/conserved/  hypothetical | 4 | 20.5% | 0.99 |
|  | GMOY008040 | 143 | Thioredoxin | 3 | 17% | 0.61 |
|  | GMOY002708 | 79 | GmmPer66 | 1 | 2% | 0.05 |
|  | GMOY009756 | 79 | Trypsin/PRO3 | 1 | 1.8% | 0.04 |
|  | GMOY000672 | 60 | Serine protease 6 | 1 | 4.9% | 0.12 |
|  | GMOY007847 | 41 | Uncharacterised/conserved/  hypothetical | 1 | 4.9% | 0.17 |
|  | GMOY011805 | 39 | Choline o-acyltransferase | 1 | 1.1% | 0.04 |
|  | GMOY005801 | 35 | Microtubule associated protein xmap215 | 1 | 0.4% | 0.02 |
| 29 | GMOY009744 | 251 | Uncharacterised/conserved/  hypothetical | 3 | 15% | 0.5 |
|  | GMOY002708 | 194 | GmmPer66 | 3 | 6.8% | 0.17 |
|  | GMOY010278 | 130 | Cyclophilin type peptidyl-prolyl cis-trans isomerase | 1 | 4.8% | 0.1 |
|  | GMOY000466 | 124 | C-type lectin | 1 | 5.3% | 0.13 |
|  | GMOY007063 | 122 | Midgut trypsin | 1 | 3.5% | 0.14 |
|  | GMOY009587 | 107 | PRO2 | 1 | 9.5% | 0.29 |
|  | GMOY011263 | 51 | Lethal 2 35Di/Hsp 20 | 1 | 9.9% | 0.21 |
|  | GMOY005682 | 48 | Myosin light chain | 1 | 5.8% | 0.22 |
|  | GMOY007847 | 36 | Uncharacterised/conserved/  hypothetical | 1 | 4.9% | 0.17 |
|  | GMOY003850 | 32 | ADP ribosylation factor 79F | 1 | 5.5% | 0.18 |
| 30 | GMOY000747 | 240 | Nucleoside diphosphate kinase | 2 | 12.6% | 0.6 |
|  | GMOY010846 | 169 | 40S ribosomal protein S23 | 1 | 14% | 0.24 |
|  | GMOY002708 | 100 | GmmPer66 | 2 | 4% | 0.11 |
|  | GMOY010278 | 97 | Cyclophilin type peptidyl-prolyl *cis*-trans isomerase | 1 | 4.2% | 0.1 |
|  | GMOY007063 | 95 | Midgut trypsin | 1 | 3.5% | 0.14 |
|  | GMOY009587 | 56 | PRO2 | 1 | 9.5% | 0.29 |
|  | GMOY008757 | 51 | Uncharacterised/conserved/  hypothetical | 1 | 1.5% | 0.02 |
|  | GMOY011263 | 49 | Lethal 2 35Di/Hsp 20 | 1 | 9.9% | 0.21 |
|  | GMOY002617 | 47 | Uncharacterised/conserved/  hypothetical | 1 | 4.6% | 0.23 |
|  | GMOY004802 | 36 | Uncharacterised/conserved/  hypothetical | 1 | 9.9% | 0.24 |
|  | GMOY011805 | 30 | Choline O-acyltransferase | 1 | 1.1% | 0.04 |
