# Supplementary material for: An Investigation into the Protein Composition of the Teneral Glossina morsitans morsitans Peritrophic Matrix
Source: PLoS Negl Trop Dis. 2014 Apr 24;8(4):e2691. doi: 10.1371/journal.pntd.0002691 (PMC3998921; doi:10.1371/journal.pntd.0002691)
Supplement: Table S2 — List of Sodalis glossinidus proteins associated with the Glossina morsitans morsitans peritrophic matrix. (DOCX) [file pntd.0002691.s004.docx]

**Supporting Information**

**An investigation into the protein composition of the teneral *Glossina morsitans morsitans* peritrophic matrix.**

Clair Rose^1^, Rodrigo Belmonte^2,3^, Stuart Armstrong^4^, Gemma Molyneux^1^ Lee Haines^2^, Michael Lehane^2^, Jonathan Wastling^4^ and Alvaro Acosta-Serrano^1,2,*^

Department of ^1^Parasitology and ^2^Vector Biology, Liverpool School of Tropical Medicine, Liverpool, UK, ^4^Institute for Infection Biology, University of Liverpool, Liverpool, UK

^3^Current address: School of Biological Sciences, University of Aberdeen, Aberdeen, UK

**Table S2.** List of *Sodalis glossinidus* proteins associated with the *Glossina morsitans morsitans* peritrophic matrix

| **Protein ID^1^** | **Protein Name** | **In-gel^2^**  **(# peptides/**  **Band No)** | **In-solution**  **(# peptides)** |
| --- | --- | --- | --- |
| SG0306 | GroEL 60 kDa chaperonin**^3^** | (1) | (3) |
| SG0058 | FliC flagellin | N.D.**^4^** | (2) |
| SG1007 | OmpF outer membrane pore protein**^5^** | N.D. | (2) |
| SG1030 | OmpA outer membrane protein A**^5^** | N.D. | (2) |
| SG1474 | Putative chitinase | N.D. | (2) |
| SG2412 | AtpA ATP synthase subunit alpha | (2) | (1) |
| SG0131 | RplA 50S ribosomal protein L1 | N.D. | (1) |
| SG0127 | Tuf Elongation factor Tu (EF-Tu) | (1) | (1) |
| SG1759 | HisS Histidyl-tRNA synthetase | (1) | (1) |
| Ps_SGL0763 | Putative phage integrase | (1) | (1) |
| SG0134 | RpoB DNA-directed RNA Pol subunit beta | N.D. | (1) |
| SG0135 | RpoC1 DNA directed RNA Pol subunit gamma | N.D. | (1) |
| SG2230 | GlnA glutamine synthetase | N.D. | (1) |
| SG1265 | MsbB lipid A biosynthesis acyltransferase | N.D. | (1) |
| SG0887 | Pal peptidoglycan-associated lipoprotein precursor | (1) | (1) |
| Ps_SGL0342c | Putative exported protein | (1) | (1) |
| SG1015 | AsnS asparaginyl-tRNA synthetase |  | (1) |
| SG0430 | ATP dependent helicase HepA | (2) |  |
| Ps_SGL0112c | Periplasmic trehalase precursor | (2) |  |
| SG0482 | Hypoxanthine phosphoribosyltransferase | (1) |  |
| Ps_SGL1307c | CheA chemotaxis protein | (1) |  |
| SG0955 | LysP lysine specific permease/transporter | (2) |  |
| SG0087 | RpoH RNA polymerase factor sigma 23 | (1) |  |
| SG2405 | tRNA uridine 5-carboxymethylaminomethyl modification protein GidA | (1) |  |
| SG0284 | N-acylhomoserine lactone synthase Ypel1 | (1) |  |
| SG1864 | Multidrug efflux transport protein | (1) |  |
| Ps_SGL1035c | Putative N-ethylmaleimide reductase | (1) |  |

**^1^**Genome downloaded from Eugeni, E. et al., (2010). doi:10.1186/1471-2164-11-449

**^2^**As shown in Fig 1B

**^3^**Its presence in the PM was validated by Western blotting (Figure 3)

**^4^**Not detected

**^5^**The presence of both OMP isoforms has been also detected by Western blotting using an anti *Sodalis* OMP polyclonal serum (Rose C., unpublished).
